# Heterogeneous Rhodium Single-Atom-Site Catalyst Enables Chemoselective Carbene N–H Bond Insertion

**DOI:** 10.1021/jacs.4c01408

**Published:** 2024-04-07

**Authors:** Yuanjun Chen, Ruixue Zhang, Zhiwen Chen, Jiangwen Liao, Xuedong Song, Xiao Liang, Yu Wang, Juncai Dong, Chandra Veer Singh, Dingsheng Wang, Yadong Li, F. Dean Toste, Jie Zhao

**Affiliations:** † Key Laboratory for Advanced Materials and Joint International Research Laboratory of Precision Chemistry and Molecular Engineering, Feringa Nobel Prize Scientist Joint Research Center, Frontiers Science Center for Materiobiology and Dynamic Chemistry, School of Chemistry and Molecular Engineering, East China University of Science and Technology, Shanghai, 200237, People’s Republic of China; ‡ Department of Chemistry, Tsinghua University, Beijing, 100084, People’s Republic of China; § Department of Materials Science and Engineering, University of Toronto, Toronto, Ontario M5S3E4, Canada; ∥ Beijing Synchrotron Radiation Facility, Institute of High Energy Physics, Chinese Academy of Sciences, Beijing 100049, People’s Republic of China; ⊥ Shanghai Synchrotron Radiation Facility, Zhangjiang Laboratory, Shanghai Advanced Research Institute, Chinese Academy of Sciences, Shanghai, 201204, People’s Republic of China; # Chemical Science Division, Lawrence Berkeley National Laboratory, Berkeley, California 94720, United States; 7 Department of Chemistry, University of California, Berkeley, California 94720, United States

## Abstract

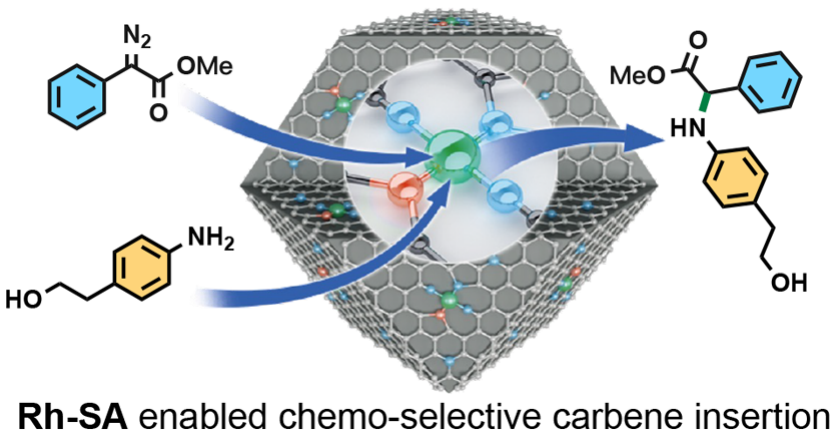

Transition-metal-catalyzed carbene insertion reactions of a nitrogen–hydrogen bond have emerged as robust and versatile methods for the construction of C–N bonds. While significant progress of homogeneous catalytic metal carbene N–H insertions has been achieved, the control of chemoselectivity in the field remains challenging due to the high electrophilicity of the metal carbene intermediates. Herein, we present an efficient strategy for the synthesis of a rhodium single-atom-site catalyst (Rh-SA) that incorporates a Rh atom surrounded by three nitrogen atoms and one phosphorus atom doped in a carbon support. This Rh-SA catalyst, with a catalyst loading of only 0.15 mol %, exhibited exceptional catalytic performance for heterogeneous carbene insertion with various anilines and heteroaryl amines in combination with diazo esters. Importantly, the heterogeneous catalyst selectively transformed aniline derivatives bearing multiple nucleophilic moieties into single N–H insertion isomers, while the popular homogeneous Rh_2_(OAc)_4_ catalyst produced a mixture of overfunctionalized side products. Additionally, similar selectivities for N–H bond insertion with a set of stereoelectronically diverse diazo esters were obtained, highlighting the general applicability of this heterogeneous catalysis approach. On the basis of density functional theory calculations, the observed selectivity of the Rh-SA catalyst was attributed to the insertion barriers and the accelerated proton transfer assisted by the phosphorus atom in the support. Overall, this investigation of heterogeneous metal-catalyzed carbene insertion underscores the potential of single-atom-site catalysis as a powerful and complementary tool in organic synthesis.

## Introduction

Recent advancements in material science and characterization technologies have fueled growing interest in the development of heterogenized organic reactions using new catalytically active materials.^1−13^ Among these materials, single-atom-site catalysts (SACs) have emerged as a versatile class of potential heterogeneous catalysts due to their exceptional atom-utilization efficiency and fully exposed active sites.^14−25^ The inherent homogeneity in the geometric and tunable electronic structures of SACs has garnered significant attention. Similar to homogeneous catalysts, precise modifications of the material support of SACs enable the fine-tuning of both selectivity and reactivity. Consequently, understanding the structural correlation of SACs and their catalytic performance is crucial to the rational design of SACs at the atomic level to target specific reaction outcomes.^14,17^ Notably, over the past decade, SACs have demonstrated their potential in a wide range of heterogeneous reactions, including a spectrum of heterogeneous hydroboration, hydrosilylation, and hydrogenation of alkenes and alkynes.^26−45^ The promising results achieved thus far motivate further exploration into the application of SACs toward expanding the scope and diversity of organic transformations promoted by heterogeneous catalysts.

Transition-metal-catalyzed carbene N–H insertions represent a powerful strategy for constructing C–N bonds, which are commonly found in pharmaceuticals and natural products.^46−59^ By employing an appropriate transition-metal catalyst, diazo ester can be readily decomposed into a metal carbene intermediate, enabling nucleophilic addition of amines to the electrophilic carbon center.^46,60−62^ The outcome of this reaction is effectively controlled by the combination of transition metals with ligands that possesses distinct stereoelectronic properties. Notably, ligand selection on homogeneous Rh and Cu complexes allows for the modulation of the electrophilicity of the metal carbene intermediate, leading to selective carbene insertion reactions of C–H bonds.^63−68^ In contrast, the development of a highly selective metal carbene N–H insertion reaction for substrates featuring multiple reactive sites remains limited.^69^ This challenge can, in part, be attributed to the high electrophilicity of metal carbene species, resulting in unselective reactions.

In recent experiments, we successfully demonstrated the effectiveness of a heterogeneous single-atom iridium catalyst (Ir-SA) for site-selective carbene insertion into O–H bonds.^70^ To achieve high selectivity, it was crucial to engineer the appropriate material support for the SACs, which altered the oxidant state and the electron density of the metal center. Building on these promising results, we aimed to develop a novel platform unprecedented for SACs for chemoselective carbene N–H insertion reactions. We hypothesized that incorporating a phosphorus atom in the nitrogen-coordinating module of SACs, resulting in a more electron-rich system, could lower the electrophilicity of metal carbene. We envisioned that the reduced electrophilicity would enable the heterogeneous catalysis of chemoselective N–H insertion reactions.

Based on these hypotheses, we initiated the exploration of P-doped SAC catalyst Rh**-**SA and its application in the aforementioned selective heterogeneous catalysis.

## Results and Discussion

### Preparation and Characterization of Rh-SA

**Figure 1:**
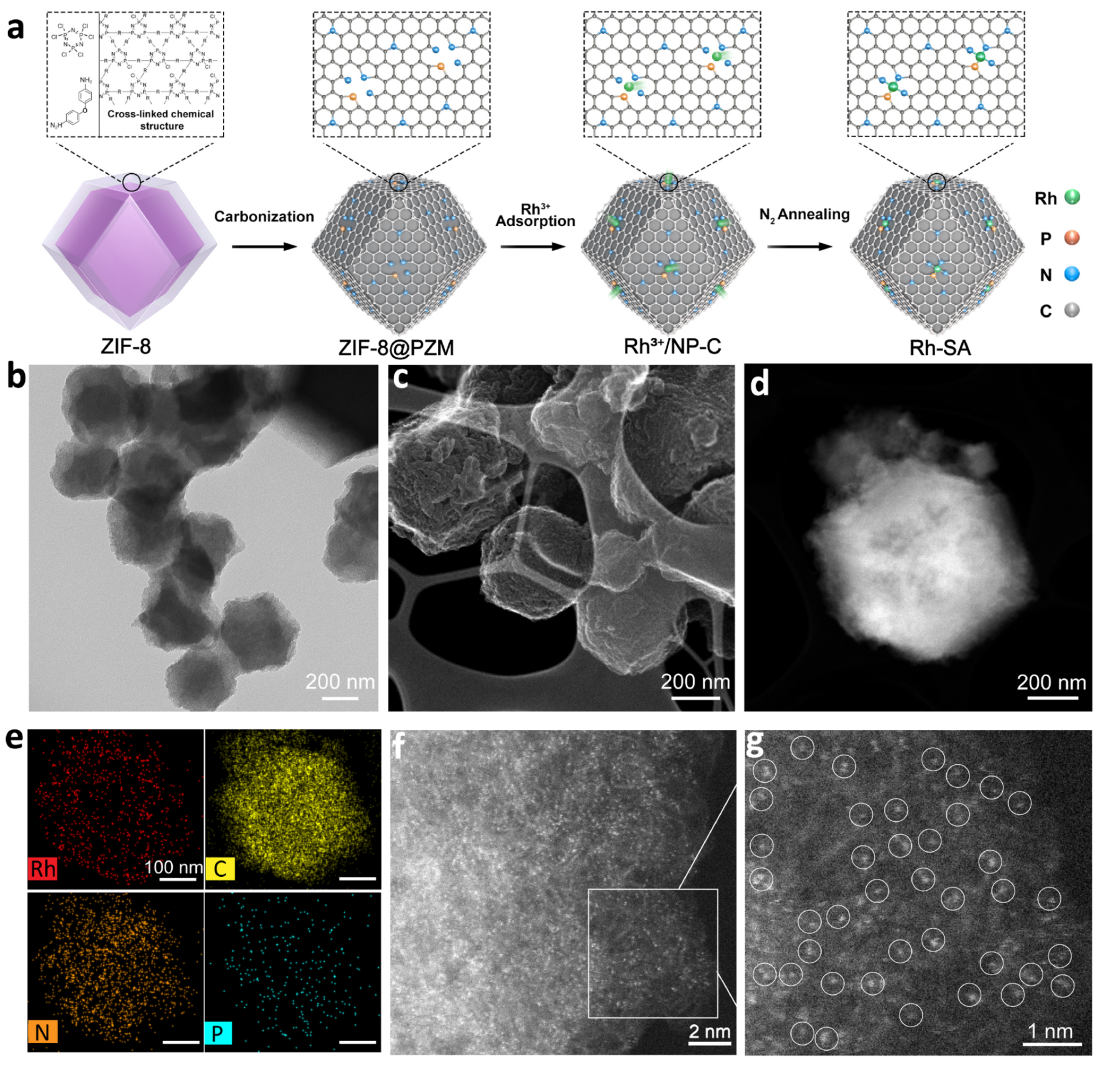
Synthesis and structural characterizations of Rh-SA. (a) Illustration of the synthetic strategy of Rh-SA. (b) TEM image of Rh-SA. (c) STEM secondary electron image of Rh-SA. (d) HAADF-STEM image of Rh-SA. (e) The corresponding EDS mappings of Rh-SA in (d) (Rh, red; C, yellow; N, orange; P, cyan). (f, g) AC HAADF-STEM image (f) and enlarged AC HAADF-STEM image (g) of Rh-SA marked by the rectangle in (f).

The prominence of Rh in homogeneous catalysts for transformation of diazo compounds prompted us to investigate the synthesis of the Rh-SA and its applications in heterogeneous catalysis. A typical synthetic route of the Rh-SA catalyst is presented in Figure 1a. The synthesis began with the preparation of zeolite imidazolate framework 8 (ZIF-8), which was then coated with poly(cyclotriphosphazene-*co*-4,4-diaminodiphenyl ether) to form the ZIF-8@PZM composite. Transmission electron microscopy (TEM) revealed that the morphology of the obtained ZIF-8@PZM composite resembled a polyhedron, with a slightly more rounded surface compared to that of the unmodified ZIF-8 (Figures S1a and S2a). In addition, X-ray diffraction (XRD) analysis demonstrated that the XRD pattern of ZIF-8@PZM closely matched that of ZIF-8 (Figures S1b and S2b), implying that the incorporation of the PZM shell had minimal impact on the crystallization of ZIF-8. Subsequently, the ZIF-8@PZM composite was carbonized at 950 °C under a N_2_ atmosphere, resulting in the formation of a nitrogen (N) and phosphorus (P) co-doped carbon support (NP-C) (Figure S3a). The broad peak observed in the XRD spectra of NP-C was attributed to the (002) plane of graphitic carbon (Figure S3b). The NP-C was then dispersed in a methanol solution of rhodium(III) chloride, enabling the capture of rhodium(III) ions within the nanostructures of the carbon support, leading to the formation of Rh^3+^/NP-C. Finally, annealing of Rh^3+^/NP-C at 250 °C under a N_2_ atmosphere yielded the desired catalyst, Rh-SA, in which Rh single atoms were successfully stabilized on the NP-C support.

Examination of the TEM and high-angle annular dark field scanning TEM (HAADF-STEM) images revealed that the Rh-SA catalysts exhibited a polyhedral shape with a wrinkled edge, which was similar to that of NP-C (Figure 1b,c and Figure S4). The enlarged HAADF-STEM image demonstrated the absence of observable aggregated bright spots corresponding to Rh nanoparticles in the engineered Rh-SA catalyst (Figure 1d). In addition, the energy-dispersive spectroscopy (EDS) mapping analysis indicated a homogeneous distribution of Rh, P, N, and C elements throughout the entire carbon nanostructure of the Rh-SA catalyst (Figure 1e). Consistent with the HAADF-STEM findings, the XRD pattern of the Rh-SA catalyst exhibited a broad peak of carbon nanostructures, whereas no signals of metallic Rh nanoparticles were detected (Figure S5). Additionally, the Rh loading of the Rh-SA catalyst was determined to be 0.78 wt % using inductively coupled plasma optical emission spectrometry (ICP-OES). From the aberration-corrected (AC) HAADF-STEM image of Rh-SA, the Rh single atoms were clearly identified as bright dots, which are marked by white circles (Figure 1f,g). These findings provided strong evidence that the structure of the Rh-SA catalyst consisted of Rh single atoms distributed on a N, P-doped carbon support.

To further investigate the chemical bonding states of C, N, and P in the Rh-SA catalyst, X-ray photoelectron spectroscopy (XPS) analysis was conducted. The C 1s spectrum revealed three distinct peaks corresponding to C=C (284.7 eV), C–N (287.5 eV), and C–P (285.7 eV) bonding states, respectively (Figure S6). Deconvolution of the N 1s spectrum yielded five peaks located at 398.6, 399.4, 400.2, 401.2, and 403.8 eV, corresponding to pyridinic N, N–Rh, pyrrolic N, graphitic N, and oxidized N, respectively (Figure S7). The P 2p spectrum exhibits two peaks at binding energies of 132.7 and 134.8 eV, associated with the P–C and P–Rh contributions, respectively (Figure S8). The XPS, XRD, and EDS analyses collectively confirmed the doping of N and P species within the carbon support nanostructures, as described earlier.

### Atomic Structural Analysis of Rh-SA by XAFS Spectroscopy

**Figure 2:**
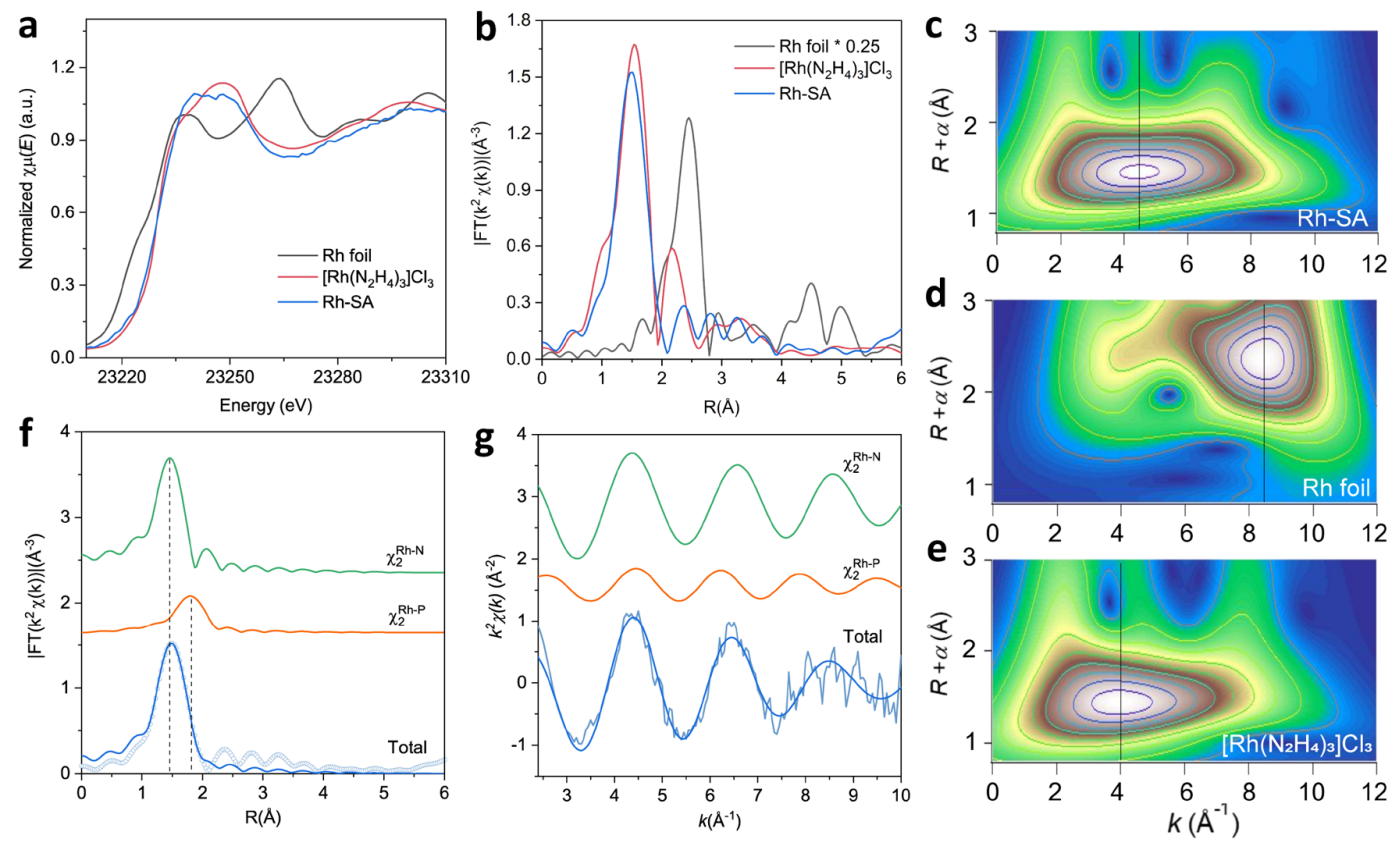
Atomic structural analysis of Rh-SA was performed by XAFS spectroscopy. (a) Rh K-edge XANES profiles of Rh-SA and its reference samples. (b) Rh K-edge EXAFS Fourier transforms of the Rh-SA and its reference samples. The Fourier transforms are not corrected for the phase shift. (c–e) Rh K-edge wavelet transform EXAFS contour plots of Rh-SA (c), Rh foil (d), and [Rh(N_2_H_4_)_3_]Cl_3_ (e). (f, g) Rh K-edge EXAFS fitting analysis of Rh-SA in R space (f) and *k* space (g).

To gain insight into the chemical environment and coordination structure of Rh species in Rh-SA, X-ray absorption fine structure spectroscopy (XAFS) was performed. The Rh K-edge X-ray absorption near edge structure (XANES) spectra of Rh-SA, Rh foil, and tris(ethylenediamine)rhodium(III) chloride ([Rh(N_2_H_4_)_3_]Cl_3_) were recorded and compared (Figure 2a). The absorption edge position in the XANES spectra of Rh-SA closely resembled that of [Rh(N_2_H_4_)_3_]Cl_3_ (Figure 2a), indicating that the Rh species possess a positive charge, slightly smaller than 3^+^. Further analysis of the Rh K-edge extended XAFS (EXAFS) Fourier transforms of Rh-SA revealed a prominent peak at about 1.5 Å. This feature is indicative of backscattering between Rh and light atoms (e.g., N and P), consistent with the Rh–N peak in [Rh(N_2_H_4_)_3_]Cl_3_.

A detailed comparison between Rh-SA and [Rh(N_2_H_4_)_3_]Cl_3_ revealed distinct features in the XAFS analysis. The Rh-SA spectrum exhibited a slightly broadened major peak with reduced intensity compared to that of [Rh(N_2_H_4_)_3_]Cl_3_ (Figure 2b). This observation can be attributed to the symmetry decrease induced by Rh–P coordination in the first coordination shell of Rh-SA. Moreover, when compared to the XAFS spectra of Rh foil, no noticeable Rh–Rh coordination peak at about 2.4 Å was observed in Rh-SA, consistent with the presence of isolated Rh single atoms. Subsequent analysis using EXAFS wavelet transform (WT) analysis (Figure 2c-2e) allowed for the identification of backscattering atoms and provided enhanced resolution in the R and *k* spaces. The WT plot of Rh-SA exhibited only a single intensity maximum at about 4.4 Å^–1^, consistent with the intensity maximum associated with Rh–N backscattering in [Rh(N_2_H_4_)_3_]Cl_3_. Notably, no Rh–Rh coordination peak was observed in Rh-SA, in contrast to the intensity maximum at about 8.5 Å^–1^ attributed to Rh–Rh coordination in the Rh foil. These findings are consistent with the atomic dispersion of Rh species in Rh-SA.

To elucidate the structural coordination environment of the species in Rh-SA, quantitative least-squares EXAFS curve-fitting analysis was performed. The analysis revealed the major peak at 1.5 Å in the best fit result for Rh-SA, which could be attributed to a combination of Rh–N and Rh–P coordination (Figure 2f and g). The coordination numbers of N and P atoms in the first coordination sphere of Rh–N and Rh–P were estimated to be 3.5 and 1.1, respectively, with the average bond lengths of 2.03 and 2.38 Å (Table S1). These results indicate a Rh_1_N_3_P structural composition at the Rh single-atom site. For comparison, the EXAFS curve-fitting analysis of the Rh foil and [Rh(N_2_H_4_)_3_]Cl_3_ were also conducted (Figures S9–S12 and Table S1). Based on this structural analysis, the RhN_3_P structural model for Rh-SA was established by density functional theory (DFT) calculations (Figure S13).

### Catalysis Study toward Chemoselective Carbene N–H Bond Insertions

**Scheme 1:**
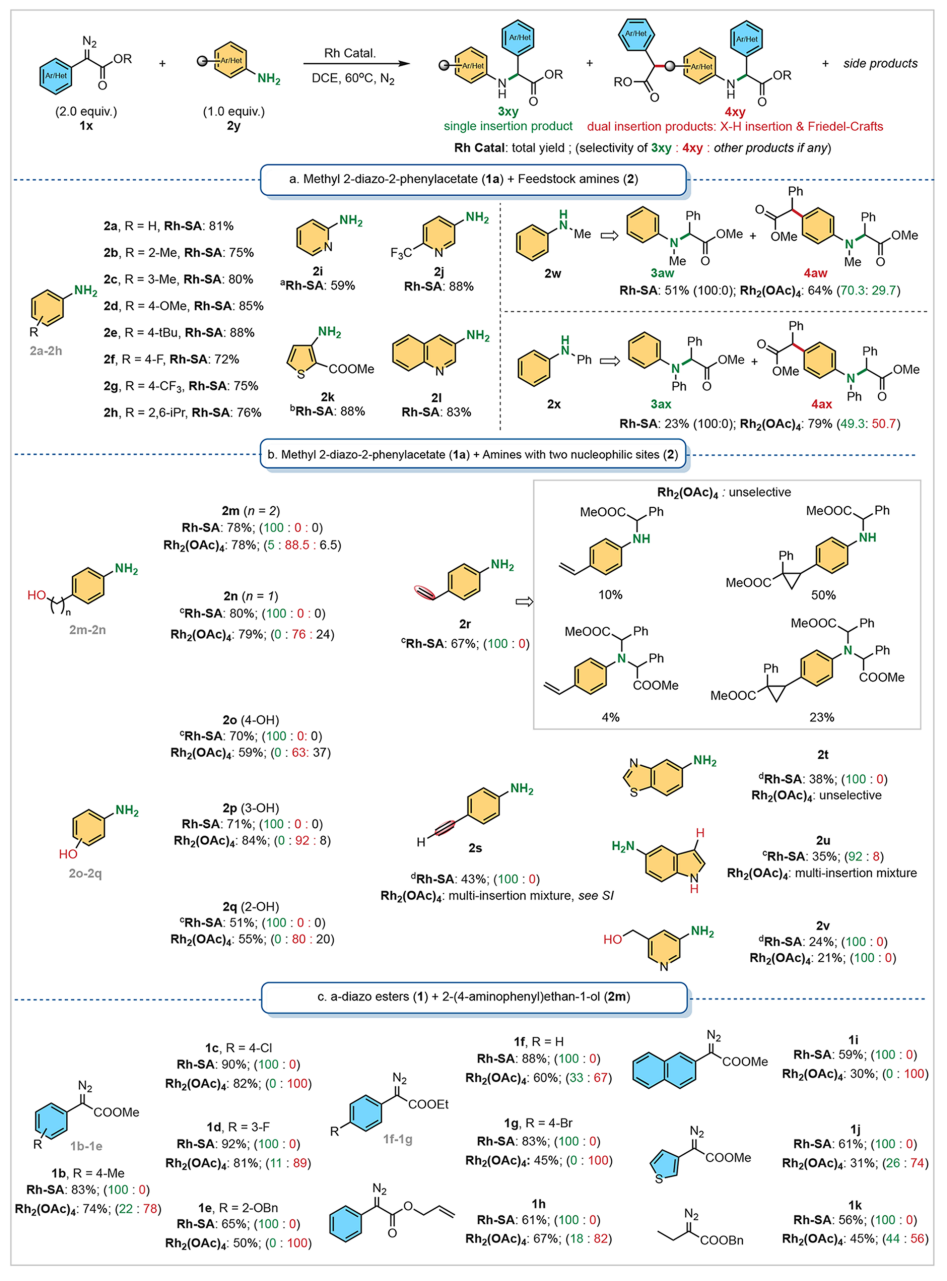
Rh-Catalyzed Carbene N–H Bond Insertions For heterogeneous catalysis, diazo ester **1** (0.2 mmol), amine **2** (0.1 mmol), 1,2-dichloroethane as solvent (0.5 mL, conc. 0.20 M in general, and 2 mL, conc. 0.05 M for **2m**-participating reactions) under nitrogen conditions, 60 °C, 18 h, 0.15 mol % Rh-SA was used. For homogeneous catalysis, diazo ester **1** (0.4 mmol), amine **2** (0.2 mmol), 1,2-dichloroethane as solvent (4 mL, conc. 0.05 M) under nitrogen conditions, 60 °C, 18 h, 3.6 mol % Rh_2_(OAc)_4_ was used (^a^80 °C, ^b^diazo ester 1 (0.1 mmol) was used, ^c^0.3 mol % Rh-SA was used, ^d^70 °C).

With the synthesized heterogeneous Rh-SA catalyst in hand, its activity and selectivity in catalytic carbene N–H insertion reactions were investigated next. At the outset, a variety of anilines and heteroaromatic amines were examined as the coupling partners using α-phenylmethyl diazoacetate **1a** as the carbene precursor (Scheme 1a). Employing a catalyst loading down to 0.15 mol % of Rh-SA catalyst, reaction of aniline derivatives **2a**–**2g** bearing electronically different substituents, such as alkyl, methoxy, fluoro, and trifluoromethyl groups, resulted in the formation of the corresponding N–H insertion products in good to excellent yields. Notably, even sterically hindered 2,5-diisopropyl aniline **2h** produced the desired adduct in 72% yield. Furthermore, heteroaromatic amines featuring pyridinyl (**2i**,**2j**), thiophenyl (**2k**), and quinolinyl (**2l**) groups were also successfully utilized in the heterogeneous catalysis. Interestingly, the Rh-SA catalyst provided reasonable yields of the desired products when using secondary anilines (**2w** and **2x**) as substrates, where homogeneous Rh_2_(OAc)_4_ gave unselective mixtures of the single N–H and dual N–H/C–H insertion products. Unfortunately, aliphatic amines, including benzylic amines, all exhibited significantly reduced activity with both heterogeneous Rh-SA and homogeneous Rh_2_(OAc)_4_ catalyst systems (see Figure S61 for details). These results highlight the potential of the Rh-SA catalyst in metal carbene-mediated N–H insertion reactions with excellent functional group tolerance.

The chemoselectivity of the heterogeneous Rh-SA catalyst was investigated by examining the reaction of diazoester **1a** with aniline derivatives bearing multiple potentially reactive motifs. Moreover, a comparison of the reaction outcomes was made between the heterogeneous Rh-SA and homogeneous Rh_2_(OAc)_4_ catalysts. Two other Rh(III)-based catalysts generally exhibited low reactivity toward carbene insertion reactions (see Figure S61 for details). As depicted in Scheme 1b and Figures S62–101, the engineered Rh-SA selectively promoted the single N–H insertion reaction of aniline substrates containing alcohols (**2m**,**2n**), whereas the classical Rh_2_(OAc)_4_ catalyst led to the preferential formation of dual insertion (N–H, O–H) and triple insertion (N–H, N–H, and O–H) products. Aminophenol derivatives (**2o**–**2q**) displayed similar trends in chemoselectivity, with the Rh-SA catalyst resulting in preferential N–H insertions. Notably, an unselective mixture of side products generated through either iterative X–H insertions or aromatic substitutions was formed under explicit homogeneous Rh catalysis in these three cases. Additionally, applications of anilines bearing alkenyl and alkynyl functional groups (**2r**,**2s**) predominantly generated the corresponding single N–H insertion products under the heterogeneous conditions. In contrast, employing Rh_2_(OAc)_4_ as a catalyst resulted in the formation of side products from uncontrolled multiple insertions as well as cyclization reactions. Moreover, high selectivities were obtained in the reaction of **2t**–**2v**, which feature heteroarene scaffolds such as benzothiazole, indole, and pyridine.

In addition to the scope of anilines, various (hetero) aryl diazo esters **1b**–**1j** were also tested in catalysis using 4-aminophenethyl alcohol **2q** as the coupling nucleophile (Scheme 1c). The heterogeneous Rh-SA catalysis exhibited similarly high chemoselectivity toward N–H insertions in high yields. In contrast, Rh_2_(OAc)_4_ preferentially led to dual-insertion products or a mixture of single- and dual-insertion products. Interestingly, when allyl 2-diazo-2-phenylacetate **1h** was used in the heterogeneous catalysis, no intramolecular cyclopropanation was observed, and the single N–H insertion product was formed in 61% yield. Furthermore, alkyl diazo ester **1k** furnished exclusively the N–H insertion product using the Rh-SA catalyst. These results demonstrate the viability of the Rh-SA catalyst for metal carbene N–H insertion reactions.

### Mechanistic Studies

**Figure 3:**
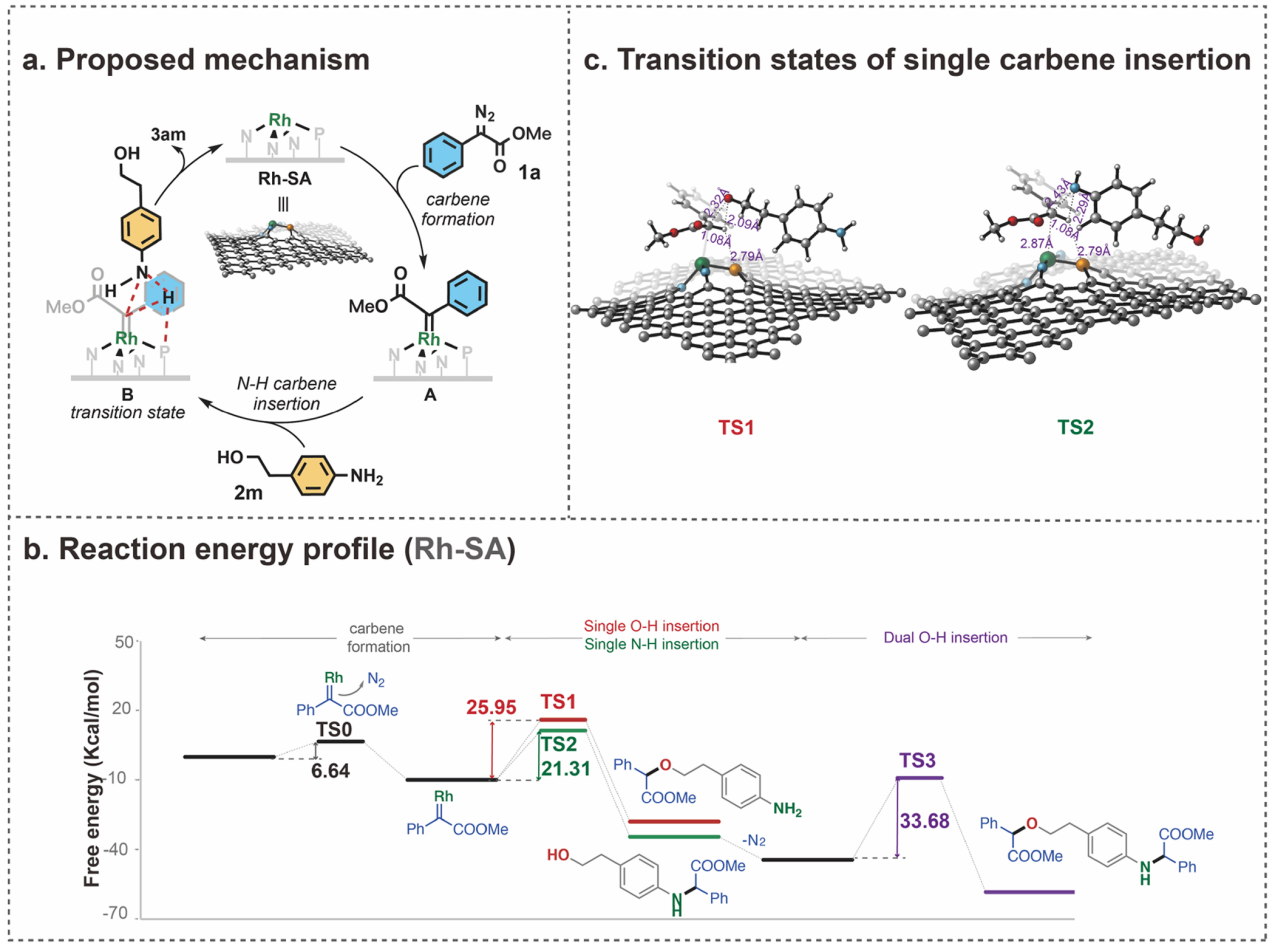
Mechanistic studies were carried out by DFT calculations. (a) Proposed mechanism for heterogeneous Rh-SA-catalyzed carbene N–H insertion. (b) Free energy diagram for the selective N–H bond insertion in heterogeneous Rh-SA catalysis. (c) The transition state structures of single O–H insertion **TS1** and single N–H insertion **TS2**. All the energies were corrected to free energy at 333 K. Details of optimized structures are in the Supporting Information.

To gain a deeper understanding of the catalytic behavior exhibited and the chemoselective N–H bond insertion reaction, subsequent investigations using DFT calculations were conducted to analyze the energy barriers in heterogeneous Rh-SA catalysis (see Figure 3). Accordingly, α-phenylmethyl diazoacetate (**1a**) and 4-aminophenethyl alcohol (**2m**) were chosen as the model substrates. The putative catalytic cycle (Figure 3a) encompassed the elementary steps involving the formation of a metal carbene (C=Rh) through the decomposition of a diazo ester, followed by subsequent insertion of the X–H bond (X = N or O) into the metal carbene intermediate. As expected, the rhodium carbene formation was exothermic and exhibited a small activation barrier of 6.64 kcal/mol (**TS0**) (Figure 3b). Understanding the relative energy barriers in the subsequent formation of the O–H and N–H insertion products, as well as the mono- and dual-insertion products, is crucial to correlate the origin of selectivity in catalysis. The calculations show that the carbene N–H insertion step features a reaction barrier of 21.31 kcal/mol (**TS2**), which is smaller than that of the corresponding single carbene O–H insertion (**TS1**, 25.95 kcal/mol), resulting in the selective formation of **3am**.^71^ It is noteworthy that the Bader charge (+0.82) at the Rh atom of RhN_3_P carbene species was observed to be smaller in comparison to the cases of the analogue RhN_4_ (+1.07) and the homogeneous Rh_2_(OAc)_4_ (+1.12), respectively. As a result, the metal carbene of the engineered RhN_3_P features lower electrophilicity, thus enhancing the selectivity of subsequent insertion toward the N–H bond. Finally, conversion of **3am** into the corresponding dual-insertion product **4am** is prohibited due to the presence of the larger reaction barrier of 33.68 kcal/mol in this case.

Interestingly, as shown in Figure 3c, the distance between the P atom and the transferred H atoms in both **TS1** and **TS2** is 2.79 Å, which is within the van der Waals force. In addition, the charge density difference indicates the electron distributions of P and the transferred H atoms are opposite, thus inducing the presence of electrostatic attraction between P and H atoms for further stabilization of these two transition states.^72^ This interaction accelerated the proton transfer in carbene insertion and suggested the X–H insertion step in the heterogeneous catalytic system is proceeding via a concerted addition. Consistent with this hypothesis, the analogous Rh–N_4_ catalyst, lacking the P atom, afforded **3am** in high selectivity but with significantly diminished 18% yield. Moreover, DFT calculations supported that participation of the P atom plays a critical role in the observed activity of the Rh**-**SA catalyst (see details in the SI). This phenomenon is different from the pathway of traditional homogeneous Rh_2_(OAc)_4_, in which proton shift is believed to occur after the addition of X to the carbene carbon.^73^

To further understand the proposed reaction pathway, kinetic and Hammett-plot analyses were carried out (for more details, see the SI). The kinetic analysis of the reaction catalyzed by Rh-SA revealed that the reaction rate is dependent on the concentration of two starting materials (**1a** and **2a**) as well as the amount of Rh-SA catalyst. Additionally, Hammett-type analysis illustrated that the electronic nature of the substituted anilines and phenyl diazo esters can both influence the relative reaction rate. In detail, for the Hammett plot of relative reaction rates of para-substituted anilines, a slightly better correlation was found with σ+ (*R*^2^ = 0.99) than for σ (*R*^2^ = 0.97). In both cases, the Hammett plot yields a positive ρ value (e.g., ρ = +1.08 for σ+). This positive ρ contrasts sharply with related studies of iron porphyrin-catalyzed aniline N–H insertion reactions (ρ = −0.66).^74^ Moreover, iridium-porphyrin reactions, while showing a negative ρ value, were significantly less sensitive to the electronic effects on the aniline (ρ = 0.15).^75^ A relative rate analysis of para-substituted phenyl diazo esters produced a negative ρ value of −1.06 in the Hammett plot using σ+. The results of the Hammett analysis are consistent with the results of the DFT calculations. For example, the Bader charge on the nitrogen of the aniline (−1.22) becomes less negative (−0.98) in the rate-determining transition (see Table S3). This phenomenon aligns with the findings of the DFT studies, which find a concerted, highly asynchronous transition state in the carbene insertion step involving proton transfer from the aniline to the carbene. This contrasts with the majority of homogeneous-catalyzed processes and may, in part, be the source of the observed differences in chemoselectivity.

## Conclusion

By leveraging techniques from material science, a heterogeneous Rh single-atom catalyst (Rh-SA) was successfully prepared. Structural confirmation using a series of characterizations, including EXAFS and XANES, supports the conclusion that the Rh atom center is coordinated to three N atoms and one P atom. The Rh-SA catalyst was applied to carbene N–H insertions, enabling efficient transformations of a variety of functionalized aniline derivatives in high yields with excellent site selectivity. Based on the DFT studies, the origin of both reactivity and selectivity is rationalized by the differentiation of energy barriers of carbene insertions and the accelerated proton transfer resulting in coordinating phosphorus atoms in the material support. These investigations further highlight the potential and promise of heterogeneous single-atom catalysis in organic synthesis.

## Supporting Information Available

Experimental details, including general information for chemicals and characterization, supplementary characterization of the Rh-SA catalyst, methods for catalysis, data for products, supplementary NMR spectra of products (PDF)
Supplementary DFT data (PDF)
