# Supplementary material for: Heterogeneous Rhodium Single-Atom-Site Catalyst Enables Chemoselective Carbene N–H Bond Insertion
Source: J Am Chem Soc. 2024 Apr 7;146(15):10847–56. doi: 10.1021/jacs.4c01408 (PMC11027138; doi:10.1021/jacs.4c01408)
Supplement: Supplementary file 1 — ja4c01408_si_001.pdf [file ja4c01408_si_001.pdf]

Supplementary Information for

Heterogeneous Rhodium Single-Atom-Site Catalyst Enables Chemoselective Carbene N-H Bond Insertion

Yuanjun Chen[1,2‡], Ruixue Zhang[1,‡], Zhiwen Chen[3,‡], Jiangwen Liao[4], Xuedong Song[1], Xiao Liang[2], Yu Wang[5], Juncai Dong[4], Chandra Veer Singh[3], Dingsheng Wang[2,*], Yadong Li[2,*], F. Dean Toste[6,7*], Jie Zhao[1,*]

‡These authors contributed equally to this work
Correspondence to: zhaojie@ecust.edu.cn
fdtoste@berkeley.edu
ydli@mail.tsinghua.edu.cn
wangdingsheng@mail.tsinghua.edu.cn

This PDF file includes:

General information for chemicals and characterization
Supplementary characterization of Rh-SA catalyst
Supplementary DFT calculation
Supplementary mechanistic experiment
Supplementary methods for catalysis
Supplementary data for reactions with crude NMR analysis
Supplementary data for products
Supplementary references

General information for chemicals and characterization

Chemicals

Chemicals employed in catalyst preparation: rhodium (III) chloride hydrate, zinc nitrate hexahydrate, 2-methylimidazole, phosphonitrilic chloride trimer, bis(4-aminophenyl) ether and tris(ethylenediamine)rhodium(III) chloride were obtained from Alfa Aesar. Triethylamine and methanol were obtained from Sinoparm Chemical reagent Beijing Co., Ltd.. The homogeneous catalyst rhodium (II) acetate dimer was purchased from Strem chemical.

Chemicals employed in catalysis study were of analytical grade and used as received from the suppliers without extra purification. Solvents were all degassed through "Freeze-Pump-Thaw" procedure before using in catalytic experiments. Flash chromatography was performed with Fluka silica gel 60 (0.040 - 0.063 μm grade). Compounds were visualized by UV-light at 254 nm and by dipping the plates in an ethanolic vanillin or an aqueous $KMnO_4$ solution followed by heating.

Caution: diazo esters employed as carbene precursors should be prepared and stored in limited scale.

Characterization

X-ray diffraction (XRD) data were obtained on a Rigaku MiniFlex 600 diffractometer (Bragg–Brentano geometry) equipped with a monochromatized Cu Kα radiation source (λ = 1.5406 Å, 40 kV, and 15 mA). X-ray photoelectron spectra (XPS) were collected a with a Thermo Fisher ESCALAB 250Xi using Al Kα X-rays as the excitation source. Transmission electron microscope (TEM) images were recorded with Hitachi HT7700 instrument. Scanning transmission electron microscopy (STEM) images were recorded by using a Hitachi HF3300 equipped with a cold field emission electron gun, and a Brucker silicon-drift EDS detector operated at 300 kV. High-angle annular dark-field scanning transmission electron microscopy (HAADF-STEM) images and elemental maps for the determination of element composition and distribution were recorded with JEOL 200F high resolution TEM operated at 200 keV, equipped with a probe spherical aberration corrector. Metal contents in samples were determined by inductively coupled plasma optical emission spectrometer (ICP-OES) on Optima 7300 DV.

X-ray absorption fine structure (XAFS) measurements of samples were carried out at the BL14W1 station in Shanghai Synchrotron Radiation Facility (SSRF, 3.5GeV, 250mA maximum, Si(311) double crystals). The Rh K-edge data of the Rh single atom samples were collected in fluorescence excitation mode and XAFS spectra of the references were recorded in transmission mode using ionization chamber at room temperature. All XAFS raw data were background-subtracted, normalized and Fourier-transformed by standard procedures with the ATHENA module implemented in the IIRFFIT software packages. Subsequently, the EXAFS χ(k) data were Fourier transformed to real space using a Hanning windows (dk=1.0 Å-1) to separate the EXAFS contributions from different coordination shells. To obtain the quantitative structural parameters

around central atoms, least-squares curve parameter fitting was performed using the ARTEMIS module of IIRFFIT software packages.

Proton nuclear magnetic resonance (1H-NMR) data were acquired on a Bruker AV400 (400 MHz), Bruker Avance500 (500 MHz). Chemical shifts are reported in delta (δ) units, in parts per million (ppm) downfield from tetramethylsilane. Splitting patterns are designated as s, singlet; d, doublet; t, triplet; q, quartet; sept, septet; m, multiplet, br, broad. Proton decoupled Carbon-13 nuclear magnetic resonance (13C-NMR) data were acquired at 100 MHz on a Bruker AV400 spectrometer, or at 125 MHz on a Bruker Avance500. Chemical shifts are reported in ppm relative to the center line of a triplet at 77.0 ppm for chloroform-d. High resolution mass spectra were recorded by an Agilent LC-MS TOF and are given in m/z.

Catalyst preparation

First, zeolitic imidazolate frameworks (ZIF-8) as raw materials were prepared via a facile solvothermal method. Initially, 75 mL methanol solution of zinc nitrate hexahydrate (0.128 M) and 75 mL methanol solution of 2-methylimidazole (0.512 M) were mixed and stirred vigorously at room temperature for 5 min. Then, the resulting solution was transferred and sealed in a container, followed by heating to 35 °C for 6 h to improve the crystallinity of ZIF-8. The as-obtained ZIF-8 was washed thoroughly with methanol for several times and dried in vacuum at 80 °C. Subsequently, 800 mg ZIF-8 sample was dispersed in 40 mL methanol, and added into the 200 mL methanol solution with 640 mg of bis(4-aminophenyl) ether and 320 mg of phosphonitrilic chloride trimer under continuous stirring. Then, 16 mL triethylamine and 100 mL methanol were mixed and slowly dropped into the above suspension, following by heating to 40 °C for 14 h to prepare the ZIF-8 coating poly-(cyclotriphospazene-co-4,4'-diaminodiphenylether) (PZM) sample, which was marked as ZIF-8@PZM. The as-obtained ZIF-8@PZM was annealed in N_2 atmosphere at 950 °C for 3 h (heating rate: 5° min^{-1}). The N_2-treated sample was denoted as NP-C. For the synthesis of Rh-SA sample, 200 mg NP-C was dispersed in 20 mL methanol, and 10 mL methanol solution of rhodium(III) chloride hydrate (1 mg/mL) was slowly dropped into the above suspension of NP-C under continuous stirring for more than 24 h, followed by washing by methanol, drying and annealing in N_2 atmosphere at 250 °C for 2 h (heating rate: 2° min^{-1}). Finally, the Rh-SA catalyst was prepared and used without further purification. The Rh-N_4 control sample was prepared via the same synthesis procedure as the Rh-SA catalyst, except without PZM coating.

Supplementary characterization of Rh-SA catalyst

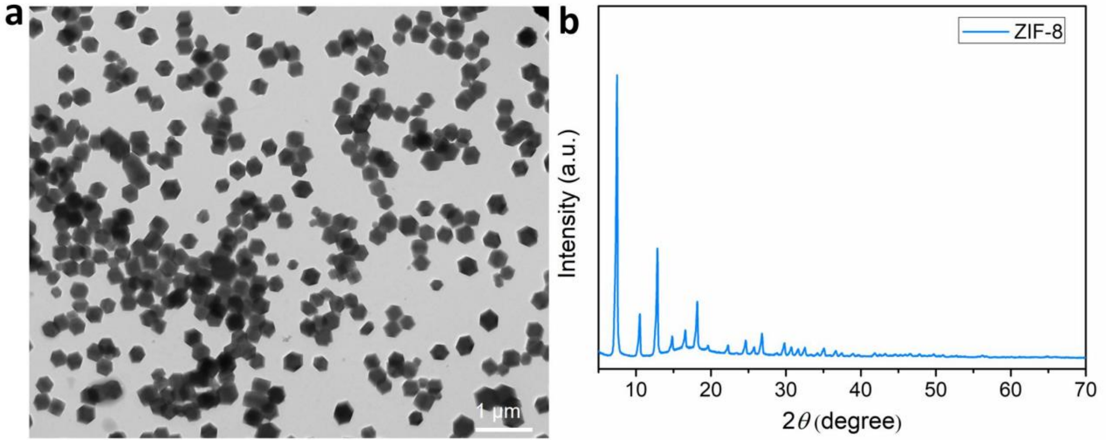

Figure S1. (a) TEM image of ZIF-8. (b) XRD pattern of ZIF-8.

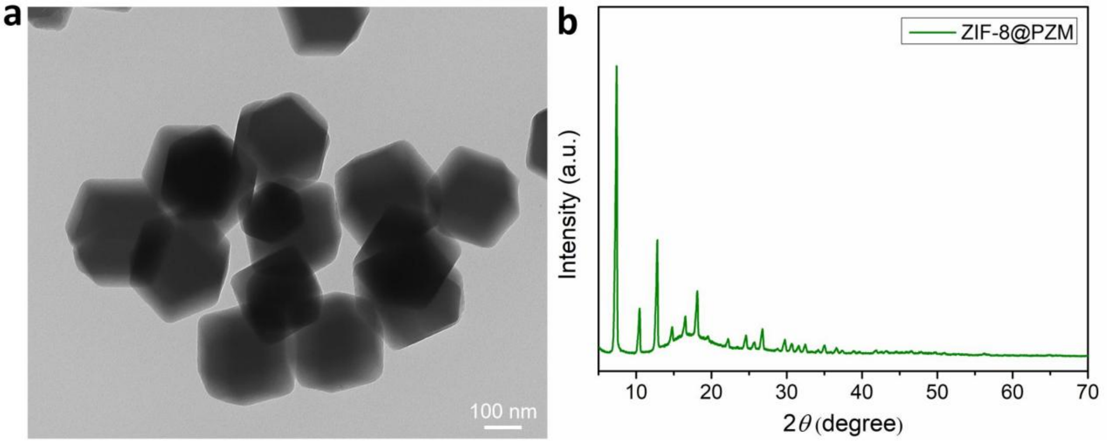

Figure S2. (a) TEM image of ZIF-8@PZM. (b) XRD pattern of ZIF-8@PZM.

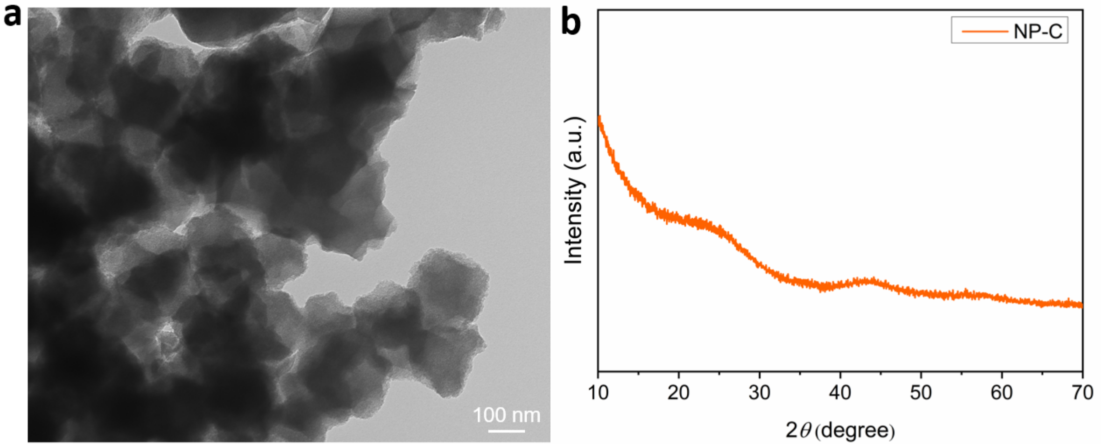

Figure S3. (a) TEM image NP-C. (b) XRD pattern of NP-C.

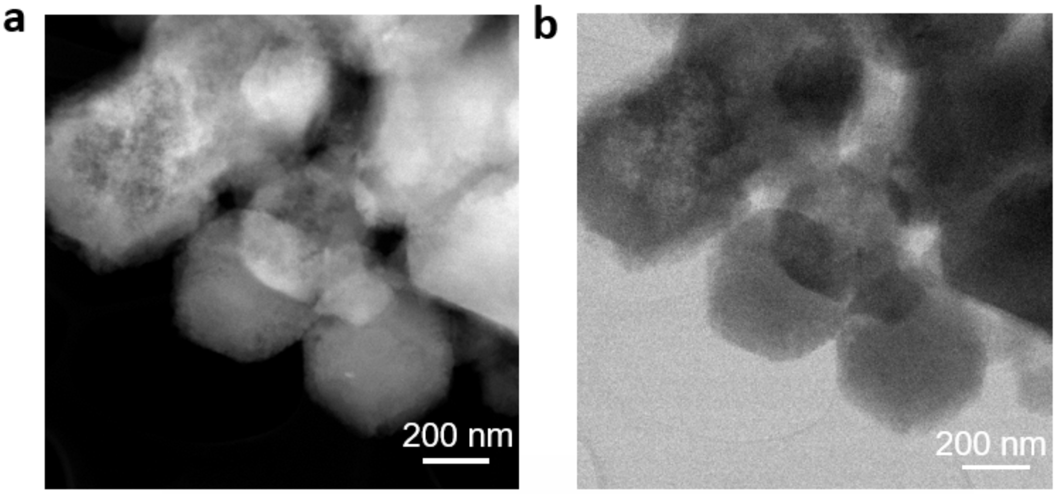

Figure S4. (a) HAADF-STEM image and (b) STEM-bright field image of the Rh-SA catalyst.

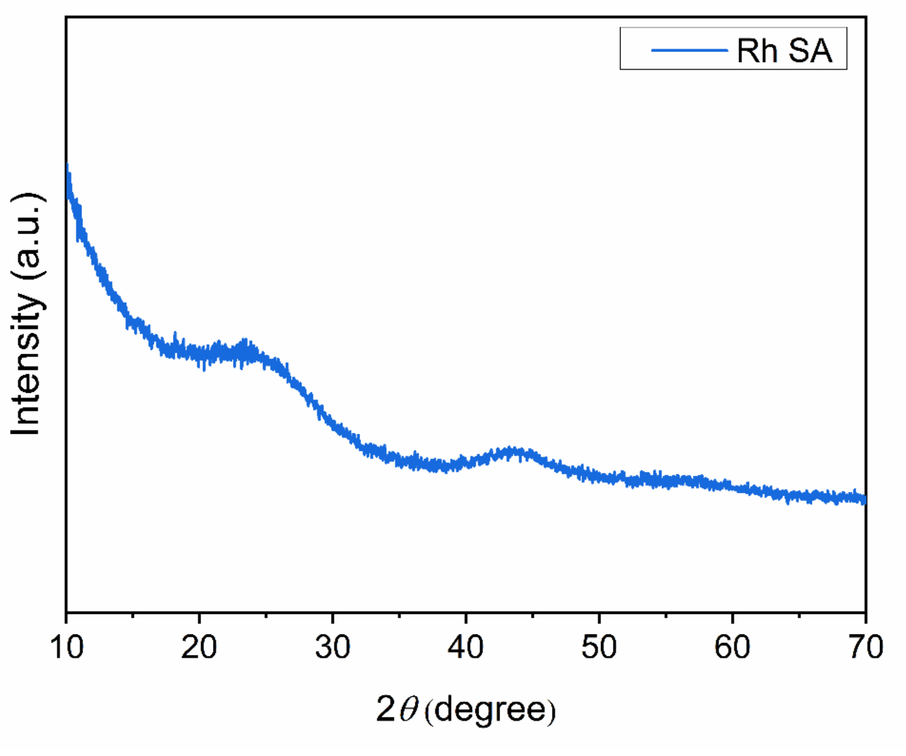

Figure S5. XRD patterns of the Rh-SA catalyst.

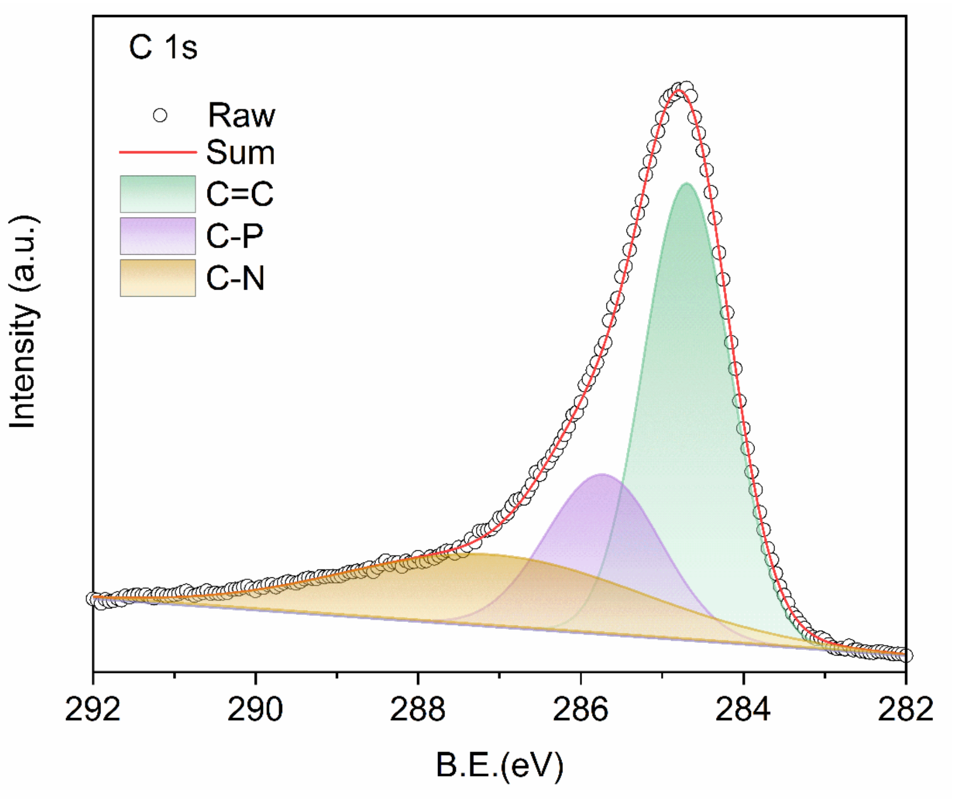

Figure S6. The C 1s spectrum of the Rh-SA catalyst.

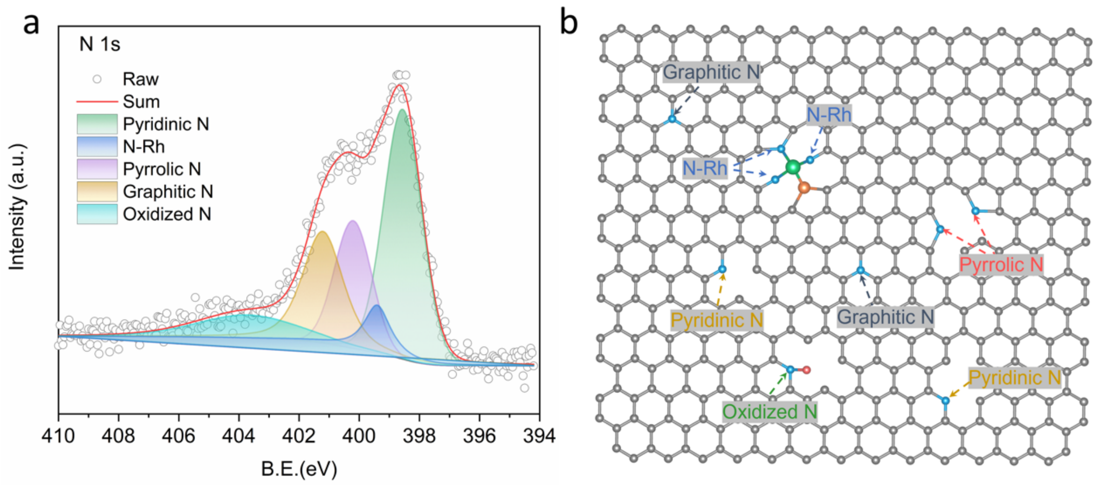

Figure S7. a. The N 1s spectrum of the Rh-SA catalyst. b. Schematic illustration of the structures of pyridinic N, N-Rh, pyrrolic N, graphitic N, and oxidized N (Rh, light green; N, light blue; P, orange; C, gray; O, red).

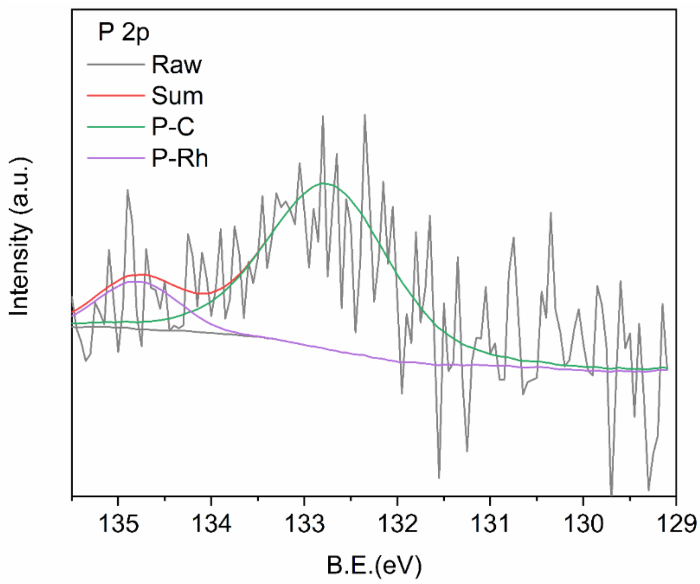

Figure S8. The P 2p spectrum of the Rh-SA catalyst.

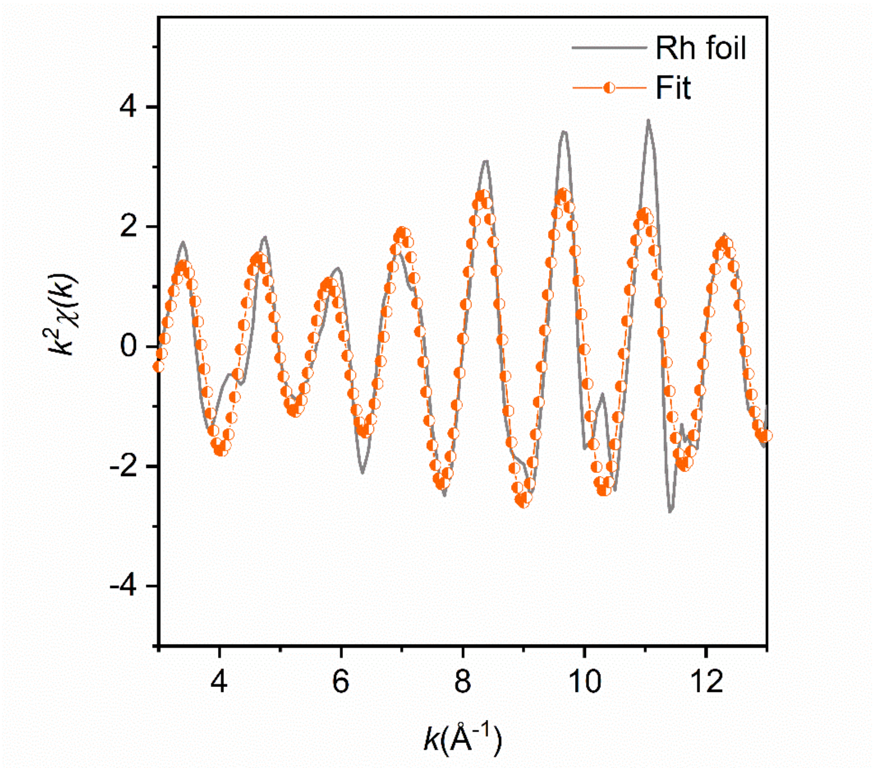

Figure S9. Rh K-edge EXAFS fitting analysis of Rh-SA in *k* space.

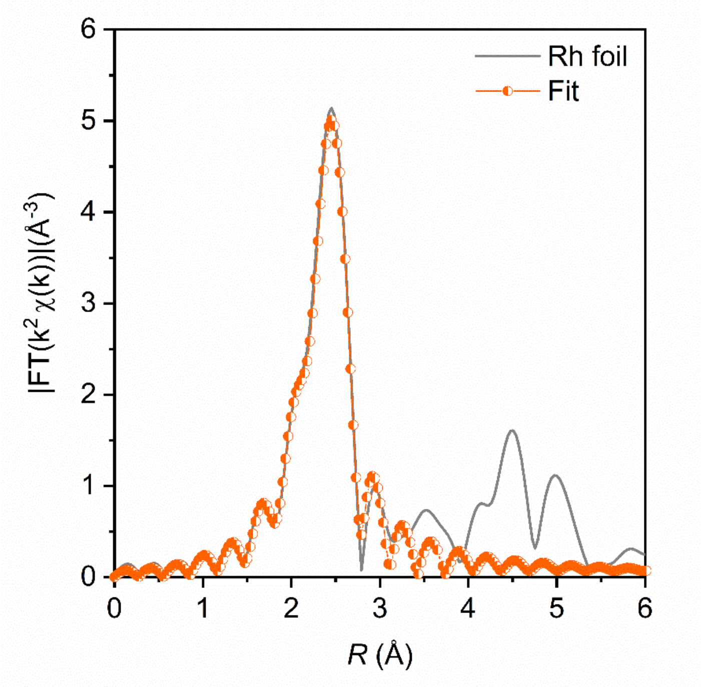

Figure S10. Rh K-edge EXAFS fitting analysis of Rh foil in R space.

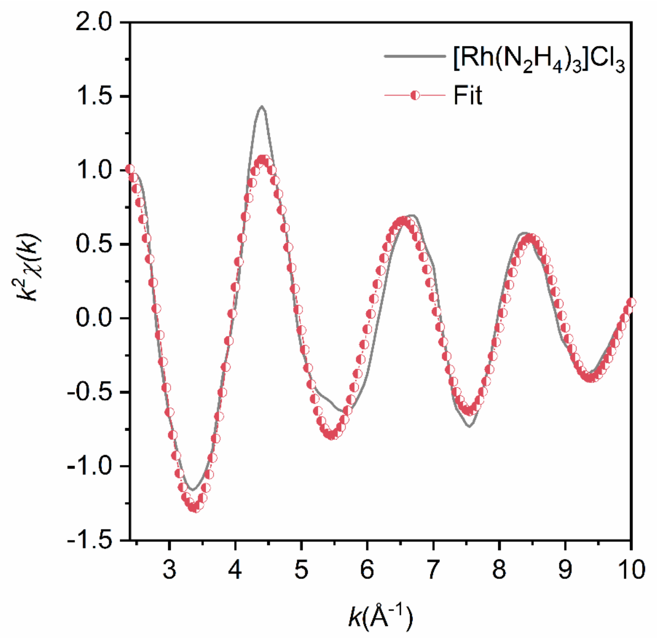

Figure S11. Rh K-edge EXAFS fitting analysis of $[Rh(N_2H_4)_3]Cl_3$ in *k* space.

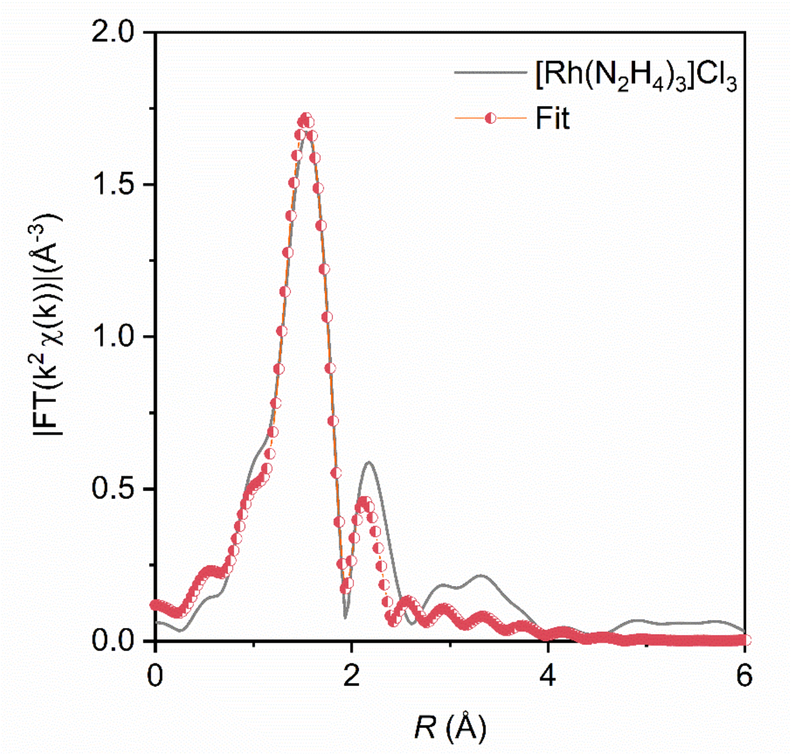

Figure S12. Rh K-edge EXAFS fitting analysis of $[Rh(N_2H_4)_3]Cl_3$ in R space.

Table S1. Rh K-edge EXAFS curve Fitting Parameters of Rh-SA and its reference samples.[a]

Sample	path	N	R (Å)	σ^2 (Å^2)	ΔE_0 (eV)	R, %
Rh foil[b]	Rh-Rh	12	2.68	0.005	4.6	0.1
$[Rh(N_2H_4)_3]Cl_3$[c]	Rh-N	6	2.07	0.004	2.5	0.3
Rh-SA[c]	Rh-N	3.5	2.03	0.003	0.2	0.3
	Rh-P	1.1	2.38	0.006	-4.8	

[a]*N*,coordination number; *R*, distance between absorber and backscatter atoms ;σ^2, Debye–Waller factor to account for both thermal and structural disorders; ΔE_0, inner potential correction; *R* factor (%) indicates the goodness of the fit. Error bounds (accuracies) that characterize the structural parameters obtained by EXAFS spectroscopy were estimated as $N \pm 20\%$; $R \pm 1\%$; $\sigma^2 \pm 20\%$; $\Delta E_0 \pm 20\%$. S_0^2 was fixed to 1.0 as determined from Rh foil fitting. Bold numbers indicate fixed coordination number (*N*) according to the crystal structure. It should be noted that the N and O coordinations around the Rh absorber in sample are indistinguishable by EXAS due to their nearly identical scattering ability of photoelectron. [b]Fitting range: $3.0 \leq k$ (/Å) ≤ 13.0 and $1 \leq R$ (Å) ≤ 2.8. [c]Fitting range: $2.4 \leq k$ (/Å) ≤ 10.0 and $1.0 \leq R$ (Å) ≤ 2.0.

Supplementary DFT calculation

(1) Methods

All calculations were conducted with spin-polarized DFT calculations using the Vienna Ab initio Simulation Package (VASP).[9] The interaction between valence electrons and ionic cores was described by the Projector-augmented wave pseudopotential and the exchange -correlation effects were considered by the Perdew-Burke-Ernzerhof functional of the generalized gradient approximation.[10,11] A 8 × 8 supercell with 15 Å vacuum space were used to avoid interactions between the system and its mirror images. The kinetic energy cutoff for the wave-function calculations was 550 eV. A smearing width of 0.05 eV was used for the Fermi smearing function. The geometric relaxation was performed with the convergence energy and force of 1×10^{-5} eV and 0.05 eV/Å, respectively. All the transition states were calculated through the climbing image nudged elastic band (CI-NEB) method with the convergence force smaller than 0.1 eV/Å.[12] Free energy corrections were conducted in this work to simulate the reaction conditions at 333 K. Standard free energy corrections were calculated by the equation of $\Delta G = \Delta E_{\mathrm{DFT}} + \Delta E_{\mathrm{ZPE}} + \Delta E_{\mathrm{U}} - T\Delta S$. ΔE_{DFT}, ΔE_{ZPE}, ΔE_{U}, and ΔS indicate the electronic energy from DFT calculations, the zero-point energy correction, inner energy correction, and entropy change, respectively.

*(2) DFT calculation with **Rh-SA***

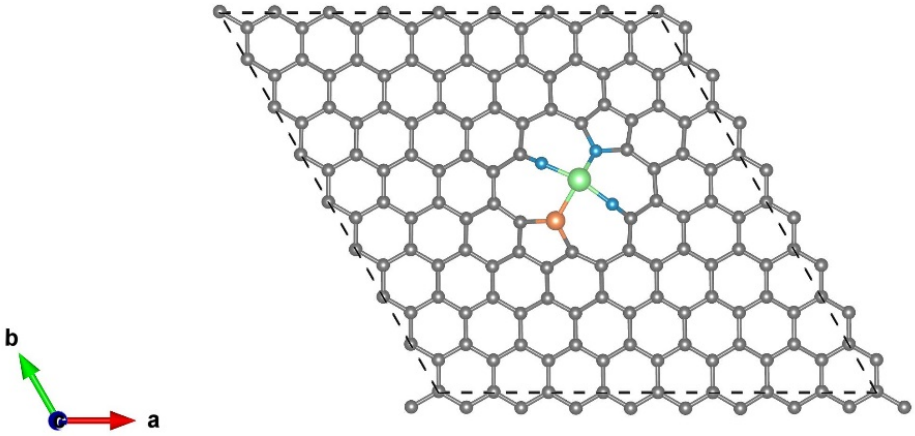

Figure S13. Schematic model of Rh-SA was established by DFT calculations (Rh, light green; N, light blue; P, orange; C, gray).

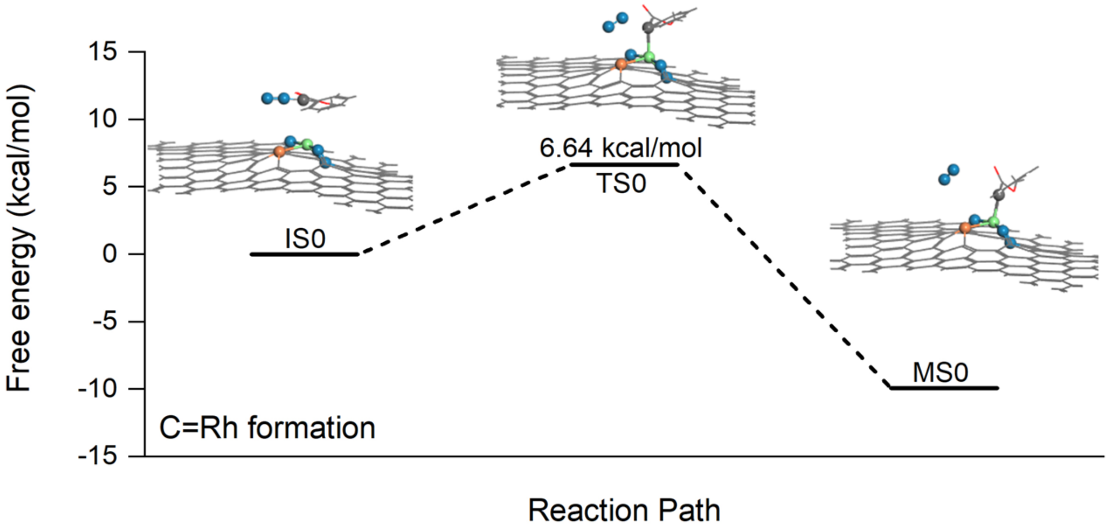

Figure S14. Reaction process of C=Rh formation on heterogeneous Rh-SA catalyst with the corresponding free energy and structures. White, red, gray, blue, orange, and green balls indicate H, O, C, N, P, and Rh atoms.

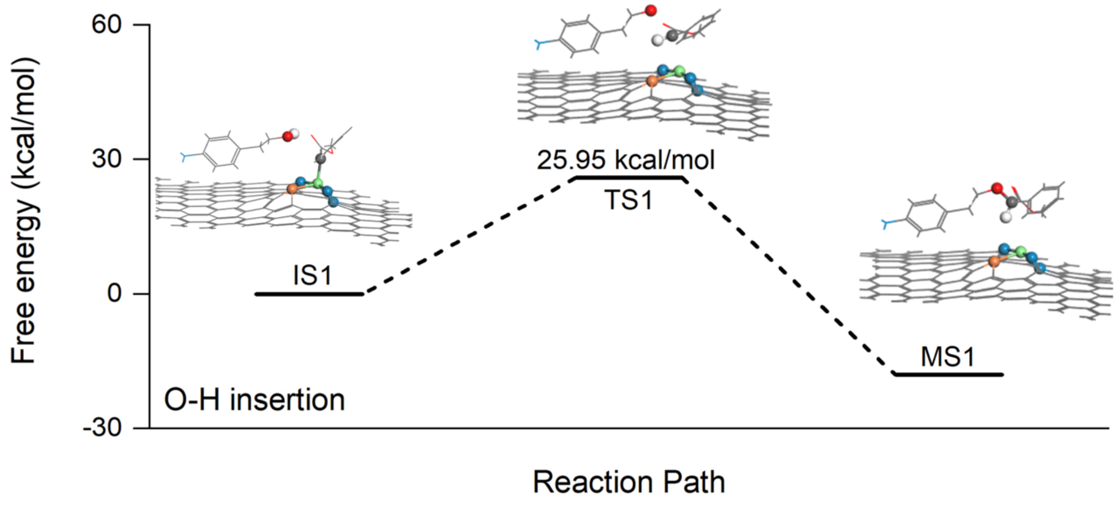

Figure S15. Reaction process of O-H insertion on heterogeneous Rh-SA catalyst with the corresponding free energy and structures. White, red, gray, blue, orange, and green balls indicate H, O, C, N, P, and Rh atoms.

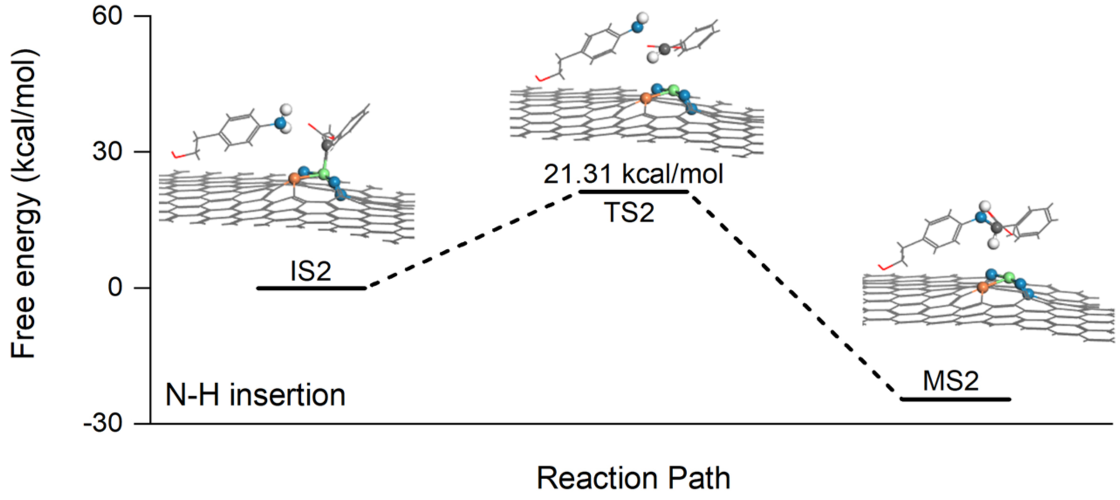

Figure S16. Reaction process of N-H insertion on heterogeneous Rh-SA catalyst with the corresponding free energy and structures. White, red, gray, blue, orange, and green balls indicate H, O, C, N, P, and Rh atoms.

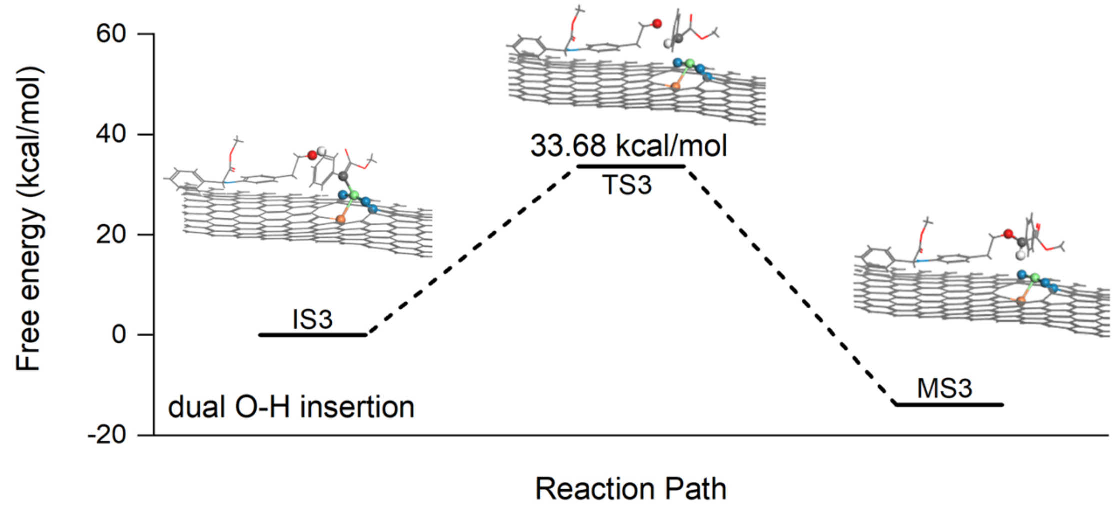

Figure S17. Reaction process of dual O-H insertion on heterogeneous Rh-SA catalyst with the corresponding free

energy and structures. White, red, gray, blue, orange, and green balls indicate H, O, C, N, P, and Rh atoms.

To validate TS1 and TS2, we conducted frequency calculations, revealing one imaginary frequencies of 335.38 cm^{-1} and 317.96 cm^{-1} for TS1 and TS2, respectively. The oscillation modes were subsequently analyzed using Jmol, as depicted in **Figure S18**. The direction of the respective oscillation aligns with the intended reaction process, affirming the validity of both TS1 and TS2.

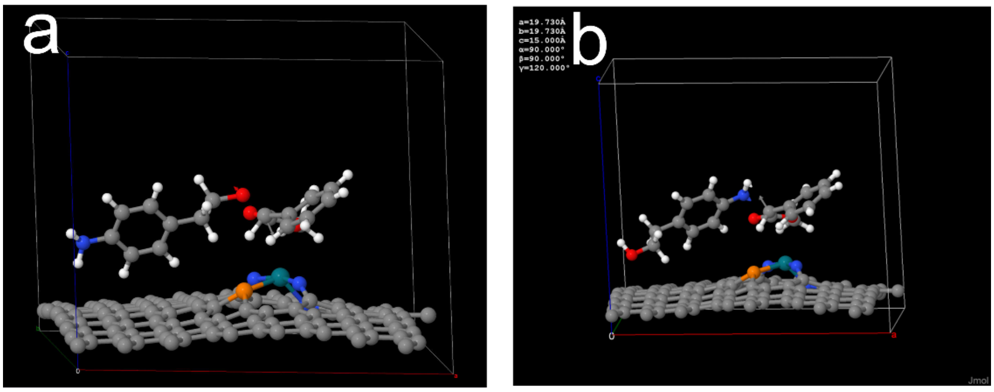

Figure S18. The structures of TS1 (a) and TS2 (b) for Rh-SA, with the oscillating modes depicted at their imaginary frequencies of 335.38 cm^{-1} and 317.96 cm^{-1}, respectively.

Table S2. Key distances between C-H, C-N, O-C, P-H, N-H, and O-H during the reaction process on Rh SA catalyst.

	C-H distance (Å)	C-N distance (Å)	O-C distance (Å)	P-H distance (Å)	N-H distance (Å)	O-H distance (Å)
IS1	2.74		3.11	4.19		0.98
TS1	1.08		2.32	2.80		2.10
MS1	1.11		1.42	3.12		2.09
IS2	3.03	3.62		3.51	1.02	
TS2	1.08	2.45		2.80	2.27	
MS2	1.11	1.44		3.08	2.08	

Table S3. Bader charge analysis of key elements (Rh, C, H, P, N, and O) during the reaction processes using Rh-SA.

	Rh	C	H	P	N	O
IS0	+0.77	+0.36		+1.24		
TS0	+0.80	-0.01		+1.23		
MS0	+0.82	-0.12		+1.16		
IS1	+0.83	-0.12	+0.65	+1.17		-1.17
TS1	+0.80	-0.12	+0.15	+1.28		-0.83
MS1	+0.78	+0.48	+0.06	+1.28		-1.03
IS2	+0.82	-0.10	+0.47	+1.13	-1.22	
TS2	+0.77	-0.05	+0.13	+1.28	-0.98	
MS2	+0.73	+0.37	+0.07	+1.29	-1.19	
IS3	+0.92	-0.12	+0.63	+1.08		-1.15
TS3	+0.89	-0.13	+0.20	+1.04		-0.84
MS3	+0.86	+0.50	+0.02	+1.00		-1.01

(3) DFT calculation with ***Rh₂(OAc)₄*** catalyst

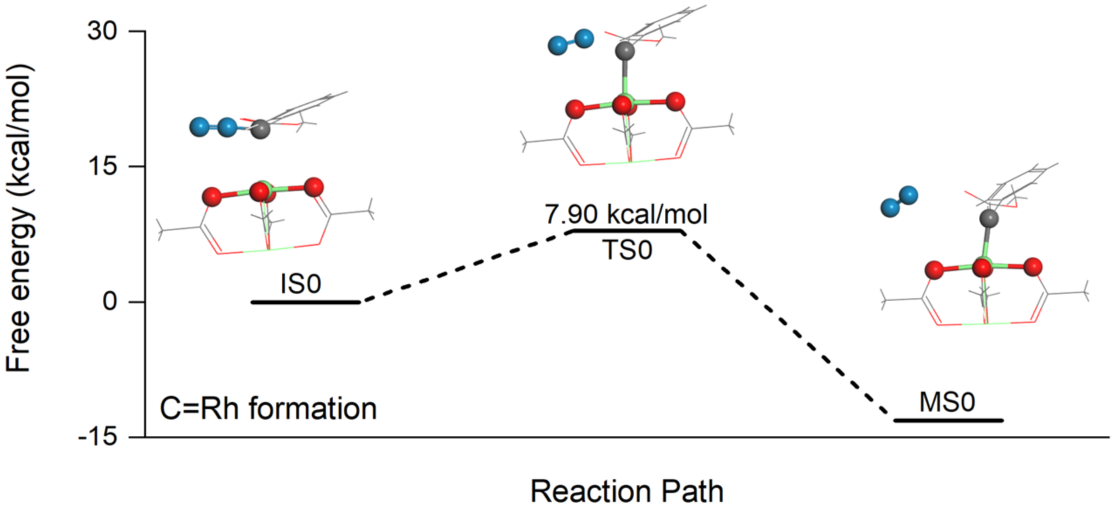

Figure S19. Reaction process of C=Rh formation on homogeneous $Rh_2(OAc)_4$ catalyst with the corresponding free energy and structures. White, red, gray, blue, orange, and green balls indicate H, O, C, N and Rh atoms.

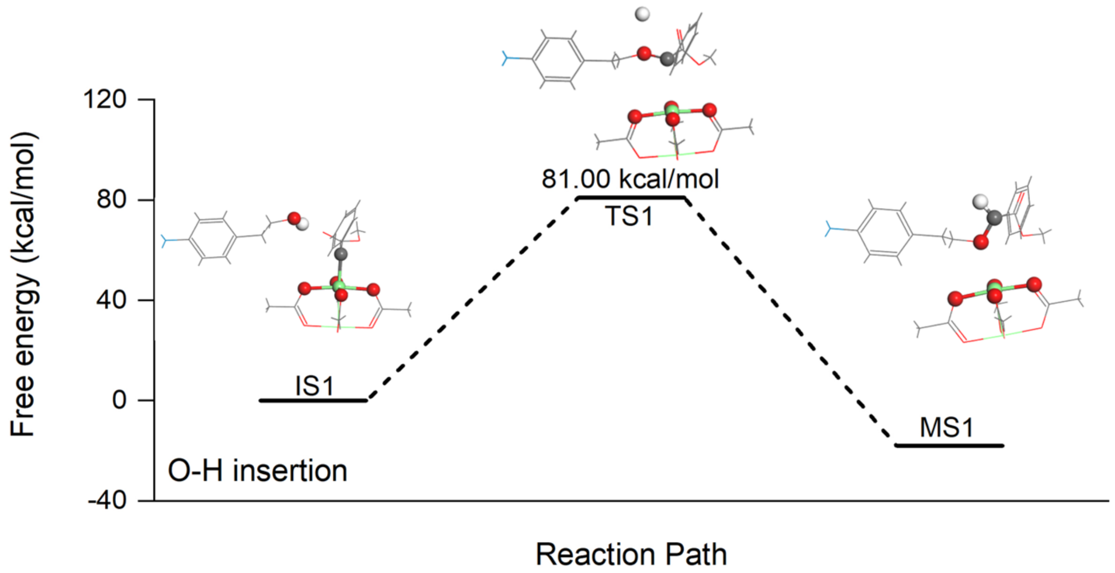

Figure S20. Reaction process of O-H insertion on homogeneous $Rh_2(OAc)_4$ catalyst with the corresponding free energy and structures. White, red, gray, blue, orange, and green balls indicate H, O, C, N and Rh atoms.

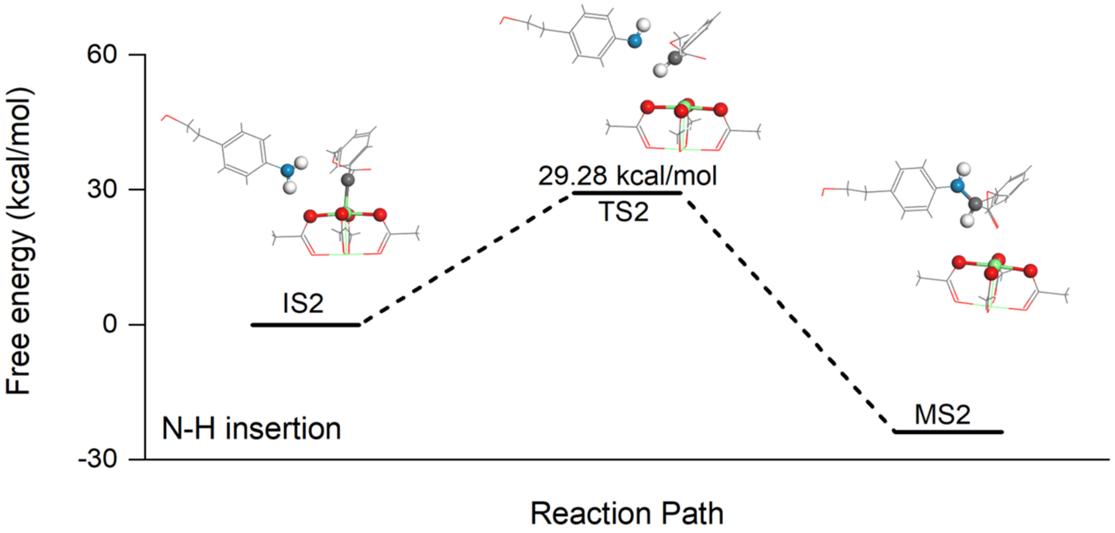

Figure S21. Reaction process of N-H insertion on homogeneous $Rh_2(OAc)_4$ catalyst with the corresponding free energy and structures. White, red, gray, blue, orange, and green balls indicate H, O, C, N and Rh atoms.

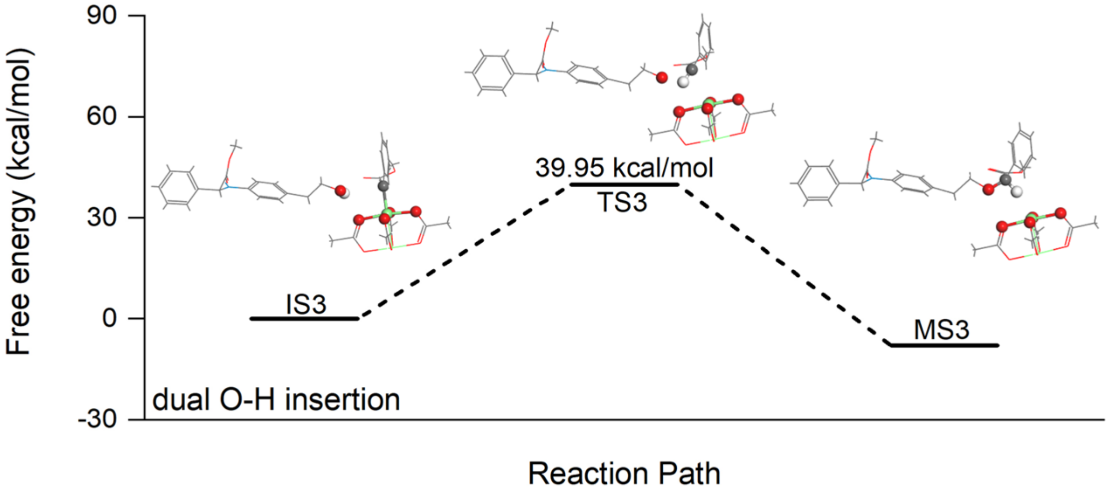

Figure S22. Reaction process of dual O-H insertion on homogeneous $Rh_2(OAc)_4$ catalyst with the corresponding free energy and structures. White, red, gray, blue, orange, and green balls indicate H, O, C, N and Rh atoms.

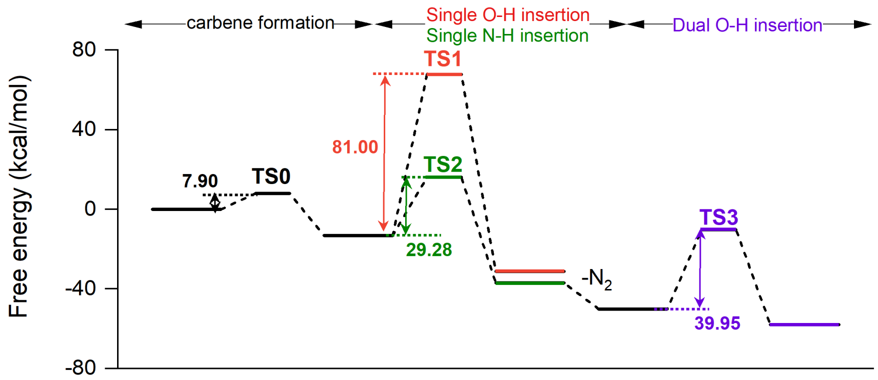

Figure S23. Free energy diagram for the selective N-H bond insertion in heterogeneous $Rh_2(OAc)_4$ catalyst.

Table S4. Bader charge analysis of key elements (Rh, C, H, P, N, and O) during the reaction processes using $Rh_2(OAc)_4$.

	Rh	C	H	N	O
IS0	+0.98	+0.32			
TS0	+1.05	-0.01			
MS0	+1.12	-0.06			
IS1	+1.10	-0.09	+0.64		-1.17
TS1	+0.95	+0.52	+0.01		-1.03
MS1	+1.02	+0.45	+0.08		-1.04
IS2	+1.14	-0.08	+0.48	-1.26	
TS2	+0.98	-0.15	+0.17	-1.02	
MS2	+1.04	+0.40	+0.09	-1.18	
IS3	+1.08	-0.07	+0.60		-1.12
TS3	+0.96	-0.12	+0.24		-0.85
MS3	+0.95	+0.51	+0.06		-1.02

*(4) DFT calculation with **Rh-N₄** catalyst*

For better understanding the role of P atom in Rh-SA, we prepared the anoloug *Rh-N_4* catalyst without P on support via same procedure. The characterization of this catalyst is performed and illustrated in **Figure S24**. The catalytic performance of Rh-N_4 system was valued in the reaction between diazo ester **1a** and **2m**, affording **3am** in 18% yield with high selectivity of single N-H insertion (*see* **Figure S25**).

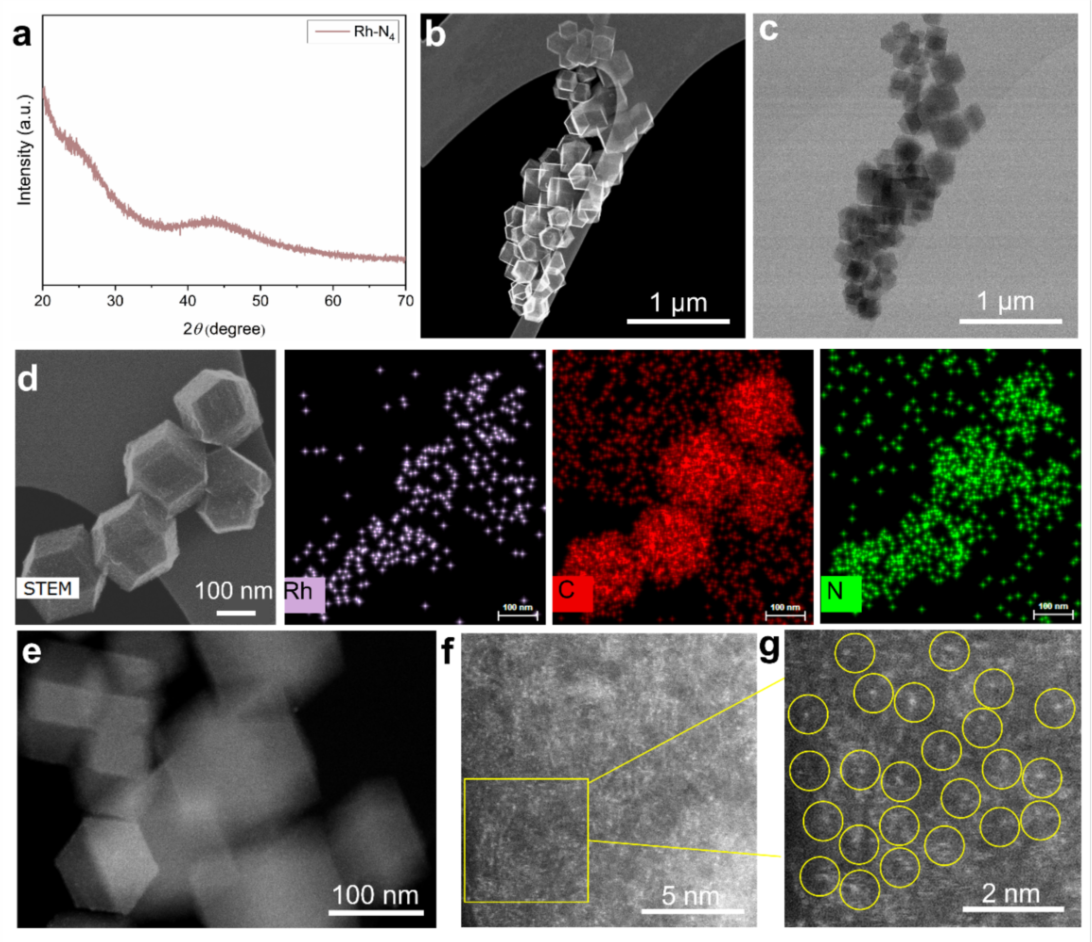

Figure S24. Structural characterizations of Rh-N_4 catalyst as a control sample. a, XRD pattern; b, STEM secondary electron image; c, STEM-bright field image of Rh-N_4; d, STEM secondary electron image and the corresponding EDS mappings of Rh-N_4 (Rh, pink; C, red; N, green); e, HAADF-STEM image of Rh-N_4; f-g, AC HAADF-STEM image (f) and enlarged AC HAADF-STEM image (g) of Rh-N_4.

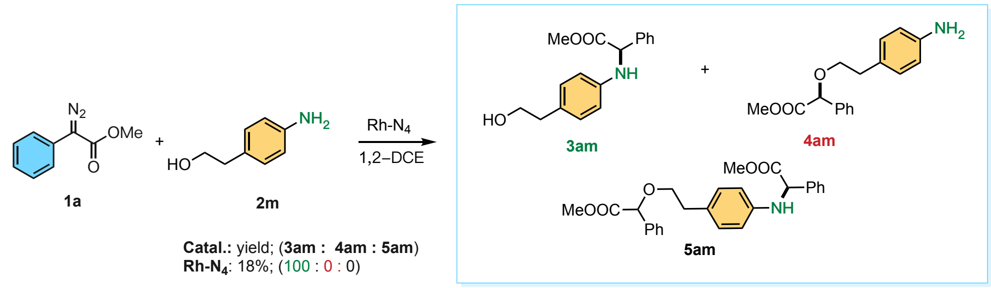

Figure S25. Catalytic study of Rh-N_4 catalyst for comparison.

DFT calculations with a Rh-N_4 system indicated that the corresponding free energy

barrier values for single O-H insertion, single N-H insertion, and dual O-H insertion are 37.94, 27.76, and 38.50 kcal/mol, respectively. These barrier values are much larger than those (25.95, 21.31, and 33.68 kcal/mol) of Rh-N_3P system. These results suggested Rh-N_4 system exhibited lower catalytic activity but comparable selectivity in single N-H insertion, which is consisted with experimental results.

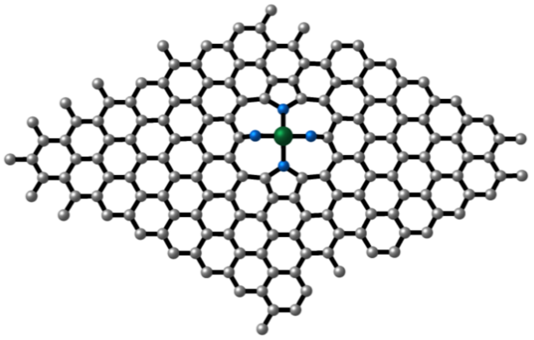

Figure S26. Schematic model of Rh-N_4 was established by DFT calculations (Rh, light green; N, light blue; C, gray).

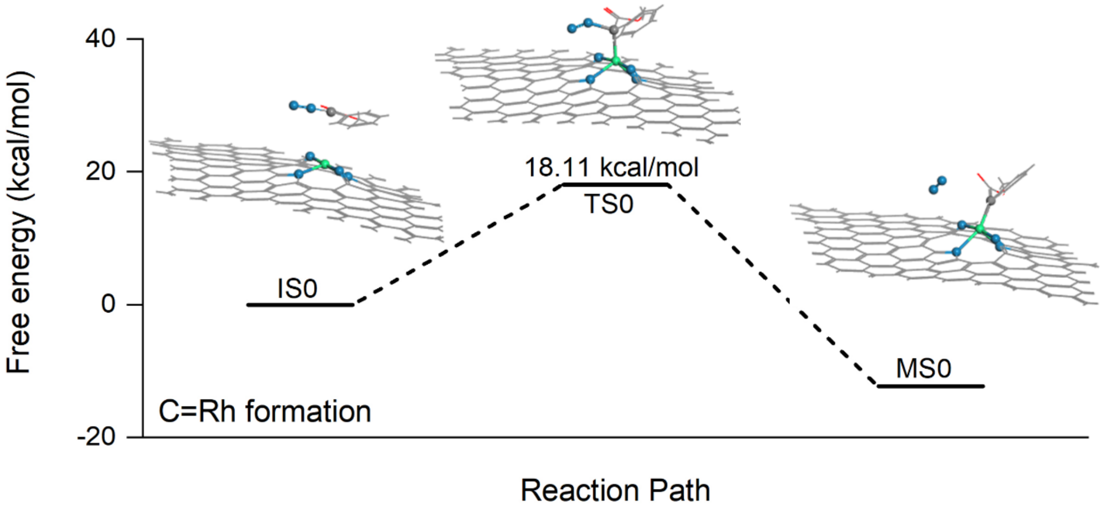

Figure S27. Reaction process of C=Rh formation on heterogeneous Rh-N_4 catalyst with the corresponding free energy and structures. White, red, gray, blue, and green balls indicate H, O, C, N, and Rh atoms.

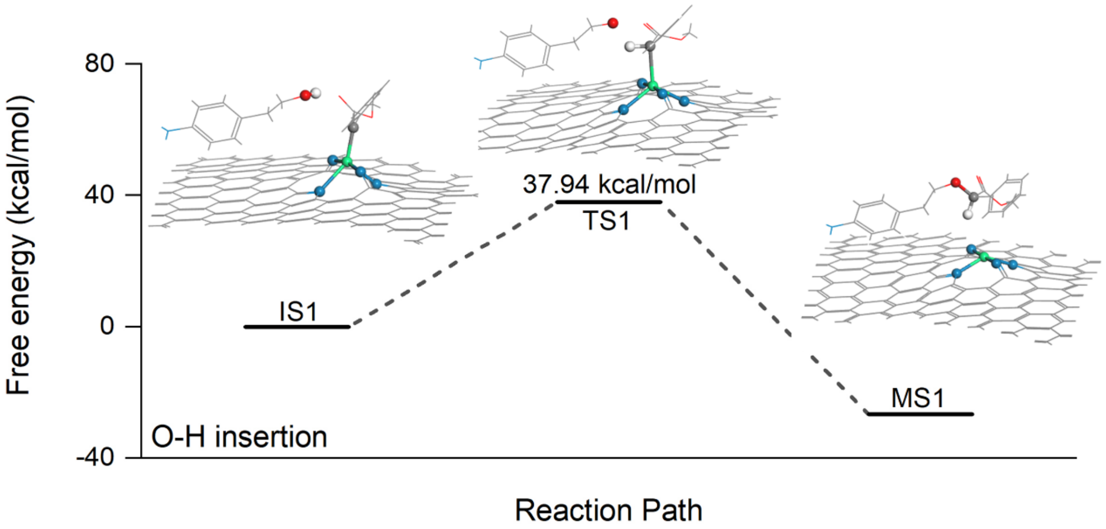

Figure S28. Reaction process of O-H insertion on heterogeneous Rh-N_4 catalyst with the corresponding free energy and structures. White, red, gray, blue, and green balls indicate H, O, C, N, and Rh atoms.

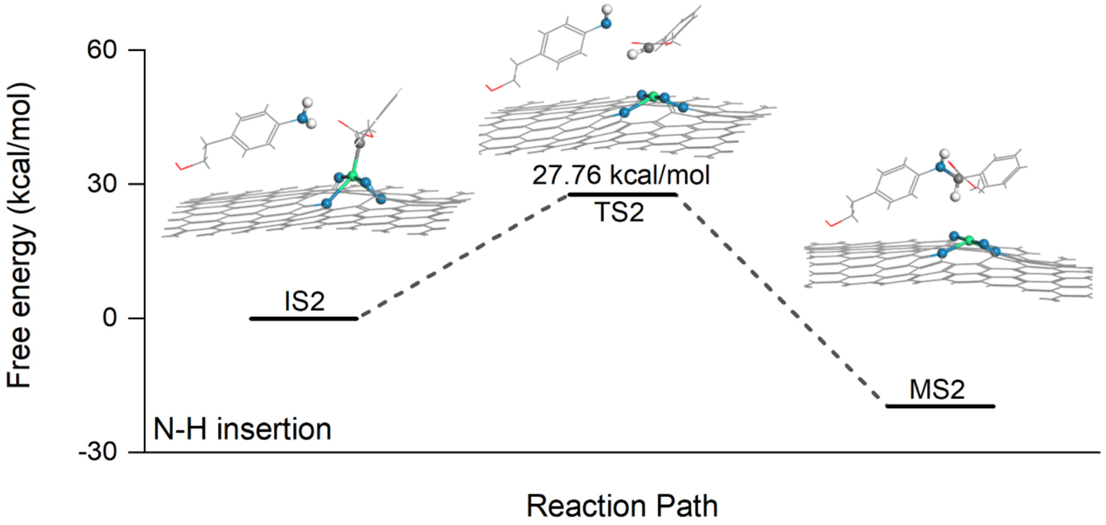

Figure S29. Reaction process of N-H insertion on heterogeneous Rh-N_4 catalyst with the corresponding free energy and structures. White, red, gray, blue, and green balls indicate H, O, C, N, and Rh atoms.

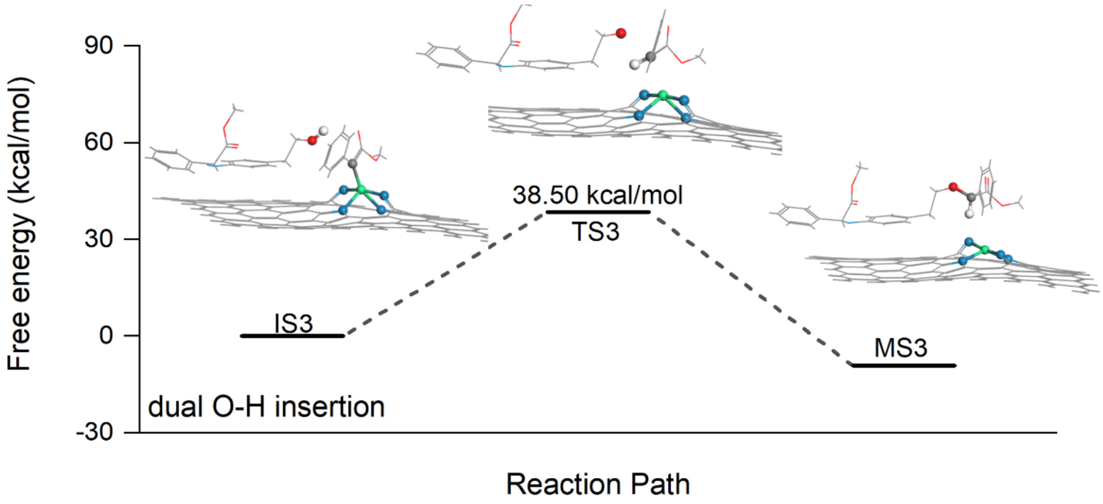

Figure S30. Reaction process of dual O-H insertion on heterogeneous Rh-N_4 catalyst with the corresponding free energy and structures. White, red, gray, blue, and green balls indicate H, O, C, N, and Rh atoms.

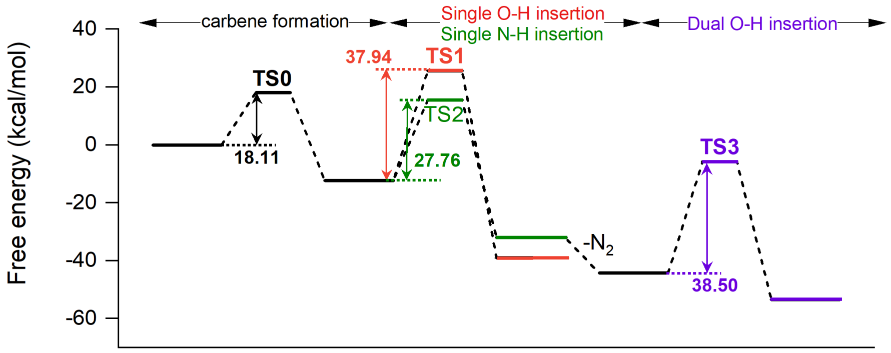

Figure S31. Free energy diagram for the selective N-H bond insertion with heterogeneous Rh-N_4 catalyst.

Table S5. Bader charge analysis of key elements (Rh, C, H, P, N, and O) during the reaction processes using Rh-N_4 catalyst.

	Rh	C	H	N	O
IS0	+1.09	+0.39			
TS0	+1.11	-0.01			
MS0	+1.11	-0.13			
IS1	+1.06	-0.12	+0.60		-1.13
TS1	+1.12	-0.16	+0.23		-0.89
MS1	+1.09	+0.50	+0.05		-1.03
IS2	+1.07	-0.12	+0.49	-1.25	
TS2	+1.11	-0.02	+0.11	-1.01	
MS2	+1.08	+0.37	+0.07	-1.04	
IS3	+1.19	-0.07	+0.61		-1.13
TS3	+1.17	-0.13	+0.20		-0.84
MS3	+1.08	+0.51	+0.04		-1.02

To clarify the role of P atom, the atomic and electronic structures of **TS2** for in both systems were analyzed, as shown in **Figure S32**. For the system of Rh-N_3P, the distance between P and the transferred H atoms is 2.80 Å, which is within the van der Waals force. Moreover, the charge density difference indicates the electron distributions of P atom and the transferred H atoms are opposite, resulting in the electrostatic attraction between P and the transferred H atoms. The addition electrostatic P-H interaction stabilizes the **TS2**, thus accelerate the X-H insertion process and enhanced the efficiency of product generation.

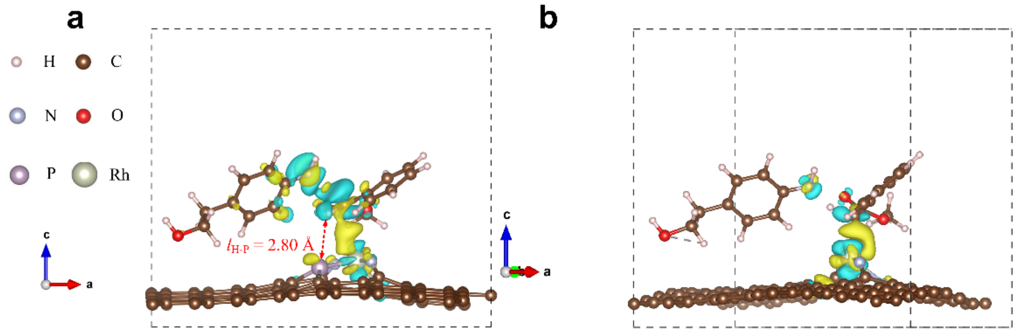

Figure S32. Electron density difference of **TS2** for the systems of Rh-N_3P (a) and Rh-N_4 (b). The loss and enrichment of electrons are indicated in blue and yellow.

*(5) DFT calculation with **Rh-N$_3$P-pyridine** catalyst*

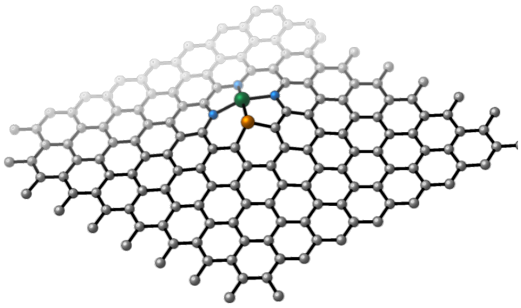

Figure S33. Schematic model of Rh-N$_3$P-pyridine catalyst was established by DFT calculations (Rh, light green; N, light blue; C, gray).

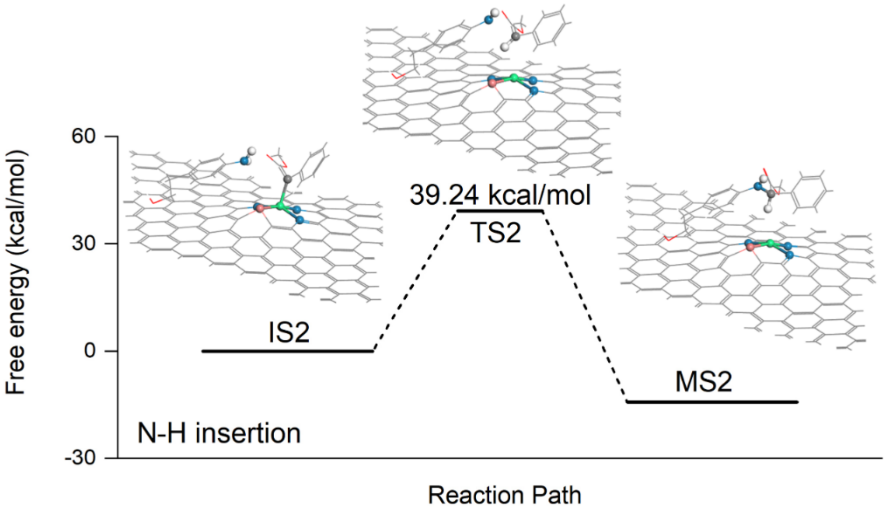

Figure S34. Free energy diagram for single N-H bond insertion with Rh-N$_3$P-pyridine catalyst.

Supplementary mechanistic experiment

Section 1. Kinetic profiles with variation of amount of 1a, 2a and Rh-SA

The relative rates were experimentally calculated by changing the concentration of aniline **2a** with excess of diazo ester **1a (Fig. S35- S38)**, changing the concentration of diazo ester **1a** with excess of aniline **2a** (**Fig. S39- S42**), as well as changing the concentration of Rh-SA catalyst with excess of substrates (**Fig. S43- S46**), respectively. Samples of reaction were collected regularly at the indicated time, and analyzed by ^{1}H-NMR spectroscopy using 1,3,5-trimethoxybenzene as the internal standard to determine the yielding of **3aa**. All experiments were repeated three times to obtain the average of yields for the establishment of the initial rates versus the concentration of coupling components.

1.1. Kinetic profiles – variation of concentrations of aniline 2a with excess of diazo 1a

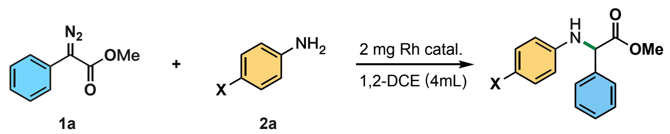

1a
0.25 mmol

2a
0.025/0.0375/0.05 mmol

3aa

Note: In this set of experiments, keeping diazo **1a** (0.25 mmol) in excess, three parallel reactions in variation of amount of aniline **2a** (0.025 / 0.0375 / 0.05 mmol, concentration 6.25 / 9.375 / 12.5 mM, respectively) in anhydrous 1,2-DCE (4.0 mL) at 60 °C were performed. The established kinetic profiles were depicted in **Figures S35- S37**. Upon analysis of data, the reaction rate was shown to be constant regardless variation of concentrations of aniline **2a**. (**Figure S38**)

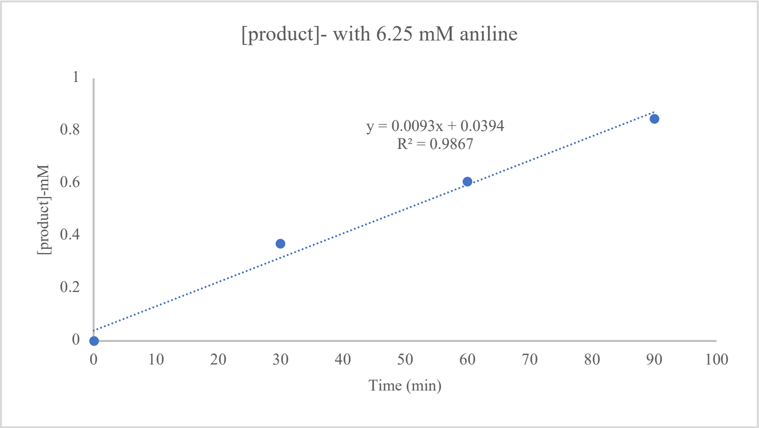

Figure S35. Product formation as a function of time when [aniline **2a**] = **6.25 mM**.

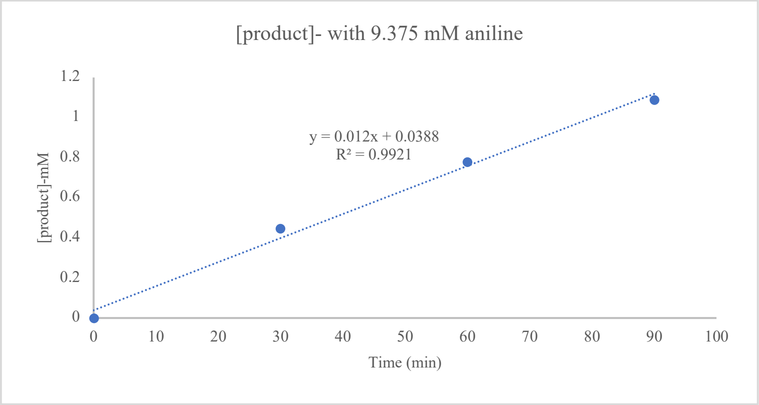

Figure S36. Product formation as a function of time when [aniline **2a**] = **9.375 mM**.

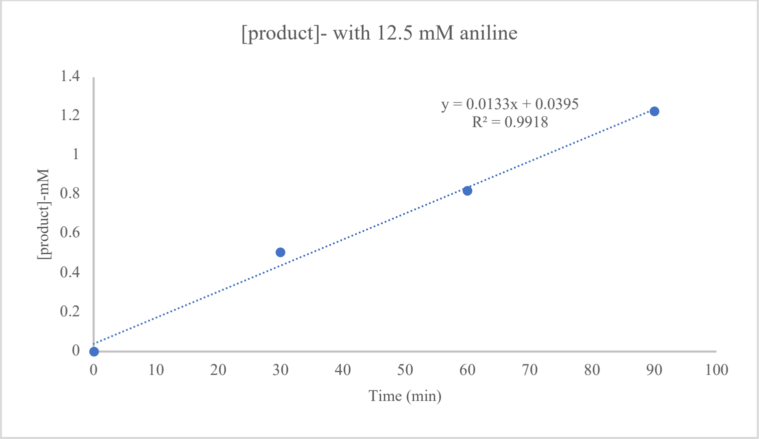

Figure S37. Product formation as a function of time when [aniline **2a**] = **12.5 mM**.

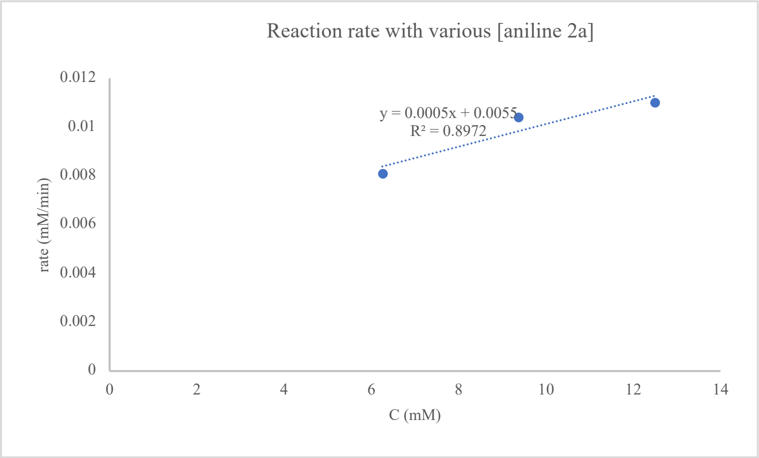

Figure S38. Rate of product formation as a function of [aniline **2a**].

1.2. Kinetic profiles – variation of concentrations of diazo 1a with excess of aniline 2a

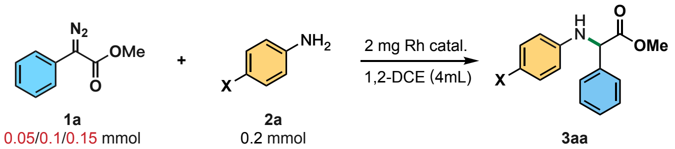

Note: In this set of experiments, keeping aniline **2a** (0.2 mmol) in excess, three parallel reactions in variation of amount of diazo **1a** (0.05 / 0.10 / 0.15 mmol, concentration 12.5 / 25 / 37.5 mM,

respectively) in anhydrous 1,2-DCE (4.0 mL) at 60 ºC were performed. The established kinetic profiles were depicted in **Figure S39- S41.** Upon analysis of data, reaction initial rate apparently was influenced by the concentration of diazo **1a** as demonstrated with a positive slope depicted in **Figure S42**.

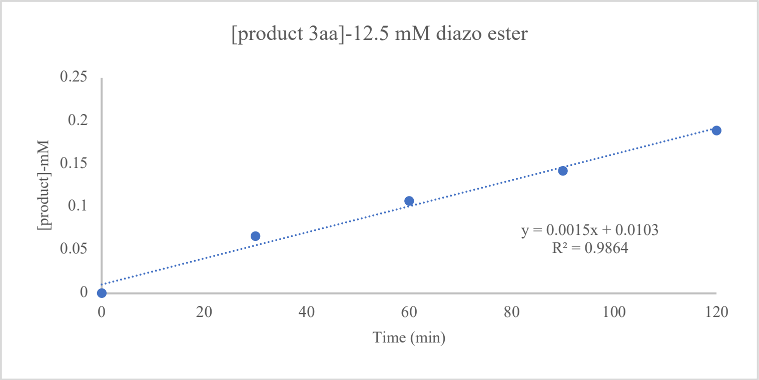

Figure S39. Product formation as a function of time when [diazo **1a**] = **12.5 mM**.

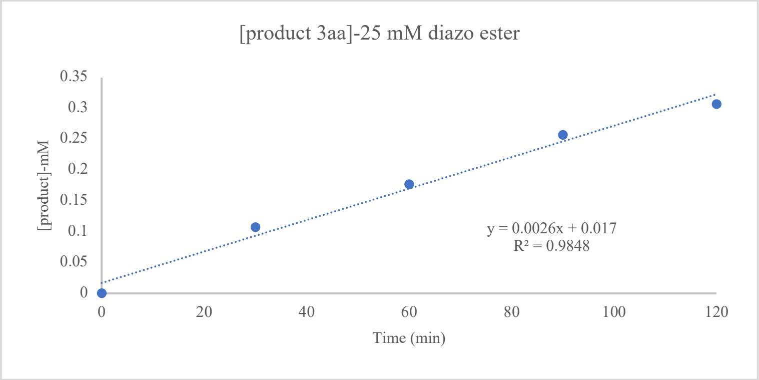

Figure S40. Product formation as a function of time when [diazo **1a**] = **25 mM**.

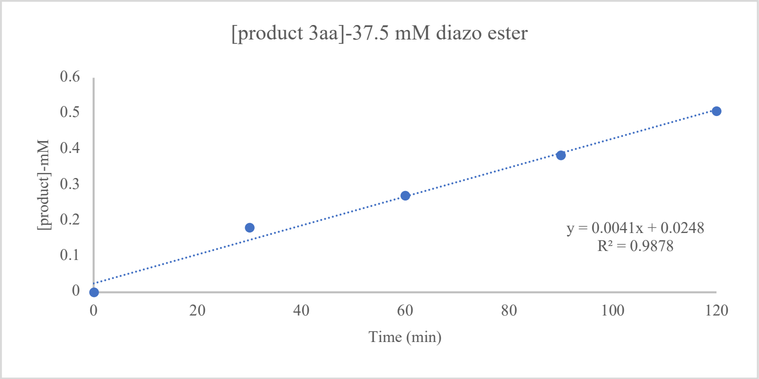

Figure S41. Product formation as a function of time when [diazo **1a**] = **37.5 mM**.

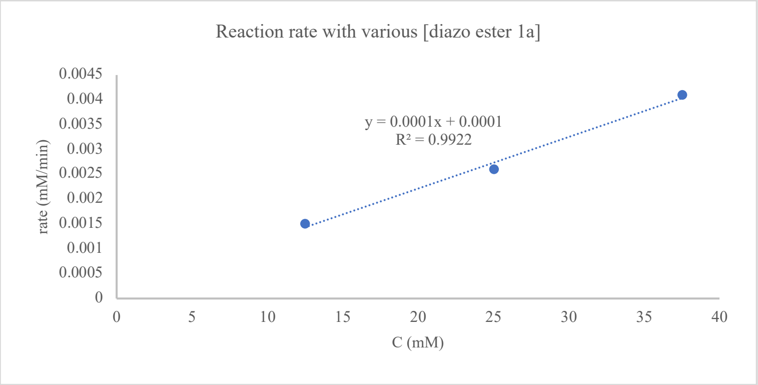

Figure S42. Rate of product formation as a function of [diazo **1a**]

1.3. Kinetic profiles – variation of concentrations of Rh-SA with excess of substrates

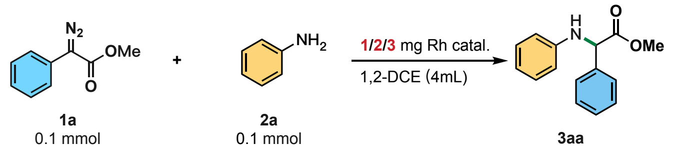

Note: In this set of experiments, keeping diazo **1a** (0.1 mmol) and aniline **2a** (0.1 mmol) in excess, three parallel reactions in variation of amount of Rh-SA (1 / 2 / 3 mg, respectively) in anhydrous 1,2-DCE (4.0 mL) at 60 ºC were performed. The established kinetic profiles were depicted in **Figures S43-S45.** Upon analysis of data, the reaction rate was shown to be constant regardless variation of concentrations of Rh-SA. (**Figure S46**)

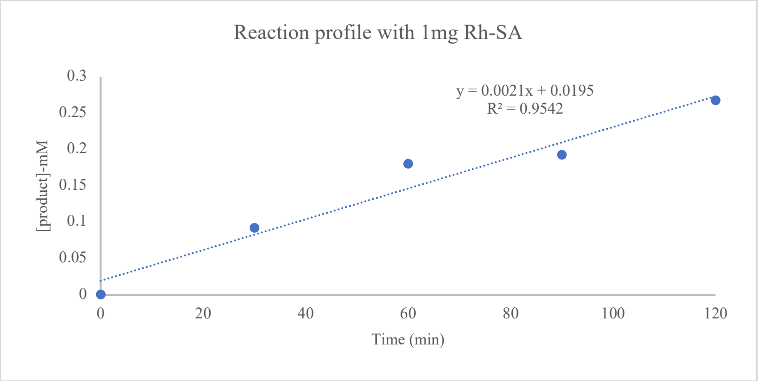

Figure S43. Product formation as a function of time with **1 mg** Rh-SA.

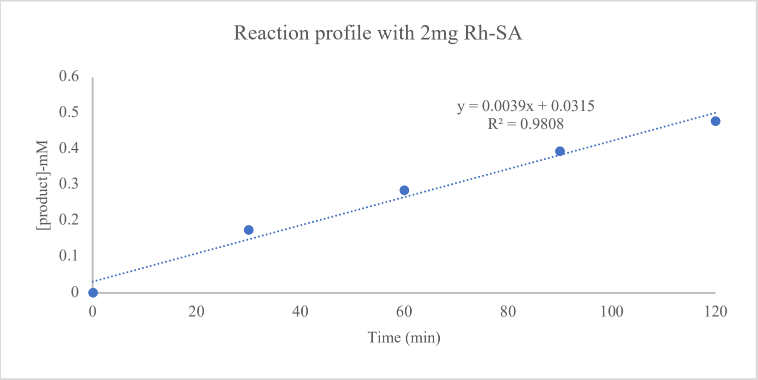

Figure S44. Product formation as a function of time with **2 mg** Rh-SA.

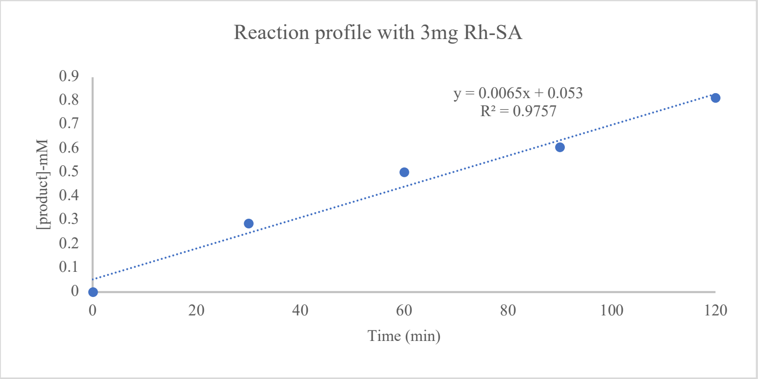

Figure S45. Product formation as a function of time with **3 mg** Rh-SA.

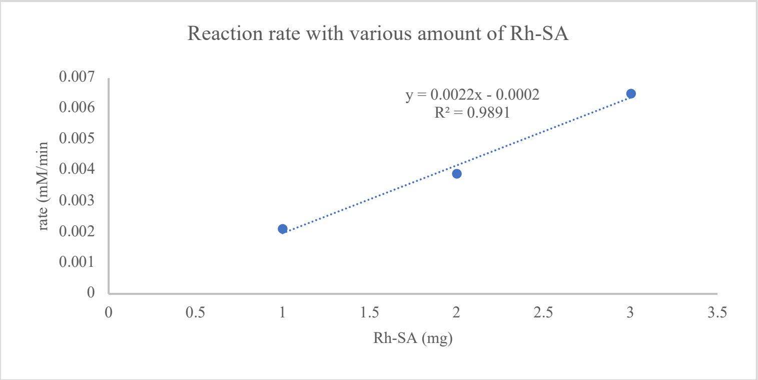

Figure S46. Rate of product formation as a function of various amount of Rh-SA.

Section 2. Hammett-Plot analysis

To gain better understanding on the putative mechanism, we next carried out the Hammett-plot analysis using a set of *para*-substituted anilines and *para*-substituted phenyl diazo esters under the identical procedure: In a N_2 glovebox, a mixture of diazo ester **1** (0.20 mmol), aniline **2** (0.10 mmol) and Rh-SA catalyst (2.0 mg) in anhydrous 1,2-DCE (4.0 mL) was sealed in an 8.0 mL-sized microwave tube. The reaction was further stirred at 60 °C. Samples of reaction were collected regularly at the indicated time, and analyzed by ^{1}H-NMR spectroscopy using 1,3,5-trimethoxybenzene or 1,3-dinitrobenzene as the internal standards to determine the yield of **3**.

2.1. Hammett-Plot with para-substituted anilines

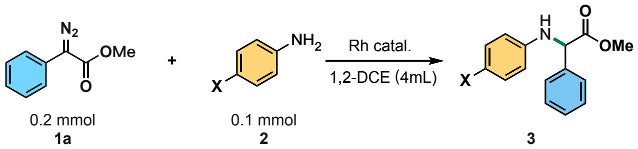

Note: In this set of experiments, keeping phenyl diazo esters **1a** (0.2 mmol) as the carbene precursor, parallel reactions in variation of *para*-substituted anilines **2** (*p*-OMe, *p*-Me, *p*-F, *p*-Br, *p*-COOMe, *p*-CF_3, in a scale of 0.10 mmol) were compared to the case using standard unsubstituted aniline. The established kinetic profiles were depicted in **Figures S47-S53**. Finally, the Hammett-Plot analysis showing the $\log(k_X/k_H)$ against σ values was shown in both **Table S6** and **Figure S54** with a positive ρ value of +1.67.

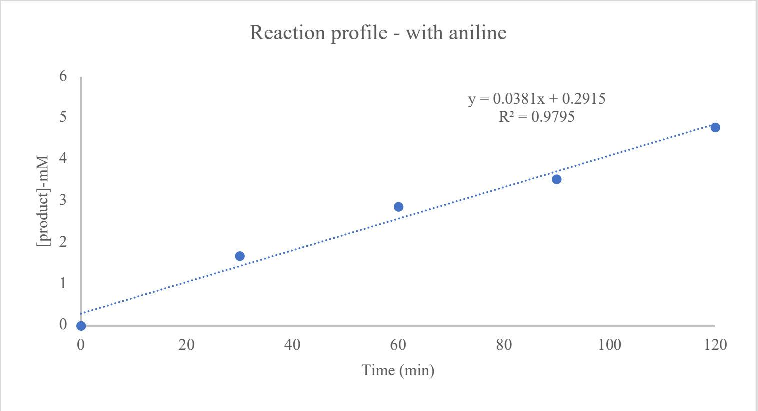

Figure S47. Reaction profile when using diazo ester **1a** and aniline **2a**

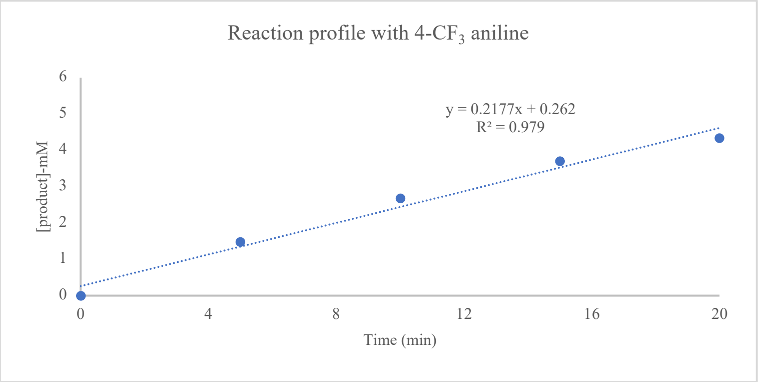

Figure S48. Reaction profile when using diazo ester **1a** and 4-CF_3 aniline

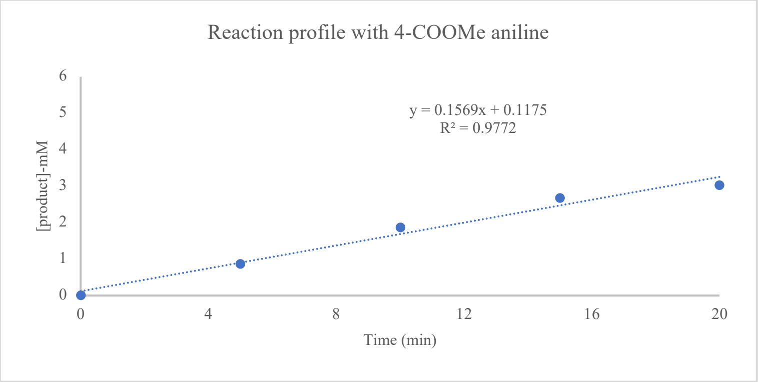

Figure S49. Reaction profile when using diazo ester **1a** and 4-COOMe aniline

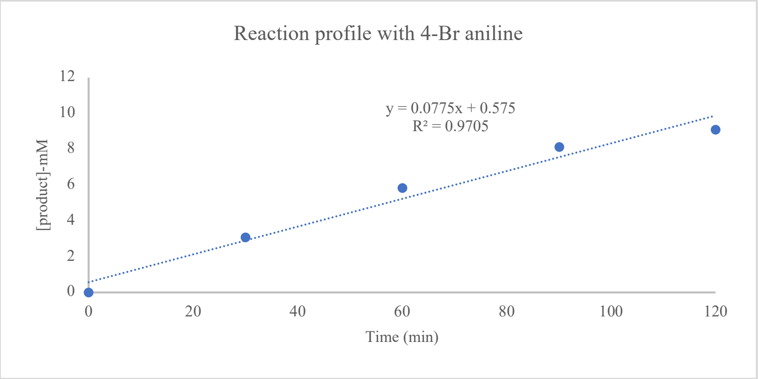

Figure S50. Reaction profile when using diazo ester **1a** and 4-Br aniline

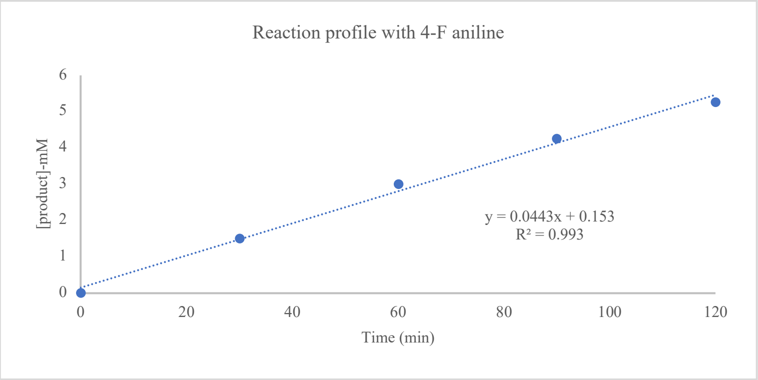

Figure S51. Reaction profile when using diazo ester **1a** and 4-F aniline

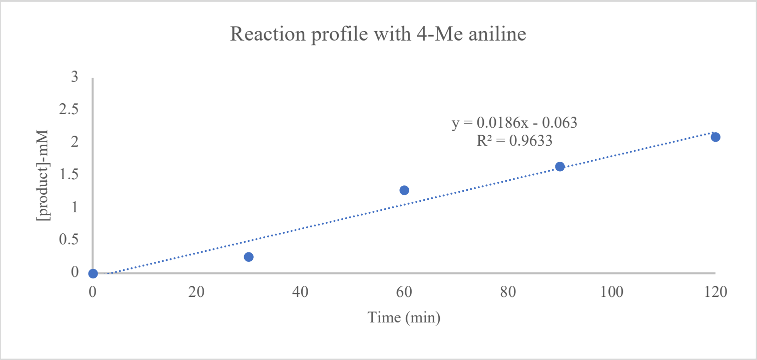

Figure S52. Reaction profile when using diazo ester **1a** and 4-Me aniline

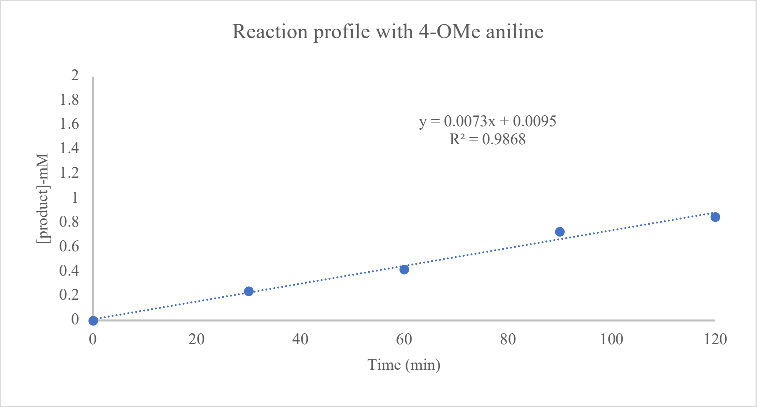

Figure S53. Reaction profile when using diazo ester **1a** and 4-OMe aniline

Table S6. Rate constants and data for Hammett plot with anilines

4-X aniline	σ	Rate (mM/min) vs [product]	K_X/K_H	$\log(K_X/K_H)$
**4-CF_3**	0.54	0.2177	5.713910761	0.756933453
**4-COOMe**	0.45	0.1569	4.118110236	0.614697968
**4-Br**	0.23	0.0775	2.034120735	0.308376727
**4-F**	0.062	0.0443	1.162729659	0.065478751
**4-H**	0	0.0381	1	0
**4-Me**	-0.17	0.0186	0.488188976	-0.311412031
**4-OMe**	-0.27	0.0073	0.191601050	-0.717602116

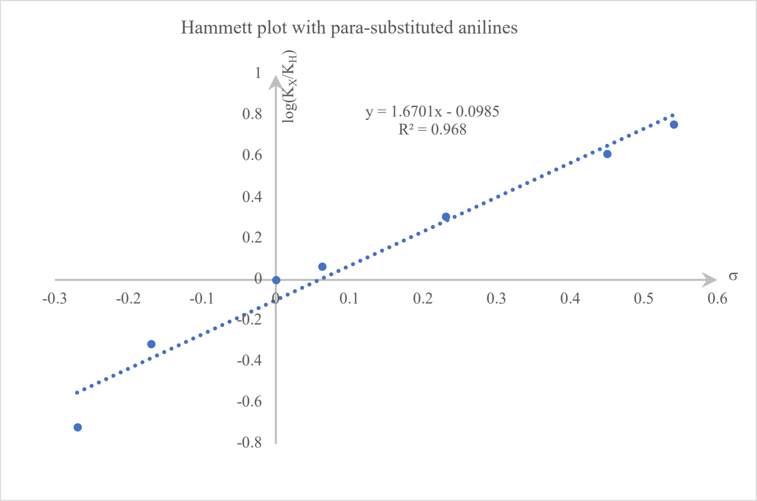

Figure S54. Hammett plot based on the reactions using para-substituted anilines

2.2. Hammett-Plot with *para*-substituted diazo esters

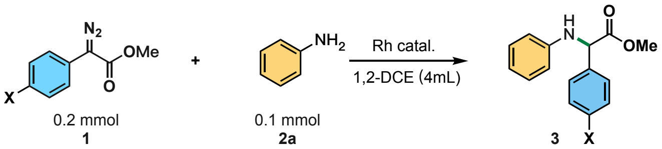

Note: In this set of experiments, keeping anilines **2a** (0.1 mmol) as the coupling partner, parallel reactions in variation of phenyl diazo esters **1** (*p*-OMe, *p*-Me, *p*-F, *p*-Br, in a scale of 0.20 mmol) were compared to the case using the standard phenyl diazo ester **1a**. The established kinetic profiles were depicted in **Figures S55-S59**. Finally, the Hammett-Plot analysis showing the log(k_X/k_H) against σ values was shown in both **Table S7** and **Figure S60** with a negative ρ value of -1.94.

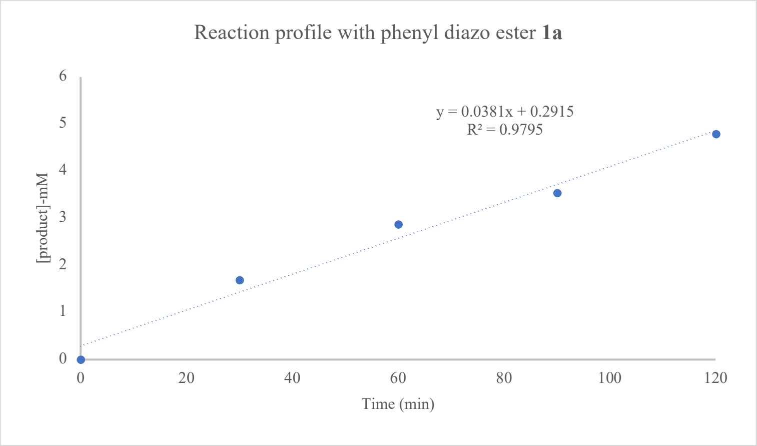

Figure S55. Reaction profile when using diazo ester **1a** and aniline **2a**.

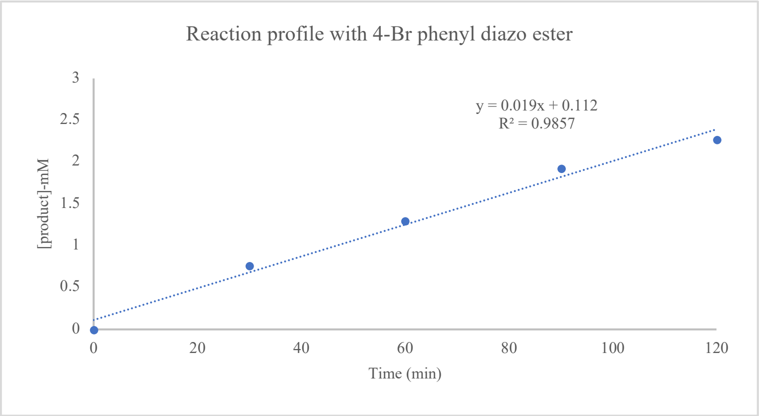

Figure S56. Reaction profile when using 4-Br phenyl diazo ester and aniline **2a**.

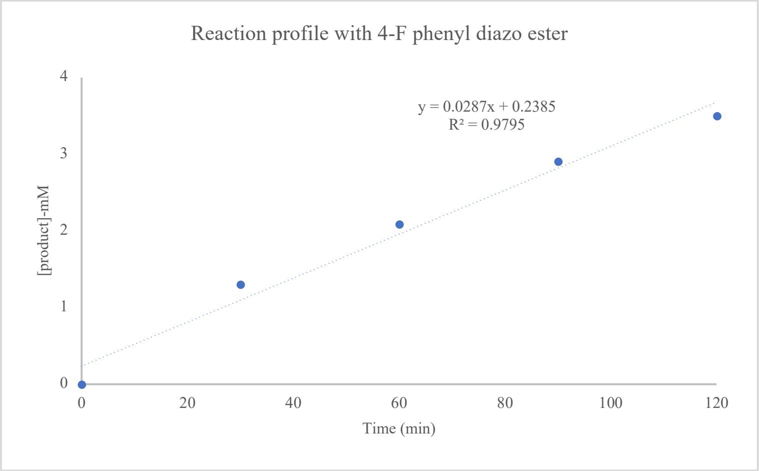

Figure S57. Reaction profile when using 4-F phenyl diazo ester and aniline **2a**.

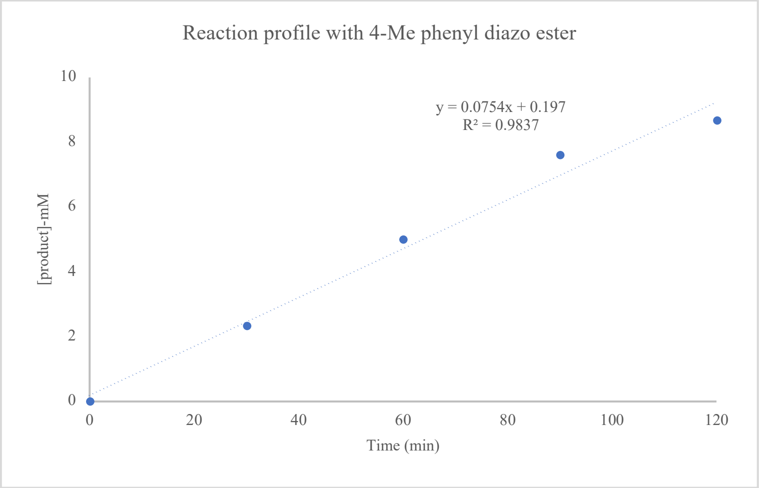

Figure S58. Reaction profile when using 4-Me phenyl diazo ester and aniline **2a**.

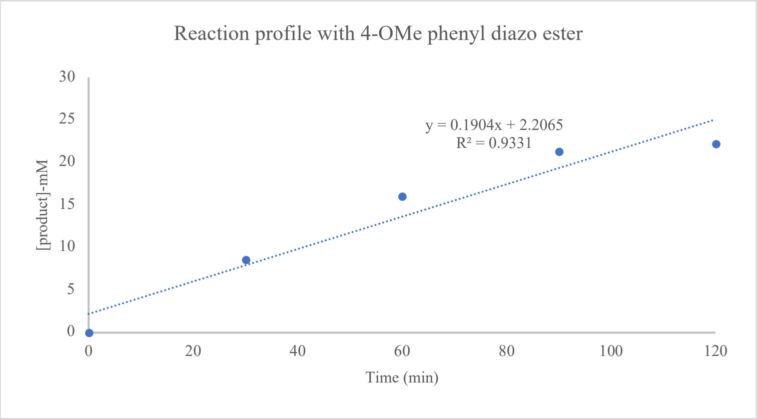

Figure S59. Reaction profile when using 4-OMe phenyl diazo ester and aniline **2a**.

Table S7. Rate constants and data for Hammett plot with diazo esters

4-X phenyl diazo ester	σ	Rate (mM/min) vs [product]	K_X/K_H	$\log(K_X/K_H)$
**4-Br**	0.23	0.019	0.498687664	-0.302171375
**4-F**	0.062	0.0287	0.75328084	-0.123043079
**4-H**	0	0.0381	1	0
**4-Me**	-0.17	0.0754	1.979002625	0.29644637
**4-OMe**	-0.27	0.1904	4.997375328	0.698741968

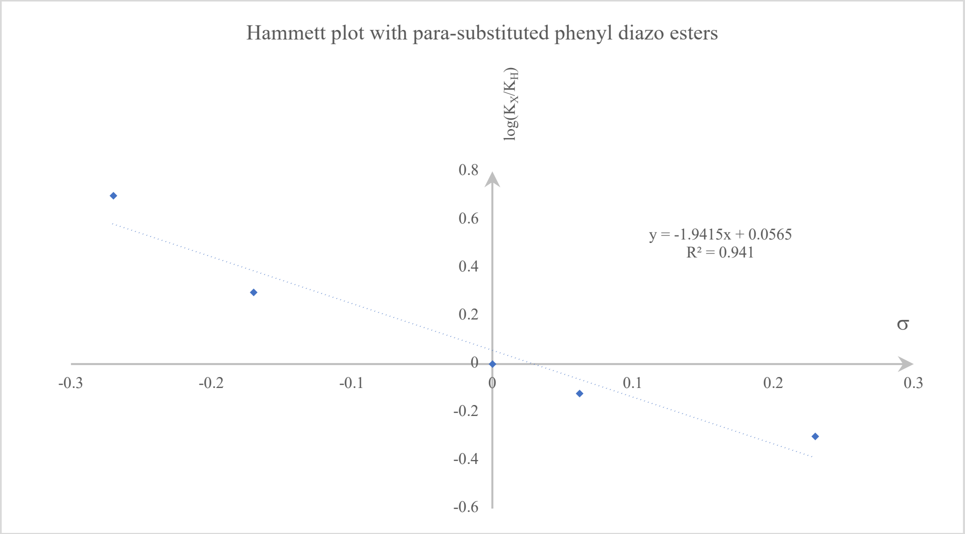

Figure S60. Hammett plot based on the reactions using para-substituted phenyl diazo esters.

Supplementary methods for catalysis

General procedure for heterogeneous catalysis

In glovebox, diazo ester **1x** (0.20 mmol, 2.0 equiv.) and (Hetero)aryl amine **2y** (0.1 mmol, 1.0 equiv.) were added sequentially to a suspension of Rh-SA (2 mg, 0.15 mol%) in 1,2-dichloroethane (0.5 mL to 2 mL) in a two-dram vial. The reaction vial was then sealed and stirred at 60 ºC over 18 hours. After cooling down to room temperature, volatiles were removed by rotary evaporator and the obtained crude mixture was purified on silica gel by flash chromatography using petroleum ether and EtOAc as eluents. The molar ratio of products was determined by ^{1}H-NMR spectroscopy, using trimethoxybenzene (TMB) as in-situ standard.

General procedure for homogeneous catalysis

In glovebox, diazo ester **1x** (0.40 mmol, 2.0 equiv.) and (Hetero)aryl amine **2y** (0.2 mmol, 1.0 equiv.) were added sequentially to a solution of $Rh_2(OAc)_4$ (0.8 mg, 3.6 mol%) in 1,2-dichloroethane (4 mL) in a two-dram vial. The reaction vial was then sealed and stirred at 60 ºC over 18 hours. After cooling down to room temperature, volatiles were removed by rotary evaporator and the obtained crude mixture was purified on silica gel by flash chromatography using petroleum ether and EtOAc as eluents. The molar ratio of products was determined by ^{1}H-NMR spectroscopy, using trimethoxybenzene (TMB) as in-situ standard.

Additional scope studies.

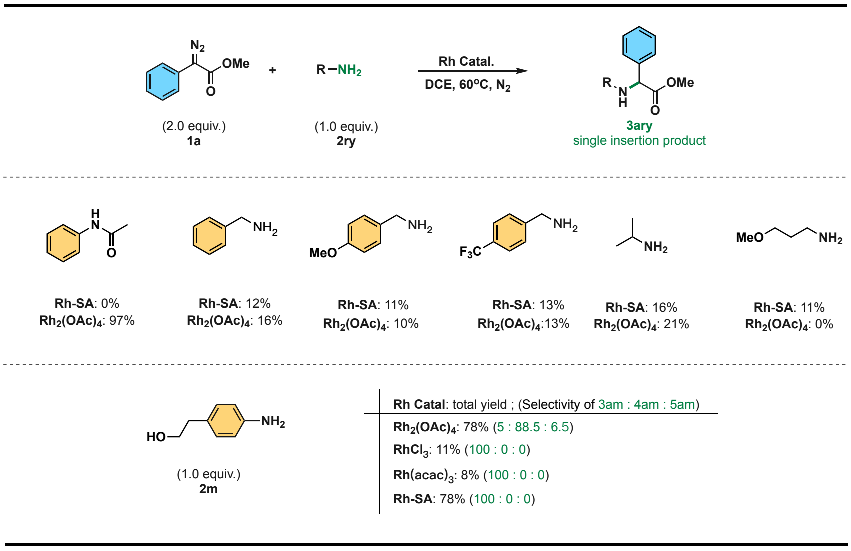

Figure S61. Additional scope study and comparison on different Rh-catalysts.

Supplementary data for reactions with crude NMR analysis

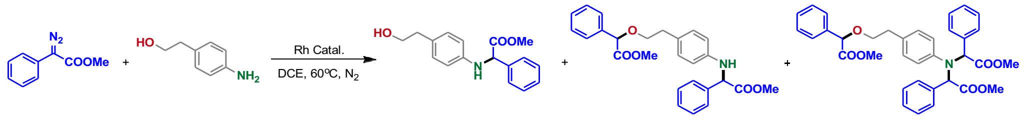

1a 2m 3am 4am 5am

total yield; (3am: 4am:5am)

Rh-SA: 78% yield; (100: 0: 0)

$Rh_2(OAc)_4$: 78% yield; (5: 88.5: 6.5)

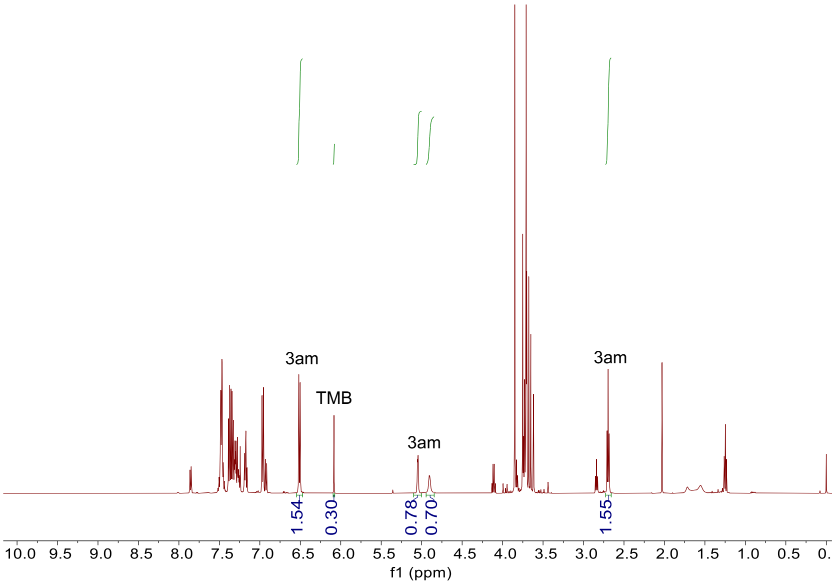

Figure S62. ^{1}H-NMR spectrum of crude reaction mixture using Rh-SA as the catalyst.

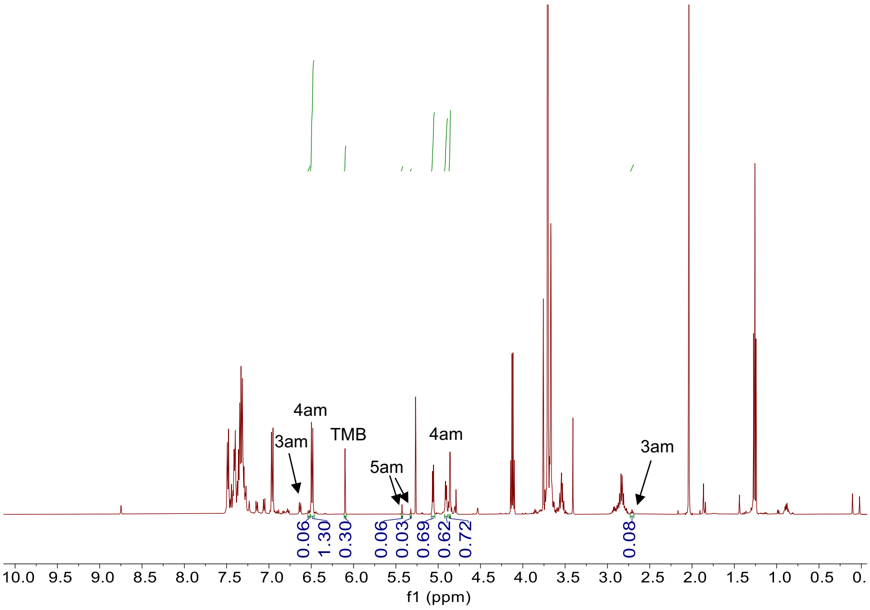

Figure S63. ^{1}H-NMR spectrum of crude reaction mixture using $Rh_2(OAc)_4$ as the catalyst.

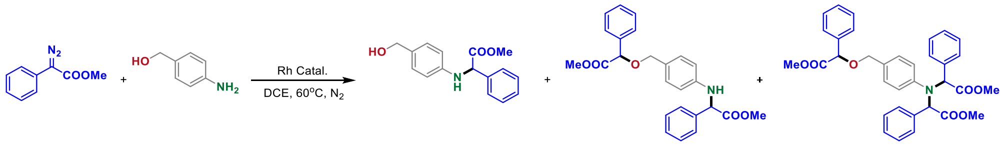

1a **2n** **3an** **4an** **5an**

total yield; (3an: 4an: 5an)

Rh-SA: 80% yield; (100: 0:0)

$Rh_2(OAc)_4$: 79% yield; (0: 76: 24)

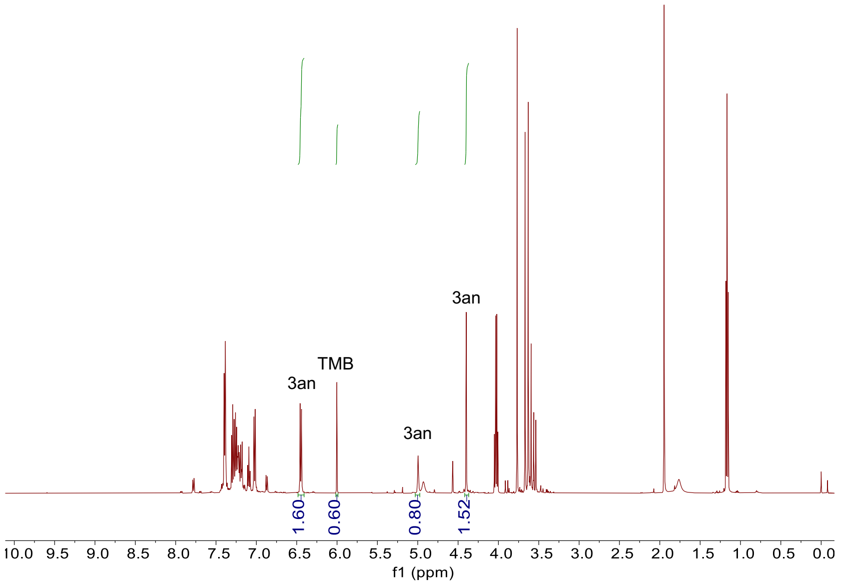

Figure S64. ^{1}H-NMR spectrum of crude reaction mixture using Rh-SA as the catalyst.

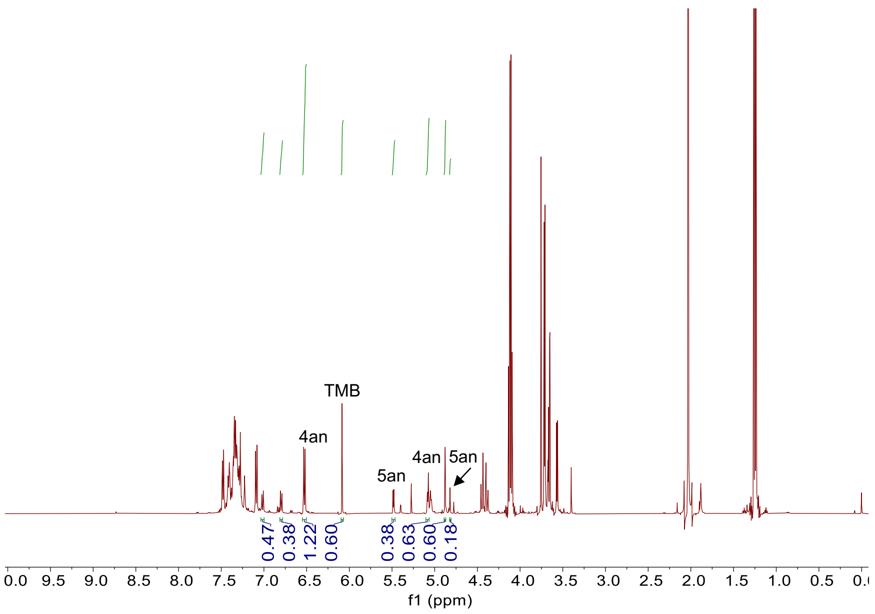

Figure S65. ^{1}H-NMR spectrum of crude reaction mixture using $Rh_2(OAc)_4$ as the catalyst.

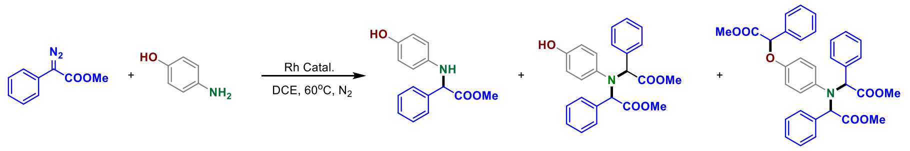

1a **2o** **3ao** **4ao** **5ao**

total yield; (3ao: 4ao: 5ao)

Rh-SA: 70% yield; (100: 0: 0)

$Rh_2(OAc)_4$: 59% yield; (0: 63: 37)

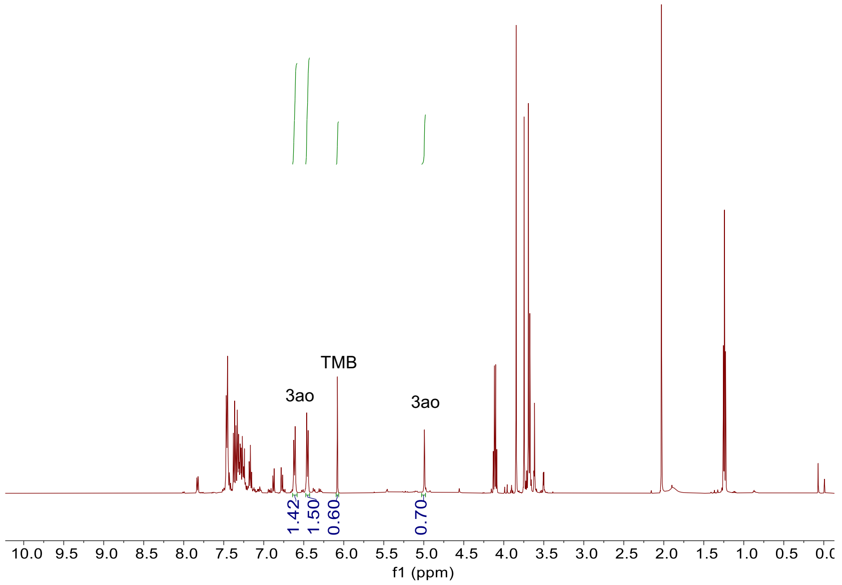

Figure S66. ^{1}H-NMR spectrum of crude reaction mixture using Rh-SA as the catalyst.

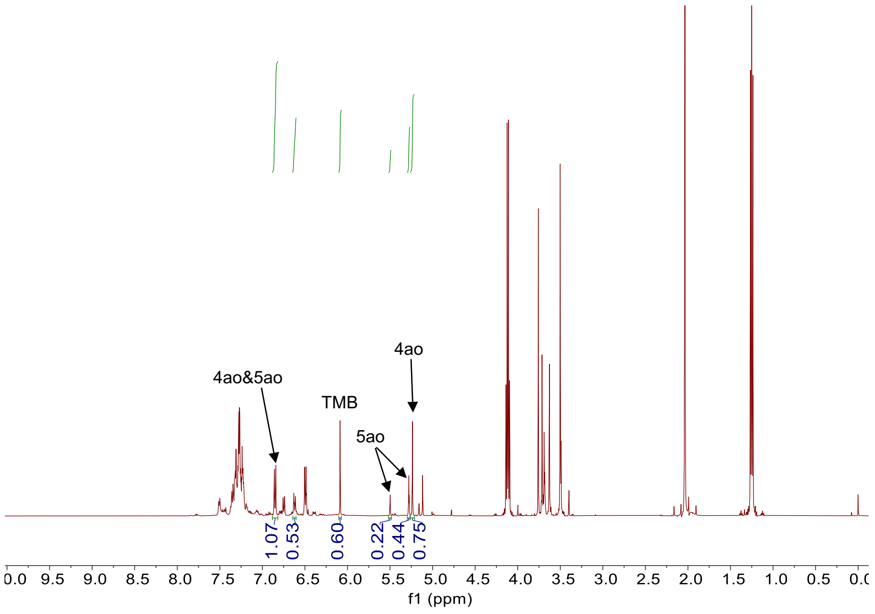

Figure S67. ^{1}H-NMR spectrum of crude reaction mixture using $Rh_2(OAc)_4$ as the catalyst.

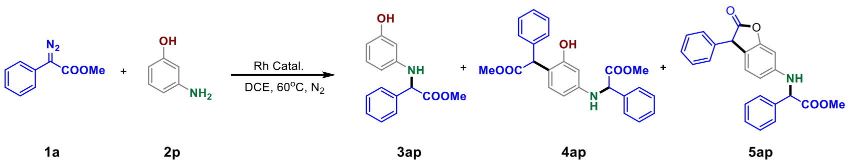

total yield; (3ap: 4ap: 5p)

Rh-SA: 71% yield; (100: 0: 0)

$Rh_2(OAc)_4$: 84% yield; (0: 92: 8)

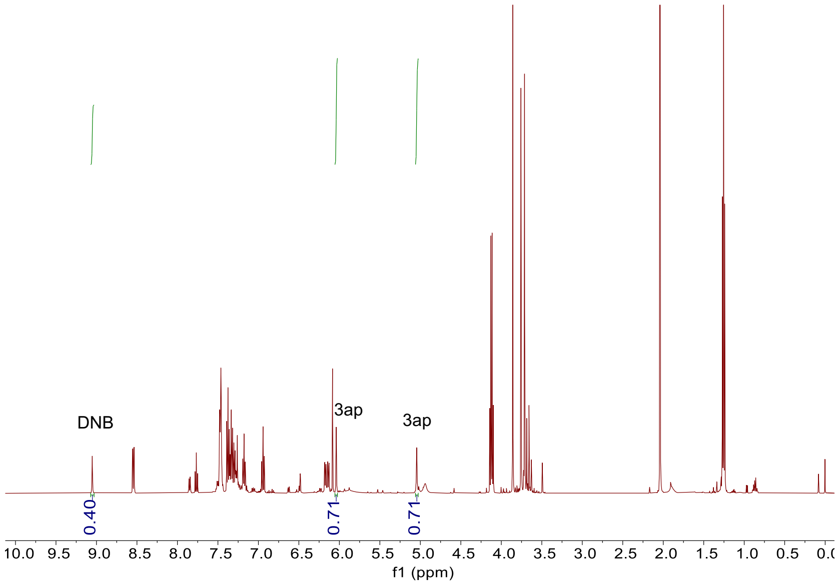

Figure S68. ^{1}H-NMR spectrum of crude reaction mixture using Rh-SA as the catalyst.

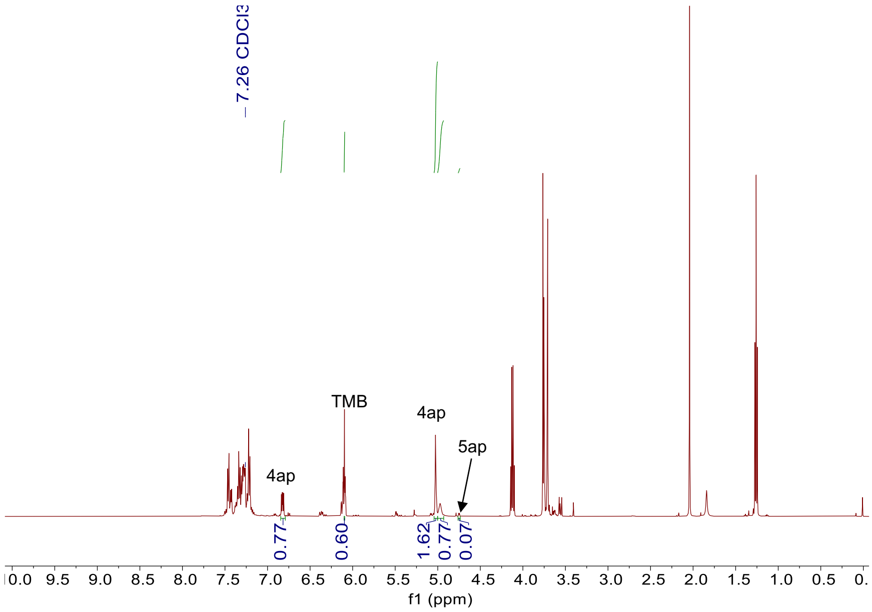

Figure S69. ^{1}H-NMR spectrum of crude reaction mixture using $Rh_2(OAc)_4$ as the catalyst.

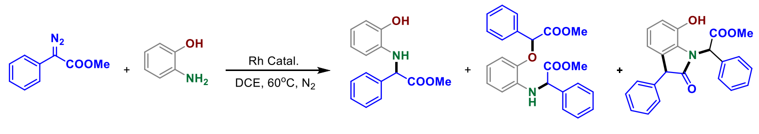

total yield; (3aq: 4aq: 5aq)

Rh-SA: 51% yield; (100: 0: 0)

$Rh_2(OAc)_4$: 55% yield; (0: 80: 20)

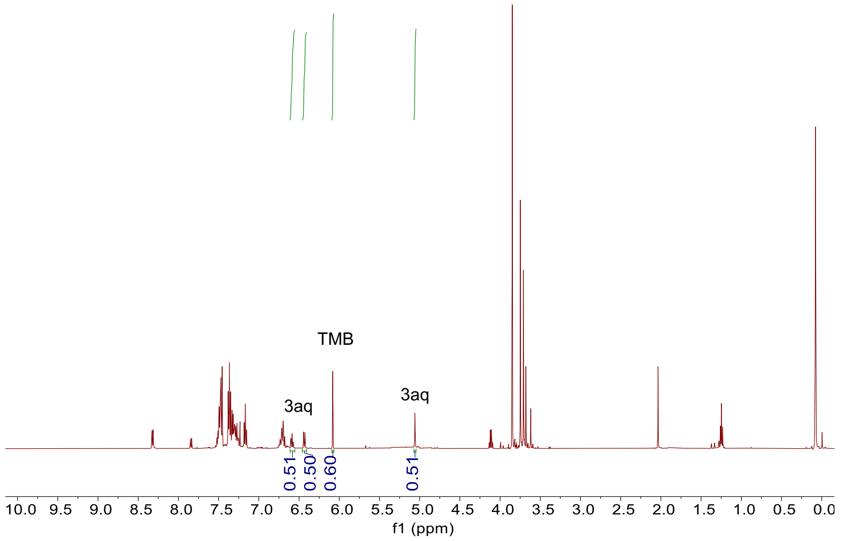

Figure S70. ^{1}H-NMR spectrum of crude reaction mixture using Rh-SA as the catalyst.

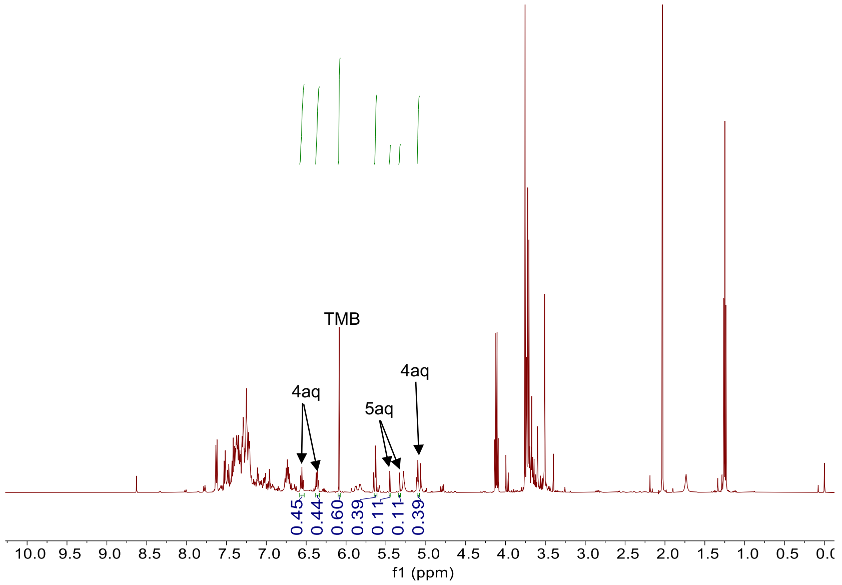

Figure S71. ^{1}H-NMR spectrum of crude reaction mixture using $Rh_2(OAc)_4$ as the catalyst.

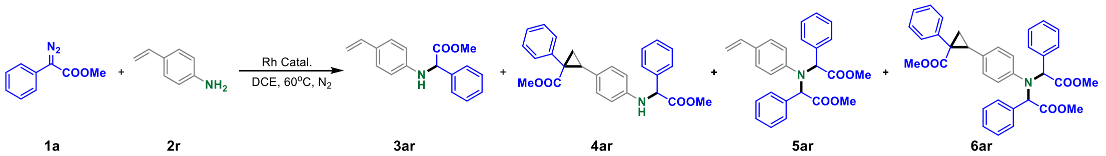

total yield; (3ar: 4ar: 5ar: 6ar)

Rh-SA: 67% yield; (100: 0: 0: 0)

$Rh_2(OAc)_4$: 87% yield; (11.5: 57.5: 4.6: 26.4)

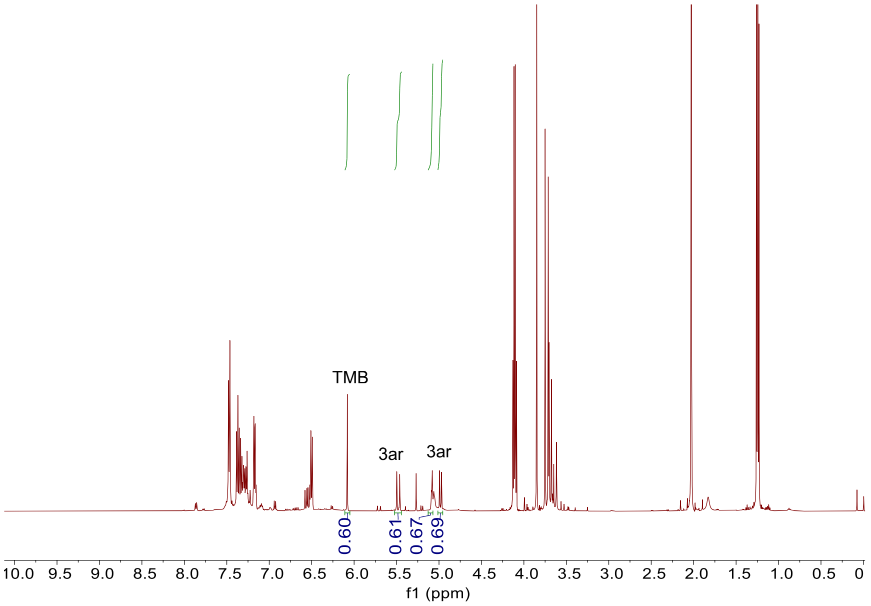

Figure S72. ^{1}H-NMR spectrum of crude reaction mixture using Rh-SA as the catalyst.

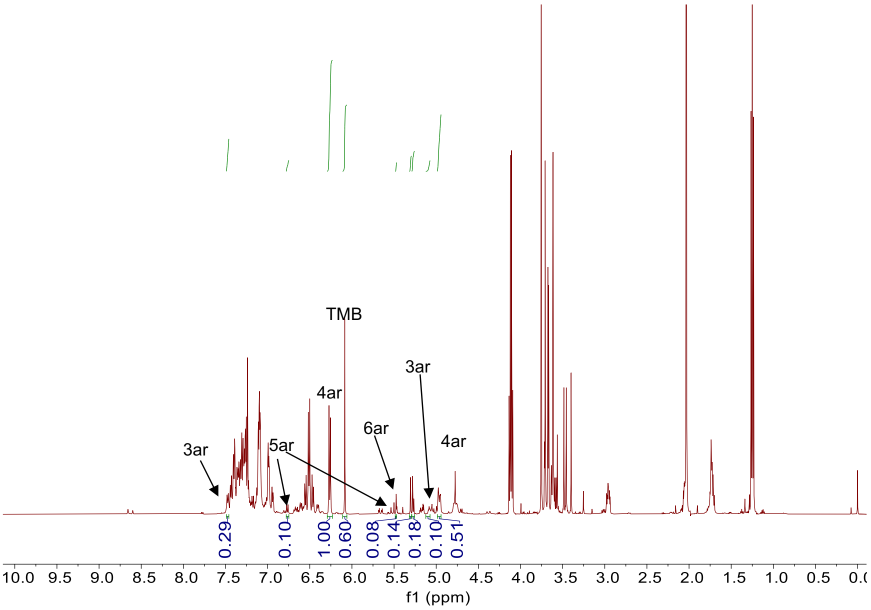

Figure S73. ^{1}H-NMR spectrum of crude reaction mixture using $Rh_2(OAc)_4$ as the catalyst.

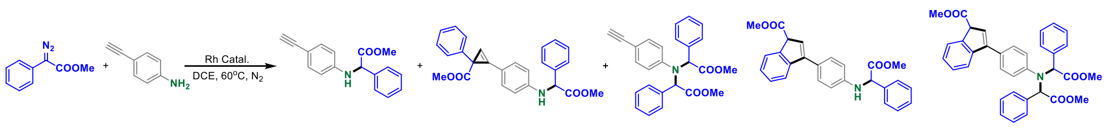

1a 2s 3as 4as 5as 6as 7as

total yield; (3as: 4as: 5as: 6as: 7as)

Rh-SA: 43% yield; (100: 0: 0: 0: 0)

$Rh_2(OAc)_4$: 69% yield; (30.4: 17.4: 4.4: 24.6: 23.2)

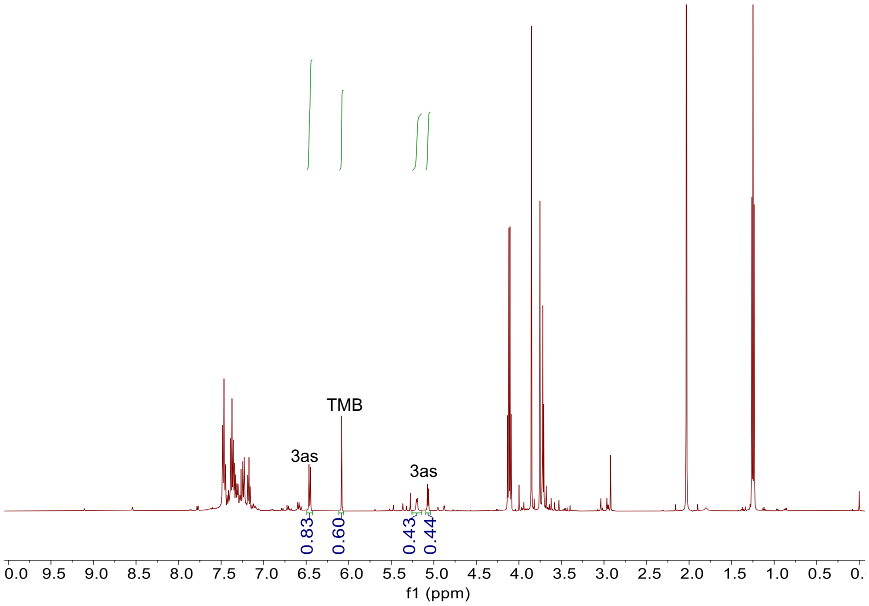

Figure S74. ^{1}H-NMR spectrum of crude reaction mixture using Rh-SA as the catalyst.

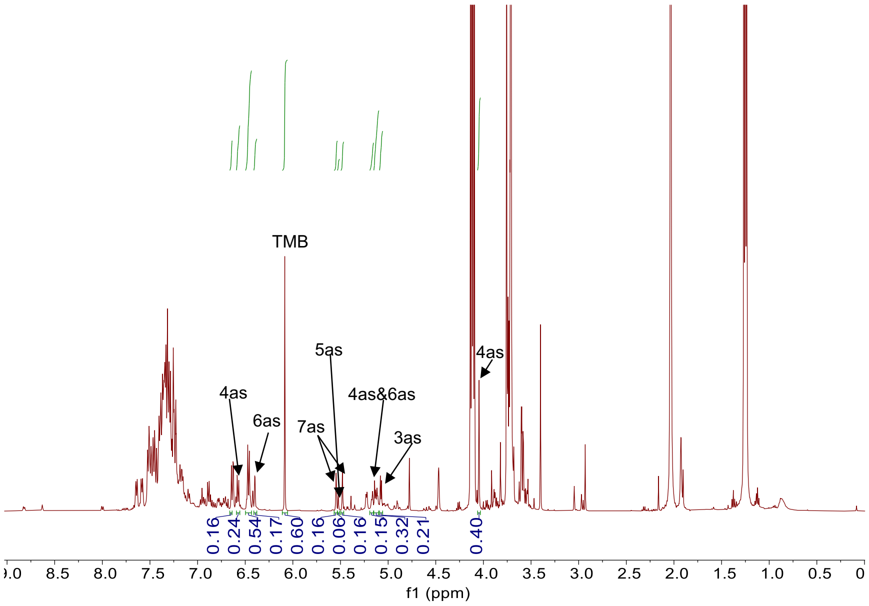

Figure S75. ^{1}H-NMR spectrum of crude reaction mixture using $Rh_2(OAc)_4$ as the catalyst.

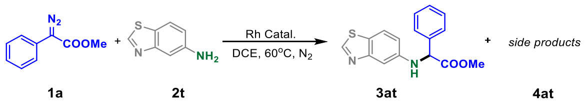

total yield; (3at: 4at)

Rh-SA: 38% yield; (100: 0)

$Rh_2(OAc)_4$: 59% yield; (68: 32)

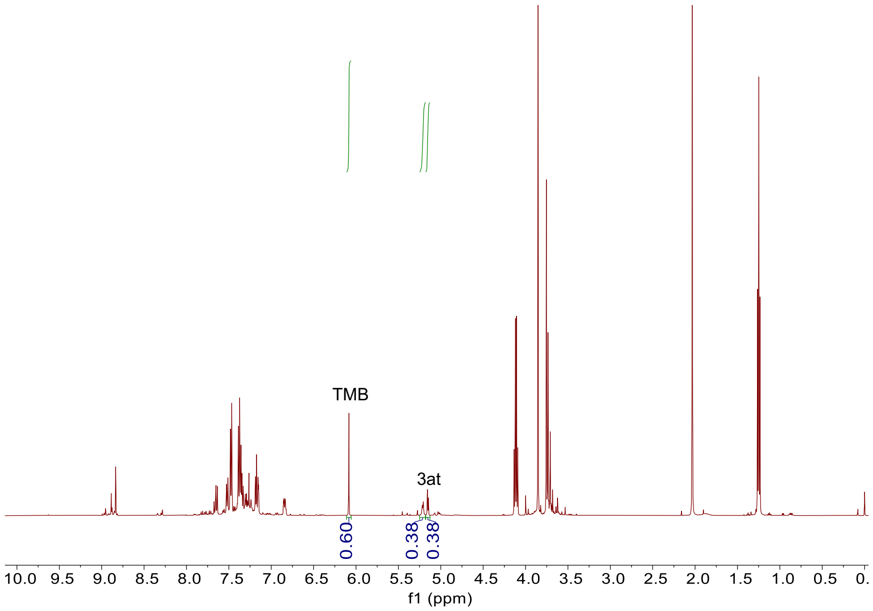

Figure S76. ^{1}H-NMR spectrum of crude reaction mixture using Rh-SA as the catalyst.

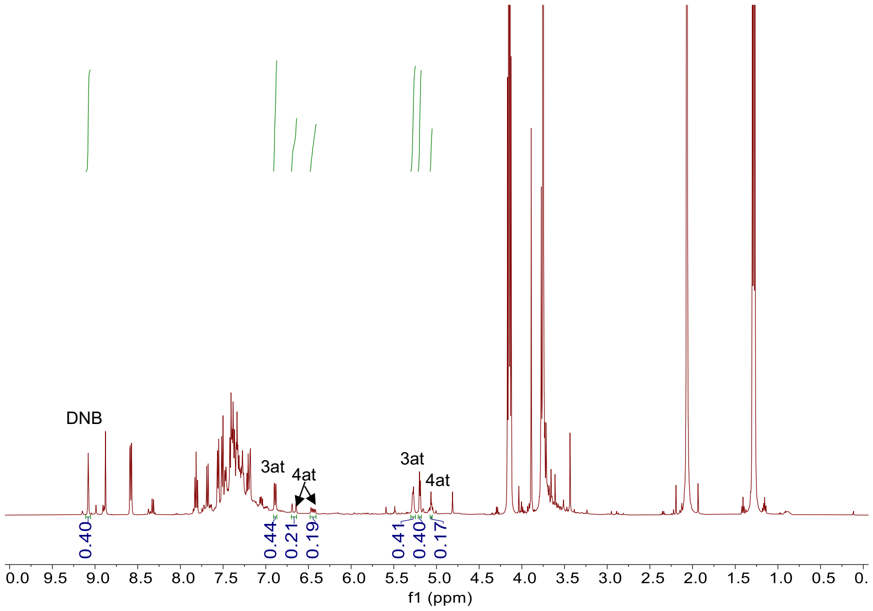

Figure S77. ^{1}H-NMR spectrum of crude reaction mixture using $Rh_2(OAc)_4$ as the catalyst.

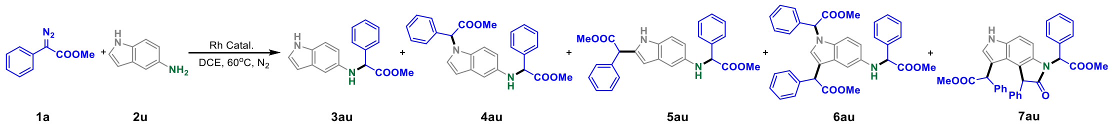

total yield; (3au: 4au: 5au: 6au:7au)

Rh-SA: 35% yield; (100: 0: 0: 0: 0)

$Rh_2(OAc)_4$: 100% yield; (25: 16: 6: 44: 9)

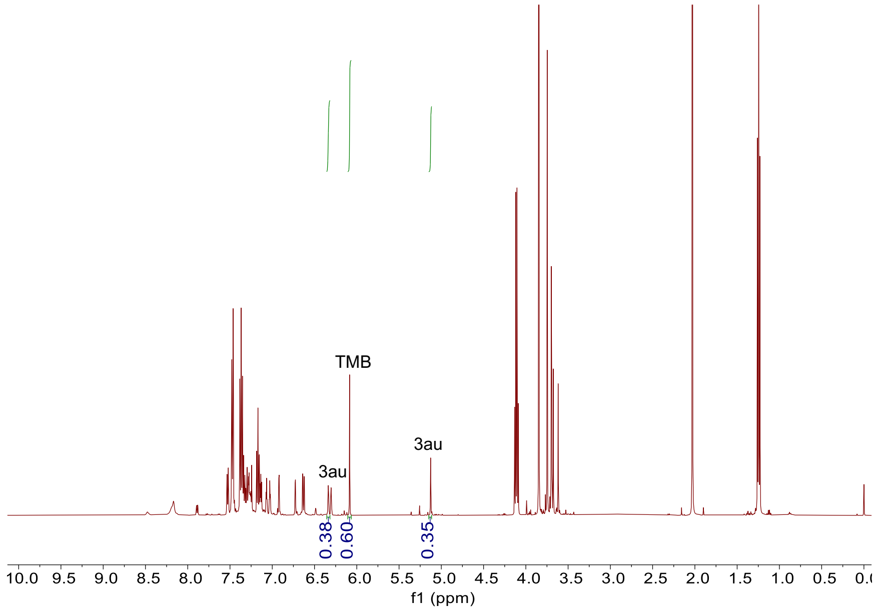

Figure S78. ^{1}H-NMR spectrum of crude reaction mixture using Rh-SA as the catalyst.

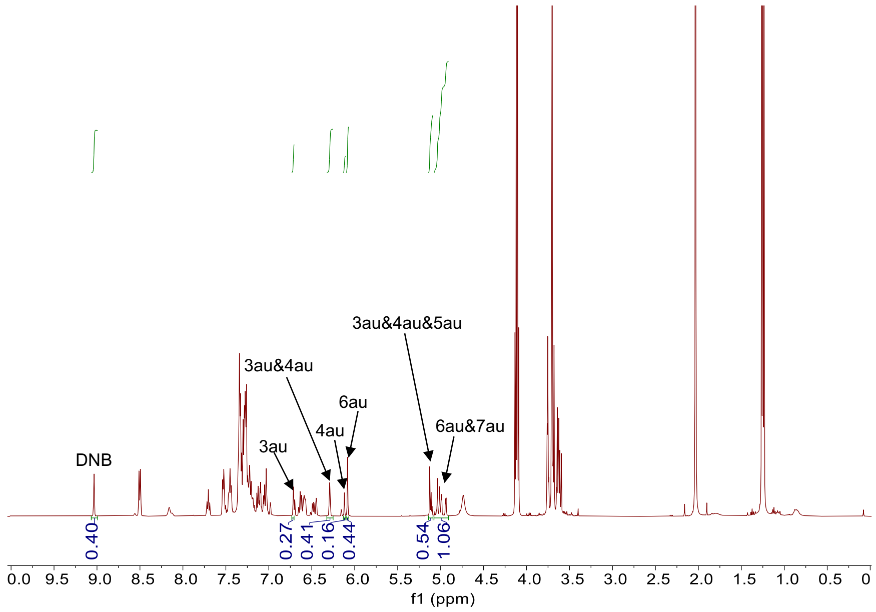

Figure S79. ^{1}H-NMR spectrum of crude reaction mixture using $Rh_2(OAc)_4$ as the catalyst.

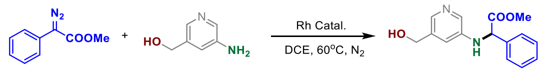

1a **2v** **3av**

Rh-SA: 24% yield

$Rh_2(OAc)_4$: 21% yield

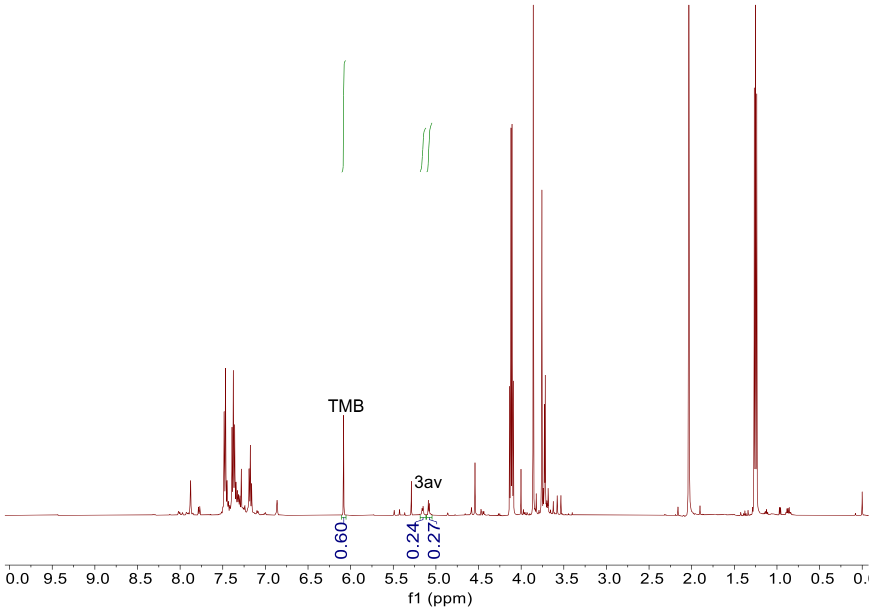

Figure S80. ^{1}H-NMR spectrum of crude reaction mixture using Rh-SA as the catalyst.

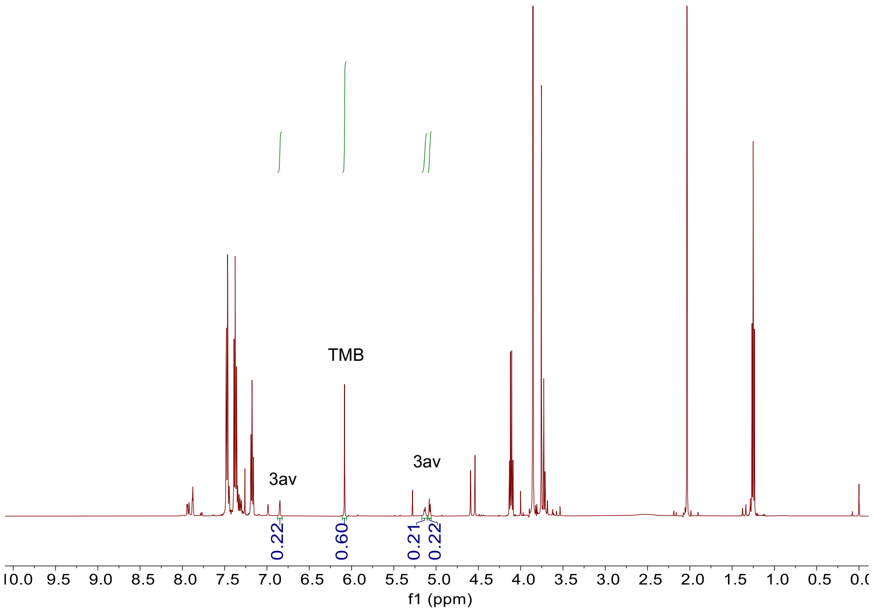

Figure S81. ^{1}H-NMR spectrum of crude reaction mixture using $Rh_2(OAc)_4$ as the catalyst.

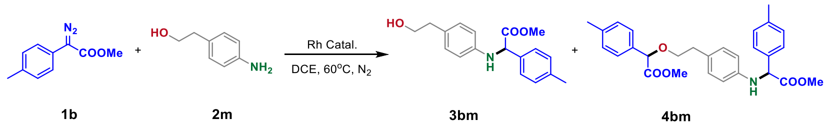

total yield; (3bm: 4bm)

Rh-SA: 83% yield; (100: 0)

$Rh_2(OAc)_4$: 74% yield; (22: 78)

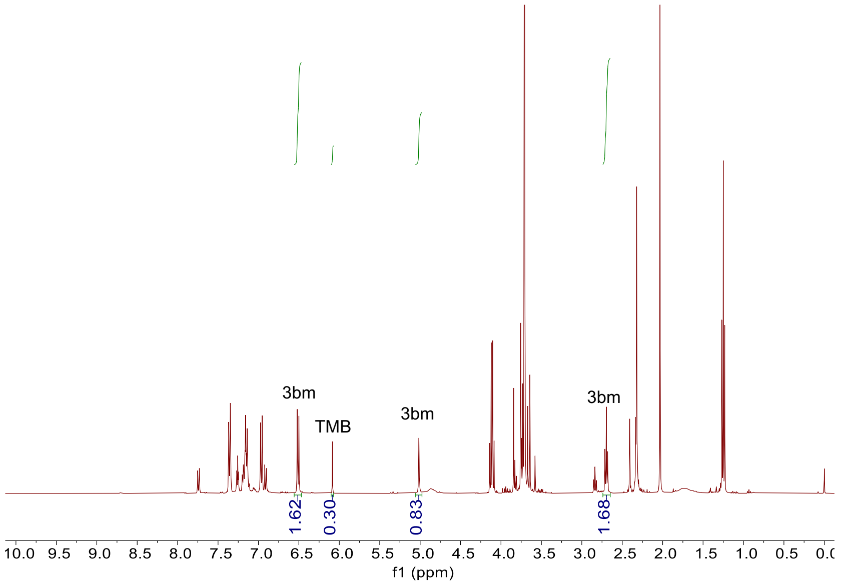

Figure S82. ^{1}H-NMR spectrum of crude reaction mixture using Rh-SA as the catalyst.

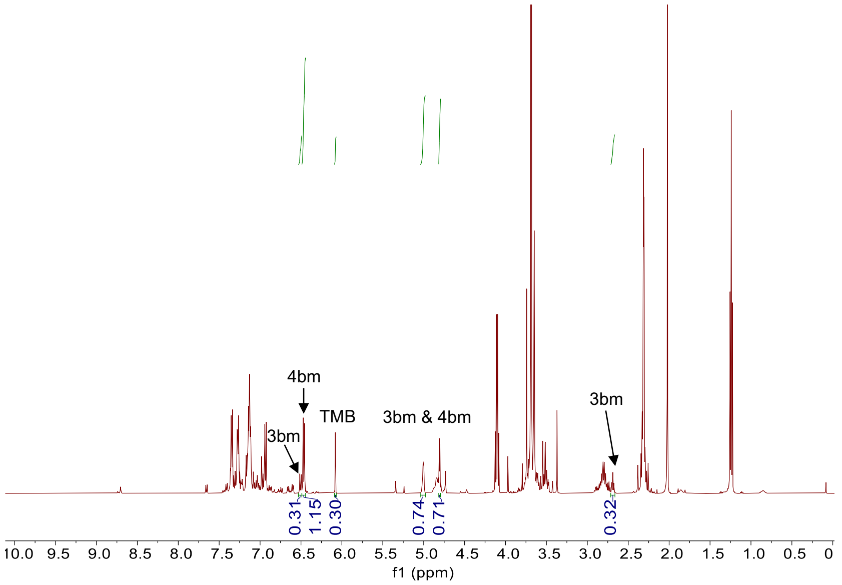

Figure S83. ^{1}H-NMR spectrum of crude reaction mixture using $Rh_2(OAc)_4$ as the catalyst.

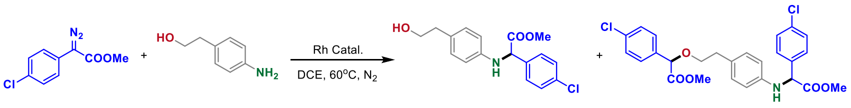

1c 2m 3cm 4cm

Total yield; (3cm: 4cm)

Rh-SA: 90% yield; (100: 0)

$Rh_2(OAc)_4$: 82% yield; (0: 100)

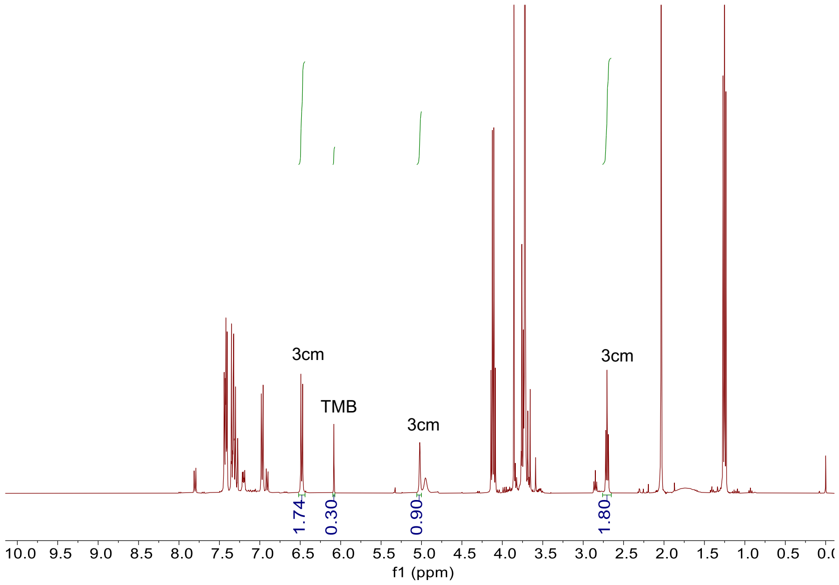

Figure S84. ^{1}H-NMR spectrum of crude reaction mixture using Rh-SA as the catalyst.

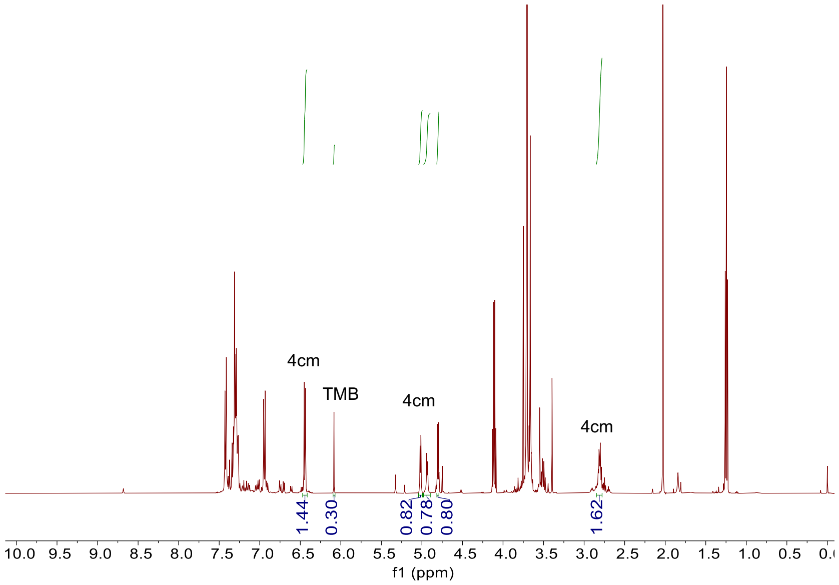

Figure S85. ^{1}H-NMR spectrum of crude reaction mixture using $Rh_2(OAc)_4$ as the catalyst.

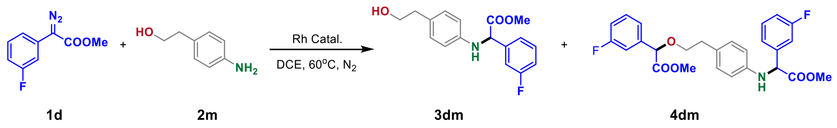

total yield; (3dm: 4dm)

Rh-SA: 92% yield; (100: 0)

$Rh_2(OAc)_4$: 81% yield; (11: 89)

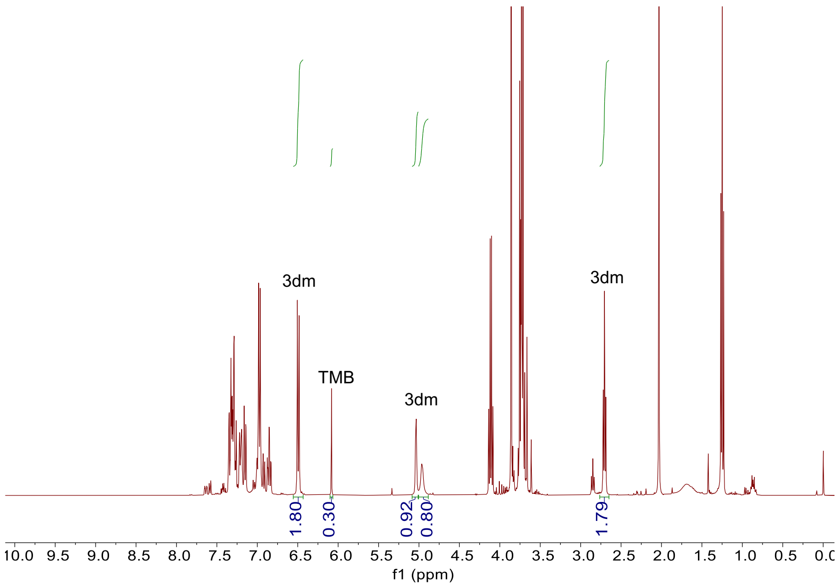

Figure S86. ^{1}H-NMR spectrum of crude reaction mixture using Rh-SA as the catalyst.

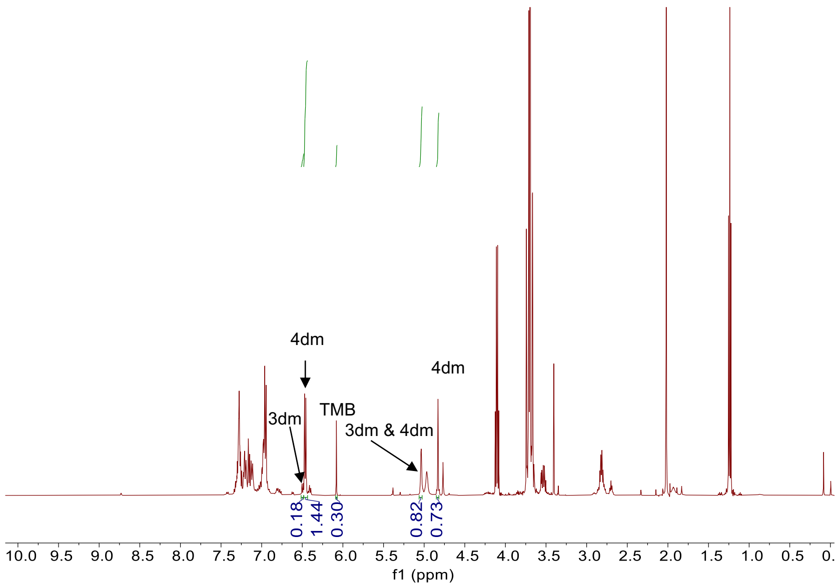

Figure S87. ^{1}H-NMR spectrum of crude reaction mixture using $Rh_2(OAc)_4$ as the catalyst.

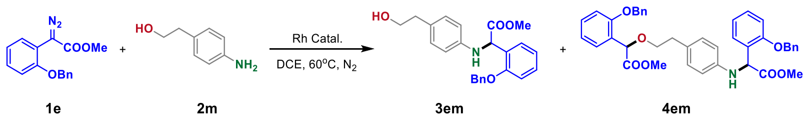

total yield; (3em: 4em)

Rh-SA: 65% yield; (100: 0)

$Rh_2(OAc)_4$: 50% yield; (0: 100)

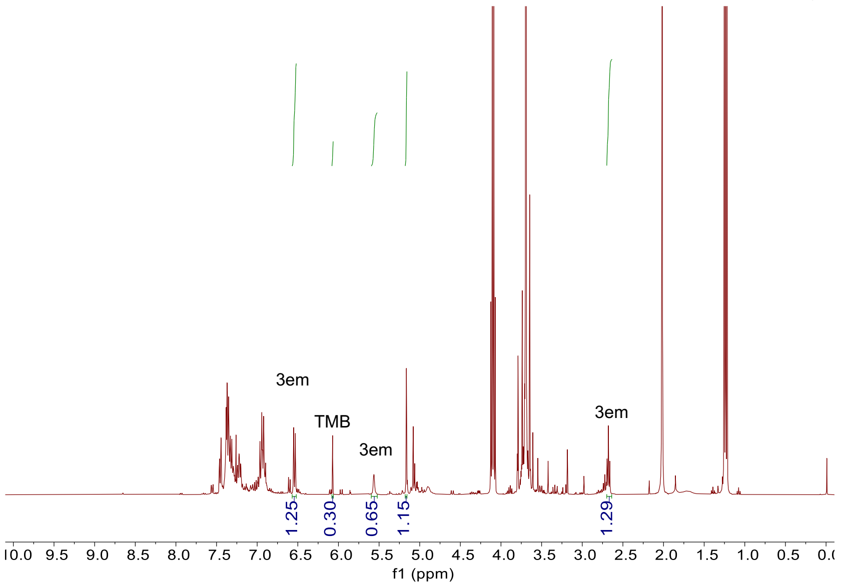

Figure S88. ^{1}H-NMR spectrum of crude reaction mixture using Rh-SA as the catalyst.

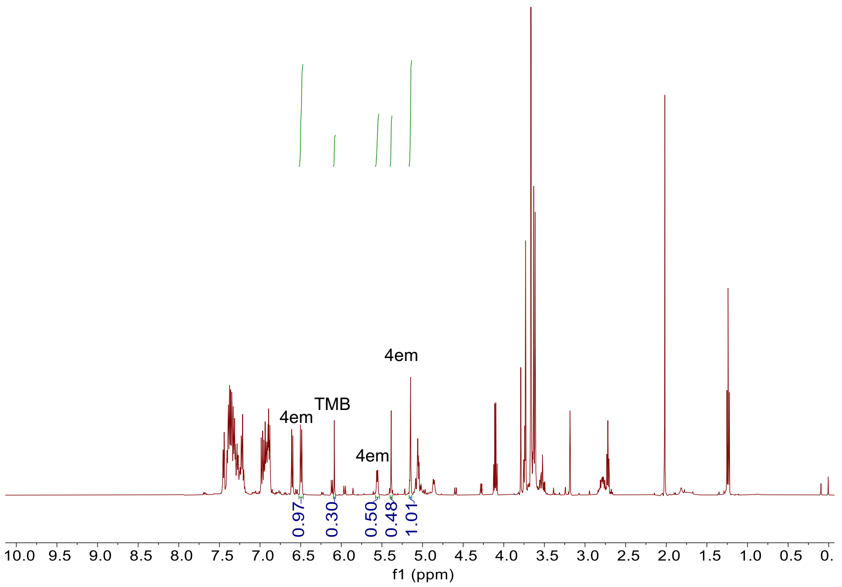

Figure S89. ^{1}H-NMR spectrum of crude reaction mixture using $Rh_2(OAc)_4$ as the catalyst.

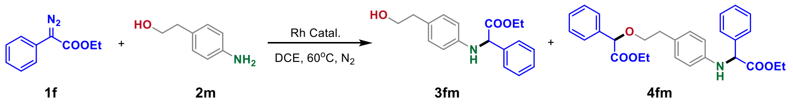

total yield; (3fm: 4fm)

Rh-SA: 88% yield; (100: 0)

$Rh_2(OAc)_4$: 60% yield; (33: 67)

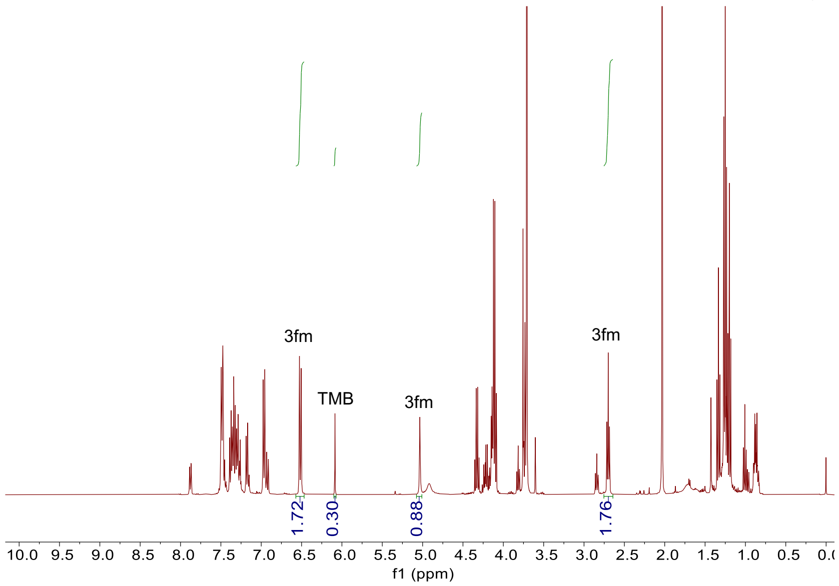

Figure S90. ^{1}H-NMR spectrum of crude reaction mixture using Rh-SA as the catalyst.

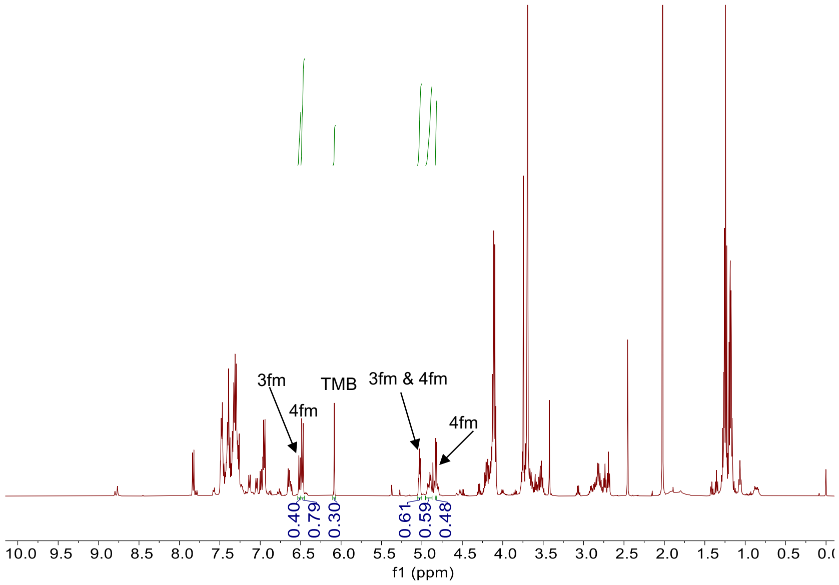

Figure S91. ^{1}H-NMR spectrum of crude reaction mixture using $Rh_2(OAc)_4$ as the catalyst.

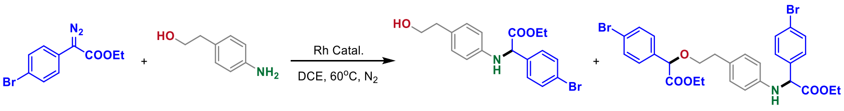

1g **2m** **3gm** **4gm**

total yield; (3gm: 4gm)

Rh-SA: 83% yield; (100: 0)

$Rh_2(OAc)_4$: 45% yield; (0: 100)

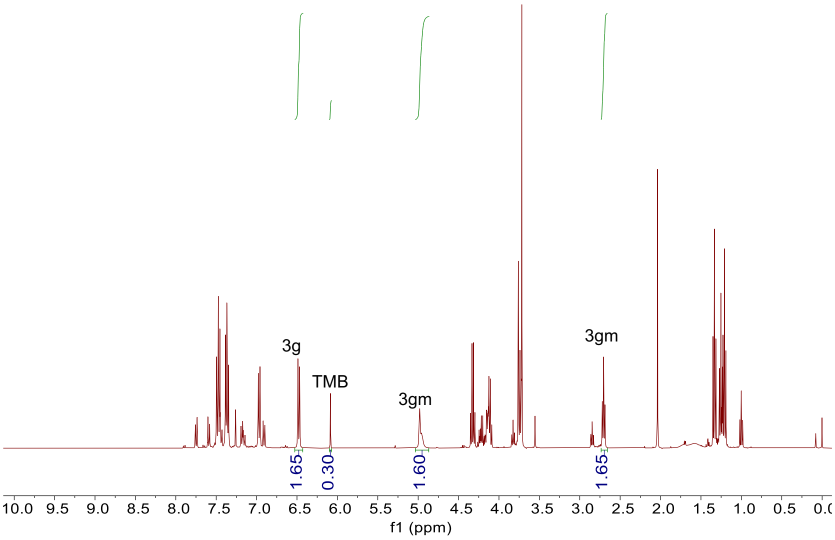

Figure S92. ^{1}H-NMR spectrum of crude reaction mixture using Rh-SA as the catalyst.

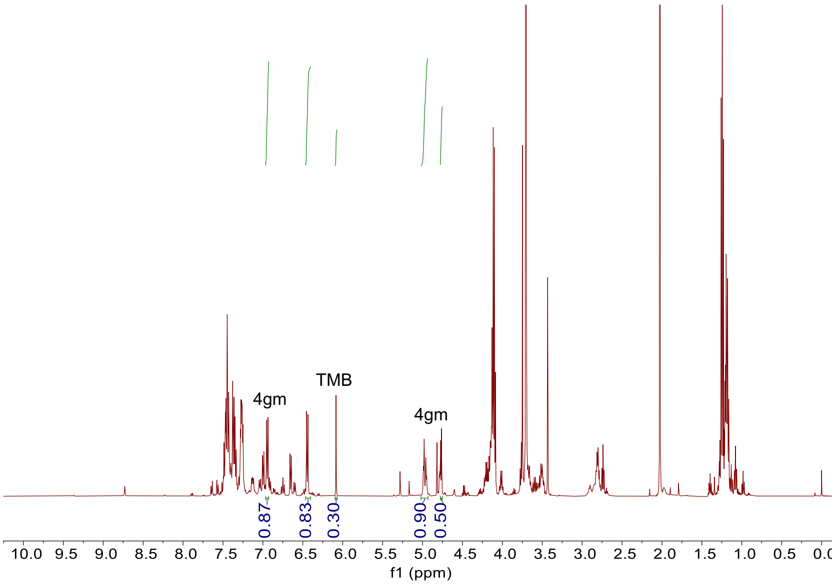

Figure S93. ^{1}H-NMR spectrum of crude reaction mixture using $Rh_2(OAc)_4$ as the catalyst.

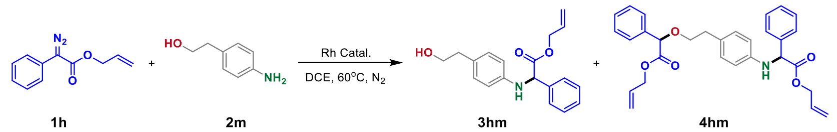

1h 2m 3hm 4hm

total yield; (3hm: 4hm)

Rh-SA: 61% yield; (100: 0)

$Rh_2(OAc)_4$: 67% yield; (18: 82)

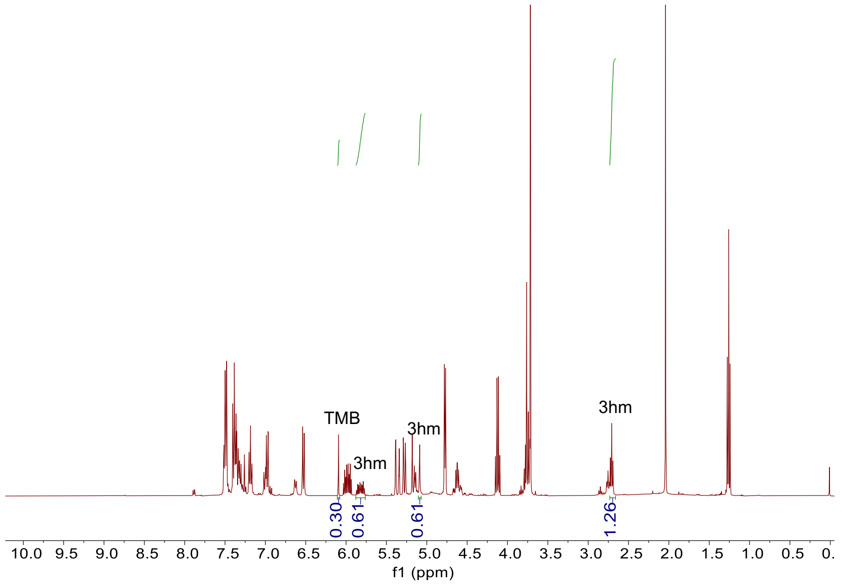

Figure S94. ^{1}H-NMR spectrum of crude reaction mixture using Rh-SA as the catalyst.

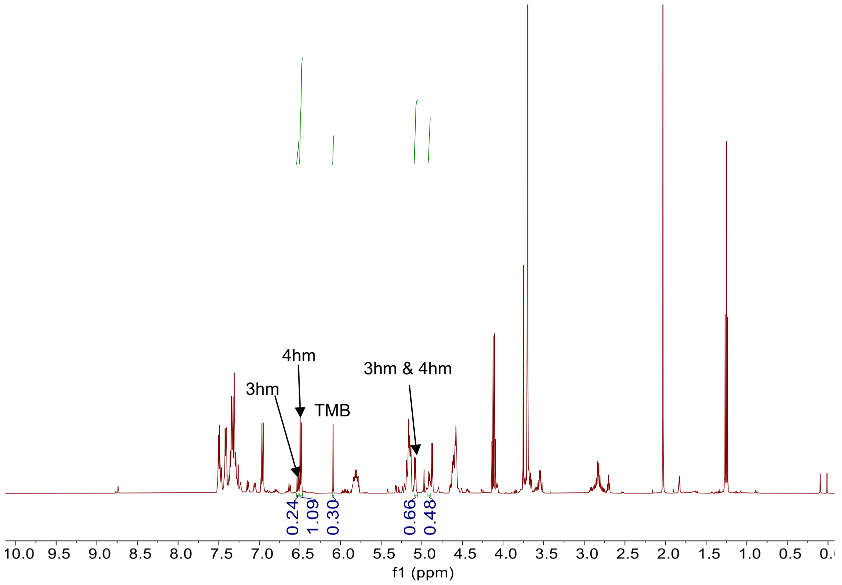

Figure S95. ^{1}H-NMR spectrum of crude reaction mixture using $Rh_2(OAc)_4$ as the catalyst.

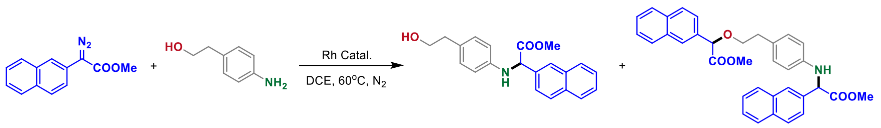

total yield; (3im: 4im)

Rh-SA: 59% yield; (100: 0)

$Rh_2(OAc)_4$: 30% yield; (0: 100)

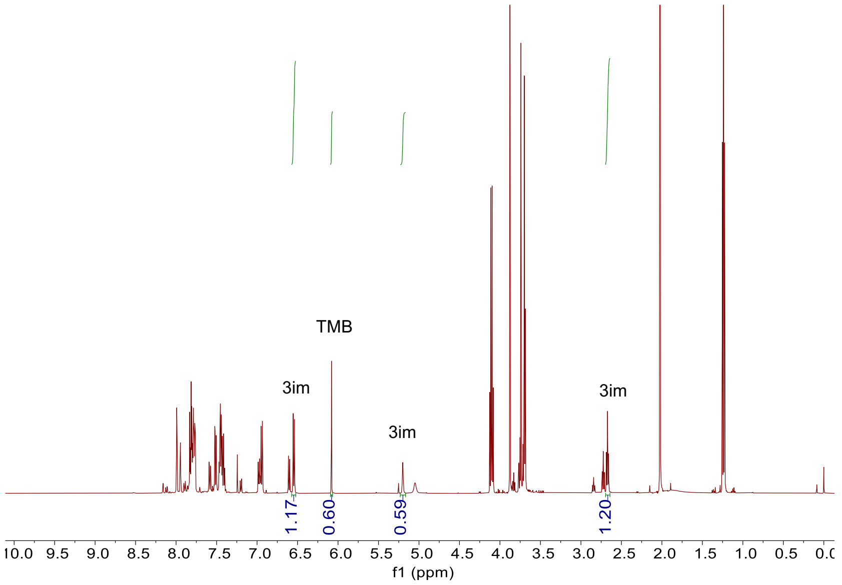

Figure S96. ^{1}H-NMR spectrum of crude reaction mixture using Rh-SA as the catalyst.

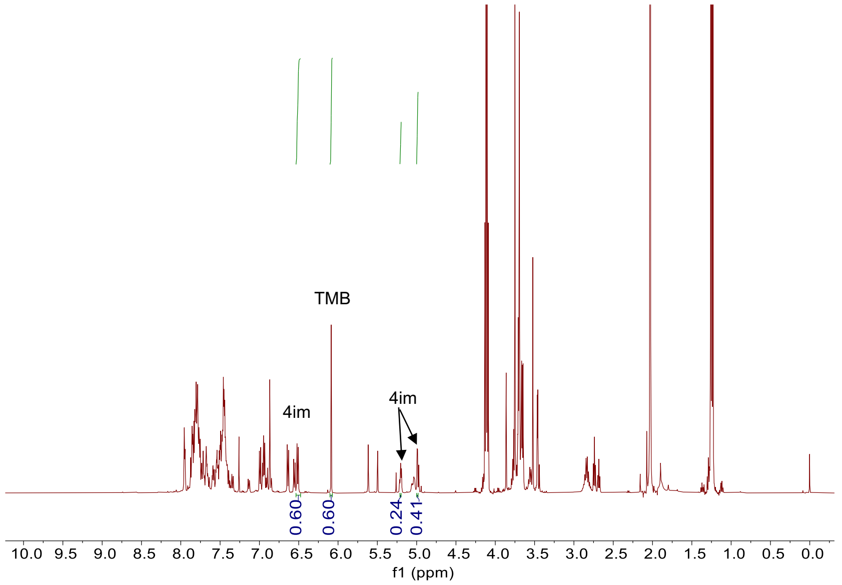

Figure S97. ^{1}H-NMR spectrum of crude reaction mixture using $Rh_2(OAc)_4$ as the catalyst.

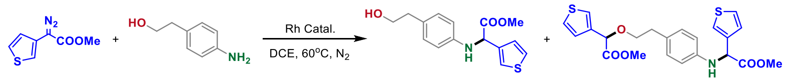

1j 2m 3jm 4jm

total yield; (3jm: 4jm)

Rh-SA: 61% yield; (100: 0)

$Rh_2(OAc)_4$: 31% yield; (26: 74)

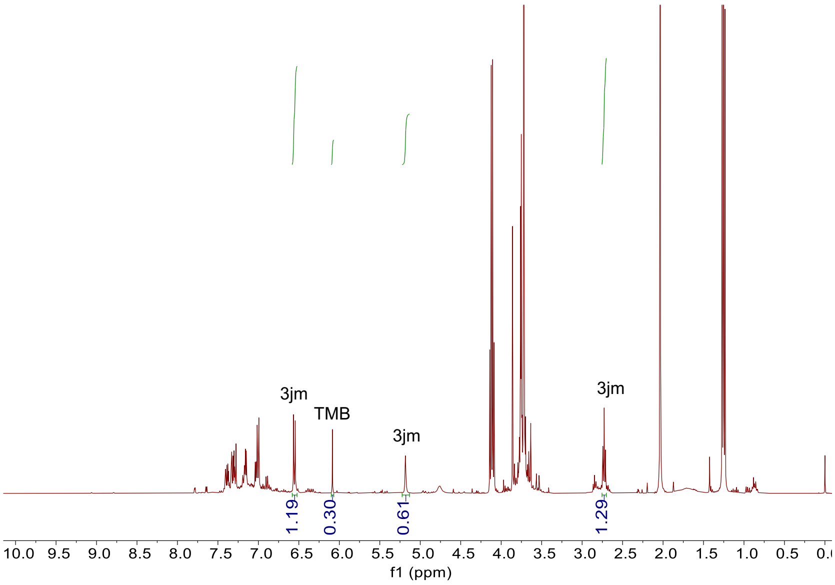

Figure S98. ^{1}H-NMR spectrum of crude reaction mixture using Rh-SA as the catalyst.

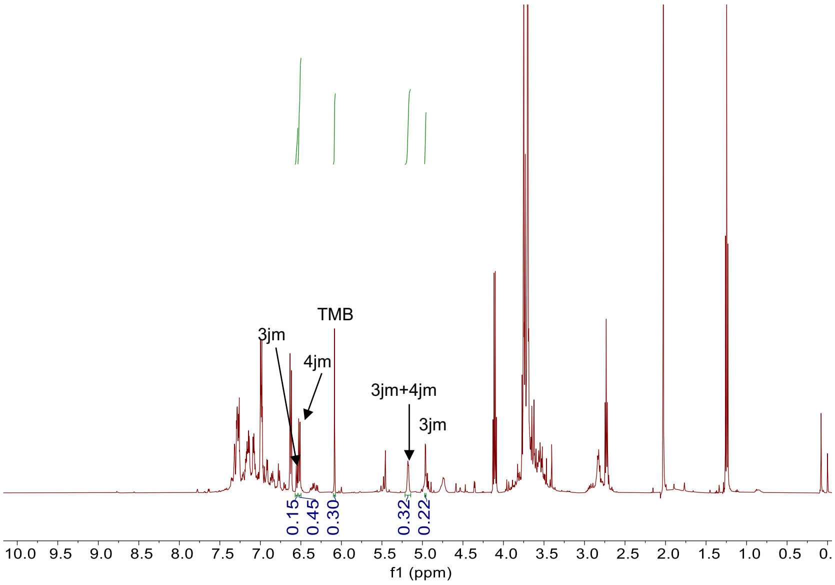

Figure S99. ^{1}H-NMR spectrum of crude reaction mixture using $Rh_2(OAc)_4$ as the catalyst.

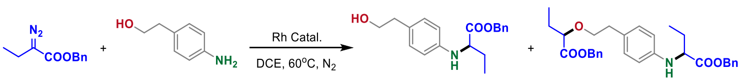

1k 2m 3km 4km

total yield; (3km: 4km)

Rh-SA: 56% yield; (100: 0)

$Rh_2(OAc)_4$: 45% yield; (44: 56)

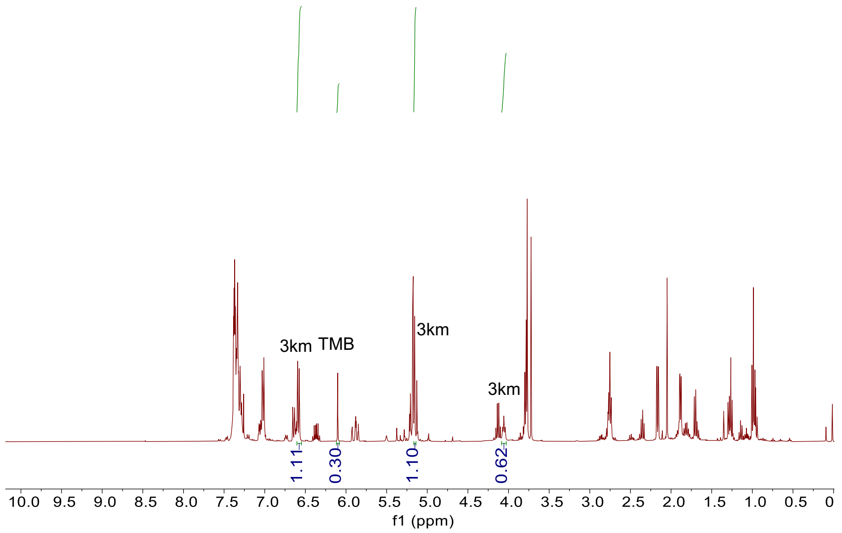

Figure S100. ^{1}H-NMR spectrum of crude reaction mixture using Rh-SA as the catalyst.

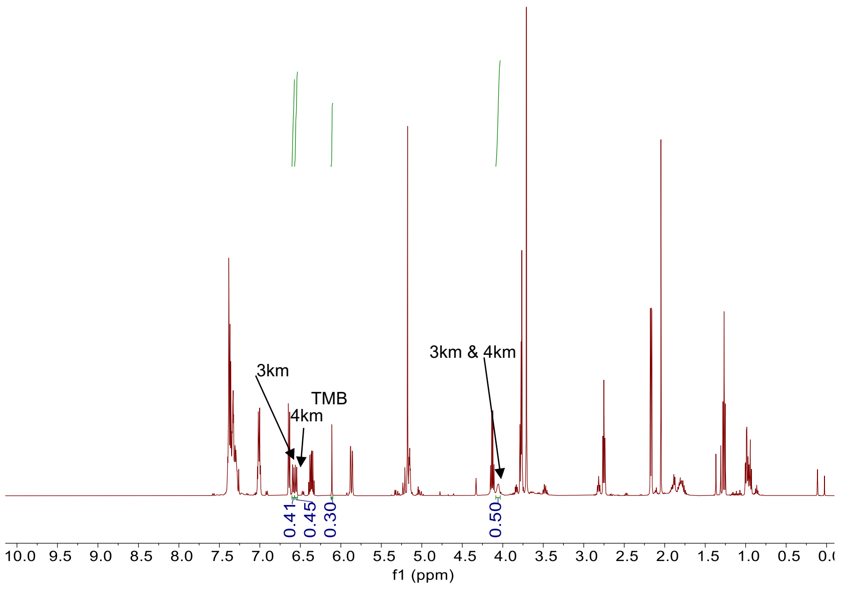

Figure S101. ^{1}H-NMR spectrum of crude reaction mixture using $Rh_2(OAc)_4$ as the catalyst.

Supplementary data for products

Methyl (R)-2-phenyl-2-(phenylamino)acetate (3aa)[1]

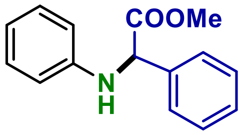

^{1}H-NMR (500 MHz, $CDCl_3$) δ p.p.m. 7.51 (dd, J = 7.3, 1.8 Hz, 2H), 7.41 – 7.30 (m, 3H), 7.18 – 7.09 (m, 2H), 6.72 (t, J = 7.3 Hz, 1H), 6.57 (d, J = 8.0 Hz, 2H), 5.10 (d, J = 4.7 Hz, 1H), 4.98 (d, J = 5.1 Hz, 1H), 3.74 (s, 3H); **^{13}C-NMR** (126 MHz, $CDCl_3$) δ 172.46, 146.03, 137.71, 129.36, 129.00, 128.44, 127.37, 118.22, 113.49, 60.82, 52.95; **HRMS (ESI)** calcd. for $[M+H]^+$: 242.1176 m/z, found: 242.1167 m/z.

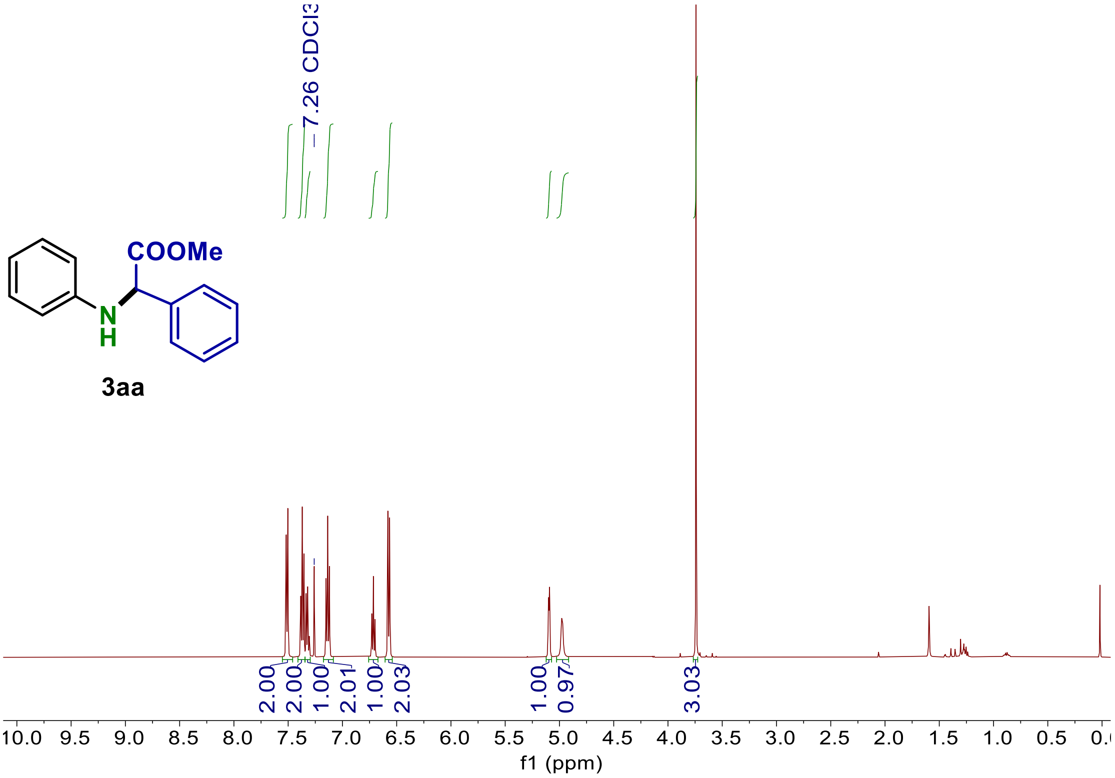

Figure S102. ^{1}H-NMR spectra of 3aa.

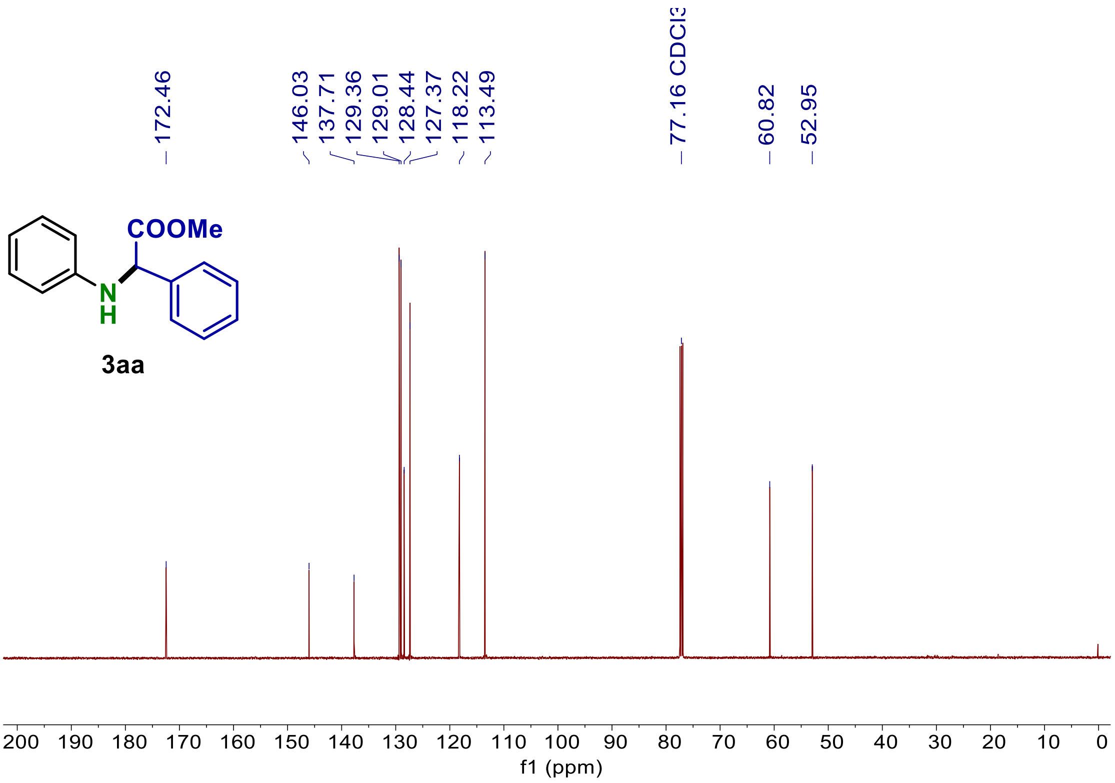

Figure S103. ^{13}C-NMR spectra of 3aa.

Methyl (R)-2-phenyl-2-(o-tolylamino)acetate (3ab)[2]

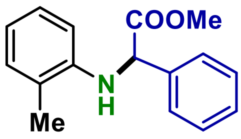

^{1}**H-NMR** (500 MHz, $CDCl_3$) δ p.p.m. 7.51 (dd, *J* = 7.0, 1.6 Hz, 2H), 7.39 – 7.33 (m, 2H), 7.33 – 7.28 (m, 1H), 7.07 (d, *J* = 7.4 Hz, 1H), 7.01 – 6.94 (m, 1H), 6.68 – 6.61 (m, 1H), 6.33 (d, *J* = 8.0 Hz, 1H), 5.12 (s, 1H), 4.89 (s, 1H), 3.74 (s, 3H), 2.28 (s, 3H); ^{13}**C-NMR** (101 MHz, $CDCl_3$) δ 172.66, 144.08, 137.82, 130.35, 129.02, 128.43, 127.35, 127.13, 122.60, 117.83, 110.74, 60.80, 53.02, 17.69; **HRMS (ESI)** calcd. for $[M+H]^+$: 256.1332 m/z, found: 256.1332 m/z.

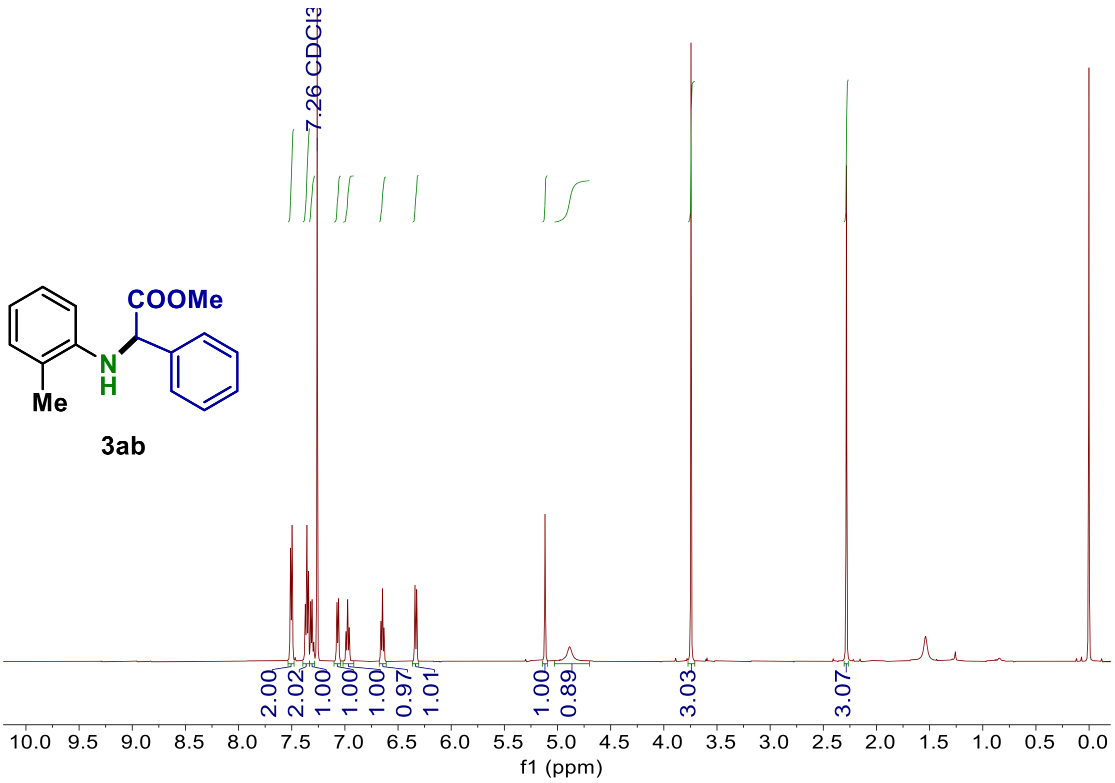

Figure S104. ¹H-NMR spectra of 3ab.

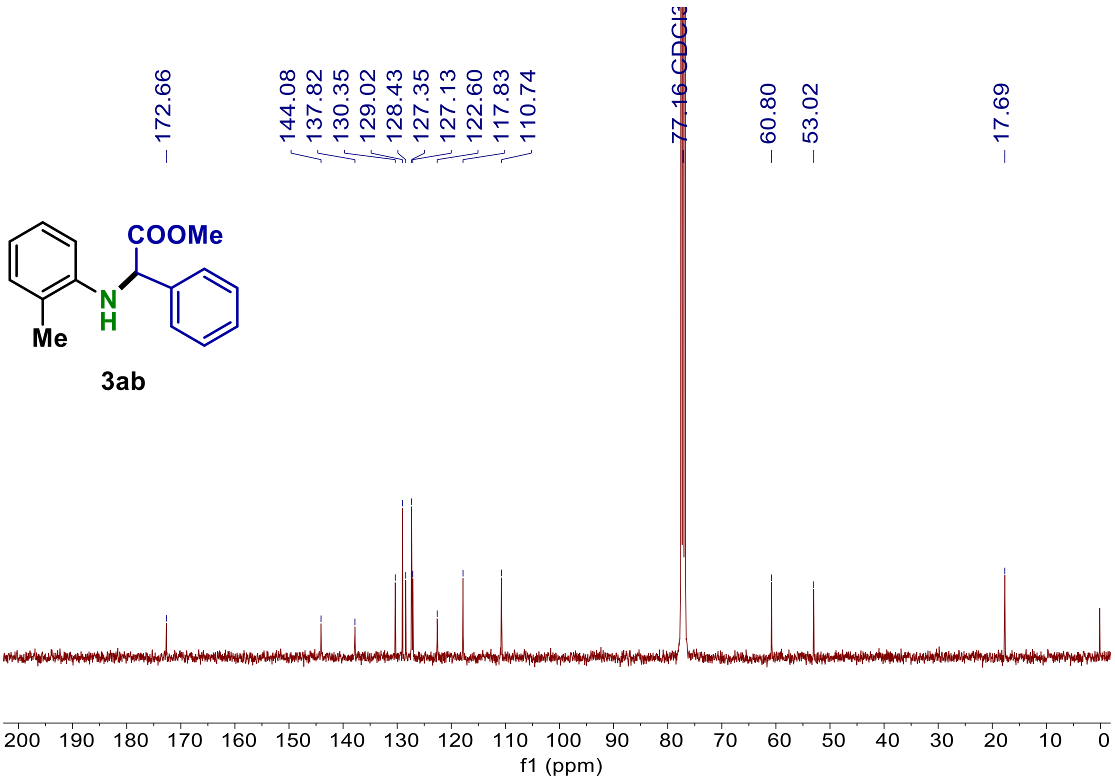

Figure S105. ¹³C-NMR spectra of 3ab.

Methyl (R)-2-phenyl-2-(m-tolylamino)acetate (3ac) [2]

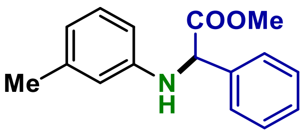

¹H-NMR (500 MHz, $CDCl_3$) δ p.p.m. 7.52 (dd, *J* = 7.7, 1.4 Hz, 2H), 7.41 – 7.29 (m, 3H), 7.03 (t, *J* = 7.7 Hz, 1H), 6.55 (d, *J* = 7.4 Hz, 1H), 6.44 (t, *J* = 2.0 Hz, 1H), 6.37 (dd, *J* = 8.0, 2.4 Hz, 1H), 5.10 (s, 1H), 4.91 (s, 1H), 3.74 (s, 3H), 2.25 (s, 3H); **¹³C-NMR** (101 MHz, $CDCl_3$) δ 172.53, 146.09, 139.13, 137.81, 129.23, 128.98, 128.39, 127.34, 119.21, 114.43, 110.44, 60.82, 52.90, 21.69; **HRMS (ESI)** calcd. for $[M+H]^+$: 256.1332 m/z, found: 256.1327 m/z.

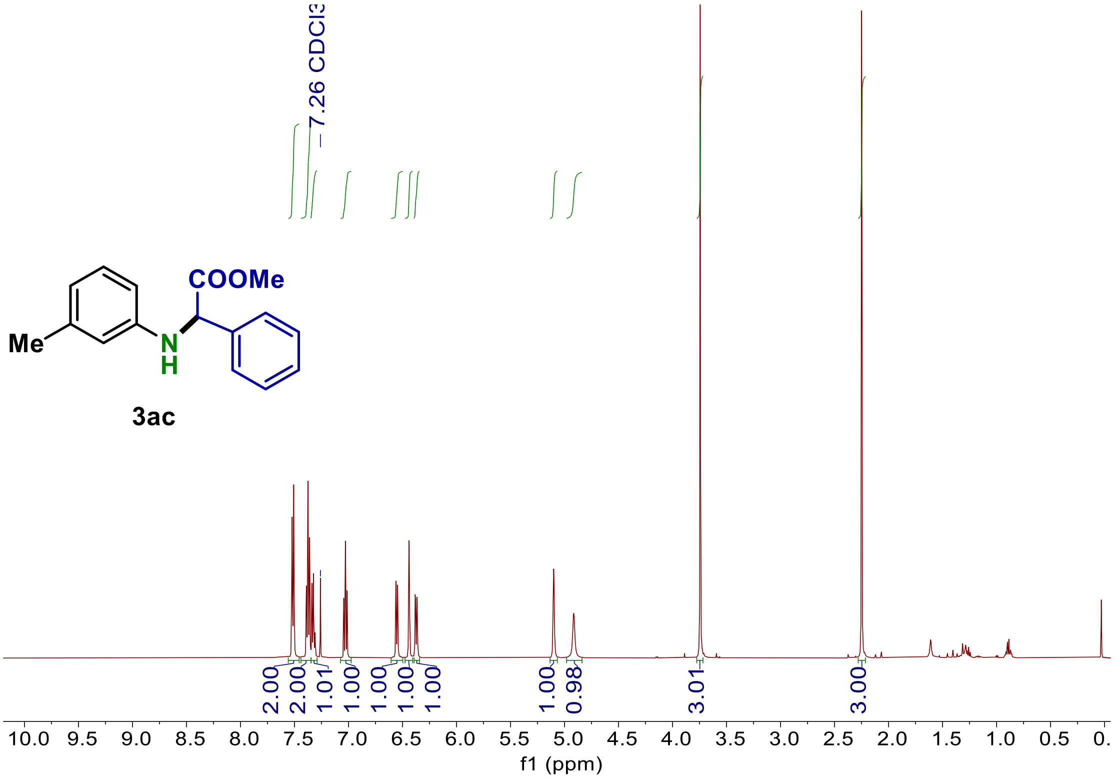

Figure S106. ¹H-NMR spectra of 3ac.

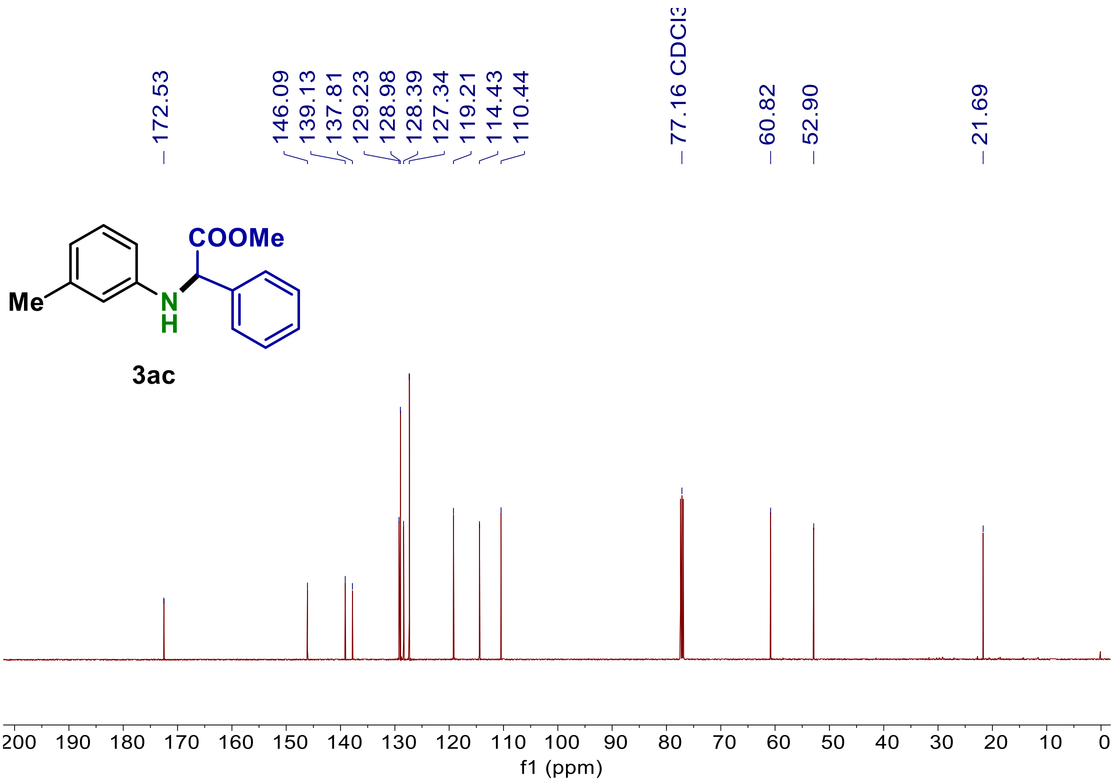

Figure S107. ^{13}C-NMR spectra of 3ac.

Methyl (R)-2-((4-methoxyphenyl)amino)-2-phenylacetate (3ad)[2]

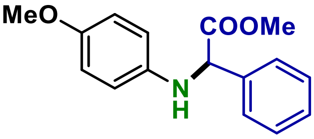

^{1}H-NMR (500 MHz, $CDCl_3$) δ p.p.m. 7.52 – 7.47 (m, 2H), 7.40 – 7.34 (m, 2H), 7.34 – 7.28 (m, 1H), 6.73 (d, J = 8.8 Hz, 2H), 6.54 (d, J = 8.8 Hz, 2H), 5.03 (s, 1H), 4.67 (s, 1H), 3.73 (s, 3H), 3.71 (s, 3H); **^{13}C-NMR** (126 MHz, $CDCl_3$) δ 172.67, 152.64, 140.31, 137.91, 128.97, 128.39, 127.40, 114.97, 114.88, 61.77, 55.80, 52.83; **HRMS (ESI)** calcd. for $[M+H]^+$: 272.1281 m/z, found: 272.1279 m/z.

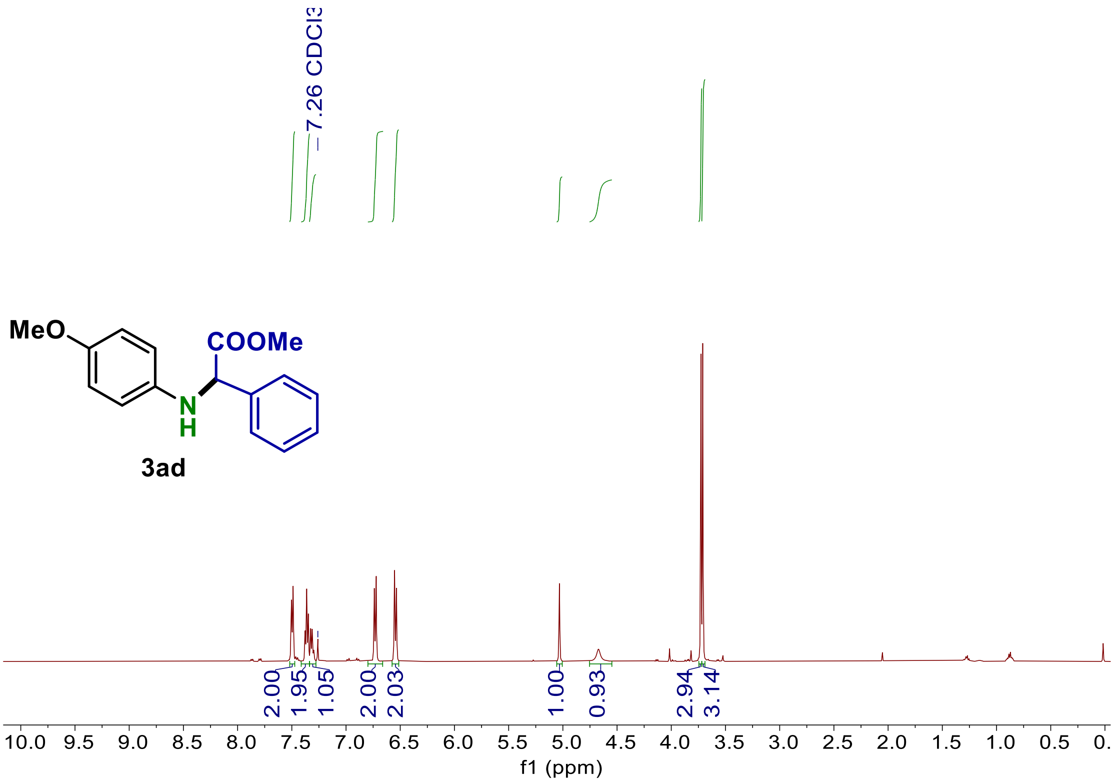

Figure S108. ^{1}H-NMR spectra of 3ad.

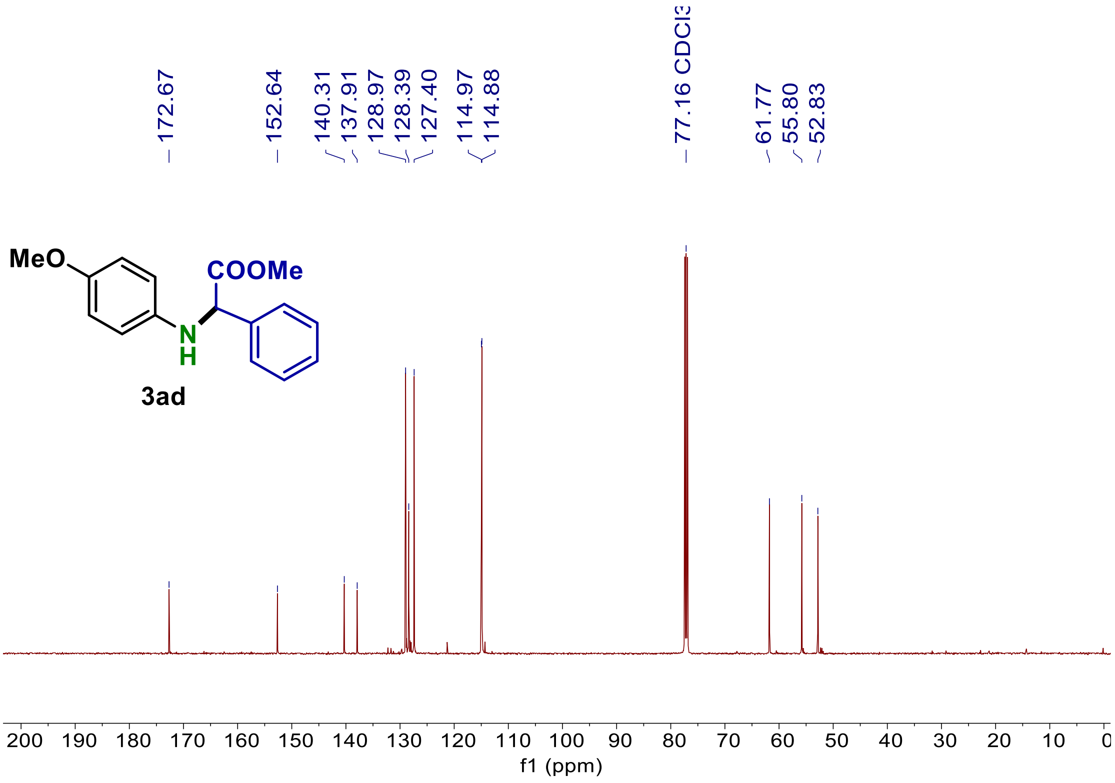

Figure S109. ^{13}C-NMR spectra of 3ad.

Methyl (R)-2-((4-(tert-butyl)phenyl)amino)-2-phenylacetate (3ae) [2]

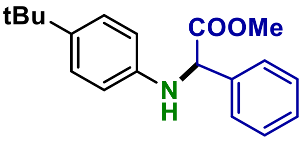

^{1}H-NMR (500 MHz, $CDCl_3$) δ p.p.m. 7.55 – 7.47 (m, 2H), 7.37 (dd, *J* = 8.2, 6.4 Hz, 2H), 7.34 – 7.29 (m, 1H), 7.19 – 7.13 (m, 2H), 6.56 – 6.50 (m, 2H), 5.06 (s, 1H), 4.87 (s, 1H), 3.74 (s, 3H), 1.25 (s, 9H); **^{13}C-NMR** (126 MHz, $CDCl_3$) δ 172.61, 143.77, 140.94, 138.04, 128.98, 128.38, 127.42, 126.16, 113.14, 61.15, 52.92, 33.97, 31.61; **HRMS (ESI)** calcd. for $[M+H]^+$: 298.1802 m/z, found: 298.1793 m/z.

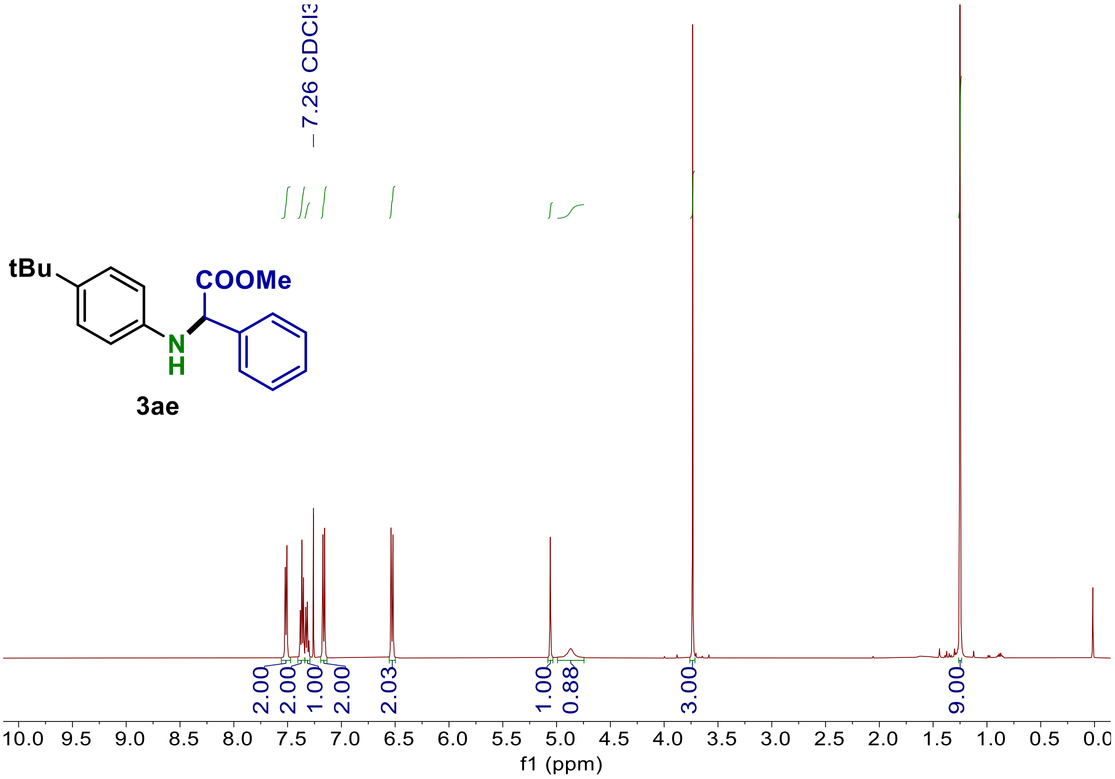

Figure S110. ^{1}H-NMR spectra of 3ae.

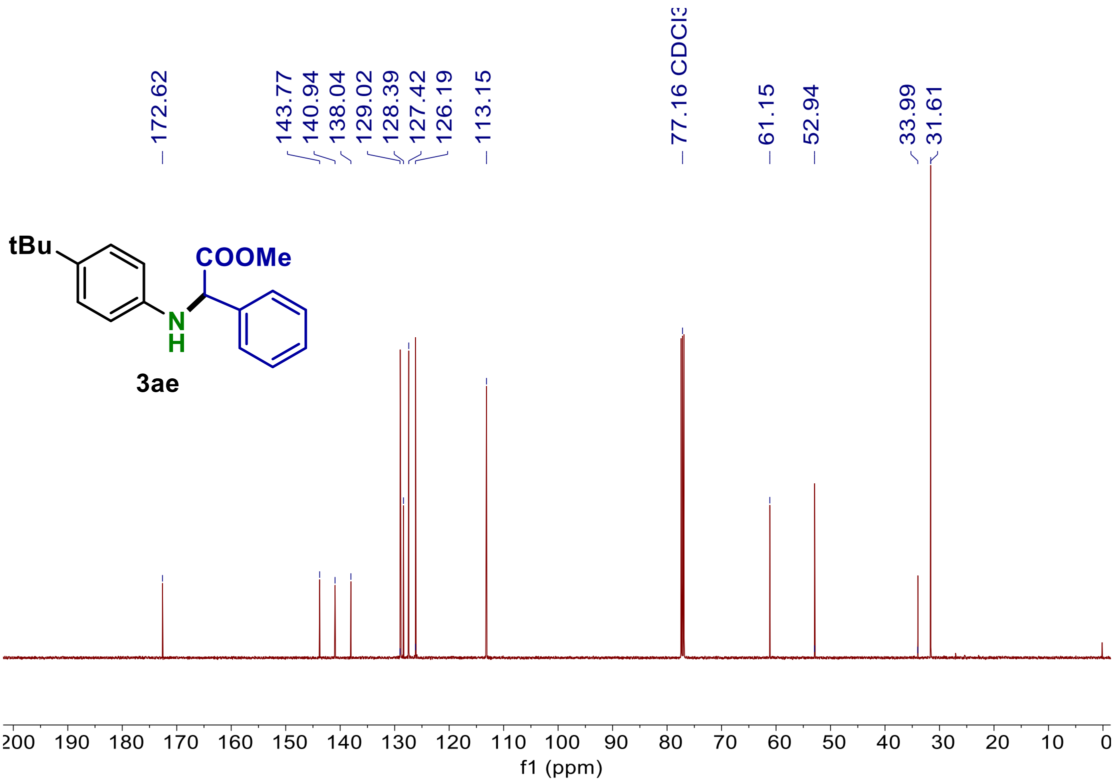

Figure S111. ^{13}C-NMR spectra of 3ae.

Methyl (R)-2-((4-fluorophenyl)amino)-2-phenylacetate (3af) [2]

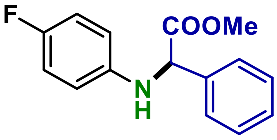

^{1}H-NMR (400 MHz, $CDCl_3$) δ p.p.m. 7.47 (d, *J* = 7.0 Hz, 2H), 7.40 – 7.29 (m, 3H), 6.83 (t, *J* = 8.7 Hz, 2H), 6.54 – 6.44 (m, 2H), 5.01 (s, 1H), 3.73 (s, 3H); **^{13}C-NMR** (101 MHz, $CDCl_3$) δ 172.40, 157.41, 155.07, 142.37, 137.50, 129.07, 128.56, 127.37, 115.97, 115.75, 114.44, 114.37, 61.38, 53.02; **HRMS (ESI)** calcd. for $[M+H]^+$: 260.1081 m/z, found: 260.1081 m/z.

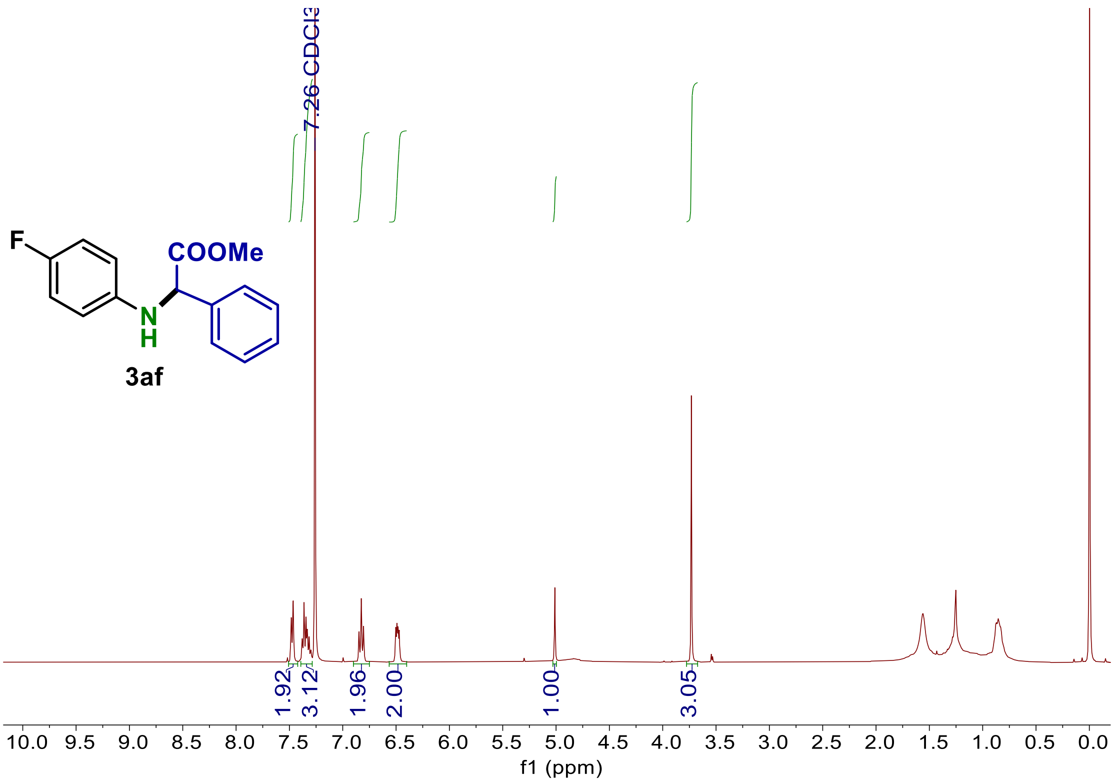

Figure S112. ^{1}H-NMR spectra of 3af.

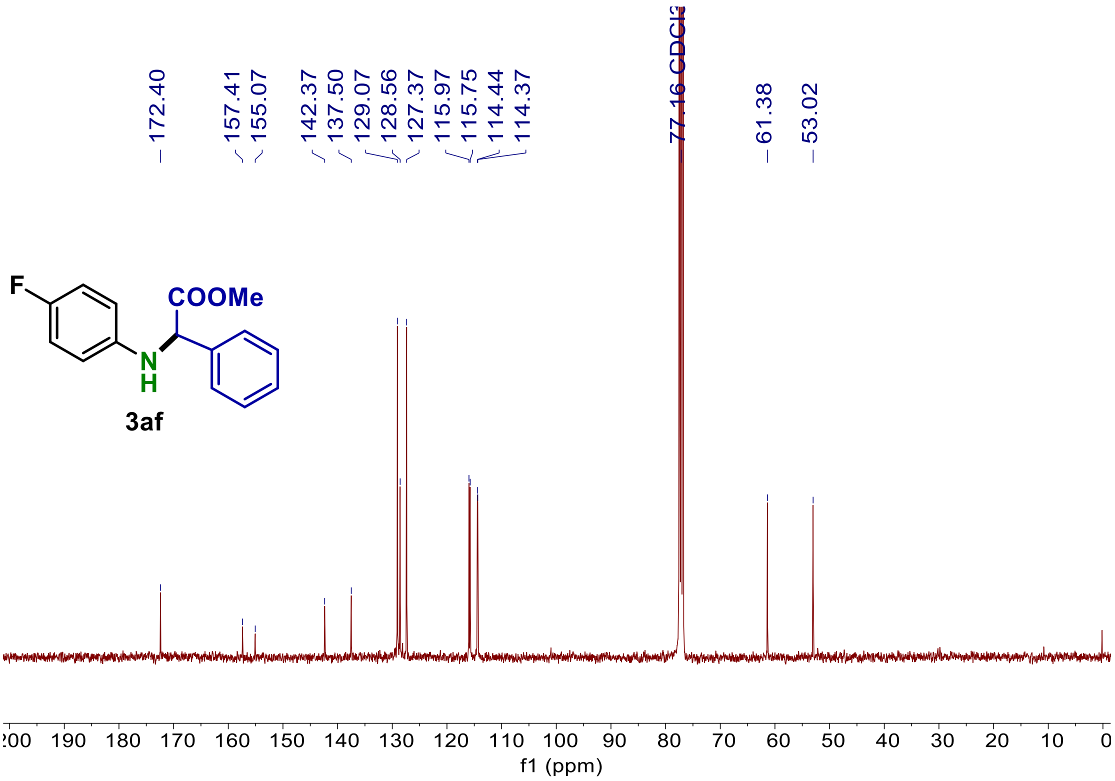

Figure S113. ^{13}C-NMR spectra of 3af.

Methyl (R)-2-phenyl-2-((4-(trifluoromethyl)phenyl)amino)acetate (3ag)[3]

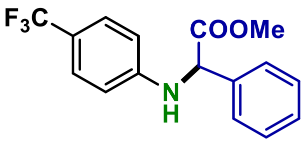

^{1}H-NMR (500 MHz, $CDCl_3$) δ p.p.m. 7.50 – 7.44 (m, 2H), 7.40 – 7.30 (m, 5H), 6.55 (d, *J* = 8.5 Hz, 2H), 5.34 (d, *J* = 5.5 Hz, 1H), 5.09 (d, *J* = 5.4 Hz, 1H), 3.75 (s, 3H); **^{13}C-NMR** (126 MHz, $CDCl_3$) δ 171.88, 148.37, 136.89, 129.19, 128.75, 127.29, 126.77, 126.73, 112.77, 60.29, 53.19; **HRMS (ESI)** calcd. for $[M+H]^+$: 310.1049 m/z, found: 310.1049 m/z.

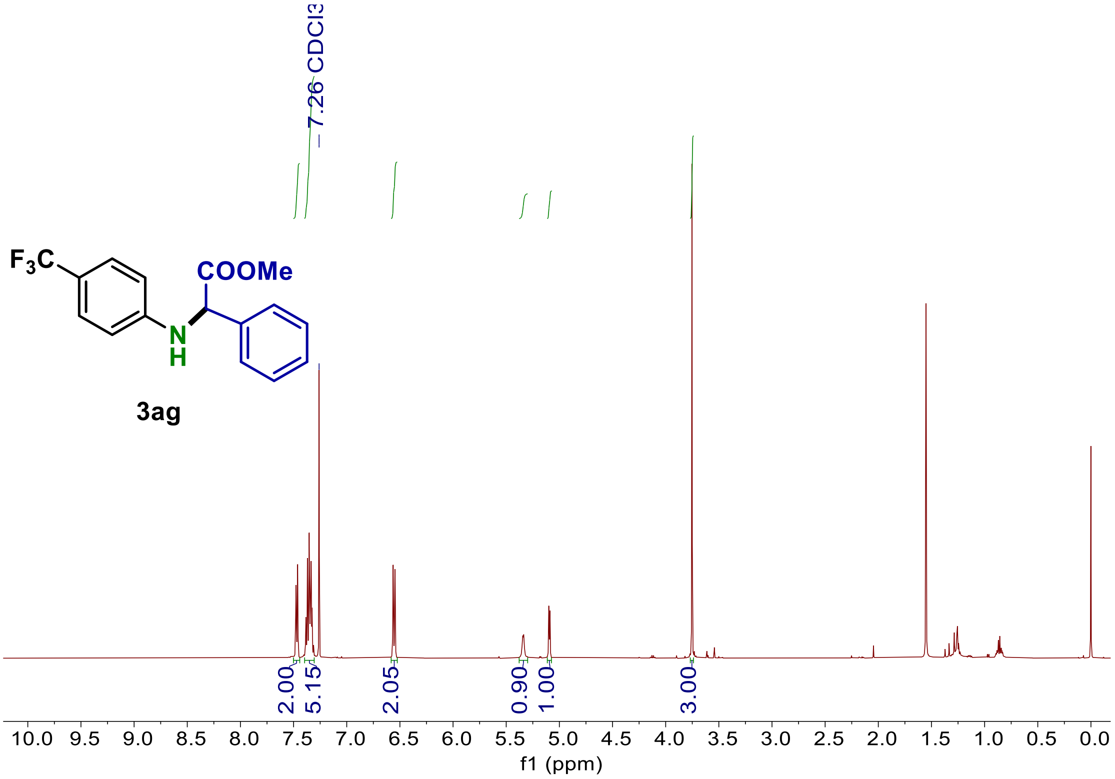

Figure S114. ^{1}H-NMR spectra of 3ag.

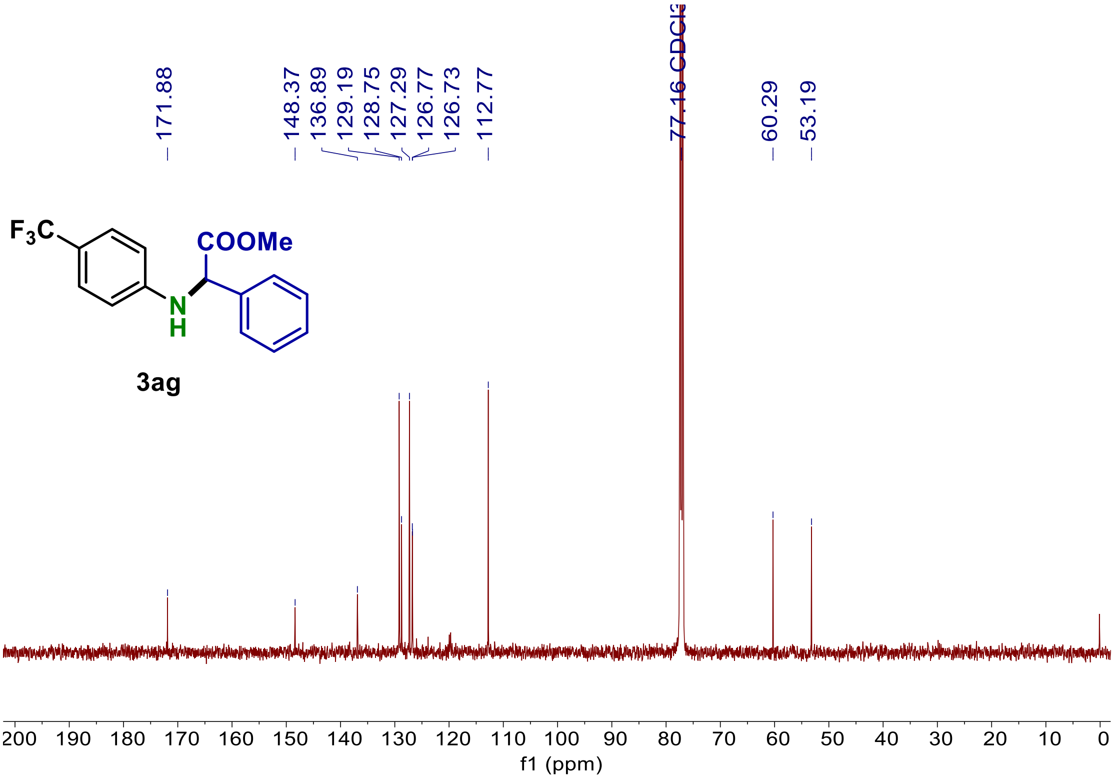

Figure S115. ^{13}C-NMR spectra of 3ag.

Methyl (R)-2-((2,6-diisopropylphenyl)amino)-2-phenylacetate (3ah)[4]

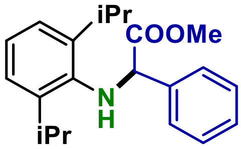

^{1}H-NMR (500 MHz, $CDCl_3$) δ p.p.m. 7.43 – 7.28 (m, 5H), 7.11 – 7.01 (m, 3H), 4.70 (s, 1H), 4.34 (s, 1H), 3.71 (s, 3H), 3.19 (hept, J = 6.8 Hz, 2H), 1.20 (d, J = 6.8 Hz, 6H), 1.11 (d, J = 6.8 Hz, 6H); **^{13}C-NMR** (126 MHz, $CDCl_3$): δ 173.87, 142.04, 141.34, 138.61, 128.78, 128.21, 127.08, 123.76, 66.95, 52.50, 27.79, 24.43, 24.17; **HRMS (ESI)** calcd. for $[M+H]^+$: 326.2115 m/z, found: 326.2105 m/z.

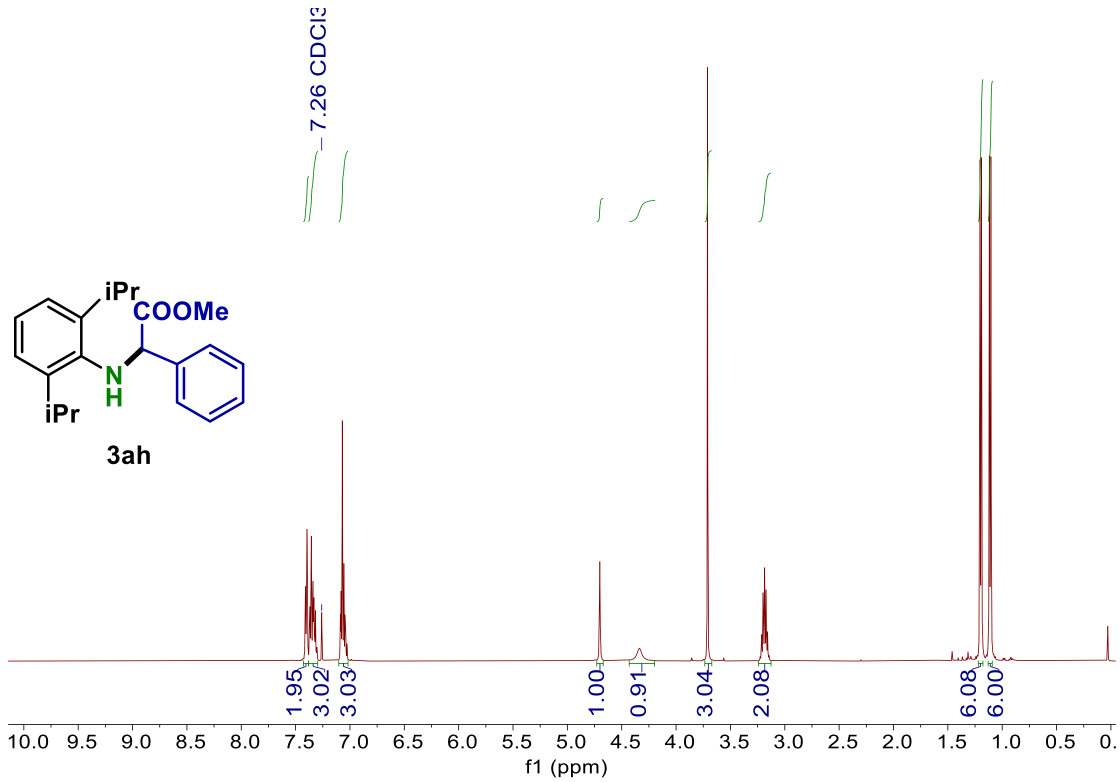

Figure S116. ^{1}H-NMR spectra of 3ah.

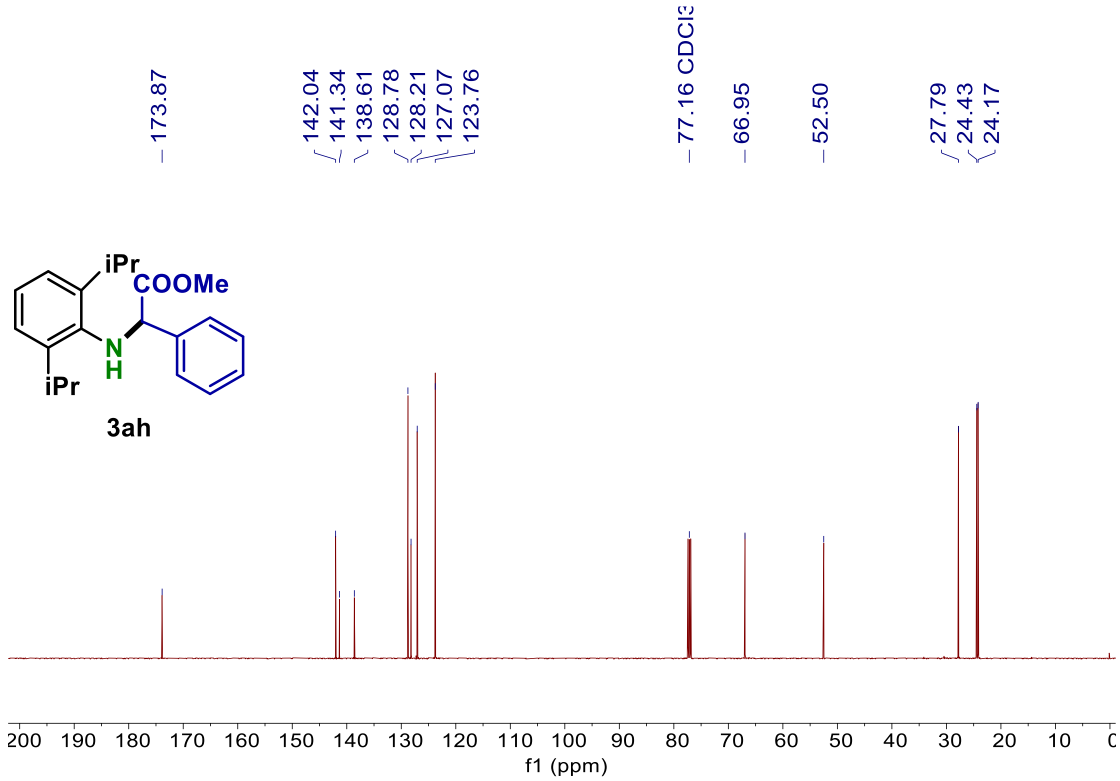

Figure S117. ^{13}C-NMR spectra of 3ah.

Methyl (R)-2-phenyl-2-(pyridin-2-ylamino)acetate (3ai)[5]

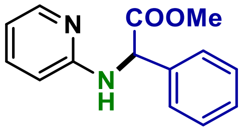

¹H NMR (500 MHz, $CDCl_3$) δ p.p.m. 8.09 (ddd, *J* = 5.2, 1.9, 0.9 Hz, 1H), 7.51 – 7.46 (m, 2H), 7.40 – 7.30 (m, 4H), 6.60 (ddd, *J* = 7.2, 5.1, 1.0 Hz, 1H), 6.41 (dt, *J* = 8.4, 1.0 Hz, 1H), 5.57 (d, *J* = 6.8 Hz, 1H), 5.41 (d, *J* = 6.7 Hz, 1H), 3.74 (s, 3H); **¹³C-NMR** (126 MHz, $CDCl_3$) δ 172.64, 156.97, 148.16, 137.56, 137.44, 129.03, 128.49, 127.56, 114.00, 108.49, 58.79, 52.80; **HRMS (ESI)** calcd. for $[M+H]^+$: 243.1128 m/z, found: 243.1128 m/z.

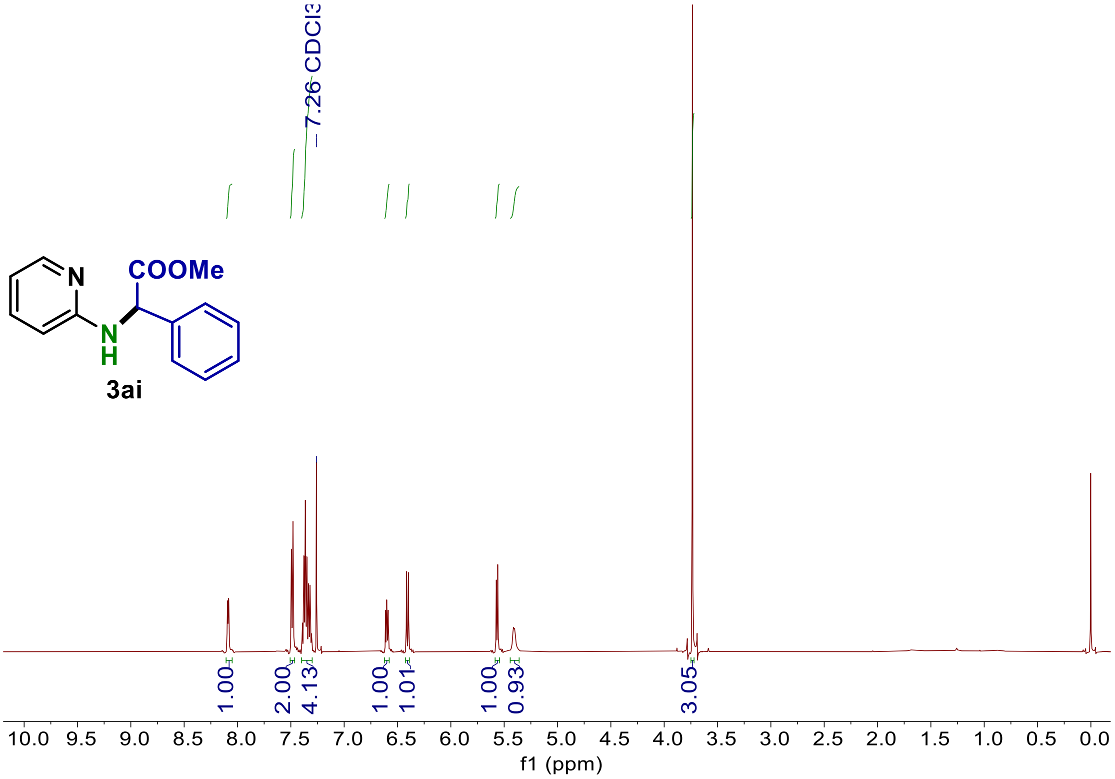

Figure S118. ¹H-NMR spectra of 3ai.

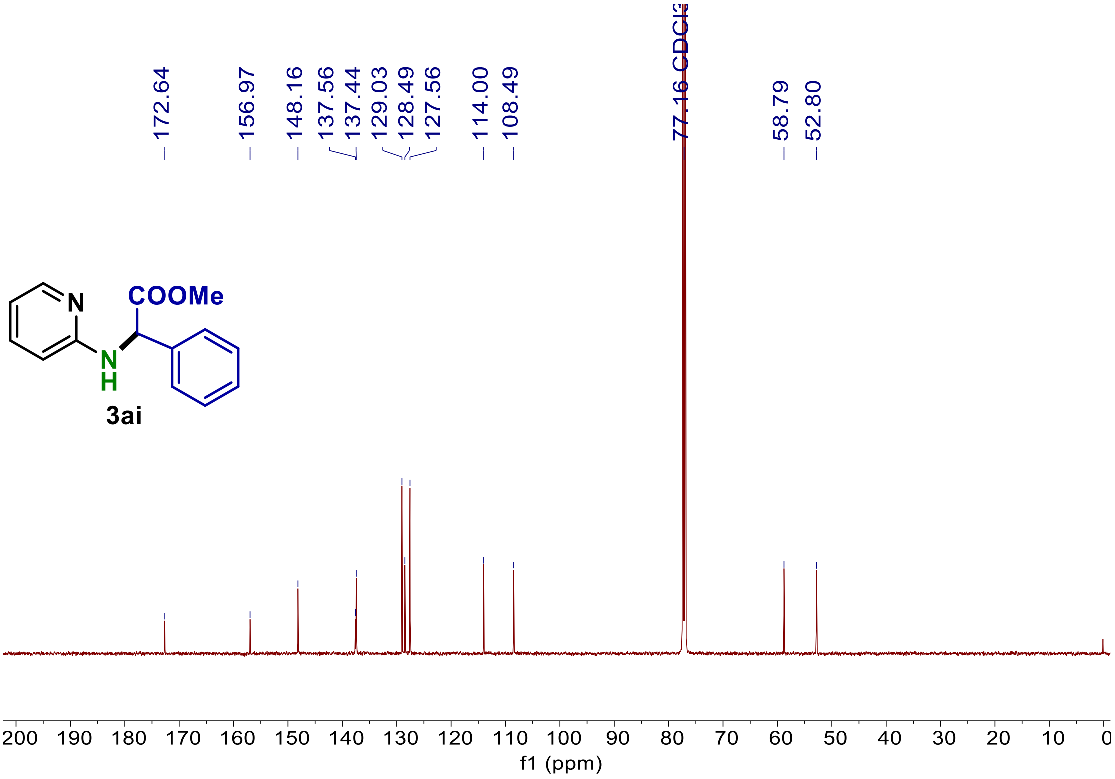

Figure S119. ^{13}C-NMR spectra of 3ai.

Methyl (R)-2-phenyl-2-((6-(trifluoromethyl)pyridin-3-yl)amino)acetate (3aj)

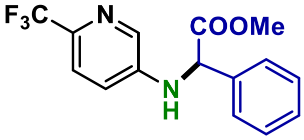

^{1}H NMR (400 MHz, $CDCl_3$) δ p.p.m. 8.07 (d, J = 2.8 Hz, 1H), 7.49 – 7.42 (m, 2H), 7.41 – 7.31 (m, 4H), 6.77 (dd, J = 8.6, 2.8 Hz, 1H), 5.54 (d, J = 5.4 Hz, 1H), 5.08 (d, J = 5.3 Hz, 1H), 3.77 (s, 3H); **^{13}C-NMR** (126 MHz, $CDCl_3$) δ 171.37, 143.71, 136.31, 135.97, 129.37, 129.06, 127.27, 121.24, 121.22, 121.19, 118.58, 59.93, 53.41; **HRMS (ESI)** calcd. for $[M+H]^+$: 311.1002 m/z, found: 311.1002 m/z.

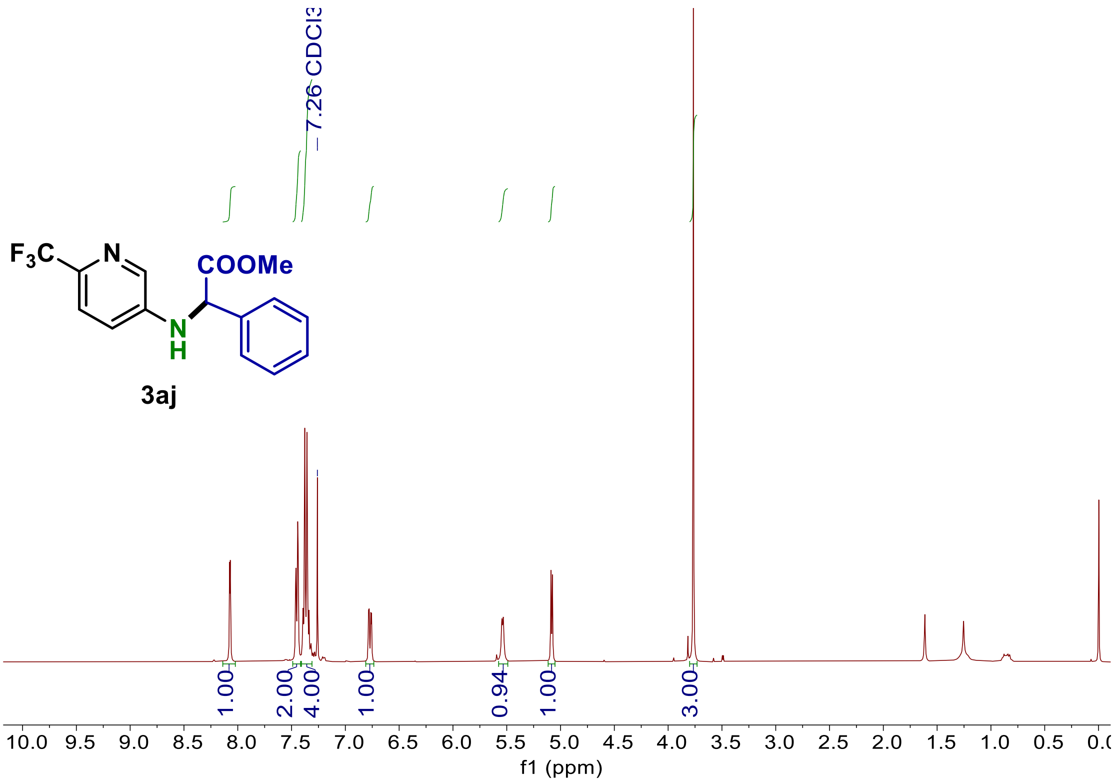

Figure S120. ^{1}H-NMR spectra of 3aj.

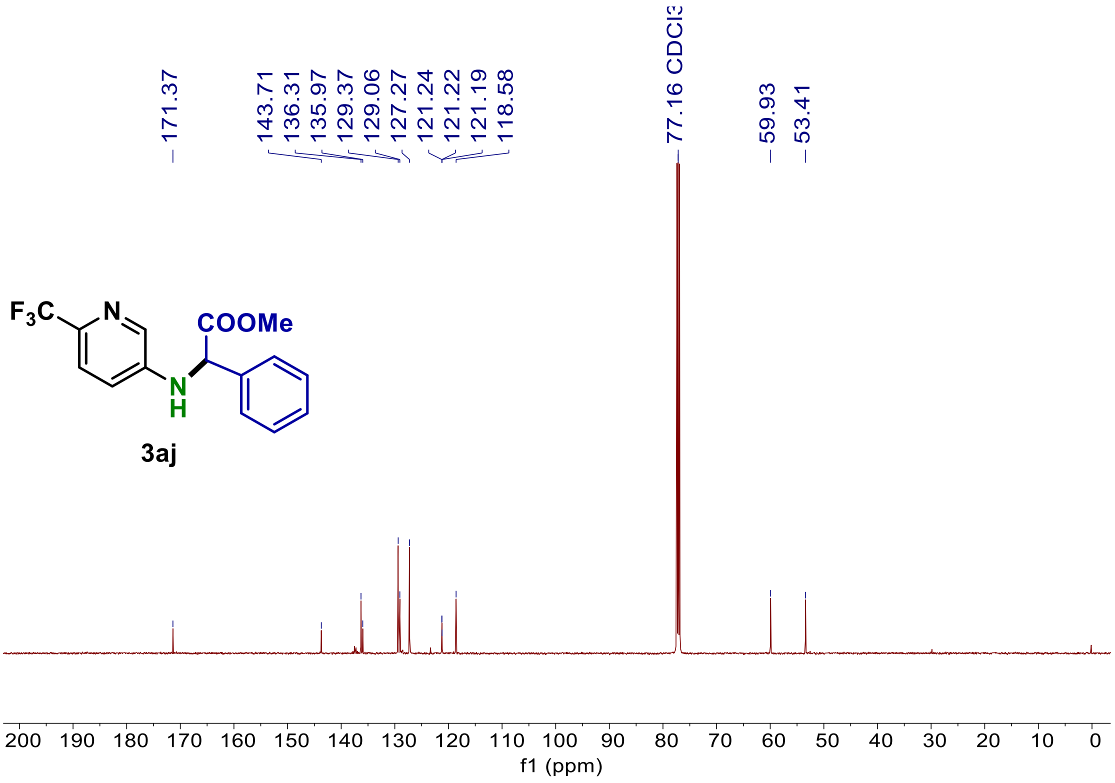

Figure S121. ^{13}C-NMR spectra of 3aj.

Methyl (R)-3-((2-methoxy-2-oxo-1-phenylethyl)amino)thiophene-2-carboxylate (3ak)

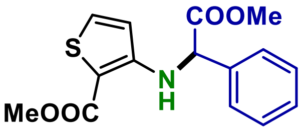

¹H-NMR (500 MHz, $CDCl_3$) δ p.p.m. 7.87 (d, *J* = 6.2 Hz, 1H), 7.49 – 7.45 (m, 2H), 7.39 – 7.29 (m, 3H), 7.22 (d, *J* = 5.4 Hz, 1H), 6.36 (d, *J* = 5.5 Hz, 1H), 5.17 (d, *J* = 6.4 Hz, 1H), 3.86 (s, 3H), 3.74 (s, 3H); **¹³C-NMR** (126 MHz, $CDCl_3$) δ 171.22, 165.21, 153.54, 137.35, 132.22, 129.13, 128.67, 127.24, 116.69, 101.18, 62.04, 53.06, 51.46; **HRMS (ESI)** calcd. for $[M+H]^+$: 306.0795m/z, found: 306.0795 m/z.

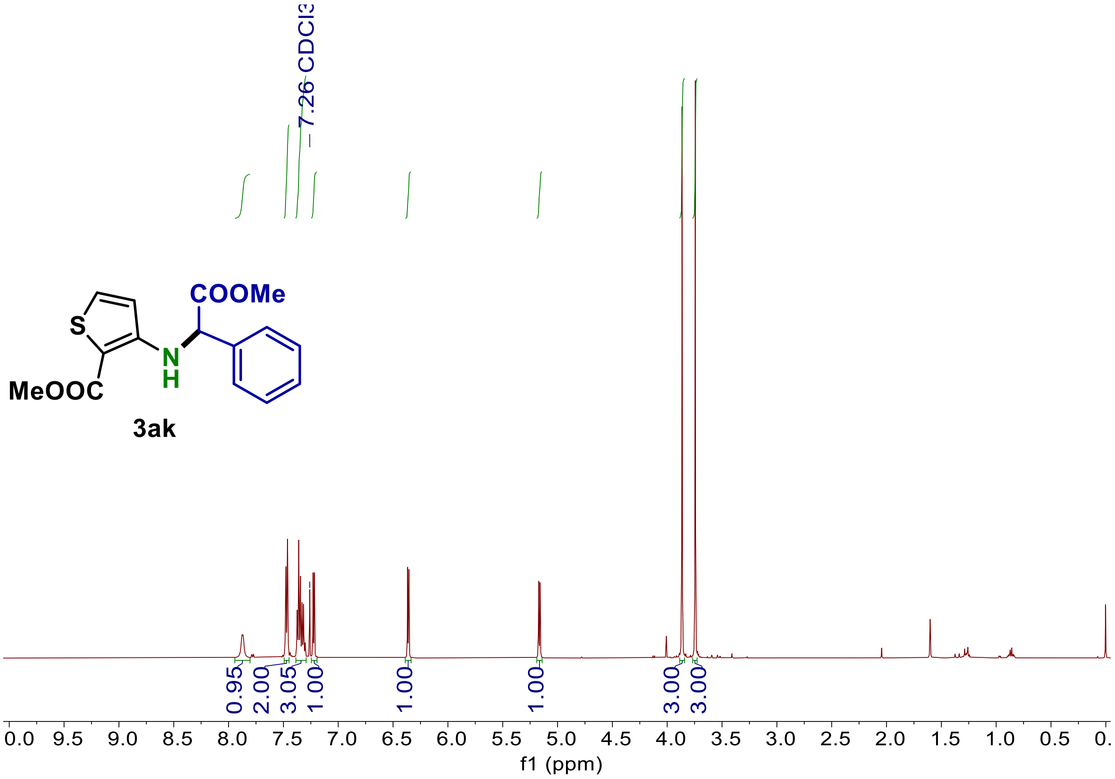

Figure S122. ¹H-NMR spectra of 3ak.

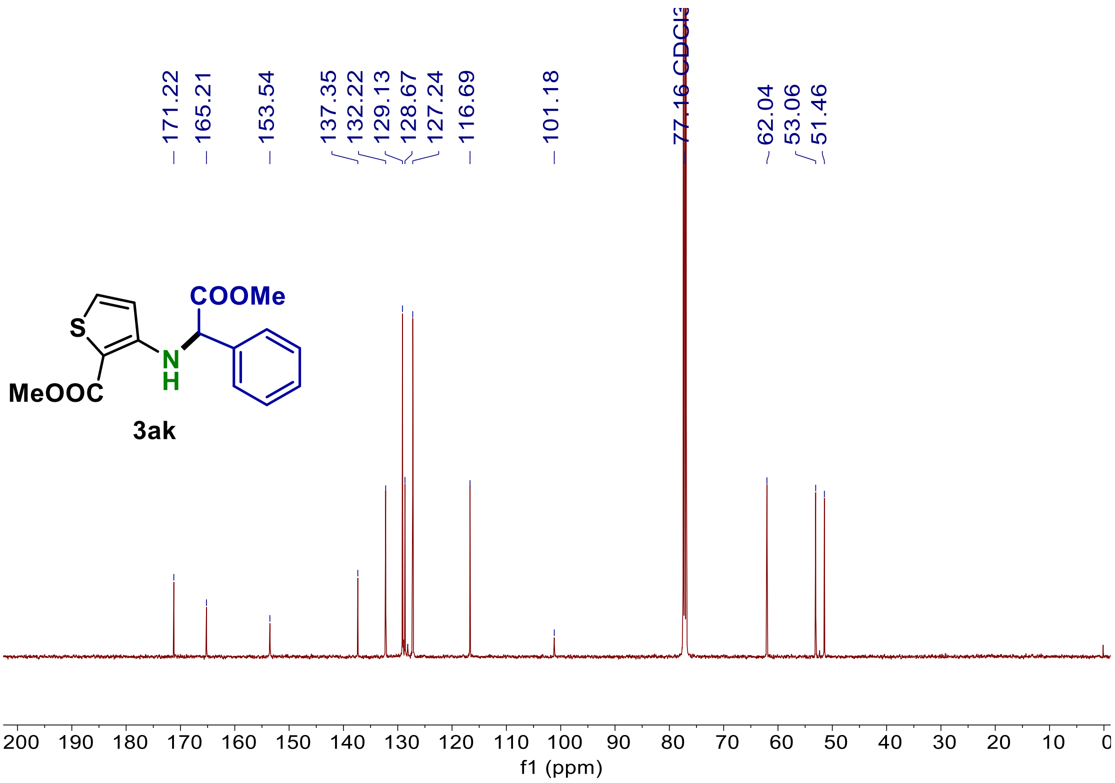

Figure S123. ^{13}C-NMR spectra of 3ak.

Methyl (R)-2-phenyl-2-(quinolin-3-ylamino)acetate (3al)

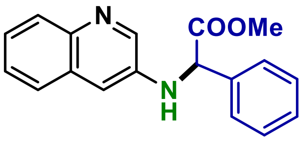

^{1}H-NMR (500 MHz, $CDCl_3$) δ p.p.m. 8.55 (d, J = 2.8 Hz, 1H), 7.94 – 7.90 (m, 1H), 7.55 – 7.51 (m, 2H), 7.49 (dd, J = 7.9, 1.7 Hz, 1H), 7.40 – 7.36 (m, 3H), 7.36 – 7.31 (m, 2H), 6.82 (d, J = 2.8 Hz, 1H), 5.36 (d, J = 5.7 Hz, 1H), 5.17 (d, J = 5.7 Hz, 1H), 3.77 (s, 3H); **^{13}C-NMR** (126 MHz, $CDCl_3$) δ 171.84, 143.35, 142.48, 139.23, 136.53, 129.23, 129.15, 129.12, 128.83, 127.35, 127.05, 126.18, 125.48, 111.69, 60.48, 53.22; **HRMS (ESI)** calcd. for $[M+H]^+$: 293.1285 m/z, found: 293.1285 m/z.

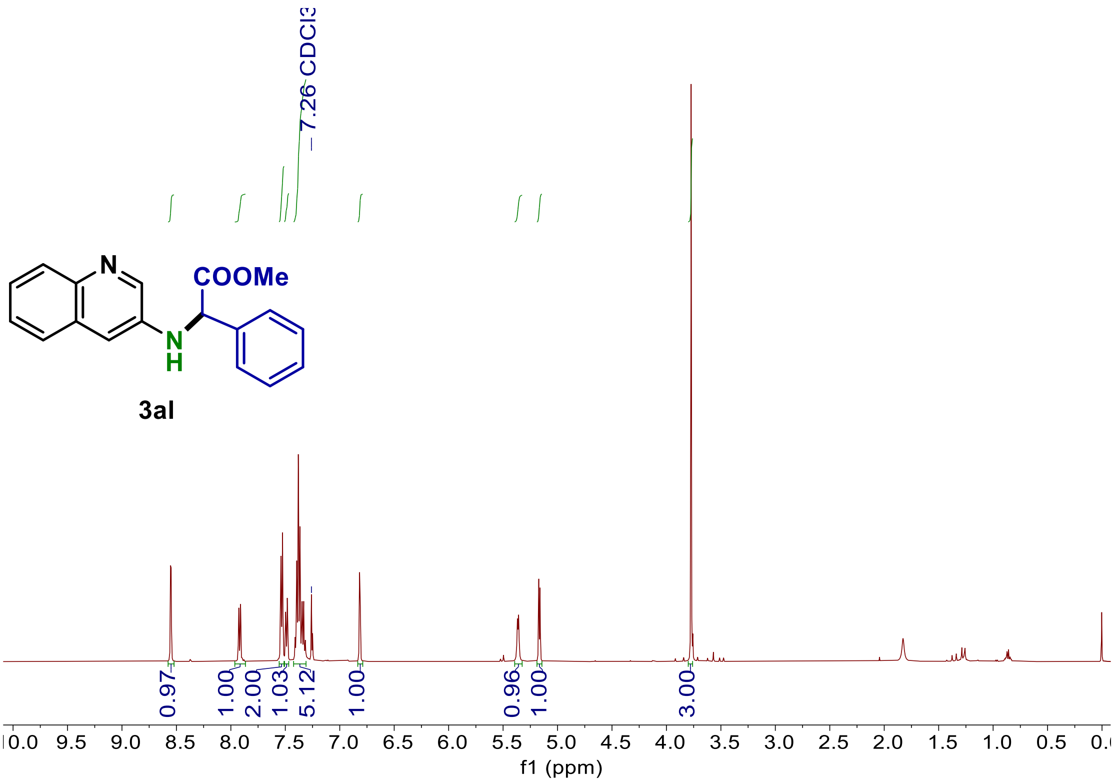

Figure S124. ^{1}H-NMR spectra of 3al.

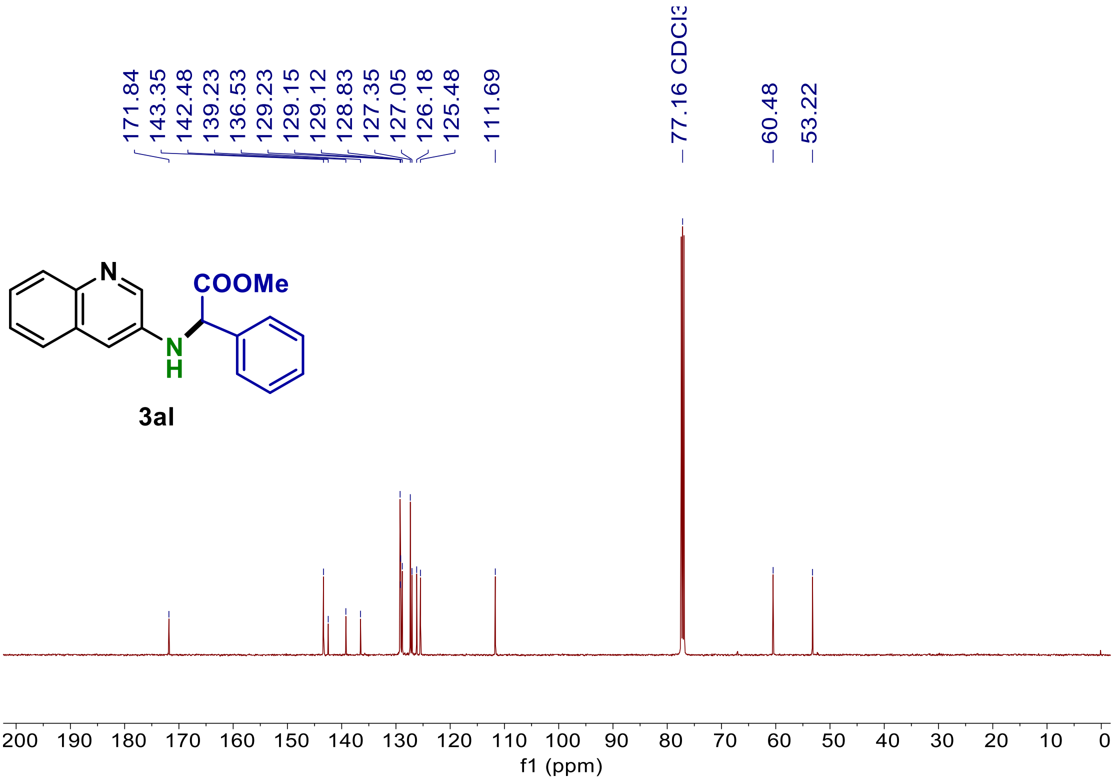

Figure S125. ^{13}C-NMR spectra of 3al.

Methyl (R)-2-((4-(2-hydroxyethyl)phenyl)amino)-2-phenylacetate (3am)[6]

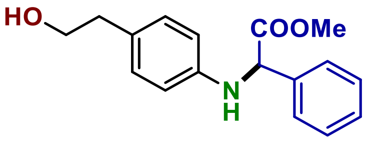

^{1}H-NMR (500 MHz, $CDCl_3$) δ p.p.m. 7.51 – 7.47 (m, 2H), 7.39 – 7.33 (m, 2H), 7.33 – 7.29 (m, 1H), 6.98 (d, *J* = 8.4 Hz, 2H), 6.52 (d, *J* = 8.4 Hz, 2H), 5.06 (s, 1H), 3.76 (t, *J* = 6.5 Hz, 2H), 3.73 (s, 3H), 2.72 (t, *J* = 6.5 Hz, 2H); **^{13}C-NMR** (126 MHz, $CDCl_3$) δ 172.50, 144.74, 137.77, 129.99, 129.04, 128.48, 127.79, 127.40, 113.80, 63.99, 61.01, 52.96, 38.39; **HRMS (ESI)** calcd. for $[M+H]^+$: 286.1438 m/z, found: 286.1434 m/z.

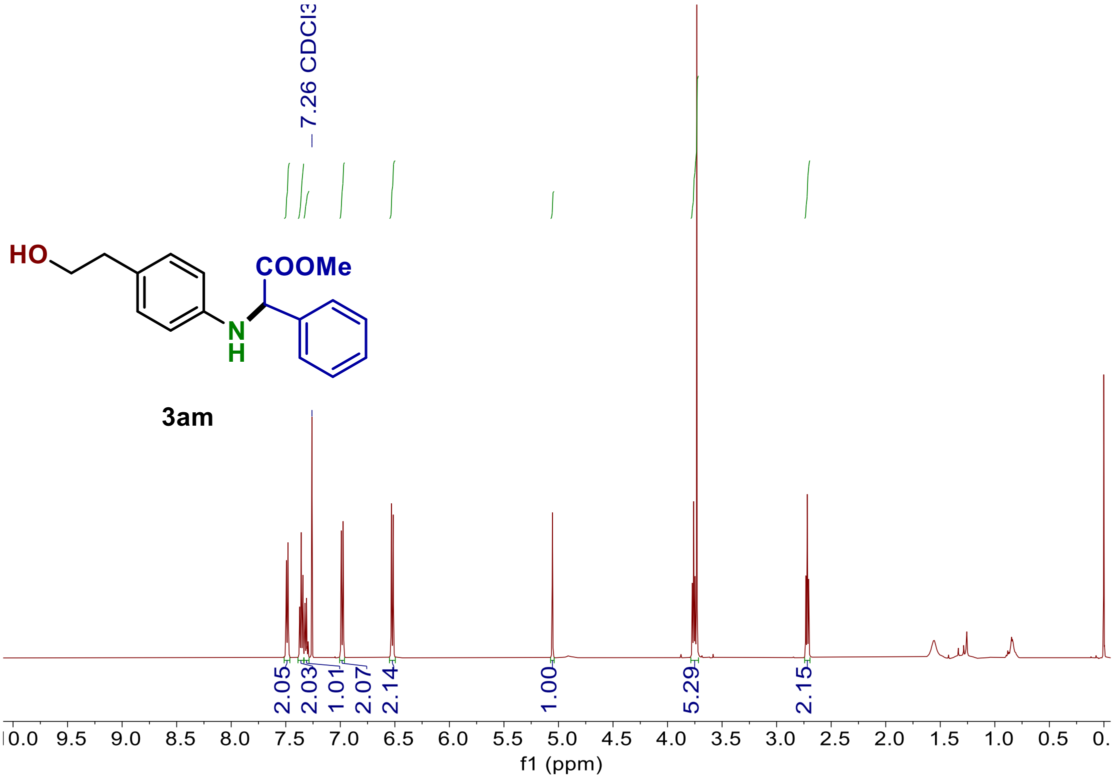

Figure S126. ^{1}H-NMR spectra of 3am.

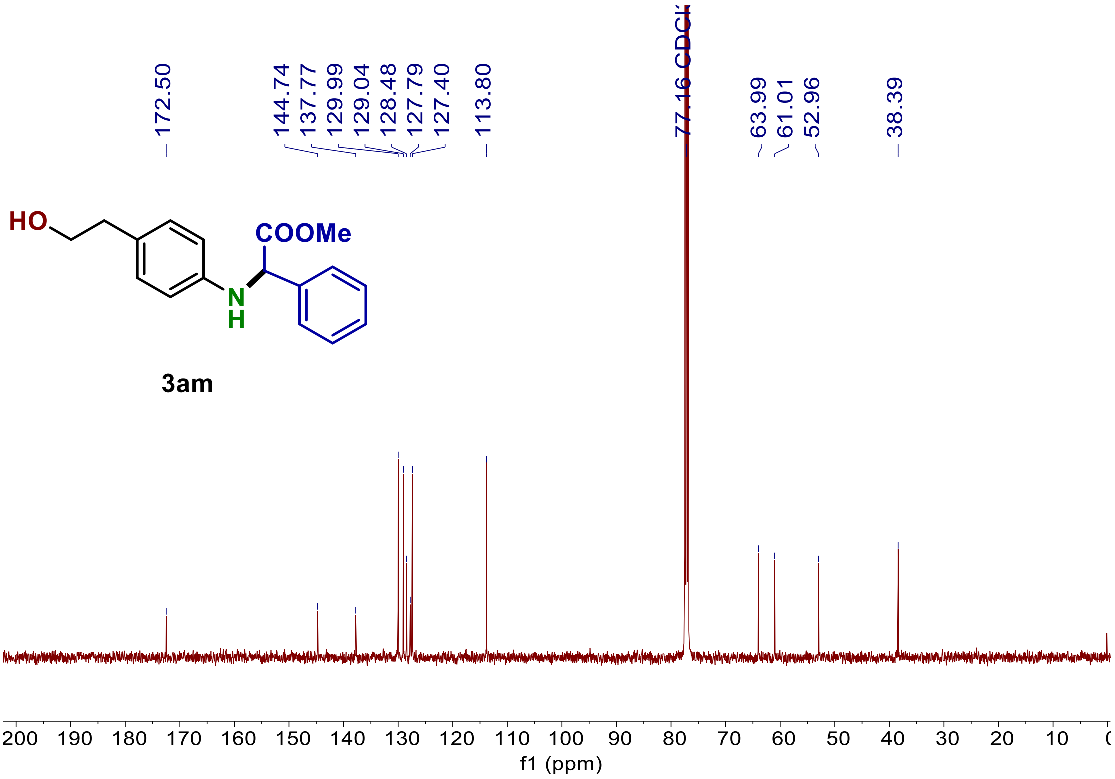

Figure S127. ^{13}C-NMR spectra of 3am.

Methyl (R)-2-((4-(2-((R)-2-methoxy-2-oxo-1-phenylethoxy)ethyl)phenyl)amino)-2-phenylacetate (4am) [6]

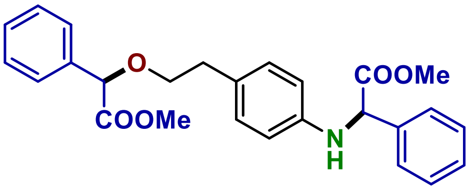

^{1}H-NMR (500 MHz, $CDCl_3$) δ p.p.m. 7.50 – 7.46 (m, 2H), 7.40 (dt, J = 7.6, 1.9 Hz, 2H), 7.37 – 7.27 (m, 6H), 6.95 (d, J = 8.4 Hz, 2H), 6.48 (d, J = 8.4 Hz, 2H), 5.05 (s, 1H), 4.85 (d, J = 2.6 Hz, 1H), 3.72 (s, 3H), 3.71 – 3.61 (m, 4H), 3.57 – 3.49 (m, 1H), 2.83 (dqd, J = 16.1, 7.3, 3.9 Hz, 2H); **^{13}C-NMR** (126 MHz, $CDCl_3$) δ 172.50, 171.50, 144.53, 137.79, 136.63, 129.88, 129.00, 128.74, 128.71, 128.43, 127.89, 127.38, 127.31, 113.59, 81.27, 71.33, 61.01, 52.92, 52.36, 35.42; **HRMS (ESI)** calcd. for $[M+H]^+$: 434.1962 m/z, found: 434.1976 m/z.

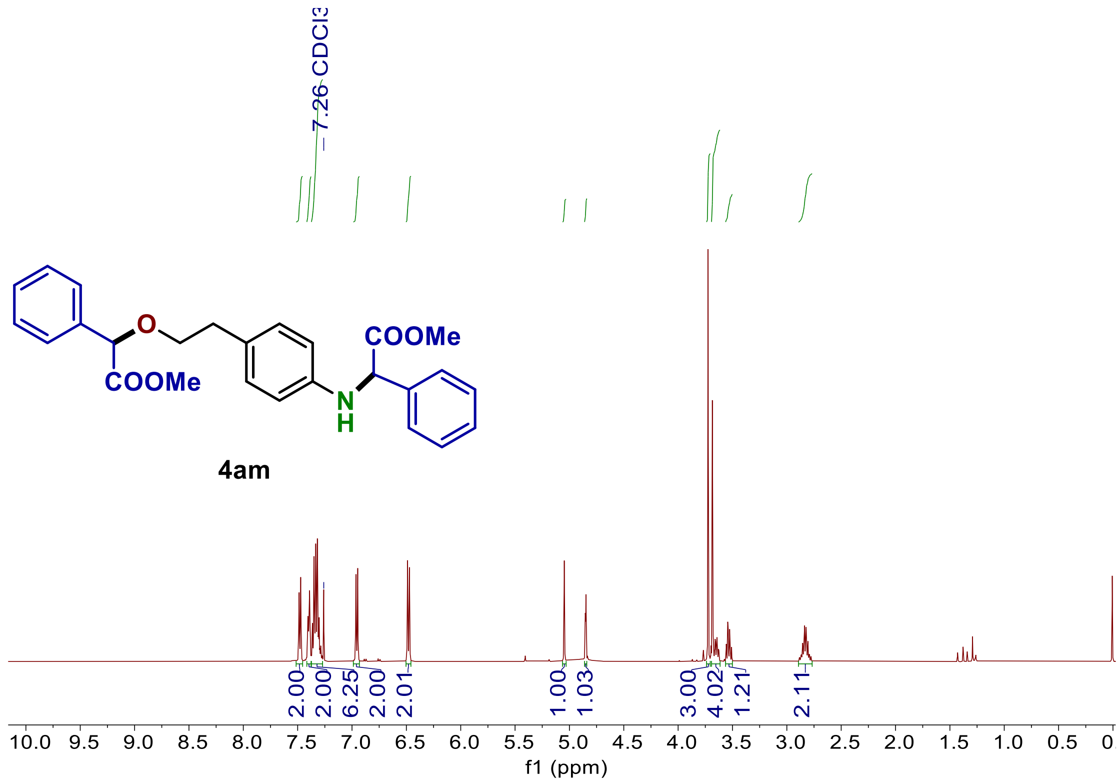

Figure S128. ^{1}H-NMR spectra of 4am.

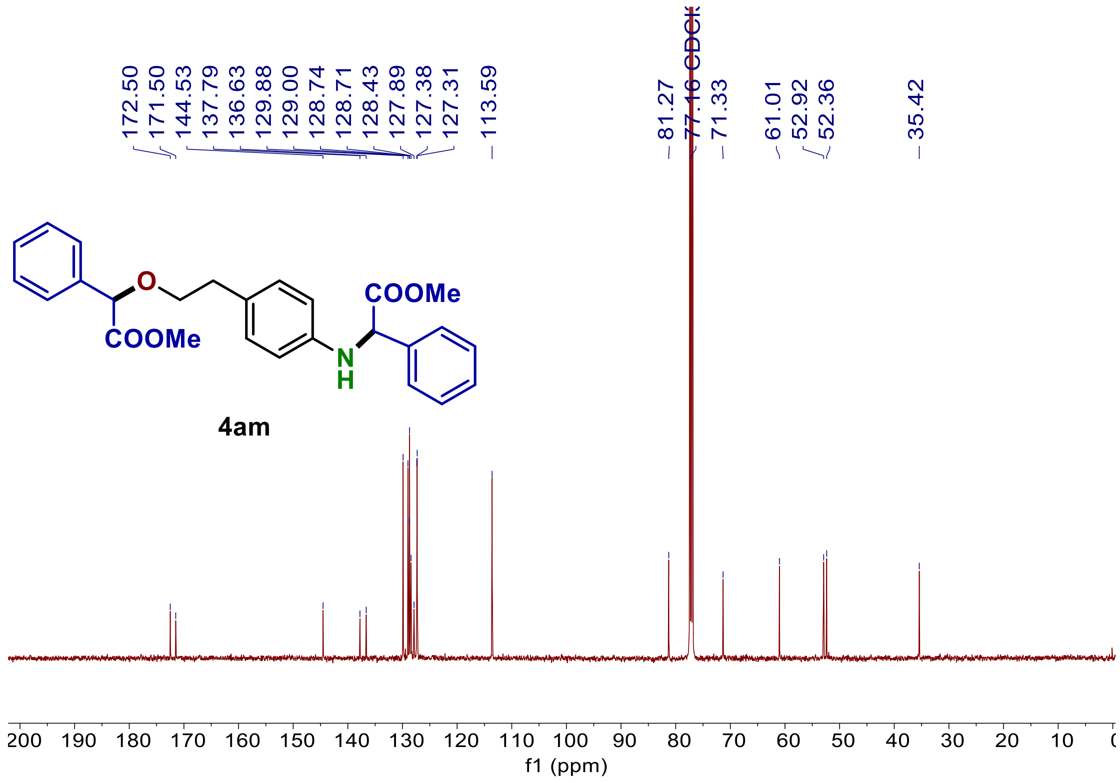

Figure S129. ^{13}C-NMR spectra of 4am.

Methyl (R)-2-((4-(hydroxymethyl)phenyl)amino)-2-phenylacetate (3an)

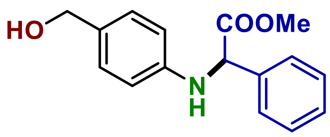

^{1}H-NMR (500 MHz, $CDCl_3$) δ p.p.m. 7.48 (dt, *J* = 6.3, 1.4 Hz, 2H), 7.37 – 7.32 (m, 2H), 7.32 – 7.28 (m, 1H), 7.11 – 7.05 (m, 2H), 6.54 – 6.49 (m, 2H), 5.07 (s, 1H), 4.95 (s, 1H), 4.31 (s, 2H), 3.73 (s, 3H); **^{13}C-NMR** (101 MHz, $CDCl_3$) δ 172.43, 145.54, 137.63, 129.63, 129.02, 128.46, 128.02, 127.38, 113.38, 71.63, 60.83, 52.99; **HRMS (ESI)** calcd. for [M+H]$^+$: 272.1281 m/z, found: 272.1281 m/z.

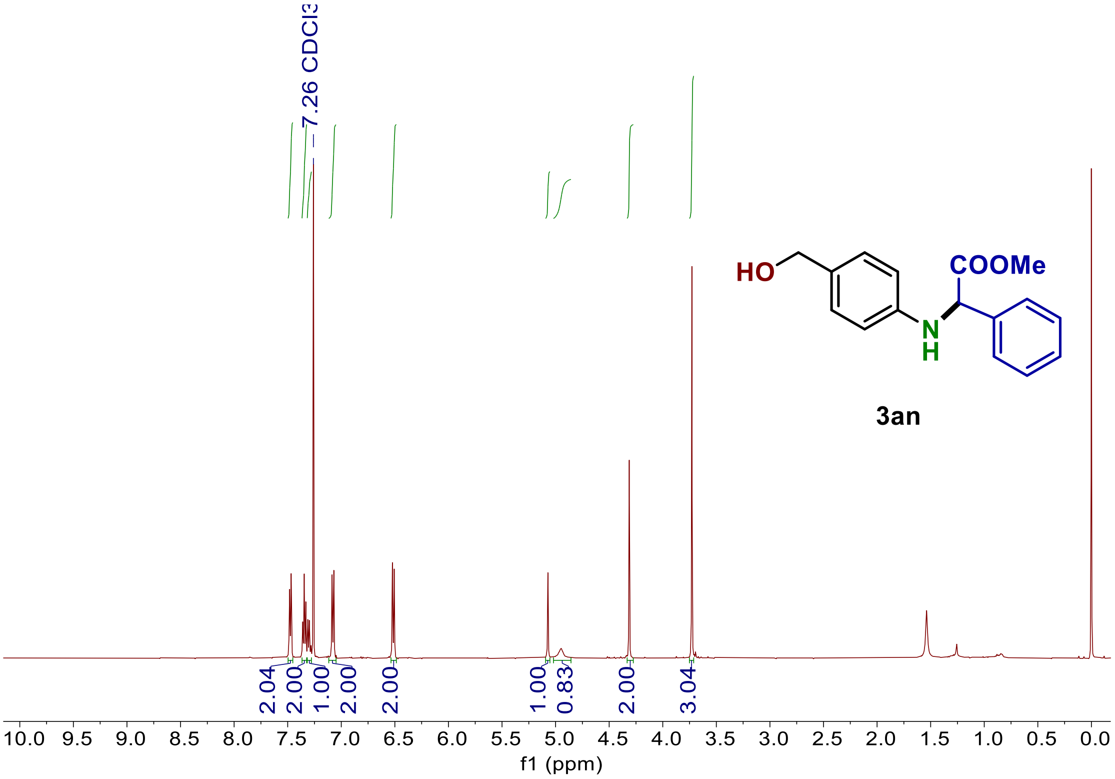

Figure S130. ^{1}H-NMR spectra of 3an.

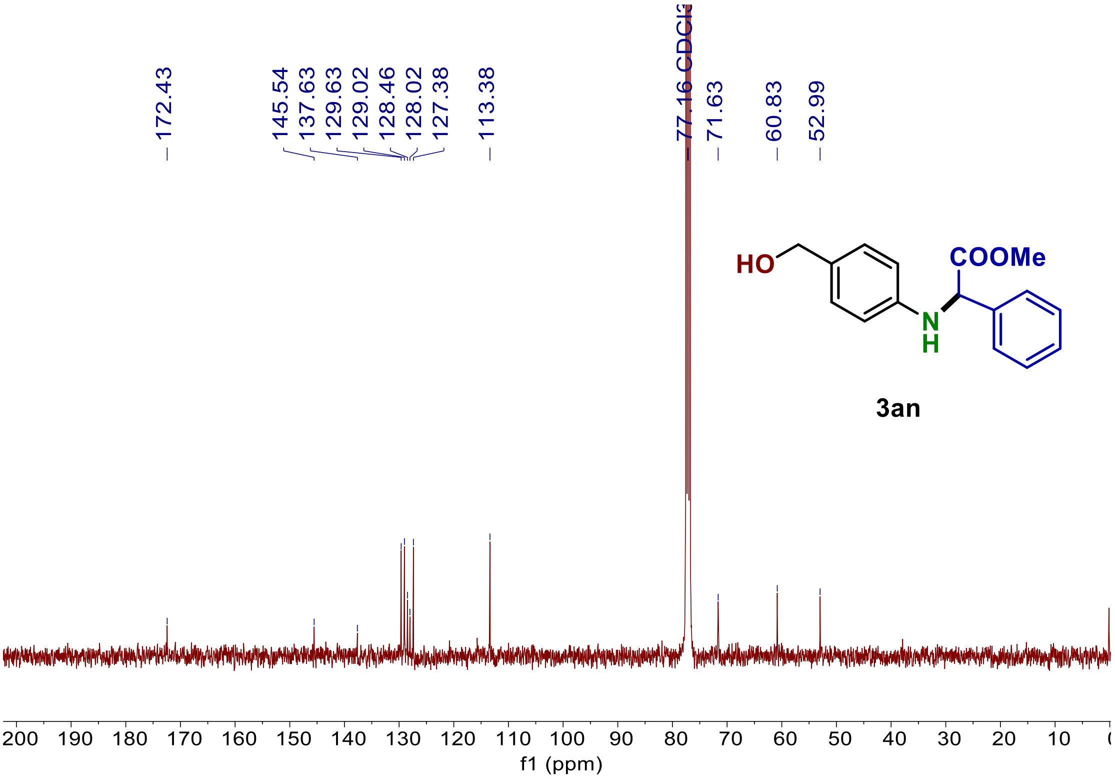

Figure S131. ^{13}C-NMR spectra of 3an.

Methyl (R)-2-((4-(((S)-2-methoxy-2-oxo-1-phenylethoxy)methyl)phenyl)amino)-2-phenylacetate (4an)

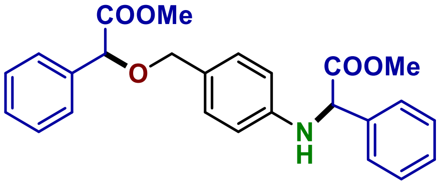

^{1}H-NMR (400 MHz, $CDCl_3$) δ p.p.m. 7.51 – 7.45 (m, 2H), 7.41 (dt, J = 5.6, 2.0 Hz, 2H), 7.39 – 7.29 (m, 6H), 7.09 (d, J = 8.3 Hz, 2H), 6.52 (d, J = 8.4 Hz, 2H), 5.08 (s, 1H), 4.88 (s, 1H), 4.48 – 4.35 (m, 2H), 3.74 (s, 3H), 3.67 (d, J = 1.7 Hz, 3H); **^{13}C-NMR** (101 MHz, $CDCl_3$) δ 172.35, 171.56, 145.91, 137.56, 136.56, 130.07, 129.05, 128.74, 128.51, 127.57, 127.37, 126.37, 113.38, 78.98, 71.07, 60.76, 53.03, 52.38; **HRMS (ESI)** calcd. for $[M+H]^+$: 420.1805 m/z, found: 420.1808 m/z.

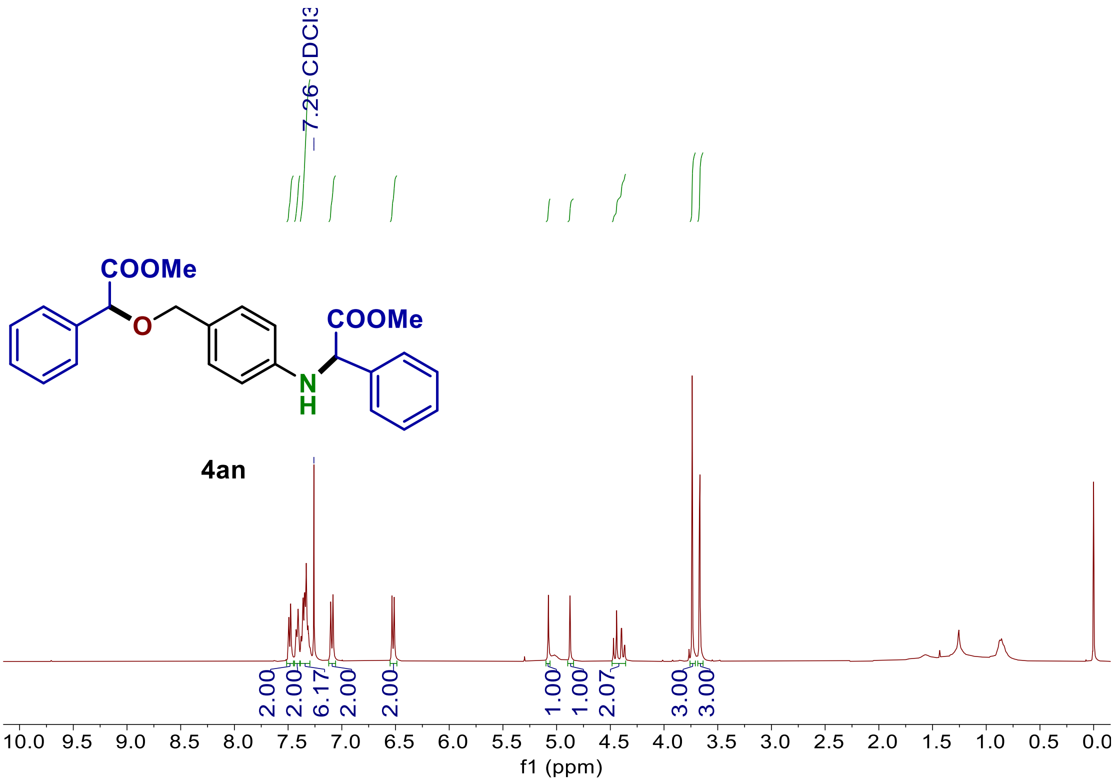

Figure S132. ¹H-NMR spectra of 4an.

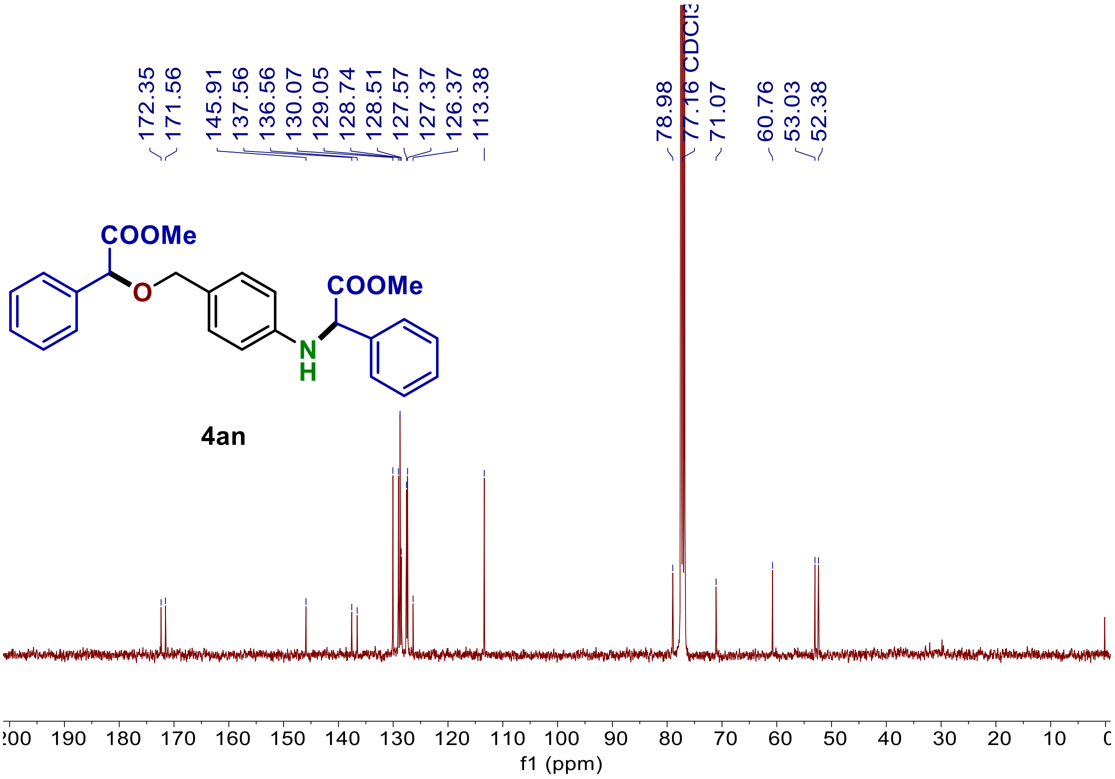

Figure S133. ^{13}C-NMR spectra of 4an.

Methyl (R)-2-((4-hydroxyphenyl)amino)-2-phenylacetate (3ao)[7]

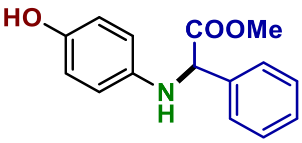

^{1}H-NMR (500 MHz, $CDCl_3$) δ p.p.m. 7.50 – 7.45 (m, 2H), 7.39 – 7.33 (m, 2H), 7.33 – 7.28 (m, 1H), 6.64 (d, *J* = 8.8 Hz, 2H), 6.48 (d, *J* = 8.7 Hz, 2H), 5.01 (s, 1H), 3.72 (s, 3H); **^{13}C-NMR** (126 MHz, $CDCl_3$) δ 172.67, 148.24, 140.35, 137.84, 129.02, 128.46, 127.44, 116.32, 115.11, 61.86, 52.89; **HRMS (ESI)** calcd. for $[M+H]^+$: 258.1125 m/z, found: 258.1126 m/z.

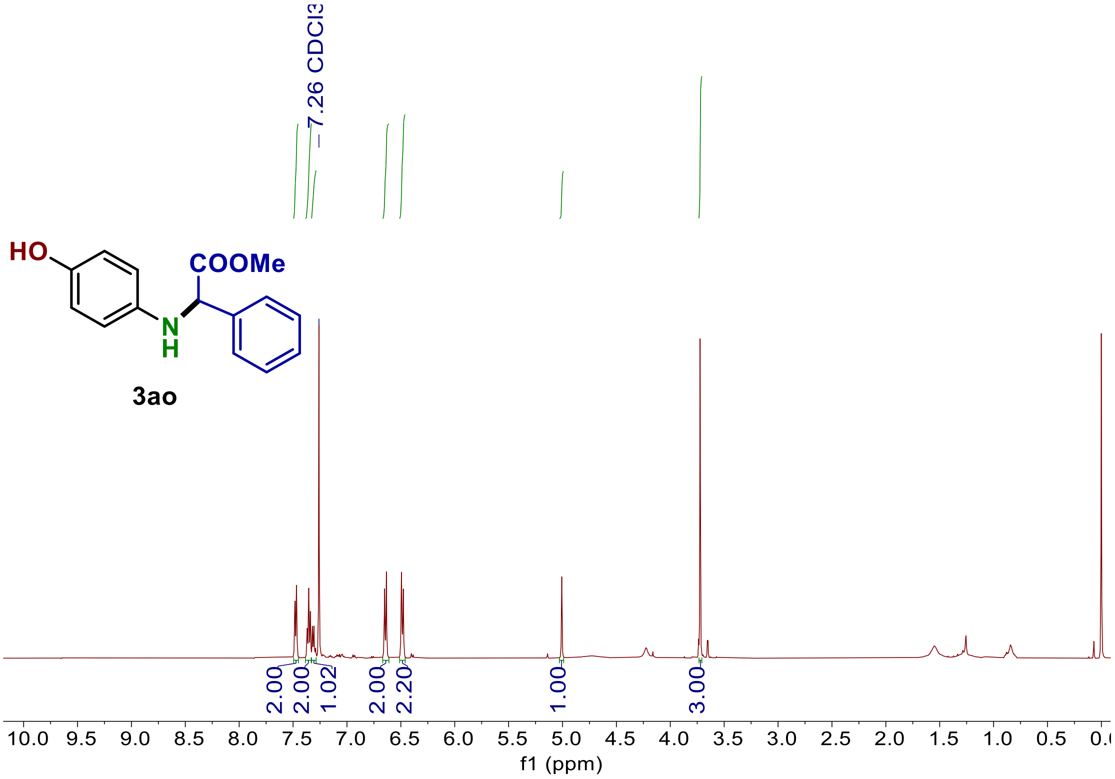

Figure S134. ^{1}H-NMR spectra of 3ao.

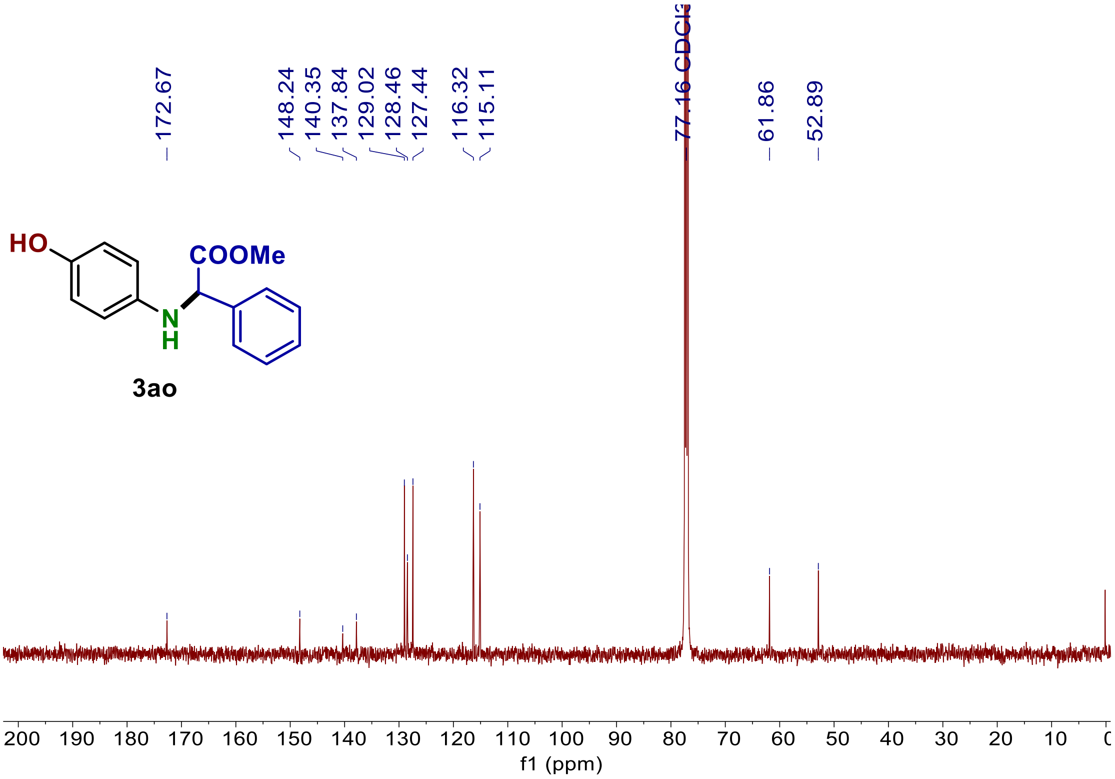

Figure S135. ^{13}C-NMR spectra of 3ao.

Methyl (S)-2-((4-hydroxyphenyl)((R)-2-methoxy-2-oxo-1-phenylethyl)amino)-2-phenylacetate (4ao)

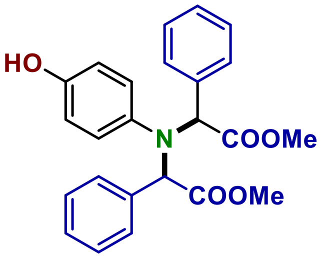

^{1}H-NMR (500 MHz, $CDCl_3$) δ p.p.m. 7.34 – 7.27 (m, 10H), 6.86 (d, *J* = 8.8 Hz, 2H), 6.50 (m, *J* = 8.7 Hz, 2H), 5.24 (s, 2H), 4.44 (s, 1H), 3.52 (s, 6H); **HRMS (ESI)** calcd. for $[M+H]^+$: 406.1649 m/z, found: 406.1653 m/z.

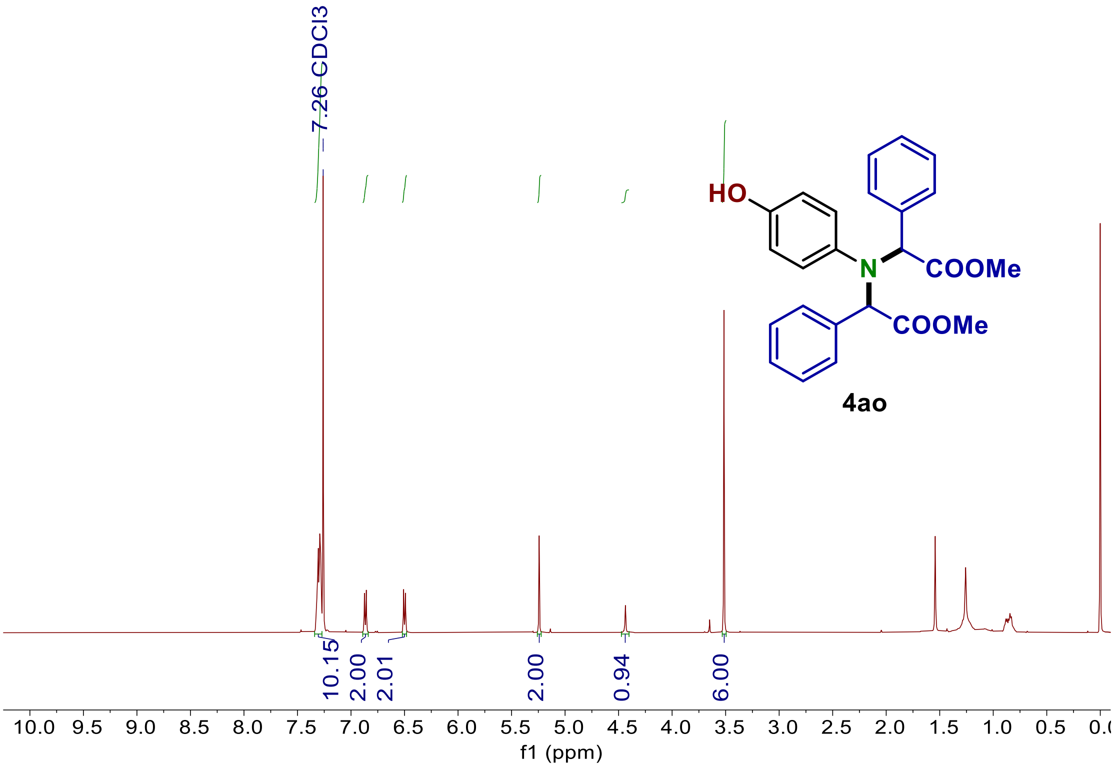

Figure S136. ^{1}H-NMR spectra of 4ao.

Methyl (S)-2-((4-((S)-2-methoxy-2-oxo-1-phenylethoxy)phenyl)((R)-2-methoxy-2-oxo-1-phenylethyl)amino)-2-phenylacetate (5ao)

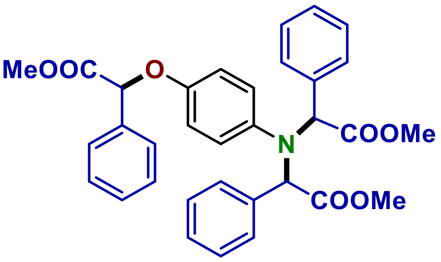

^{1}H-NMR (400 MHz, $CDCl_3$) δ p.p.m. 7.55 – 7.48 (m, 2H), 7.41 – 7.33 (m, 3H), 7.33 – 7.27 (m, 10H), 6.87 – 6.82 (m, 2H), 6.65 – 6.60 (m, 2H), 5.49 (s, 1H), 5.27 (d, *J* = 3.4 Hz, 2H), 3.70 (s, 3H), 3.50 (d, *J* = 3.5 Hz, 6H); **HRMS (ESI)** calcd. for $[M+H]^+$: 554.2173 m/z, found: 554.2183 m/z.

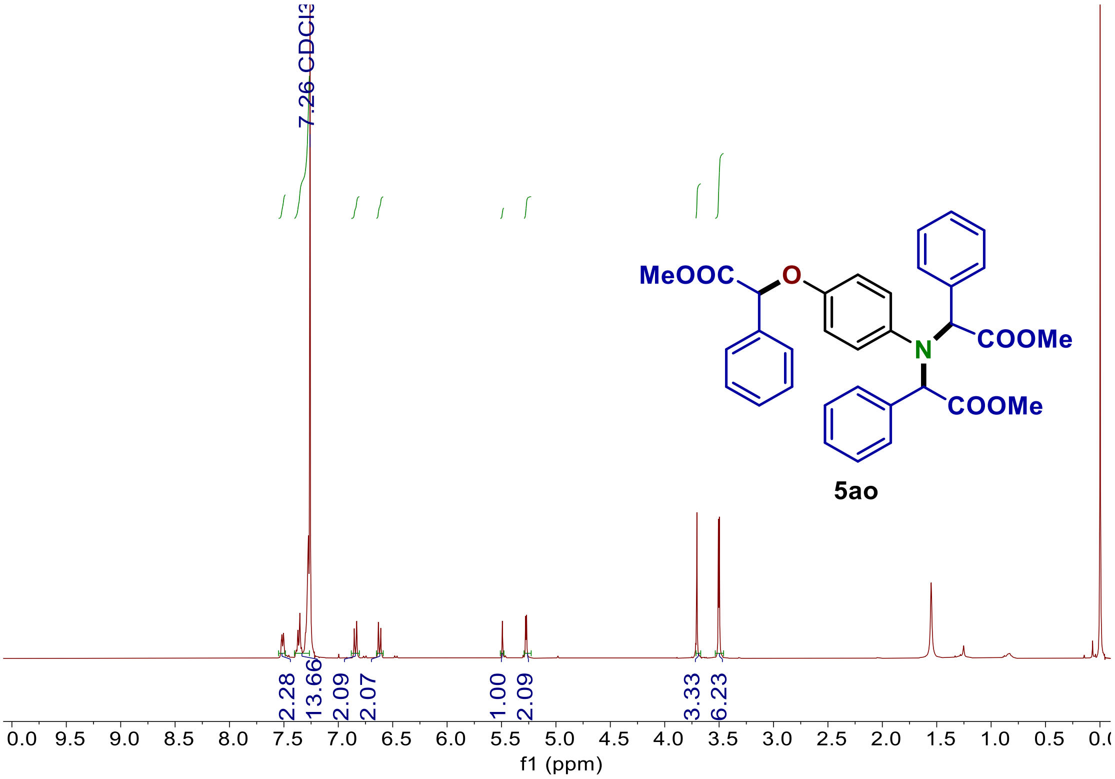

Figure S137. ^{1}H-NMR spectra of 5ao.

Methyl (R)-2-((3-hydroxyphenyl)amino)-2-phenylacetate (3ap)[8]

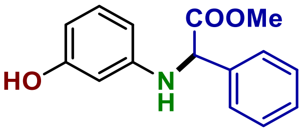

^{1}**H-NMR** (400 MHz, $CDCl_3$) δ p.p.m. 7.52 – 7.42 (m, 2H), 7.39 – 7.28 (m, 3H), 6.97 (t, *J* = 8.1 Hz, 1H), 6.21 – 6.13 (m, 2H), 6.02 (t, *J* = 2.3 Hz, 1H), 5.05 (s, 1H), 3.73 (s, 3H); ^{13}**C-NMR** (101 MHz, $CDCl_3$) δ 172.47, 156.70, 147.60, 137.52, 130.38, 129.05, 128.51, 127.34, 106.51, 105.28, 100.42, 60.75, 53.05; **HRMS (ESI)** calcd. for $[M+H]^+$: 258.1125 m/z, found: 258.1125 m/z.

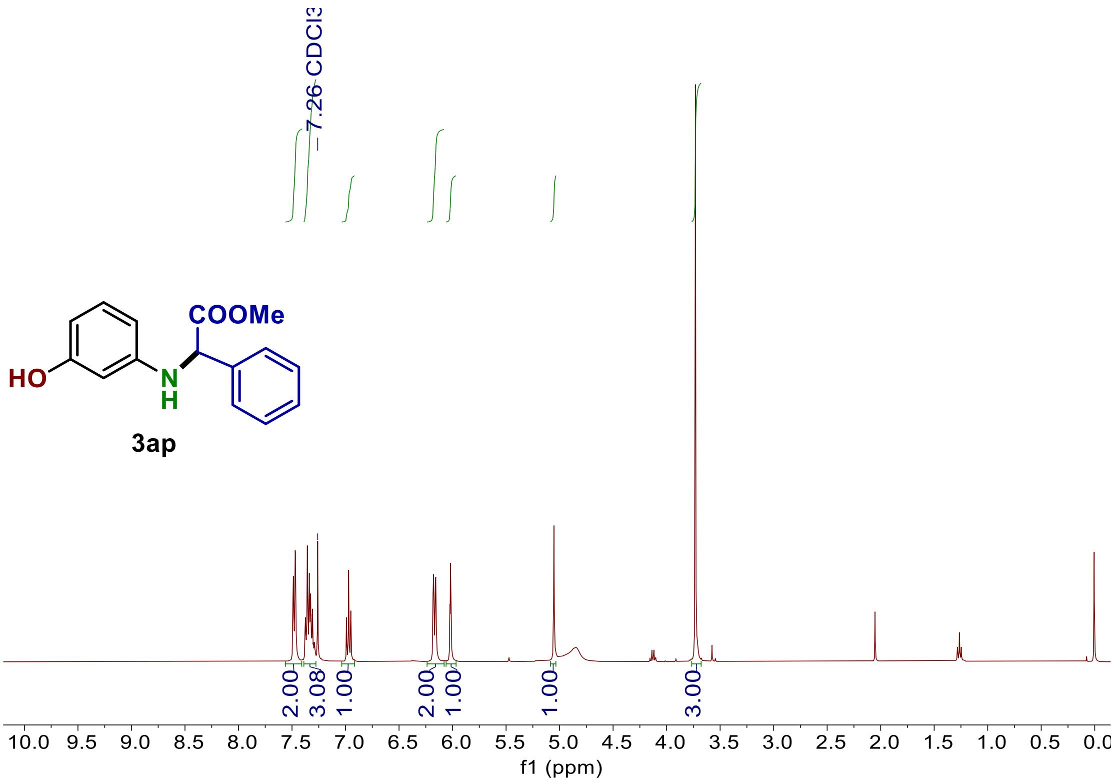

Figure S138. ^{1}H-NMR spectra of 3ap.

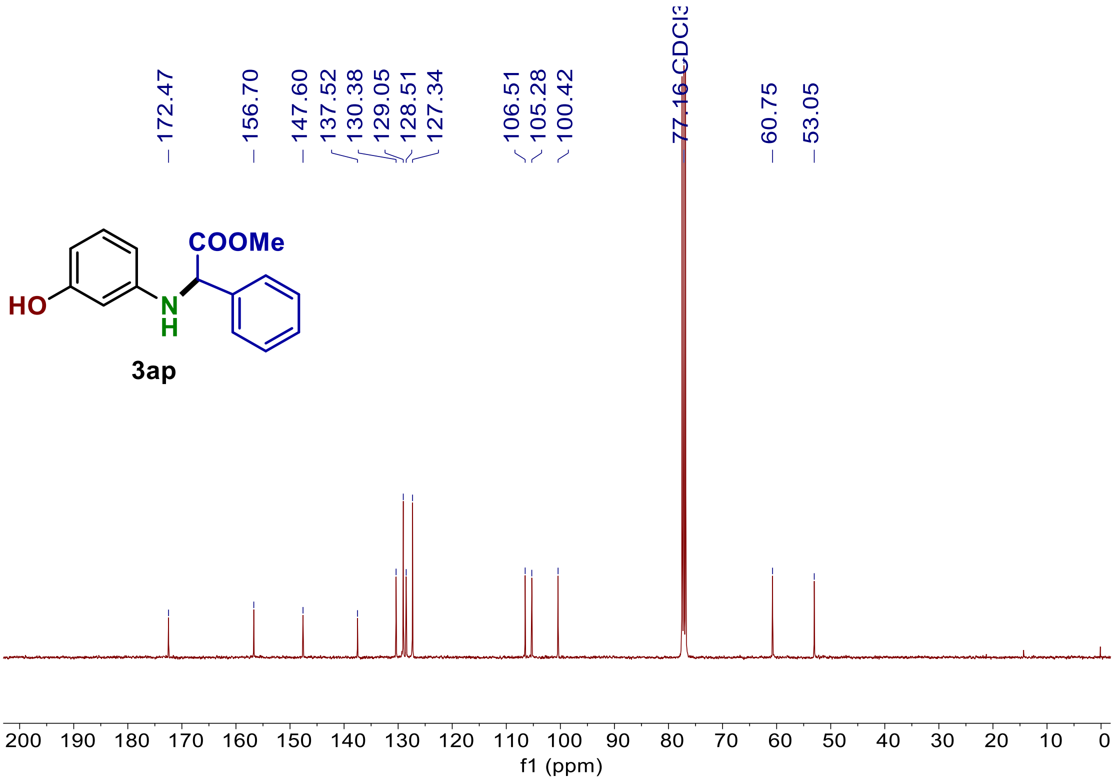

Figure S139. ^{13}C-NMR spectra of 3ap.

Methyl (R)-2-(2-hydroxy-4-(((R)-2-methoxy-2-oxo-1-phenylethyl)amino)phenyl)-2-phenylacetate (4ap)

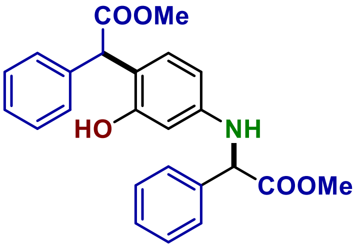

^{1}H-NMR (400 MHz, $CDCl_3$) δ p.p.m. 7.50 – 7.44 (m, 2H), 7.42 – 7.28 (m, 5H), 7.25 – 7.13 (m, 4H), 6.84 (dd, *J* = 8.5, 5.0 Hz, 1H), 6.18 – 6.07 (m, 2H), 5.03 (s, 1H), 4.96 (s, 1H), 3.79 (d, *J* = 1.3 Hz, 3H), 3.73 (s, 3H); **HRMS (ESI)** calcd. for $[M+H]^+$: 406.1649 m/z, found: 406.1657 m/z.

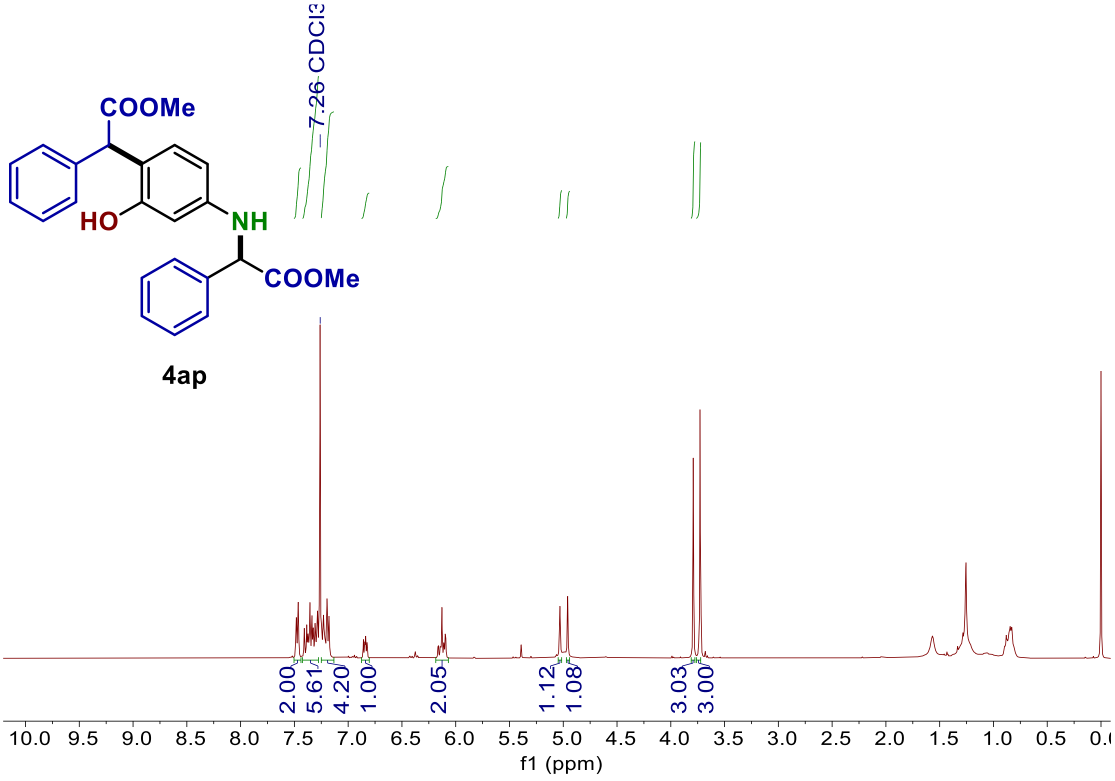

Figure S140. ^{1}H-NMR spectra of 4ap.

Methyl (R)-2-(((S)-2-oxo-3-phenyl-2,3-dihydrobenzofuran-6-yl)amino)-2-phenylacetate (5ap)

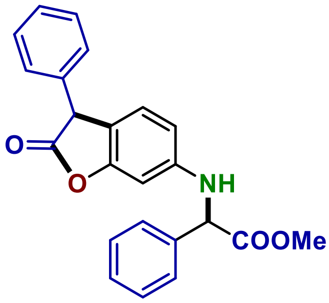

^{1}H-NMR (500 MHz, $CDCl_3$) δ p.p.m. 7.51 – 7.47 (m, 2H), 7.41 – 7.28 (m, 6H), 7.22 – 7.18 (m, 2H), 6.95 – 6.89 (m, 1H), 6.39 – 6.32 (m, 2H), 5.23 (s, 1H), 5.07 (s, 1H), 4.75 (d, *J* = 3.7 Hz, 1H), 3.76 (s, 3H); **HRMS (ESI)** calcd. for $[M+H]^+$: 374.1387 m/z, found: 374.1392 m/z.

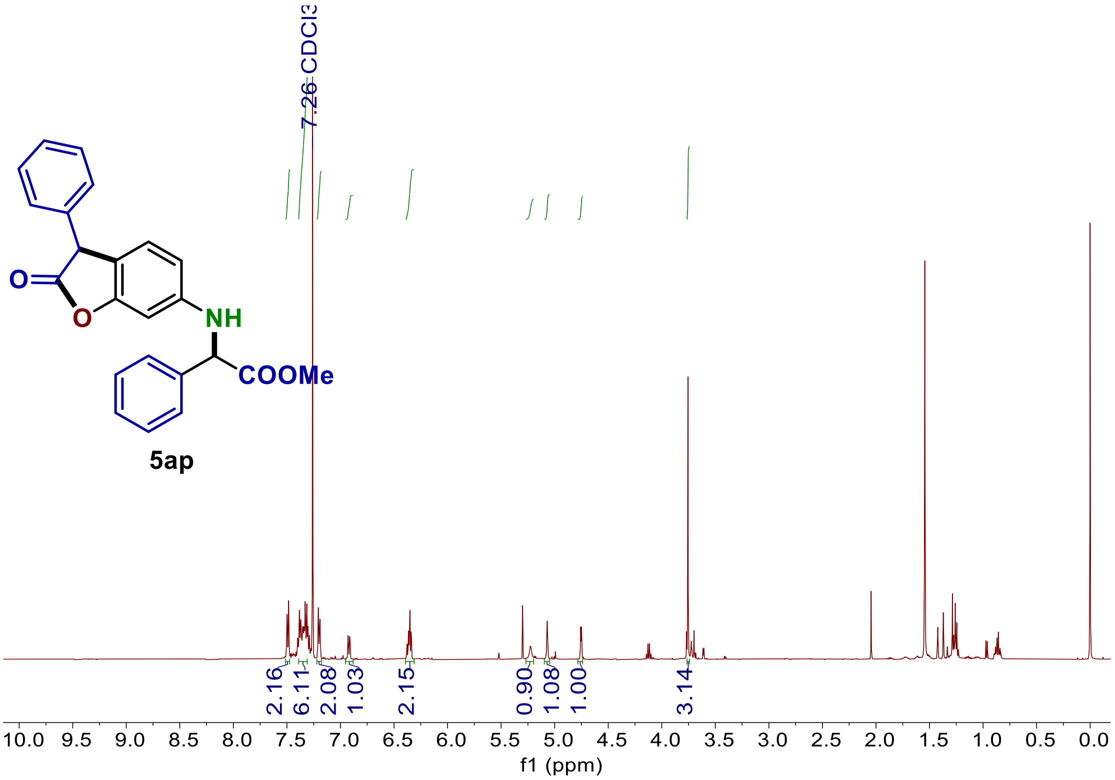

Figure S141. ^{1}H-NMR spectra of 5ap.

Methyl (R)-2-((2-hydroxyphenyl)amino)-2-phenylacetate (3aq)[8]

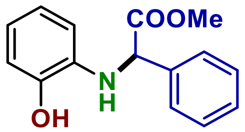

^{1}H-NMR (400 MHz, $CDCl_3$) δ p.p.m. 7.52 – 7.47 (m, 2H), 7.40 – 7.29 (m, 3H), 6.73 (td, J = 5.7, 2.9 Hz, 2H), 6.64 (td, J = 7.5, 1.5 Hz, 1H), 6.48 (dd, J = 8.1, 1.5 Hz, 1H), 5.07 (s, 1H), 3.74 (s, 3H); **^{13}C-NMR** (101 MHz, $CDCl_3$) δ 172.96, 144.41, 137.66, 134.94, 129.04, 128.51, 127.42, 121.53, 119.06, 114.82, 113.77, 61.62, 52.98; **HRMS (ESI)** calcd. for $[M+H]^+$: 258.1125 m/z, found: 258.1124 m/z.

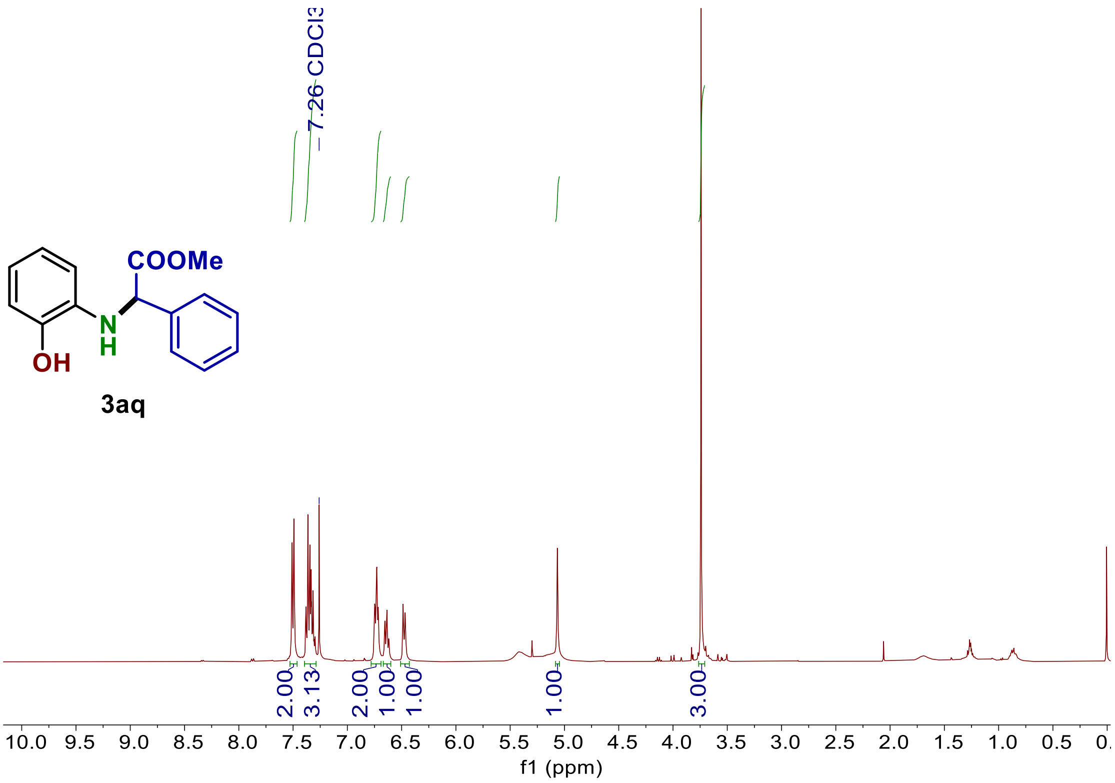

Figure S142. ^{1}H-NMR spectra of 3aq.

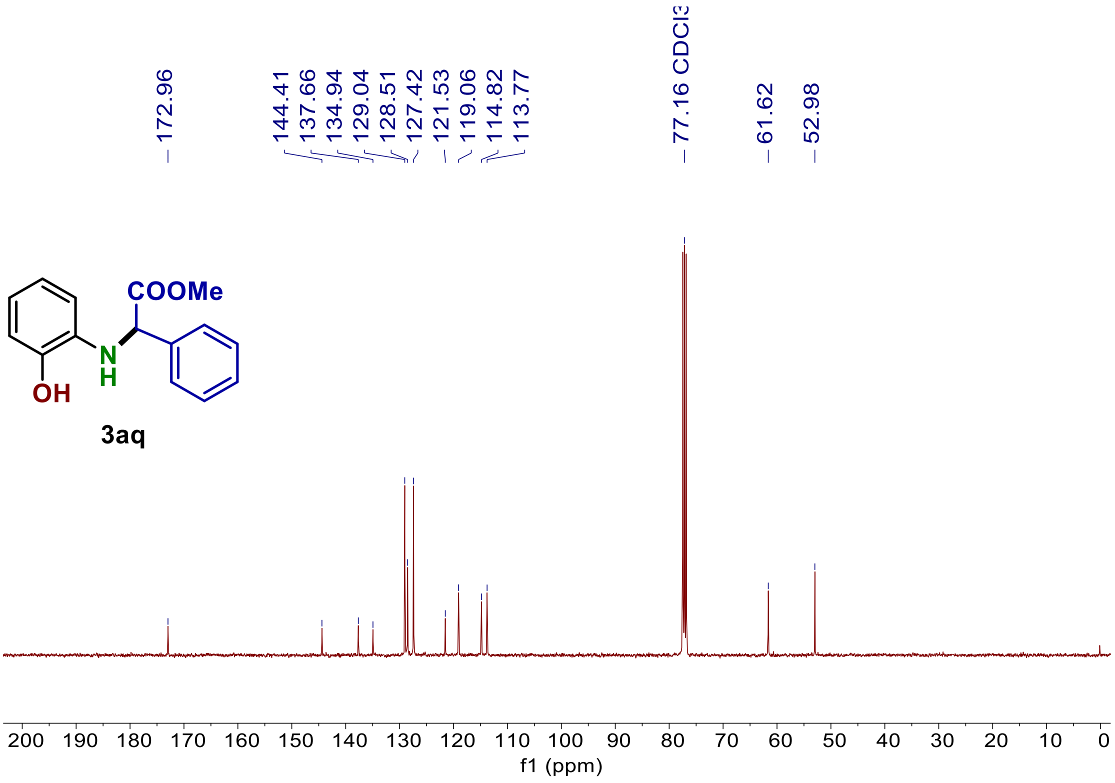

Figure S143. ^{13}C-NMR spectra of 3aq.

Methyl (R)-2-((2-((R)-2-methoxy-2-oxo-1-phenylethoxy)phenyl)amino)-2-phenylacetate (4aq)

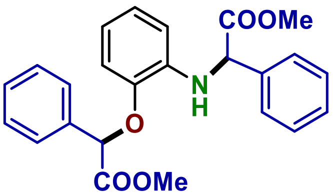

^{1}H-NMR (500 MHz, $CDCl_3$) δ p.p.m. 7.63 (d, *J* = 7.5 Hz, 2H), 7.48 (d, *J* = 7.5 Hz, 2H), 7.41 (dt, *J* = 14.5, 6.9 Hz, 3H), 7.32 (dt, *J* = 15.1, 7.2 Hz,3H), 6.78 – 6.70 (m, 2H), 6.56 (t, *J* = 7.5 Hz, 1H), 6.38 – 6.34 (m, 1H), 5.62 (s, 1H), 5.10 (s, 1H), 3.76 (s, 3H), 3.74 (s, 3H); **HRMS (ESI)** calcd. for $[M+H]^+$: 406.1649 m/z, found: 406.1657 m/z.

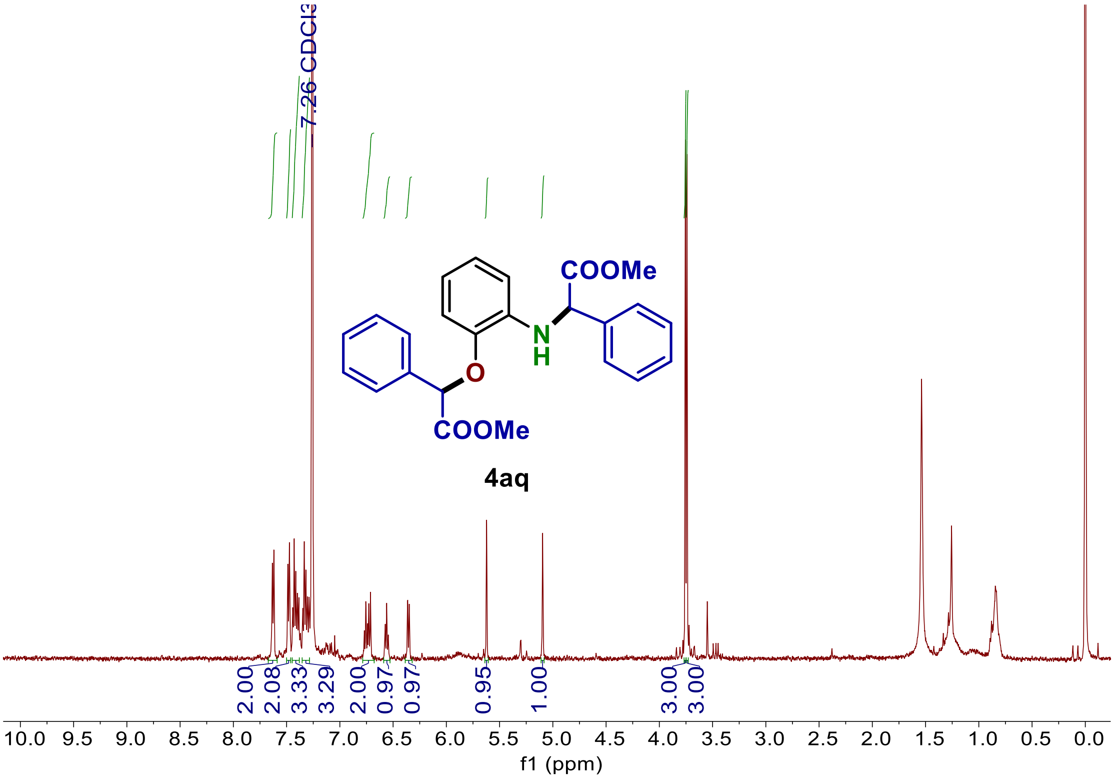

Figure S144. ^{1}H-NMR spectra of 4aq.

Methyl (R)-2-((S)-7-hydroxy-2-oxo-3-phenylindolin-1-yl)-2-phenylacetate (5aq)

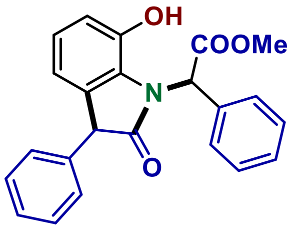

^{1}H-NMR (500 MHz, $CDCl_3$) δ p.p.m. 7.39 (dd, *J* = 5.1, 2.0 Hz, 3H), 7.29 (dd, *J* = 6.8, 2.8 Hz, 2H), 7.22 (dt, *J* = 5.2, 2.8 Hz, 5H), 7.08 (ddd, *J* = 8.4, 5.3, 3.5 Hz, 1H), 7.01 (d, *J* = 8.0 Hz, 1H), 6.97 (d, *J* = 0.9 Hz, 1H), 6.96 (s, 1H), 5.45 (s, 1H), 5.32 (s, 1H), 3.68 (s, 3H); **HRMS (ESI)** calcd. for $[M+H]^+$: 374.1387 m/z, found: 374.1378m/z.

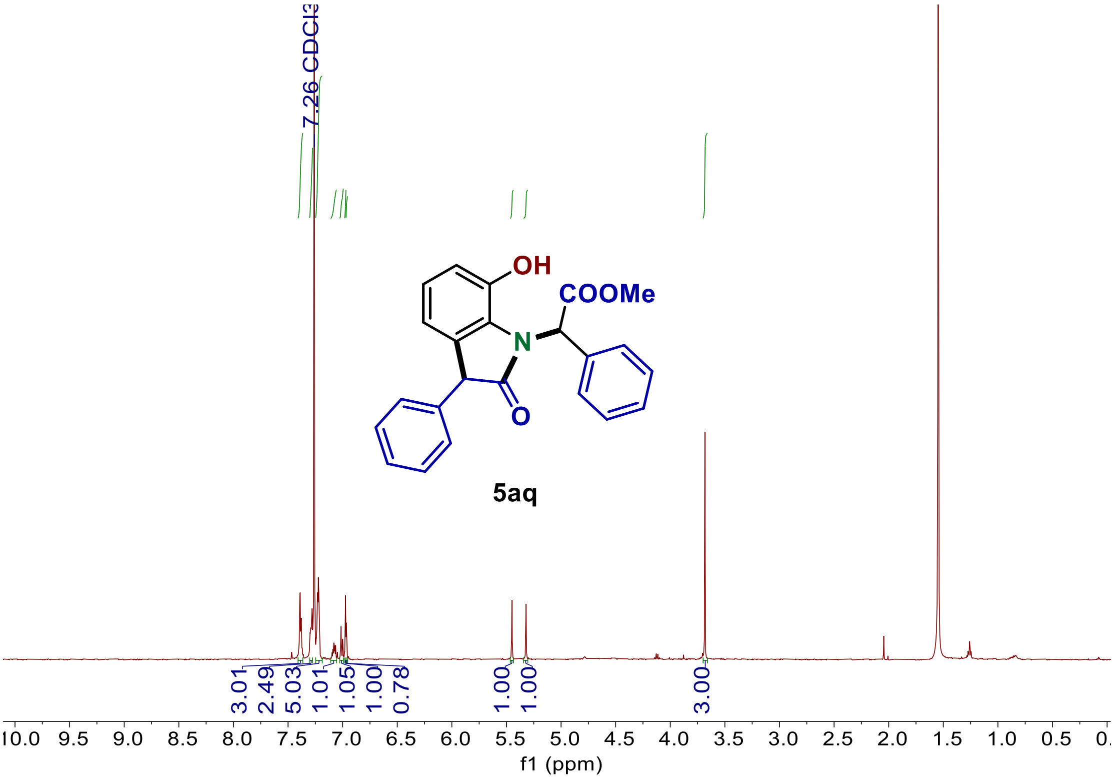

Figure S145. ^{1}H-NMR spectra of 5aq.

Methyl (R)-2-phenyl-2-((4-vinylphenyl)amino)acetate (3ar)

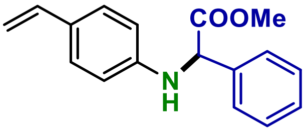

^{1}**H-NMR** (500 MHz, $CDCl_3$) δ p.p.m. 7.48 (dt, *J* = 6.3, 1.3 Hz, 2H), 7.38 – 7.33 (m, 2H), 7.33 – 7.29 (m, 1H), 7.19 (d, *J* = 8.5 Hz, 2H), 6.57 (dd, *J* = 17.6, 10.9 Hz, 1H), 6.51 (d, *J* = 8.5 Hz, 2H), 5.49 (dd, *J* = 17.6, 1.1 Hz, 1H), 5.09 (s, 1H), 5.00 (dd, *J* = 10.9, 1.1 Hz, 1H), 3.74 (s, 3H); ^{13}**C-NMR** (126 MHz, $CDCl_3$) δ 172.32, 145.72, 137.57, 136.62, 129.06, 128.52, 128.07, 127.47, 127.37, 113.48, 110.06, 60.75, 53.01; **HRMS (ESI)** calcd. for $[M+H]^+$: 268.1332 m/z, found: 268.1332 m/z.

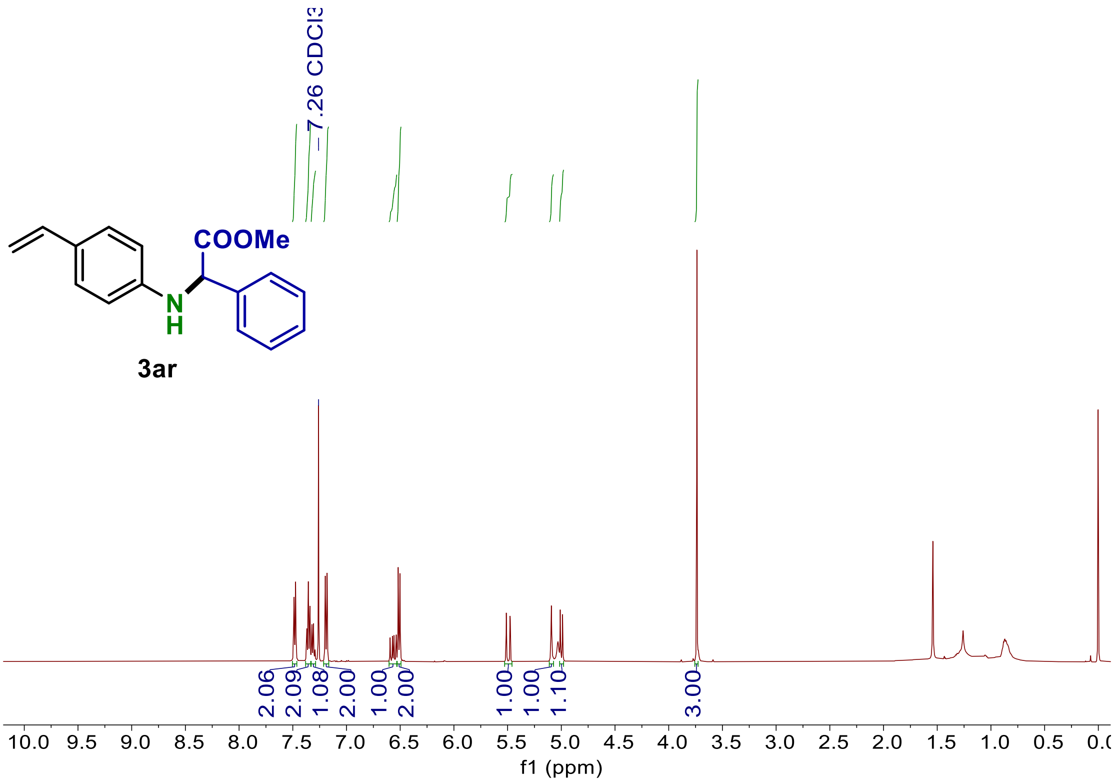

Figure S146. ^{1}H-NMR spectra of 3ar.

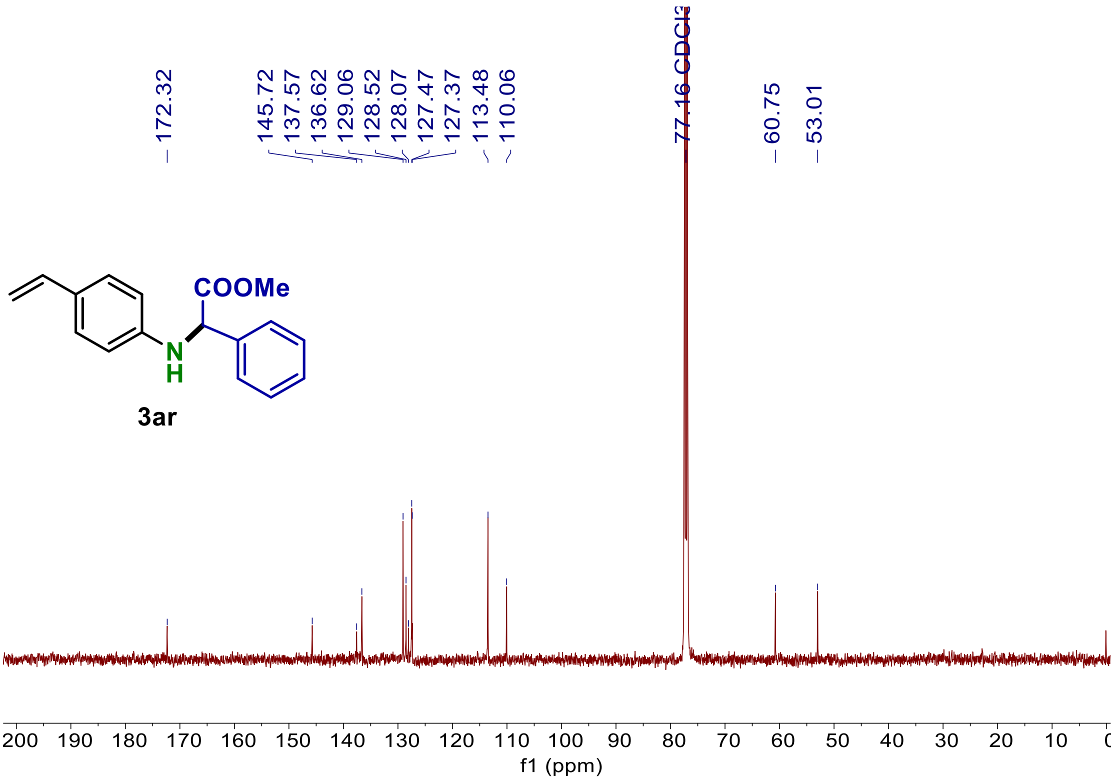

Figure S147. ^{13}C-NMR spectra of 3ar.

Methyl (2S)-2-(4-(((R)-2-methoxy-2-oxo-1-phenylethyl)amino)phenyl)-1-phenylcyclopropane-1-carboxylate (4ar)

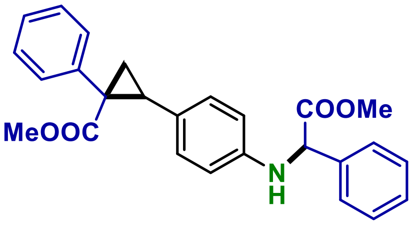

^{1}H-NMR (500 MHz, $CDCl_3$) δ p.p.m. 7.40 (d, J = 7.3 Hz, 2H), 7.34 – 7.28 (m, 3H), 7.12 (m, 3H), 7.03-6.95 (m, 2H), 6.51 (d, J = 8.2 Hz, 2H), 6.27 (d, J = 8.2 Hz, 2H), 4.97 (d, J = 9.7 Hz, 1H), 3.69 (d, J = 4.7 Hz, 3H), 3.63 (d, J = 1.8 Hz, 3H), 2.98-2.92 (m, 1H), 2.07-2.03 (m, 1H), 1.75 – 1.69 (m, 1H); **HRMS (ESI)** calcd. for [M+H]+: 416.1856 m/z, found: 416.1863 m/z.

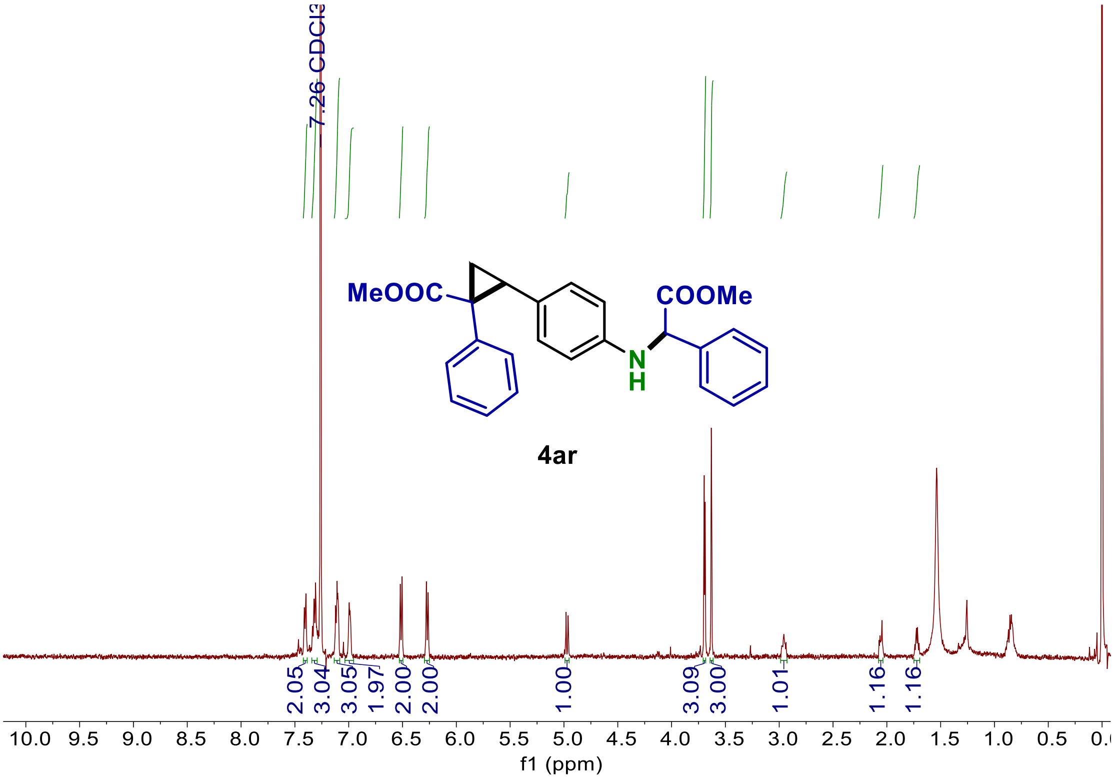

Figure S148. ^{1}H-NMR spectra of 4ar.

Methyl (S)-2-(((R)-2-methoxy-2-oxo-1-phenylethyl)(4-vinylphenyl)amino)-2-phenylacetate (5ar)

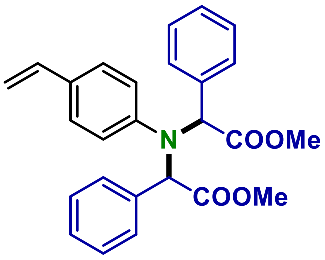

^{1}H-NMR (500 MHz, $CDCl_3$) δ p.p.m. 7.36 (d, J = 6.9 Hz, 4H), 7.30-7.26 (m, 6H), 7.11 (d, J = 8.6 Hz, 2H), 6.76 (d, J = 8.6 Hz, 2H), 6.56 (dd, J = 17.6, 10.8 Hz, 1H), 5.53 (d, J = 17.6 Hz, 1H), 5.47 (s, 2H), 5.05 (d, J = 11.1 Hz, 1H), 3.57 (s, 6H); **HRMS (ESI)** calcd. for $[M+H]^+$: 416.1856 m/z, found: 416.1864 m/z.

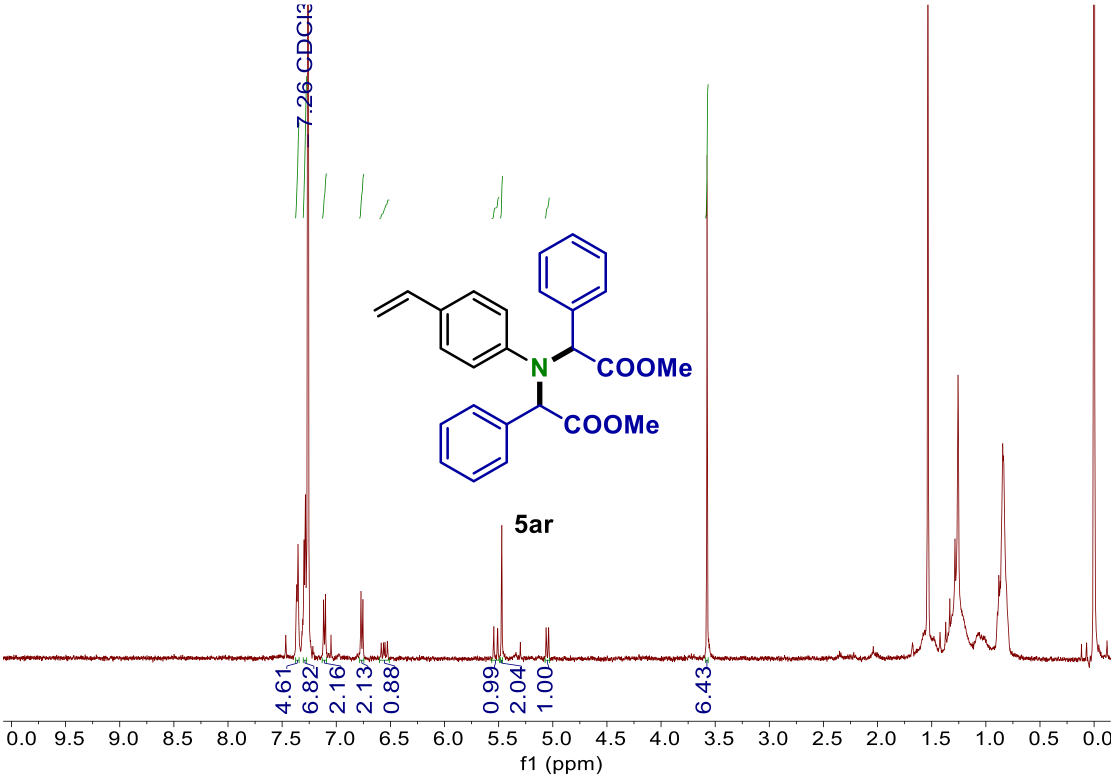

Figure S149. ^{1}H-NMR spectra of 5ar.

Methyl (2S)-2-(4-(((R)-2-methoxy-2-oxo-1-phenylethyl)((S)-2-methoxy-2-oxo-1-phenylethyl)amino)phenyl)-1-phenylcyclopropane-1-carboxylate (6ar)

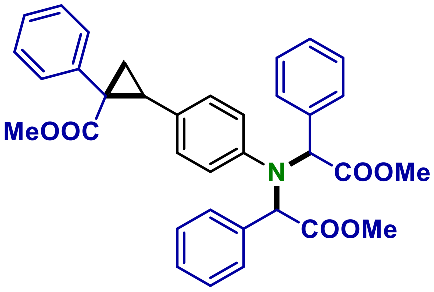

Isomer A: ^{1}**H-NMR** (500 MHz, $CDCl_3$) δ p.p.m 7.29-7.19 (m, 10H), 7.12-7.06 (m, 3H), 6.94 (d, *J* = 7.0 Hz, 2H), 6.55 (d, *J* = 8.3 Hz, 2H), 6.46 (d, *J* = 8.3 Hz, 2H), 5.29 (d, *J* = 12.2 Hz, 2H), 3.63 (s, 3H), 3.48 (d, *J* = 15.1 Hz, 6H), 2.96 (t, *J* = 8.4 Hz, 1H), 2.07 – 2.02 (m, 1H), 1.76 – 1.72 (m, 1H); **HRMS (ESI)** calcd. for $[M+H]^+$: 564.2381 m/z, found: 564.2390m/z.

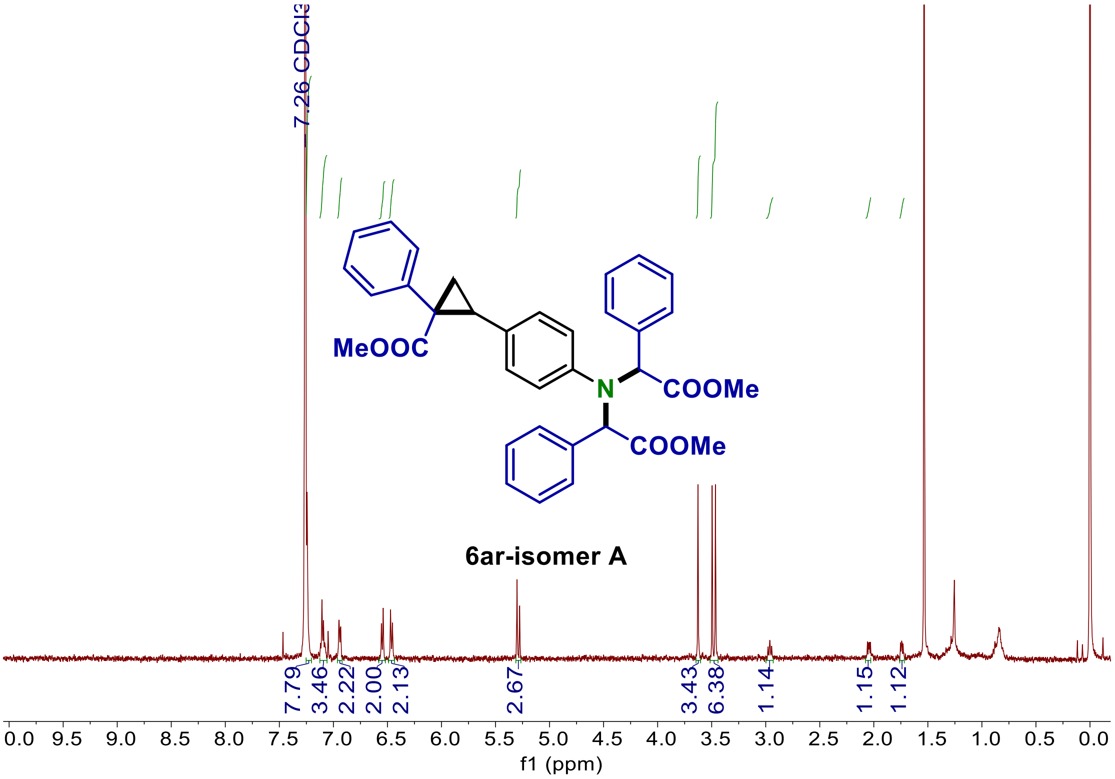

Figure S150. ^{1}H-NMR spectra of 6ar-isomer A.

Isomer B: ^{1}H-NMR (500 MHz, $CDCl_3$) δ p.p.m 7.24 – 7.17 (m, 6H), 7.16 – 7.07 (m, 7H), 6.96 (dd, *J* = 15.5, 6.4 Hz, 2H), 6.56 (t, *J* = 6.5 Hz, 2H), 6.48 (dd, *J* = 17.7, 8.4 Hz, 2H), 5.15 (d, *J* = 3.9 Hz, 2H), 3.63 (s, 3H), 3.60 (d, *J* = 7.5 Hz, 6H), 3.01 – 2.93 (m, 1H), 2.08 – 2.02 (m, 1H), 1.77 – 1.73 (m, 1H); **HRMS (ESI)** calcd. for $[M+H]^+$: 564.2381 m/z, found: 564.2393m/z.

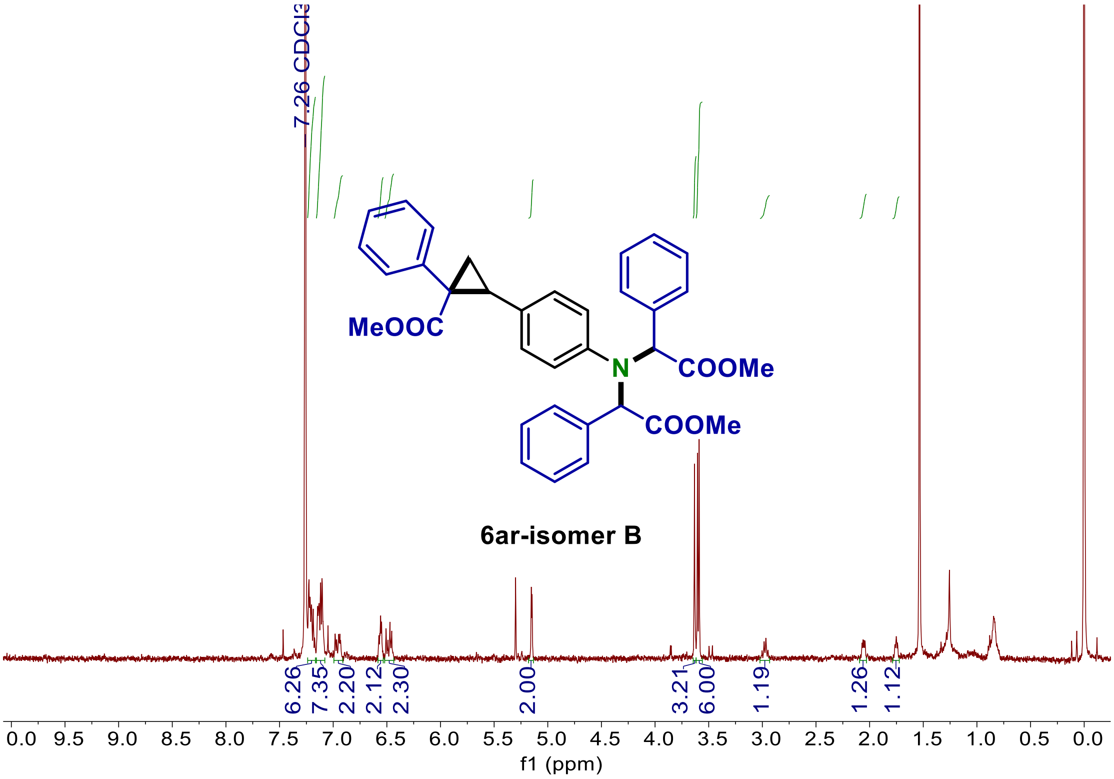

Figure S151. ^{1}H-NMR spectra of 6ar-isomer B.

Methyl (R)-2-((4-ethynylphenyl)amino)-2-phenylacetate (3as)

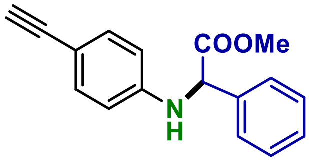

^{1}H-NMR (400 MHz, $CDCl_3$) δ p.p.m. 7.50 – 7.42 (m, 2H), 7.40 – 7.29 (m, 3H), 7.24 (d, *J* = 1.9 Hz, 2H), 6.46 (m, d, *J* = 8.6 Hz, 2H), 5.18 (d, *J* = 5.7 Hz, 1H), 5.08 (d, *J* = 5.8 Hz, 1H), 3.74 (s, 3H), 2.93 (s, 1H); **^{13}C-NMR** (126 MHz, $CDCl_3$) δ 172.06, 146.26, 137.18, 133.56, 129.13, 128.65, 127.32, 113.15, 111.07, 84.51, 75.09, 60.44, 53.11; **HRMS (ESI)** calcd. for $[M+H]^+$: 226.1176 m/z, found: 226.1177 m/z.

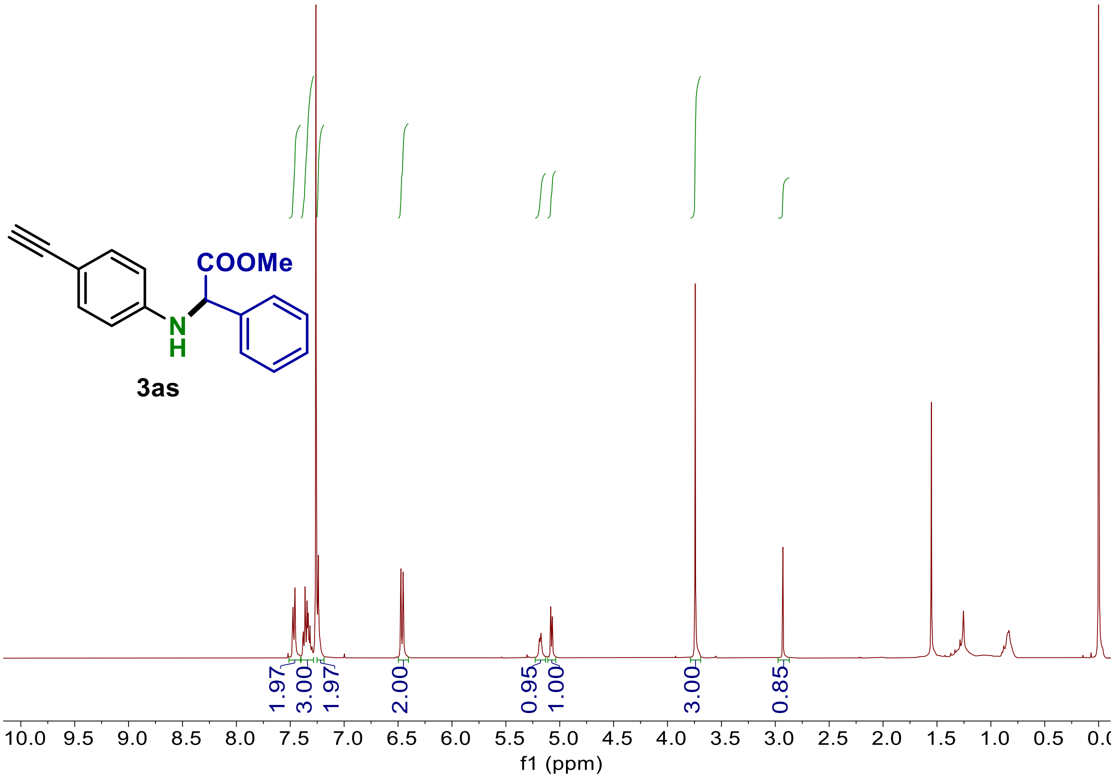

Figure S152. ^{1}H-NMR spectra of 3as.

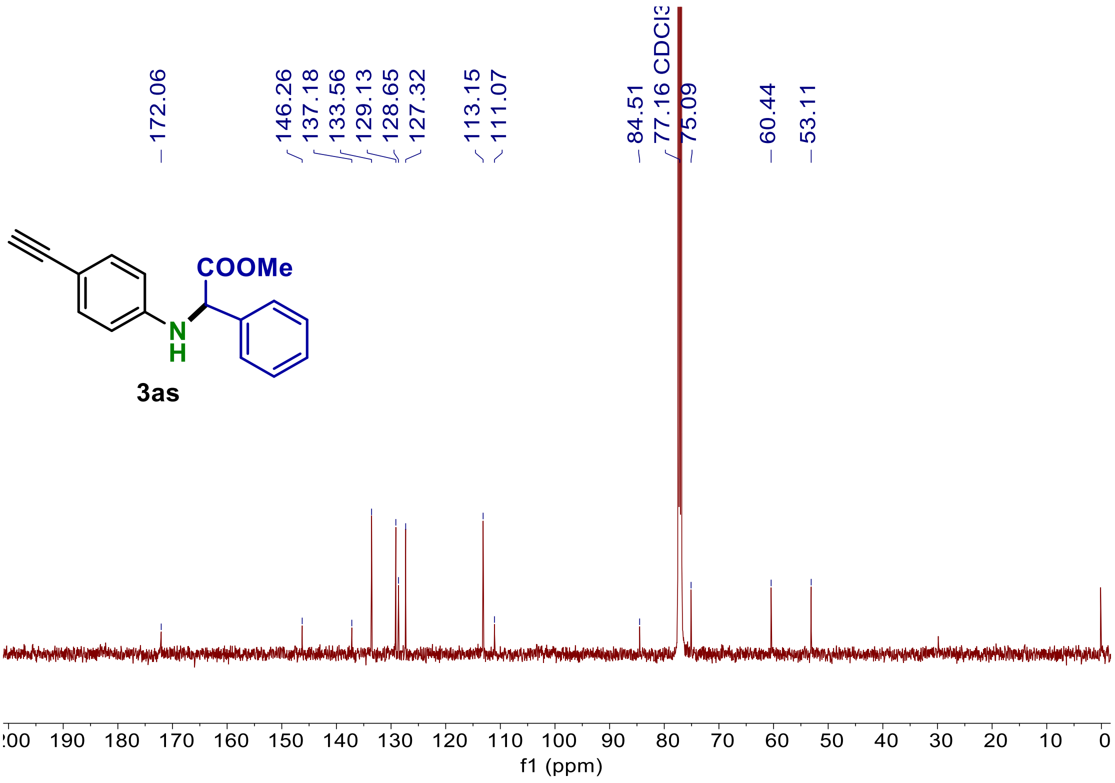

Figure S153. ^{13}C-NMR spectra of 3as.

Methyl 2-(4-(((R)-2-methoxy-2-oxo-1-phenylethyl)amino)phenyl)-1-phenylcycloprop-2-ene-1-carboxylate (4as)

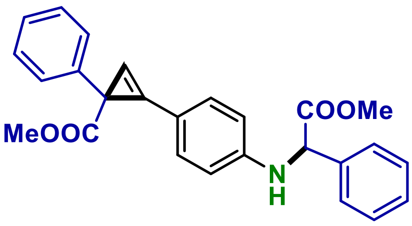

^{1}H-NMR (400 MHz, $CDCl_3$) δ p.p.m. 7.64 – 7.58 (m, 2H), 7.56 – 7.49 (m, 2H), 7.43 – 7.33 (m, 7H), 7.19 (t, J = 7.4 Hz, 1H), 6.64 (s, 1H), 6.59 (d, J = 8.6 Hz, 2H), 5.14 (d, J = 5.6 Hz, 1H), 5.08 (d, J = 6.0 Hz, 1H), 4.08 (s, 3H), 3.77 (s, 3H); **HRMS (ESI)** calcd. for $[M+H]^+$: 414.1700 m/z, found: 414.1697 m/z.

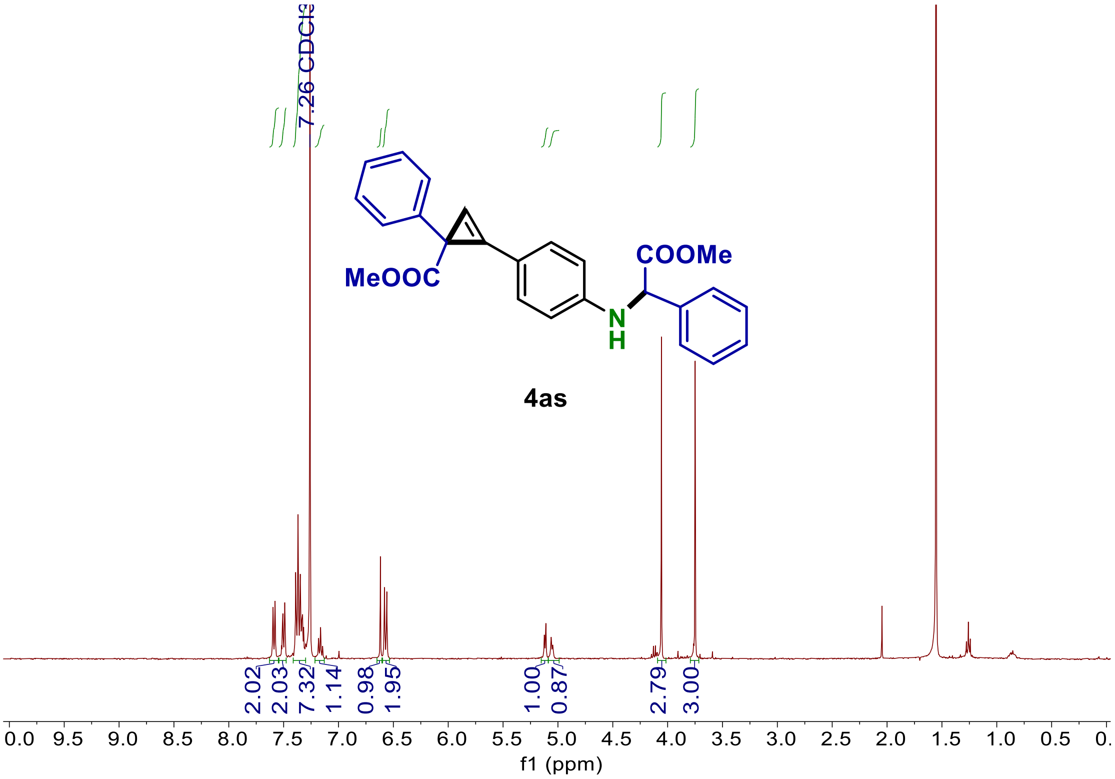

Figure S154. ^{1}H-NMR spectra of 4as.

Methyl (S)-2-((4-ethynylphenyl)((R)-2-methoxy-2-oxo-1-phenylethyl)amino)-2-phenylacetate (5as)

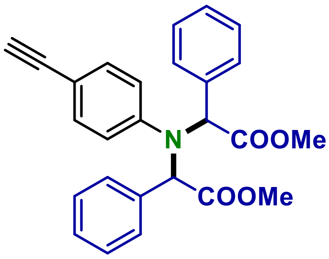

^{1}H-NMR (400 MHz, $CDCl_3$) δ p.p.m. 7.37 – 7.27 (m, 10H), 7.18 (d, J = 8.8 Hz, 2H), 6.74 – 6.67 (m, 2H), 5.52 (s, 2H), 3.59 (s, 6H), 2.95 (s, 1H).

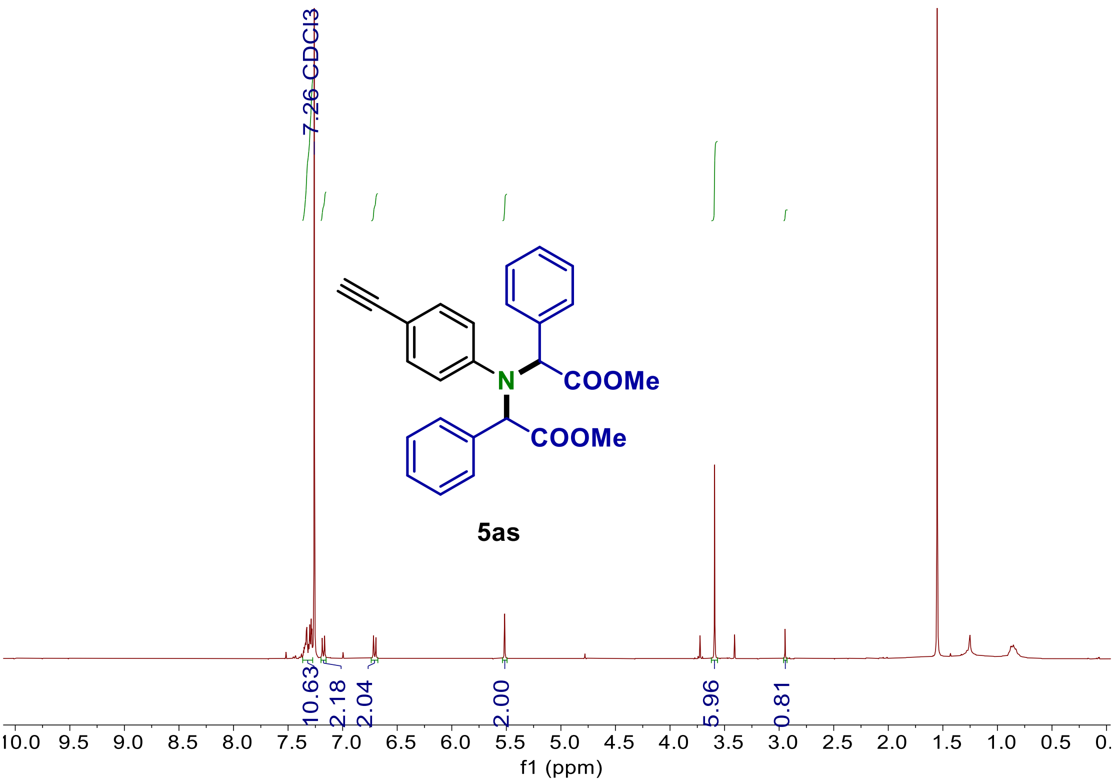

Figure S155. ^{1}H-NMR spectra of 5as.

Methyl (S)-3-(4-(((R)-2-methoxy-2-oxo-1-phenylethyl)amino)phenyl)-1H-indene-1-carboxylate (6as)

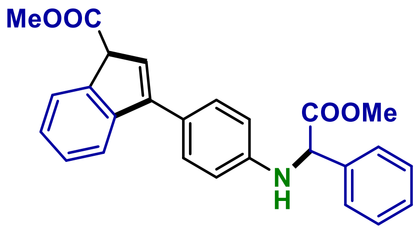

^{1}H-NMR (500 MHz, $CDCl_3$) δ p.p.m. 7.65 (d, J = 7.5 Hz, 1H), 7.52 (d, J = 7.5 Hz, 3H), 7.43 – 7.30 (m, 7H), 6.63 (d, J = 8.3 Hz, 2H), 6.40 (d, J = 2.4 Hz, 1H), 5.13 (s, 1H), 4.48 (s, 1H), 3.76 (s, 3H), 3.72 (s, 3H); **HRMS (ESI)** calcd. for $[M+H]^+$: 414.1700 m/z, found: 414.1696 m/z.

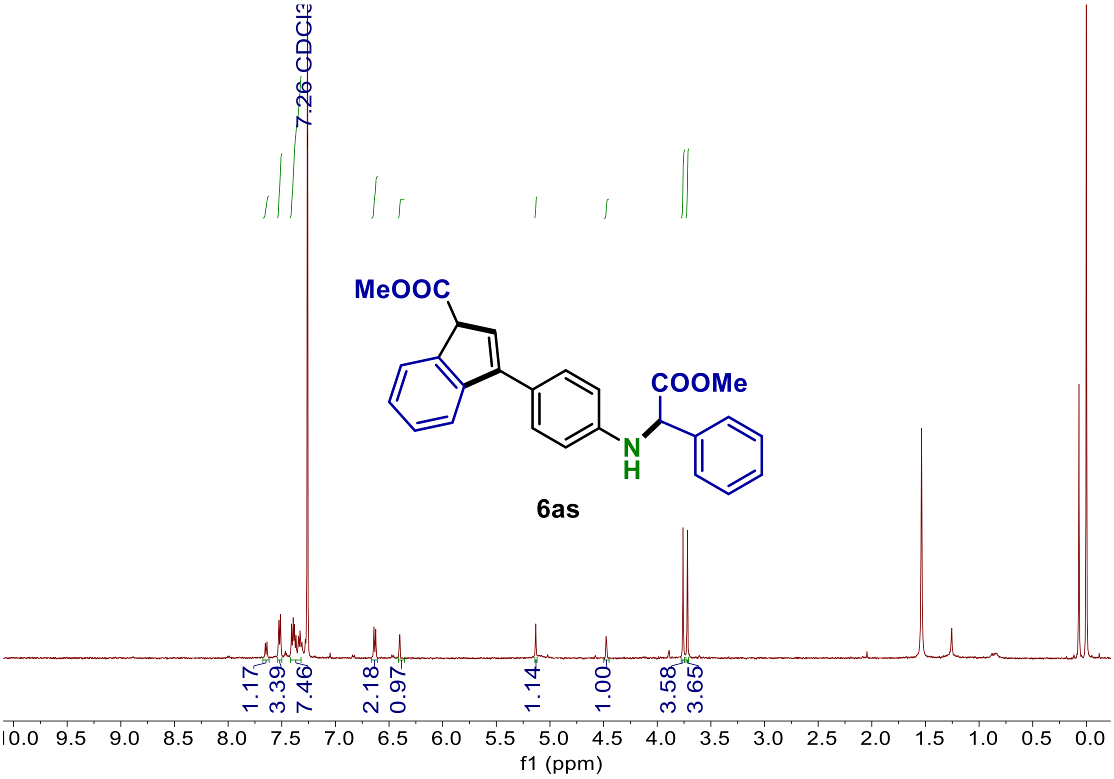

Figure S156. ^{1}H-NMR spectra of 6as.

Methyl (S)-3-(4-(((R)-2-methoxy-2-oxo-1-phenylethyl)((S)-2-methoxy-2-oxo-1-phenylethyl)amino)phenyl)-1H-indene-1-carboxylate (7as)

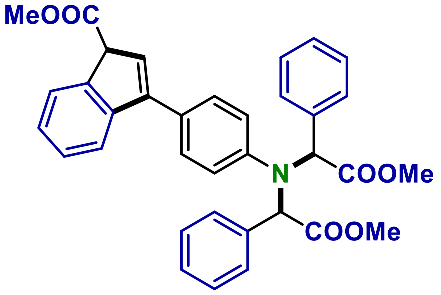

Isomer A: ^{1}**H-NMR** (500 MHz, $CDCl_3$) δ p.p.m. 7.64 (d, J = 7.4 Hz, 1H), 7.50 (d, J = 7.5 Hz, 1H), 7.40 (d, J = 7.3 Hz, 4H), 7.36 – 7.22 (m, 10H), 6.88 (d, J = 8.3 Hz, 2H), 6.42 (d, J = 2.3 Hz, 1H), 5.53 (d, J = 2.1 Hz, 2H), 4.47 (s, 1H), 3.72 (s, 3H), 3.60 (d, J = 3.1 Hz, 6H); **HRMS (ESI)** calcd. for $[M+H]^+$: 562.2224 m/z, found: 562.2223 m/z.

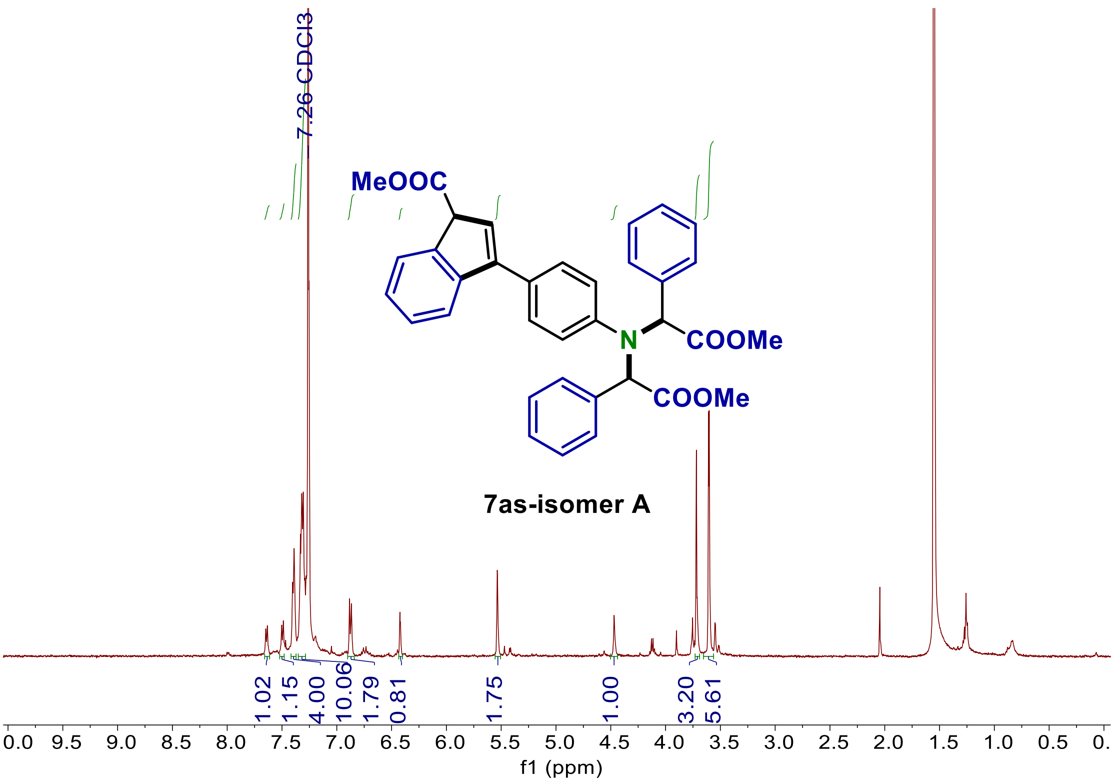

Figure S157. ^{1}H-NMR spectra of 7as-isomer A.

Isomer B: ^{1}H-NMR (500 MHz, $CDCl_3$) δ p.p.m. 7.65 (d, *J* = 7.3 Hz, 1H), 7.50 (d, *J* = 7.6 Hz, 1H), 7.39-7.15 (s, 14H), 6.92 (d, *J* = 8.1 Hz, 2H), 6.44 (d, *J* = 2.3 Hz, 1H), 5.47 (s, 2H), 4.48 (s, 1H); 3.75 (s, 6H), 3.72 (s, 3H).**HRMS (ESI)** calcd. for $[M+H]^+$: 562.2224 m/z, found: 562.2223 m/z.

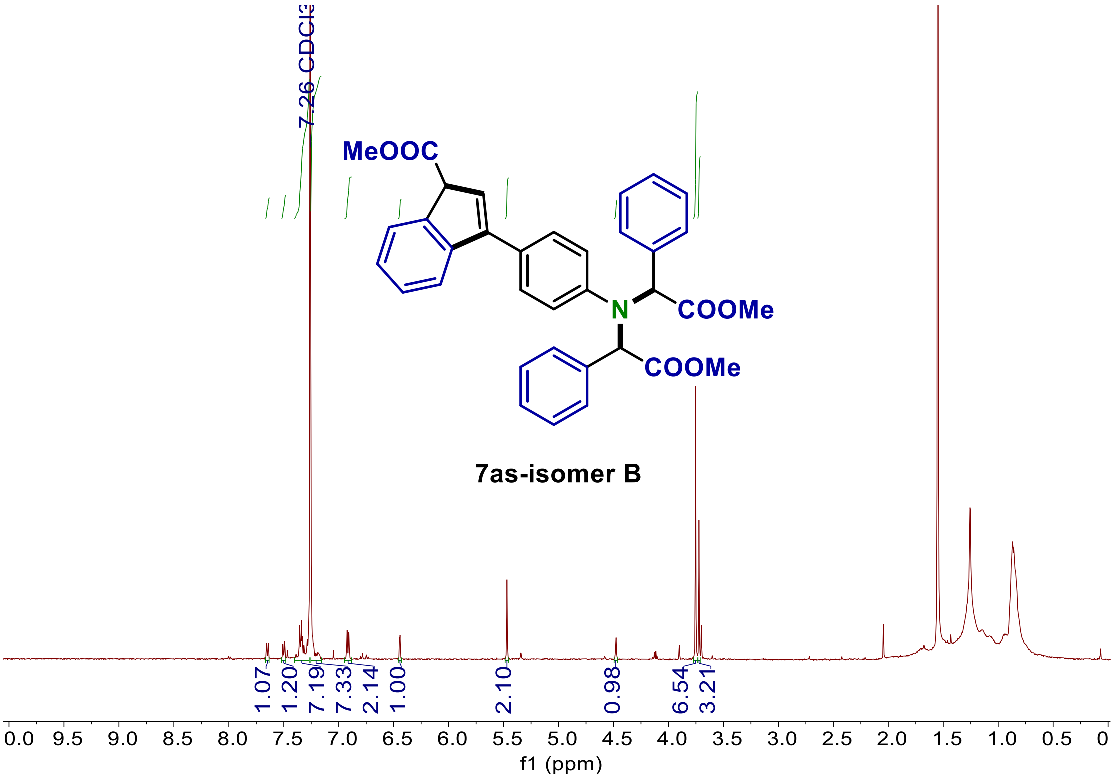

Figure S158. ^{1}H-NMR spectra of 7as-isomer B.

Methyl (R)-2-(benzo[d]thiazol-5-ylamino)-2-phenylacetate (3at)

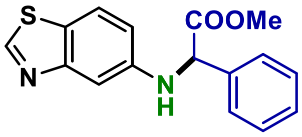

^{1}H-NMR (500 MHz, $CDCl_3$) δ p.p.m. 8.86 (s, 1H), 7.66 (d, *J* = 8.7 Hz, 1H), 7.55 – 7.51 (m, 2H), 7.36 (t, *J* = 7.6 Hz, 2H), 7.33 – 7.29 (m, 1H), 7.16 (d, *J* = 2.3 Hz, 1H), 6.86 (dd, *J* = 8.7, 2.3 Hz, 1H), 5.16 (s, 1H), 3.76 (s, 3H); **^{13}C-NMR** (126 MHz, $CDCl_3$) δ 172.30, 154.58, 145.26, 137.24, 129.14, 128.64, 127.46, 123.18, 122.19, 115.37, 105.60, 61.01, 53.08, 29.86; **HRMS (ESI)** calcd. for $[M+H]^+$: 299.0849 m/z, found: 299.0849 m/z.

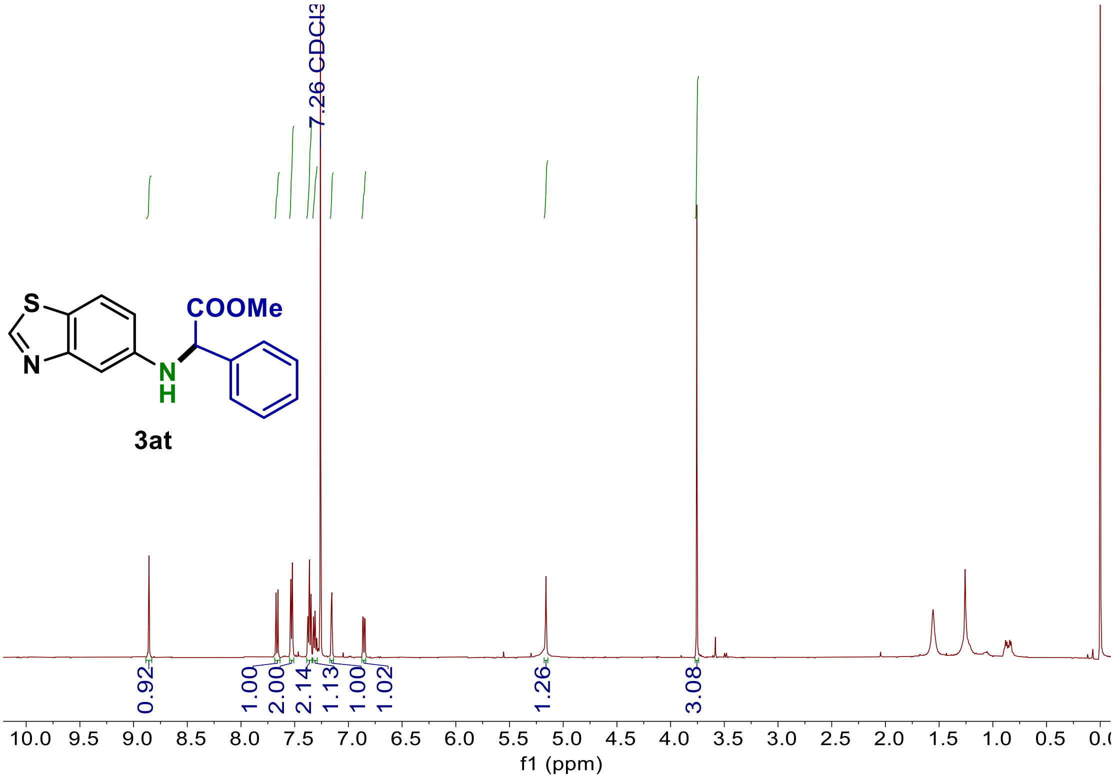

Figure S159. ^{1}H-NMR spectra of 3at.

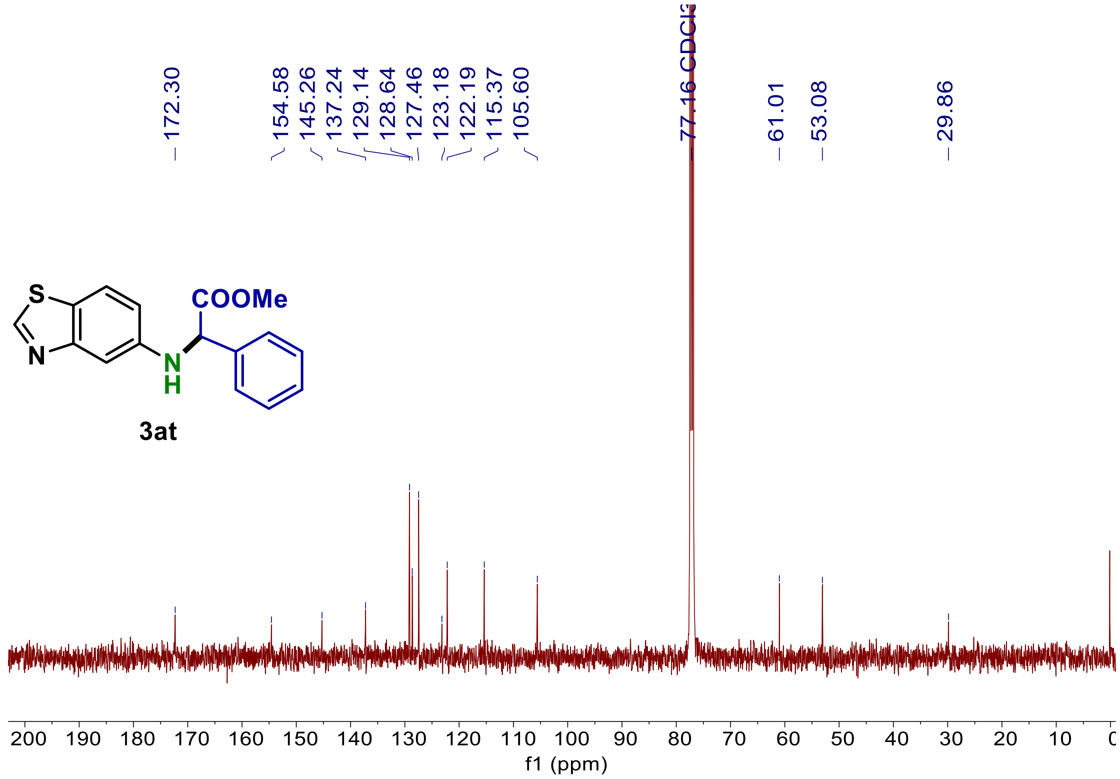

Figure S160. ^{13}C-NMR spectra of 3at.

Methyl (R)-2-((1H-indol-5-yl)amino)-2-phenylacetate (3au)

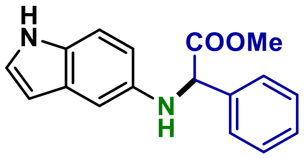

^{1}H-NMR (500 MHz, $CDCl_3$) δ p.p.m. 7.54 (d, J = 7.2 Hz, 2H), 7.38 – 7.34 (m, 2H), 7.33 – 7.28 (m, 1H), 7.19 (d, J = 8.6 Hz, 1H), 7.09 (t, J = 2.8 Hz, 1H), 6.74 (d, J = 2.2 Hz, 1H), 6.66 (dd, J = 8.6, 2.3 Hz, 1H), 6.34 (ddd, J = 3.2, 2.0, 1.0 Hz, 1H), 5.13 (s, 1H), 3.73 (s, 3H); **^{13}C-NMR** (126 MHz, $CDCl_3$) δ 173.05, 140.30, 138.24, 130.51, 128.96, 128.77, 128.31, 127.49, 124.60, 112.65, 111.84, 103.42, 102.07, 62.31, 52.80; **HRMS (ESI)** calcd. for $[M+H]^+$: 281.1285 m/z, found: 281.1285 m/z.

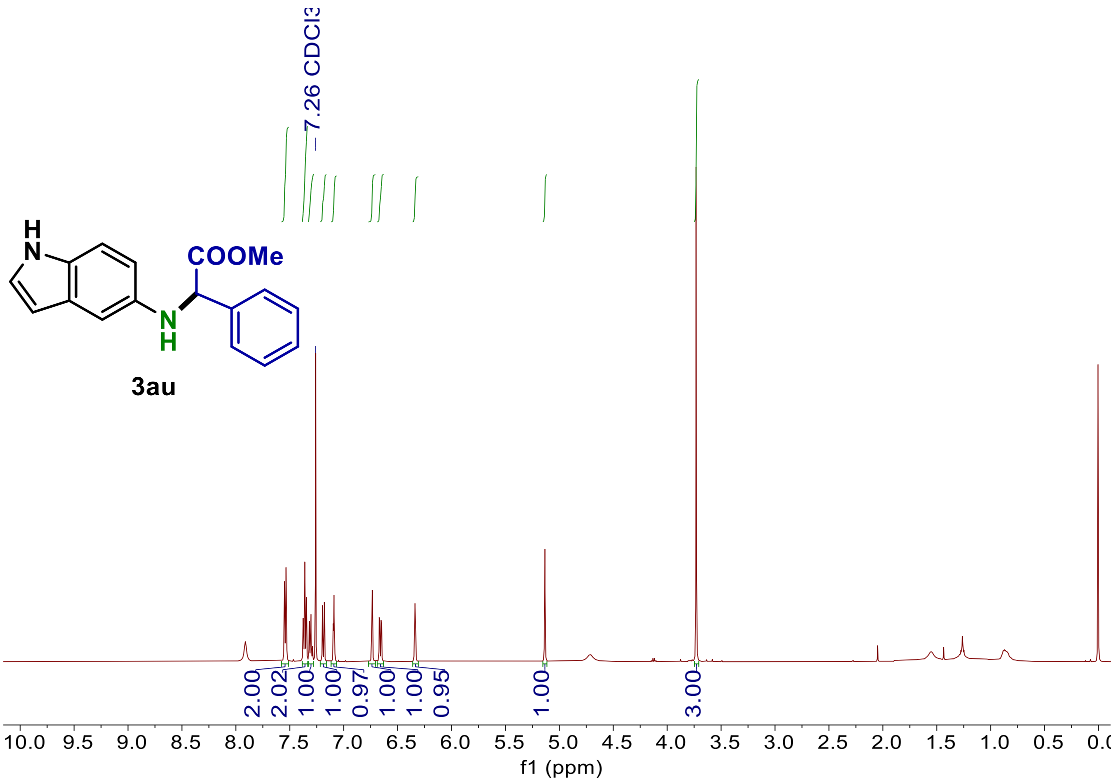

Figure S161. ^{1}H-NMR spectra of 3au.

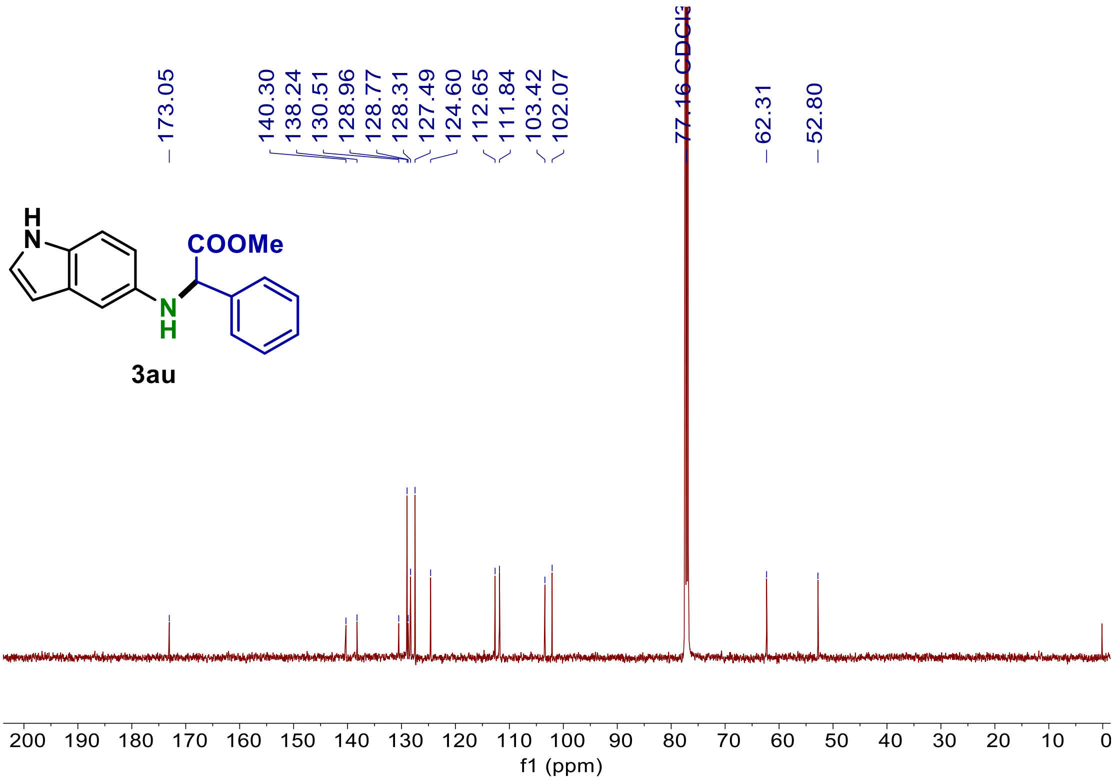

Figure S162. ^{13}C-NMR spectra of 3au.

Methyl (R)-2-((1-((S)-2-methoxy-2-oxo-1-phenylethyl)-1H-indol-5-yl)amino)-2-phenylacetate (4au)

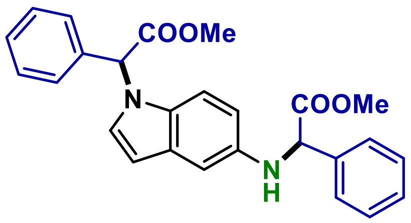

^{1}H-NMR (500 MHz, $CDCl_3$) δ p.p.m. 7.53 (d, *J* = 7.6 Hz, 2H), 7.39 – 7.28 (m, 8H), 7.11 (d, *J* = 8.7 Hz, 1H), 6.99 (t, *J* = 2.7 Hz, 1H), 6.71 (d, *J* = 2.3 Hz, 1H), 6.65 (d, *J* = 8.7 Hz, 1H), 6.30 (d, *J* = 3.2 Hz, 1H), 6.12 (s, 1H), 5.11 (s, 1H), 3.78 (s, 3H), 3.73 (s, 3H); **HRMS (ESI)** calcd. for $[M+H]^+$: 429.1809 m/z, found: 429.1813 m/z.

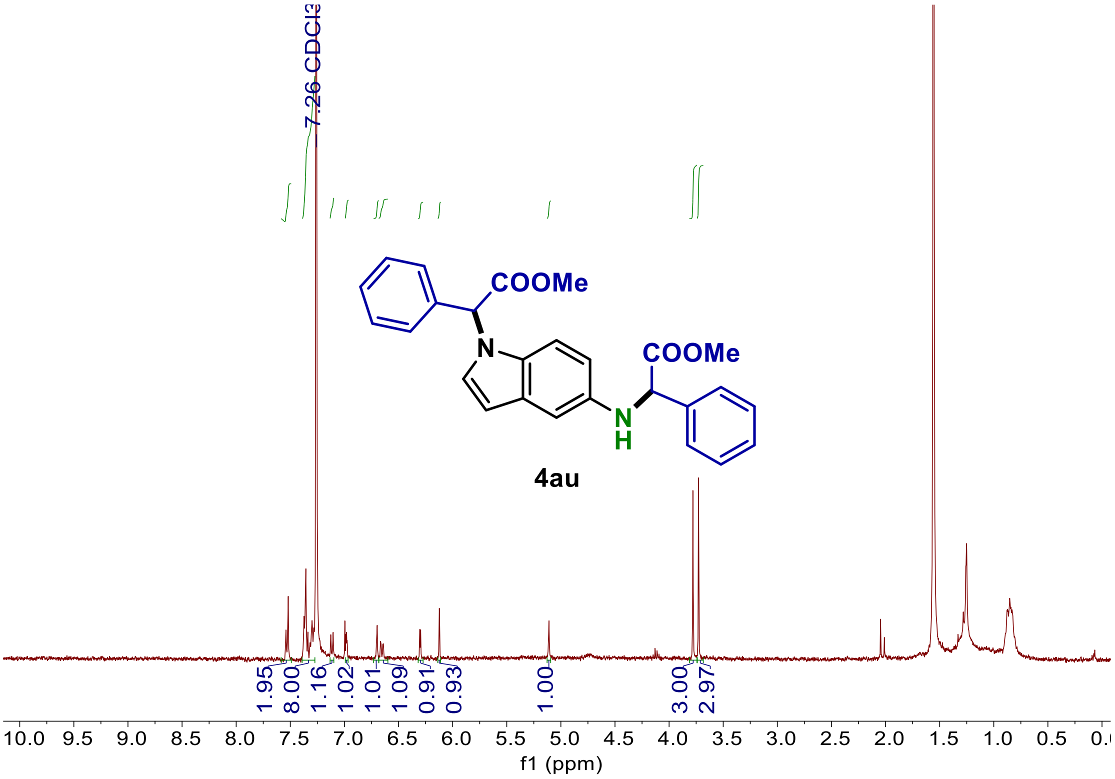

Figure S163. ^{1}H-NMR spectra of 4au.

Methyl (R)-2-((2-((S)-2-methoxy-2-oxo-1-phenylethyl)-1H-indol-5-yl)amino)-2-phenylacetate (5au)

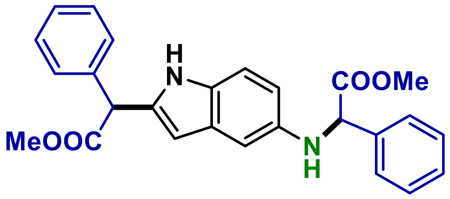

^{1}H-NMR (400 MHz, $CDCl_3$) δ p.p.m. 8.48 (s, 1H), 7.54 – 7.49 (m, 2H), 7.37 – 7.26 (m, 8H), 7.16 – 7.11 (m, 1H), 6.64 – 6.58 (m, 2H), 6.17 (t, *J* = 1.5 Hz, 1H), 5.14 (s, 1H), 5.10 (s, 1H), 3.76 (d, *J* = 1.4 Hz, 3H), 3.72 (d, *J* = 0.8 Hz, 3H).

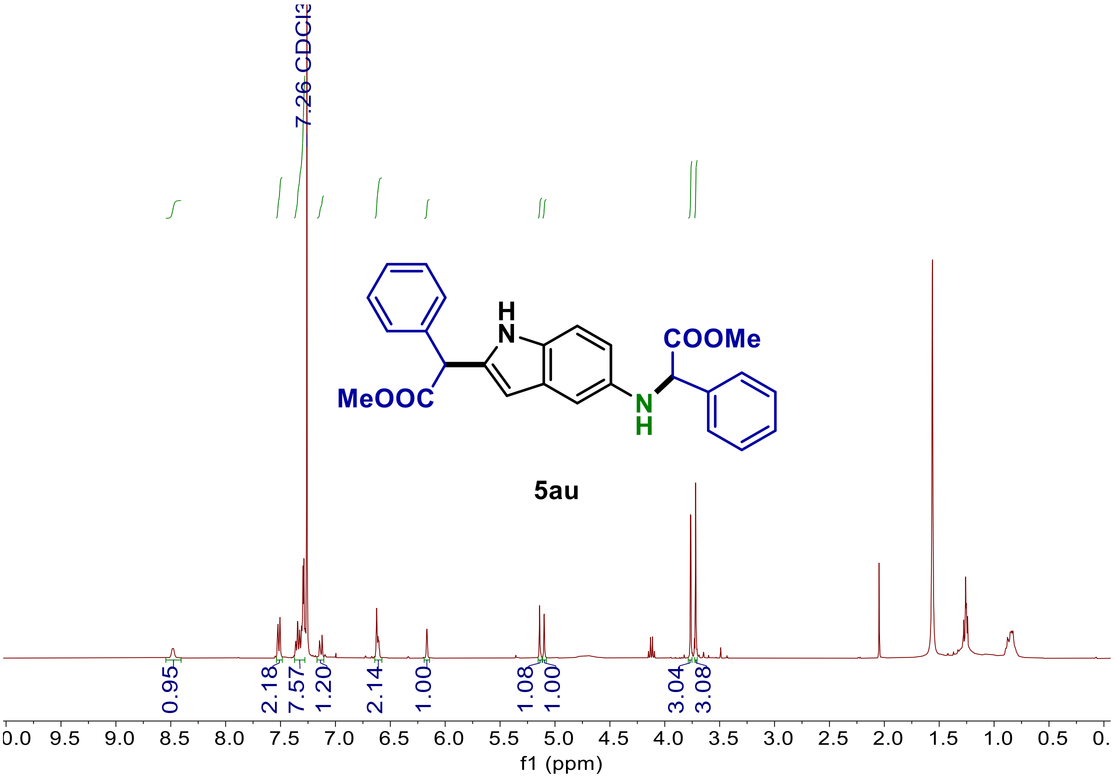

Figure S164. ^{1}H-NMR spectra of 5au.

Dimethyl 2,2'-(5-(((R)-2-methoxy-2-oxo-1-phenylethyl)amino)-1H-indole-1,3-diyl)(2S,2'S)-bis(2-phenylacetate) (6au)

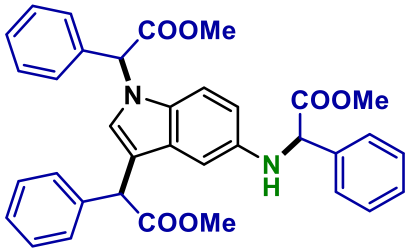

Isomers: ^{1}H-NMR (400 MHz, $CDCl_3$) δ p.p.m. δ 7.46 (ddq, *J* = 5.6, 2.9, 1.7 Hz, 2H), 7.40 – 7.16 (m, 13H), 7.11 – 7.02 (m, 2H), 6.59 (ddt, *J* = 8.4, 3.6, 2.2 Hz, 1H), 6.50 – 6.41 (m, 1H), 6.09 (s, 1H), 5.07 – 4.90 (m, 2H), 3.77 (d, *J* = 1.6 Hz, 3H), 3.71 – 3.59 (m, 6H). **HRMS (ESI)** calcd. for $[M+H]^+$: 577.2333 m/z, found: 577.2337 m/z.

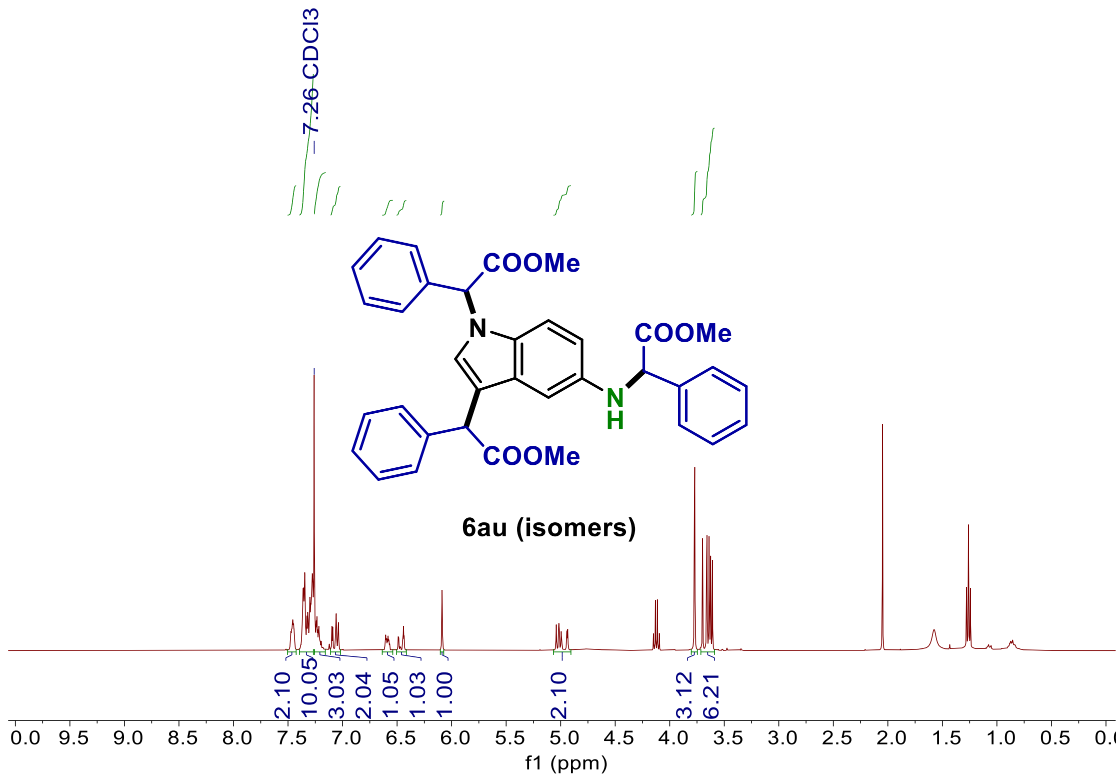

Figure S165. ^{1}H-NMR spectra of 6au.

Dimethyl 2,2'-((S)-7-oxo-8-phenyl-7,8-dihydropyrrolo[3,2-e]indole-1,6(3H)-diyl)(2R,2'R)-bis(2-phenylacetate) (7au)

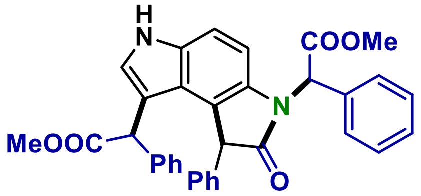

Isomers: ^{1}H-NMR (400 MHz, $CDCl_3$) δ p.p.m. δ 7.84 (s, 1H), 7.48 (t, J = 6.6 Hz, 2H), 7.39 – 7.18 (m, 13H), 7.14 (dd, J = 8.8 Hz, 1.7 Hz, 1H), 7.08 (dd, J = 13.4 Hz, 2.1 Hz, 1H), 6.64 – 6.59 (m, 1H), 6.49 (d, J = 17.6 Hz, 1H), 5.09 – 4.96 (m, 2H), 3.70 (d, J = 5.6 Hz, 3H), 3.66 (d, J = 6.3 Hz, 3H).

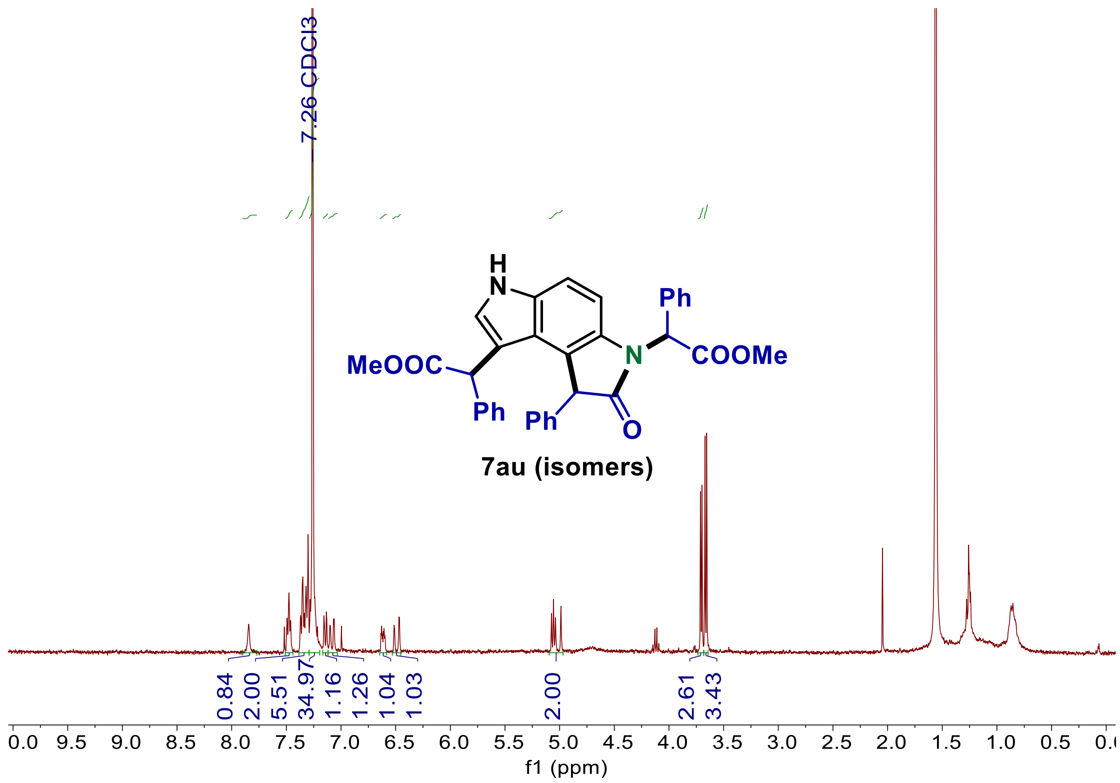

Figure S166. ^{1}H-NMR spectra of 7au.

Methyl (R)-2-((5-(hydroxymethyl)pyridin-3-yl)amino)-2-phenylacetate (3av)

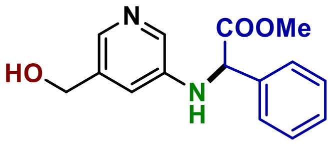

^{1}H-NMR (500 MHz, CDCl$_3$) δ p.p.m. 7.92 (s, 2H), 7.47 (d, *J* = 7.4 Hz, 2H), 7.35 (dt, *J* = 14.3, 7.2 Hz, 3H), 6.87 (s, 1H), 5.16 (s, 1H), 5.09 (d, *J* = 5.1 Hz, 1H), 4.58 (s, 2H), 3.75 (s, 3H); **^{13}C NMR** (126 MHz, CDCl$_3$) δ 171.88, 137.86, 136.68, 135.29, 131.07, 129.23, 128.99, 128.83, 127.34, 118.52, 62.84, 60.22, 53.23; **HRMS (ESI)** calcd. for [M+H]$^+$:273.1234 m/z, found: 273.1233 m/z.

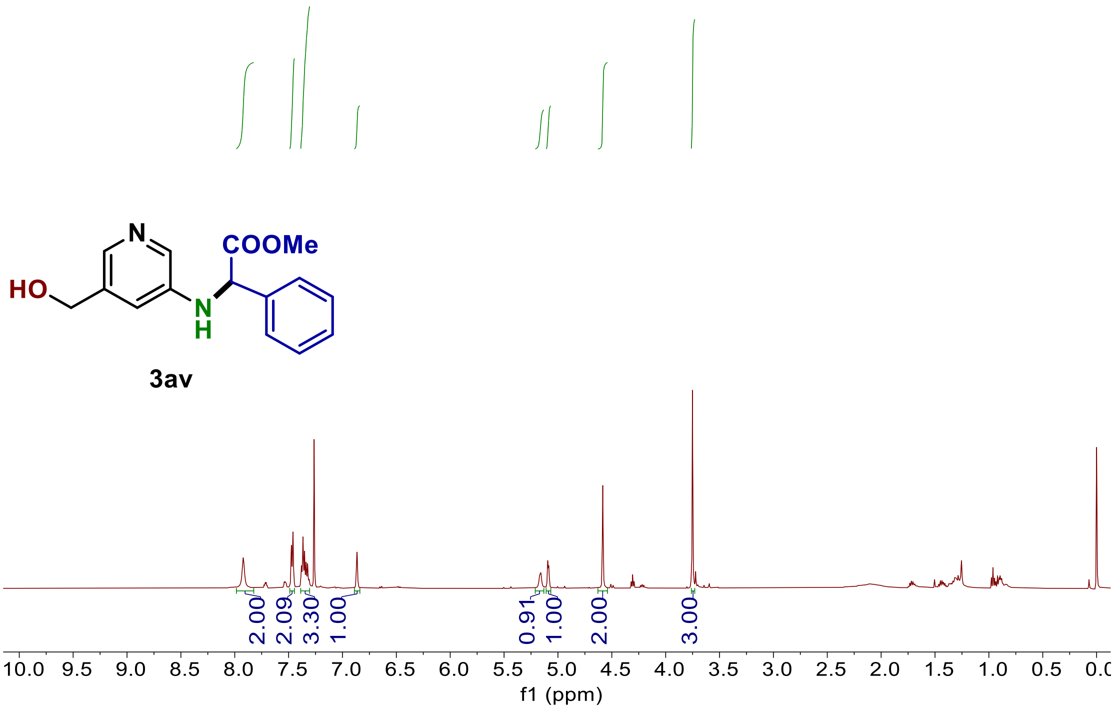

Figure S167. ^{1}H-NMR spectra of 3av.

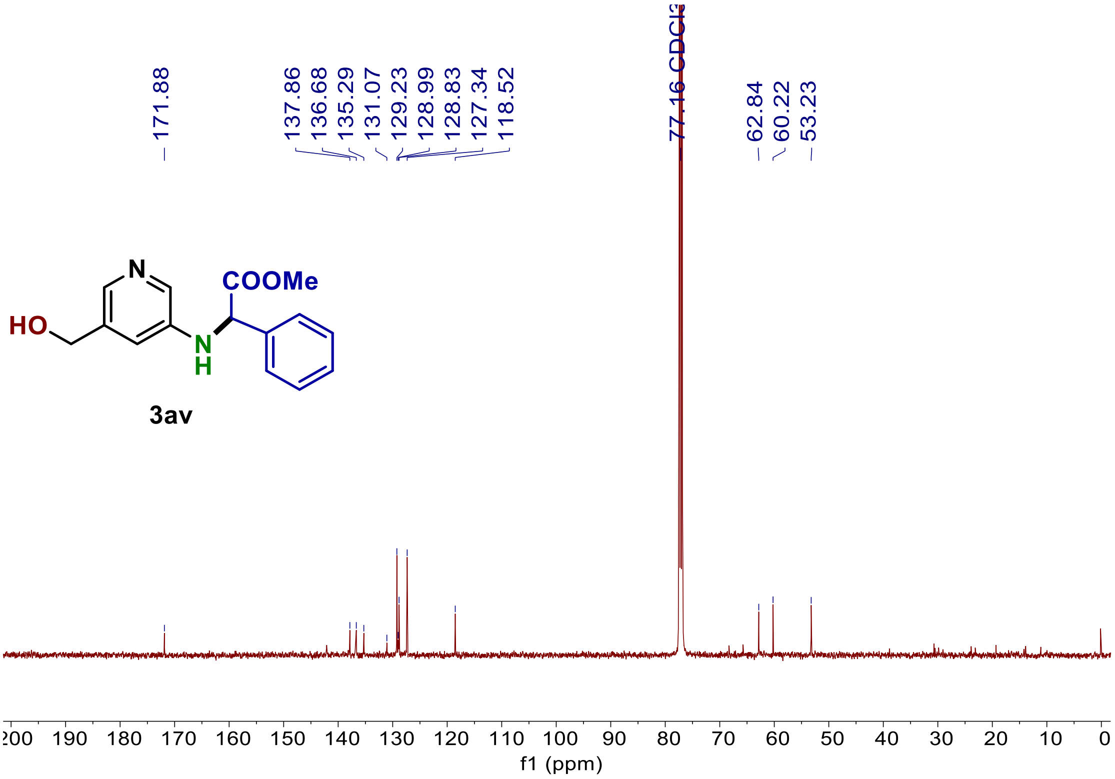

Figure S168. ^{13}C-NMR spectra of 3av.

Methyl (R)-2-(methyl(phenyl)amino)-2-phenylacetate (3aw)

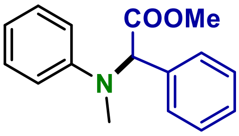

^{1}H-NMR (500 MHz, $CDCl_3$) δ p.p.m. 7.42 – 7.32 (m, 3H), 7.31 – 7.26 (m, 4H), 6.89 (d, J = 8.2 Hz, 2H), 6.82 (t, J = 7.2 Hz, 1H), 5.68 (s, 1H), 3.79 (s, 3H), 2.81 (s, 3H); **^{13}C-NMR** (126 MHz, $CDCl_3$) δ 172.48, 149.96, 135.93, 129.44, 128.81, 128.55, 128.26, 118.27, 113.62, 65.88, 52.15, 34.65; **HRMS (ESI)** calcd. for $[M+H]^+$: 256.1332 m/z, found: 256.1332 m/z.

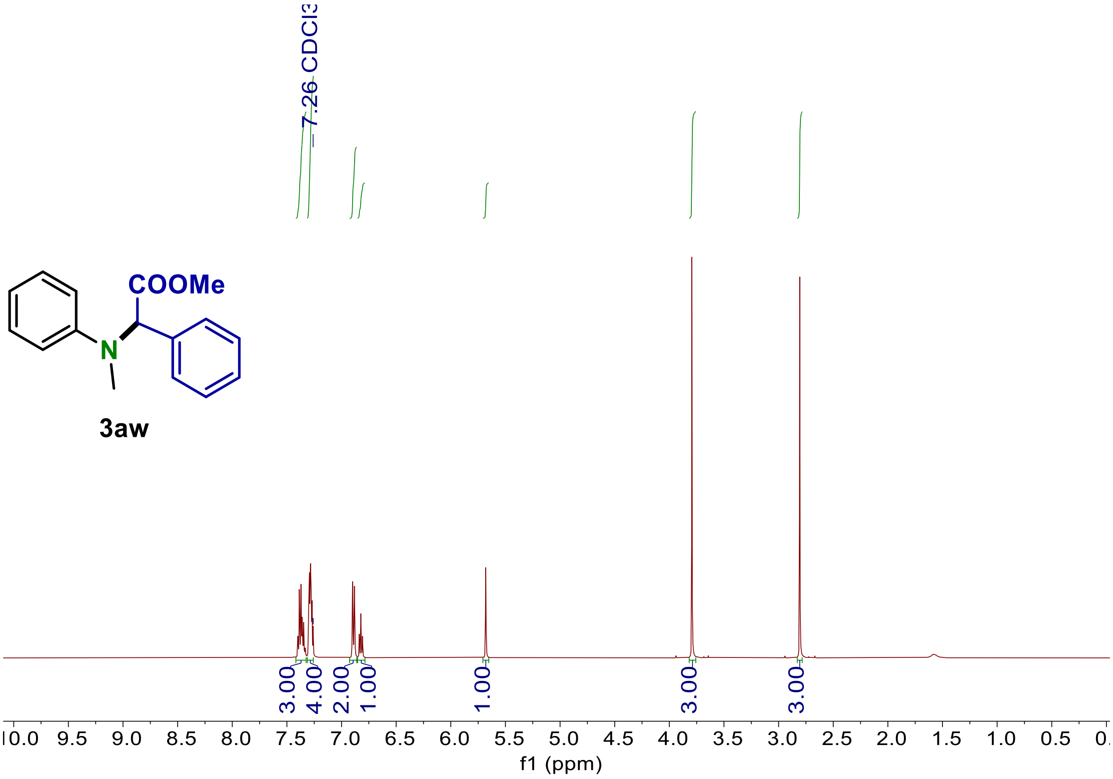

Figure S169. ^{1}H-NMR spectra of 3aw.

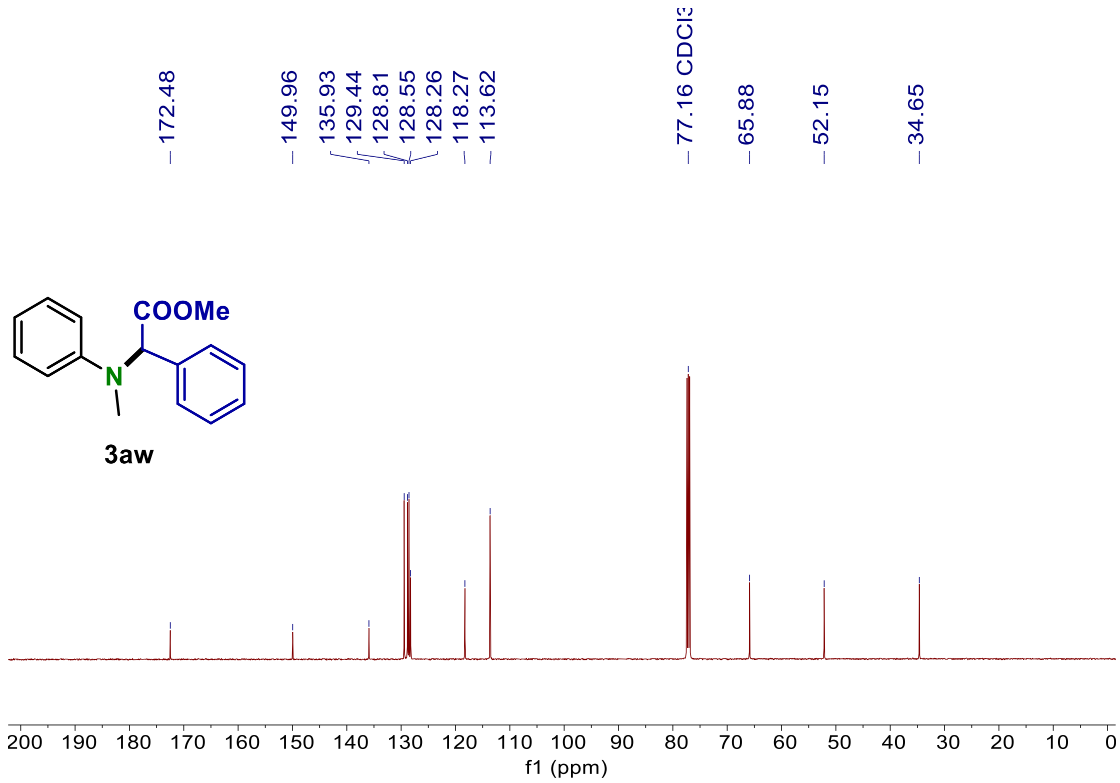

Figure S170. ^{13}C-NMR spectra of 3aw.

Methyl (R)-2-(4-(((R)-2-methoxy-2-oxo-1-phenylethyl)(methyl)amino)phenyl)-2-phenylacetate (4aw)

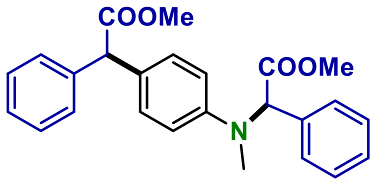

^{1}H-NMR (500 MHz, $CDCl_3$) δ p.p.m. 7.40 – 7.29 (m, 7H), 7.28 – 7.26 (m, 3H), 7.23 – 7.18 (m, 2H), 6.81 (d, *J* = 8.5 Hz, 2H), 5.64 (s, 1H), 4.96 (s, 1H), 3.78 (s, 3H), 3.74 (s, 3H), 2.78 (s, 3H); **^{13}C-NMR** (126 MHz, $CDCl_3$) δ 173.59, 172.44, 149.04, 139.32, 135.87, 129.63, 128.84, 128.68, 128.64, 128.52, 128.29, 127.95, 127.24, 113.33, 65.55, 56.28, 52.38, 52.18, 34.52; **HRMS (ESI)** calcd. for $[M+H]^+$: 404.1856 m/z, found: 404.1859 m/z.

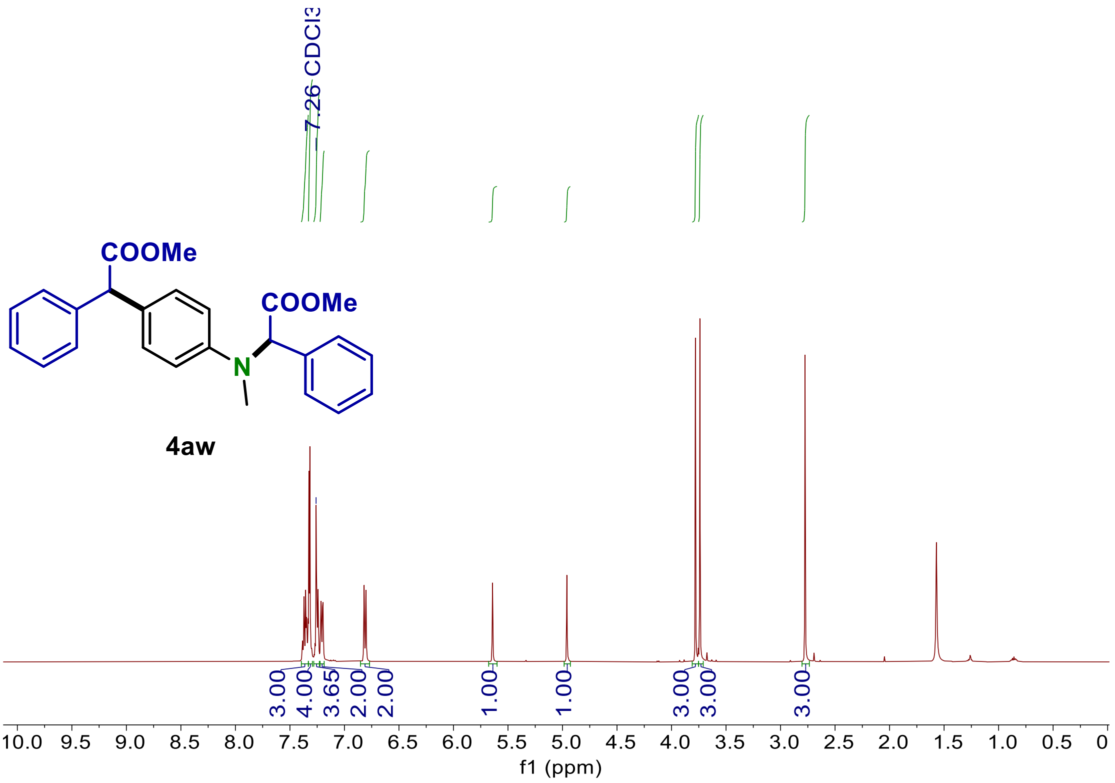

Figure S171. ^{1}H-NMR spectra of 4aw.

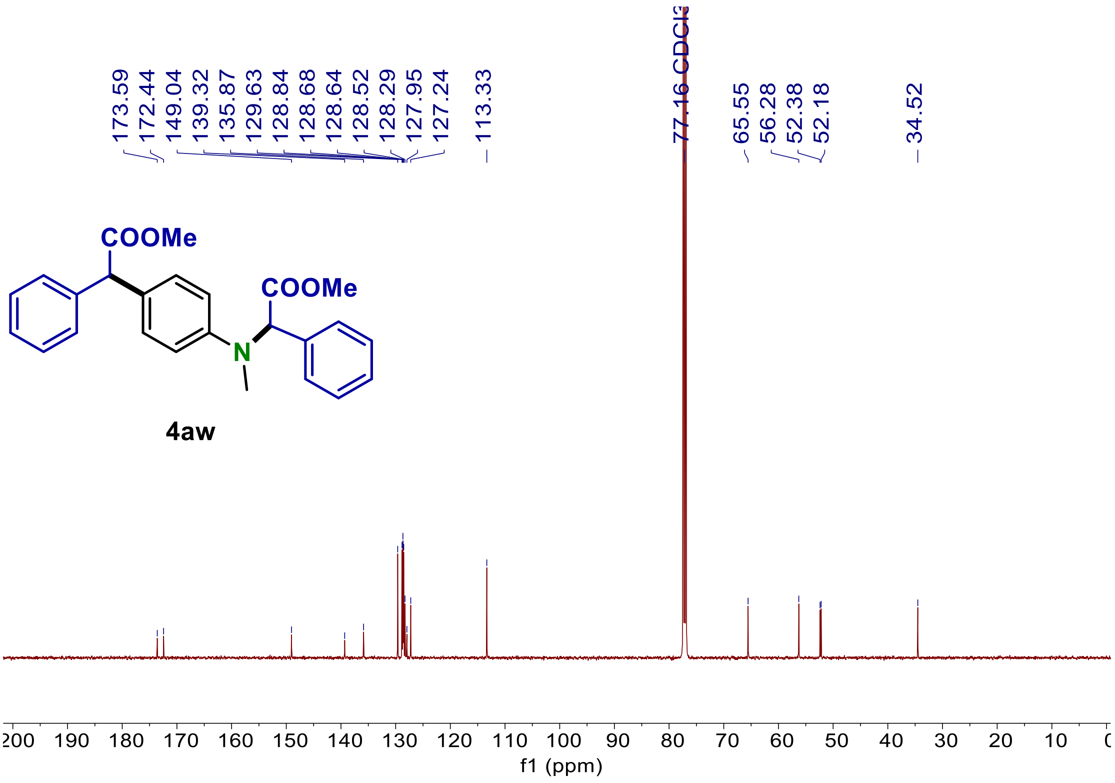

Figure S172. ^{13}C-NMR spectra of 4aw.

Methyl (R)-2-(diphenylamino)-2-phenylacetate (3ax)

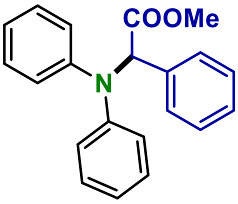

^{1}**H-NMR** (500 MHz, $CDCl_3$) δ p.p.m. 7.24 – 7.14 (m, 9H), 6.96 (tt, *J* = 7.3, 1.1 Hz, 2H), 6.91 – 6.85 (m, 4H), 5.79 (s, 1H), 3.77 (s, 3H); ^{13}**C-NMR** (126 MHz, $CDCl_3$) δ 172.62, 146.49, 135.20, 129.70, 129.11, 128.36, 128.17, 122.90, 122.42, 66.99, 52.48; **HRMS (ESI)** calcd. for $[M+H]^+$: 318.1489 m/z, found: 318.1489 m/z.

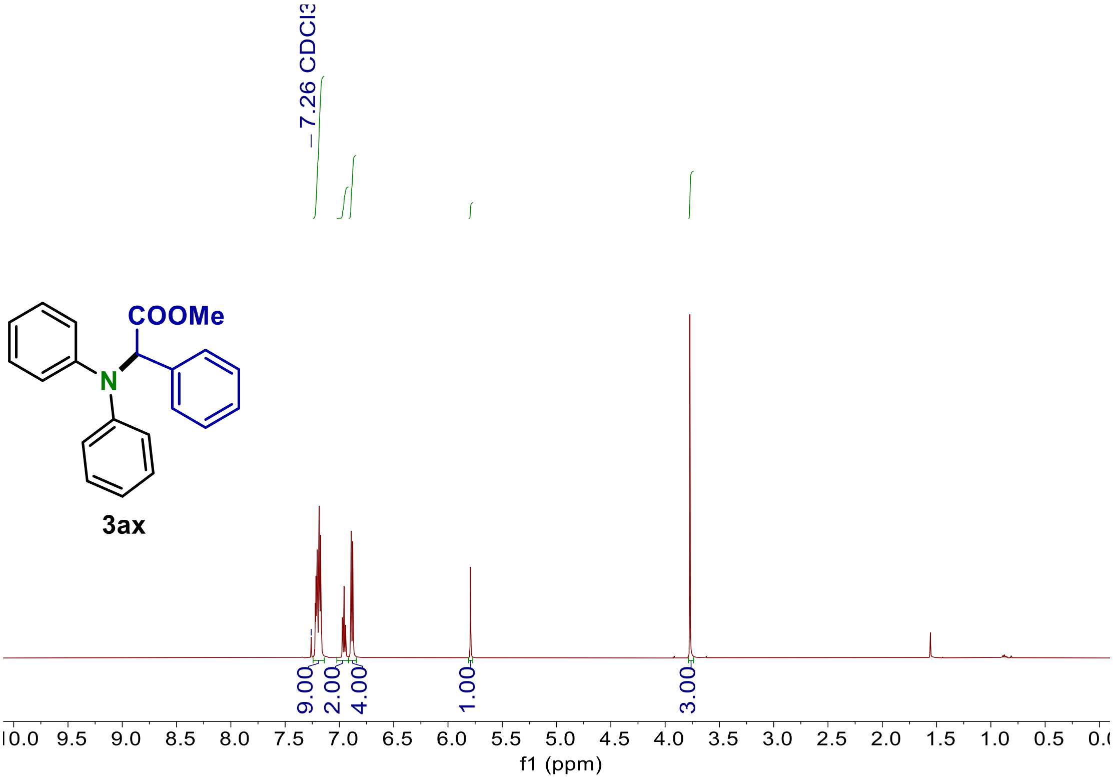

Figure S173. ^{1}H-NMR spectra of 3ax.

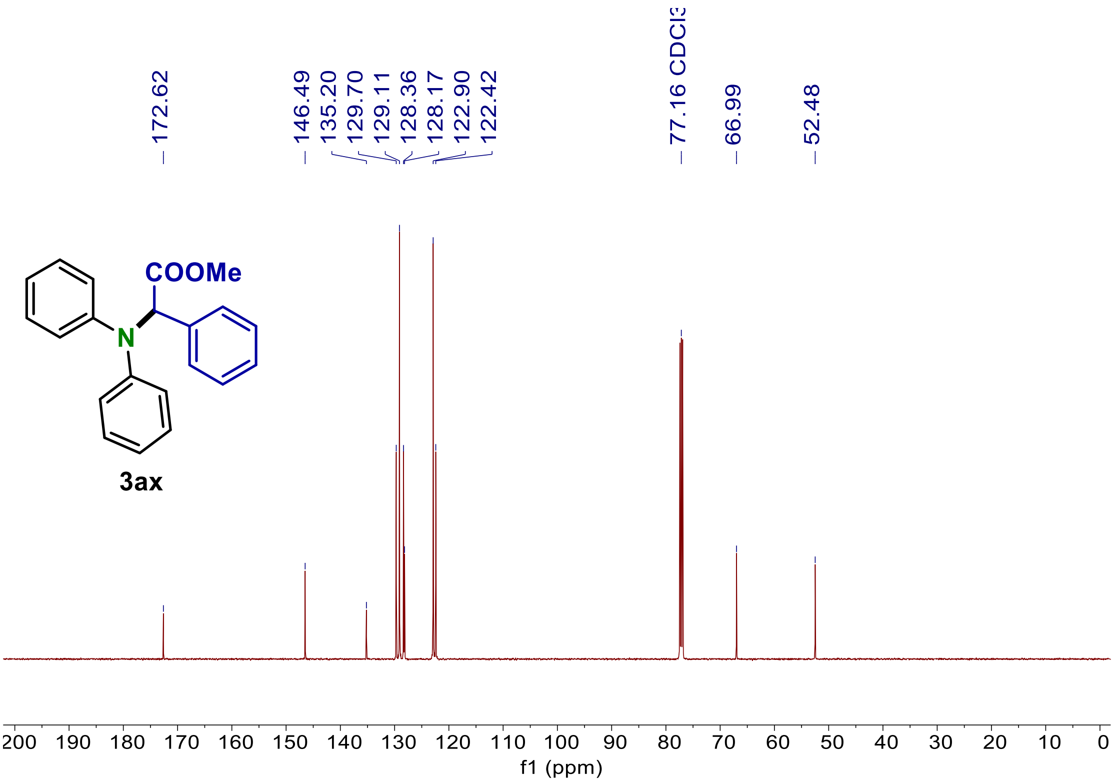

Figure S174. ^{13}C-NMR spectra of 3ax.

Methyl (R) -2-(4-(((R)-2-methoxy-2-oxo-1-phenylethyl)(phenyl)amino)phenyl)-2-phenylacetate (4ax)

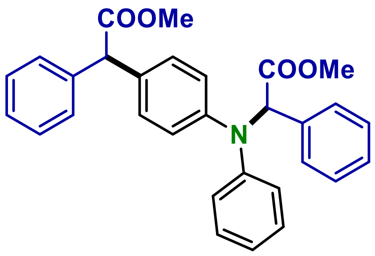

^{1}H-NMR (500 MHz, $CDCl_3$) δ p.p.m. 7.24 – 7.14 (m, 9H), 6.96 (tt, *J* = 7.3, 1.1 Hz, 2H), 6.91 – 6.85 (m, 4H), 5.79 (s, 1H), 3.77 (s, 3H); **^{13}C-NMR** (126 MHz, $CDCl_3$) δ 173.34, 172.57, 145.62, 135.09, 131.53, 129.74, 129.33, 129.11, 128.67, 128.37, 128.20, 127.30, 124.93, 124.67, 123.46, 123.33, 121.08, 120.80, 66.91, 56.39, 52.52, 52.41; **HRMS (ESI)** calcd. for $[M+H]^+$: 466.2013 m/z, found: 466.2017 m/z.

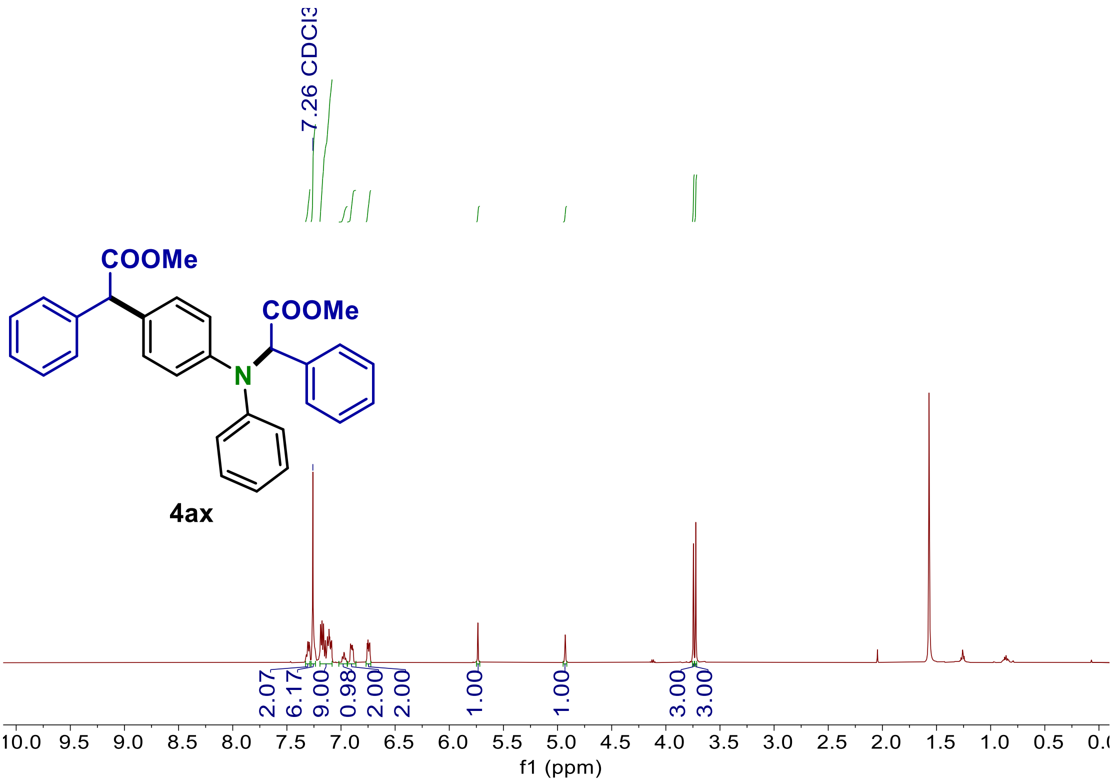

Figure S175. ^{1}H-NMR spectra of 4ax.

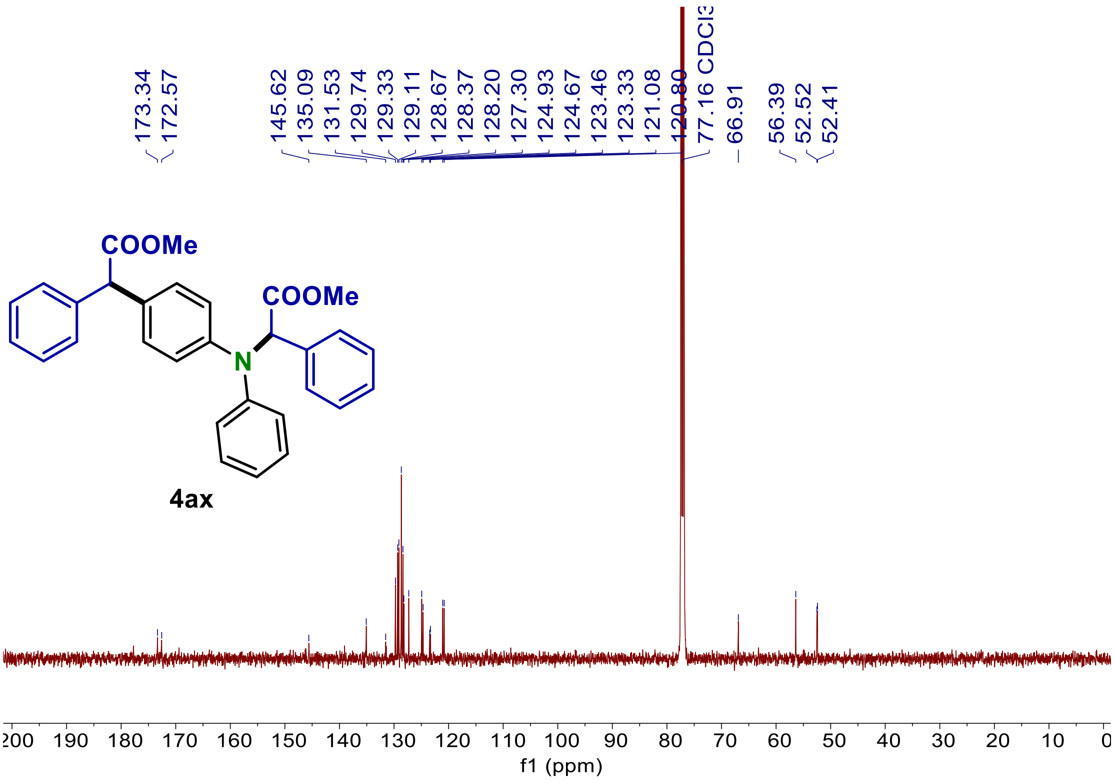

Figure S176. ^{13}C-NMR spectra of 4ax.

Methyl (R)-2-((4-(2-hydroxyethyl)phenyl)amino)-2-(p-tolyl)acetate (3bm)

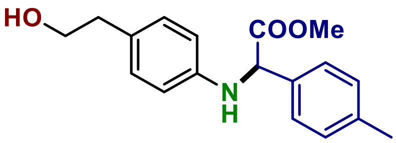

¹H-NMR (500 MHz, $CDCl_3$) δ p.p.m. 7.37 (d, *J* = 7.9 Hz, 2H), 7.16 (d, *J* = 7.8 Hz, 2H), 6.98 (d, *J* = 8.4 Hz, 2H), 6.52 (d, *J* = 8.4 Hz, 2H), 5.02 (s, 1H), 4.87 (s, 1H), 3.75 (t, *J* = 6.5 Hz, 2H), 3.72 (s, 3H), 2.72 (t, *J* = 6.5 Hz, 2H), 2.34 (s, 3H); **¹³C-NMR** (126 MHz, $CDCl_3$) δ 172.66, 144.78, 138.26, 134.72, 129.95, 129.72, 127.68, 127.26, 113.74, 63.97, 60.68, 52.88, 38.36, 21.27; **HRMS (ESI)** calcd. for $[M+H]^+$: 300.1594 m/z, found: 300.1591 m/z.

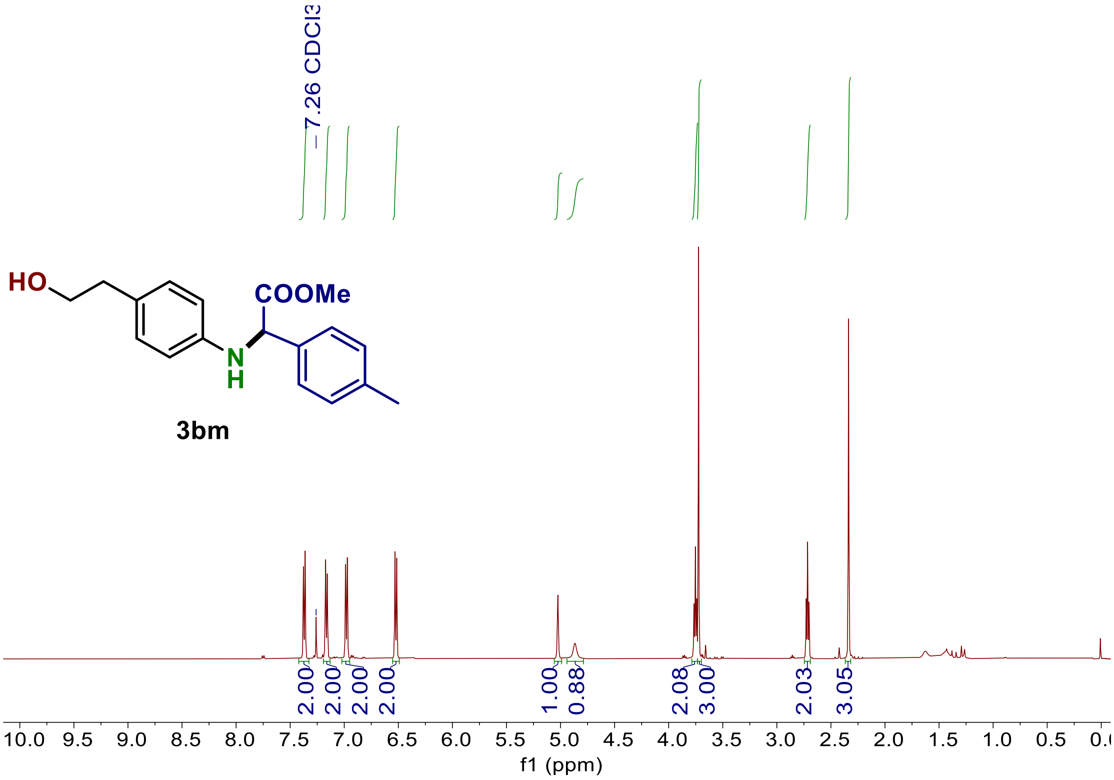

Figure S177. ¹H-NMR spectra of 3bm.

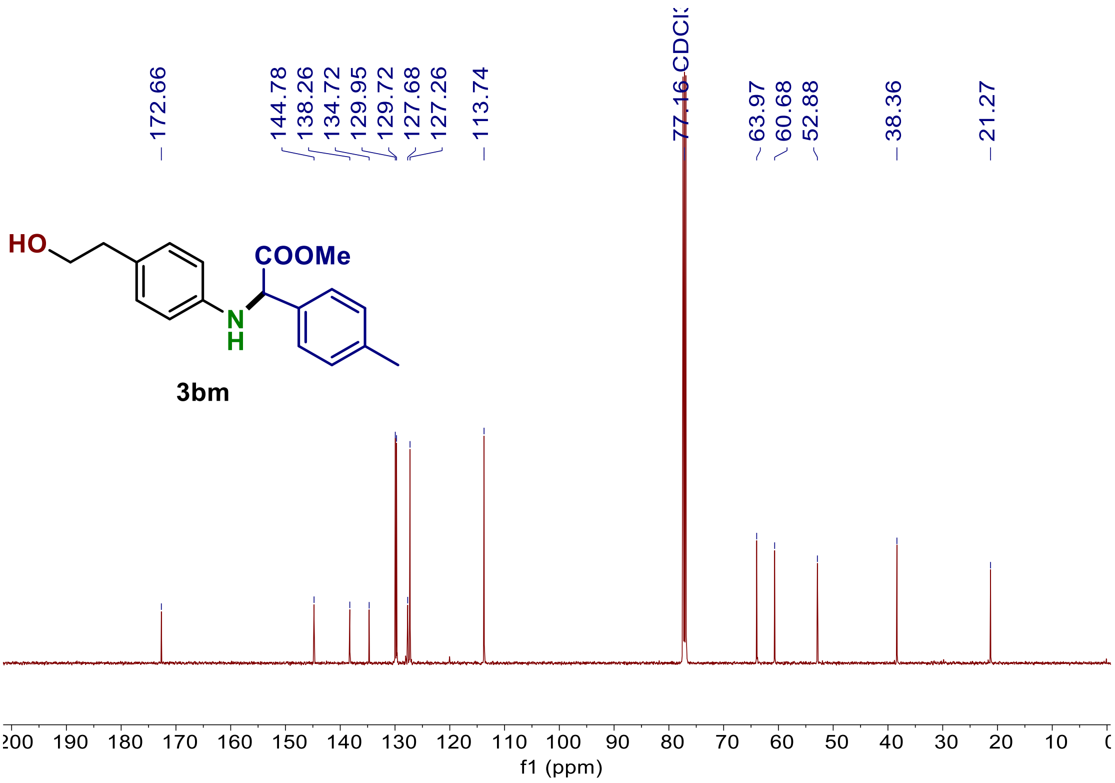

Figure S178. ^{13}C-NMR spectra of 3bm.

Methyl (R)-2-((4-(2-((R)-2-methoxy-2-oxo-1-(p-tolyl)ethoxy)ethyl)phenyl)amino)-2-(p-tolyl)acetate (4bm)

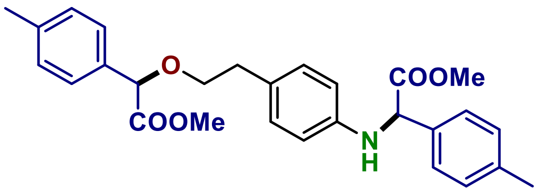

^{1}H-NMR (500 MHz, $CDCl_3$) δ p.p.m. 7.36 (d, J = 7.9 Hz, 2H), 7.28 (dd, J = 8.2, 2.7 Hz, 2H), 7.15 (t, J = 6.7 Hz, 4H), 6.95 (d, J = 8.2 Hz, 2H), 6.47 (d, J = 8.3 Hz, 2H), 5.01 (s, 1H), 4.82 (d, J = 3.8 Hz, 1H), 3.72 (s, 3H), 3.68 (d, J = 1.4 Hz, 3H), 3.66 – 3.58 (m, 1H), 3.56 – 3.48 (m, 1H), 2.88 – 2.75 (m, 2H), 2.33 (d, J = 3.3 Hz, 6H); **^{13}C-NMR** (126 MHz, $CDCl_3$) δ 172.67, 171.64, 144.59, 138.59, 138.21, 134.77, 133.66, 129.86, 129.70, 129.41, 127.82, 127.30, 127.26, 113.56, 81.10, 71.20, 60.71, 52.86, 52.32, 35.42, 21.33, 21.28; **HRMS (ESI)** calcd. for $[M+H]^+$: 462.2275 m/z, found: 462.2271 m/z.

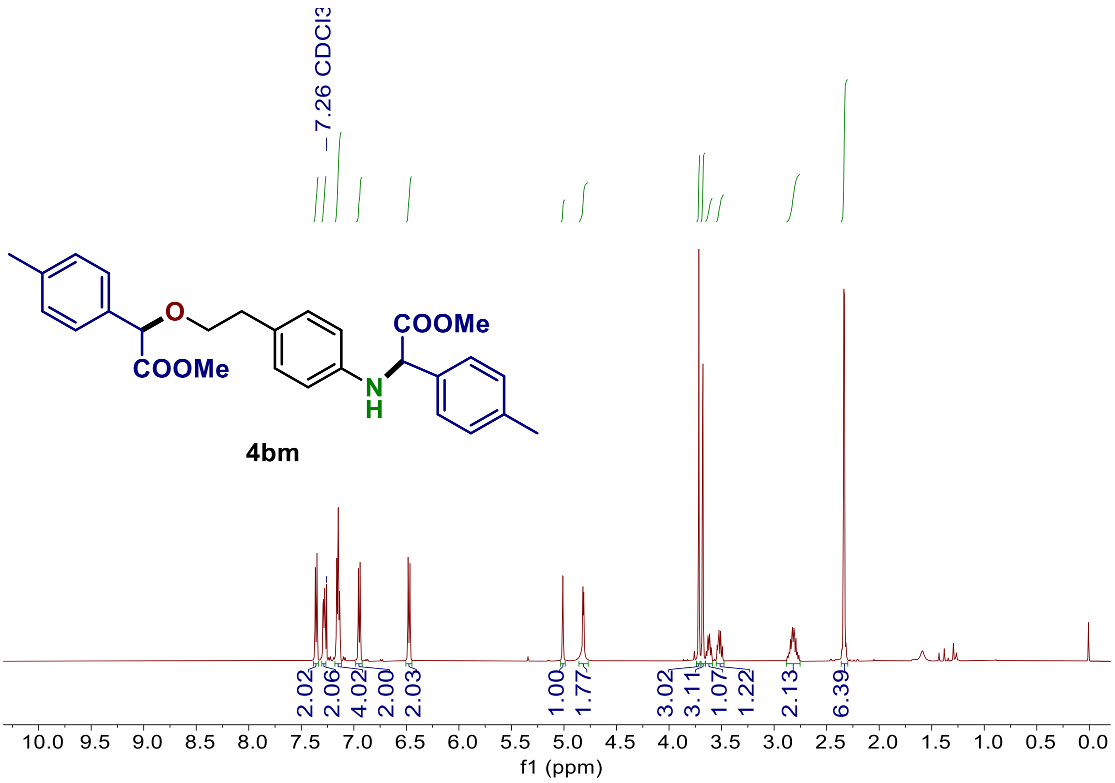

Figure S179. ^{1}H-NMR spectra of 4bm.

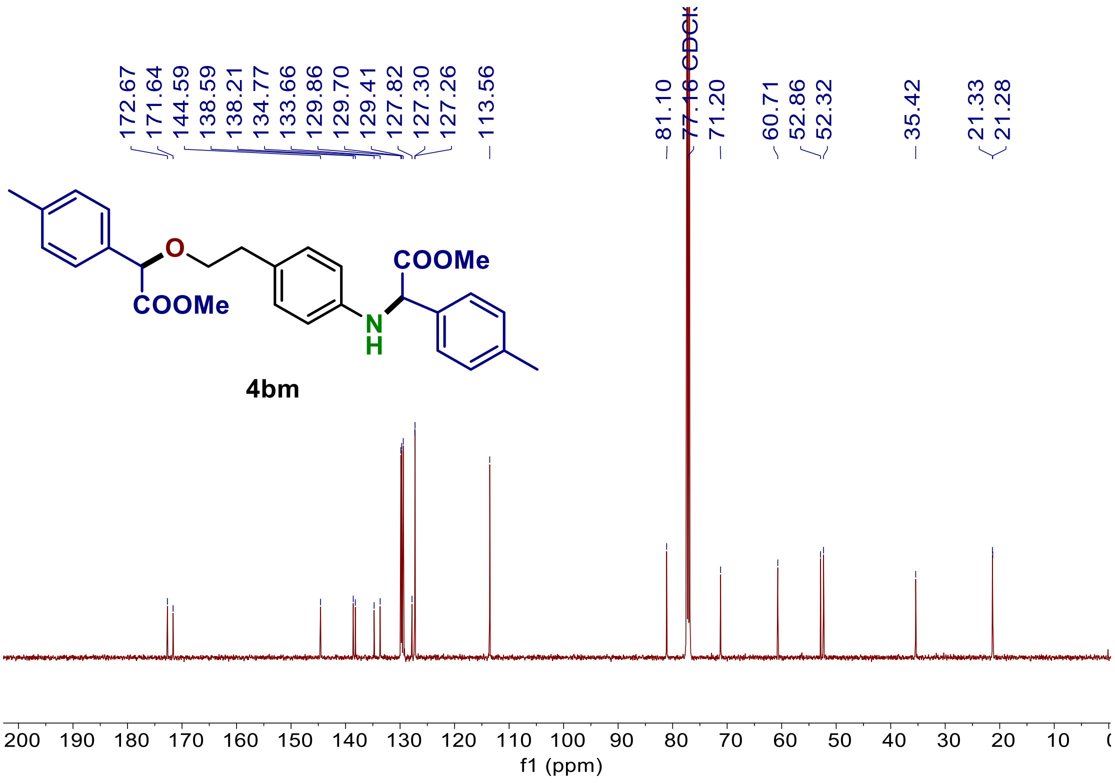

Figure S180. ^{13}C-NMR spectra of 4bm.

Methyl (R)-2-(4-chlorophenyl)-2-((4-(2-hydroxyethyl)phenyl)amino)acetate (3cm)

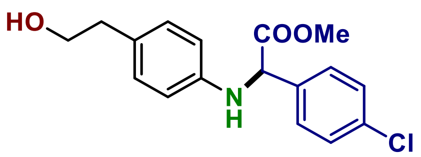

^{1}H-NMR (500 MHz, $CDCl_3$) δ p.p.m. 7.46 – 7.41 (m, 2H), 7.35 – 7.30 (m, 2H), 6.98 (d, J = 8.2 Hz, 2H), 6.48 (d, J = 8.5 Hz,, 2H), 5.02 (s, 1H), 4.93 (s, 1H), 3.76 (t, J = 6.5 Hz, 2H), 3.74 (s, 3H), 2.72 (t, J = 6.5 Hz, 2H); **^{13}C-NMR** (126 MHz, $CDCl_3$) δ 171.98, 144.39, 136.37, 134.31, 130.02, 129.22, 128.74, 128.05, 113.83, 63.98, 60.35, 53.12, 38.37; **HRMS (ESI)** calcd. for $[M+H]^+$: 320.1048 m/z, found: 320.1048 m/z.

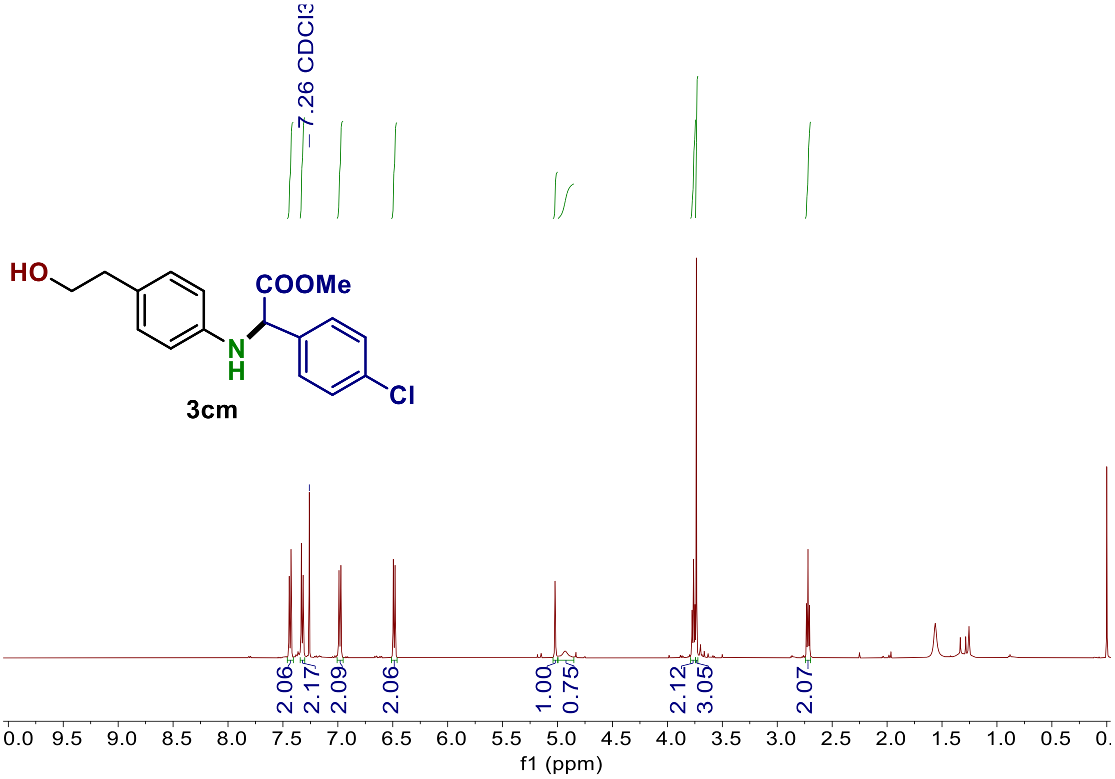

Figure S181. ^{1}H-NMR spectra of 3cm.

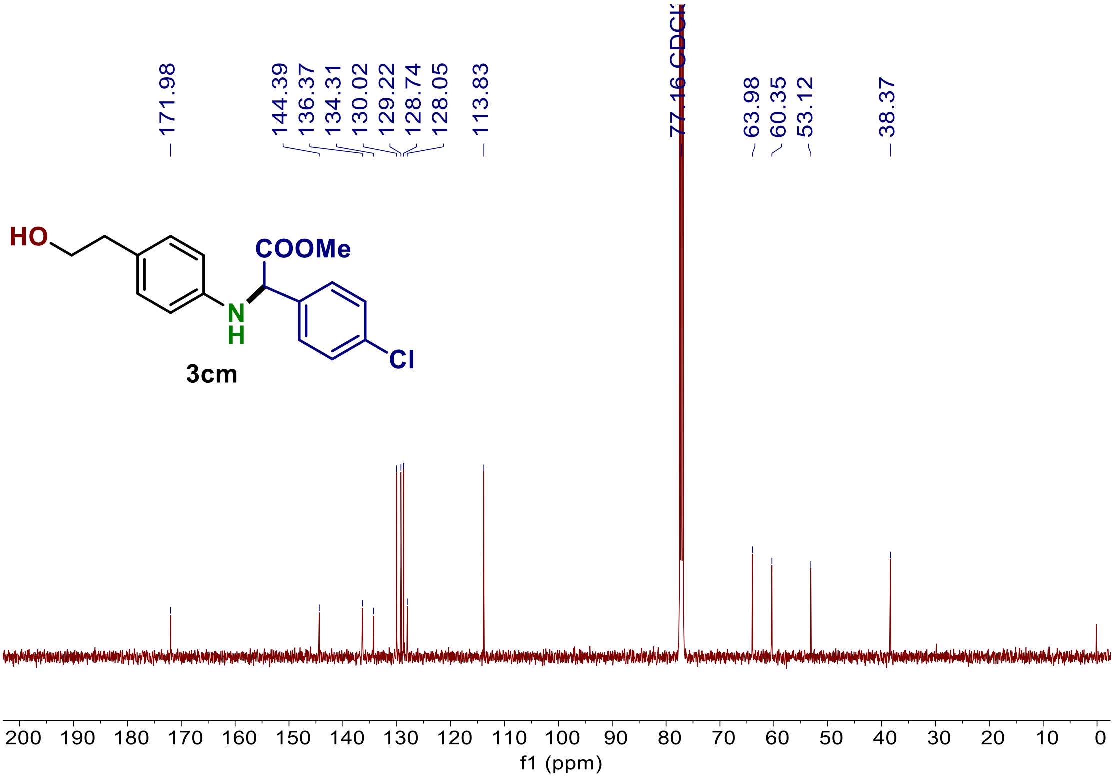

Figure S182. ^{13}C-NMR spectra of 3cm.

Methyl (R)-2-(4-chlorophenyl)-2-((4-(2-((R)-1-(4-chlorophenyl)-2-methoxy-2-oxoethoxy)ethyl)phenyl)amino)acetate (**4cm**)

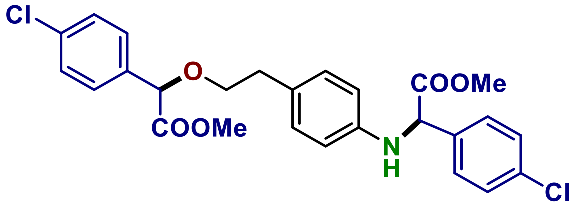

^{1}H-NMR (500 MHz, $CDCl_3$) δ p.p.m. 7.43 (d, J = 8.3 Hz, 2H), 7.31 (td, J = 8.4, 6.3 Hz, 6H), 6.95 (d, J = 8.1 Hz, 2H), 6.45 (d, J = 8.1 Hz, 2H), 5.02 (d, J = 2.9 Hz, 1H), 4.91 (s, 1H), 4.81 (d, J = 4.6 Hz, 1H), 3.73 (s, 3H), 3.68 (s, 3H), 3.65 (td, J = 6.1, 3.1 Hz, 1H), 3.55 – 3.47 (m, 1H), 2.88 – 2.76 (m, 2H); **^{13}C-NMR** (126 MHz, $CDCl_3$) δ 171.96, 171.09, 144.21, 136.37, 135.15, 134.62, 134.26, 129.91, 129.18, 128.88, 128.72, 128.60, 128.01, 113.61, 80.52, 71.36, 60.32, 53.08, 52.47, 35.38; **HRMS (ESI)** calcd. for $[M+H]^+$: 502.1183 m/z, found: 502.1181m/z.

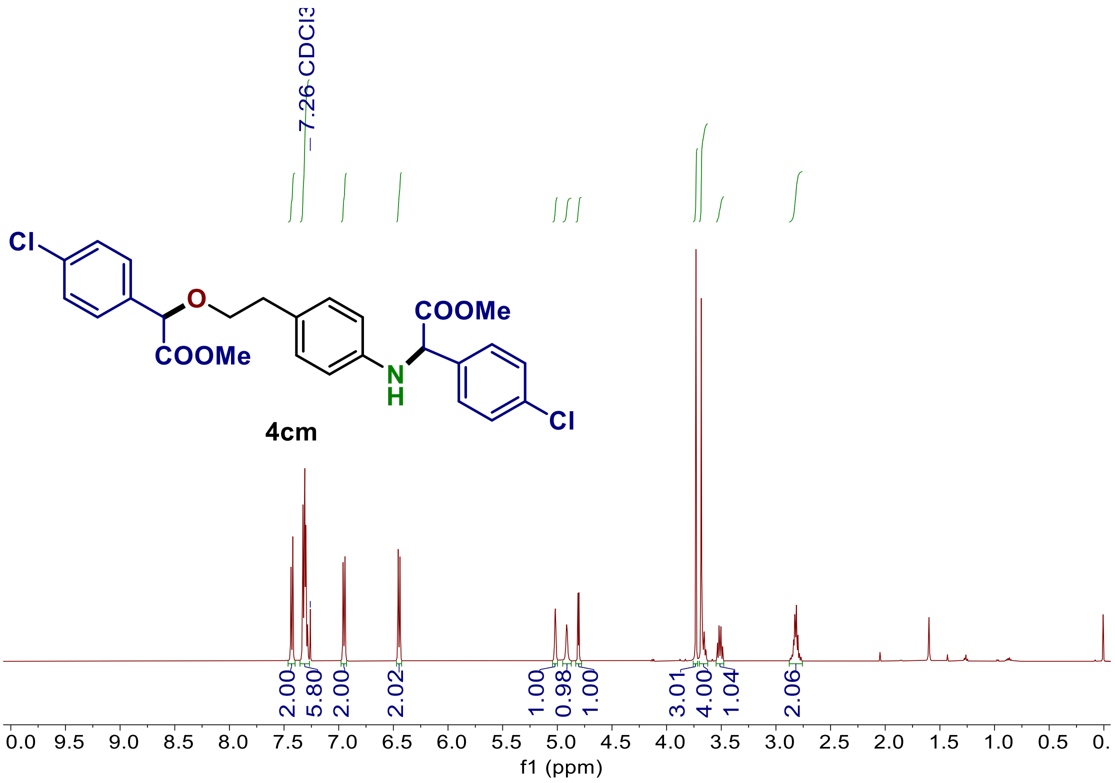

Figure S183. ^{1}H-NMR spectra of 4cm.

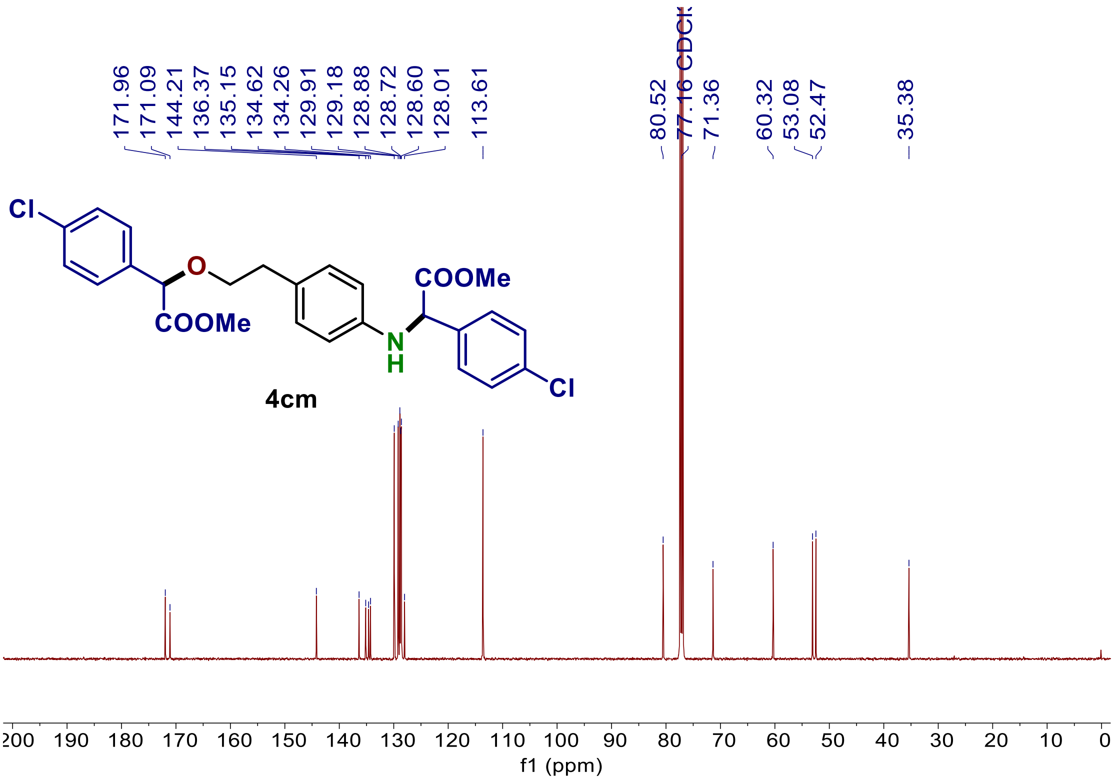

Figure S184. ^{13}C-NMR spectra of 4cm.

Methyl (R)-2-(3-fluorophenyl)-2-((4-(2-hydroxyethyl)phenyl)amino)acetate (3dm)

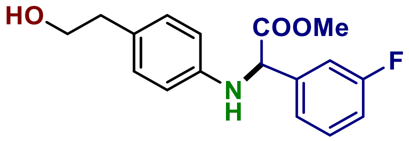

^{1}H-NMR (500 MHz, $CDCl_3$) δ p.p.m. 7.36 – 7.27 (m, 2H), 7.21 (dt, *J* = 9.5, 2.0 Hz, 1H), 7.03 – 6.95 (m, 3H), 6.50 (d, *J* = 8.4 Hz, 2H), 5.04 (s, 1H), 3.76 (t, *J* = 6.5 Hz, 2H), 3.74 (s, 3H), 2.72 (t, *J* = 6.5 Hz, 2H); **^{13}C-NMR** (126 MHz, $CDCl_3$) δ 171.87, 164.22, 162.26, 144.39, 140.49, 140.44, 130.54, 130.48, 130.01, 128.08, 123.07, 123.05, 115.55, 115.38, 114.47, 114.29, 113.82, 63.97, 60.53, 53.14, 38.36; **HRMS (ESI)** calcd. for $[M+H]^+$: 304.1343 m/z, found: 304.1338m/z.

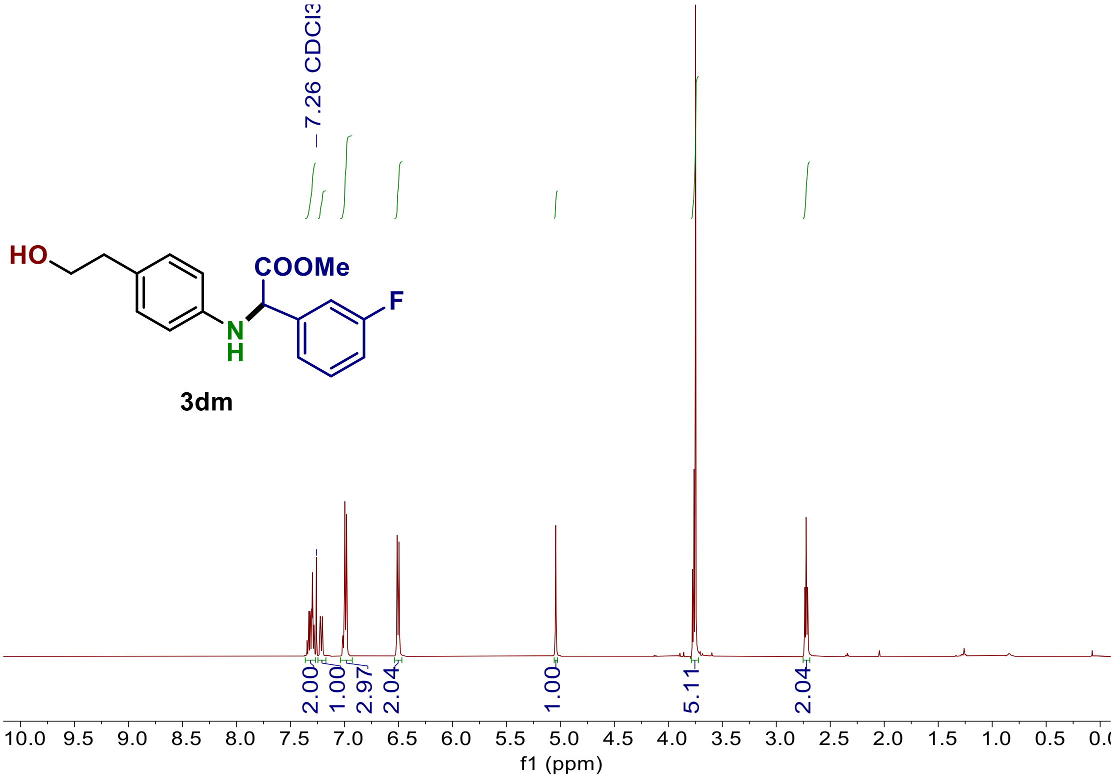

Figure S185. ^{1}H-NMR spectra of 3dm.

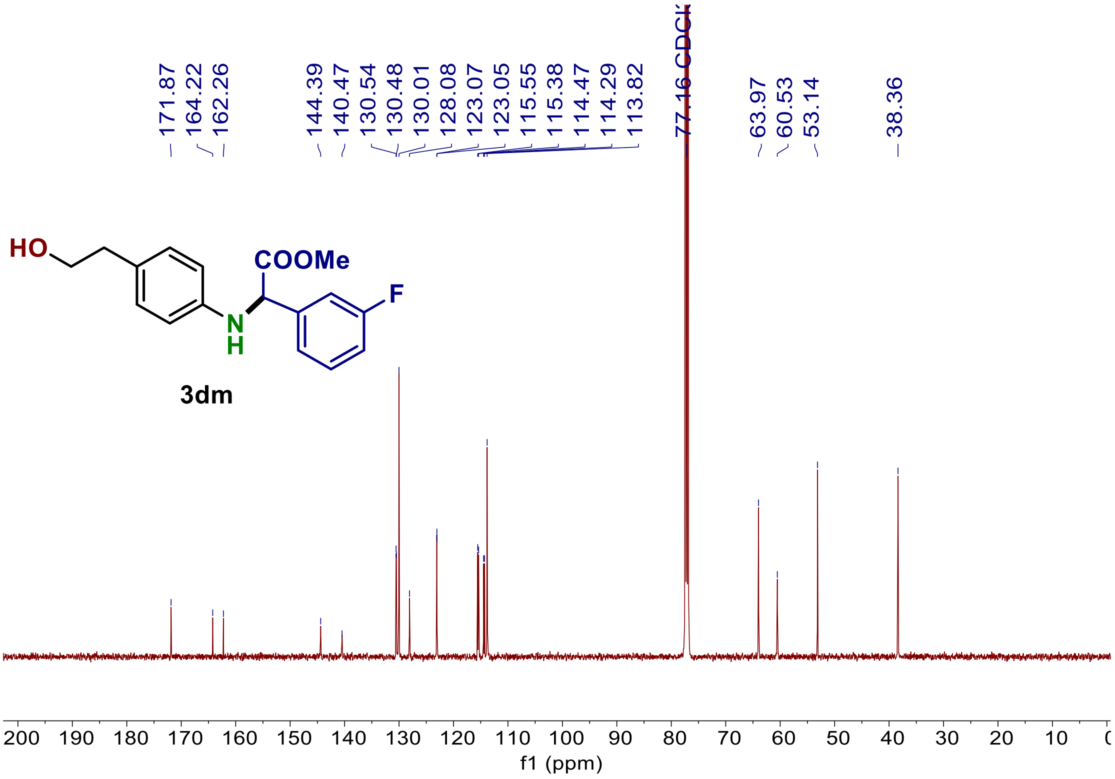

Figure S186. ^{13}C-NMR spectra of 3dm.

Methyl (R)-2-(3-fluorophenyl)-2-((4-(2-((S)-1-(3-fluorophenyl)-2-methoxy-2-oxoethoxy)ethyl)phenyl)amino)acetate (**4dm**)

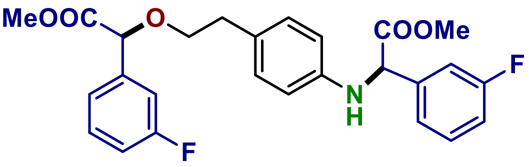

^{1}H-NMR (500 MHz, $CDCl_3$) δ p.p.m. 7.34 – 7.27 (m, 3H), 7.20 (dt, *J* = 9.4, 2.1 Hz, 1H), 7.18 – 7.15 (m, 1H), 7.12 (dq, *J* = 9.7, 2.2 Hz, 1H), 7.00 (dd, *J* = 9.8, 6.4 Hz, 2H), 6.96 (d, *J* = 8.4 Hz, 2H), 6.46 (d, *J* = 8.4 Hz, 2H), 5.03 (s, 1H), 4.83 (s, 1H), 3.74 (s, 3H), 3.69 (d, *J* = 1.4 Hz, 3H), 3.68 – 3.64 (m, 1H), 3.53 (td, *J* = 8.6, 6.9 Hz, 1H), 2.82 (hept, *J* = 7.0 Hz, 2H); **^{13}C-NMR** (126 MHz, $CDCl_3$) δ171.87, 171.00, 164.22, 163.96, 162.66, 162.26, 161.99, 144.28, 140.52, 140.46, 139.10, 139.04, 130.52, 130.46, 130.24, 130.17, 129.93, 128.03, 123.06, 123.04, 122.85, 115.78, 115.61, 115.53, 115.36, 114.46, 114.32, 114.28, 114.16, 113.63, 80.61, 71.48, 60.55, 53.13, 52.52, 35.40; **HRMS (ESI)** calcd. for $[M+H]^+$: 470.1774 m/z, found: 470.1761 m/z.

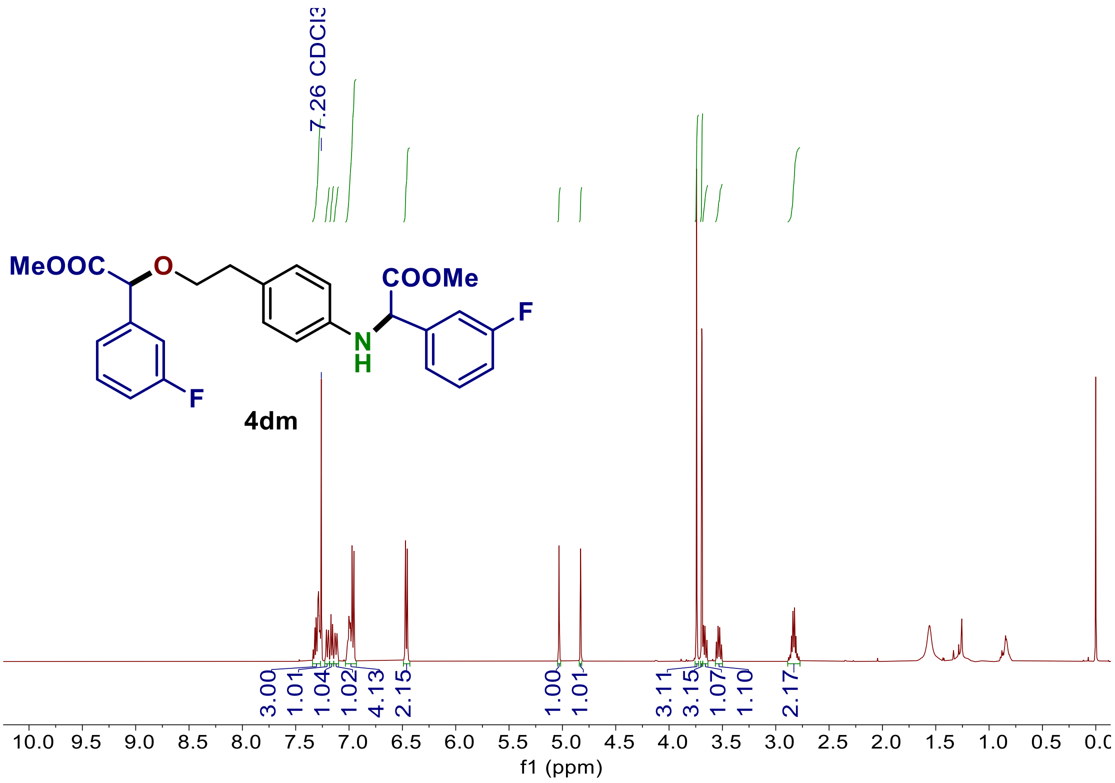

Figure S187. ^{1}H-NMR spectra of 4dm.

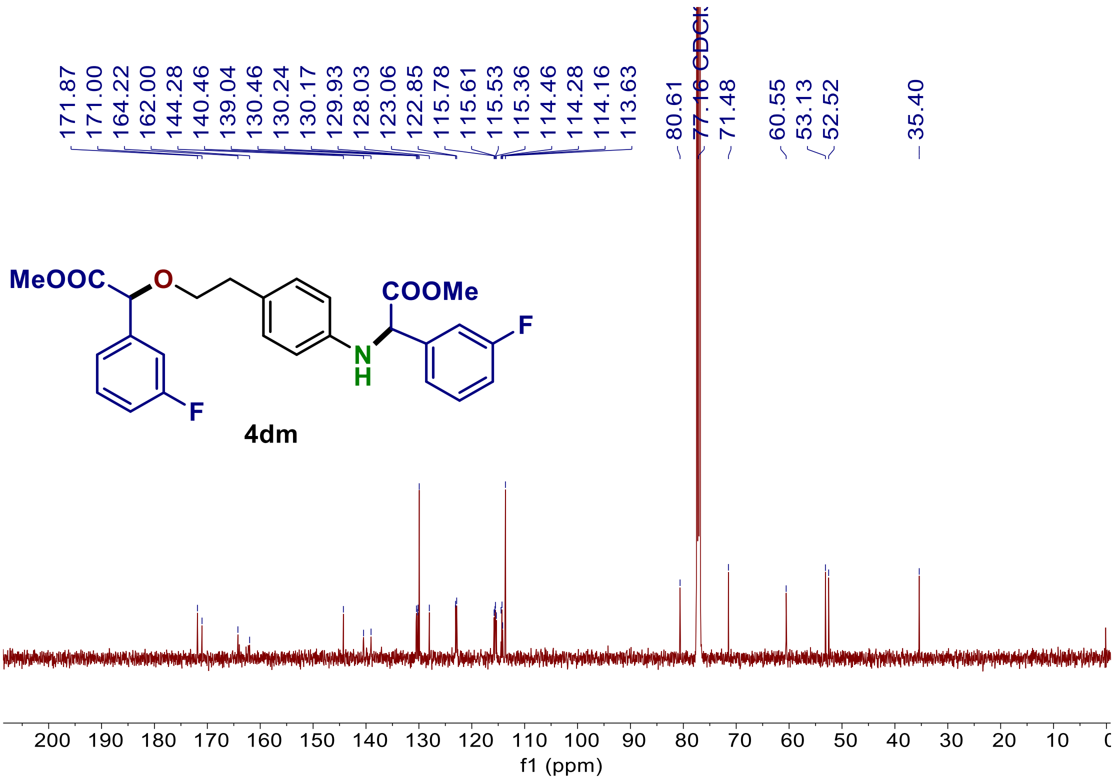

Figure S188. ^{13}C-NMR spectra of 4dm.

Methyl (R)-2-(2-(benzyloxy)phenyl)-2-((4-(2-hydroxyethyl)phenyl)amino)acetate (3em)

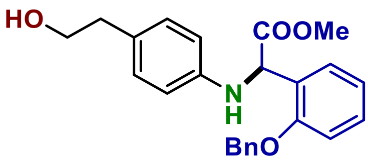

^{1}H-NMR (400 MHz, $CDCl_3$) δ p.p.m. 7.50 – 7.44 (m, 2H), 7.38 (dtd, J = 12.8, 7.7, 3.5 Hz, 4H), 7.28 – 7.28 (m, 1H), 7.00 – 6.91 (m, 4H), 6.55 (d, J = 4.8 Hz, 2H), 5.58 (s, 1H), 5.19 (s, 2H), 3.76 (t, J = 6.6 Hz, 2H), 3.68 (s, 3H), 2.71 (t, J = 6.5 Hz, 2H); **^{13}C-NMR** (101 MHz, $CDCl_3$) δ 172.95, 156.36, 145.12, 137.00, 129.92, 129.52, 128.76, 128.41, 128.13, 127.56, 127.36, 126.86, 121.46, 113.89, 112.52, 70.45, 64.01, 55.15, 52.72, 38.41; **HRMS (ESI)** calcd. for $[M+H]^+$: 392.1856 m/z, found: 392.1857 m/z.

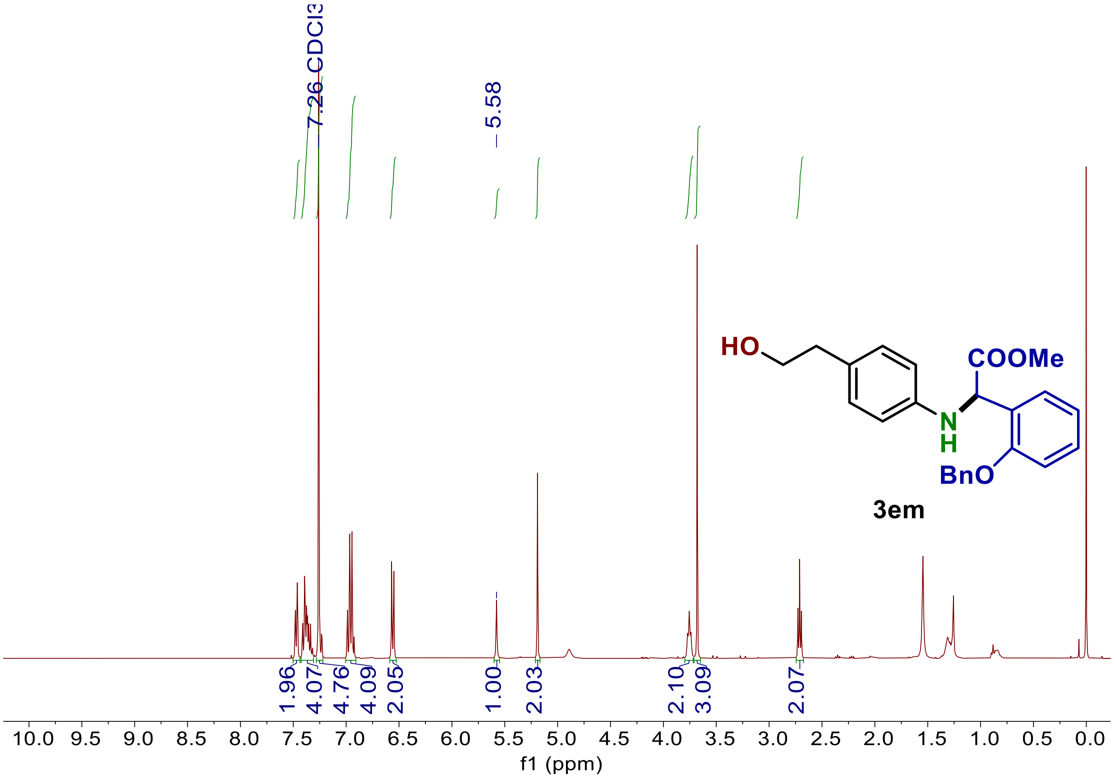

Figure S189. ^{1}H-NMR spectra of 3em.

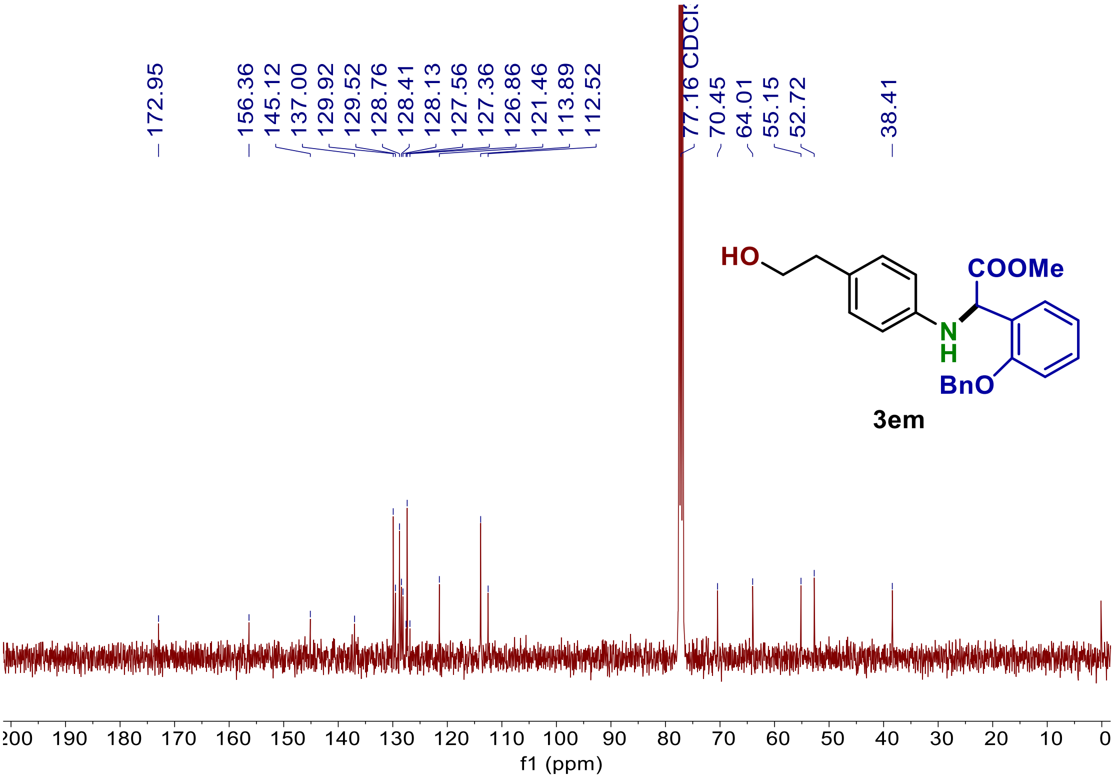

Figure S190. ^{13}C-NMR spectra of 3em.

Methyl (R)-2-(2-(benzyloxy)phenyl)-2-((4-(2-((S)-1-(2-(benzyloxy)phenyl)-2-methoxy-2-oxoethoxy)ethyl)phenyl)amino)acetate (**4em**)

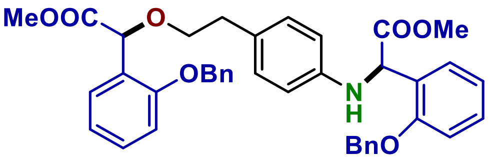

^{1}H-NMR (500 MHz, $CDCl_3$) δ p.p.m. 7.46 (d, J = 7.4 Hz, 2H), 7.42 – 7.20 (m, 12H), 6.98 – 6.89 (m, 4H), 6.88 (d, J = 8.4 Hz, 2H), 6.49 (d, J = 8.1 Hz, 2H), 5.55 (s, 1H), 5.38 (s, 1H), 5.17 (s, 2H), 5.13 – 5.02 (m, 2H), 3.66 (s, 3H), 3.64 (s, 3H), 3.69 – 3.61 (m, 1H), 3.53 (td, J = 9.1, 6.4 Hz, 1H), 2.78 (qdd, J = 13.9, 9.1, 6.4 Hz, 2H); **^{13}C-NMR** (126 MHz, $CDCl_3$) δ 172.96, 171.80, 156.36, 156.25, 144.80, 137.05, 137.01, 129.80, 129.46, 128.75, 128.66, 128.50, 128.41, 128.11, 128.00, 127.82, 127.35, 127.32, 126.90, 125.84, 121.43, 121.30, 113.66, 112.49, 112.32, 75.12, 71.57, 71.56, 70.43, 70.40, 55.21, 52.68, 52.17, 35.44; **HRMS (ESI)** calcd. for $[M+H]^+$: 646.2799 m/z, found: 646.2806 m/z.

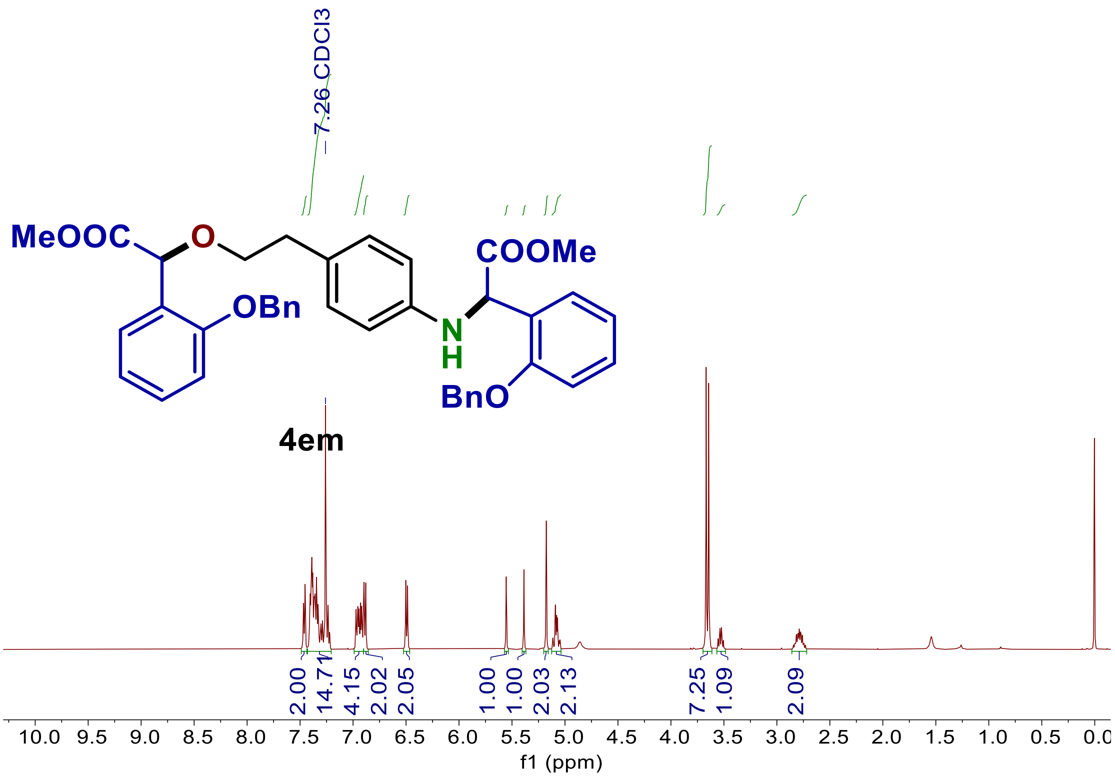

Figure S191. ^{1}H-NMR spectra of 4em.

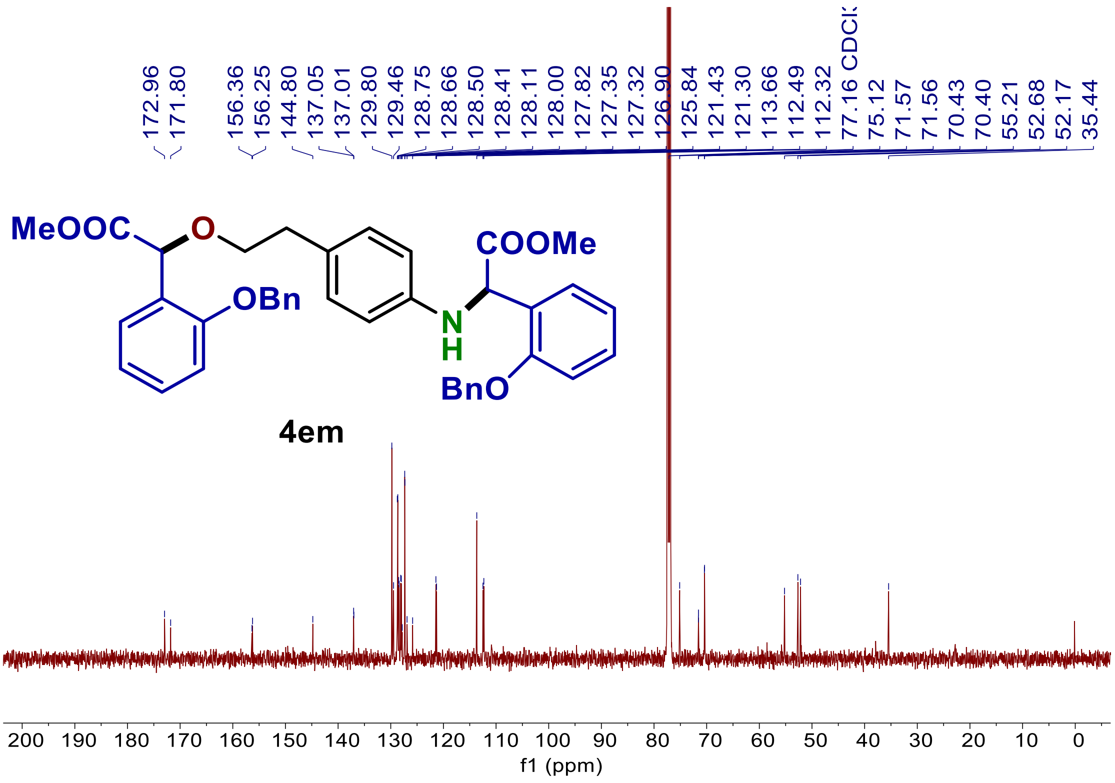

Figure S192. ^{13}C-NMR spectra of 4em.

Ethyl (R)-2-((4-(2-hydroxyethyl)phenyl)amino)-2-phenylacetate (3fm)

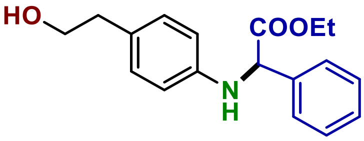

^{1}H-NMR (500 MHz, $CDCl_3$) δ p.p.m. 7.49 (dd, *J* = 7.3, 1.8 Hz, 2H), 7.39 – 7.28 (m, 3H), 6.98 (d, *J* = 8.4 Hz, 2H), 6.52 (d, *J* = 8.4 Hz, 2H), 5.04 (d, *J* = 5.7 Hz, 1H), 4.90 (d, *J* = 5.9 Hz, 1H), 4.23 (dq, *J* = 10.7, 7.1 Hz, 1H), 4.14 (dq, *J* = 10.8, 7.1 Hz, 1H), 3.76 (q, *J* = 6.2 Hz, 2H), 2.72 (t, *J* = 6.5 Hz, 2H), 1.21 (t, *J* = 7.1 Hz, 3H); **^{13}C-NMR** (126 MHz, $CDCl_3$) δ 171.98, 144.81, 137.90, 129.97, 128.96, 128.37, 127.67, 127.34, 113.79, 64.00, 61.96, 61.06, 38.39, 14.18; **HRMS (ESI)** calcd. for $[M+H]^+$: 300.1594 m/z, found: 300.1605 m/z

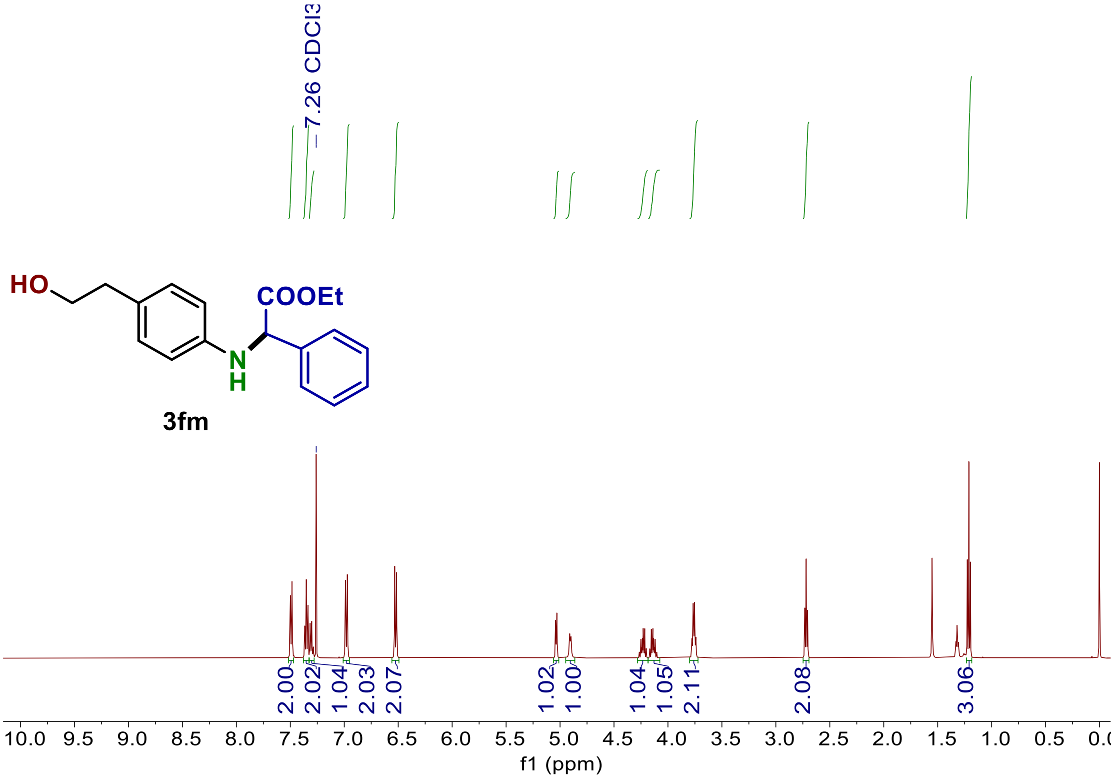

Figure S193. ^{1}H-NMR spectra of 3fm.

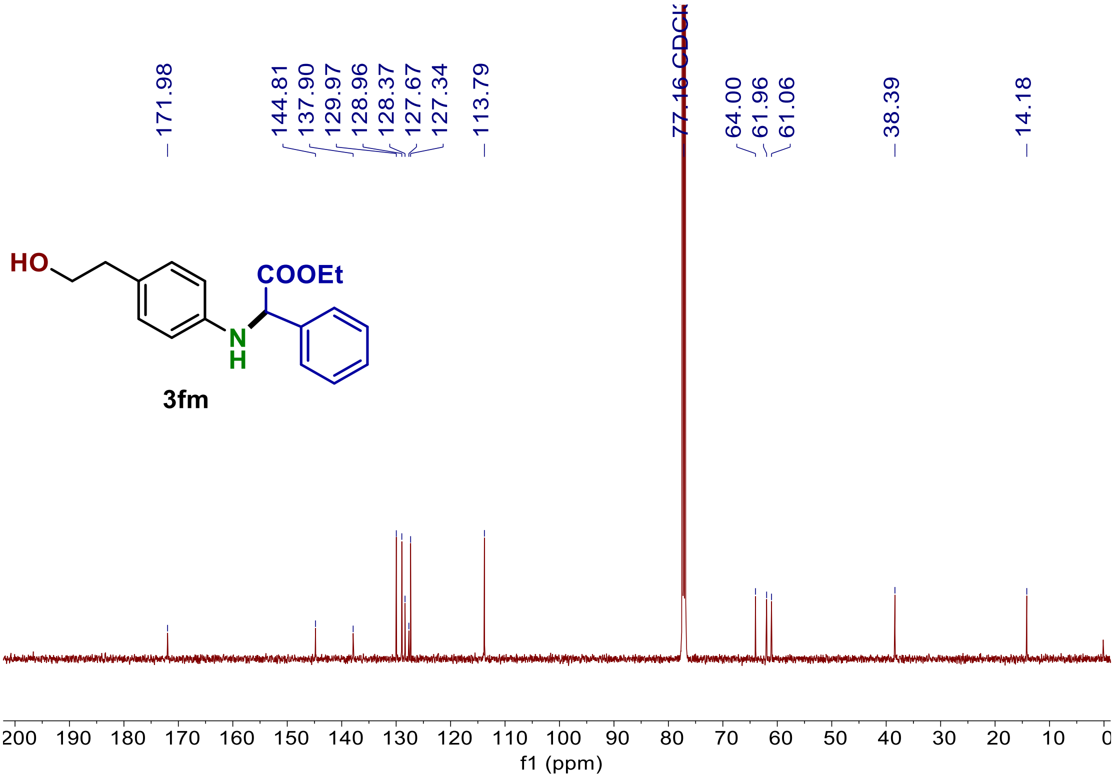

Figure S194. ^{13}C-NMR spectra of 3fm.

Ethyl (R)-2-((4-(2-((S)-2-ethoxy-2-oxo-1-phenylethoxy)ethyl)phenyl)amino)-2-phenylacetate (4fm)

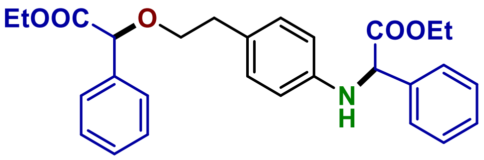

^{1}H-NMR (500 MHz, $CDCl_3$) δ p.p.m. 7.50 – 7.45 (m, 2H), 7.40 (dt, J = 7.5, 1.8 Hz, 2H), 7.37 – 7.27 (m, 6H), 6.95 (d, J = 8.4 Hz, 2H), 6.47 (d, J = 8.4 Hz, 2H), 5.02 (s, 1H), 4.82 (d, J = 2.7 Hz, 1H), 4.26 – 4.08 (m, 4H), 3.70 – 3.61 (m, 1H), 3.53 (td, J = 8.9, 6.6 Hz, 1H), 2.90 – 2.76 (m, 2H), 1.23 – 1.16 (m, 6H); **^{13}C-NMR** (126 MHz, $CDCl_3$) δ 172.00, 171.08, 144.60, 137.93, 136.79, 129.89, 128.93, 128.65, 128.33, 127.86, 127.34, 127.29, 113.59, 81.34, 71.32, 61.92, 61.29, 61.08, 35.44, 14.23, 14.17; **HRMS (ESI)** calcd. for $[M+H]^+$: 462.2275 m/z, found: 462.2259 m/z

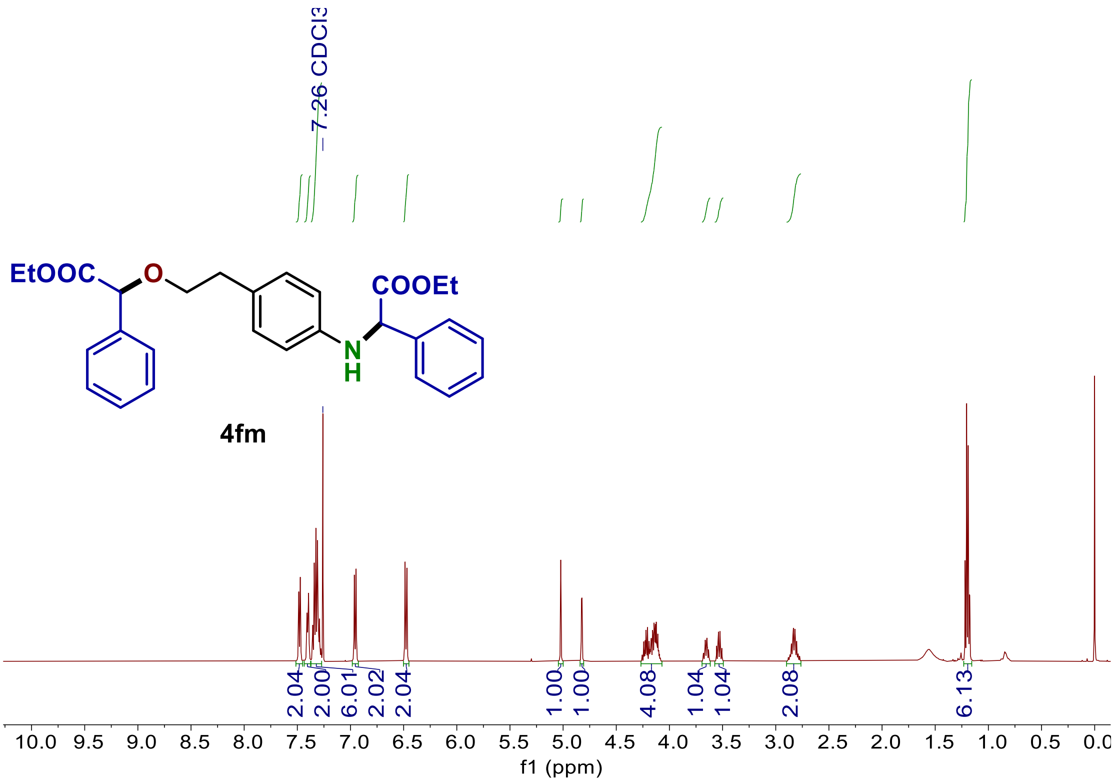

Figure S195. ^{1}H-NMR spectra of 4fm.

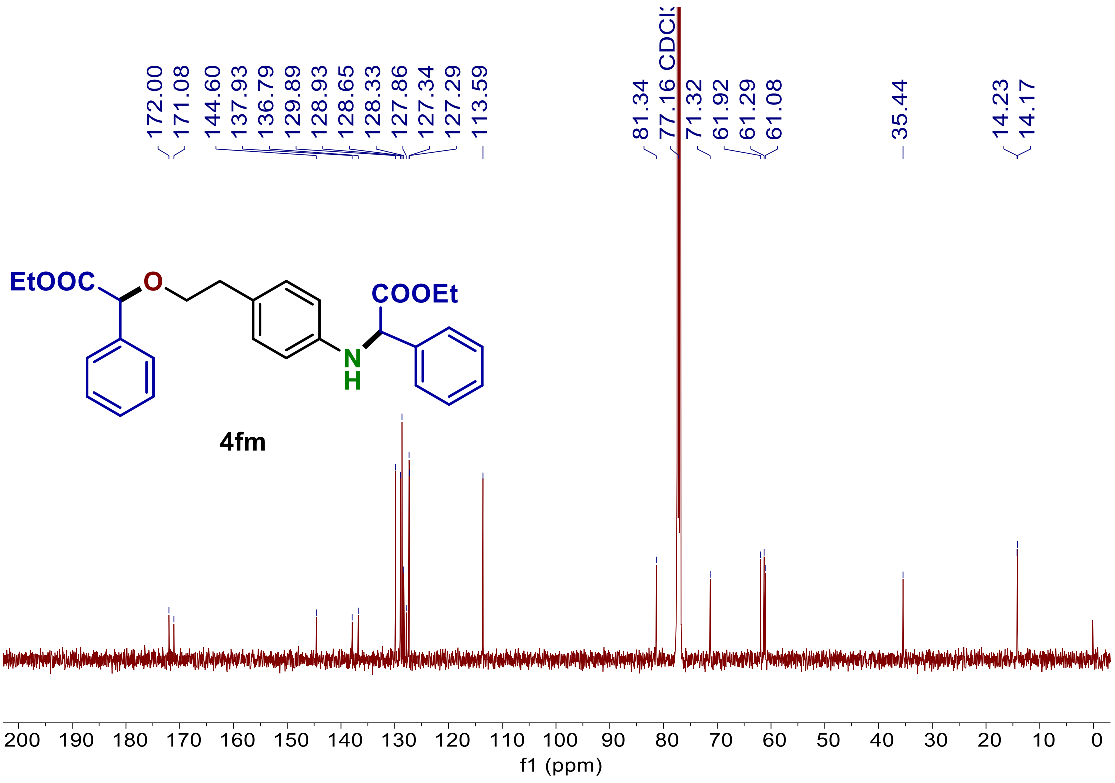

Figure S196. ^{13}C-NMR spectra of 4fm.

Ethyl (R)-2-(4-bromophenyl)-2-((4-(2-hydroxyethyl)phenyl)amino)acetate (3gm)

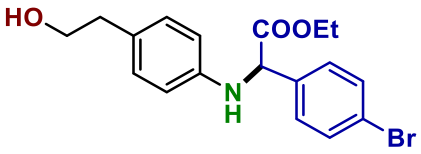

[1]**H-NMR** (500 MHz, $CDCl_3$) δ p.p.m. δ 7.50 – 7.45 (m, 2H), 7.41 – 7.36 (m, 2H), 6.98 (d, J = 8.5 Hz, 2H), 6.48 (d, J = 8.5 Hz, 2H), 4.98 (d, J = 4.7 Hz, 1H), 4.95 (d, J = 5.6 Hz, 1H), 4.23 (dq, J = 10.7, 7.1 Hz, 1H), 4.14 (dq, J = 10.8, 7.2 Hz, 1H), 3.76 (t, J = 6.5 Hz, 2H), 2.72 (t, J = 6.5 Hz, 2H), 1.22 (t, J = 7.1 Hz, 3H); **^{13}C-NMR** (126 MHz, $CDCl_3$) δ 171.36, 144.40, 137.06, 132.08, 130.00, 129.03, 127.94, 122.33, 113.80, 63.97, 62.22, 60.46, 38.36, 14.16; **HRMS (ESI)** calcd. for $[M+H]^+$: 378.0699m/z, found: 378.0697 m/z.

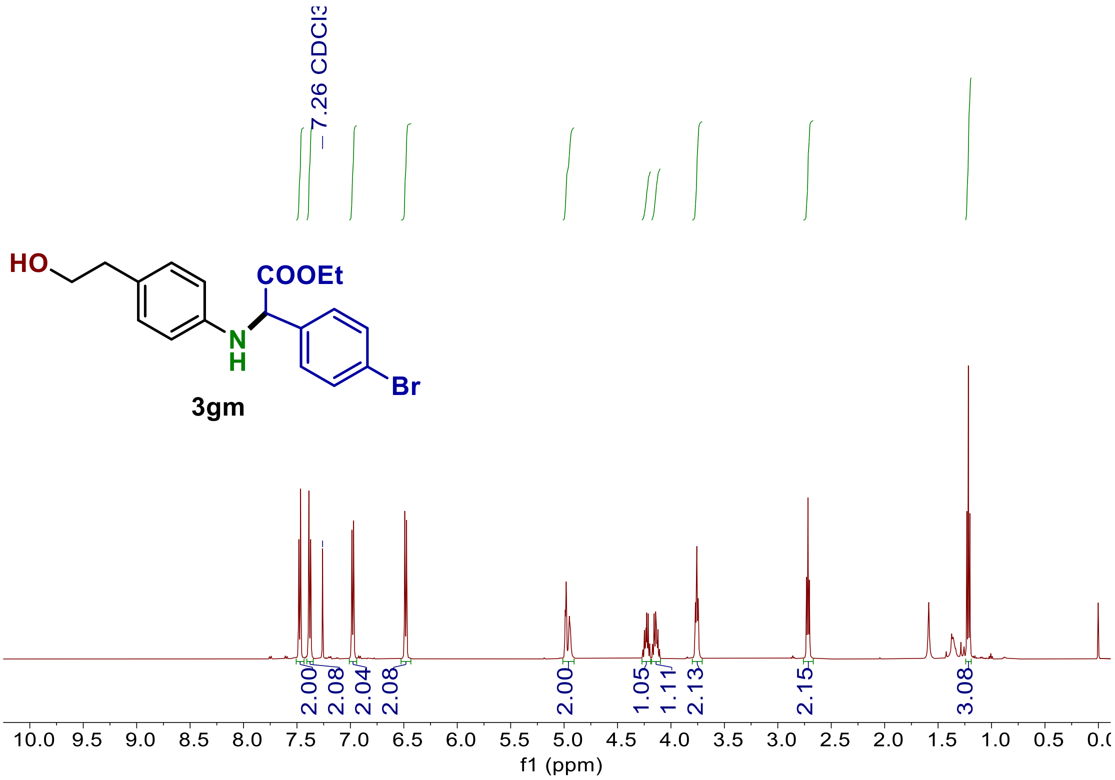

Figure S197. ^{1}H-NMR spectra of 3gm.

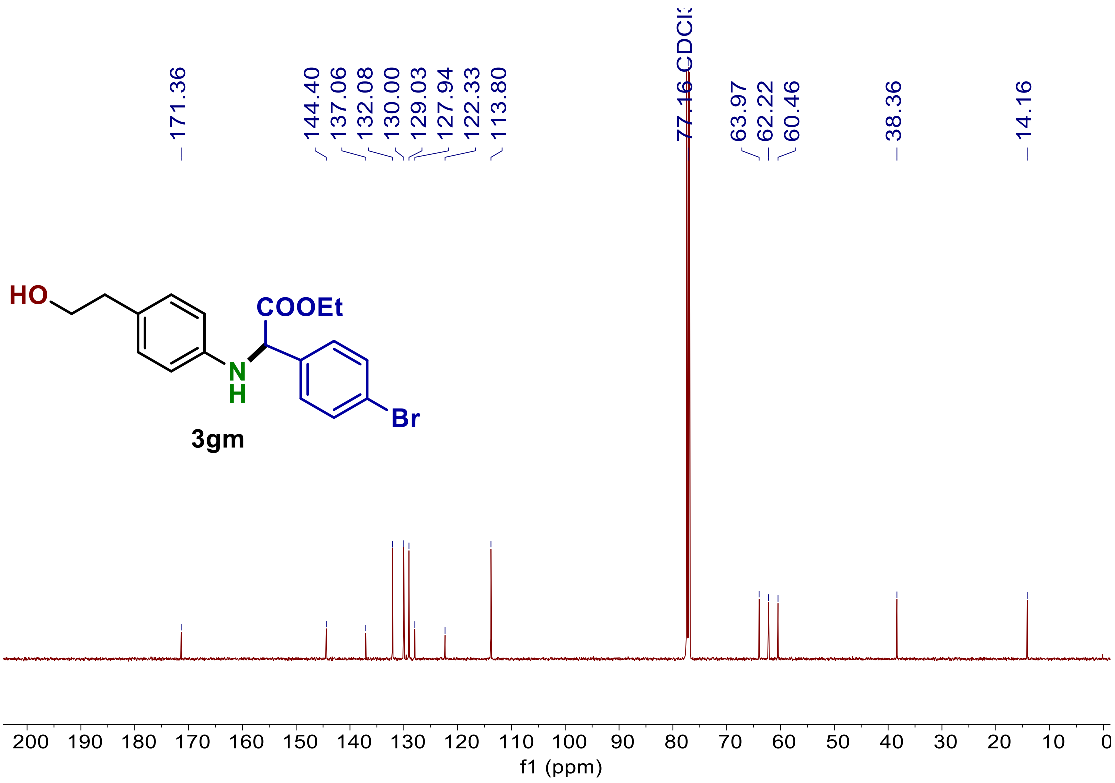

Figure S198. ^{13}C-NMR spectra of 3gm.

Ethyl (R)-2-(4-bromophenyl)-2-((4-(2-((S)-1-(4-bromophenyl)-2-ethoxy-2-oxoethoxy)ethyl)phenyl)amino)acetate (**4gm**)

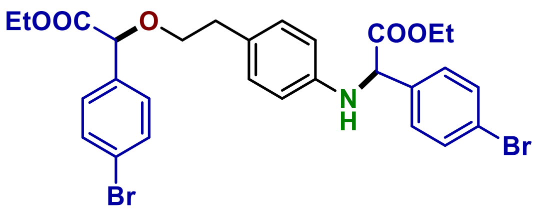

^{1}**H-NMR** (500 MHz, $CDCl_3$) δ 7.49 – 7.42 (m, 4H), 7.39 – 7.35 (m, 2H), 7.28 – 7.24 (m, 2H), 6.95 (d, *J* = 8.1 Hz, 2H), 6.43 (d, *J* = 8.5 Hz, 2H), 4.97 (d, *J* = 5.5 Hz, 1H), 4.91 (d, *J* = 5.6 Hz, 1H), 4.76 (d, *J* = 4.8 Hz, 1H), 4.27 – 4.08 (m, 4H), 3.70 – 3.63 (m, 1H), 3.54 – 3.47 (m, 1H), 2.80 (tt, *J* = 14.0, 6.3 Hz, 2H), 1.23 – 1.16 (m, 6H). ^{13}**C-NMR** (126 MHz, $CDCl_3$) δ 171.38, 170.61, 144.25, 137.09, 135.84, 132.08, 131.80, 129.93, 129.04, 128.90, 128.01, 122.73, 122.33, 113.61, 80.66, 71.36, 62.21, 61.49, 60.47, 35.41, 14.22, 14.16; **HRMS (ESI)** calcd. for $[M+H]^+$: 618.0485 m/z, found: 618.0483 m/z.

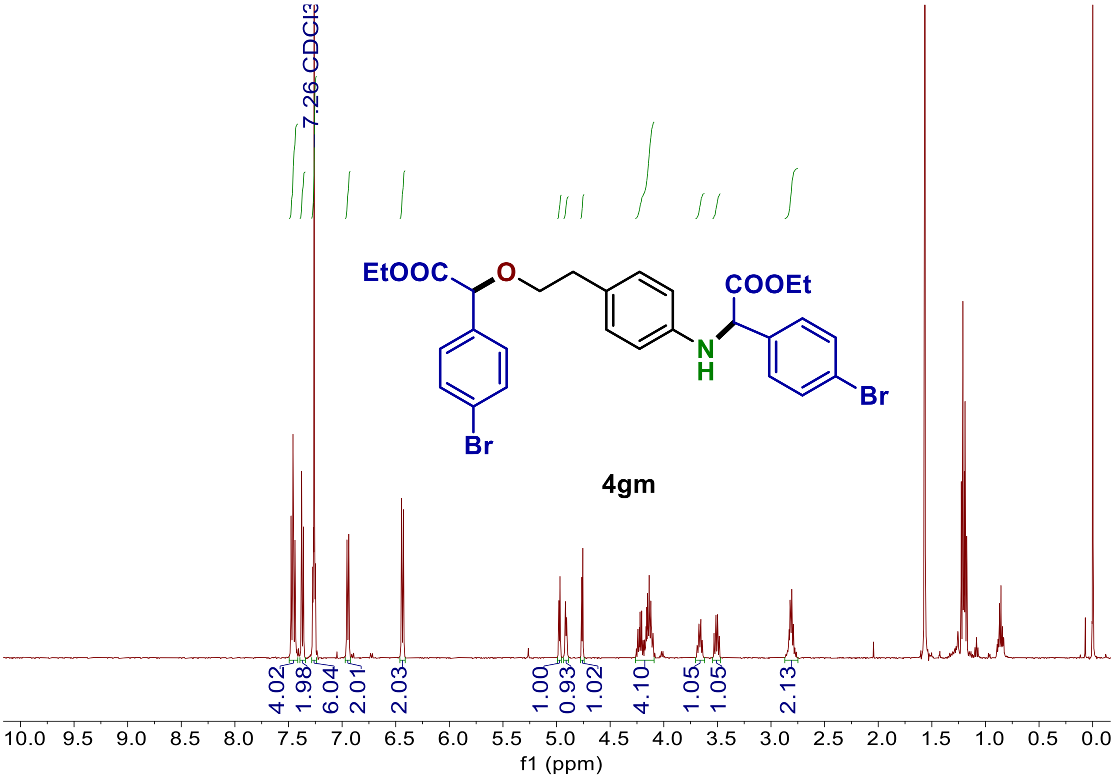

Figure S199. ^{1}H-NMR spectra of 4gm.

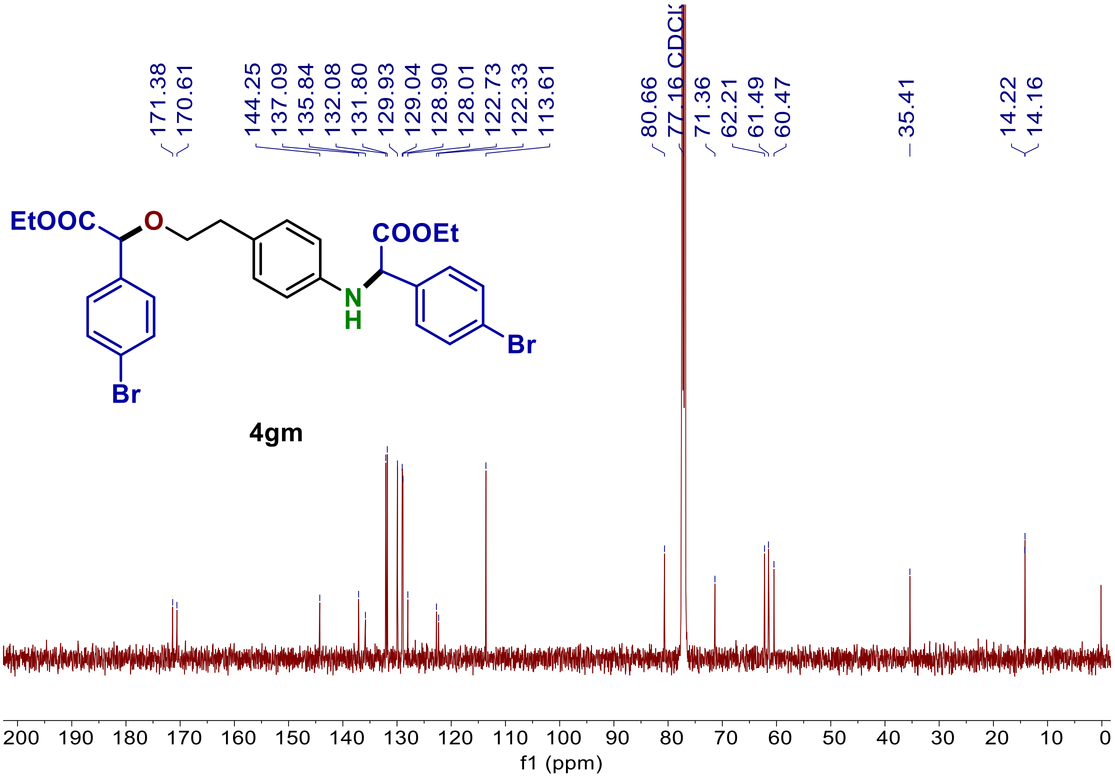

Figure S200. ^{13}C-NMR spectra of 4gm.

Allyl (R)-2-((4-(2-hydroxyethyl)phenyl)amino)-2-phenylacetate (3hm)

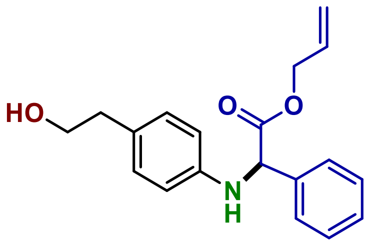

^{1}H-NMR (500 MHz, $CDCl_3$) δ p.p.m. 7.53 – 7.48 (m, 2H), 7.39 – 7.28 (m, 3H), 6.98 (d, J = 8.2 Hz, 2H), 6.52 (d, J = 8.2 Hz, 2H), 5.82 (ddt, J = 16.5, 10.9, 5.6 Hz, 1H), 5.18 (q, J = 1.4 Hz, 1H), 5.15 (dq, J = 8.9, 1.4 Hz, 1H), 5.08 (s, 1H), 4.69 – 4.56 (m, 2H), 3.76 (t, J = 6.5 Hz, 2H), 2.72 (t, J = 6.5 Hz, 2H); **^{13}C-NMR** (126 MHz, $CDCl_3$) δ 171.69, 144.75, 137.76, 131.52, 129.99, 129.02, 128.48, 127.78, 127.40, 118.62, 113.82, 66.26, 64.00, 61.08, 38.39; **HRMS (ESI)** calcd. for $[M+H]^+$: 312.1594 m/z, found: 312.1590 m/z.

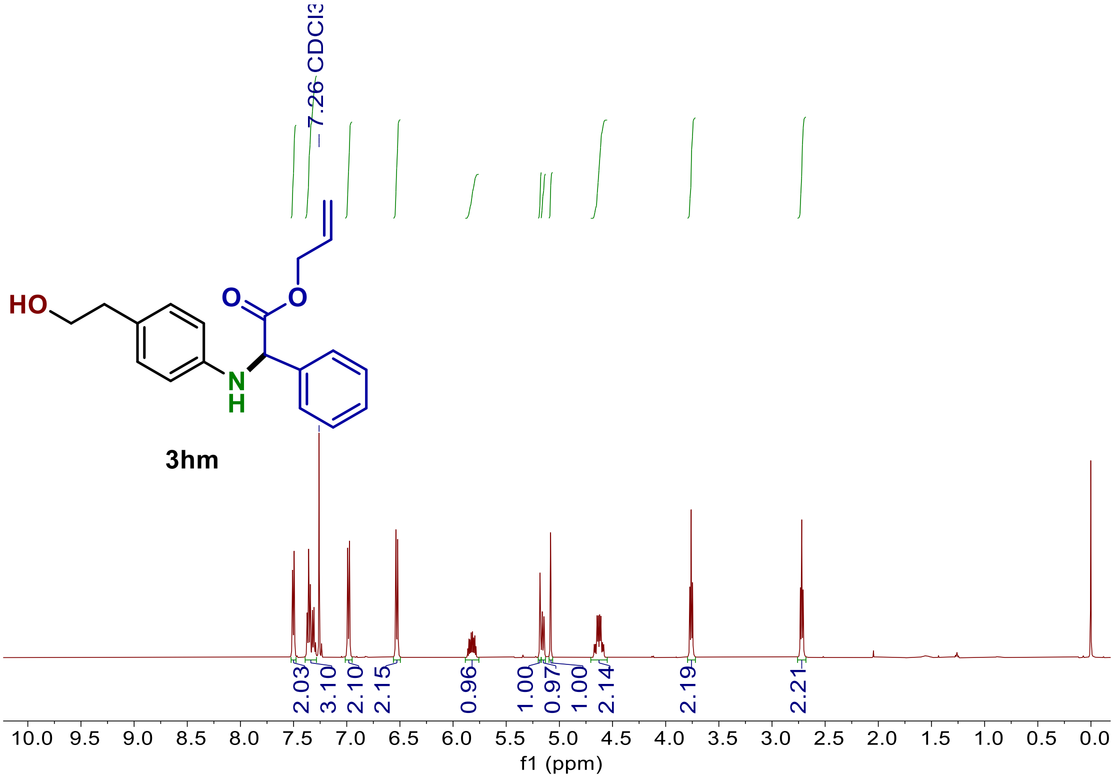

Figure S201. ^{1}H-NMR spectra of 3hm.

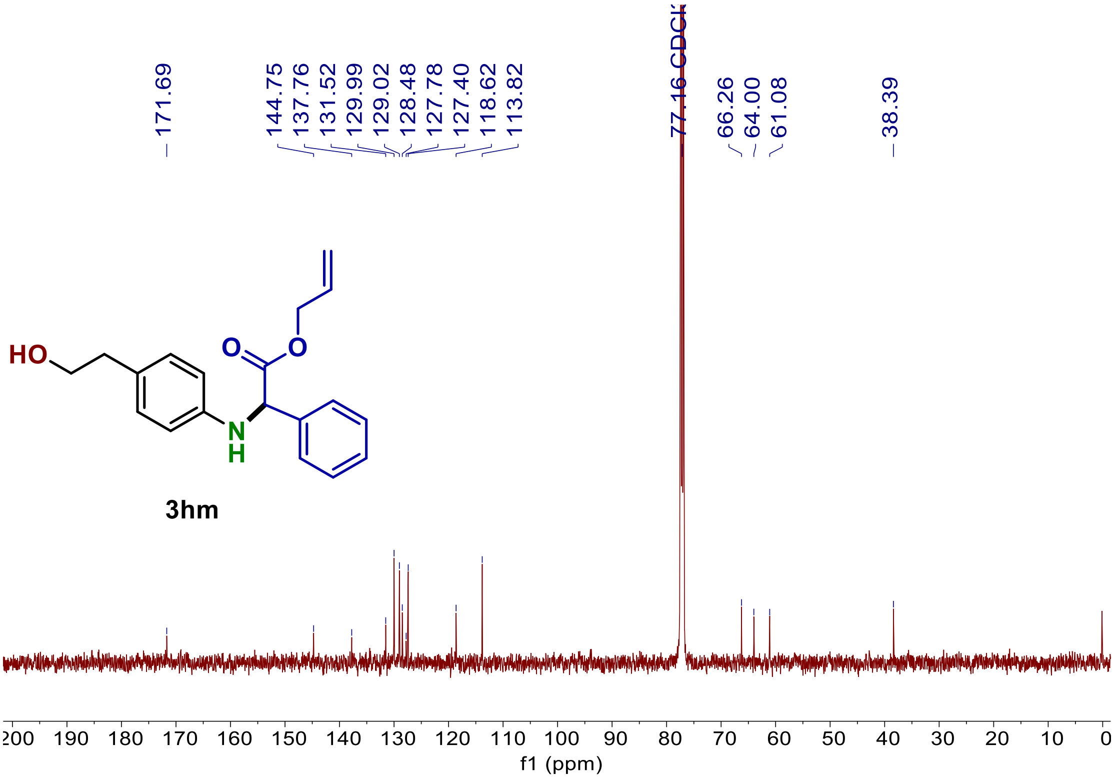

Figure S202. ^{13}C-NMR spectra of 3hm.

Allyl (R)-2-((4-(2-((S)-2-(allyloxy)-2-oxo-1-phenylethoxy)ethyl)phenyl)amino)-2 phenylacetate (4hm)

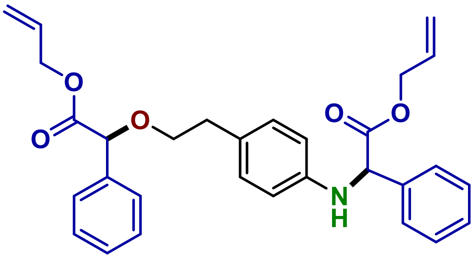

^{1}H NMR (500 MHz, $CDCl_3$) δ p.p.m. 7.52 – 7.47 (m, 2H), 7.41 (dt, *J* = 7.4, 1.9 Hz, 2H), 7.38 – 7.27 (m, 6H), 6.95 (d, *J* = 8.4Hz, 2H), 6.48 (d, *J* = 8.4Hz, 2H), 5.82 (ddtd, *J* = 16.3, 11.0, 5.6, 1.7 Hz, 2H), 5.21 – 5.12 (m, 4H), 5.07 (s, 1H), 4.86 (d, *J* = 3.7 Hz, 1H), 4.68 – 4.53 (m, 4H), 3.70 – 3.62 (m, 1H), 3.54 (td, *J* = 8.8, 6.6 Hz, 1H), 2.90 – 2.75 (m, 2H); **^{13}C-NMR** (126 MHz, $CDCl_3$) δ 171.70, 170.72, 144.53, 137.80, 136.65, 131.77, 131.53, 129.90, 128.99, 128.73, 128.69, 128.44, 127.94, 127.39, 127.33, 118.59, 118.50, 113.61, 81.30, 71.33, 66.22, 65.67, 61.10, 35.44; **HRMS (ESI)** calcd. for $[M+H]^{+}$: 486.2275 m/z, found: 486.2255m/z.

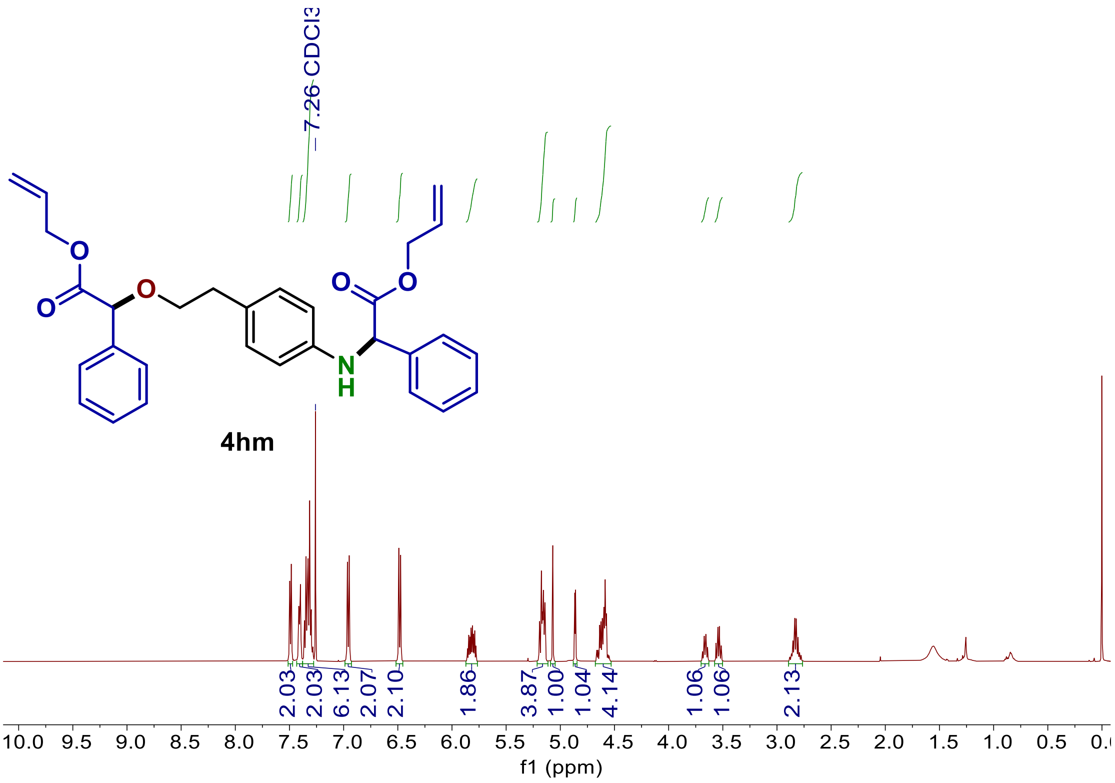

Figure S203. ^{1}H-NMR spectra of 4hm.

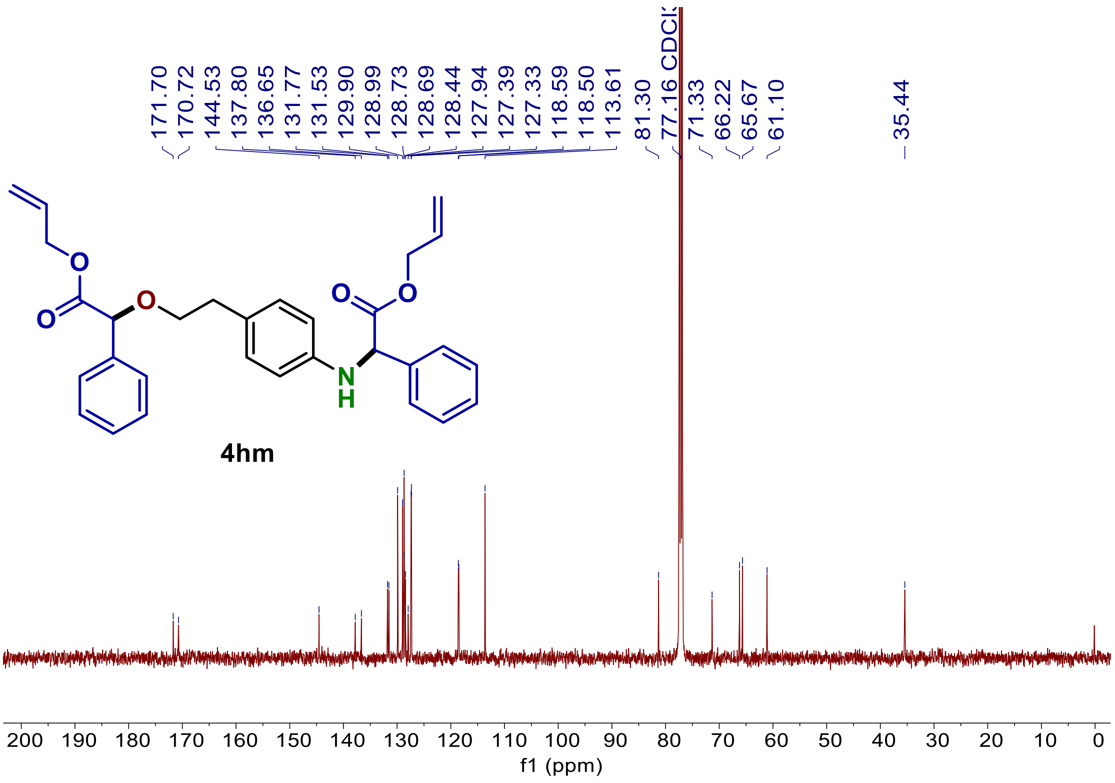

Figure S204. ^{13}C-NMR spectra of 4hm.

Methyl (R)-2-((4-(2-hydroxyethyl)phenyl)amino)-2-(naphthalen-2-yl)acetate (3im)

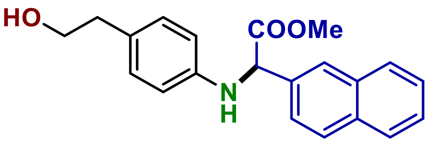

^{1}H-NMR (500 MHz, $CDCl_3$) δ p.p.m. 7.96 (s, 1H), 7.87 – 7.80 (m, 3H), 7.60 (dd, J = 8.5, 1.8 Hz, 1H), 7.52 – 7.45 (m, 2H), 6.97 (d, J = 8.2 Hz, 2H), 6.56 (d, J = 8.1 Hz, 2H), 5.22 (s, 1H), 5.04 (s, 1H), 3.73 (s,3H), 3.77-3.70 (m, 2H), 2.71 (t, J = 6.5 Hz, 2H); **^{13}C-NMR** (126 MHz, $CDCl_3$) δ 172.44, 144.73, 135.29, 133.50, 133.42, 130.00, 128.92, 128.20, 127.86, 127.82, 126.60, 126.50, 126.43, 125.06, 113.87, 63.98, 61.17, 53.03, 38.38; **HRMS (ESI)** calcd. for $[M+H]^{+}$: 336.1594 m/z, found: 336.1596 m/z.

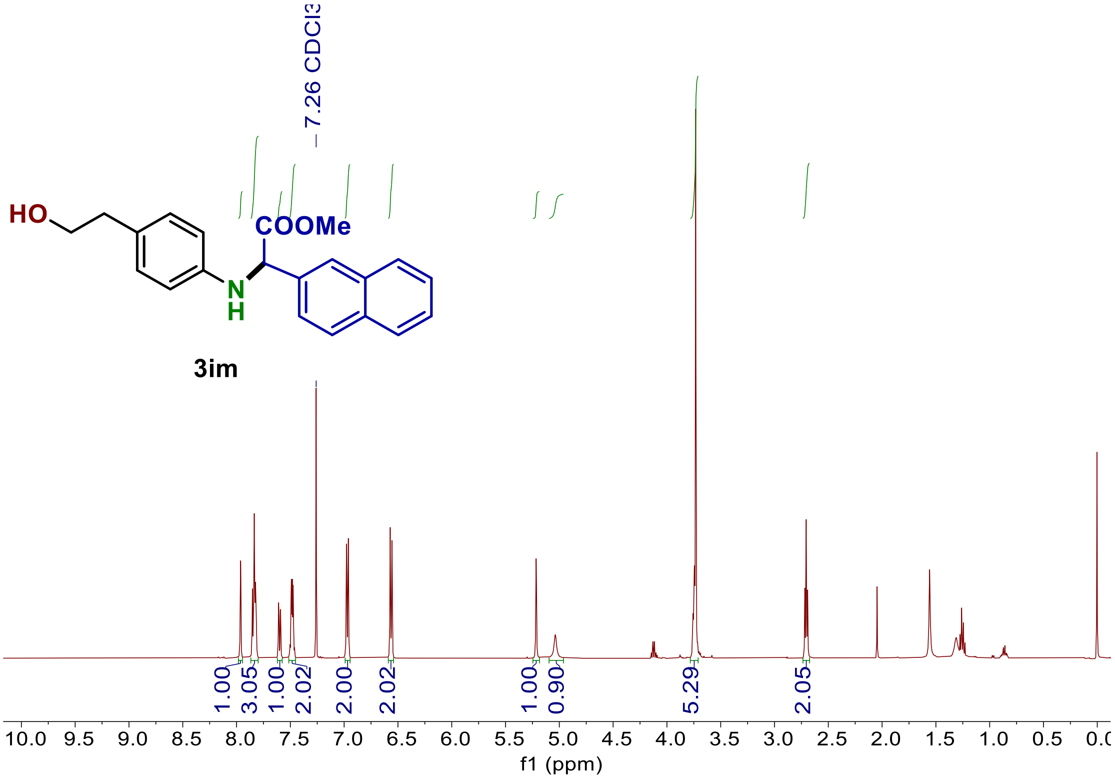

Figure S205. ^{1}H-NMR spectra of 3im.

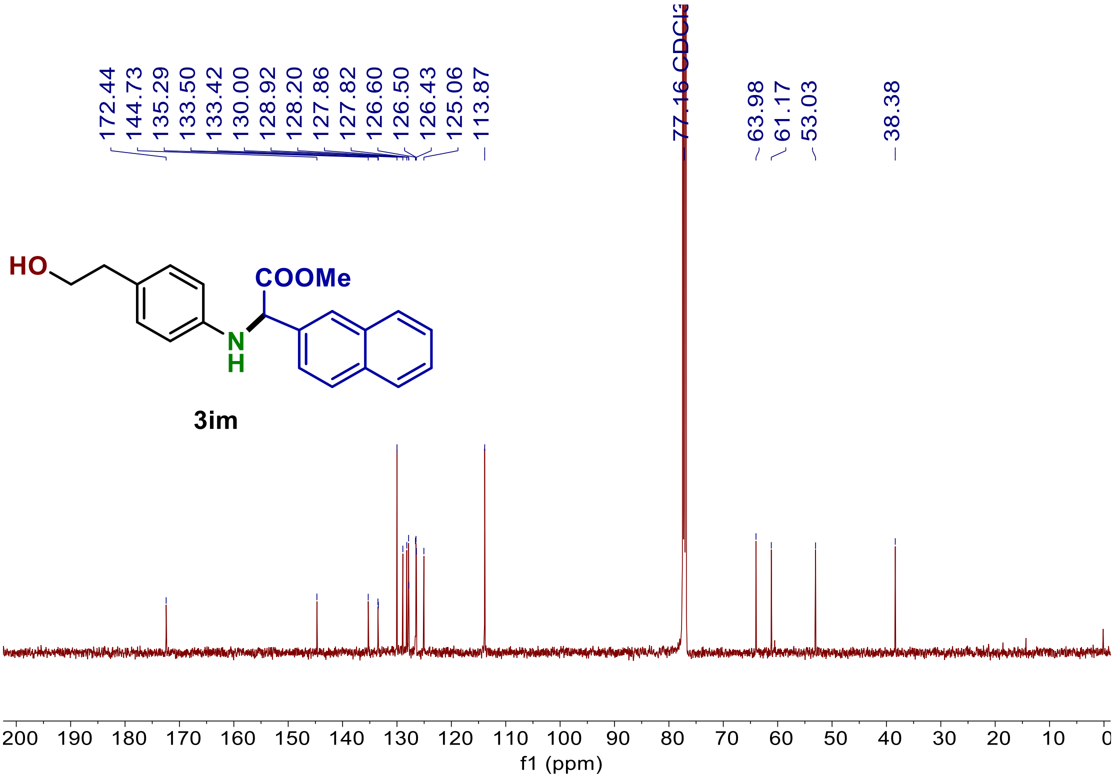

Figure S206. ^{13}C-NMR spectra of 3im.

Methyl (R)-2-((4-(2-((R)-2-methoxy-1-(naphthalen-2-yl)-2-oxoethoxy)ethyl)phenyl)amino)-2-(naphthalen-2-yl)acetate (4im)

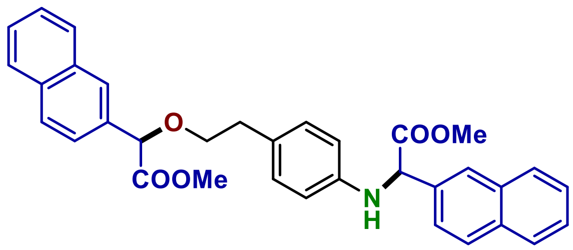

^{1}H-NMR (400 MHz, $CDCl_3$) δ p.p.m. 7.95 (d, J = 1.8 Hz, 1H), 7.80 (ddd, J = 16.3, 8.6, 4.8 Hz, 7H), 7.59 (dd, J = 8.5, 1.8 Hz, 1H), 7.47 (tddd, J = 6.9, 5.6, 3.9, 2.0 Hz, 5H), 6.94 (d, J = 8.1 Hz, 2H), 6.51 (d, J = 8.1 Hz, 2H), 5.20 (s, 1H), 4.99 (d, J = 2.8 Hz, 1H), 3.72 (d, J = 1.1 Hz, 3H), 3.69 – 3.62 (m, 1H), 3.67 (d, J = 3.9 Hz, 3H), 3.55 (td, J = 8.7, 6.7 Hz, 1H), 2.84 (h, J = 6.6 Hz, 2H); **HRMS** (**ESI**) calcd. for $[M+H]^+$: 534.2275 m/z, found: 534.2284 m/z.

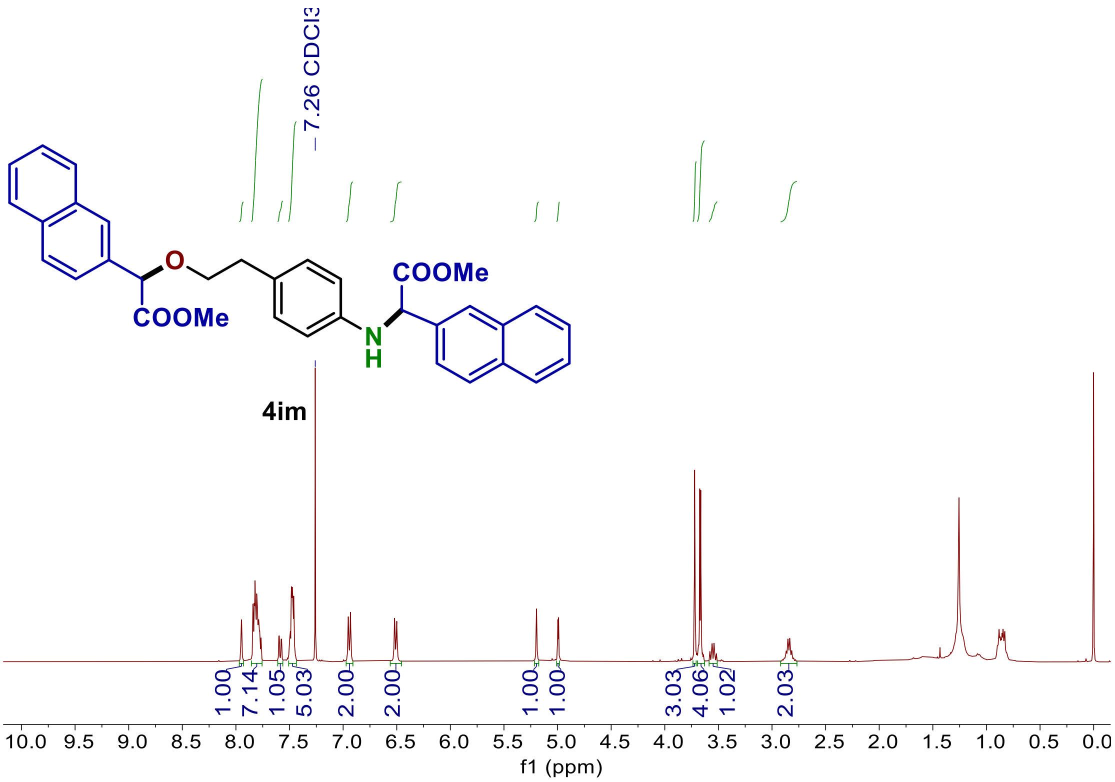

Figure S207. ^{1}H-NMR spectra of 4im.

Methyl (R)-2-((4-(2-hydroxyethyl)phenyl)amino)-2-(thiophen-3-yl)acetate (3jm)

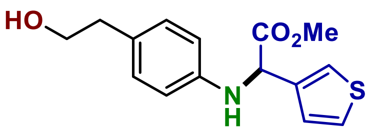

^{1}H-NMR (400 MHz, CDCl$_3$) δ p.p.m 7.34 (dt, J = 2.1, 1.1 Hz, 1H), 7.31 (dd, J = 5.0, 3.0 Hz, 1H), 7.16 (dd, J = 5.0, 1.4 Hz, 1H), 7.01 (d, J = 8.4 Hz, 2H), 6.56 (d, J = 8.4 Hz, 2H), 5.18 (s, 1H), 3.77 (t, J = 6.5 Hz, 2H), 3.76 (s, 3H), 2.74 (t, J = 6.5 Hz, 2H); **^{13}C-NMR** (126 MHz, CDCl$_3$) δ 172.24, 144.87, 138.32, 130.04, 128.12, 126.66, 126.61, 123.13, 113.87, 63.99, 57.19, 52.94, 38.40; **HRMS (ESI)** calcd. for $[M+H]^+$: 292.1002 m/z, found: 292.0995 m/z.

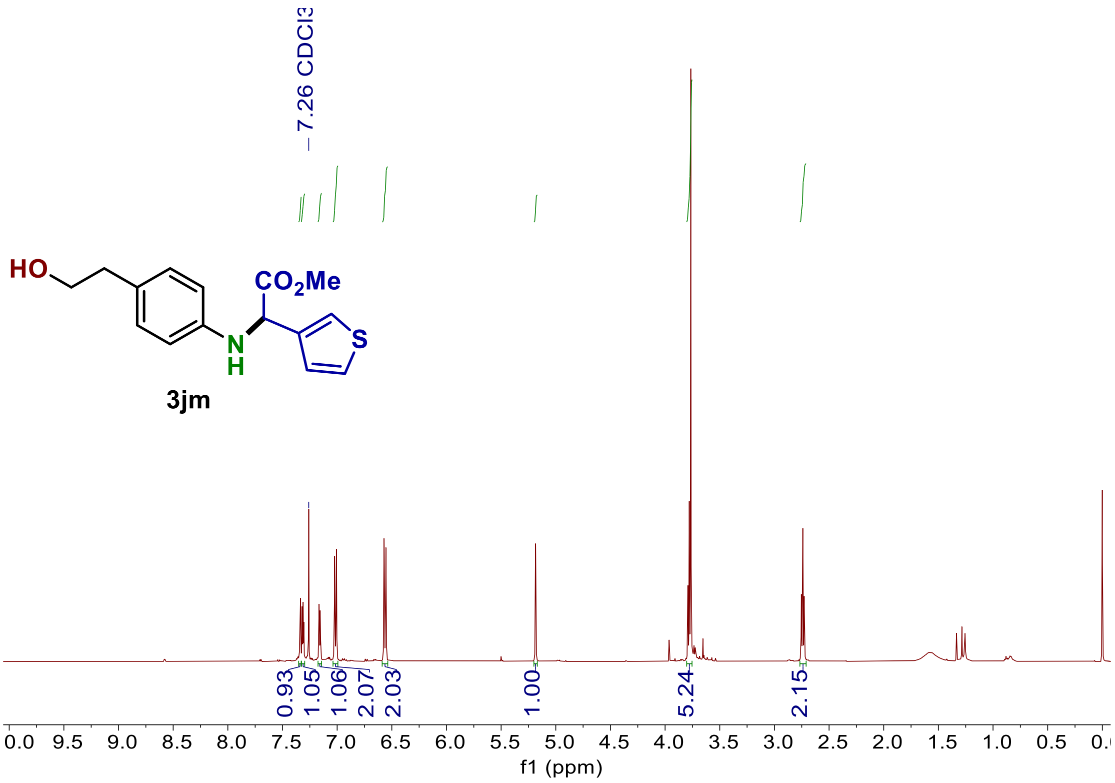

Figure S208. ^{1}H-NMR spectra of 3jm.

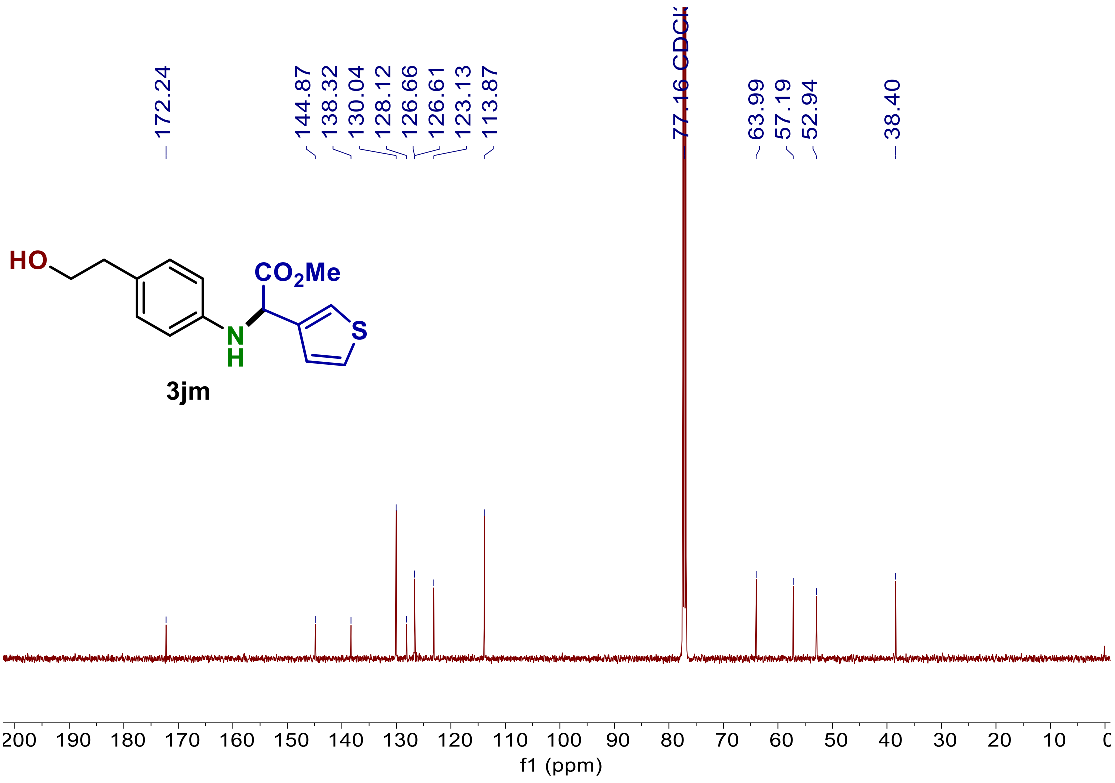

Figure S209. ^{13}C-NMR spectra of 3jm.

Methyl (R)-2-((4-(2-((S)-2-methoxy-2-oxo-1-(thiophen-3-yl)ethoxy)ethyl)phenyl)amino)-2-(thiophen-3-yl)acetate (4jm)

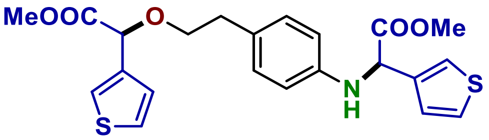

^{1}H-NMR (500 MHz, $CDCl_3$) δ p.p.m. 7.34 – 7.32 (m, 1H), 7.32 – 7.27 (m, 3H), 7.15 (dd, J = 4.9, 1.4 Hz, 1H), 7.09 (dt, J = 4.8, 1.3 Hz, 1H), 6.99 (d, J = 8.4 Hz, 2H), 6.52 (d, J = 8.4 Hz, 2H), 5.18 (s, 1H), 4.97 (d, J = 2.9 Hz, 1H), 3.76 (s, 3H), 3.73 (s, 3H), 3.72 – 3.66 (m, 1H), 3.60 – 3.53 (m, 1H), 2.89 – 2.79 (m, 2H); **^{13}C-NMR** (126 MHz, $CDCl_3$) δ 172.24, 171.19, 144.66, 138.32, 137.31, 129.94, 128.26, 126.62, 126.40, 126.32, 123.69, 123.12, 116.49, 113.72, 77.53, 71.41, 57.23, 52.92, 52.44, 35.43; **HRMS (ESI)** calcd. for $[M+H]^+$: 446.1090 m/z, found: 446.1089 m/z.

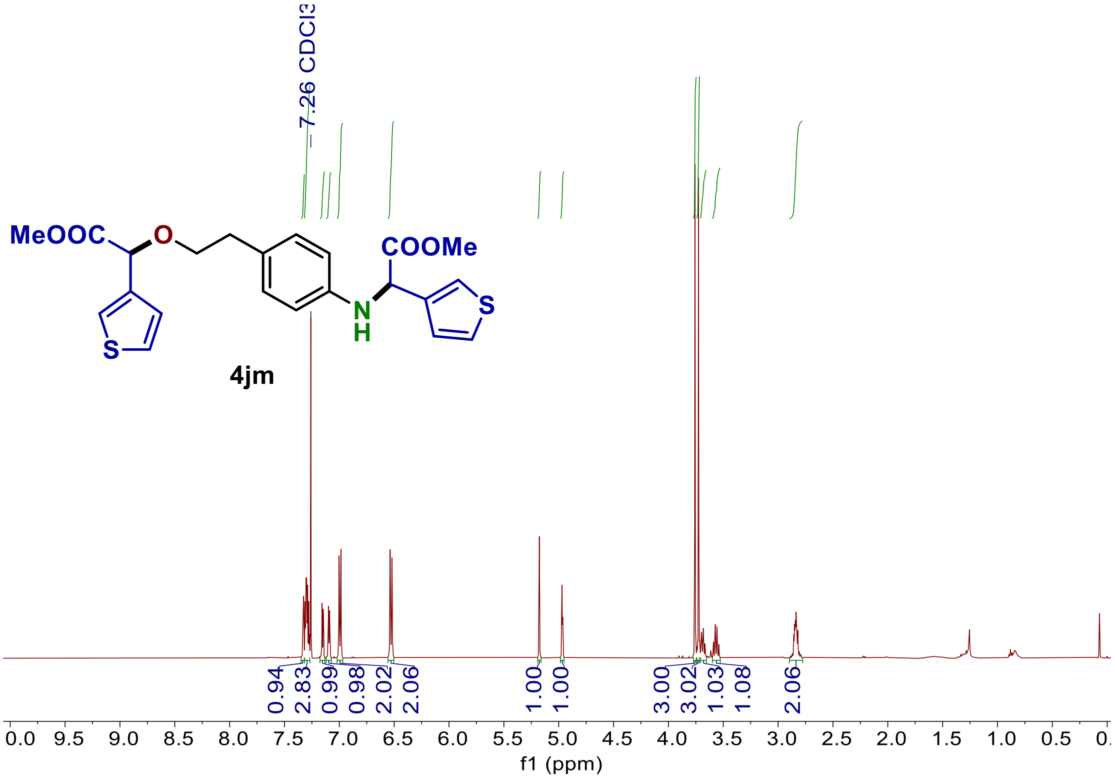

Figure S210. ^{1}H-NMR spectra of 4jm.

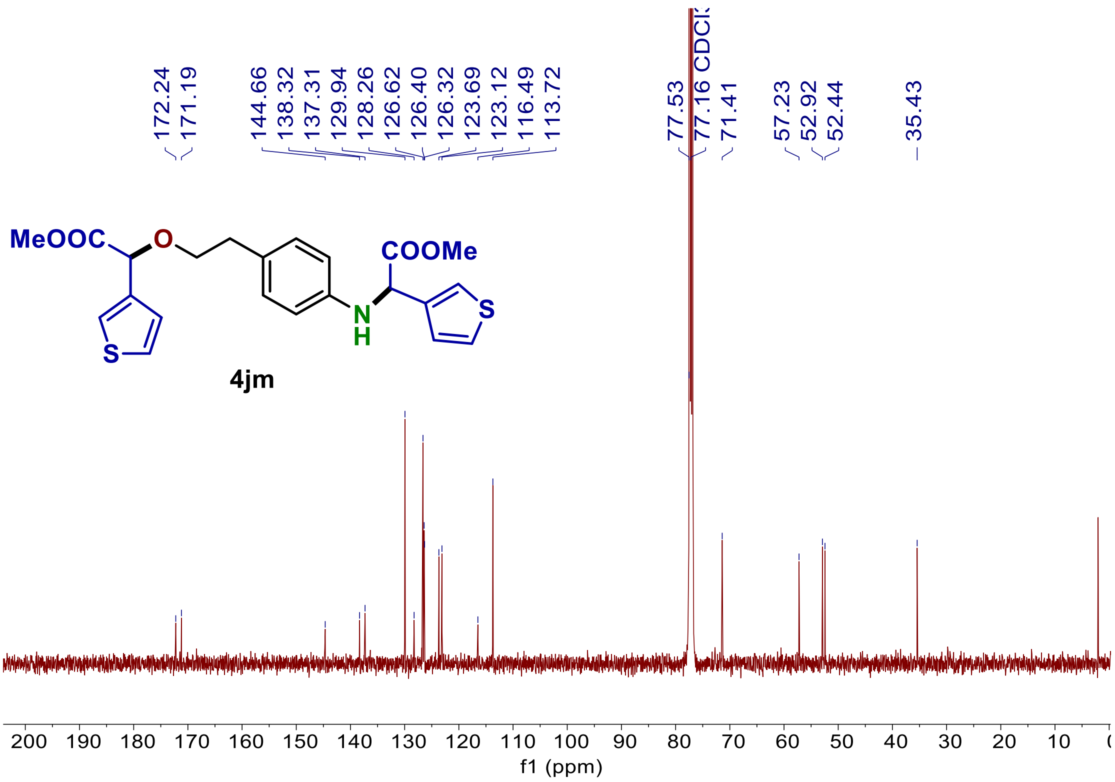

Figure S211. ^{13}C-NMR spectra of 4jm.

Benzyl (S)-2-((4-(2-hydroxyethyl)phenyl)amino)butanoate (**3km**)

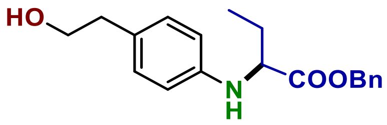

^{1}**H-NMR** (500 MHz, $CDCl_3$) δ p.p.m. 7.39 – 7.27 (m, 5H), 7.02 (d, J = 8.2 Hz, 2H), 6.59 (d, J = 8.4 Hz, 2H), 5.15 (d, J = 1.8 Hz, 2H), 4.05 (t, J = 6.3 Hz, 1H), 3.79 (t, J = 6.5 Hz, 2H), 2.75 (t, J = 6.5 Hz, 2H), 1.95 – 1.75 (m, 2H), 0.98 (t, J = 7.4 Hz, 3H); ^{13}**C-NMR** (126 MHz, $CDCl_3$) δ 174.06, 145.48, 135.69, 130.05, 128.70, 128.50, 128.40, 128.12, 114.05, 66.92, 64.01, 58.26, 38.41, 26.22, 10.09; **HRMS (ESI)** calcd. for $[M+H]^+$: 314.1751 m/z, found: 314.1736 m/z.

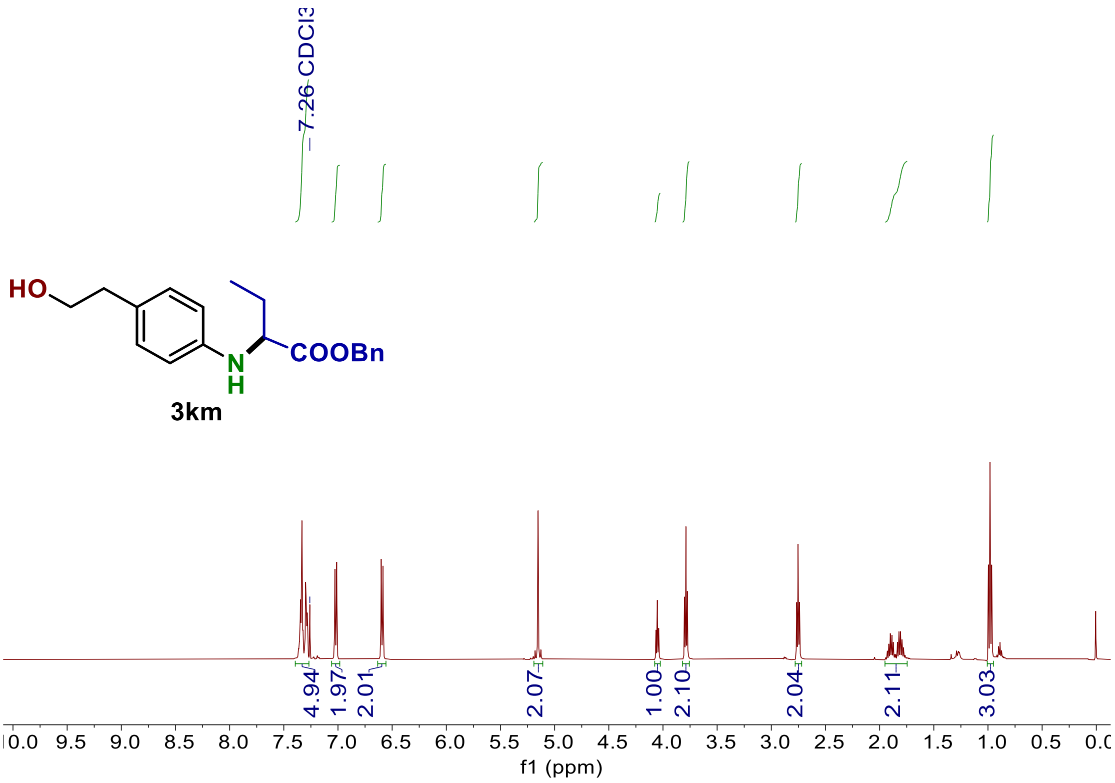

Figure S212. ^{1}H-NMR spectra of 3km.

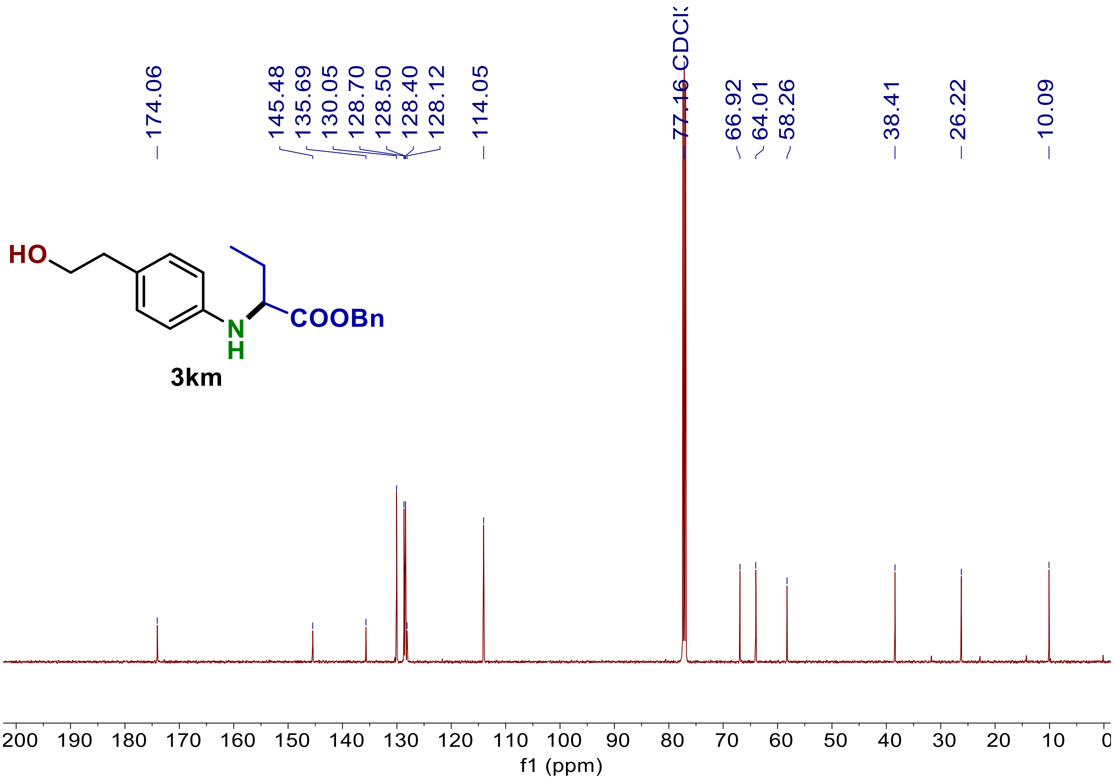

Figure S213. ^{13}C-NMR spectra of 3km.

Benzyl (R)-2-(4-(((S)-1-(benzyloxy)-1-oxobutan-2-yl) amino) phenethoxy) butanoate (4km)

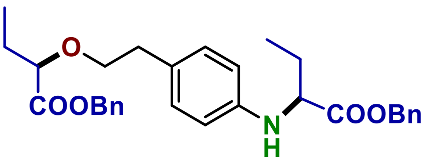

^{1}H-NMR (500 MHz, $CDCl_3$) δ p.p.m. 7.38 – 7.24 (m, 10H), 6.98 (d, *J* = 8.4 Hz, 2H), 6.53 (d, *J* = 8.4 Hz, 2H), 5.23 – 5.12 (m, 4H), 4.08 –4.0 (m, 2H), 3.84 – 3.70 (m, 2H), 3.45 (q, *J* = 8.2 Hz, 1H), 2.79 (t, *J* = 7.3 Hz, 2H), 1.88 (dt, *J* = 13.9, 6.9 Hz, 1H), 1.83 – 1.69 (m, 3H), 0.97 (t, *J* = 7.4 Hz, 3H), 0.92 (t, *J* = 7.4 Hz, 3H); **^{13}C-NMR** (126 MHz, $CDCl_3$) δ 174.16, 172.99, 145.40, 135.74, 129.94, 128.72, 128.48, 128.46, 128.43, 128.39, 128.33, 113.74, 80.59, 72.07, 66.89, 66.54, 58.24, 35.49, 26.37, 26.28, 10.09, 9.84; **HRMS (ESI)** calcd. for $[M+H]^+$: 490.2588 m/z, found: 490.2570m/z.

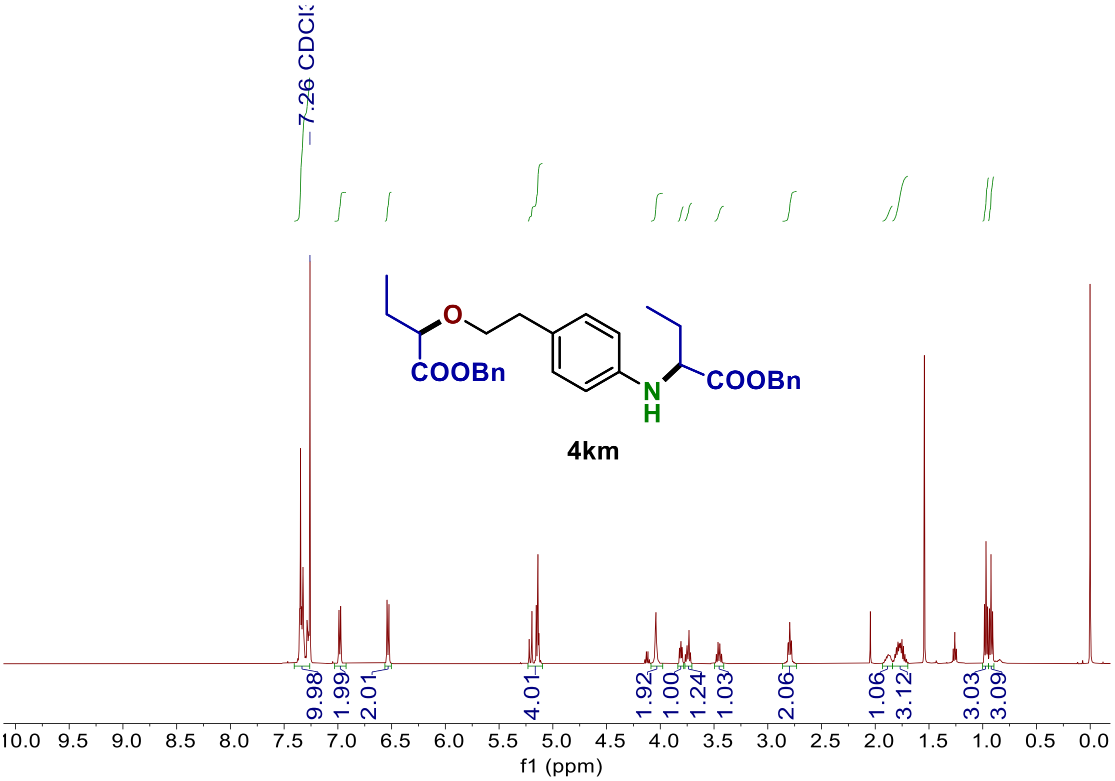

Figure S214. ^{1}H-NMR spectra of 4km.

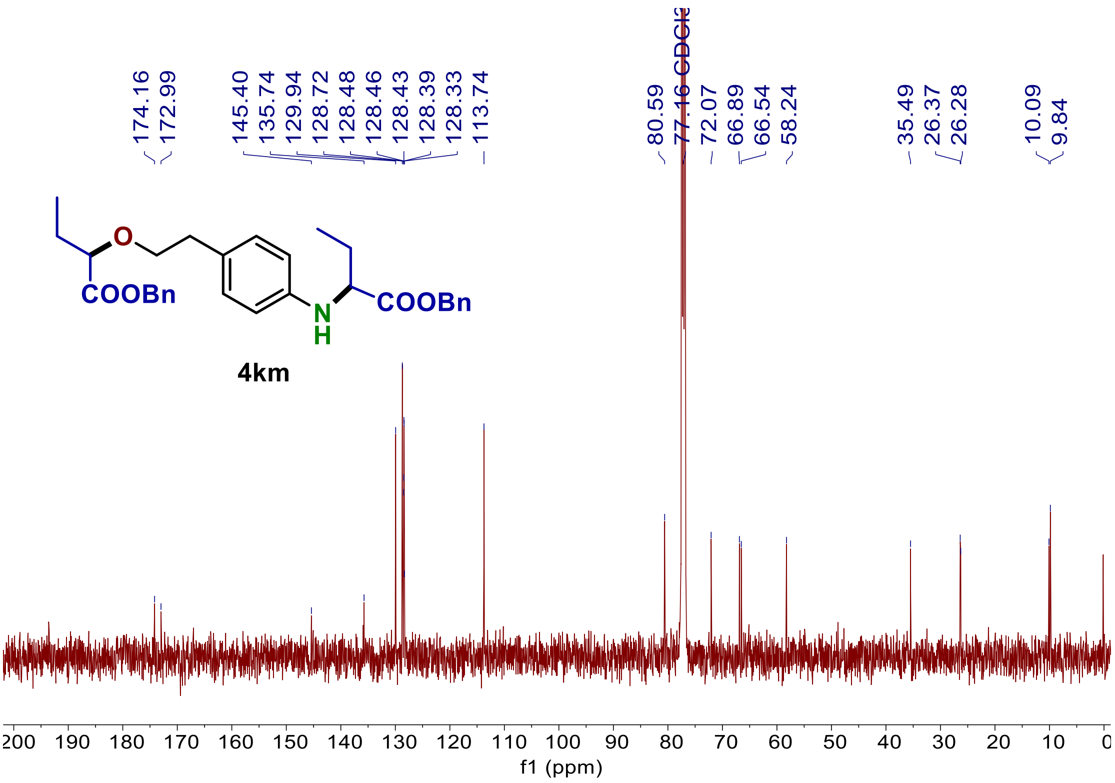

Figure S215. ^{13}C-NMR spectra of 4km.

Supplementary references

(1) Fan, Q. G.; Li, P. X. Visible-light-mediated 1,2-acyl migration: the reaction of secondary enamino ketones with singlet oxygen. *Angew. Chem. Int. Ed.* **2014**, *53*, 12201-12204.

(2) Hu, S.; Wu, J.; Lu, Z.; Wang, J.; Tao, Y.; Jiang, M.; Chen, F. TfOH-catalyzed N-H insertion of α-substituted-α-diazoesters with anilines provides access to unnatural α-amino esters. *J Org Chem*. **2021**, *86*, 3223-3231.

(3) Mangion, I. K.; Nwamba, I. K.; Shevlin, M.; Huffman, M. A. Iridium-catalyzed X−H insertions of sulfoxonium ylides. *Org. Lett.* **2009**, *11*, 3566-3569.

(4) Loskutova, N. L.; Shvydkiy, N. V.; Nelyubina, Y. V.; Perekalin, D. S. Insertion of carbenoids into X-H bonds catalyzed by the cyclobutadiene rhodium complexes. *J Organomet Chem*, **2018**, *867*, 86-91.

(5) Alcaide, B.; Plumet, J.; Sierra, M. A. One-pot synthesis of N-(2-heteroaryl)-.alpha.-amino esters by the regiospecific 2-N-(.alpha.-alkoxycarbonyl)alkylation of 2-aminoazines and -azoles with glyoxals and alcohols promoted by perchloric acid. *J. Org. Chem.* **1990**, *55*, 3143-3147.

(6) Zhao, J.; Ji, S. F.; Guo, C. X.; Li, H.; Dong, J. C.; Guo, P.; Wang, D. S.; Li, Y. D.; Toste, F. D. A heterogeneous iridium single-atom-site catalyst for highly regioselective carbenoid O-H bond insertion. *Nat. Catal.* **2021**, *4*, 523-531.

(7) Wu, W; Zhao, H. Y.; Chen, J. C.; Zhang, F. Q.; Fan, B. M. Umpolung reactivity of imine ester: visible-light mediated transfer hydrogenation of α-aryl imino esters by phenylsilane and water. *Chem.Eur. J.* **2022**, *28*, e202202460(1-5).

(8) Wang, L. H.; Perveen, S.; Ouyang, Y. Z.; Zhang, S.; Jiao, J.; He, G.; Nie, Y.; Li, P. F. Well-defined, versatile and recyclable half-sandwich nickelacarborane catalyst for selective carbene-transfer reactions. *Chem.Eur.J.* **2021**, *27*, 5754-5760.

(9) Kresse, G.; Furthmuller, J. Efficient iterative schemes for ab initio total-energy calculations using a plane-wave basis set. *Phys. Rev. B*, **1996**, *54*, 11169.

(10) Blochl, P. E. Projector augmented-wave method. *Phys. Rev. B,* **1994**, *50*, 17953-17979.

(11) Perdew, J. P.; Burke, K.; Ernzerhof, M. Generalized gradient approximation made simple. *Phys. Rev. Lett.,* **1996**, *77*, 3865.

(12) Henkelman, G.; Uberuaga, B. P.; Jonsson, H. A climbing image nudged elastic band method for finding saddle points and minimum energy paths. *J. Chem. Phys.* **2000**, *113*, 9901-9904.
