# Supplementary material for: Heterogeneous Rhodium Single-Atom-Site Catalyst Enables Chemoselective Carbene N–H Bond Insertion
Source: J Am Chem Soc. 2024 Apr 7;146(15):10847–56. doi: 10.1021/jacs.4c01408 (PMC11027138; doi:10.1021/jacs.4c01408)
Supplement: Supplementary file 2 — ja4c01408_si_002.pdf [file ja4c01408_si_002.pdf]

DFT Information for:

Heterogeneous Rhodium Single-Atom Site Catalyst Enables Chemoselective Carbene N-H Bond Insertion

Yuanjun Chen[2‡], Ruixue Zhang[1,‡], Zhiwen Chen[3,‡], Jiangwen Liao[4], Xuedong Song[1], Xiao Liang[2], Yu Wang[5], Juncai Dong[4], Chandra Veer Singh[3], Dingsheng Wang[2,*], Yadong Li[2,*], F. Dean Toste[6,7*], Jie Zhao[1,*]

‡These authors contributed equally to this work
Correspondence to: zhaojie@ecust.edu.cn
fdtoste@berkeley.edu
ydli@mail.tsinghua.edu.cn
wangdingsheng@mail.tsinghua.edu.cn

RhN$_3$P SA (pyrrolic N): IS0
1.00000000000000
19.7297992706000009 0.0000000000000000 0.0000000000000000
-9.8648996353000005 17.0865073798999987 0.0000000000000000
0.0000000000000000 0.0000000000000000 15.0000000000000000
C N P Rh H O
131 5 1 1 8 2
Direct

0.0014536089272737	0.0019305785233493	0.0779098279490428
0.0421433643810332	0.0847894583388226	0.0819801755773857
0.1262896887918122	0.0006762128189785	0.0819944375139268
0.1670958516760819	0.0836919728179623	0.0842740234681800
0.2509579536487327	0.9997537188001656	0.0948144891986008
0.2922343933645153	0.0830120581053967	0.0969556277762163
0.3756835226711132	0.9993215535389982	0.1096460691506849
0.4168858873656429	0.0827395860460113	0.1129274051927751
0.5006627449600047	0.9994381813326599	0.1179916230851772
0.5417019694442125	0.0831683350967668	0.1204122718044326
0.6256427179639344	0.9990305410284438	0.1165398874548989
0.6666146856568851	0.0830299212042896	0.1174291495351714
0.7503752471035195	0.0004119224002756	0.1045210913991979
0.7915217975843958	0.0840572781206406	0.1056928961119787
0.8754506516474785	0.0014976704047797	0.0867565908168522
0.9166549091661742	0.0847332906843505	0.0905882887131744
0.9999659923087024	0.1263331561748421	0.0877679300914902
0.0411943049965591	0.2094349071855807	0.0947646581992369
0.1251674941481053	0.1256063282080044	0.0842150192121997
0.1659454675560958	0.2085040159342004	0.0913042514668691
0.2499931084876358	0.1244889454152790	0.0914772231549145
0.2903603210032862	0.2069899233019403	0.0977447947294366
0.3750928027513051	0.1242784131714840	0.1081198705967579
0.4158285591196088	0.2072772404784498	0.1170194903031818
0.4998693294416967	0.1250457552518972	0.1208113817614058
0.5407088111285706	0.2087176847395526	0.1290785716929573
0.6249324768057355	0.1250574709005041	0.1218552977947120
0.6659352707489495	0.2088546754185620	0.1265255621791105
0.7498249598616715	0.1256641754371325	0.1136966090848720
0.7908867538005211	0.2093619471173432	0.1170214223804506
0.8747284938009690	0.1261075602574538	0.0998821329514313
0.9159585230205033	0.2094524519837189	0.1046907562796813
0.9993535530397566	0.2510369939679781	0.1025484941016174
0.0407733002761543	0.3341581080223192	0.1095833056009397
0.1244549603880953	0.2507258178159680	0.0967047332688003
0.1657221125026529	0.3335886463649259	0.1077088018935985

0.2484390828547381	0.2488901012510161	0.0976477204396979
0.2884061466104276	0.3309944289373358	0.1112839850531929
0.3724691807826931	0.2469967521689345	0.1114313767414583
0.4111807601926307	0.3276716438896576	0.1281248686805829
0.4984560382001643	0.2503594481478367	0.1321693919694878
0.5382988848637215	0.3336372643618534	0.1512948833898528
0.6239598444906402	0.2505089979859902	0.1330028788707473
0.6645860709809045	0.3341614177193198	0.1415210476184365
0.7491803819432138	0.2511236856454030	0.1245962516423440
0.7901310466305387	0.3350793754442848	0.1278847685189951
0.8742163275542475	0.2510876491880003	0.1130051359362442
0.9155008388609007	0.3345846532959038	0.1166453006522112
0.9989858916996368	0.3758917591494640	0.1147652718694007
0.0409505928375384	0.4591663251991678	0.1178111283623831
0.1241935663454841	0.3753709254766049	0.1125360168024752
0.1665218965153872	0.4583700272090463	0.1201904504334110
0.2487222885119887	0.3743410838397357	0.1165967456655261
0.2918176188902466	0.4569945355553687	0.1315977036349989
0.3690938700207916	0.3697572247875694	0.1280930069348993
0.4108800515377859	0.4485177830914700	0.1556287112723561
0.4899512746625082	0.3695311721470155	0.1557080219380376
0.6221166945189770	0.3741712030435903	0.1559833055214261
0.6682692482376756	0.4587976285638211	0.1717237751139834
0.7486062813819349	0.3769790467973536	0.1360906106016645
0.7902723071462594	0.4613707111948712	0.1334581094051580
0.8737430658210371	0.3764717822927502	0.1225024017029591
0.9153553354703141	0.4599397325349869	0.1217482020280241
0.9989709435199648	0.5009538025828728	0.1187181486648444
0.0405783651957711	0.5841814306736030	0.1161633954107967
0.1246767672235749	0.5002037938155314	0.1198218540192034
0.1665660695097763	0.5834460342957587	0.1209911643479469
0.2501786738129449	0.4992139491437679	0.1282624852740810
0.2919550207632068	0.5824638132998913	0.1318766501866383
0.3750018884406756	0.4968195304871857	0.1511208222441726
0.4154396215573263	0.5807093707027617	0.1559674665253664
0.7492108632102038	0.5040817782443916	0.1403068684161691
0.7857324713180341	0.5875112671631105	0.1261117441972741
0.8734573433872885	0.5020444268226395	0.1238647046368148
0.9139565084610004	0.5852210402338909	0.1166049367780106
0.9983747112953864	0.6258897535606216	0.1121625908502260
0.0419669240469529	0.7089208812454412	0.1040690884707151
0.1245618439525932	0.6251386061841226	0.1166986130402428
0.1672067590173111	0.7083513163312087	0.1128672979508248
0.2503418184125805	0.6244354085589814	0.1253788839833981

0.2926446562788806	0.7076936156379259	0.1234600729279280
0.3755337531565732	0.6230604059977543	0.1405875024451355
0.4184508504261054	0.7070723777937958	0.1350785373225852
0.4998542866692893	0.6266685684957184	0.1723553242490516
0.5454205906812923	0.7072735955580940	0.1400208400135936
0.6793688000449346	0.7074005628624320	0.1200572197196261
0.7488767865929402	0.6378229364092297	0.1200185652351193
0.7982631811780589	0.7156198124028654	0.0939737458078301
0.8711267619782880	0.6271501046449621	0.1123957819857764
0.9174202904956297	0.7097574748708556	0.0965773025427772
0.0008134735483903	0.7506454544526806	0.0947705879962645
0.0430217850285305	0.8339871902529866	0.0865128501541097
0.1256049180700951	0.7500561705163619	0.1050608793576008
0.1676560408333180	0.8332777729550709	0.0993906712120019
0.2509005642812606	0.7494069463753289	0.1160553544714347
0.2926331421011918	0.8327474120936762	0.1122723519761737
0.3765673961174433	0.7486240249364132	0.1268157744672723
0.4179731607156141	0.8322242133462816	0.1217322463428903
0.5027612676525179	0.7485629333906032	0.1327088140100896
0.5434707892096617	0.8317895851907299	0.1233842678813015
0.6289563094862218	0.7440975653469071	0.1260551924653231
0.6685815707444477	0.8295092044020643	0.1124725244589260
0.7571777328404238	0.7567753034423735	0.0940346193283918
0.7953561599792854	0.8379113295498570	0.0859740062727169
0.8794064789177718	0.7538342952158160	0.0857912284150986
0.9198167426987828	0.8363797268093682	0.0777165080168869
0.0024667798042086	0.8768692162656363	0.0789748486052763
0.0434229638963475	0.9599673881274159	0.0778869228364037
0.1262634116301035	0.8751972818804150	0.0903228680860629
0.1678546137940493	0.9585199627106493	0.0877090068178730
0.2509948062166622	0.8745103277385896	0.1042151037458452
0.2925735869437062	0.9579080608866233	0.1024242430533281
0.3760983057659377	0.8740153815953985	0.1160492037900584
0.4174102835897743	0.9575106213192360	0.1146364255609751
0.5014106114026146	0.8737710705873756	0.1213093359716201
0.5424387066272373	0.9574233474584479	0.1187692624010219
0.6266591385721247	0.8723735384100928	0.1165819035949827
0.6673493202255107	0.9568068701475407	0.1124668146018861
0.7512377977235991	0.8758724818723828	0.0968293549699813
0.7921142154252240	0.9592700585311537	0.0950918522407575
0.8778927583708453	0.8783264286823584	0.0777942946207720
0.9183530969394971	0.9609653428450432	0.0790751960776091
0.5880024931466192	0.5518717422129967	0.4314906951275866
0.6392417592074431	0.5172995431154247	0.4391667268733183

0.6087304163058687	0.4367485359270146	0.4236039262494861
0.6565511470783006	0.4036445578371543	0.4290201260833119
0.7359158352070535	0.4500827989199986	0.4497140263489214
0.7665066074106458	0.5298703954575044	0.4658261277232351
0.7189442461557072	0.5634545438236258	0.4611775849121085
0.6068259641758699	0.6343729397322215	0.4236004170831263
0.7017776391643842	0.7617270913199343	0.3779588693852196
0.5372281203451287	0.6039509032392684	0.2243650879690591
0.6752973692001177	0.6338605529898794	0.1373598124984431
0.6459569045943622	0.4973132998886437	0.2218606640682972
0.5118819290654496	0.5038793230021184	0.4403983428956619
0.4460892932378321	0.4608922945836456	0.4431766721634724
0.4942235807071184	0.4527897958984891	0.2078414165312805
0.6031661662557496	0.5621200160200368	0.2278451417824674
0.7438815388541647	0.6257553998924759	0.4736806058969040
0.8282501128907861	0.5667842355902495	0.4826418643852223
0.7734983502326417	0.4243116278592639	0.4533296639911643
0.6316821214351900	0.3412770551187729	0.4158777686092592
0.5473800842810160	0.3997145841832598	0.4051057243376898
0.6668694490749404	0.7632642814843480	0.3217731468028075
0.7640585560190161	0.7923563236089068	0.3609159898067982
0.6907302343471269	0.7876971931593961	0.4368948227327132
0.5600384998393658	0.6567177142336764	0.4372247612674138
0.6819496884805178	0.6817724092154387	0.3957802345283628

Total energy (E): -1297.34981241 eV
Temperature (T): 333.0 K

Zero-point energy E_ZPE	:	96.736 kcal/mol	4.194876 eV
Thermal correction to U(T):		106.131 kcal/mol	4.602266 eV
Thermal correction to H(T):		106.131 kcal/mol	4.602266 eV
Thermal correction to G(T):		86.996 kcal/mol	3.772518 eV
Entropy S	:	240.416 J/(mol*K)	0.002492 eV/K
Entropy contribution T*S	:	80058.421 J/(mol)	0.829747 eV

RhN3P SA (pyrrolic N): TS0 (imaginary frequency: 310.335797 cm^{-1})
1.00000000000000

19.7297992706000009	0.0000000000000000	0.0000000000000000
-9.8648996353000005	17.0865073798999987	0.0000000000000000
0.0000000000000000	0.0000000000000000	15.0000000000000000

C	N	P	Rh	H	O
131	5	1	1	8	2

Direct

0.0013358662422672	0.0019563851587396	0.0797660675973536
0.0421664030021989	0.0848665973290773	0.0830810986914133

0.1262998301213714	0.0009739547252559	0.0830802164784034
0.1672414947610945	0.0840489661003564	0.0855976165163954
0.2510365514214749	1.0000446168935473	0.0953503652128992
0.2923625402883367	0.0834115165796674	0.0984043939651606
0.3756742499718753	0.9995244996128740	0.1098632184133863
0.4168721284932160	0.0829662658567552	0.1140241295597263
0.5005517472373022	0.9994918914590150	0.1181173451281455
0.5415733371481632	0.0832360460341675	0.1209290446595267
0.6254321397658424	0.9989512815835762	0.1168651627378812
0.6664211713839410	0.0830020226139321	0.1175365716559975
0.7500894626153870	0.0002479970241703	0.1055295425283649
0.7912637629511181	0.0839467382653633	0.1058990112709522
0.8752153142514513	0.0013127033882626	0.0887669787564692
0.9164784068175033	0.0846298691652169	0.0913705778103469
-0.0001885485503113	0.1263531956718918	0.0884612683161559
0.0410446257603989	0.2094475973936465	0.0951631060743915
0.1252662689023097	0.1259001873561661	0.0854758994319731
0.1661165239093432	0.2088450763703271	0.0930675218726736
0.2502276225535156	0.1250341865409893	0.0934231605888179
0.2907452733782064	0.2075873107153678	0.1010343247588233
0.3751272202281928	0.1246834558536801	0.1101119789753562
0.4158270022428963	0.2076256161600069	0.1204645409545321
0.4997694087296434	0.1251828191192079	0.1219671588460733
0.5406297828349542	0.2089246588479606	0.1310574892133348
0.6247639146966090	0.1250608787297639	0.1222567491593749
0.6657650500072874	0.2088739126904589	0.1270838039220661
0.7495793511530976	0.1255833306936341	0.1135758308274324
0.7906515352647155	0.2092891740607610	0.1166082256241450
0.8744668965352562	0.1260032655252598	0.0999994235317796
0.9157444761347796	0.2093896948443414	0.1043125883485679
0.9991452977361337	0.2509805320362789	0.1023686162717520
0.0405625228396297	0.3341022084716239	0.1092801246703187
0.1244150852826720	0.2508642911387971	0.0978463770557963
0.1656041382481457	0.3336373664580475	0.1090824986723878
0.2486548292051207	0.2494948618080731	0.1008850939166527
0.2885005953295339	0.3314432892042276	0.1154745992900181
0.3727565969917475	0.2476644327185897	0.1156866798814772
0.4116017200403626	0.3284706881463947	0.1338694108399095
0.4983882078600554	0.2506153029286231	0.1356420079733674
0.5384242005263569	0.3339401989538789	0.1549487308132123
0.6238200789659812	0.2506163901459765	0.1343265465996023
0.6644632231104259	0.3343846436868637	0.1423356241965466
0.7489412828128890	0.2510414594360852	0.1244530761993570
0.7899569235114317	0.3350770869335614	0.1275437518305959

0.8739729526393275	0.2510235186740956	0.1123755603523272
0.9152768162644239	0.3345161759770315	0.1158627982901330
0.9987935693140384	0.3758295186917843	0.1140595722085384
0.0408208515013023	0.4591159119763376	0.1170643701587283
0.1239669451412653	0.3753497221782666	0.1129200442933198
0.1662198856724474	0.4582944875125193	0.1204975391438497
0.2485432815947144	0.3744504072364417	0.1194381885773416
0.2914691761007110	0.4570419672826949	0.1343777821010972
0.3693943585853928	0.3706330737148432	0.1336615779208779
0.4108315226819068	0.4491580467692685	0.1615760364869220
0.4901711104796652	0.3702498861119095	0.1620844419539310
0.6219668066759407	0.3745280183136218	0.1566531371507992
0.6690083938942245	0.4596282446799406	0.1729705986255827
0.7485192549657530	0.3769172057946971	0.1360593561261510
0.7906423196043535	0.4613798634111189	0.1332826747146388
0.8736003500078200	0.3764171519303346	0.1217737527534369
0.9153924836772431	0.4598805959938859	0.1209460005416384
0.9989589785266437	0.5009457824030755	0.1178434054360742
0.0405119555652779	0.5841815146141582	0.1154278626854696
0.1244839573929363	0.5001667590523967	0.1193898344637503
0.1663398604097086	0.5834353267500511	0.1203970804141374
0.2498591921739590	0.4991936441068234	0.1294207386626504
0.2914216877677522	0.5823781910637789	0.1324496735853051
0.3744406992866898	0.4971045397116448	0.1552425585643020
0.4143838605122265	0.5808465980981659	0.1590146653338904
0.7501336610253792	0.5040097306288220	0.1410029263858129
0.7855058212713598	0.5869754067933373	0.1272805451588856
0.8736907105989078	0.5020078959304024	0.1233932200298710
0.9139874194808618	0.5851908683735907	0.1167288206586070
0.9984047765936029	0.6259438212341588	0.1119626360837218
0.0419496000188976	0.7089620864137789	0.1041945739396302
0.1244705996480045	0.6251796658305592	0.1159025630133682
0.1671667901988078	0.7083928814080841	0.1121210816013350
0.2500251118518508	0.6244118769344129	0.1251239622166666
0.2924846699047234	0.7076618932680241	0.1230240975562903
0.3748840227381167	0.6229479689234102	0.1418236342111518
0.4182385483037978	0.7070790017004096	0.1357322101299280
0.4989658750586947	0.6264869234130449	0.1772095490323080
0.5451003321350070	0.7067580584384221	0.1421249653352555
0.6784016783748839	0.7062302592848910	0.1233250303590460
0.7474616816148321	0.6366845485484538	0.1226410998610490
0.7976529022231394	0.7150408982664027	0.0997011152323271
0.8711406567297107	0.6270600686755549	0.1136707495278045
0.9173042451453596	0.7097255451008758	0.0991439459983665

0.0007323660304935	0.7507398610307142	0.0961452919296638
0.0429230478721032	0.8340513715432254	0.0880472932748417
0.1256247868664018	0.7501456545277533	0.1046627111789310
0.1676732511234633	0.8333995767805285	0.0992023319140149
0.2508447265720608	0.7494296654816139	0.1153371374362339
0.2926469245197350	0.8328383574629774	0.1116538677031225
0.3764760695897285	0.7486130177044211	0.1267356929699426
0.4178999260955688	0.8322492578394610	0.1215043920120882
0.5025651298129741	0.7482883540014177	0.1337825054811405
0.5432349529555389	0.8315682674411302	0.1241790834080592
0.6282567291412643	0.7434885750252840	0.1285254060828777
0.6681965477748489	0.8291942347461423	0.1151652459875878
0.7567065367560533	0.7563489897555999	0.1000940324304340
0.7951484960164443	0.8375465990208618	0.0915908959010776
0.8789409716180022	0.7536490555863755	0.0906899493965473
0.9195078178281009	0.8361531115991262	0.0822350348617737
0.0022468240470416	0.8768380618823348	0.0815760392702787
0.0433436736590742	0.9600223844866054	0.0796772412585162
0.1262591996415197	0.8753695247229317	0.0908426149132728
0.1679271783870162	0.9587379274126465	0.0883731837428368
0.2510355002224721	0.8746979342008284	0.1038132390777973
0.2925988331325536	0.9581100334705521	0.1024547423464152
0.3760500838256934	0.8740934018613943	0.1156040130533805
0.4173446672399959	0.9576319270547137	0.1145096765770835
0.5012339119818299	0.8736617496328931	0.1214959329704277
0.5422876134066696	0.9573929776799553	0.1189436045216402
0.6263786562993092	0.8721459182779367	0.1180382586030688
0.6671330929202445	0.9566622606595743	0.1133793322106479
0.7509630877364463	0.8756167616511717	0.1005191684837440
0.7918965712930011	0.9590373946677270	0.0973292173282593
0.8775960283137344	0.8781385932246454	0.0825602656980573
0.9181423745620380	0.9607886990295390	0.0819525508385309
0.5993434542296883	0.5538564439879958	0.3749200317664798
0.6422990516815315	0.5171656709944940	0.4129548931153801
0.6120946843700203	0.4358828322612069	0.4031686221603375
0.6558128578794629	0.4015810246389338	0.4285263403535242
0.7313774630429196	0.4478357211965903	0.4628628707643474
0.7616227547759721	0.5281569796731486	0.4742670168591669
0.7172177473475594	0.5625959350963390	0.4508399555155103
0.6181676338039938	0.6344646028265222	0.4061361012927885
0.7042145284098177	0.7700513331098642	0.3741366371606820
0.5309369871256375	0.6007440373391258	0.2322945470914634
0.6723274327964998	0.6312787070331882	0.1388329621618398
0.6481302712795630	0.4990974407626088	0.2217324481209761

0.4975585272674980 0.4947196284394285 0.4210580417091465
0.4432407299850397 0.4477173303165521 0.3855213621761875
0.4923921515641068 0.4515808634650448 0.2191148989763634
0.6023593181368727 0.5625131132815505 0.2389650495518046
0.7419459578230420 0.6255077066155369 0.4586965179941052
0.8203845444927064 0.5644431679270433 0.5011991200999922
0.7665125006661286 0.4212181834463880 0.4804459277963979
0.6314853756407591 0.3387171274166388 0.4200383164278504
0.5540502743606507 0.3997205622559689 0.3745766924392487
0.6711322032497907 0.7809531229526083 0.3227026000225558
0.7671234853497105 0.8061845956672441 0.3619009717349578
0.6884412229200226 0.7813984746261677 0.4402949437373995
0.5781193356808996 0.6499719838341199 0.4540562633173203
0.6867485429178131 0.6894952122012297 0.3670408541441310
Total energy (E): -1296.96548268 eV
Temperature (T): 333.0 K
Zero-point energy E_ZPE : 95.113 kcal/mol 4.124504 eV
Thermal correction to U(T): 104.904 kcal/mol 4.549058 eV
Thermal correction to H(T): 104.904 kcal/mol 4.549058 eV
Thermal correction to G(T): 84.774 kcal/mol 3.676130 eV
Entropy S : 252.927 J/(mol*K) 0.002621 eV/K
Entropy contribution T*S : 84224.729 J/(mol) 0.872928 eV

RhN3P SA (pyrrolic N): MS0
1.00000000000000
19.7297992706000009 0.0000000000000000 0.0000000000000000
-9.8648996353000005 17.0865073798999987 0.0000000000000000
0.0000000000000000 0.0000000000000000 15.0000000000000000
C N P Rh H O
131 5 1 1 8 2
Direct
0.0017517776720228 0.0020900002925652 0.0810922424642908
0.0425838009147072 0.0849479454100035 0.0855159488182352
0.1265272592398990 0.0009478311551368 0.0849301605855028
0.1675093833625212 0.0839723180592571 0.0881421579089588
0.2510571786515903 0.9997880693184680 0.0965728443946581
0.2923841416368601 0.0831038180034355 0.0998666427641101
0.3756875594758284 0.9991679378038463 0.1096695256899416
0.4169037168037005 0.0825736437369107 0.1137304778882875
0.5006552327179704 0.9991410748229290 0.1164568280832608
0.5417041094731734 0.0828362339342965 0.1191005467290148
0.6256274380423967 0.9986810366910206 0.1145850823776621
0.6666741032619332 0.0827158203322041 0.1155351386253275

0.7504148593257504	0.0002226511873921	0.1040778509453396
0.7916136850729659	0.0838958718809170	0.1051830765932574
0.8756592014801996	0.0014752181761363	0.0887580079061774
0.9169017010403281	0.0847076574932300	0.0924644447805724
0.0002478596674166	0.1264149385375423	0.0906704717333294
0.0414861518174811	0.2094226700293850	0.0976032295105753
0.1255992811457426	0.1258929467058728	0.0883736570442633
0.1664642466329759	0.2088033165656762	0.0960842392938684
0.2504258430942689	0.1248291976500741	0.0956180837731185
0.2910263420645841	0.2074374295459223	0.1030288958488238
0.3751865328474039	0.1243480776650998	0.1105440879836136
0.4159708018760426	0.2072639454366313	0.1200312392671223
0.4998466231812248	0.1246814969691832	0.1204703765350879
0.5408132863643623	0.2084676900092788	0.1283411823907221
0.6249610408390941	0.1246424049215901	0.1198635856470154
0.6659633470492329	0.2084077495946674	0.1237154755552667
0.7498655996719347	0.1254354795457595	0.1120998536621576
0.7909213784761114	0.2091455645190432	0.1146498801523792
0.8748451938980853	0.1259756901445138	0.1003879277255263
0.9161617354581663	0.2093634130397075	0.1046766380217441
0.9995458045409354	0.2509504214113553	0.1037751835337541
0.0409430727776914	0.3340410032384576	0.1102559006912710
0.1247938286740422	0.2507749245988803	0.1007604786909848
0.1660528121700511	0.3335771070570007	0.1116965784565337
0.2490387346733990	0.2494261947576391	0.1034597504776765
0.2890927068064888	0.3314658064644145	0.1174242128859136
0.3731296083961290	0.2475713901065660	0.1160730962792514
0.4123923683249164	0.3287639052392806	0.1325552827870485
0.4986822961901376	0.2501783683733227	0.1332803129788744
0.5390452767339886	0.3335958704022925	0.1514073157810602
0.6239957772348079	0.2499648539402975	0.1302099133624174
0.6646405133046825	0.3336359341641983	0.1367016036645404
0.7491544399302549	0.2508255448006981	0.1210854964542419
0.7901502410953526	0.3349364435060710	0.1229159323011528
0.8743114585111865	0.2509596152277001	0.1112287919582370
0.9155735264800433	0.3344374890925195	0.1139692499854010
0.9991070033042925	0.3757481184699150	0.1133638470998299
0.0410406146744911	0.4590272911617229	0.1157760882638933
0.1243537806833667	0.3752961933077635	0.1145662169894176
0.1665411936564918	0.4582362281665878	0.1211950025202404
0.2489610545485786	0.3743709526658360	0.1216418808379170
0.2920158502559914	0.4570704020047165	0.1354440964129953
0.3702944517393863	0.3707747148613422	0.1337338781031261
0.4119755121475566	0.4492442306703180	0.1631166280480605

0.4913586449940711	0.3707698773669475	0.1598241347146548
0.6223731394153769	0.3734125672544197	0.1511916922963265
0.6687037184774833	0.4589071365689086	0.1655217142133151
0.7486860457548097	0.3767452343746233	0.1298520701940410
0.7903586913936261	0.4611750605999343	0.1260393630314033
0.8738192990230551	0.3763375440785578	0.1179598494532871
0.9154129523924753	0.4597052360600940	0.1166223620678092
0.9990710201801831	0.5007989862873166	0.1149413660085345
0.0405549035364825	0.5839943694317028	0.1123926997680580
0.1247122610906355	0.5000913630018415	0.1188939822519409
0.1664373873008837	0.5832947616412724	0.1192478991519654
0.2502383605822538	0.4991388560056054	0.1297998452535904
0.2915818830371094	0.5823070081278325	0.1315160063589464
0.3751857453787440	0.4972402794589701	0.1557048718974364
0.4147945353892126	0.5809640227512658	0.1569157022488708
0.7495695677992295	0.5038940751563287	0.1323403902201920
0.7852954215310294	0.5865990859234521	0.1193463495086482
0.8735434095895916	0.5017831726040956	0.1173846089923811
0.9139035843781925	0.5849284574568060	0.1110520439025637
0.9984122372007299	0.6257306610808252	0.1079559139095619
0.0419611612478957	0.7087432669477258	0.1013203976494970
0.1245230902601368	0.6249955535644421	0.1139762230743683
0.1671947215838203	0.7081753946785445	0.1104795485768529
0.2501365209151571	0.6242734001074569	0.1239204314201501
0.2925575502922748	0.7074449179089533	0.1213406567872635
0.3750594923494445	0.6229073397403232	0.1395875937026436
0.4183523250771962	0.7068111088533833	0.1323319975699542
0.4996926888448707	0.6269908947514542	0.1728846466219524
0.5453468202536361	0.7068080177725498	0.1370918496488180
0.6785192423740388	0.7063834190639707	0.1171144950321596
0.7473612845111153	0.6364549139339911	0.1152820357833633
0.7977844190882882	0.7151073990223578	0.0940123829454589
0.8710944143339758	0.6267989432453140	0.1073309587806056
0.9173671653372263	0.7096162575802609	0.0944965813443854
0.0008009805231353	0.7506165370282872	0.0932255904242609
0.0430628047149975	0.8340044972834566	0.0869389645557803
0.1256421455659301	0.7499312227112458	0.1030530563212495
0.1676925584523140	0.8331818529110765	0.0986519140158502
0.2508859746878234	0.7492127397015351	0.1139326362339678
0.2926647616830570	0.8325549553261450	0.1106009057207505
0.3765700887127416	0.7483650959791939	0.1241661969292944
0.4179678749502534	0.8319590318665162	0.1190751275138809
0.5027502850980851	0.7481036110327285	0.1292372233926952
0.5434040852252552	0.8314030253131607	0.1201994321559451

0.6285307800157680	0.7434533582930731	0.1231783327616633
0.6684617628630590	0.8292210688130996	0.1109022198925532
0.7568750909408235	0.7565017372756164	0.0949221772717429
0.7954992418920540	0.8378051900235431	0.0880282736070154
0.8790981373245139	0.7537123283854192	0.0862646570160315
0.9198149789784752	0.8363552155323704	0.0797992478974668
0.0024932104871712	0.8769256021650133	0.0808563520719124
0.0436416343246655	0.9601126253099633	0.0807100043486637
0.1263306084016932	0.8752299023616253	0.0908485725871795
0.1680240283129386	0.9585906416130825	0.0896070783472081
0.2510319829533093	0.8744387766741916	0.1035257977287027
0.2925870643938137	0.9577967974291891	0.1028071978282819
0.3760997007504585	0.8737794500020770	0.1141308527318318
0.4174070373739648	0.9573083659249711	0.1134246765300805
0.5013493292412162	0.8733999917553956	0.1185505589718392
0.5424290633242307	0.9571050883869652	0.1166048345390907
0.6265765979390997	0.8720231151386992	0.1143782497788198
0.6673645055717915	0.9565310689217076	0.1109054964971958
0.7513268615755941	0.8757505553568312	0.0973567360466456
0.7923153124999693	0.9591953905546565	0.0958315922533836
0.8780217749398990	0.8784179704302270	0.0805065885689698
0.9185979698428467	0.9610096374573441	0.0818642946298228
0.6269021999926860	0.5680220946171533	0.3607182974629186
0.6543767633216928	0.5224406772669735	0.4072251881717039
0.6191882564551601	0.4404824445504706	0.3957103180914611
0.6523406070587715	0.3996312021174301	0.4338421112481388
0.7215980499055379	0.4394430794341599	0.4832871909801847
0.7565137478616923	0.5202905839675314	0.4968805434629711
0.7227848482159023	0.5612510916343859	0.4605012157823211
0.6399927788306254	0.6421996003749456	0.4013548151012732
0.6963168205920331	0.7807419267523251	0.3785300387410303
0.5312269672199015	0.5999712593138967	0.2265743983246938
0.6721682223948274	0.6305594613124342	0.1314763390514505
0.6426608135335256	0.4931243999473906	0.2147132437130989
0.4472236437553623	0.4518031159560138	0.4808438855269157
0.4112307037310078	0.4610684277955385	0.4304446724846280
0.4929323446212389	0.4501995192695155	0.2210343356463077
0.6051901786786250	0.5648932073078042	0.2344031014655730
0.7504778188123665	0.6244017690372471	0.4701778796078450
0.8104091167715485	0.5512883128479498	0.5354854614463591
0.7484281450042388	0.4074547732596522	0.5114252306169716
0.6246963448779419	0.3365039061776540	0.4245096897220022
0.5654105068452780	0.4097829585746433	0.3570540150019232
0.6830542131917373	0.8056101959066617	0.3202094486289446

0.7574504726153224 0.8186741783401063 0.3987215839345089
0.6568754990485132 0.7726974698318818 0.4340388462930597
0.6090558818677088 0.6433679728778404 0.4716778169621872
0.6869620392113487 0.7061509343018427 0.3508462647177212

Total energy (E): -1297.57227727 eV

Temperature (T): 333.0 K

Zero-point energy E_ZPE : 94.295 kcal/mol 4.089018 eV
Thermal correction to U(T): 104.384 kcal/mol 4.526519 eV
Thermal correction to H(T): 104.384 kcal/mol 4.526519 eV
Thermal correction to G(T): 82.213 kcal/mol 3.565107 eV
Entropy S : 278.565 J/(mol*K) 0.002887 eV/K
Entropy contribution T*S : 92762.177 J/(mol) 0.961412 eV

RhN3P SA (pyrrolic N): IS1

1.00000000000000
19.7297992706000009 0.0000000000000000 0.0000000000000000
-9.8648996353000005 17.0865073798999987 0.0000000000000000
0.0000000000000000 0.0000000000000000 15.0000000000000000
C N P Rh H O
139 4 1 1 19 3
Direct
0.0021432472745204 0.0016724065863565 0.0815795639716412
0.0430302596137522 0.0845375255506464 0.0843989084151857
0.1269139135407737 0.0005954448965569 0.0846138999475440
0.1679298782542038 0.0836200236164170 0.0862208984458267
0.2514971471165299 0.9995238400652843 0.0962871482602087
0.2928587390709335 0.0828480995316041 0.0984739069791884
0.3761407119252224 0.9989429822502826 0.1100133891877993
0.4173840414033862 0.0823669334181200 0.1134593891188932
0.5010952291208305 0.9989111679875056 0.1175334301054674
0.5421530200017060 0.0826278366497545 0.1198623817276533
0.6260714031229729 0.9984048468118377 0.1163694575298132
0.6671167667696096 0.0824479495595583 0.1170002724618898
0.7508513666678638 -0.0001477360972173 0.1061344922119019
0.7920683461832715 0.0835267285367048 0.1067169916642853
0.8760837474161840 0.0010403283724787 0.0904276296181760
0.9173836889329323 0.0842922144551107 0.0931174137760528
0.0007058926003084 0.1259908386429095 0.0894489691373381
0.0419923042822740 0.2091228231972211 0.0939153817729337
0.1260756694078008 0.1255603123447837 0.0854310007596953
0.1670507815534775 0.2086042572424682 0.0902067084198829
0.2509221519187853 0.1245402803708484 0.0928396352032187
0.2916580201105051 0.2072633617795510 0.0980933971293601
0.3756810445847605 0.1241195045567674 0.1091549859174653

0.4164928737727877	0.2071152019048110	0.1176200239268843
0.5003313513904809	0.1245284320186900	0.1204453108076997
0.5412748636402067	0.2083469587014923	0.1279319051261179
0.6253995314294223	0.1244234329729661	0.1208417535739705
0.6664321560474371	0.2082201927846671	0.1242239174130317
0.7503118863558886	0.1251005679113951	0.1133046040263715
0.7914030934706887	0.2088037647166128	0.1150243219776501
0.8753092933191932	0.1255757749787923	0.1010838649026971
0.9166275484335282	0.2089991221074598	0.1039058929225556
0.0000201092677839	0.2506202903094931	0.1007980044558628
0.0414395980442683	0.3337902678361194	0.1056308325698970
0.1253855929280193	0.2506031086950172	0.0940133940470543
0.1667257495908960	0.3335595763910532	0.1031748288491376
0.2497166290580850	0.2493034609711768	0.0965193301849152
0.2898525553951115	0.3314828582261324	0.1091416368672711
0.3737636724543758	0.2474466217801409	0.1115929684532680
0.4129773549273488	0.3287087289926289	0.1273993034853928
0.4991162634970454	0.2501061676474353	0.1318054592148792
0.5392908300223229	0.3335447832317069	0.1497474690462386
0.6244516209158758	0.2498788016766229	0.1302532874946564
0.6650985204692113	0.3336161940331288	0.1364453062958031
0.7496345755743389	0.2505275744916222	0.1212574444974458
0.7906384569008323	0.3346161934328897	0.1223371153072492
0.8747750236185953	0.2506005632290441	0.1105690742516382
0.9160582396143507	0.3340922615372767	0.1121006687907745
0.9995780493960295	0.3754257596496739	0.1098256524124049
0.0415144265569425	0.4587055263280702	0.1117226254535744
0.1249092410177333	0.3751149681837110	0.1076087871410307
0.1670547932125456	0.4579987198322949	0.1147865630321300
0.2496699888113271	0.3743745431623900	0.1128350909005822
0.2924576784391019	0.4568021049984275	0.1282768977053305
0.3708387891886942	0.3706761354628334	0.1270223428332670
0.4119379825115178	0.4486989203659526	0.1578033074789463
0.4913962002909548	0.3705536757946459	0.1567822076630850
0.6226833510350289	0.3735813069379086	0.1502987415047061
0.6696217370284968	0.4589846706898268	0.1651863690296576
0.7491457823052071	0.3764323748683940	0.1293260497873419
0.7910240575775295	0.4608261812414552	0.1249741476104428
0.8742900711221991	0.3759767296288684	0.1163858327321732
0.9159536465902172	0.4593521950076098	0.1142945228680835
0.9995639443555209	0.5004567458462407	0.1117772089030465
0.0410307025154271	0.5836611600451297	0.1096541888766940
0.1251766993597072	0.4997787054866175	0.1139520646048914
0.1668741483081597	0.5829808917734211	0.1153269147149200

0.2506826958663460	0.4988655662789879	0.1235218367866587
0.2919176266984538	0.5819462463654037	0.1270929116052330
0.3752120073248611	0.4966724353215765	0.1502955186286453
0.4147095560211096	0.5802851724594362	0.1534991972048179
0.7503879212212299	0.5034733180225567	0.1318449546382727
0.7858024967693489	0.5862003172894027	0.1187977775091705
0.8741168385535236	0.5014057019964560	0.1155374654252066
0.9143970882374285	0.5845475300363135	0.1092973432200277
0.9988861371939138	0.6253790041754209	0.1060014172893919
0.0424194816287657	0.7084352971839853	0.1001433801975638
0.1249738775641630	0.6247038132689593	0.1111564195436823
0.1676278184782828	0.7079150922085333	0.1087961288413100
0.2505228743386033	0.6239553346545998	0.1203720001527158
0.2929739189813993	0.7071613663473229	0.1193380669742083
0.3752821906070724	0.6224548388764852	0.1363757926745796
0.4186996881520304	0.7064509319815226	0.1308906855369929
0.4993008670142033	0.6257005435341787	0.1712248036264752
0.5455687249122070	0.7060790699888818	0.1369305525459470
0.6788986748645434	0.7058513936938855	0.1181775499135285
0.7477401347787275	0.6359542620763046	0.1153301873165578
0.7981693374274416	0.7147017510486150	0.0945118659428915
0.8715435165627340	0.6264047509884340	0.1063107879284516
0.9178134100783136	0.7092729517367405	0.0940592323149916
0.0012511800077019	0.7503224340556367	0.0928211377800630
0.0435055403438596	0.8337284745745406	0.0871545768130619
0.1260858724143534	0.7496780002121165	0.1020631610197638
0.1681257921052843	0.8329517967274684	0.0984734556607345
0.2512850093845543	0.7489452051055341	0.1127353021355920
0.2930872061335936	0.8323367371488601	0.1105481487747123
0.3769763542313315	0.7480818405751593	0.1230917507053138
0.4184116856238946	0.8317314064791731	0.1192601569156084
0.5031176179319352	0.7476395743491095	0.1291442487639957
0.5438042925137535	0.8310561563810942	0.1211862341101990
0.6288440279916325	0.7429355350839930	0.1241924484605627
0.6688495276296370	0.8288010986304399	0.1126994223239041
0.7572763506253439	0.7560958821704344	0.0961414725037376
0.7959114048753057	0.8374243132985172	0.0898176294842485
0.8795026279146244	0.7533543691161505	0.0866452373702948
0.9202167036376728	0.8360086555642189	0.0807828511246641
0.0028821328964874	0.8765999924240677	0.0815645271486957
0.0440146982655079	0.9597425162702691	0.0811655079442328
0.1267729260393098	0.8749857198632823	0.0909766081021363
0.1684459363682557	0.9583105541790732	0.0895385925260263
0.2514536886213301	0.8742172190078744	0.1036390976962556

0.2930352368444081	0.9575791599515848	0.1030157971621046
0.3765274934264729	0.8735737590373307	0.1146121083685805
0.4178465215813929	0.9570944785468837	0.1142212416233221
0.5017943147085958	0.8731346823955717	0.1194994858735694
0.5428702873506002	0.9568581419451150	0.1180313490916736
0.6269900096416280	0.8716673790624951	0.1160817811723063
0.6677993872158362	0.9562083268961784	0.1128704054953358
0.7517443433307069	0.8753570432145582	0.0995215251999163
0.7927563478456132	0.9587948366225232	0.0980409053739474
0.8784104039398388	0.8780341390710104	0.0820894529746543
0.9190012210005628	0.9605837456100030	0.0833028245552481
0.6169680398324840	0.5617227984250247	0.3614203145398276
0.6420442200100722	0.5163765087355970	0.4119056446959966
0.6125700573255681	0.4357889881267066	0.3948846501214537
0.6424296566308683	0.3950074671585878	0.4399774559168015
0.7025458943103746	0.4334126661721189	0.5025639356076477
0.7312998610092816	0.5126234960949135	0.5218305249227410
0.7009061280310118	0.5535728953704432	0.4778525744845886
0.6191570012318486	0.6313512076981914	0.4017046224527446
0.6619865201653836	0.7666529567718621	0.3865102368040690
0.3520208698665363	0.3435595967445771	0.4438474727896184
0.2651217138816225	0.3070176903459451	0.4291362941783493
0.2355326635475043	0.3160555633624627	0.3477339404447089
0.1555701013196535	0.2842321166521423	0.3336042332909929
0.1013905869757583	0.2412784317950930	0.4009731933795824
0.1304186037189783	0.2324209302783591	0.4831198990898545
0.2107740896550684	0.2649515163635638	0.4963909559486951
0.3912467394769859	0.4311343335361755	0.4639314979641715
0.5294853942536913	0.5973732078064601	0.2250716222795903
0.6724138236243861	0.6299537430693267	0.1317563714458767
0.6465551121194159	0.4958158963326788	0.2145566120403712
0.0213260058962121	0.2114365265901785	0.3872696856404350
0.4910080789082146	0.4486072785892350	0.2187494650263144
0.6036861663480615	0.5626105457043263	0.2334985990926897
0.7238914208246588	0.6155193225735049	0.4921502043922156
0.7777220161136051	0.5423660819888946	0.5709832085068206
0.7267831659159302	0.4014710871359378	0.5367801898583323
0.6188576883612509	0.3328690438221653	0.4265956363036649
0.5646245933051673	0.4058107462287922	0.3478319610883450
0.6023434565578036	0.7545440665889518	0.3723413821557505
0.7046313039718073	0.8148706970321298	0.3451583753881918
0.6747426637442671	0.7797438294335350	0.4576747757362033
0.3804906481969183	0.3359753575822104	0.3844144602910519
0.2317870864504498	0.2570584911299315	0.5608092274608254

0.0896811524079529 0.1998864231716770 0.5369560453722332
0.2761976295194397 0.3485865093782106 0.2939082812268506
0.1343359311193282 0.2923849273185508 0.2696563289634330
0.3639731115516825 0.3149032942570793 0.4997655191449113
0.0037885215946358 0.2023245408177499 0.3225922324345143
0.9852280235098758 0.1659440256237358 0.4270113067842099
0.3627069435772516 0.4405531593957290 0.5224153253571445
0.3832946479727758 0.4618491168451017 0.4066393075435697
0.4999993143347541 0.5166149888487922 0.4847297162368520
0.5753912225057316 0.6259075324875051 0.4632915497358970
0.6694538408827544 0.6996759391529032 0.3613646687593568
0.4722947072349510 0.4593231708035375 0.4805624806418795
Total energy (E): -1409.47316239 eV
Temperature (T): 333.0 K
Zero-point energy E_ZPE : 200.842 kcal/mol 8.709337 eV
Thermal correction to U(T): 217.287 kcal/mol 9.422443 eV
Thermal correction to H(T): 217.287 kcal/mol 9.422443 eV
Thermal correction to G(T): 183.882 kcal/mol 7.973863 eV
Entropy S : 419.720 J/(mol*K) 0.004350 eV/K
Entropy contribution T*S : 139766.745 J/(mol) 1.448581 eV

RhN3P SA (pyrrolic N): TS1 (imaginary frequency: 335.383863 cm^{-1})
1.00000000000000
19.7297992706000009 0.0000000000000000 0.0000000000000000
-9.8648996353000005 17.0865073798999987 0.0000000000000000
0.0000000000000000 0.0000000000000000 15.0000000000000000
C N P Rh H O
139 4 1 1 19 3
Direct
0.0026164552679499 0.0023360185594307 0.0771019740784629
0.0434212724098564 0.0852613843053258 0.0793517225725676
0.1274847888901868 0.0011313325219092 0.0791364792184395
0.1684036260109998 0.0841960156790203 0.0800060244459643
0.2522007066273335 0.0001895128634641 0.0909620173820652
0.2935079310187550 0.0834973010385227 0.0922930945158223
0.3768873154100423 0.9997355253623311 0.1062265357537368
0.4181255454512617 0.0832179333541645 0.1091731768005013
0.5018465469966871 0.9998902679077899 0.1159450314542748
0.5428571414432005 0.0835916192378792 0.1180790903650369
0.6268289058066199 0.9995038509696031 0.1159430280205415
0.6678050630135342 0.0834995408685111 0.1162749402881368
0.7515764751601370 0.0008815348708992 0.1049120613221233
0.7927673786957969 0.0845830809233506 0.1050380715833990

0.8766550793234738	0.0019117970749790	0.0874759946468012
0.9178870328737067	0.0851961721857791	0.0896320557510952
0.0012054321244789	0.1268225279362090	0.0852168891811886
0.0425579293758844	0.2100306595865389	0.0903346829003961
0.1264984036509523	0.1261733615358724	0.0797677530567928
0.1674279561443053	0.2092280747809895	0.0849908679852350
0.2514040158690098	0.1250384147156706	0.0862602927409641
0.2919411619346045	0.2076875826758615	0.0914421008675990
0.3763820074091249	0.1248514871503202	0.1035775790273754
0.4171137424207457	0.2078053562164409	0.1120505637128368
0.5010146625266144	0.1254360837871022	0.1176577602868332
0.5418769149478337	0.2091768188521328	0.1259437657631214
0.6261027193618243	0.1254826310495139	0.1200545706104533
0.6671265456659204	0.2092810600554478	0.1246828507804271
0.7510484284782821	0.1261715201982969	0.1125308005223637
0.7920921636207112	0.2098498011274872	0.1153327425659938
0.8759396575022977	0.1265760692031579	0.0985871022441297
0.9172455665913173	0.2099933236341653	0.1022436834944129
0.0006546379710670	0.2516039540610596	0.0985955027593502
0.0421082186845285	0.3347724147434073	0.1046111383153092
0.1259172211847592	0.2514081479912550	0.0900151030253053
0.1673721365255825	0.3344252365378735	0.0997180679173625
0.2500794337088255	0.2497245265966332	0.0904727995586707
0.2902874330906082	0.3319507899268402	0.1031767916921542
0.3740426115422908	0.2477654114397355	0.1052523929935043
0.4128961965710559	0.3287213170306597	0.1206596188604236
0.4996553718523342	0.2507971577578813	0.1280860701324555
0.5395324090357447	0.3340022014156041	0.1475541633054116
0.6250913265818367	0.2508946699085080	0.1308332587540939
0.6656431345526881	0.3344317361611915	0.1402667084055517
0.7503418630053633	0.2515654815283959	0.1229509709250396
0.7913190127005364	0.3355737652641014	0.1264503911972708
0.8754686471766201	0.2516193486099271	0.1107067129082387
0.9167630453806361	0.3351064603033888	0.1138539358032662
0.0002584431911269	0.3763931503389677	0.1108538955868570
0.0422068520978399	0.4596609971214769	0.1139301741060896
0.1256528664884753	0.3760758953231816	0.1059092218816923
0.1678436771578591	0.4589182568627596	0.1144880707183307
0.2503745315332968	0.3751494870908560	0.1083335108921298
0.2932522010361769	0.4575615354113995	0.1248782639264877
0.3711141975039741	0.3706861657678971	0.1198148885919105
0.4127432225032265	0.4491690281637853	0.1496023995635776
0.4913356382774774	0.3704392832547305	0.1505998955236630
0.6228546126980153	0.3740955821279514	0.1542618400889814

0.6691128034354196	0.4589770041685304	0.1723377051039008
0.7498146817820689	0.3773775561523059	0.1353911234530404
0.7915338874983606	0.4618180473401949	0.1332415444050576
0.8749813351710142	0.3769670576698132	0.1204355376652119
0.9165499339779071	0.4603493709324187	0.1196985278033763
0.0001954034751271	0.5013916022128480	0.1158473413110605
0.0417573787883255	0.5845698759627390	0.1135885655746659
0.1259176201736714	0.5006723216354159	0.1154234812338365
0.1677695384437542	0.5838797819894752	0.1174697770447573
0.2515702304810078	0.4997319039668119	0.1228508766582567
0.2931713752495664	0.5828548296565613	0.1284923604231769
0.3762630968400745	0.4972918214252979	0.1455606833918895
0.4161701687003303	0.5804883922844805	0.1528782353530399
0.7505709630416416	0.5045506074925908	0.1406970525818015
0.7867085039231598	0.5876571046803485	0.1269777205690416
0.8746361023339196	0.5024233593693378	0.1228165308605227
0.9150938233515984	0.5855931792168052	0.1157958593048337
0.9995410514882019	0.6262873071568047	0.1105338597367545
0.0430877964005918	0.7092688495996063	0.1025233796062104
0.1257488187892617	0.6255369184211865	0.1137530080920203
0.1684052852012572	0.7087597110971136	0.1101987987152173
0.2515430358280189	0.6248662110242275	0.1223833049295245
0.2937892576577844	0.7080466751131140	0.1212453987042123
0.3766103197303622	0.6233310543753054	0.1387012909600779
0.4195292024952207	0.7074753641258404	0.1347005118928907
0.5008225402707809	0.6264813376952834	0.1718760998737764
0.5466497539708417	0.7081676242425465	0.1410753761909465
0.6801705970863845	0.7071910382003269	0.1231921388916716
0.7494645586040914	0.6378892769501358	0.1225700267924023
0.7994379312257283	0.7161427197810948	0.0988340337052618
0.8722793870125803	0.6275031906714958	0.1128394721564272
0.9185220299725457	0.7101674100663604	0.0978109536206364
0.0018925119264801	0.7510538594871593	0.0944271878973822
0.0441154106394358	0.8343680449556942	0.0857954897870692
0.1267988146215455	0.7504589754507403	0.1027279961050945
0.1688049973500699	0.8336294588859834	0.0968023159867027
0.2520704787989336	0.7497838516057719	0.1135305776349322
0.2938278925529322	0.8331528871883975	0.1096718728641528
0.3777638487125645	0.7489971487764495	0.1256152929966411
0.4191627333152612	0.8326680482716762	0.1205004736878792
0.5039799444485858	0.7491944314623340	0.1335676027606069
0.5446401128343966	0.8323552135084036	0.1239683917433609
0.6298714856280648	0.7443447013819935	0.1278933159013858
0.6697785205031438	0.8300334508861121	0.1144833569784097

0.7583814528873996	0.7572410125269806	0.0993464116941539
0.7966096008004555	0.8382915671208853	0.0902766870356132
0.8804447995003079	0.7543488641673236	0.0892379242436130
0.9209098600661147	0.8367956799964088	0.0803332843637083
0.0035431008626957	0.8772608162635067	0.0792455643557599
0.0445541468405576	0.9603671254286209	0.0767583046851149
0.1274079831020513	0.8755775179101768	0.0881329162745901
0.1690561587283547	0.9589051324061546	0.0846835064920702
0.2522050191240529	0.8749227923535176	0.1012166872437137
0.2937864665741308	0.9583189663911701	0.0989142454388222
0.3773004851414669	0.8744342572805009	0.1138673211512781
0.4185733083368581	0.9579321723756660	0.1120502981782463
0.5025605941347476	0.8742582495995719	0.1207718726302370
0.5436176033737586	0.9579154695967809	0.1177183914406978
0.6278573793766936	0.8728439194029924	0.1176232533736978
0.6685714180798786	0.9573099452477465	0.1128308492697437
0.7524704026337555	0.8763402642702752	0.0996055000049891
0.7933691081380363	0.9597217429244151	0.0965285158477284
0.8790215520084526	0.8787309439678403	0.0808710714733307
0.9195149366685337	0.9613546948278074	0.0801688094724948
0.5750838255970044	0.5332901095627937	0.4015250026819496
0.6295642021238598	0.5091814449423993	0.4327997168863659
0.6148285570528810	0.4329604977905203	0.4126280997255855
0.6638167090652980	0.4065096026131484	0.4439038997961289
0.7279339569804937	0.4547283714287450	0.4980892656220123
0.7424848931226788	0.5296909838099835	0.5202171159610882
0.6944746617489853	0.5570296991411090	0.4874888273075319
0.5873158574309988	0.6137488172657208	0.4103431237492826
0.6804338828602815	0.7486986293853407	0.3819247916320446
0.3774569431667454	0.3653993491406569	0.4468142340067252
0.2911174698199760	0.3280658114339687	0.4286736337487810
0.2628218774286784	0.3431811967441220	0.3496684893192495
0.1829923406128582	0.3092245177601993	0.3326958975528001
0.1275227773796422	0.2579594423948815	0.3946123660320600
0.1551256925938543	0.2431705911876048	0.4746445447269051
0.2352298602538169	0.2777620568804383	0.4907747229883031
0.4132797716491155	0.4455551115136347	0.4958534307835833
0.5378867877587725	0.6033961011415879	0.2222261893689224
0.6749230170727776	0.6325077883517689	0.1386193282684755
0.6465142315601135	0.4962892345820752	0.2229786087551172
0.0476059165125340	0.2253896308020394	0.3782231868855718
0.4947354655605479	0.4526051736707110	0.2023237956652622
0.6025679673597502	0.5602463081468164	0.2345429961502499
0.7061680494338934	0.6152754316363758	0.5058846090365420

0.7913338231985185 0.5669469012338745 0.5639344071261516
0.7659109131450723 0.4337013911199030 0.5235683848581730
0.6519740113219915 0.3478761387863226 0.4265573306706138
0.5643419793183628 0.3948816366385089 0.3714416102520638
0.6290351784264836 0.7511258681486603 0.3583809956231732
0.7291936776566231 0.7778483113104988 0.3348839466861409
0.6976445081393507 0.7764050876959555 0.4478865531324868
0.4091334931279530 0.3748381357360164 0.3830847685545062
0.2549407379446837 0.2646100082221689 0.5531326227248626
0.1133164376734798 0.2039578264386047 0.5242448178589244
0.3044213689838975 0.3818997415916297 0.2996400748879905
0.1628524405778258 0.3219072095197265 0.2705355215648900
0.3902908627648841 0.3266895353362368 0.4874040238821556
0.0318592362634129 0.2222449801004026 0.3132746976609326
0.0115636963266484 0.1761649582438100 0.4128561990738608
0.3791444618897873 0.4367581663814394 0.5583493439782826
0.4036910220411040 0.4878511755334916 0.4563432986071315
0.5164351232601592 0.4883090548924065 0.3840972083201569
0.5379465451415413 0.6311629942481964 0.4291411420988940
0.6634156562516398 0.6685826620790190 0.3872176688486955
0.4902338224546527 0.4760947533172622 0.5205782960431841
Total energy (E): -1408.25873994 eV
Temperature (T): 333.0 K
Zero-point energy E_ZPE : 198.431 kcal/mol 8.604792 eV
Thermal correction to U(T): 214.603 kcal/mol 9.306050 eV
Thermal correction to H(T): 214.603 kcal/mol 9.306050 eV
Thermal correction to G(T): 181.826 kcal/mol 7.884731 eV
Entropy S : 411.821 J/(mol*K) 0.004268 eV/K
Entropy contribution T*S : 137136.439 J/(mol) 1.421319 eV

RhN3P SA (pyrrolic N): MS1
1.00000000000000
19.7297992706000009 0.0000000000000000 0.0000000000000000
-9.8648996353000005 17.0865073798999987 0.0000000000000000
0.0000000000000000 0.0000000000000000 15.0000000000000000
C N P Rh H O
139 4 1 1 19 3
Direct
0.0022079904776685 0.0023086604963803 0.0735780267273538
0.0429379640202645 0.0852143633175199 0.0755481076719315
0.1271005717249265 0.0010590898739124 0.0750296509208927
0.1679444385216134 0.0841145577265308 0.0749957065038447
0.2518903254996356 0.0002120361231037 0.0864934149489344

0.2932172716123189	0.0835026790872380	0.0866262056065385
0.3766223248157360	0.9998110674908325	0.1019932396196600
0.4178519113010021	0.0832597546581430	0.1039797572883879
0.5015225208486032	0.9999109152936396	0.1127116775799595
0.5425766326901930	0.0836496658494854	0.1144224221793673
0.6265154164967730	0.9995295072942649	0.1138568843674364
0.6674931708716602	0.0835227464061102	0.1140410760709376
0.7513044370875336	0.0009431875589908	0.1032595460033908
0.7924527064838872	0.0845761789273804	0.1032785036072620
0.8763070766824552	0.0019444837778152	0.0848649368869172
0.9175158649794658	0.0851955764698081	0.0870606465509603
0.0007744975840673	0.1267606310286126	0.0820117434730897
0.0420932803185076	0.2099831510472152	0.0871416604858551
0.1260056615602905	0.1260809882185963	0.0750649838208701
0.1668814611013875	0.2091090256930281	0.0798551272249614
0.2509578786993191	0.1249705324135269	0.0803068043779634
0.2914003604940109	0.2075836629101845	0.0846439847763885
0.3761332644468603	0.1248195979371981	0.0974445365429072
0.4169025877982134	0.2078926562521505	0.1051540804700367
0.5007690984276497	0.1255264871439292	0.1128726784340004
0.5415745383721466	0.2091894111193252	0.1207922448751334
0.6257873843450814	0.1255496162113326	0.1170675869656769
0.6668047645391737	0.2093394954447392	0.1215811929382317
0.7507341456102272	0.1261887212704974	0.1106366418479891
0.7918208265203770	0.2098972731921979	0.1134299178956344
0.8756232648192422	0.1265987956164868	0.0965269295977186
0.9168568583226158	0.2099716492744403	0.1002405609234110
0.0002518526280762	0.2515795682085171	0.0961418766467056
0.0416910404891415	0.3347568730521969	0.1022952158263969
0.1254107860237671	0.2513749345822378	0.0858690435934995
0.1667716520899552	0.3343957445879165	0.0953580155608737
0.2494961915833372	0.2495630783920874	0.0841038473280828
0.2895929549744539	0.3318343912492996	0.0965552763008785
0.3735844814679981	0.2476116768179251	0.0979543626375630
0.4122687310649227	0.3283627638163146	0.1138605500832473
0.4993574359992234	0.2508632387752167	0.1217614114006900
0.5390002558313570	0.3339965743243545	0.1413017097637867
0.6247613515421759	0.2509741866013740	0.1267624254893526
0.6653372575006022	0.3345211059870898	0.1363299372869672
0.7500748515752571	0.2516339134240279	0.1206518897566004
0.7910365182067016	0.3355753213736134	0.1244764099243316
0.8751457067277912	0.2516230369059916	0.1090041309018631
0.9164288744611337	0.3351211836821344	0.1124463153770664
-0.0001141603774997	0.3764196498825430	0.1092806085781995

0.0418083023670634	0.4596758756557791	0.1125981931752679
0.1251591673159098	0.3760206444669616	0.1027291416249484
0.1674304636167594	0.4589595319663463	0.1113610079603289
0.2499302380892972	0.3752197773391551	0.1026441587738772
0.2928335718737791	0.4576299011227674	0.1194862527458503
0.3702822114780771	0.3705209094938304	0.1132018885600152
0.4117619555836201	0.4489688206007494	0.1421119187965199
0.4906526923268525	0.3698872903397930	0.1430139374514094
0.6225774903275072	0.3743615974326879	0.1496770221254137
0.6684114460202693	0.4585404030813771	0.1688817698507562
0.7494419443005566	0.3774045288624446	0.1328563153234014
0.7911541157501177	0.4618523045359655	0.1318850133578120
0.8746746741061305	0.3769840706804444	0.1192169333794706
0.9162535713266967	0.4604583409505422	0.1190669739803458
0.9998389668635013	0.5014590968144012	0.1152021589973665
0.0414505404432284	0.5847022817559528	0.1131239158938697
0.1255341571937249	0.5007228155939052	0.1133757285024508
0.1674080157427524	0.5839425018289238	0.1155672426475257
0.2510734051542125	0.4997525384871512	0.1187113089063371
0.2927533780755417	0.5829004768507712	0.1245972400803002
0.3758071693442594	0.4971984921191838	0.1398764581059329
0.4159086040591285	0.5808040666806834	0.1487084339898522
0.7500990701281463	0.5045234476699255	0.1399244808049725
0.7868083835315440	0.5881814254715427	0.1276765787719868
0.8743650165504329	0.5025629678302939	0.1224742703110616
0.9149006356345830	0.5858001002976511	0.1159198238064806
0.9992881423130706	0.6264643425807951	0.1104048874309774
0.0428592189932663	0.7094697452154431	0.1019667572646069
0.1254092623128682	0.6256595849781628	0.1127019929905473
0.1680801419633718	0.7088791533230557	0.1089279189467118
0.2511603449653427	0.6249300793125818	0.1195975523147977
0.2934951968775107	0.7081941342479441	0.1186294200137994
0.3762007816686863	0.6234412673940577	0.1343790769249021
0.4191900427162027	0.7075160169358853	0.1309645632768651
0.4997585549185093	0.6263514491994712	0.1687780268229448
0.5461509119390596	0.7077225457935816	0.1389939002273434
0.6806456189962379	0.7083486924887020	0.1231418572573234
0.7501416881345208	0.6387556977329737	0.1231061346600382
0.7993823483020146	0.7164260910847222	0.0965508064578072
0.8721390316582784	0.6278154943780960	0.1130907732632034
0.9183647646979226	0.7103788235735216	0.0969103158117282
0.0016869561003867	0.7512032611384436	0.0933463080673217
0.0438335621300931	0.8344375217700186	0.0836490287896602
0.1264879061337962	0.7506043248977563	0.1016112995087246

0.1685265497926388	0.8337680129939726	0.0947490747821449
0.2517834050792825	0.7499523692807817	0.1114875696877053
0.2935250909825083	0.8332742718580861	0.1071275084700485
0.3773885164692215	0.7491309168606894	0.1225068462805272
0.4188147747323838	0.8327379985711756	0.1176435099259446
0.5035391532730380	0.7490115664089069	0.1304646586158106
0.5443107856249705	0.8322960110120472	0.1216630023524714
0.6299536164495995	0.7448037717505572	0.1274727234527667
0.6695882579771264	0.8301480103182158	0.1133626932924770
0.7583449133017618	0.7575643946332916	0.0966271482280276
0.7963722596947663	0.8385170636758332	0.0872736048782466
0.8803267563356006	0.7544576557447277	0.0868391285729869
0.9206286113635066	0.8368567637801700	0.0772890276522182
0.0032466004771381	0.8772877230129947	0.0763782518458043
0.0441785666430593	0.9603396096752862	0.0732312053739968
0.1271054891148857	0.8756467584798523	0.0855667601573498
0.1686892132490431	0.9589230411016856	0.0809377126735954
0.2519027958817381	0.8750119136868491	0.0984604865237088
0.2935003109741836	0.9583835410119972	0.0950407702656054
0.3769744049091296	0.8745276951632933	0.1109202292925294
0.4183018326967721	0.9580097546352518	0.1085710029180870
0.5022512314598374	0.8742630450655234	0.1182094112962515
0.5432939706014206	0.9579028048074790	0.1151991910551646
0.6275714769396635	0.8729292683346301	0.1160242729374829
0.6682716876838465	0.9573372123276659	0.1111709050804776
0.7522318353534713	0.8764696477960380	0.0976308183475307
0.7930637172174756	0.9597940488995997	0.0945038793599052
0.8787383383231386	0.8787940892653181	0.0775888233449586
0.9191687483681883	0.9613944028849976	0.0770465478609886
0.5652878390749188	0.5251950136781955	0.4593852455019136
0.6308343424168432	0.5077984295873510	0.4760425372215307
0.6286023276796194	0.4431277926481746	0.4345216846799659
0.6874562431339436	0.4249548329856515	0.4502287336494717
0.7491994888701544	0.4714290794218113	0.5077841201972481
0.7516332709649880	0.5359036966694352	0.5497323157050976
0.6926307713546842	0.5538741110434825	0.5339661859363024
0.5940467802124608	0.6135860643456074	0.4566795181502259
0.6811347535958765	0.7320544256082979	0.3817161161067871
0.3947295333025454	0.3806270537974212	0.4451895540898537
0.3080797727529982	0.3422910507510247	0.4266684963316543
0.2792899567811324	0.3579427750591633	0.3484958607246443
0.1993682296351309	0.3218785669999953	0.3308846662011672
0.1443996259265807	0.2677739372037466	0.3912227747373623
0.1725830534379033	0.2526087027815430	0.4705427678002195

0.2527445761491727	0.2893934309327672	0.4873898111699030
0.4278248461660165	0.4594164436851315	0.4940622887134861
0.5351958651082024	0.6020767598166178	0.2217286802386603
0.6770213514273409	0.6353149941221063	0.1415412672410888
0.6445313476768387	0.4954006722994191	0.2196020775996423
0.0643295043967200	0.2326546273135690	0.3740015433734356
0.4946442845724311	0.4528553791261566	0.1937989984562354
0.6007231933904901	0.5594667496265684	0.2259531266448399
0.6947085763812412	0.6043143791496646	0.5667196025850941
0.7997471772399261	0.5724643246661675	0.5946136708369817
0.7954573126917737	0.4575722002365311	0.5199003459562699
0.6853744403453330	0.3746890864436757	0.4171063868382647
0.5806960463594156	0.4070923834863738	0.3890736576554676
0.6333953086336627	0.7441341210834852	0.3690661267058202
0.7205918857521216	0.7490623997520790	0.3242881568348165
0.7131107906164897	0.7634972992839834	0.4418655957590230
0.4266031115402114	0.3892696427699964	0.3819284746199490
0.2728653135311134	0.2758105148773763	0.5491518086900048
0.1312167792888115	0.2113030703205379	0.5190615891063489
0.3204404961165158	0.3988158525059878	0.2995777134402478
0.1788186536870506	0.3351026326707011	0.2693780832449561
0.4070343574080176	0.3421919332714703	0.4869375182698071
0.0491048628416286	0.2300830744446834	0.3089141903290940
0.0296642906533868	0.1819412991195646	0.4069482023338761
0.3933600618438481	0.4522474415331929	0.5544768235344151
0.4250108206773630	0.5038604091633950	0.4518995524217062
0.5414322055183721	0.5031164458459801	0.3918793711270068
0.5700599154383119	0.6479753116835288	0.5023452772034162
0.6506257215596265	0.6488809474577426	0.3929188354921998
0.5063588419933051	0.4870236909318427	0.5249716184271307

Total energy (E): -1410.32062518 eV
Temperature (T): 333.0 K
Zero-point energy E_ZPE : 201.987 kcal/mol 8.758998 eV
Thermal correction to U(T): 217.888 kcal/mol 9.448518 eV
Thermal correction to H(T): 217.888 kcal/mol 9.448518 eV
Thermal correction to G(T): 185.413 kcal/mol 8.040262 eV
Entropy S : 408.036 J/(mol*K) 0.004229 eV/K
Entropy contribution T*S : 135876.011 J/(mol) 1.408256 eV

RhN3P SA (pyrrolic N): IS2
1.00000000000000

19.7297992706000009	0.0000000000000000	0.0000000000000000
-9.8648996353000005	17.0865073798999987	0.0000000000000000

0.0000000000000000 0.0000000000000000 15.0000000000000000
C N P Rh H O
139 4 1 1 19 3
Direct

0.0063266936893716	0.0049395478843381	0.0801316410057658
0.0470246310387412	0.0876592519760258	0.0853978050008090
0.1310350433409350	0.0038099853947392	0.0834311561941557
0.1718828573777502	0.0867219181757143	0.0876564788589518
0.2555884917418776	0.0027102476365002	0.0935731266133308
0.2968606852548227	0.0859902280774344	0.0976201180177645
0.3802974691705138	0.0021251498023359	0.1051965028982377
0.4214753234516378	0.0855275407182515	0.1097828201190691
0.5052295970508979	0.0020901513169564	0.1113528909106653
0.5462253427332523	0.0858121238127521	0.1141984697788489
0.6301379540820371	0.0016417926522024	0.1097904447617865
0.6711134808274397	0.0856763530607089	0.1107492750293271
0.7549249191767864	0.0030935330435091	0.1005411987520815
0.7960783608721153	0.0867645462302177	0.1018922328795428
0.8802568236861428	0.0043145604738535	0.0870801553322751
0.9214299805262994	0.0874938706851837	0.0912170483312164
0.0046695180584298	0.1290788057629154	0.0901603283213018
0.0457901878332520	0.2120443168227787	0.0965560772979269
0.1299404572184749	0.1285619385776312	0.0884627191143653
0.1707373807082752	0.2114236657026864	0.0957502706905153
0.2547966644246153	0.1276233304265730	0.0945258559154633
0.2953707676926725	0.2101756883945979	0.1017564013839890
0.3796902957121560	0.1272546277475721	0.1076894026930206
0.4204162587346150	0.2101238734221212	0.1176834226140788
0.5043987971923267	0.1277037916355988	0.1161252221751460
0.5453156878737397	0.2115033071410287	0.1242784845275405
0.6294325037095950	0.1276369314896070	0.1148022802517736
0.6704045233823014	0.2114336765196878	0.1182470672016714
0.7542813572716883	0.1283200328487361	0.1077557944342203
0.7953194632762096	0.2120156089718652	0.1097629284412631
0.8793168613020582	0.1287821217645606	0.0981031099999050
0.9205323787637204	0.2121405592507494	0.1018623698345015
0.0038576209005456	0.2536451868815404	0.1014500722006798
0.0452315970544852	0.3367923396088937	0.1067216959363667
0.1290506489897894	0.2533817139303257	0.0996987792866674
0.1702791607991709	0.3362586676272445	0.1090856924960305
0.2533094090954083	0.2520977374041039	0.1022361124330401
0.2933494716201274	0.3341972713064236	0.1147931379267501
0.3775037145401464	0.2503386664625721	0.1141357346376949
0.4167534089350936	0.3315491960935737	0.1303309313997411

0.5031761146772150	0.2531790149988505	0.1303369292357970
0.5434853380132657	0.3366281686581620	0.1485663920433658
0.6284639261712196	0.2530510603459353	0.1251034964672533
0.6691191812337860	0.3367823790365391	0.1310350716642517
0.7535377411557285	0.2537509375053881	0.1152311222467171
0.7945331784777389	0.3378354375056081	0.1163011680254245
0.8786776381645927	0.2537943971812944	0.1069435176117039
0.9199397854423026	0.3372801153409267	0.1087567046456841
0.0034579577186084	0.3785747251234824	0.1085202185279329
0.0454091294549366	0.4618613204771432	0.1101372374653791
0.1286106872786269	0.3780472930655291	0.1109902312960752
0.1707813487126766	0.4610285414450349	0.1165755062388490
0.2531711494451273	0.3770830150911014	0.1183406981945457
0.2961462545895135	0.4598318253851782	0.1308568976066658
0.3746216271739829	0.3735958778698548	0.1305964080606739
0.4164196140881044	0.4522955784843803	0.1585243128284660
0.4955974295193331	0.3735270620498758	0.1579305935344867
0.6268952955392203	0.3766916783584149	0.1466002101978439
0.6739759196968080	0.4621522152678241	0.1603312597819078
0.7530693634024812	0.3796584251718105	0.1230302169062411
0.7949241848232510	0.4640237582632879	0.1186737888694733
0.8781951095007987	0.3792001479899397	0.1114646812324798
0.9198907418904505	0.4625569404967393	0.1095074756413341
0.0035146595670479	0.5036374851345321	0.1083601261022497
0.0450454786418607	0.5868259950748141	0.1058846357157052
0.1290265785919773	0.5029016536015692	0.1136006080559821
0.1707932342999977	0.5861240658038223	0.1137606212893031
0.2544544452093234	0.5019444761034714	0.1248725503239483
0.2957476701318020	0.5850949191696779	0.1259120515799384
0.3793790614843878	0.5001026602803302	0.1500278705534380
0.4187035980332575	0.5836918118480747	0.1499109750285664
0.7542517701121454	0.5066604411029521	0.1256281278127442
0.7900146511854008	0.5895400694881442	0.1126030354806462
0.8780713308177958	0.5046183427481108	0.1097667718619404
0.9184731660588866	0.5877642646306815	0.1036683054005701
0.0029700645471802	0.6285716793466168	0.1010638478873402
0.0466133657939366	0.7116378124455000	0.0951606363471662
0.1289886069512275	0.6278394230883707	0.1081692144632870
0.1717329036977787	0.7110502002369117	0.1049882295936530
0.2544435689343904	0.6271296016031915	0.1184144240454724
0.2970436837470335	0.7103227275492245	0.1156604595187414
0.3792191499540015	0.6257027884361080	0.1331463547592456
0.4228361866714802	0.7096794987151279	0.1259891509323614
0.5036570635972512	0.6291626519961357	0.1649993605026980

0.5497533363222544	0.7092217759027516	0.1296048137911809
0.6831095126025316	0.7092243053629261	0.1105684769870835
0.7524854769996236	0.6394565088879518	0.1085914233396223
0.8025892981242232	0.7180574700865923	0.0879082589914332
0.8757274810954083	0.6296752420934190	0.1003107739264811
0.9220545384139996	0.7125518151342757	0.0881621215765748
0.0055162061279176	0.7535558730308902	0.0874239014604802
0.0477759339071539	0.8369856432315107	0.0824513104508475
0.1302669359636474	0.7528326886160707	0.0975692489114251
0.1723392122814985	0.8361006085025068	0.0939260661425433
0.2554201968868020	0.7521073091785155	0.1085461682118593
0.2972805417934075	0.8354847321434039	0.1054305897756103
0.3811300076546848	0.7512765602059703	0.1182496379649999
0.4225957271595938	0.8349138544859011	0.1133834317564924
0.5072494700922974	0.7507836432325202	0.1228708815028721
0.5479183652727169	0.8342086145245734	0.1145792423835094
0.6328991996555380	0.7461340107311603	0.1165766699340358
0.6729309611950568	0.8319921203084494	0.1055778393423911
0.7615143397381713	0.7593854688063905	0.0891925953660314
0.8001776984207609	0.8407480026995716	0.0835883662216543
0.8839400217815868	0.7567604761540737	0.0805868641682837
0.9245949833559802	0.8393857437474274	0.0756289701006535
0.0071945178681667	0.8799331648012051	0.0773756201987483
0.0482380416330366	0.9630468116879540	0.0789048914467898
0.1310304516765333	0.8782034415238980	0.0868282145911009
0.1726015367593554	0.9615144249426548	0.0868092880374097
0.2556637376903249	0.8773608632091056	0.0988837228974407
0.2971846976590666	0.9607306660135817	0.0987224036175430
0.3807090266756154	0.8767326406410296	0.1087808869851164
0.4220098871665732	0.9602721152088742	0.1083266789585045
0.5059458708742087	0.8763186910715611	0.1130817487163762
0.5469911853551024	0.9600477622845485	0.1114636698678481
0.6310638819107793	0.8748580521295668	0.1091575737047461
0.6718621258664429	0.9594234121487090	0.1063090852984065
0.7558938838407303	0.8785866636762926	0.0932513160573022
0.7968940311209336	0.9620496253383990	0.0929157456153541
0.8827161041662527	0.8813810970398085	0.0771710398998260
0.9232240562465586	0.9638833265240007	0.0801294279924516
0.6251903162957647	0.5830304172465401	0.3525574032782621
0.6698974024282837	0.5614799689267174	0.4101436343966095
0.6667631433965845	0.4883025434805784	0.4036858348215751
0.7145521380709861	0.4720615151868461	0.4568394964270341
0.7661063688510981	0.5277310273877158	0.5179668468589051
0.7688201147841580	0.5996355310551118	0.5268391630984780

0.7209827575394711	0.6162409184093430	0.4741563033971630
0.6070799405741393	0.6438953926502007	0.3835272459626116
0.6325926466419204	0.7742968260800116	0.3664874732739903
0.1200155449204958	0.2149534677612615	0.4117956298265407
0.2036922488911063	0.2785373218974236	0.4265227326217177
0.2423131845896018	0.3398291855836706	0.3651863825143541
0.3199646035872023	0.3981241692968771	0.3770230394531364
0.3630278218833026	0.3968551889381388	0.4513014862553182
0.3243776738126353	0.3357549213704585	0.5135381536285296
0.2464722562861165	0.2781764353653562	0.5010955835394973
0.1137006008699084	0.1437848322568630	0.3627515043624629
0.5332639562666821	0.6002594122007747	0.2183931750803388
0.6767574078760444	0.6333995087369503	0.1245817655162088
0.6512899409758942	0.4989381590544256	0.2106540635184846
0.4415743802138810	0.4521231322118550	0.4612740271957825
0.4961859236353754	0.4527413393405343	0.2194206119596156
0.6099166257531853	0.5677737409947611	0.2256773936787401
0.7244292589385989	0.6730427798973874	0.4797558948601711
0.8089023980108968	0.6427926935187357	0.5745204055268329
0.8043038796364904	0.5150818128587357	0.5586871600584311
0.7122252797839986	0.4158783847503043	0.4506931552348866
0.6266811965917110	0.4452052778025209	0.3561920042580961
0.5716362757408812	0.7508969059095826	0.3453966429319406
0.6722212539742395	0.8246067052073223	0.3256285743196992
0.6383667416343227	0.7903069381149189	0.4372609685851427
0.0883193839243348	0.2376511118180439	0.3728358190088611
0.2181488212419392	0.2320858480806058	0.5511681707855448
0.3559383328592461	0.3341880597109563	0.5727054183616468
0.2104141032453187	0.3421943468807985	0.3068439355011502
0.3480192186114681	0.4455896824145020	0.3286594787290574
0.0897494254976895	0.1953128622737268	0.4765927556101579
0.4619576317579998	0.5051464953485604	0.4314405008715293
0.4639639899711515	0.4574672987142489	0.5234162612887555
0.1451374645277016	0.1198644768143608	0.4007993001729059
0.1415595649186036	0.1613391720651678	0.2968504081358287
0.0076348191548734	0.0664700200419417	0.4025415506754309
0.5506813967004629	0.6275640634273660	0.4318133589063958
0.6562762490991553	0.7164255610604435	0.3513743444393745
0.0342898633730946	0.0841547382995563	0.3454992516067527

Total energy (E): -1409.35528592 eV

Temperature (T): 333.0 K

Zero-point energy E_ZPE : 200.463 kcal/mol 8.692893 eV

Thermal correction to U(T): 215.922 kcal/mol 9.363282 eV

Thermal correction to H(T): 215.922 kcal/mol 9.363282 eV

Thermal correction to G(T): 185.213 kcal/mol 8.031589 eV
Entropy S : 385.852 J/(mol*K) 0.003999 eV/K
Entropy contribution T*S : 128488.797 J/(mol) 1.331693 eV

RhN3P SA (pyrrolic N): TS2 (imaginary frequency: 317.962105 cm^{-1})
1.00000000000000
19.7297992706000009 0.0000000000000000 0.0000000000000000
-9.8648996353000005 17.0865073798999987 0.0000000000000000
0.0000000000000000 0.0000000000000000 15.0000000000000000
C N P Rh H O
139 4 1 1 19 3
Direct

0.0027486782592610	0.0026053288057740	0.0754891773586324
0.0433516844560873	0.0853807179603456	0.0793923187097412
0.1274746840887527	0.0012729444247460	0.0785026006555869
0.1682121810646634	0.0842369105286200	0.0806243575258271
0.2521340045156916	0.0003068929538795	0.0897357481401858
0.2933580438253033	0.0835448649259015	0.0917721244816549
0.3768722488445832	0.9998856476038885	0.1034891529434670
0.4180929455385298	0.0833628520164269	0.1066872707016634
0.5018642384916877	0.0000974594503270	0.1116908459637318
0.5428873933399189	0.0838162132201052	0.1139683262996287
0.6268456738503461	0.9997115534467220	0.1109422981478645
0.6677920677520979	0.0836788115629361	0.1117544250467504
0.7516233404736862	0.0010947632648102	0.1003034242409600
0.7927728240458950	0.0847356420254072	0.1014989692637842
0.8768007090965085	0.0021840025454431	0.0841533061156310
0.9179500371453921	0.0853650423804886	0.0878913839455441
0.0012032051332649	0.1268695181230479	0.0852187751481828
0.0423582683684620	0.2098855632453122	0.0919226248580994
0.1262749165337995	0.1261117978587616	0.0809877516501043
0.1670122755648134	0.2089666357844615	0.0875975628085427
0.2511225825149370	0.1249669402462597	0.0868829064029133
0.2915111085752651	0.2075020982143495	0.0925871813983311
0.3762915092632672	0.1249199349174644	0.1022287888128410
0.4170214896996576	0.2078774721822878	0.1108238847366729
0.5010660545928451	0.1256843246886060	0.1142810324052422
0.5419244267530512	0.2094045854955369	0.1224447356751998
0.6261038077697820	0.1256910814955481	0.1155981901337837
0.6671155011787612	0.2094846456772647	0.1201630986908911
0.7510267089988354	0.1263156463027326	0.1086352502796307
0.7920627554664583	0.2099783423817773	0.1117823095706768
0.8759676620598569	0.1266983012225382	0.0964554135679564

0.9171798590276642	0.2100254374769499	0.1009858608279979
0.0005304882897727	0.2515216246524049	0.0992007221234694
0.0419030854680630	0.3346456831341112	0.1055769809260327
0.1255421593835126	0.2510931622886712	0.0933808662946246
0.1668782146847990	0.3340068507086615	0.1036490189677734
0.2495990188707896	0.2493803741831692	0.0928014086450886
0.2897615069891157	0.3315732704391909	0.1054743757192255
0.3737041008786121	0.2476142801480921	0.1053797531908573
0.4124683275747765	0.3284299213908556	0.1208381752624969
0.4997259857758349	0.2510549063632317	0.1255982482837507
0.5396023697694821	0.3342878960519850	0.1452049500269859
0.6251614927354942	0.2511477136373584	0.1264544815785407
0.6657427580242649	0.3346948258400859	0.1355832495169410
0.7503315159526548	0.2517441586411056	0.1186832474441015
0.7912700804387839	0.3356874995795019	0.1219579492546306
0.8753984525218474	0.2516832963676135	0.1083608009199028
0.9166527710285347	0.3351521622329816	0.1115764525700961
0.0001252376316343	0.3764100817716974	0.1099602479022800
0.0420533672034364	0.4596995603702941	0.1123609946133050
0.1253332052393594	0.3758591060534248	0.1082598880468363
0.1675866569203707	0.4588439906687490	0.1148995329762085
0.2498326488605625	0.3747595057989134	0.1113002511721266
0.2929199952364382	0.4574739953572065	0.1252397912782190
0.3706418417157450	0.3703471802666611	0.1205300783358854
0.4127552161797954	0.4493743881840814	0.1481709283660466
0.4912614056653328	0.3703414525126732	0.1493605201490974
0.6232639472959027	0.3745675095049588	0.1507301058156445
0.6693990212150782	0.4592889897753972	0.1677651149880943
0.7497474698521197	0.3775768118195569	0.1302174932952102
0.7912469641048135	0.4619280201550462	0.1275707027177330
0.8748739484049372	0.3770613262982458	0.1167597003238549
0.9164004855312091	0.4604720895551061	0.1157466039031854
0.0000565235967473	0.5014810182935098	0.1128044596676390
0.0416651546284122	0.5846557237794249	0.1100368772791577
0.1257519377874878	0.5007020747511207	0.1141949800794502
0.1676567106340073	0.5839494432338118	0.1148545313483369
0.2513444182188619	0.4997338310373418	0.1221963571112038
0.2930803663829908	0.5829775077221863	0.1253842748832823
0.3763287193643386	0.4974913658768010	0.1432206714751602
0.4163874880247757	0.5808091352722549	0.1470329036877374
0.7500441508350340	0.5046262082424859	0.1347778726031166
0.7867463375326789	0.5880548961441835	0.1204383599988769
0.8744293821690576	0.5025373654699016	0.1177184453985684
0.9150265435862017	0.5857160852694673	0.1106447747563613

0.9994618371600699	0.6263641280758065	0.1061796045407879
0.0431083549310692	0.7094066948554358	0.0983980393821292
0.1256613811192281	0.6256087380574197	0.1104687385632686
0.1683649894914592	0.7088375499992139	0.1066015776504916
0.2514622138168198	0.6249555299301015	0.1190330349746052
0.2937735530796005	0.7081465148702416	0.1169635116317526
0.3766541346806173	0.6235130217449687	0.1334014855280261
0.4195898851994480	0.7076007825467674	0.1284253234053569
0.5012741637706695	0.6268440814673438	0.1623922147867346
0.5467806460592147	0.7084955605225537	0.1322292453722095
0.6803580334131806	0.7075194951824796	0.1144343489116591
0.7501291047776235	0.6383976378462668	0.1149070765709308
0.7998475872582325	0.7165795292723022	0.0910971338264916
0.8722350850401438	0.6276705236998823	0.1068965282782151
0.9186574110405373	0.7104019481626132	0.0921329658450565
0.0020348042064711	0.7512418088207816	0.0899644050347974
0.0442530008780849	0.8346109096126885	0.0823499320768591
0.1267898515128117	0.7505620776644666	0.0991621615643886
0.1688090442419119	0.8337609536523136	0.0938427476449449
0.2520394710161361	0.7498697730368843	0.1096705509355606
0.2938093670439951	0.8332589632013283	0.1060021933431237
0.3777643928551949	0.7491143630864010	0.1203407515619742
0.4191847056228581	0.8328145581077843	0.1154433820131267
0.5040511725049580	0.7494424288261821	0.1262851113962983
0.5447073676428809	0.8326273655097657	0.1174906186831448
0.6300075876387504	0.7446503502510434	0.1193308034397991
0.6698733491935639	0.8303134202679515	0.1071863310651870
0.7586363556032474	0.7575676902028690	0.0910824690402501
0.7967993641417633	0.8386723055160065	0.0832633956317085
0.8808608362495669	0.7547343868493901	0.0828140119722995
0.9212387717326195	0.8372436720781699	0.0751107838726791
0.0037831994477982	0.8776150317810656	0.0757942607751551
0.0446642407685277	0.9606647592956934	0.0749984132845556
0.1274637474225214	0.8757498612076974	0.0856438950442639
0.1690272271881859	0.9590806180912017	0.0833510683799541
0.2521884886921565	0.8750285806915528	0.0983257468009382
0.2937613353729196	0.9584473502549224	0.0966790288272251
0.3773196768483036	0.8745873315579693	0.1097565262080222
0.4185903796372252	0.9580914852871443	0.1083381449259702
0.5025953812601786	0.8744872741668912	0.1152815321285617
0.5436258536909260	0.9581260042248489	0.1127470110956785
0.6279062857676906	0.8730829873151900	0.1111384118604271
0.6685975461072835	0.9575446701354097	0.1073860927077286
0.7526109671734821	0.8766440626118025	0.0930983025184625

0.7934630670979397	0.9599991766626900	0.0916937006871110
0.8793096785989677	0.8791169731408885	0.0755228715379674
0.9197116181149541	0.9616897725533726	0.0768064945078177
0.5692272813626197	0.5449379177041626	0.4077662132806114
0.6351236791511086	0.5416362720659323	0.4448177009983458
0.6419359394356663	0.4745381982715767	0.4277466259012991
0.7049971076949836	0.4689347933402948	0.4598518651463933
0.7624398758875259	0.5291290383825731	0.5121040980360438
0.7556619266926278	0.5947952316034454	0.5319815809651152
0.6933492706845984	0.6014228931735639	0.4988394558953845
0.5611523528094565	0.6156125139574931	0.4014862511576196
0.6321643600393544	0.7542323504156320	0.3788954481468968
0.1606575517764478	0.2496992605528672	0.3842895824333026
0.2416388552459713	0.3077818208445068	0.4166542698907527
0.2875991993727922	0.3797095370829718	0.3722988688836151
0.3603817214499455	0.4353785979269396	0.4038798456335063
0.3936060377371308	0.4205675735623906	0.4812029292349815
0.3486966740508775	0.3453024682127743	0.5222173939995309
0.2741433208070193	0.2921352476026970	0.4922599688445887
0.1561076967200708	0.1777900291134554	0.3382443447831405
0.5397767780035199	0.6041337556807783	0.2104532323155221
0.6754522063796268	0.6330661789881372	0.1299274937966747
0.6471070239037003	0.4965152324067222	0.2201570914717925
0.4620018260586806	0.4783343371717639	0.5148393552514859
0.4954091618843267	0.4535813737117500	0.1996740246924721
0.6043200374761434	0.5618970881018840	0.2245834043934698
0.6891527064020978	0.6530062963441206	0.5146345140317024
0.7996440300988464	0.6414455836996399	0.5735994293800103
0.8119798263021629	0.5247699611286012	0.5373489142012282
0.7100109512262085	0.4177232984259963	0.4436192029293943
0.5979114843088366	0.4280006776491644	0.3861546382747055
0.5798879543888554	0.7425506948468312	0.3409586751471715
0.6855002970746922	0.7919614671422327	0.3410157718903625
0.6327041344098910	0.7819551075863336	0.4426552372325921
0.1396366881243707	0.2777692714265022	0.3370361299258445
0.2396266256699039	0.2371901849423372	0.5284162014209871
0.3726087139116110	0.3319355002082274	0.5810019863028768
0.2641593906799798	0.3923006403265769	0.3125222286594651
0.3940844078299959	0.4920573392633681	0.3711969157547353
0.1196287852991097	0.2290094385940634	0.4412145650645694
0.5209223056154545	0.4918649388569252	0.3811324867381377
0.4829367313457809	0.4559892693117082	0.5626765301293569
0.1767771599233191	0.1480874789830472	0.3840956345474707
0.1943742426612236	0.1962717145309037	0.2793544147735229

0.0437820925738109 0.1035221883752328 0.3558440296094960
0.4996411708959405 0.6161877924389964 0.3968442216929046
0.6331543186277158 0.6826527156069322 0.3951723213000253
0.0793121789165818 0.1244300855346540 0.3053030853563766
Total energy (E): -1408.34256515 eV
Temperature (T): 333.0 K
Zero-point energy E_ZPE : 199.137 kcal/mol 8.635384 eV
Thermal correction to U(T): 214.721 kcal/mol 9.311198 eV
Thermal correction to H(T): 214.721 kcal/mol 9.311198 eV
Thermal correction to G(T): 183.172 kcal/mol 7.943088 eV
Entropy S : 396.404 J/(mol*K) 0.004108 eV/K
Entropy contribution T*S : 132002.549 J/(mol) 1.368110 eV

RhN3P SA (pyrrolic N): MS2
1.00000000000000
19.7297992706000009 0.0000000000000000 0.0000000000000000
-9.8648996353000005 17.0865073798999987 0.0000000000000000
0.0000000000000000 0.0000000000000000 15.0000000000000000
C N P Rh H O
139 4 1 1 19 3
Direct
0.0003869824519851 0.0003631670914827 0.0729017308816221
0.0410955190244632 0.0831557857078386 0.0770765617053100
0.1252339491061029 -0.0007897613352163 0.0762142259548591
0.1659724490273649 0.0821717130749798 0.0782145890584907
0.2500622021519399 0.9983505077980248 0.0874967833870573
0.2913086070418688 0.0816311370612040 0.0889758825084861
0.3748576105647892 0.9979757376061830 0.1012898590413405
0.4160942454386751 0.0814305127756051 0.1038405350132418
0.4997735818528505 0.9979890510187128 0.1100689602063422
0.5408312743747040 0.0817384625481101 0.1120586812107560
0.6246983443470330 0.9975317687240838 0.1100113546511944
0.6656733343662742 0.0815417495169125 0.1106412703784781
0.7493976193390346 -0.0011781204633351 0.0994576808391544
0.7905341262896297 0.0824825348591546 0.1004348305093134
0.8744640649739344 -0.0001289265268478 0.0820942395168048
0.9156888955916272 0.0831009399411503 0.0860115343386196
0.9989177464160689 0.1246981364341679 0.0831418189755185
0.0400439682423834 0.2077355190045602 0.0898448954609974
0.1240454229816414 0.1240302041482850 0.0785613361094011
0.1647866931527776 0.2069033309781046 0.0849232321227186
0.2489358605568087 0.1230441994356270 0.0840998210238832
0.2894105003890101 0.2056044977544620 0.0889739516572924

0.3742735275869792	0.1229962761641234	0.0987455724558307
0.4151557929941243	0.2061371003458164	0.1058239256424549
0.4990363821059209	0.1236498580552407	0.1114686177744032
0.5399551288069964	0.2074365886941600	0.1186872770970036
0.6240146470111753	0.1236113410596269	0.1140501053904936
0.6650383306924459	0.2074312272091340	0.1183430813489228
0.7488418231877335	0.1241438884728735	0.1075865402185382
0.7899358755830903	0.2078513730531496	0.1105946630841861
0.8737244943918198	0.1245027520272246	0.0949898597553133
0.9149712604302701	0.2078501172943168	0.0994576780832974
0.9982977898317168	0.2493762087916773	0.0973720720616069
0.0396640377071927	0.3325164497113841	0.1037731962788162
0.1232781714821044	0.2490537909342282	0.0908840588738230
0.1645446008813371	0.3319477057895381	0.1009002272884282
0.2473691771079423	0.2475076802711712	0.0894565265436001
0.2874519192217440	0.3297378071264059	0.1013545563224132
0.3717455949646606	0.2458330813708022	0.1000137510532062
0.4109221551554085	0.3270331011433925	0.1135056008113669
0.4978623603974840	0.2492624230635067	0.1200522264081401
0.5379646095422309	0.3327478675085545	0.1373200221732542
0.6230809295265805	0.2491414402870018	0.1234683216002066
0.6638033456295356	0.3328543207062198	0.1318093087052978
0.7482422146961010	0.2496488286626902	0.1172261018274759
0.7892910061935057	0.3336563495594564	0.1204032384457767
0.8732484509898379	0.2495524724531217	0.1070689232103522
0.9145253548230294	0.3330563168174320	0.1103425086103496
0.9979865107963892	0.3743367031842649	0.1085271860553633
0.0399679023877388	0.4576445309084582	0.1109860269754527
0.1230374416125781	0.3737581050501024	0.1060556316432742
0.1653093829157063	0.4567807860897372	0.1128222236365545
0.2476840591080925	0.3729366056627380	0.1076941515203158
0.2907575706057017	0.4556614568156669	0.1217936544794684
0.3687065537343472	0.3691475181462154	0.1146472629021725
0.4108070371654714	0.4480376682460017	0.1413705240623900
0.4901119633021592	0.3692426897802163	0.1390625275227001
0.6213496624883009	0.3731180900734160	0.1442118349629524
0.6677783549575573	0.4575407313847636	0.1641278128097448
0.7478437972220434	0.3755559878017978	0.1278694700004533
0.7898524952305120	0.4600546886600923	0.1261076328962496
0.8728915491048572	0.3750122824316665	0.1156212552941944
0.9146124902119175	0.4584996395038041	0.1148238572849977
0.9981547812581432	0.4995321195217207	0.1116971161404518
0.0397465927205337	0.5827687255161381	0.1089794250212105
0.1236136859306399	0.4987077482850304	0.1125208718978235

0.1654670589564039	0.5819856241786342	0.1131476423276939
0.2489956139299187	0.4977415730513679	0.1197193061216404
0.2904872512019973	0.5809250884923423	0.1231148070755848
0.3741196859475462	0.4958376770358975	0.1399135002409468
0.4135469436582632	0.5793905798416958	0.1467627354607428
0.7493263004994937	0.5027946801977119	0.1345504558807465
0.7853474330161676	0.5861166306277908	0.1220508793483154
0.8729697246308340	0.5007055590303809	0.1170770887549678
0.9133680217306095	0.5838834710150339	0.1103009961767598
0.9976948693756703	0.6245509443157865	0.1053776769108443
0.0411695773455817	0.7075498270165231	0.0973341951803524
0.1236678106016588	0.6237676712607256	0.1090617356245831
0.1663802781210405	0.7069702587017985	0.1051537434061757
0.2491239795296292	0.6229902193566034	0.1170725685028900
0.2916957596693370	0.7062599944733163	0.1152353903346567
0.3739335382450197	0.6215632099439590	0.1317506677441997
0.4174727778986360	0.7056661265305563	0.1267252170945752
0.4976730908022170	0.6246845290279291	0.1659949180470821
0.5445128479752082	0.7054205042088129	0.1333227121987537
0.6789783808085657	0.7063247038423472	0.1176890311277102
0.7484118696326852	0.6365957811624120	0.1174075255529223
0.7971876880270680	0.7138119380914041	0.0890292839839493
0.8706639167001363	0.6258764022937929	0.1070141663042545
0.9166317263829165	0.7083320114201791	0.0908673804755608
0.0000174110890146	0.7493042196065207	0.0884999038905764
0.0421333013371127	0.8325771055918884	0.0802821226752405
0.1248180054196261	0.7486888105437211	0.0978152198693462
0.1668354761337267	0.8319002161714399	0.0922041216010999
0.2500352902553871	0.7480002154344640	0.1080521857344174
0.2918302518353219	0.8313740618041325	0.1042739515640329
0.3756899867952301	0.7472335813908825	0.1185876473841603
0.4171443985535498	0.8308463281401280	0.1137876226635413
0.5018397334571454	0.7468511912064685	0.1250686549158145
0.5425648307844196	0.8302160350759330	0.1166916653651698
0.6279586620603971	0.7426545527622096	0.1219546470310817
0.6676788184363575	0.8280701817372356	0.1079080362857929
0.7561718521612844	0.7551071475574865	0.0892316556812494
0.7943441685501575	0.8362200621019559	0.0808284854393668
0.8783477403968587	0.7521720071613461	0.0800074556542742
0.9187401054206006	0.8346904539157078	0.0718303063797239
0.0014318751691051	0.8753205894513184	0.0731343734780294
0.0423850834766163	0.9584036136925428	0.0724370531209945
0.1253797191651738	0.8738194612002849	0.0837291474721789
0.1669394301983622	0.9571049791561399	0.0812381199678451

0.2501950167638667	0.8731648907575956	0.0965332507879046
0.2917431383008440	0.9565424136939725	0.0945838196464193
0.3752504390105852	0.8726336318779983	0.1080016572438881
0.4165481560587629	0.9561331932422097	0.1064826311831045
0.5004997461072980	0.8722622296380939	0.1140428157102778
0.5415405672618188	0.9559430072457579	0.1115660247649364
0.6257268380457356	0.8708705793678857	0.1109586556542228
0.6664288807117250	0.9552969442160186	0.1067916063689394
0.7502912310867837	0.8742732744359198	0.0922678787484413
0.7911718725664775	0.9576373818304442	0.0904112214986065
0.8768104063800748	0.8766857873898646	0.0722802354900389
0.9172870060829025	0.9593107115387148	0.0740987033591198
0.5505470076098620	0.5339732546880351	0.4531314136806305
0.6274002844786587	0.5372051324186750	0.4745749056113948
0.6426430914753897	0.4816704061757536	0.4353510708074461
0.7125540066655410	0.4826134037315398	0.4525709754226270
0.7678689419838864	0.5388532862104558	0.5100348186386591
0.7526393152439108	0.5936757301993346	0.5503504205833177
0.6826841398849149	0.5929091656814209	0.5327102514543359
0.5563896856933640	0.6148908570801986	0.4558492884868061
0.6165335730535484	0.7387397931929276	0.3843567149435724
0.1772965515916617	0.2670373492698486	0.3782230117104833
0.2575140092381522	0.3244245195087581	0.4139164519356588
0.3095732128565021	0.3908572159199303	0.3651363485647808
0.3850627514786990	0.4428097980068657	0.3952070526356300
0.4121364074808631	0.4285013649382667	0.4760483828110928
0.3595856017172070	0.3624633425673921	0.5264715359327198
0.2841651153315522	0.3120524147789168	0.4959121700975149
0.1743193830788274	0.1940381575911862	0.3378807449012364
0.5292633013847567	0.5978422712647907	0.2204592938702814
0.6758977919514340	0.6340659270207186	0.1388229581297154
0.6446826814361822	0.4950255727425595	0.2141179862658163
0.4875909329022435	0.4763356672380781	0.5069170168925990
0.4950526967126191	0.4523372281281700	0.1904537418645871
0.5995635003740555	0.5593649043390733	0.2225680867720249
0.6716336600052678	0.6366111446264596	0.5635080770499452
0.7955919501573780	0.6377214524860193	0.5952630288103833
0.8228883289544535	0.5401543745114276	0.5230142349143642
0.7240647850021310	0.4398643341936740	0.4202241712262196
0.5997399624994504	0.4382599205347731	0.3898121803389631
0.5609399513202954	0.7301520252649620	0.3576464630075766
0.6637589888435832	0.7700719727165338	0.3361414124370329
0.6294012484055540	0.7707227449865846	0.4476876732738902
0.1599264274354652	0.2949091201851847	0.3264396199712458

0.2450336963014234 0.2616449400157145 0.5369016455123592
0.3787498892990974 0.3507989281188456 0.5901783078395592
0.2903899012604185 0.4029784311333000 0.3018499244943891
0.4222546148537309 0.4947032012127533 0.3556918405445518
0.1332244245303555 0.2483188072259066 0.4319963505009435
0.5374579402343415 0.5158173571364447 0.3821109213630044
0.5035779193741130 0.4588732810897838 0.5626264855626778
0.1902374384632349 0.1640401418649907 0.3889849412494057
0.2172442743000719 0.2112587654671252 0.2836049238013089
0.0604313481303132 0.1227145004573854 0.3447426585203859
0.5162760350195160 0.6324952831683825 0.5009593713827336
0.6117460420846380 0.6638064359774197 0.3971521182928083
0.1000885687677795 0.1410993530928497 0.2981743466663838
Total energy (E): -1410.47443145 eV
Temperature (T): 333.0 K
Zero-point energy E_ZPE : 202.004 kcal/mol 8.759707 eV
Thermal correction to U(T): 217.440 kcal/mol 9.429111 eV
Thermal correction to H(T): 217.440 kcal/mol 9.429111 eV
Thermal correction to G(T): 186.466 kcal/mol 8.085937 eV
Entropy S : 389.179 J/(mol*K) 0.004034 eV/K
Entropy contribution T*S : 129596.580 J/(mol) 1.343174 eV

RhN3P SA (pyrrolic N): IS3
1.00000000000000
19.7297992706000009 0.0000000000000000 0.0000000000000000
-9.8648996353000005 17.0865073798999987 0.0000000000000000
0.0000000000000000 0.0000000000000000 15.0000000000000000
C N P Rh H O
148 4 1 1 27 5
Direct
0.0384968653448467 0.0167668071424489 0.1247742011613431
0.0792617644140646 0.0995975288855937 0.1244773830172478
0.1632440403764444 0.0156875926670308 0.1268612390637365
0.2040576734770800 0.0985735926776354 0.1245032829337507
0.2881223119314298 0.0147724697218973 0.1338065374381473
0.3294464430056820 0.0980650434048675 0.1306587765661747
0.4130799635160196 0.0145896371510738 0.1437186351891682
0.4544066395532772 0.0980851416229577 0.1418810488333068
0.5380671050931383 0.0147944671811970 0.1519221864249558
0.5791268388576055 0.0985342143553635 0.1511266617788678
0.6629565338494697 0.0142446362799541 0.1533573953844031
0.7039541705305530 0.0982319652065705 0.1526527466974397
0.7876066733703532 0.0154991749447763 0.1447385693612491

0.8288645712015054	0.0991928087948039	0.1446696229886232
0.9126328844708866	0.0164243642854085	0.1308349629083214
0.9538733421122200	0.0996787630270661	0.1317784017443386
0.0370399737874844	0.1413175695304599	0.1276869695484711
0.0783112203601279	0.2244473472623189	0.1297686052665452
0.1621789024540508	0.1404669172347773	0.1233296748589570
0.2030633545760683	0.2235172981614319	0.1239142811992913
0.2871521372691339	0.1394938061837682	0.1257974049892161
0.3278741914062161	0.2221775923932042	0.1248831844285266
0.4125884256762229	0.1395938487652028	0.1350730285337886
0.4537352957499063	0.2229752144802405	0.1349421868478807
0.5374128600670584	0.1404812535832735	0.1474361417537615
0.5784072147161959	0.2245065324391362	0.1498567019602892
0.6622905385208870	0.1403023493603613	0.1533380979710786
0.7033316819900129	0.2241409661089983	0.1556549898226740
0.7871718336713421	0.1408428663774938	0.1502505796648766
0.8282846277922608	0.2245375806406269	0.1522684862427277
0.9119980619897565	0.1411849883601248	0.1389629596538274
0.9533891658066967	0.2245885159273237	0.1412761876430918
0.0366544510825176	0.2661692854204003	0.1365082211516985
0.0781897820434761	0.3493600861668388	0.1405482142413662
0.1616063733667735	0.2657813544773090	0.1275875494850653
0.2031245395883408	0.3488440588174932	0.1326598004624373
0.2857284735683664	0.2642294479649850	0.1244434669655811
0.3262458924541332	0.3468075996756860	0.1295514507504908
0.4105880194601274	0.2627287721840610	0.1288923940038466
0.4509574988085181	0.3451342652929177	0.1331684742237071
0.5366993605540198	0.2666360336901523	0.1450134481902954
0.5771625675501424	0.3510817906141025	0.1535358835869037
0.6614005167213268	0.2659768962993614	0.1562307060457829
0.7019181627281625	0.3495390642557263	0.1646828897068701
0.7865100088419654	0.2663207954890954	0.1570114269415130
0.8276092972374006	0.3503261814623065	0.1611526612195734
0.9116665276817612	0.2663032830055528	0.1487423722514181
0.9530642769406075	0.3497976408347074	0.1515167944314665
0.0365303740603859	0.3910600515110598	0.1478242649221125
0.0785012342304411	0.4743295658569272	0.1513159253646881
0.1616551091990289	0.3906626711306703	0.1391523938004936
0.2040444909482424	0.4735684667796189	0.1462087963526860
0.2864781450410009	0.3899034184089237	0.1345400862285022
0.3301026930024708	0.4727801724281314	0.1453126642668745
0.4086467423944366	0.3870943082052710	0.1350214841064354
0.4534315024762302	0.4676525444872040	0.1491345343700123
0.5317612871304045	0.3899922382203383	0.1460228614186947

0.6591081847234335	0.3897526653832102	0.1699982350939174
0.7031898970396961	0.4721572658176151	0.1995717769286524
0.7860376535096890	0.3920653580351002	0.1675275582193272
0.8278097104051656	0.4764137306632770	0.1695121050856676
0.9113569537869228	0.3916931293415840	0.1577905319872380
0.9529228957272046	0.4750128506843209	0.1586306612693932
0.0365683954790917	0.5160751820447600	0.1553166639318322
0.0780617379935342	0.5992779480850879	0.1551018858309076
0.1622028602950384	0.5153458107276662	0.1506812593018715
0.2039702157351000	0.5985095940487497	0.1538890778330366
0.2880150269502743	0.5145200191220035	0.1496563717947074
0.3294230735700674	0.5974912704808316	0.1566310035383506
0.4143981248260462	0.5131154392347697	0.1555508751938142
0.4529611723679343	0.5952168139032313	0.1712735490504594
0.7870080890082710	0.5190184191147682	0.1780234167832739
0.8226198165090450	0.6020269879668496	0.1700503950071161
0.9110663419792746	0.5170886145795784	0.1626615629511136
0.9514108518645591	0.6003471963205866	0.1594101242071471
0.0359159877052020	0.6411118231758971	0.1548803943464197
0.0794065656508908	0.7241053443642318	0.1493358892870877
0.1620072925452881	0.6402553460902339	0.1541158636770434
0.2046478283800654	0.7235095060789110	0.1524542719472342
0.2877537290855544	0.6395384619717028	0.1562174916813172
0.3300169098486055	0.7227366568732844	0.1578191338812870
0.4128983209141252	0.6380219733759903	0.1651526509691324
0.4556648248255065	0.7221762727551051	0.1673161276625049
0.5355905751648929	0.6393809257709017	0.1991779557337491
0.5826113071601174	0.7229133466407515	0.1764242988871168
0.7153273232186865	0.7202674764789828	0.1660058382518565
0.7839886762827862	0.6517041088800698	0.1674905166347388
0.8354137759430619	0.7306975989972475	0.1472965589428011
0.9086131824732917	0.6422890260569053	0.1587558373524007
0.9549477157181566	0.7251321758206413	0.1471034652532811
0.0381609327391441	0.7660051458981137	0.1437747977560194
0.0802429268398425	0.8492153800713170	0.1360271343843808
0.1630492356369241	0.7652850837053260	0.1482815348304106
0.2049942295751658	0.8484672677576026	0.1430152183786708
0.2883105605830637	0.7645896668302375	0.1538800384339662
0.3300783102519778	0.8479873609546033	0.1508482337057002
0.4140041571184859	0.7638330482998542	0.1614235271339140
0.4554071675330412	0.8475830004284702	0.1581205480951426
0.5400032764059520	0.7638455505837732	0.1682264886523805
0.5807227859800724	0.8471423956053108	0.1609605968797821
0.6656272049813733	0.7587506021154764	0.1674545293178289

0.7057402753053679	0.8446205531516439	0.1544027304001788
0.7943881092009891	0.7716055874360712	0.1460715407774477
0.8326072171574855	0.8526348793611519	0.1363600456944703
0.9165598871464937	0.7690893525868143	0.1396826755077789
0.9570385763545473	0.8513942853350368	0.1310198768575445
0.0395711825392023	0.8919058984117335	0.1300393900885184
0.0804591610822535	0.9748734924739364	0.1261975445242455
0.1634583537278956	0.8903850946157507	0.1365090685807687
0.2049785159827388	0.9735177706188864	0.1317016370519134
0.2883536036482889	0.8897740326326860	0.1446044778821858
0.3298807201266299	0.9730787593704421	0.1403696905682478
0.4135430481019831	0.8893619966186928	0.1529105725933411
0.4547981315811604	0.9728388283764330	0.1496537819978080
0.5386942151657567	0.8890966932944089	0.1580630053328436
0.5797930506709306	0.9727838857454667	0.1547698315027503
0.6638998786762976	0.8874895704213220	0.1560560490713206
0.7046912888053044	0.9720172377696853	0.1515247283540588
0.7884761033428060	0.8909675655695265	0.1415298039626772
0.8293591274412270	0.9742163917439117	0.1378621800867370
0.9150493031687195	0.8932296675945114	0.1292115929258865
0.9554529528927098	0.9757714705414875	0.1268324032110212
0.5675287415879819	0.4986720046866114	0.3808719876408356
0.5201942298087940	0.4197661617296968	0.4106147426595768
0.4688246960922064	0.3598271599582667	0.3519162047068074
0.4254253276714138	0.2823724050734046	0.3793251861533801
0.4310767495363665	0.2618911127500527	0.4672418949526134
0.4804961544050408	0.3203035006522116	0.5273286235556975
0.5243335620740621	0.3978045085648637	0.5002656409068271
0.6028599951761334	0.5629185174974642	0.4441420280244751
0.7016882855226421	0.7006796266155316	0.4410104291848702
0.3251566638865444	0.4328719510159475	0.3738595130275243
0.2443953789915731	0.3620612397945419	0.3680235583455031
0.1778764116383675	0.3707137919001851	0.3677488969305442
0.1026530273284480	0.3065338201216161	0.3644432922907511
0.0902869755856514	0.2297115255337900	0.3613374856927137
0.1564152996621830	0.2201738332249200	0.3609786393262337
0.2316454335343538	0.2855784813053204	0.3646194357567856
0.9912013739752844	0.0868626920843899	0.3738326717225574
0.9026406658233741	0.0361847143045166	0.3828209264288123
0.8572841361720345	0.0646698142691375	0.4221636218433712
0.7762296758614702	0.0170031987700313	0.4297564676730677
0.7395494735234740	0.9396326074784999	0.3997915017878551
0.7846774555699918	0.9102619067487387	0.3628884226714719
0.8655405329366884	0.9581928815021838	0.3542016599678153

0.0275744570996237	0.0731790556125589	0.4586210382044615
0.0615265510534637	0.1075731629066311	0.6093356059008329
0.3539057424692059	0.4573638165908496	0.4700533903441252
0.5665573995456601	0.6105379053094334	0.2506349198024199
0.7091348333416448	0.6454970031890367	0.1805388838541256
0.6751182763155993	0.5037999877111435	0.2508847845652429
0.0142718037272874	0.1674224124466007	0.3567745998085692
0.5556430838812511	0.4920103915061697	0.1390234147251994
0.6202351459177509	0.5566464883191007	0.2666027008713592
0.5628848580367696	0.4423847631148956	0.5475378086727890
0.4849953279429412	0.3048310035141030	0.5960376191608506
0.3971356668532866	0.2010969502561756	0.4889556152404824
0.3867273923595769	0.2376110052087214	0.3320454145016858
0.4631820967693454	0.3762743011391099	0.2846563021133765
0.6591404671700938	0.7201339392324840	0.4288488479732469
0.7561775981667058	0.7373794489688600	0.4047958880290815
0.7126795558723167	0.7016152196293283	0.5127368912606064
0.3254765463655785	0.4832950515107609	0.3419710060434504
0.2819567888469391	0.2766101478343434	0.3640537672078433
0.1496361162538133	0.1619022465970327	0.3566486140054763
0.1854666347652059	0.4294941067731485	0.3693182368326986
0.0521670286545221	0.3153165342184509	0.3636210461402204
0.9002875265982369	0.9352077596628614	0.3240288623307684
0.7565169076637004	0.8501796508046472	0.3386338730922127
0.6761697145479967	0.9025058388123716	0.4049735068646766
0.7418096895887545	0.0404506050153523	0.4596211063166207
0.8850218773696525	0.1241094075666488	0.4487423031981312
0.1223191320375530	0.1220027126759881	0.5983720054531566
0.0272079611306089	0.0468386934080632	0.6326782920309377
0.0588030799749751	0.1479992263052257	0.6571560640767971
0.3681428110964664	0.4231377378874702	0.3378615499428189
0.0105641890699611	0.0627340588638563	0.3194703943429961
0.4653288062117577	0.5343560871751662	0.4982192461821848
0.9714710125112006	0.1812513826118057	0.3616027458864784
0.3674568469764318	0.4146868886841996	0.5007706326100829
0.3066014291926595	0.4571103902662118	0.5098188775370495
0.5785003905731158	0.5715892805062295	0.5163162913494834
0.6719172884561828	0.6223481165138728	0.4063505203797042
0.4205548235913458	0.5345357358089800	0.4706202724197042
0.0500252437109095	0.0257484369682500	0.4623187811781996
0.0287644735473908	0.1177659993250561	0.5275168662511541

Total energy (E): -1534.9952 eV

Temperature (T): 333.0 K

Zero-point energy E_ZPE : 293.547 kcal/mol 12.729412 eV

Thermal correction to U(T): 317.311 kcal/mol 13.759925 eV
Thermal correction to H(T): 317.311 kcal/mol 13.759925 eV
Thermal correction to G(T): 270.094 kcal/mol 11.712367 eV
Entropy S : 593.271 J/(mol*K) 0.006149 eV/K
Entropy contribution T*S : 197559.288 J/(mol) 2.047558 eV

RhN3P SA (pyrrolic N): TS3 (imaginary frequency: 451.290673 cm^{-1})
1.00000000000000
19.7297992706000009 0.0000000000000000 0.0000000000000000
-9.8648996353000005 17.0865073798999987 0.0000000000000000
0.0000000000000000 0.0000000000000000 15.0000000000000000
C N P Rh H O
148 4 1 1 27 5
Direct
0.0270909604703831 0.0161590868397061 0.1204582794537702
0.0678901865430960 0.0989871260885239 0.1217095881737253
0.1517774742404013 0.0150058728506648 0.1236504541677059
0.1926190618447229 0.0979157075131759 0.1236119717237444
0.2765673032209137 0.0140639735824576 0.1317404878716799
0.3178546109840182 0.0973533979873304 0.1314674745608368
0.4015528272276143 0.0138790674978932 0.1412256061583246
0.4428473617023316 0.0973592016856178 0.1414343320654879
0.5265873475396414 0.0141022255077409 0.1485208808427105
0.5676496293850942 0.0978491742654832 0.1486306880653704
0.6515294935688877 0.0136362967961967 0.1496606510865863
0.6925134440224524 0.0976200720716139 0.1490159065279702
0.7762193982511107 0.0149295307477046 0.1409469862941679
0.8174372785839235 0.0985890988504388 0.1410572142845949
0.9012141861050215 0.0158338319817214 0.1264556417012150
0.9424834053405422 0.0990774155229020 0.1284675452281536
0.0256857173513701 0.1406760049313269 0.1252479276912785
0.0670205269403046 0.2238016610946378 0.1284942749185242
0.1508260741182547 0.1398526044112662 0.1223574766415607
0.1917610266933757 0.2228590620018606 0.1248131963876981
0.2756066391463211 0.1387909520444485 0.1276665708289397
0.3162526684720582 0.2214105105489675 0.1303934562056046
0.4009222057062119 0.1388330830990977 0.1370741001532393
0.4419536786130279 0.2221375772033061 0.1403102600782756
0.5258836555954306 0.1397621980762597 0.1467752352536178
0.5668984104037298 0.2237515969001387 0.1505826811403238
0.6508448802743698 0.1396698323210237 0.1503522581576334
0.6919083060261446 0.2235160975501810 0.1530896643029786
0.7757414230042565 0.1402318974949643 0.1466096286372860

0.8168474975825174	0.2239278196847490	0.1488235432796593
0.9006154883824563	0.1405711183217394	0.1359011926605873
0.9419822642519228	0.2239474297932917	0.1387838223770100
0.0253170394578103	0.2655088153499450	0.1349888500863120
0.0668403765269499	0.3487008746272766	0.1389394706539269
0.1503023699652205	0.2651028833850742	0.1275290952256977
0.1918276887039706	0.3481600663347892	0.1326584628042188
0.2743255667416611	0.2634723890719166	0.1286661156223476
0.3149089648958686	0.3460337698687465	0.1346830393725091
0.3987657853750850	0.2618893637511803	0.1375784230414000
0.4390084179357076	0.3441496479070085	0.1440103132562225
0.5250335676102914	0.2657845296037369	0.1488201860700911
0.5656109000478504	0.3501993412181201	0.1576478924552238
0.6499785041210877	0.2653217829255738	0.1552553975467723
0.6905758245894300	0.3488789875882569	0.1640551318183747
0.7750981389922604	0.2657272006883968	0.1538686557924107
0.8162006824338243	0.3497265311621041	0.1582782744464567
0.9002397322212885	0.2656801481635397	0.1457194479106017
0.9416284037769540	0.3491799300669128	0.1484373127516084
0.0250958518887176	0.3904238441125089	0.1450547451090168
0.0670332788467884	0.4736973581354341	0.1474878579501367
0.1503304818508198	0.3899871883586555	0.1378631024566622
0.1926890020214833	0.4729672973006256	0.1437079134857096
0.2751141970277127	0.3891715934339245	0.1360875623479139
0.3187204481985092	0.4721401557955696	0.1451221332048460
0.3970502578107862	0.3861835467671166	0.1429278261448355
0.4423651729571215	0.4674089408034124	0.1519456734981306
0.5202029087328657	0.3893465460876853	0.1531807191840932
0.6478713562908130	0.3890275046576843	0.1715805277056871
0.6918213083738418	0.4716057497406491	0.1999025295754206
0.7746884446101217	0.3914948249553623	0.1656254213158124
0.8162986124118878	0.4758541434421003	0.1675552906293003
0.8999177208201208	0.3910954117864356	0.1544915503929296
0.9414195587621449	0.4744179323101259	0.1549550693212934
0.0250555622207431	0.5154424274169102	0.1509350016686788
0.0665671435340101	0.5986252719996665	0.1498638570542443
0.1507673692141179	0.5147285635645535	0.1466656132827607
0.1925253066518356	0.5978969337275435	0.1486488049039295
0.2766482979936600	0.5139656859962961	0.1471972255874640
0.3180858236278433	0.5970393427119129	0.1522832564568514
0.4030640100687768	0.5126650155157711	0.1549986442408632
0.4416686844299227	0.5950199074894822	0.1683864063190674
0.7753526444333768	0.5184685498375663	0.1767049065985496
0.8113073285679453	0.6014932042266492	0.1669135814600139

0.8995203910548742	0.5165015372345391	0.1593438966507591
0.9399247689815713	0.5997177139721189	0.1551049434093764
0.0243921855104688	0.6404199332038338	0.1495647498218887
0.0678753746318639	0.7233823536456192	0.1433896923235761
0.1505102968945664	0.6395845668974324	0.1483407751099746
0.1931451240943395	0.7228136162862296	0.1463129469615631
0.2763386175608160	0.6389741725228690	0.1508377793635332
0.3185904714196108	0.7221507094702411	0.1520544553665766
0.4015610294595368	0.6376977208986354	0.1607848115391076
0.4442776247817337	0.7217030674238901	0.1626935247818412
0.5239499261069626	0.6388193075322983	0.1961430243873587
0.5711613259219132	0.7221703680458729	0.1733836773467241
0.7042292825013016	0.7202651700793599	0.1618968035369384
0.7731633325852679	0.6514019259510838	0.1632206135183143
0.8240413316996639	0.7301104019383627	0.1415463514640233
0.8971087723062061	0.6416386868143892	0.1542931247185335
0.9433936568676433	0.7243887895268631	0.1412554703647510
0.0266128780874615	0.7652341601938422	0.1376631479480036
0.0687185008031957	0.8484729640669590	0.1300609878385326
0.1515164295569209	0.7645573038931908	0.1422259748999760
0.1934711340753418	0.8477429817539422	0.1375584274523283
0.2768211318446819	0.7639242213425127	0.1479033282833472
0.3185696729370769	0.8472879510196835	0.1455268692852792
0.4025369355862580	0.7632661048289289	0.1562207882785251
0.4439416892993092	0.8469605264257017	0.1533431146506893
0.5286053289145167	0.7632244357445160	0.1645595690714567
0.5693312379695904	0.8465164103095234	0.1573101702789947
0.6543594586932786	0.7583358575873727	0.1644133323768704
0.6944147928448572	0.8440807737810959	0.1512245764724171
0.7829942023159714	0.7710716162791140	0.1405680507463138
0.8212046975673766	0.8521408302169317	0.1310010773004170
0.9051570602506811	0.7684290990445261	0.1333325551157593
0.9455632103084565	0.8507258247592258	0.1246453191609220
0.0281046543638324	0.8912304697807567	0.1241442446904580
0.0690087084965707	0.9742260943969588	0.1215021930677197
0.1519339915563326	0.8896525913276948	0.1313554537100876
0.1934564958086292	0.9728236524939634	0.1281513236591720
0.2768202918053374	0.8890396620787716	0.1397960759712979
0.3183507095171484	0.9723701735305271	0.1372300717162221
0.4020446718384834	0.8886796744033103	0.1482168688999014
0.4433224159126944	0.9721526799985674	0.1460397047219043
0.5272713992290421	0.8884570196829688	0.1539291871284211
0.5683415261429390	0.9721121163691454	0.1509509918175809
0.6525361443008797	0.8869447167750304	0.1527888931836215

0.6932866908115776	0.9714363265706595	0.1479240010506917
0.7771071317874205	0.8904007975674583	0.1374425885217296
0.8179542366240478	0.9736673817976647	0.1337087177777927
0.9036024102859415	0.8926298350393882	0.1233831649342396
0.9440329295255293	0.9752015461587070	0.1217855256954806
0.5581344722945811	0.5120537219935717	0.4091127672406923
0.5503056368253799	0.4397788543365128	0.4469983018510612
0.5317524468736081	0.3744652514092957	0.3928475594448930
0.5268208021028362	0.3069826348457566	0.4288052248246097
0.5382851983798079	0.3023517476482325	0.5201251152806503
0.5539953916737265	0.3657464567856850	0.5752185321847958
0.5605469129048449	0.4339268890701412	0.5394315115543651
0.6157250396622626	0.5876566378437910	0.4534698351261678
0.7330289641166954	0.7106627742631779	0.4149752018776607
0.3559493029539123	0.4224260717019991	0.3745401530317243
0.2726664280893017	0.3572452688701599	0.3680820981197616
0.2110337606105687	0.3736367182466194	0.3682694628131495
0.1333272508474695	0.3144451756010624	0.3651006810563891
0.1135866163397894	0.2351723596210941	0.3617383771430263
0.1748254026929001	0.2179913534645571	0.3605507909752660
0.2526548182384607	0.2784365764867988	0.3639735738693866
0.0061430443119622	0.0955811966674974	0.3720343388770446
0.9173655323212330	0.0506823695070353	0.3810110772640861
0.8769275134800913	0.0836797647540959	0.4225041475776415
0.7955008486816729	0.0411253436791837	0.4302763121689138
0.7535733042599566	0.9644223738649204	0.3983993781569867
0.7937625470148171	0.9305273337270754	0.3592474168790801
0.8750216870834006	0.9733906385803872	0.3503246898127481
0.0394734426800454	0.0758081024163626	0.4552973276262615
0.0761817437119404	0.1054035058099359	0.6061656052874073
0.3874369742190649	0.4438879264018873	0.4714126756653718
0.5547213199148630	0.6099694334556615	0.2487540868473347
0.6988156892231361	0.6459150042112612	0.1768132554953823
0.6635940902786838	0.5034557401721697	0.2514472436281565
0.0355328693296161	0.1778123717871361	0.3582747605061727
0.5443214088816928	0.4911318359115778	0.1386194075786344
0.6137247732640803	0.5616544614699401	0.2617442760160835
0.5711495301166104	0.4824834810842637	0.5828649131865864
0.5610813105447244	0.3621734801652917	0.6469334399002489
0.5348334346132361	0.2497331855449029	0.5482998964684191
0.5148898585520701	0.2581018086713982	0.3849567906445331
0.5241683499668336	0.3783025512475313	0.3214960346519417
0.7096600501020603	0.7506891703104712	0.4185215233182065
0.7769602510425270	0.7283666651797794	0.3621615144883863

0.7572126751878789 0.7080734491919500 0.4798247766505183
0.3625188133772641 0.4761456307214200 0.3441144670239054
0.2991536969470969 0.2636560216760725 0.3622905874261447
0.1622107966421576 0.1576259530905966 0.3556083858460338
0.2246793912075293 0.4345555430838657 0.3701661228008590
0.0867221862350157 0.3290842905814459 0.3646691389031240
0.9060668020251280 0.9470394423862880 0.3185424174643599
0.7615338904509669 0.8709228124015901 0.3335357760481252
0.6899469727193250 0.9312449453720191 0.4039861697385100
0.7649178132476290 0.0679905492313238 0.4619070759252487
0.9088580173835500 0.1425446130921587 0.4506398674226315
0.1351721208838440 0.1148634643685204 0.5940457748220684
0.0378060862696626 0.0447588497586029 0.6279030244723645
0.0777205533166548 0.1468026371392871 0.6557505925691640
0.3946484807040023 0.4068675089615535 0.3378734007317377
0.0224464827317677 0.0701242824087045 0.3161382157402421
0.5095477863468272 0.5113043901303475 0.3728981160949646
0.9960604534994302 0.1962026250854173 0.3613006680066574
0.3926257346443308 0.3950381739685673 0.5016899096524435
0.3424545840695037 0.4483159984844969 0.5120223967861021
0.6207558696192623 0.6085193258812205 0.5301940471295986
0.6709057353310338 0.6332588940338674 0.3900442124076157
0.4548782211816127 0.5153491804467463 0.4783930929319669
0.0554810887932363 0.0235601084542072 0.4564813798179269
0.0458163161767768 0.1211392753619539 0.5257490145473892
Total energy (E): -1533.44058765 eV
Temperature (T): 333.0 K
Zero-point energy E_ZPE : 291.385 kcal/mol 12.635664 eV
Thermal correction to U(T): 314.838 kcal/mol 13.652659 eV
Thermal correction to H(T): 314.838 kcal/mol 13.652659 eV
Thermal correction to G(T): 267.922 kcal/mol 11.618181 eV
Entropy S : 589.481 J/(mol*K) 0.006110 eV/K
Entropy contribution T*S : 196297.256 J/(mol) 2.034478 eV

RhN3P SA (pyrrolic N): MS3
1.00000000000000
19.7297992706000009 0.0000000000000000 0.0000000000000000
-9.8648996353000005 17.0865073798999987 0.0000000000000000
0.0000000000000000 0.0000000000000000 15.0000000000000000
C N P Rh H O
148 4 1 1 27 5
Direct
0.0211400650194807 0.0157383709314400 0.1186461406289621

0.0618256908791386	0.0985221041362037	0.1209356675049791
0.1458288632988931	0.0146744190018093	0.1230823080505626
0.1866051233086368	0.0975982578045904	0.1240352371980545
0.2705493178743466	0.0138339185710897	0.1312119708563205
0.3117610402941777	0.0970760824583624	0.1315018119403925
0.3955246433242299	0.0136261695000748	0.1397818987416300
0.4367602858144773	0.0970652933715126	0.1399048978127017
0.5205115907703183	0.0136090110910280	0.1462668934723363
0.5615846472736756	0.0973812095786788	0.1461131363618615
0.6454356812922488	0.0129404347146568	0.1472353724925640
0.6864503821758494	0.0969619485080280	0.1465237883858393
0.7701414501746416	0.0142303028433244	0.1385938824246921
0.8113105401732198	0.0978669666826544	0.1387748697114359
0.8951358733524437	0.0152335163886259	0.1238260866058039
0.9363518426720794	0.0984251893981261	0.1265717460446821
0.0196227647712440	0.1400220322903555	0.1241786647535184
0.0609200733508721	0.2231992276717076	0.1278604509651640
0.1447579791827055	0.1393830686805323	0.1226052095671030
0.1856191849238967	0.2223333247985974	0.1250895253892221
0.2695338526730561	0.1385009255455253	0.1282659276529865
0.3101131649856914	0.2211341876077048	0.1303787653009108
0.3948543674427157	0.1385509992951278	0.1361026629461972
0.4359008159099529	0.2219164860362038	0.1377673917478466
0.5199014596943106	0.1394421780030855	0.1440146233274607
0.5609364725026307	0.2234429927359319	0.1457697352044547
0.6448080059107646	0.1390765226373568	0.1473958950489730
0.6859005177896503	0.2229365535385711	0.1487453796729533
0.7696360870384505	0.1395492721368483	0.1440964313785318
0.8108126803281835	0.2232595473968937	0.1458941257176761
0.8944997851808155	0.1398876906605125	0.1339144816984534
0.9358766296506302	0.2232941599080400	0.1370138941688749
0.0192204372876440	0.2648785023033088	0.1338482301148771
0.0607612407193341	0.3480997793149649	0.1375637015784533
0.1441120572010895	0.2644695301644317	0.1271372630512152
0.1855786627863323	0.3475526857448208	0.1313574078604726
0.2681930114635812	0.2629401636659982	0.1285711146609691
0.3088076516054659	0.3456750794932287	0.1326616369637418
0.3928444356802561	0.2618374080235818	0.1355053311229021
0.4331952418022136	0.3442730789698690	0.1385169141216780
0.5190804041392473	0.2655960496964229	0.1434229070005689
0.5597150514541982	0.3502166859070064	0.1482289379886142
0.6440558122817743	0.2648408053290345	0.1490721567437210
0.6848473420811234	0.3485257988539244	0.1551332415618906
0.7691284762600337	0.2651237193179276	0.1497055606528939

0.8103380532235926	0.3491398822853799	0.1532250416851974
0.8941855172486659	0.2650283412222820	0.1432711678170334
0.9356484517299245	0.3486034267991842	0.1457750063902333
0.0190845691328100	0.3899146482907070	0.1429351821708970
0.0610276189606839	0.4732324352921674	0.1447715920088667
0.1441795713613575	0.3893815278600065	0.1362435872581439
0.1865477161810425	0.4725425703286616	0.1407236467230148
0.2688545838566030	0.3886258396396601	0.1334634762680246
0.3123684788968584	0.4716916172433939	0.1403759594627587
0.3910737609415064	0.3861091345650505	0.1374058902770948
0.4363418702900267	0.4677702347635873	0.1419701116954779
0.5148040897976905	0.3897377765081003	0.1430748518522136
0.6423843883815957	0.3891102496157168	0.1600407896010812
0.6857916682786396	0.4710613115374489	0.1876358630664253
0.7688166777475711	0.3910262019659325	0.1583018645076670
0.8105993936391871	0.4753560991451125	0.1608501242629803
0.8940058208010082	0.3905586538918114	0.1507220484853693
0.9356093183723195	0.4739713885306666	0.1511345392946913
0.0191343321990087	0.5150185231871021	0.1476535380983701
0.0606148987919058	0.5982814668947417	0.1463136480584924
0.1446826841353485	0.5143393964961512	0.1434480232056898
0.1863737121396899	0.5975550478334499	0.1445375268481020
0.2703161394718499	0.5135813001896870	0.1424891513707432
0.3116847118149008	0.5967882662664330	0.1457023372791561
0.3966977225979350	0.5123506824658208	0.1474882100116930
0.4351479637824811	0.5951417723944948	0.1607643720860898
0.7696251301432017	0.5177842809823943	0.1697947812471841
0.8058979994048204	0.6012293644937455	0.1624394612787091
0.8938438670272127	0.5160986156834775	0.1546713705917829
0.9342119634507863	0.5993589283264947	0.1510781247713846
0.0185571481713528	0.6400965178854376	0.1458546208269310
0.0620131626699159	0.7231272853707510	0.1399968195682943
0.1444386696711528	0.6392664585484747	0.1445951183303896
0.1871078863009340	0.7225287596850569	0.1426514010602736
0.2700801401832404	0.6386817910313339	0.1454370895738818
0.3125217551094912	0.7219492692326873	0.1470291200325081
0.3950575545360889	0.6376431481651997	0.1527673457760695
0.4380613945985936	0.7212948742540972	0.1559109987553025
0.5158012614582784	0.6373604864420949	0.1895082344380688
0.5643731625045774	0.7201885783160503	0.1688212483648518
0.6988662597980183	0.7206620560042927	0.1589163015846207
0.7681392557932694	0.6511677711687486	0.1589735904726902
0.8183144426500747	0.7297472222747735	0.1361953219523753
0.8914796402337363	0.6413688346876424	0.1503371016857097

0.9376415247793930	0.7241100185380835	0.1372264409423937
0.0208269605383573	0.7649550760983362	0.1342379409052571
0.0628424018662160	0.8482308828583797	0.1272564582699990
0.1455490562955404	0.7643036699437233	0.1390443063908458
0.1875043893370741	0.8475294453847660	0.1350877499830870
0.2707765760693634	0.7637217752395833	0.1440394741835484
0.3125172442763298	0.8470557228894834	0.1425184293996485
0.3962949234179345	0.7630138721740621	0.1508665325207337
0.4377884763691686	0.8465581278748829	0.1493040938691280
0.5222255055474150	0.7620852792404126	0.1588278321019868
0.5631307391627870	0.8455766581177059	0.1532652908889141
0.6484916939236923	0.7575463130930309	0.1618823214526605
0.6882994899491874	0.8431718892363178	0.1488718012034894
0.7773364652179725	0.7707938853210652	0.1359687306289166
0.8153129828170286	0.8517724439854851	0.1269735762725953
0.8994220001664344	0.7680661823577647	0.1284514883966973
0.9397128240841963	0.8504661875508586	0.1204638582248936
0.0222374466075990	0.8909684007432996	0.1212262455861251
0.0630707445988371	0.9739179192928367	0.1197663520930056
0.1459919325054272	0.8894116896040486	0.1293250387409777
0.1874423767876351	0.9726078876324175	0.1272063581631604
0.2707984293054899	0.8888106476464115	0.1376228861355094
0.3123261818811954	0.9721406190938706	0.1359383325352318
0.3959479810576426	0.8883751789039035	0.1452210291681372
0.4372899530603699	0.9718463060257626	0.1438823093452498
0.5211799529404531	0.8877822216815567	0.1503205064392076
0.5622290974534794	0.9714629959810755	0.1483367346854980
0.6463807538583306	0.8861502848656262	0.1499887154099084
0.6871002639232623	0.9706143145431460	0.1454600542631856
0.7710740207904482	0.8897157012254003	0.1347115856306179
0.8118458145743924	0.9730389001724674	0.1310860459729377
0.8977985133947179	0.8922586189889464	0.1194063183237607
0.9381054046253459	0.9747842849820529	0.1188957849652612
0.5430948595147819	0.5075500146571004	0.4500778109098497
0.5627387383108919	0.4476280109245102	0.4872474107401070
0.5740866909907459	0.3981265690558355	0.4300005595071752
0.5931751908628554	0.3440284252944482	0.4646260198452336
0.6004462725721965	0.3389000609513121	0.5566780318025522
0.5886688451592957	0.3879998807148531	0.6140702275709529
0.5700025404817721	0.4423335046092640	0.5796389434331594
0.6115835137053706	0.5903649788943893	0.4671190854097966
0.7312407677722309	0.6926623230209311	0.4050432119501601
0.3768332770108224	0.4178887317032990	0.3775744620820528
0.2915124024997847	0.3561258479827238	0.3706385025087473

0.2330228985732126	0.3769914686258342	0.3706563822028335
0.1540459076705342	0.3209635038899513	0.3673610380435832
0.1295570610060533	0.2404031549890931	0.3640252028103468
0.1878001746606194	0.2189211955110036	0.3626445362370767
0.2669249296346392	0.2761838017852132	0.3662109689899794
0.0166462742967875	0.1029684038901930	0.3715612185758363
0.9278111587438171	0.0616744921471791	0.3803149594398813
0.8901381810783303	0.0967593143472083	0.4235545876604867
0.8086954348398060	0.0568850293986949	0.4318257614629747
0.7639940933491451	-0.0191403868878554	0.3987243024637280
0.8013450536319794	0.9448831458308963	0.3575628421652178
0.8826171494259869	-0.0148833706894188	0.3480474254196689
0.0471772000445718	0.0783918996815844	0.4534698253277760
0.0843229038849947	0.1030392468838240	0.6047005229360434
0.4038988103791887	0.4337047081815146	0.4753866639467928
0.5422023695245866	0.6040938879544145	0.2448299070395650
0.6947266003178103	0.6469923731050091	0.1752370511535263
0.6553422410713393	0.5029494442626924	0.2361305152802613
0.0504904723048996	0.1862229326669977	0.3612759550261378
0.5380965593084226	0.4915243438680697	0.1209776246139548
0.6060146850036880	0.5617995880904610	0.2438076200395749
0.5610128546492551	0.4811372800650672	0.6237738186874450
0.5944031512069746	0.3841346967502257	0.6860084362969533
0.6152852277128247	0.2966848185067276	0.5839099443379786
0.6022655904925361	0.3059221540228747	0.4195110691164551
0.5687120324323902	0.4023756580269928	0.3580647737241896
0.7150709052310068	0.7384113780880384	0.4057087340237063
0.7676048868342703	0.6990520673946671	0.3470487730357310
0.7612569655111522	0.6946661017771325	0.4673197323521248
0.3866984119349775	0.4731592716882257	0.3479960444381899
0.3107109491542696	0.2577196177618556	0.3641407777398261
0.1718664464764942	0.1575790742024600	0.3572663198500353
0.2499405736760006	0.4388628264608315	0.3725397098433529
0.1100929511957748	0.3392295563949844	0.3668878996307583
0.9114892594906900	0.9571229339262505	0.3149081690712613
0.7669163845670344	0.8856822275093840	0.3310725764354981
0.7004218860837289	-0.0502806700628123	0.4051363214368530
0.7802434320452424	0.0852481232201543	0.4650209976402646
0.9242955768989213	0.1550054958609532	0.4527440306891533
0.1419412983556079	0.1093559419135155	0.5917020899041140
0.0434273611388358	0.0425460902803592	0.6248680037904286
0.0883864040666556	0.1444249212788225	0.6558181362559858
0.4125871131064994	0.3995001644486169	0.3394572820412604
0.0309724755060578	0.0770933418619373	0.3143879530064381

0.5357274023967180 0.4988908995910889 0.3764627570318860
0.0131337583502323 0.2072944551431721 0.3601510564643594
0.4074216744175912 0.3837260207938887 0.5032200448852472
0.3616125289939034 0.4414711659178397 0.5153226148863720
0.6233650222781835 0.6280522481241658 0.5348182844093384
0.6614282563987189 0.6162783398517919 0.3951138106814646
0.4766770326371541 0.5054426600219792 0.4887585238956074
0.0592534417332466 0.0234852783632669 0.4523712272812667
0.0559985899384714 0.1229836767143151 0.5255613221371904

Total energy (E): -1535.68966844 eV

Temperature (T): 333.0 K

Zero-point energy E_ZPE : 295.087 kcal/mol 12.796166 eV

Thermal correction to U(T): 318.183 kcal/mol 13.797734 eV

Thermal correction to H(T): 318.183 kcal/mol 13.797734 eV

Thermal correction to G(T): 272.212 kcal/mol 11.804216 eV

Entropy S : 577.613 J/(mol*K) 0.005987 eV/K

Entropy contribution T*S : 192345.176 J/(mol) 1.993518 eV

$Rh_2(OAc)_4$: IS0

1.00000000000000
20.0000000000000000 0.0000000000000000 0.0000000000000000
0.0000000000000000 20.0000000000000000 0.0000000000000000
0.0000000000000000 0.0000000000000000 20.0000000000000000
O C H N Rh
10 17 20 2 2

Direct

0.4544905755845767 0.4378882305432782 0.6339161605416374
0.5659654530379254 0.4585810414747719 0.6454870840081415
0.6093770743801949 0.5557438229032471 0.8843241542464311
0.3952167650798357 0.5452418181775071 0.7900849530280262
0.4062728415620169 0.5509759923072243 0.9035418305723318
0.5984328672385890 0.5573195744102332 0.7705551012761893
0.4895910795614667 0.6527994427199476 0.7799814782190340
0.5102274110070742 0.4508617853021994 0.8963156789712552
0.5035347634808963 0.4481970975308569 0.7823528326645197
0.5065020359357907 0.6545961016369956 0.8928595142690104
0.4918097898189785 0.5503096026284646 0.6549668860975478
0.5359016338013888 0.6064135246414817 0.6324629352681389
0.5123728232570709 0.6726909461219197 0.6355509854885046
0.5527238086004354 0.7258514489920750 0.6156650763705702
0.6174103339634841 0.7142433349863822 0.5920110626737286
0.6410173295462227 0.6486542267439250 0.5886413310170010
0.6010537597002874 0.5950614225968165 0.6086265509629667
0.5009292065029122 0.4765840442860306 0.6430503038438795

0.5775328283143542	0.3873211612834092	0.6413867151155553
0.6327045766421269	0.5584380585600797	0.8247021317783179
0.7076082042011994	0.5627633772816965	0.8174762261260442
0.3717256379676468	0.5481562523478433	0.8498049009121457
0.2967436512535214	0.5497273547656600	0.8567948521508157
0.4977839906885719	0.6828059103904029	0.8361388841090336
0.4973180567895918	0.7581430283576860	0.8341277241460586
0.5084817573838123	0.4205470436337191	0.8400198495694161
0.5140131292702181	0.3453726378885068	0.8408512511833545
0.6205873566918551	0.5444775645695441	0.6062080401576588
0.6913755740599062	0.6386270343674885	0.5702006102616149
0.6490001065034876	0.7558563023294833	0.5764047229638403
0.5334224196018609	0.7767478604440703	0.6192105768132482
0.4627983136389100	0.6833547062498186	0.6554225540325559
0.5499611295094312	0.3617717773103652	0.6813154006258626
0.6315351214543971	0.3813339135152448	0.6479127144083606
0.5614901357669816	0.3679286990263211	0.5926059392699851
0.7296335424946883	0.5135376802540771	0.8281521542807323
0.7278432406304207	0.5982510735628427	0.8540612459782124
0.7210271292237158	0.5777850348471083	0.7665767925275285
0.2811749677403506	0.6012193240800822	0.8682188053139093
0.2810270137948175	0.5179760050174119	0.8986287707376492
0.2727036567491991	0.5338411232594281	0.8102642921114117
0.4485402173231630	0.7757926025707518	0.8160847466692146
0.5353041152549887	0.7755217296352691	0.7984750245309616
0.5072316364537722	0.7788161261185138	0.8838231274920704
0.5660991300667703	0.3312963166031603	0.8300930464831022
0.4821557878105692	0.3236273395745781	0.8018998627284735
0.5005339787769220	0.3256930453821122	0.8901289266531793
0.4257836858107579	0.5643305189802749	0.6512593587658435
0.3703377397327505	0.5764068188647807	0.6504886554987356
0.5081212685882339	0.5527634498813601	0.8961468061309042
0.4963614647518612	0.5504954410435567	0.7768113369552768

Total energy (E): -325.75761383 eV
Temperature (T): 333.0 K

Zero-point energy E_ZPE : 225.617 kcal/mol 9.783678 eV
Thermal correction to U(T): 250.170 kcal/mol 10.848391 eV
Thermal correction to H(T): 250.170 kcal/mol 10.848391 eV
Thermal correction to G(T): 201.185 kcal/mol 8.724189 eV
Entropy S : 615.478 J/(mol*K) 0.006379 eV/K
Entropy contribution T*S : 204954.314 J/(mol) 2.124202 eV

$Rh_2(OAc)_4$: TS0 (imaginary frequency: 389.252429 cm^{-1})

1.00000000000000
20.0000000000000000 0.0000000000000000 0.0000000000000000
0.0000000000000000 20.0000000000000000 0.0000000000000000
0.0000000000000000 0.0000000000000000 20.0000000000000000
O C H N Rh
10 17 20 2 2
Direct
0.4561440010330692 0.4437788052772449 0.6303309075005866
0.5675135433697054 0.4572023347224697 0.6491215839895692
0.6095738406774573 0.5563322668984985 0.8859844448484034
0.3964810369161711 0.5477202860660131 0.7877502254968408
0.4068860790753512 0.5503320038128593 0.9012554579823955
0.6002243693379657 0.5578873921418990 0.7720428367805181
0.4914042224505935 0.6543627570066536 0.7794343277433902
0.5109391844063131 0.4513979324154520 0.8950101792870121
0.5028664686056116 0.4486887172412252 0.7810102807941375
0.5063227389763452 0.6548941232875533 0.8927267552120232
0.4977433648513666 0.5506852131928920 0.6692930171779035
0.5364249177254916 0.6055720094929453 0.6373254091844310
0.5116981841371052 0.6716138223670057 0.6382979924040519
0.5497289662080782 0.7246175185288328 0.6141042603832856
0.6139541518684618 0.7134759755114763 0.5887725148166090
0.6387181907389430 0.6482809733977918 0.5865109331130302
0.6004636508625428 0.5947166550677495 0.6098220934824484
0.5034750396488878 0.4792627235794104 0.6458329073415358
0.5752763799084545 0.3858557940291365 0.6400958087707510
0.6336546639773610 0.5588164876623229 0.8268028588549172
0.7086912191471374 0.5625378556103342 0.8206081664768105
0.3724952951039446 0.5487614797994115 0.8475486859770944
0.2975504673543675 0.5493690419966716 0.8544296152234372
0.4984313018049576 0.6835673936029110 0.8362536177826285
0.4971763669695457 0.7588989861491979 0.8349312450725578
0.5083355504743079 0.4211979136592521 0.8387961698382445
0.5133978375775770 0.3459539183363277 0.8401035904773979
0.6209128368403161 0.5444189689274659 0.6078374745643205
0.6883622010469562 0.6386318195255045 0.5659993773382325
0.6441608434691979 0.7552003061515939 0.5708066205390612
0.5292324081322292 0.7751564236192097 0.6155682324726024
0.4624187717228666 0.6815557808259564 0.6591118556912144
0.5460317925238131 0.3591949886062563 0.6780903738798494
0.6288075900445013 0.3764210016313113 0.6464461093814158
0.5585246082211486 0.3705645883292850 0.5901054983447789
0.7299813242233947 0.5128866724035749 0.8308047491794314
0.7286480246930582 0.5971667237725641 0.8581566003969975

0.7231081441301370 0.5782265015279284 0.7701981223288069
0.2813617057571489 0.6007035594524608 0.8657023586726330
0.2825477235020935 0.5176306638587698 0.8965442880626920
0.2734931777589826 0.5329288107724580 0.8081171821166192
0.4481375998873201 0.7760963950522116 0.8171400144269576
0.5348970167876111 0.7770995926444669 0.7994256934471793
0.5068336611141144 0.7790542246734629 0.8848770067284494
0.5653112221780540 0.3314105558332570 0.8291879287016182
0.4810888240186489 0.3238762656014622 0.8017305441441963
0.5001946373419428 0.3270199098919204 0.8897635703581105
0.4148973629421092 0.5635611338895049 0.6440617104536636
0.3612175378760997 0.5732004413424399 0.6582900822995446
0.5084647809319028 0.5530752730738616 0.8954364024757590
0.4972637244136129 0.5518836151558794 0.7745017709374931
Total energy (E): -325.28123673 eV
Temperature (T): 333.0 K
Zero-point energy E_ZPE : 224.131 kcal/mol 9.719262 eV
Thermal correction to U(T): 249.817 kcal/mol 10.833089 eV
Thermal correction to H(T): 249.817 kcal/mol 10.833089 eV
Thermal correction to G(T): 198.089 kcal/mol 8.589965 eV
Entropy S : 649.935 J/(mol*K) 0.006736 eV/K
Entropy contribution T*S : 216428.514 J/(mol) 2.243124 eV

$Rh_2(OAc)_4$: MS0
1.00000000000000
20.0000000000000000 0.0000000000000000 0.0000000000000000
0.0000000000000000 20.0000000000000000 0.0000000000000000
0.0000000000000000 0.0000000000000000 20.0000000000000000
O C H N Rh
10 17 20 2 2
Direct
0.4638531242846153 0.4605022761431006 0.6225051945044703
0.5724426105568993 0.4476560629905517 0.6558955289212536
0.6104179592069482 0.5582501824761879 0.8945481813727194
0.4011357863343372 0.5506922161638310 0.7870458147850070
0.4072238581296598 0.5501842090660785 0.9010687699983382
0.6060109610970735 0.5577001544550757 0.7804242047257536
0.4995574439110146 0.6561723470366745 0.7824428147285155
0.5122404068838270 0.4520984438461175 0.8969780433821352
0.5037873491269039 0.4503928660653961 0.7829860512714988
0.5051504230172381 0.6561316780877295 0.8966338983467007
0.5150593489315191 0.5495525542967317 0.6797703692875966
0.5418852378639639 0.6044362844361005 0.6409369555303055

0.5118331585835461	0.6684469435653378	0.6410176925977262
0.5425395067122591	0.7217597618262602	0.6086572841724466
0.6046051001482344	0.7131840192812081	0.5774151922871332
0.6348640150683689	0.6501291358270861	0.5765325222331349
0.6032615198313330	0.5957978289451885	0.6064345316623951
0.5144296160111993	0.4821167121753390	0.6495173549797827
0.5694525469833350	0.3784043658531049	0.6349618041521847
0.6370797323865849	0.5594227076507868	0.8365267779552777
0.7122688605967851	0.5621052323790460	0.8323840149994365
0.3751396394286279	0.5494805176066280	0.8458885822941942
0.2999194939820917	0.5483669038401053	0.8494752609232963
0.5011742592845609	0.6849882587256398	0.8398782549867135
0.4973990698442862	0.7602850529649249	0.8385677532413895
0.5087712680310631	0.4224051139471411	0.8405945511073742
0.5119286325330691	0.3470612044523583	0.8404441215604561
0.6269312330542033	0.5467054054082450	0.6063004586520355
0.6831399678923387	0.6432203713740493	0.5520960867758398
0.6291976436051021	0.7555220975720127	0.5533701541838169
0.5184150722454415	0.7706838099110220	0.6082759114145937
0.4641610964563170	0.6750214992184661	0.6662901271232207
0.5347181485322011	0.3508114831389727	0.6673452926140373
0.6204648169024898	0.3594309989472281	0.6406821940738978
0.5527658603560960	0.3744126120961573	0.5828326634838129
0.7323151814133473	0.5115247241970377	0.8402627762524052
0.7321305504213859	0.5945182595016504	0.8718482609065072
0.7280437691398702	0.5797150094402371	0.7830031690629955
0.2819664238144853	0.5994058795344397	0.8593169349579067
0.2838184014167396	0.5166861784171807	0.8912036447882381
0.2784318483837204	0.5309148541771515	0.8022570732025078
0.4476098460840040	0.7755091653907812	0.8209391000197236
0.5341769979670020	0.7799978124533626	0.8029089430231647
0.5061694665262455	0.7808330128378491	0.8885228667199744
0.5631150957431950	0.3315065600076784	0.8276665752003800
0.4781443706648830	0.3267692301696219	0.8023195159104521
0.4993543396196944	0.3273367353805499	0.8899376382124381
0.3475567593644743	0.5889757652448407	0.6313522406652516
0.3094544066894764	0.5579975143128295	0.6575443911917366
0.5088331413018405	0.5541240015862885	0.8989568986798824
0.5027848516361084	0.5534141145786113	0.7773655328778950

Total energy (E): -326.00401830 eV

Temperature (T): 333.0 K

Zero-point energy E_ZPE : 223.545 kcal/mol 9.693820 eV

Thermal correction to U(T): 250.594 kcal/mol 10.866768 eV

Thermal correction to H(T): 250.594 kcal/mol 10.866768 eV

Thermal correction to G(T): 193.740 kcal/mol 8.401385 eV
Entropy S : 714.334 J/(mol*K) 0.007404 eV/K
Entropy contribution T*S : 237873.165 J/(mol) 2.465382 eV

$Rh_2(OAc)_4$: IS1
1.00000000000000

20.0000000000000000	0.0000000000000000	0.0000000000000000
0.0000000000000000	20.0000000000000000	0.0000000000000000
0.0000000000000000	0.0000000000000000	20.0000000000000000

O	C	H	N	Rh
11	25	31	1	2

Direct

0.4610861833912531	0.4426090858578458	0.6573377932049461
0.5702093509689858	0.4298204531840690	0.6864443279828653
0.3752510949252670	0.5409363436216953	0.5990693894469635
0.6081464612930939	0.5398054916730807	0.9305055651526737
0.4061399251151044	0.5430599453020933	0.8110152631872735
0.4051537844747357	0.5332198549481730	0.9247392827969734
0.6112167032830815	0.5322517596331434	0.8164736343607968
0.5155806839239518	0.6403940095651326	0.8139188587893743
0.5097919929767442	0.4346489925586925	0.9263526859751436
0.5013463823257738	0.4347125842997350	0.8124587829899299
0.5047485102596093	0.6384762098264762	0.9277379604549877
0.5147128002412762	0.5340490962930199	0.7084371943335758
0.5189936144474291	0.5870454323425147	0.6599429669423751
0.4894856454445164	0.6505868667061534	0.6710367284630435
0.4940261139645352	0.7004493657169821	0.6229447273607523
0.5290198225313133	0.6892048043795092	0.5633815435168771
0.5588169512816289	0.6270080601892845	0.5514113521183026
0.5529833111956903	0.5761967819891440	0.5983710896982958
0.5133299383812624	0.4653519767381787	0.6811383663417158
0.5645069405051487	0.3602203255672171	0.6668412159872468
0.2791262832371032	0.6043186968971584	0.6380281438579857
0.2039710074683746	0.6047665455450676	0.6439298197880244
0.1715501179014680	0.5954737220435075	0.7053990069582756
0.1020529296839277	0.5920124319495593	0.7102207693582189
0.0618511998459922	0.5975784025556965	0.6528139512825720
0.0937955964200301	0.6078579698277813	0.5909912883145668
0.1632792437821689	0.6112715276137836	0.5870623373351485
0.3057010553415505	0.5363462523695020	0.6138643337553176
0.6385689654226023	0.5362568398826958	0.8744655381715257
0.7139220849153871	0.5356359545380155	0.8762971620568119
0.3765384270458227	0.5372580101611295	0.8678314612569025

0.3011723581562481	0.5359933739324719	0.8667444758474253
0.5101814377993839	0.6683157988143501	0.8716198509638055
0.5094586799221119	0.7436820314150954	0.8721655045784624
0.5063154809861737	0.4057833828471938	0.8696476002045100
0.5101896946124312	0.3305477865216119	0.8679421314087251
0.5756261484544895	0.5275324082454689	0.5885656706102941
0.5859472805851270	0.6182618899885353	0.5049551672582628
0.5325226318454741	0.7287826929889525	0.5260072304496014
0.4698153228524695	0.7485863891641746	0.6314874366546530
0.4615637004587332	0.6587374918083997	0.7168781772885221
0.5214982210369853	0.3370706349571280	0.6917394677562317
0.6113755052588002	0.3368382020353365	0.6826408474167704
0.5582112908222340	0.3563139227993404	0.6124842501889334
0.3027703540549050	0.6163040936917009	0.6862937279338606
0.1866003742755501	0.6194761216250266	0.5383289036007524
0.0637156467193716	0.6131015970886335	0.5456692734909770
0.2014624546699947	0.5911421691524857	0.7509279738342265
0.0785144293791381	0.5846980638011091	0.7589815419937340
0.2960800329515950	0.6423403670391533	0.6019117969998532
0.9716591421764118	0.6002114108267155	0.7017567753608946
0.2778310184450942	0.5215009295543500	0.5684059862006653
0.2958373652322021	0.4978768474668633	0.6522301267498352
0.3993476577073600	0.5050507351961547	0.6222289577001391
0.9660423391181313	0.6104614966442234	0.6183581406937462
0.7308815195303856	0.4848104590902705	0.8886862702736398
0.7317101158854017	0.5693392258770382	0.9157253301611249
0.7346896240525448	0.5498564894209163	0.8276881809406462
0.2822132644669418	0.5870144069342373	0.8747086543558441
0.2827674531125201	0.5045707353626425	0.9077125475606264
0.2827035893973336	0.5178086912210015	0.8184927041582262
0.4612618113379716	0.7613892383165286	0.8524958274231333
0.5488775992519357	0.7630817585154012	0.8393508319106906
0.5159109907326503	0.7624116549052743	0.9231976984200186
0.5623820801984027	0.3162751969885596	0.8577284340307796
0.4790229418393726	0.3106983332803965	0.8274027929762549
0.4953170112952223	0.3091811777849429	0.9161367177242205
0.9924494274650258	0.5901428514710082	0.6566048243939654
0.5066648477113622	0.5367393818501530	0.9290314884580714
0.5085261712076702	0.5374669866011285	0.8073041557884209

Total energy (E): -437.60932121 eV

Temperature (T): 333.0 K

Zero-point energy E_ZPE : 329.610 kcal/mol 14.293233 eV

Thermal correction to U(T): 361.297 kcal/mol 15.667320 eV

Thermal correction to H(T): 361.297 kcal/mol 15.667320 eV

Thermal correction to G(T): 297.574 kcal/mol 12.904033 eV
Entropy S : 800.651 J/(mol*K) 0.008298 eV/K
Entropy contribution T*S : 266616.675 J/(mol) 2.763288 eV

$Rh_2(OAc)_4$: TS1 (imaginary frequency: 201.355029 cm^{-1})

```
   1.00000000000000
     20.0000000000000000    0.0000000000000000    0.0000000000000000
      0.0000000000000000   20.0000000000000000    0.0000000000000000
      0.0000000000000000    0.0000000000000000   20.0000000000000000
   O    C    H    N    Rh
    11   25   31    1    2
Direct
  0.5007484704899222  0.4288202164383625  0.5956920957832030
  0.5573702767589214  0.4470138871566541  0.6917658070664690
  0.4063936185593438  0.5041449880072714  0.6497050170540062
  0.5963459801785365  0.5331964803596879  0.9165195979670417
  0.3812675165044614  0.5345418293562578  0.8206101129502428
  0.3936468448764339  0.5391396452313650  0.9341734985182721
  0.5857706608431856  0.5323861685545483  0.8026815153145683
  0.4847487269765088  0.6352429580329364  0.8101686211250627
  0.4927517133127437  0.4347019411169990  0.9282280930009362
  0.4811452501655060  0.4302058597635803  0.8145874768471432
  0.4987126763621443  0.6381480373883520  0.9233508122553072
  0.4699932240959209  0.5252983772286312  0.6615010768570726
  0.4959903856427085  0.5889991853853338  0.6314346602671068
  0.4882345692470539  0.6528490239470131  0.6596052037304857
  0.5129617152274020  0.7093371722924163  0.6267401068385317
  0.5449418165849679  0.7035753259726663  0.5648562934332724
  0.5527320616501452  0.6404073758141360  0.5361887662356809
  0.5291139266659014  0.5836793514877157  0.5692562676167865
  0.5111665618642951  0.4625779659102660  0.6457420696895001
  0.5881545279332768  0.3822132833188652  0.6817265670498861
  0.3184409721168038  0.6016098010648393  0.6533126355761737
  0.2423828598761607  0.6022627332827944  0.6517103889512602
  0.2044600421494451  0.5966288956931836  0.7105538614899820
  0.1348076362360056  0.5931931665506848  0.7093165349120121
  0.0997542213741242  0.5954453610214949  0.6483372897723068
  0.1373248214883996  0.6003870264382396  0.5890450578965031
  0.2069718860227586  0.6033879812103219  0.5910580976004116
  0.3387956842524232  0.5283229740265085  0.6617584989040262
  0.6198561934271094  0.5318076261931712  0.8568532100995425
  0.6949041684596965  0.5277311098209423  0.8504550211876456
  0.3586531149054671  0.5377813797553871  0.8806413770376109
```

0.2838565306174156	0.5416085927096055	0.8892361353552478
0.4930890609270096	0.6657720050947470	0.8659511028432350
0.4968225919492156	0.7409914362717699	0.8627581005669378
0.4869018520320268	0.4034826682227989	0.8725732492444631
0.4886261464686902	0.3281816698322977	0.8757269264091776
0.5349759483273721	0.5348225757954065	0.5458484677198798
0.5770959790361189	0.6349509779455479	0.4876570994115057
0.5638177197776394	0.7478990122322638	0.5391947104182220
0.5076701945509362	0.7584431812588911	0.6499969465450279
0.4653304860534545	0.6581864023034146	0.7085981715836045
0.5492382054934327	0.3439687641679864	0.6753950575426400
0.6175092179310386	0.3727604609609956	0.7269023906335101
0.6200905839909743	0.3825258838778728	0.6370808856091886
0.3374403134552769	0.6318391837489804	0.6951277439679430
0.2343831862510104	0.6068883750727448	0.5439381413722280
0.1114350771131508	0.6017584836730950	0.5409391792874345
0.2299727649095245	0.5948138581415690	0.7587870328207373
0.1069326674937418	0.5886371773839285	0.7560989816954655
0.3386625789532731	0.6224820275699606	0.6066627308115421
0.0056248730265935	0.6027966380621189	0.6889557366676146
0.3111291009296316	0.4983067688147830	0.6250436818546143
0.3257682229992258	0.5110084712531576	0.7122816277334875
0.4017195401838198	0.4920472970995285	0.5421146939206533
0.0074396875902263	0.6073897989648559	0.6047582002193449
0.7107857024299696	0.4760745188046531	0.8606966877132678
0.7187416405484701	0.5600106922357042	0.8878275475676667
0.7107952877306331	0.5414852112285408	0.7999159723506802
0.2682569455194685	0.5942714244037245	0.8860363492296827
0.2696355494507929	0.5225787952467575	0.9385830444311346
0.2583319597095570	0.5136244673883685	0.8496407605181925
0.4510095460040594	0.7607959621295429	0.8398474734824074
0.5389126079407812	0.7556946747580710	0.8306594222161646
0.5033064136624180	0.7621569854815626	0.9127953999974227
0.5404125665702809	0.3113734758257335	0.8678307723632152
0.4574445940428749	0.3065287683694002	0.8362339289788399
0.4723882519282113	0.3108644486482655	0.9250909547823208
0.0302664248422502	0.5899375701431230	0.6465447763738943
0.4952349564124438	0.5363613345089343	0.9270186977309185
0.4831799248392835	0.5325532249461463	0.8074989228653372

Total energy (E): -433.89998894 eV

Temperature (T): 333.0 K

Zero-point energy E_ZPE : 323.724 kcal/mol 14.038019 eV

Thermal correction to U(T): 354.526 kcal/mol 15.373712 eV

Thermal correction to H(T): 354.526 kcal/mol 15.373712 eV

Thermal correction to G(T): 293.028 kcal/mol 12.706898 eV
Entropy S : 772.698 J/(mol*K) 0.008008 eV/K
Entropy contribution T*S : 257308.339 J/(mol) 2.666814 eV

$Rh_2(OAc)_4$: MS1

```
1.00000000000000
    20.0000000000000000     0.0000000000000000     0.0000000000000000
     0.0000000000000000    20.0000000000000000     0.0000000000000000
     0.0000000000000000     0.0000000000000000    20.0000000000000000
   O    C    H    N    Rh
    11   25   31    1    2
Direct
  0.5401650743574137  0.4409848864255302  0.5707223815574876
  0.5394289277918820  0.4478706150295436  0.6842351027381103
  0.4325439408351410  0.5243528460279983  0.6869462719246314
  0.5882598567179780  0.5288857865610519  0.9092482959674080
  0.3670385724283493  0.5311353679568235  0.8333581947629324
  0.3882809082262948  0.5427768725148195  0.9447987230361994
  0.5687922591724962  0.5292438029434725  0.7967018002769091
  0.4675938464967592  0.6314599362333302  0.8085203642470959
  0.4827275172376162  0.4342791413226552  0.9329959872499485
  0.4676078208216797  0.4271432462040016  0.8199400419392588
  0.4945943225861268  0.6373457877129877  0.9192514588579330
  0.4672041224342724  0.5215732625427847  0.6242179262775571
  0.4996132292897835  0.5875533818862160  0.6033013841597348
  0.5050403977847892  0.6411862588332695  0.6477945278909275
  0.5337612282106761  0.7013284845968460  0.6266585010481386
  0.5576171732095494  0.7083374898261424  0.5614233526193397
  0.5526660427763462  0.6545137676171751  0.5170342854945039
  0.5237362311959560  0.5944842766550765  0.5377611200477440
  0.5194062332259185  0.4652413319963998  0.6228278370879701
  0.5907074727667661  0.3973776149496532  0.6879335746927614
  0.3336571846365948  0.5944583816363028  0.6599147836374984
  0.2583516170038233  0.5944531371462575  0.6546030353176541
  0.2178035255715992  0.5924767530080307  0.7118349850908497
  0.1482785952384217  0.5916065611402932  0.7071881827451423
  0.1163213353134906  0.5928976393909303  0.6444767111593447
  0.1566215440679786  0.5942789625088106  0.5868732935362203
  0.2261239940517518  0.5949439139798873  0.5923364122880500
  0.3604718682833524  0.5254019782771896  0.6793552142429858
  0.6071091204108143  0.5274993466265567  0.8479933190765874
  0.6812059373924030  0.5217989591213931  0.8354087872294381
  0.3490186039423097  0.5390490471804534  0.8944361434830803
```

0.2751208583222485	0.5459051288595670	0.9074355345177590
0.4832009971376265	0.6634437625288917	0.8619268040606971
0.4893724337534113	0.7382457809014018	0.8557056784089734
0.4747204549063019	0.4017038745362688	0.8783506822961568
0.4752554604017635	0.3264815625087353	0.8830912959643059
0.5202135076432854	0.5524539842391437	0.5031483306686341
0.5711059961067693	0.6593766575842755	0.4658986959487093
0.5797889009917871	0.7554522725331938	0.5450724189875334
0.5372326582039995	0.7430365159158355	0.6616863172614083
0.4865507263432374	0.6363488778186065	0.6987045152715124
0.5731309712539190	0.3505843724451121	0.6652850119094682
0.6001975204119845	0.3908698746901362	0.7414024056328785
0.6359421982265538	0.4142544959004719	0.6617999746374842
0.3508117664417319	0.6305146177641177	0.6978419875468250
0.2563481688824335	0.5965564001292124	0.5468179617797351
0.1331678230558800	0.5950696019687772	0.5375374804708288
0.2411764200592887	0.5917881802725299	0.7612303421861842
0.1179216627706193	0.5900051312079790	0.7525987389809995
0.3555545510976613	0.6099685603783070	0.6119019819305227
0.0211269308059037	0.6073797956955701	0.6798961014953563
0.3458730034366407	0.4873959132160570	0.6420312224516719
0.3405514873739065	0.5101727571953347	0.7280164550167332
0.4309896236709543	0.5073615165244909	0.5850393798135075
0.0271122721150121	0.6065521931626884	0.5959439406091870
0.6966941237200758	0.4697196847791151	0.8439972754233995
0.7088513048174907	0.5532911554421355	0.8707621029901115
0.6930581378232418	0.5355424168664372	0.7837621605053824
0.2599847603303011	0.5976671176435476	0.8965668537640586
0.2637517388275572	0.5350493570104592	0.9598712978034680
0.2468029029565428	0.5127740246489355	0.8741737021200214
0.4449509251460871	0.7586315361803322	0.8307631127814239
0.5328131244679539	0.7499207820096141	0.8241026516194337
0.4959169344915899	0.7612791651471011	0.9049032876171847
0.5267879179743429	0.3086491399943671	0.8758089370640990
0.4439075499887584	0.3045706894461920	0.8438742456083617
0.4585346173568127	0.3103832328607016	0.9327038869508747
0.0467213900531156	0.5900473158231342	0.6396621562757382
0.4885344963969778	0.5358748696097126	0.9281090575158448
0.4673144326364725	0.5291185737095971	0.8109942834301433

Total energy (E): -438.52693605 eV

Temperature (T): 333.0 K

Zero-point energy E_ZPE : 331.363 kcal/mol 14.369247 eV

Thermal correction to U(T): 361.810 kcal/mol 15.689592 eV

Thermal correction to H(T): 361.810 kcal/mol 15.689592 eV

Thermal correction to G(T): 300.731 kcal/mol 13.040955 eV
Entropy S : 767.431 J/(mol*K) 0.007954 eV/K
Entropy contribution T*S : 255554.621 J/(mol) 2.648638 eV

$Rh_2(OAc)_4$: IS2
1.00000000000000

20.0000000000000000	0.0000000000000000	0.0000000000000000
0.0000000000000000	20.0000000000000000	0.0000000000000000
0.0000000000000000	0.0000000000000000	20.0000000000000000

O	C	H	N	Rh
11	25	31	1	2

Direct

0.5856124429146945	0.6380623550921811	0.6740557582471141
0.4732479756740915	0.6389463006793292	0.6529907153250036
0.9846938009563644	0.5684929686308209	0.5143588960835146
0.6140254497772659	0.5512644083686880	0.9222077170081633
0.4071054269807775	0.5336213810813817	0.8124472612402087
0.4116293587039408	0.5358545482284711	0.9263582042911338
0.6119779846911086	0.5502992611797310	0.8081034023306657
0.4997945627179234	0.6444739685684411	0.8058729700888421
0.5206704312019284	0.4418161320576743	0.9234605606814625
0.5187690087643873	0.4396940993171228	0.8091135747045826
0.5049879551777252	0.6456656750402416	0.9200132930135183
0.5174266086492665	0.5440185047123904	0.7051276946102973
0.5249703718502872	0.4880167398366098	0.6605518897839957
0.4840021810820971	0.4305862570425063	0.6670616656508201
0.4907405114513566	0.3774436962694026	0.6227087414023060
0.5401448955075310	0.3788401787111155	0.5731594231558290
0.5818441743009402	0.4347737228279414	0.5666782598430942
0.5735450889495368	0.4894842995490194	0.6086958269876348
0.5308170728337416	0.6110270063065215	0.6762858826294575
0.4790222509294964	0.7067795036022417	0.6282570048630234
0.0802387118618384	0.6215007667256387	0.5733874148247082
0.1486189249530980	0.6083126409151376	0.6024015160621090
0.2064782949166712	0.6141472860144859	0.5630827922751607
0.2695288674537295	0.5985971731074144	0.5881329904239294
0.2776541437682098	0.5758730090207749	0.6544349370803459
0.2196996801383975	0.5703927138151386	0.6942856771971364
0.1569106566644578	0.5862096559370187	0.6684531958064142
0.0480329708158820	0.5573289438777991	0.5461324366924671
0.6419773486201663	0.5523811356515793	0.8648136157920661
0.7171303846891419	0.5551207532897777	0.8621734123039033
0.3802202732398570	0.5329982934484505	0.8710665144735401

0.3050895936175806	0.5295731720690924	0.8732068574330754
0.5001117693035440	0.6739288855563856	0.8630681796869055
0.4935103313169620	0.7488999369087637	0.8609495116908477
0.5219144948604111	0.4120059259267208	0.8670675883471048
0.5285703257207999	0.3369439944880241	0.8674800986818028
0.6058840252213002	0.5331764050851646	0.6040225593742008
0.6207062979183178	0.4357101080328718	0.5283230577958449
0.5460336430856856	0.3364367213639525	0.5393743978888992
0.4573554994369786	0.3345045616973223	0.6267166413734344
0.4457430469928028	0.4299487646253226	0.7057630918655633
0.4918881793117009	0.7405251437400793	0.6696036257903160
0.4296441572504364	0.7189494448959195	0.6080729153500641
0.5175111210010286	0.7098238048383578	0.5893128795348588
0.0833133225222440	0.6580872657550856	0.5323686531836316
0.1130925940528513	0.5814485782707379	0.7008280183428397
0.2241629712339375	0.5535382293457332	0.7460252666572202
0.2019928685489703	0.6311908783445332	0.5113786859478466
0.3135020774292556	0.6031595234631624	0.5560434899304170
0.0470120274735280	0.6430969375596831	0.6118120246689003
0.3801967265097304	0.5785337956529162	0.6551685660089319
0.3470465212235609	0.5522846562481263	0.7279107525146813
0.0436518670984540	0.5200416574353994	0.5867688232794634
0.0802531246362221	0.5354142060665268	0.5073269894401662
0.9544793878037581	0.5877356862335006	0.5472711498206735
0.7367120786534755	0.5037140695959934	0.8631488117376378
0.7367096636372423	0.5817083435545314	0.9058917077559130
0.7336663603353557	0.5789094812553799	0.8157206538917947
0.2845759310280656	0.5788106073788128	0.8597778103774019
0.2880154951349141	0.5162287549393838	0.9234525265723903
0.2867662595931790	0.4931994237371100	0.8364680780109666
0.4451173661964205	0.7621134445266193	0.8385412930257772
0.5328478494012217	0.7697593270406742	0.8288602057153369
0.4969371942100480	0.7699401353467437	0.9113517824032371
0.5801222082018711	0.3235920972533179	0.8540251318188297
0.4954003421401509	0.3148527660678582	0.8298473974779768
0.5172345530368716	0.3173518450018094	0.9173357355640803
0.3400249586436601	0.5573426757725528	0.6778182637009778
0.5129236163092870	0.5436503824761232	0.9248478148404971
0.5096326306722290	0.5421849795446718	0.8031557436270589

Total energy (E): -437.65896038 eV

Temperature (T): 333.0 K

Zero-point energy E_ZPE : 329.412 kcal/mol 14.284668 eV

Thermal correction to U(T): 360.765 kcal/mol 15.644244 eV

Thermal correction to H(T): 360.765 kcal/mol 15.644244 eV

Thermal correction to G(T): 297.690 kcal/mol 12.909051 eV
Entropy S : 792.510 J/(mol*K) 0.008214 eV/K
Entropy contribution T*S : 263905.984 J/(mol) 2.735194 eV

$Rh_2(OAc)_4$: TS2 (imaginary frequency: 169.228755 cm^{-1})

1.00000000000000

20.0000000000000000	0.0000000000000000	0.0000000000000000
0.0000000000000000	20.0000000000000000	0.0000000000000000
0.0000000000000000	0.0000000000000000	20.0000000000000000

O	C	H	N	Rh
11	25	31	1	2

Direct

0.5742092640414073	0.6263400928286955	0.6561132893787092
0.4647143488837395	0.6561852741686346	0.6457241106951639
0.0087443296563811	0.6029286441519145	0.5620455065323596
0.6019082684372670	0.5512386298992200	0.9261910320655210
0.3956474688538995	0.5404900908636149	0.8148979475120410
0.3992640621411130	0.5326602376412234	0.9285909164008210
0.5999222965989431	0.5577779874031987	0.8120173638873124
0.4893207774362874	0.6509442003518140	0.8192141246502676
0.5117782390850614	0.4402146257857894	0.9207859038049909
0.5074024440786695	0.4474729012628997	0.8069839511605823
0.4925984414577004	0.6427443544081903	0.9331793986088858
0.4889767268224137	0.5448455830330696	0.6767203338889507
0.5172670694534274	0.4854843387766252	0.6475267647596944
0.4886535182293469	0.4220800890352331	0.6619084786366450
0.5119885897589769	0.3646397865141602	0.6304353881031004
0.5639943866474006	0.3683522348726236	0.5837216275925559
0.5926434094036733	0.4304774804309160	0.5687096806843631
0.5698771080107642	0.4882125426348119	0.5998982087106762
0.5146380937783614	0.6119442243630316	0.6592947534292688
0.4873061441552747	0.7221219497469344	0.6266317691265445
0.1222790191966038	0.6400487149936753	0.5957604351876900
0.1928931103360051	0.6163159815580423	0.6064788913556348
0.2369224152870229	0.6087266883225960	0.5519835771317385
0.3004930865787165	0.5822491810116508	0.5603395386225130
0.3224396345068675	0.5583355908455079	0.6240349768844551
0.2788412492623150	0.5696274040006286	0.6794776298723569
0.2160970630514050	0.5975303020777303	0.6704464553352480
0.0751676231853931	0.5815653580448642	0.5777750380249097
0.6298284176559104	0.5570304080812549	0.8686365853509974
0.7050198859219462	0.5622576788489979	0.8687948963485029
0.3683266119589500	0.5348067819622805	0.8727150878475766

0.2931069120924851	0.5313507244366953	0.8735525014101905
0.4895528787903982	0.6758078400591471	0.8783370867605947
0.4861337036708748	0.7508840936110226	0.8839792449962813
0.5139166810580075	0.4151074919501816	0.8619234837004425
0.5265824266500150	0.3411597861771857	0.8554608856729289
0.5928807060510747	0.5361770699430830	0.5884482353624280
0.6329860360500811	0.4338762789308928	0.5321402475729773
0.5819569725720820	0.3232174917267435	0.5588817104377425
0.4892577437723682	0.3163922962283566	0.6419688282302853
0.4475686958633530	0.4193569454391470	0.6974054305277777
0.4969732499267936	0.7521627822332827	0.6715786567505569
0.4466092867328340	0.7438523888546421	0.5971792034304648
0.5334351379262426	0.7188161126894930	0.5970550425799166
0.1198718720290132	0.6766840080048658	0.5547523401402368
0.1832482741721602	0.6046342348540588	0.7135721189869246
0.2964626313103838	0.5545182679697412	0.7288036262462507
0.2203638162982091	0.6240280100611664	0.5021955874356786
0.3336706239999796	0.5763991825116997	0.5173487940838273
0.1031980585231572	0.6648165864526243	0.6411661464441836
0.4033928367627695	0.5159808055359073	0.5885522680006159
0.4455906133319507	0.5409103616634533	0.7103204206548945
0.0751821829005115	0.5442860186186086	0.6185774500654814
0.0935796184688802	0.5563653174084725	0.5325163713368537
0.9873922725355997	0.6187746581625139	0.6027677584235228
0.7263026937935351	0.5115418082830394	0.8693524253294024
0.7217952615437276	0.5879435396670148	0.9142349182400170
0.7227991233786057	0.5881481386102103	0.8239687905471476
0.2729239657602677	0.5802330840559102	0.8584939877344725
0.2747883122984658	0.5187268024785946	0.9235163492644762
0.2758544645307900	0.4943395584633457	0.8368855749865213
0.4402353102039642	0.7692030733283410	0.8600621045455814
0.5286950265093995	0.7731643592230585	0.8573717884831176
0.4870966288789666	0.7660935906647435	0.9365532370727910
0.5790368513667635	0.3336269586190455	0.8407591572461027
0.4952425425095746	0.3202188026826750	0.8156343978280638
0.5174577529869403	0.3155974104214150	0.9029748833503055
0.3777023679707985	0.5199169502822629	0.6329445653818807
0.5011932930992861	0.5415152133108776	0.9286361098777062
0.4972415470797351	0.5488968001730148	0.8094816401370499

Total energy (E): -436.31925016 eV

Temperature (T): 333.0 K

Zero-point energy E_ZPE : 327.739 kcal/mol 14.212110 eV

Thermal correction to U(T): 358.620 kcal/mol 15.551231 eV

Thermal correction to H(T): 358.620 kcal/mol 15.551231 eV

Thermal correction to G(T): 296.071 kcal/mol 12.838856 eV
Entropy S : 785.899 J/(mol*K) 0.008145 eV/K
Entropy contribution T*S : 261704.303 J/(mol) 2.712375 eV

$Rh_2(OAc)_4$: MS2
1.00000000000000
20.0000000000000000 0.0000000000000000 0.0000000000000000
0.0000000000000000 20.0000000000000000 0.0000000000000000
0.0000000000000000 0.0000000000000000 20.0000000000000000
O C H N Rh
11 25 31 1 2
Direct

0.5170530844086858	0.6023122758148165	0.7122945134997802
0.4868488958258653	0.6321567375123480	0.6080132193066757
0.0239672521121802	0.6224767069528990	0.6003092071475969
0.5974567694705066	0.5518081544379146	0.9454080802282951
0.4064747135816265	0.5576343433316414	0.8096355343321453
0.3954382822502662	0.5363630785841543	0.9212790371630338
0.6091379035337900	0.5752803425280980	0.8341324256758152
0.4977111352166277	0.6668235976843874	0.8416736449388930
0.5073909924484883	0.4444149999924220	0.9149162615173988
0.5188086423113241	0.4662820787753637	0.8033137925406181
0.4872444018006946	0.6433411347339423	0.9530250304131801
0.4530542274666017	0.5224906264339594	0.6440369688179123
0.5034882479751066	0.4689970413943130	0.6241383474815733
0.4985380795227365	0.4050583055174166	0.6520410739491810
0.5406096134499563	0.3538225287887283	0.6304331157421631
0.5881334152639301	0.3661556078388989	0.5806860033545768
0.5938553771006070	0.4300823166400238	0.5531154870887345
0.5516945501726688	0.4814238307615413	0.5748642343863209
0.4886805311347876	0.5888832805746774	0.6592984521362576
0.5176275197909945	0.6968271560740452	0.6197256305579277
0.1445410097053976	0.6503023581686541	0.6105186801743936
0.2141490316508950	0.6215811023201441	0.6065755644882247
0.2414458645884989	0.6020005966173652	0.5448245848605328
0.3039998923247885	0.5721735218861723	0.5406546882830024
0.3423997050189806	0.5604620727948737	0.5986623140528297
0.3167567590395632	0.5820142548013847	0.6605475356312777
0.2533907232611583	0.6113100717131383	0.6636371091859352
0.0906900101711970	0.5962377763096141	0.6016948604176247
0.6320428981837760	0.5654595035070022	0.8932061150527139
0.7068468412736528	0.5690465959829916	0.9018600134952904
0.3720468847720369	0.5448774988116982	0.8624119996349140

0.2973180950098679	0.5404002613376501	0.8535875340668240
0.4902747322483126	0.6834455953738947	0.9031603122573376
0.4846192760882188	0.7571408127982721	0.9180135735232521
0.5171896125947594	0.4270694585160034	0.8541000661284094
0.5288936889066382	0.3537510157293767	0.8408825174498135
0.5562756163971806	0.5314049735405768	0.5533482897006609
0.6311450357741331	0.4399623790139118	0.5145745273240181
0.6205789733413151	0.3258507024630380	0.5634785175171406
0.5363145199878990	0.3040368754558079	0.6522891533576762
0.4615719558423589	0.3959587350230411	0.6910403733483176
0.4999676965466612	0.7179728544366134	0.6671573710145285
0.5022617566184603	0.7278334795523499	0.5773441593463884
0.5722475579664735	0.6915764320795721	0.6214071342995053
0.1369347656136298	0.6886714780536191	0.5717284775452935
0.2341966348743297	0.6270576585368173	0.7123536378792972
0.3454634711447835	0.5757255875179564	0.7064670707454822
0.2130808100353653	0.6104425970688246	0.4988645954521200
0.3237520200900259	0.5572170548731610	0.4919689916939537
0.1374114323809693	0.6748794082080478	0.6593597886652138
0.4201350586127435	0.5210573322207416	0.5453239753438561
0.4301859020229049	0.5075678741049580	0.6918312727647772
0.0963400002010469	0.5578549837416268	0.6411135759305531
0.0972552980583198	0.5709401395827294	0.5532848530375786
0.0151193954207618	0.6438829476740726	0.6430624224002721
0.7272204423616540	0.5179833460729094	0.9008361256512492
0.7193449217061624	0.5911268178062001	0.9504490359353518
0.7294292760221770	0.5976962069224998	0.8610539761212695
0.2777627104302996	0.5901269559099271	0.8406850058528738
0.2735614073724270	0.5229539810299380	0.8996864191395660
0.2852488133166036	0.5067591512455215	0.8119400331408860
0.4392037801781578	0.7772641867351782	0.8945769680100119
0.5275241470935439	0.7835906041489327	0.8962973048807263
0.4826960164295710	0.7655418227681181	0.9720563822780164
0.5806722159268757	0.3466065928122345	0.8239247478885245
0.4959411448689675	0.3362857894117581	0.8006373199454486
0.5203320006093705	0.3245639746486562	0.8863861010041610
0.4024773107555326	0.5261659649915380	0.5925895623286269
0.4963819038778100	0.5435133376573404	0.9355513389005832
0.5083174324462940	0.5673110356519340	0.8191640085756384

Total energy (E): -438.82611984 eV

Temperature (T): 333.0 K

Zero-point energy E_ZPE : 331.335 kcal/mol 14.368057 eV

Thermal correction to U(T): 361.905 kcal/mol 15.693703 eV

Thermal correction to H(T): 361.905 kcal/mol 15.693703 eV

Thermal correction to G(T): 300.773 kcal/mol 13.042735 eV
Entropy S : 768.106 J/(mol*K) 0.007961 eV/K
Entropy contribution T*S : 255779.438 J/(mol) 2.650968 eV

$Rh_2(OAc)_4$: IS3
1.00000000000000

20.0000000000000000	0.0000000000000000	0.0000000000000000
0.0000000000000000	20.0000000000000000	0.0000000000000000
0.0000000000000000	0.0000000000000000	20.0000000000000000

O	C	H	N	Rh
13	34	39	1	2

Direct

0.4371377064587981	0.4222855489627091	0.6929148630110504
0.5490216436216215	0.4125087730826099	0.6713666716242386
0.3555585419850578	0.6045101785575695	0.7069415088106787
0.9624350922686840	0.6929379515948502	0.5779964031612553
0.9559829815528926	0.7862459946724581	0.6430243742813712
0.6394454329351272	0.5436696955414902	0.9063538412804424
0.4207000550802597	0.5243423897060085	0.8263566160267797
0.4391211535845815	0.5419823021739062	0.9376909363949461
0.6229279356966718	0.5406896414700675	0.7933087594316953
0.5115743673100005	0.6358265682858668	0.8026880034362553
0.5404745533115726	0.4418235664165505	0.9295600207045317
0.5301421525658220	0.4311831300807631	0.8162211989908668
0.5364457496381275	0.6450929590204638	0.9137578145232980
0.5078849523666593	0.5176616619866967	0.7053532745576956
0.5108881653158877	0.5614169418938285	0.6482724880621104
0.5245074547258674	0.6308023241623761	0.6549863569167612
0.5285878562695014	0.6719721606508829	0.5992049735866422
0.5187399336523831	0.6458220512403592	0.5350053996629258
0.5044873284833739	0.5776923445966804	0.5268859856906948
0.5006003822139479	0.5361428005525815	0.5823483821744951
0.4932975698551827	0.4466094551037896	0.6895991078765267
0.5400991687586050	0.3418644619881176	0.6590171093247703
0.2399997340997664	0.5708499573836513	0.7346028164123415
0.1668894855904714	0.5824772132888847	0.7200004376076728
0.1250437780550550	0.5318434574636829	0.6952415407361813
0.0571252514207263	0.5426629062704427	0.6845563718262502
0.0278614675750944	0.6052546420542599	0.6984445552249081
0.0698289365423436	0.6570648312725106	0.7212163853491502
0.1377137544378897	0.6450603981705932	0.7319352168766246
0.9503924645103919	0.7259692802368798	0.6359246223326557
0.9835180212111078	0.7346659197484340	0.5227554232630174

0.2881996418030285	0.6017342245294430	0.6833440846350691
0.7119898094913603	0.6542470813147148	0.6837371572248658
0.7418130386818493	0.6870317115008171	0.7377108220705393
0.8111850752779524	0.6948952621330906	0.7400803983879634
0.9270410886063476	0.6779516355491757	0.6918814164499074
0.8514807228827357	0.6703147384327739	0.6883743998772996
0.8211699529059787	0.6383824145089684	0.6337322433896518
0.7518646460050187	0.6300914394169113	0.6316725818028993
0.6599730775215247	0.5411126789184366	0.8459009015822111
0.7343753997047292	0.5365461706893488	0.8352657502872963
0.4013562013921367	0.5311552252215834	0.8873396552655466
0.3274120217938553	0.5261225546702788	0.9004481791428386
0.5223111387141441	0.6693327766096104	0.8564943481723862
0.5174537403244317	0.7443578622570232	0.8505738347429511
0.5356975769754775	0.4078167911653819	0.8758305945216244
0.5369602170126393	0.3326752115368126	0.8812524052367304
0.4894115244134091	0.4832478226284317	0.5752854682634092
0.4965320658056605	0.5571828568915413	0.4769853577573834
0.5220762014408145	0.6784289768926502	0.4913495183436238
0.5392615230962501	0.7250657009495467	0.6057128528634494
0.5307681977062713	0.6511490690777760	0.7049781353570056
0.5185531333654224	0.3174350326826281	0.7032028697014558
0.5902445151090939	0.3225325092796045	0.6485743229620992
0.5069741922029818	0.3336693022587368	0.6159006371955177
0.2500573697996402	0.5167829890475044	0.7382059589814994
0.1688420356890212	0.6858086946810403	0.7508095611317727
0.0498118228274710	0.7067343729190152	0.7314008724951867
0.1460048971255688	0.4825019760255977	0.6847218878490333
0.0256166135043184	0.5020749674954088	0.6659435456636299
0.0303466790655447	0.7603866660346817	0.5352955305096239
0.9908483411140860	0.7005093011965576	0.4806586503814336
0.2525051757336536	0.5928030565038217	0.7835429946735273
0.3686641120972978	0.5605182358702454	0.7236316920740242
0.2738353652944657	0.6539383175621366	0.6734039746778099
0.2848881628417620	0.5744441487593257	0.6352802376365287
0.8344654430550714	0.7200434012877157	0.7825608185321764
0.7110006229537064	0.7057863419109462	0.7786559812589817
0.6580211394229667	0.6468700327681515	0.6824342952235568
0.9449786111416205	0.7720253908537003	0.5112281433341888
0.7287527250796290	0.6048395253928364	0.5891999686068301
0.8519411709131395	0.6206146404914675	0.5922581091834638
0.9393172555197952	0.7044362242344466	0.7385504761645160
0.9353382218391062	0.5772997926476288	0.6650228757503740
0.7499663568659023	0.4846218395592893	0.8446237421781692

0.7603956630351753	0.5685542496671955	0.8714283010548083
0.7478598564073858	0.5503406924902016	0.7840991260432323
0.3047687515090513	0.5760322873800122	0.8957860318212421
0.3189493600780444	0.5080087795327615	0.9514701118217510
0.3036732476921127	0.4930573525693327	0.8638316212338294
0.4681991438866335	0.7581859979379877	0.8306801726261316
0.5552758402911983	0.7628605133849442	0.8152651051610371
0.5251034179264786	0.7675465040117054	0.8995895078623426
0.5805425819608252	0.3133314475528576	0.8539516644567209
0.4919563321555901	0.3118574828343060	0.8575758006894334
0.5395809503676871	0.3173443892869527	0.9337146585419941
0.9590959477957042	0.6131329071140466	0.6918842674016387
0.5393863496075346	0.5436986349640879	0.9231693912461912
0.5207027330762607	0.5327782682868294	0.8032058323407860

Total energy (E): -561.76075629 eV

Temperature (T): 333.0 K

Zero-point energy E_ZPE : 422.336 kcal/mol 18.314220 eV

Thermal correction to U(T): 461.530 kcal/mol 20.013836 eV

Thermal correction to H(T): 461.530 kcal/mol 20.013836 eV

Thermal correction to G(T): 382.774 kcal/mol 16.598654 eV

Entropy S : 989.534 J/(mol*K) 0.010256 eV/K

Entropy contribution T*S : 329514.842 J/(mol) 3.415182 eV

$Rh_2(OAc)_4$: TS3 (imaginary frequency: 215.584452 cm^{-1})

1.00000000000000

20.0000000000000000	0.0000000000000000	0.0000000000000000
0.0000000000000000	20.0000000000000000	0.0000000000000000
0.0000000000000000	0.0000000000000000	20.0000000000000000

O	C	H	N	Rh
13	34	39	1	2

Direct

0.4002287233763014	0.4289790373943572	0.6933319653549299
0.5106709558760032	0.4289272164213126	0.6627254309509775
0.3628150111808740	0.6081462927386146	0.6971329534914215
0.9701514939567726	0.6924195287799002	0.5646828092689804
0.9628725293081395	0.7829227338396927	0.6334891682954982
0.6420457309235418	0.5411911047879788	0.9018889907845720
0.4206776193440533	0.5235174884921993	0.8345781745385593
0.4431801221485794	0.5370534137890005	0.9455353769250165
0.6200147440468432	0.5326638918863131	0.7901659932905030
0.5155401668379195	0.6310015863160769	0.8046209435187002
0.5462670349931662	0.4381113414237873	0.9318125451609952
0.5256936974692649	0.4271784247139748	0.8200052034405503

0.5382062138896155	0.6411530503693339	0.9159524092555699
0.4620897592192858	0.5320250028849893	0.6870917083321108
0.4883572034122500	0.5701546656294574	0.6323844801729459
0.5091563487532986	0.6373232529103070	0.6419893906401651
0.5276612604274450	0.6764930626508951	0.5877480025765794
0.5234852324049231	0.6510302223332036	0.5225374426465382
0.5028376584725436	0.5849274546699630	0.5120699642576754
0.4869475545288349	0.5444172189514045	0.5662024452578649
0.4522980529093599	0.4589566514346546	0.6817464551234387
0.5107535653458591	0.3568672296025509	0.6616133132711781
0.2507292691858745	0.5667604607152303	0.7299648153825049
0.1784012963264714	0.5781777965513750	0.7117328722742129
0.1374375289876582	0.5272601834591308	0.6858684565279939
0.0700584565537739	0.5379544917952775	0.6719818414111768
0.0401310505160985	0.6008157475760764	0.6835217171295739
0.0815482879279022	0.6532253271464991	0.7059852016466605
0.1489442362401371	0.6413480436561415	0.7198382927710713
0.9585983187637085	0.7228425486079235	0.6240888050465413
0.9896102606033188	0.7367668671573570	0.5109018350260440
0.3005078464984315	0.5901875822231838	0.6754186442763255
0.7221379720767567	0.6494105942993732	0.6762521660235306
0.7537575153611712	0.6824922460561509	0.7290444578231394
0.8232742805346013	0.6894762176861386	0.7296479127682156
0.9378729780379285	0.6720883286263394	0.6786893390768071
0.8621833129930203	0.6638346811446535	0.6773328354648734
0.8300622729381306	0.6315987380703906	0.6239034263117462
0.7605893762030480	0.6240555729391800	0.6236595370235570
0.6597908764941034	0.5362306342337206	0.8404413851949878
0.7336861824293561	0.5329554398770630	0.8267112361687806
0.4032277878593444	0.5293948230738215	0.8958830246130579
0.3293940147605581	0.5282720152840220	0.9103555616355646
0.5251885197046849	0.6651461463057849	0.8580529705680748
0.5205477923311910	0.7399818066837196	0.8510901705881664
0.5370191522310693	0.4036317838114495	0.8785985429619695
0.5400366873479663	0.3286581321441388	0.8851090649905413
0.4700161326788430	0.4933025508813719	0.5576888963970442
0.4984102791469643	0.5653613847423083	0.4613421506586787
0.5357606373639208	0.6827991266748131	0.4799293044120790
0.5444348243590139	0.7277658489042108	0.5958049884802880
0.5103796933095266	0.6570058638240798	0.6927656055459617
0.4883549061375778	0.3369639482934119	0.7075649632002259
0.5634954743448145	0.3426189207429899	0.6579591374788195
0.4828757253536722	0.3383225517125080	0.6180321566040183
0.2601182100961463	0.5136943232871179	0.7410959295999832

0.1793889117483651	0.6827247062773503	0.7384993412509059
0.0614422092242101	0.7033005053828222	0.7137649086259030
0.1586130678228033	0.4775959022880146	0.6775049580998997
0.0393520158707419	0.4967818769806172	0.6533041064354320
0.0363724468609532	0.7625004304821168	0.5236877340303707
0.9965396049717848	0.7045538175377265	0.4672327194868282
0.2629773199257238	0.5951252123037876	0.7755217451506737
0.4348708555692383	0.5584139227327622	0.7269830702590074
0.2790054389308048	0.6344021138108759	0.6485376745804760
0.3039316881405077	0.5516468231310713	0.6352341168118987
0.8477494551546211	0.7150210730798107	0.7712121067060934
0.7242280302342321	0.7023403795337202	0.7704227307358152
0.6679743330876952	0.6428674606129141	0.6762788522212290
0.9504250071498214	0.7741126594481617	0.5017689171652578
0.7362126793261429	0.5985247482458226	0.5820709959974291
0.8594156670137104	0.6130133058622842	0.5817600563587310
0.9512033480007607	0.6969905458963419	0.7259516344530064
0.9478845489560732	0.5720011492266264	0.6489328977066831
0.7512390106148708	0.4820192513152730	0.8377502556154603
0.7605238480509995	0.5673532196104791	0.8599076237853266
0.7443340705320638	0.5446782208037556	0.7743550823812003
0.3094543136517992	0.5791385933620585	0.9040254575216908
0.3201073935492705	0.5121531990692618	0.9618596246008048
0.3036870090390148	0.4957226647872613	0.8745669298445412
0.4713925619120959	0.7531992333677772	0.8304736716809833
0.5584956996837044	0.7574074644268077	0.8153834607958930
0.5279149651131456	0.7644703596082186	0.8994531130968342
0.5832180307738662	0.3096899610002750	0.8569243486884036
0.4948806514811313	0.3063770190839041	0.8632731588623729
0.5447323347805157	0.3141732592225343	0.9376732940089031
0.9715060755979232	0.6082429575366697	0.6752891771670834
0.5430049444177117	0.5396454137749884	0.9253887477476739
0.5197449687730188	0.5287409851870589	0.8090470967476825

Total energy (E): -559.95095927 eV

Temperature (T): 333.0 K

Zero-point energy E_ZPE : 418.714 kcal/mol 18.157153 eV

Thermal correction to U(T): 456.210 kcal/mol 19.783141 eV

Thermal correction to H(T): 456.210 kcal/mol 19.783141 eV

Thermal correction to G(T): 380.983 kcal/mol 16.521009 eV

Entropy S : 945.189 J/(mol*K) 0.009796 eV/K

Entropy contribution T*S : 314747.793 J/(mol) 3.262132 eV

$Rh_2(OAc)_4$: MS3

```
1.00000000000000
  20.0000000000000000   0.0000000000000000   0.0000000000000000
   0.0000000000000000  20.0000000000000000   0.0000000000000000
   0.0000000000000000   0.0000000000000000  20.0000000000000000
 O    C    H    N    Rh
 13   34   39   1    2
Direct
 0.3769899489020516  0.4364603485190555  0.6576686726644372
 0.4903002464356274  0.4412282618685656  0.6690515074449733
 0.3761771364579616  0.5731394893949963  0.7044578652957186
 0.9772696469908878  0.6918063981153201  0.5530495046472771
 0.9679670205607077  0.7797027346617293  0.6248747293557706
 0.6452918435223570  0.5383738708985645  0.9048400976124413
 0.4221244425217541  0.5199938037487430  0.8415809927881018
 0.4467610136679225  0.5356083534806476  0.9518762480437565
 0.6202317883971251  0.5275510426699525  0.7939320258349387
 0.5160901993051968  0.6259535381441892  0.8080342676134105
 0.5503811399021410  0.4353625705471160  0.9382714917940703
 0.5259958147597711  0.4228360025960383  0.8274297115007834
 0.5411580533835035  0.6381673301339953  0.9186868591673001
 0.4339266336933287  0.5443618436435129  0.6758940200415184
 0.4618484710217438  0.5811745669044398  0.6156249665828561
 0.4964613861092651  0.6410399291414345  0.6274748069279995
 0.5243740783835801  0.6767115592435941  0.5744766807384900
 0.5181405916186795  0.6528003186024650  0.5090420444605552
 0.4835146038635413  0.5933613453815934  0.4969221899562145
 0.4552504875970593  0.5577132817686398  0.5500307426719699
 0.4282165102972699  0.4685216280815607  0.6657855640705685
 0.4931299213015256  0.3691866577073980  0.6643976806797497
 0.2596681934102560  0.5661947463694669  0.7192489859329776
 0.1887155993368589  0.5779125576330869  0.6964987407783562
 0.1487705242450460  0.5255897115997274  0.6718682839745331
 0.0813493479182035  0.5349627696569066  0.6575870325366258
 0.0504837439355677  0.5976054241879557  0.6677796641419921
 0.0910183298216142  0.6512604546614066  0.6887724815262430
 0.1585396080782934  0.6407505852696794  0.7031302172850796
 0.9655339297852188  0.7198654509048884  0.6134882648274272
 0.9954921209240615  0.7385473069913119  0.5008688831287833
 0.3146111445850684  0.5763522855733882  0.6668500571647908
 0.7317233479358491  0.6459761881945202  0.6701331598944164
 0.7650504414796225  0.6791977544055072  0.7217863050115728
 0.8346053577247238  0.6851432743956498  0.7206118840619117
 0.9473821519792275  0.6667980127001238  0.6667982319521164
 0.8718005214662540  0.6583228185325761  0.6676693535096704
```

0.8380485584565722	0.6258598234879347	0.6154375492713321
0.7684943856914943	0.6193964932474146	0.6169259871926345
0.6613103572019297	0.5328212582375590	0.8429441094365417
0.7348215051417978	0.5309895212581164	0.8269278234551585
0.4058602187750192	0.5279189194181715	0.9029891756500459
0.3322084973550925	0.5298412907229808	0.9184267951354056
0.5269203746310495	0.6610931470988003	0.8605715461602050
0.5224030048722029	0.7358587524510299	0.8526158952437356
0.5395499392767590	0.4000881003588380	0.8858802547942375
0.5435537544540134	0.3252061478494647	0.8928137482778880
0.4289142690028246	0.5108448227063128	0.5404992599037030
0.4786723959689165	0.5745532328092761	0.4459563859523564
0.5406098193460184	0.6803424833160140	0.4676408119200149
0.5513916826262796	0.7231085654891173	0.5840945910662096
0.5017166222032938	0.6587127230916331	0.6789162823229264
0.4658757653582076	0.3468968656895757	0.7064868602955222
0.5462770591535390	0.3564640317098261	0.6669654526720362
0.4709602832763908	0.3520606523877861	0.6171656850003377
0.2653256334726245	0.5150498849325879	0.7388153062957585
0.1881761874499856	0.6828419819006520	0.7215308575213824
0.0702343160062227	0.7011723702350017	0.6954560169570848
0.1706180339566652	0.4758661126462149	0.6655610438501535
0.0510813525871098	0.4928682544265676	0.6404168752589595
0.0425285654415004	0.7636754655269543	0.5138631408864777
0.0015591586063987	0.7083564612461413	0.4556742068576279
0.2710889296834505	0.6002305232783020	0.7610328206259236
0.4717012849187071	0.5504907996616180	0.7162532603938451
0.3106163269722964	0.6258274819321749	0.6427031192503962
0.3122587629220560	0.5371792342898172	0.6283461498103377
0.8605434351973806	0.7110033974764689	0.7610602563059444
0.7368318416370472	0.7002642170763371	0.7634339216153626
0.6774301528094177	0.6404710082201663	0.6714297092725319
0.9561518528931614	0.7762392143385328	0.4942666270774233
0.7427327175084724	0.5939214215527275	0.5761601421327909
0.8661069326989317	0.6063645379983768	0.5728239941123024
0.9620274839698921	0.6901381539059936	0.7145216654924407
0.9582065438027763	0.5673644170334676	0.6340897019032536
0.7540011848785840	0.4807169123797448	0.8382564861485736
0.7619104819783175	0.5666521111790710	0.8585379875691229
0.7435010121751267	0.5419577646890211	0.7740342627866061
0.3143160280810999	0.5814391370516327	0.9118504782670285
0.3228278523141060	0.5146642397405629	0.9701891998400286
0.3047399534902334	0.4980562511465581	0.8832650839089634
0.4732844211865806	0.7490334889358092	0.8319087715208235

0.5603754754697086 0.7527213527383726 0.8166683922314835
0.5299304991070228 0.7609365580220707 0.9006598101241792
0.5863247962247889 0.3067031624313854 0.8637064608958644
0.4982580186253887 0.3022760487341866 0.8719760380226086
0.5495930882638761 0.3109836277087842 0.9452881408888753
0.9817467240524824 0.6037861548935227 0.6601856161009484
0.5464770368548019 0.5370305512970975 0.9302854887677271
0.5204499636535218 0.5238866107144960 0.8155264069374731
Total energy (E): -562.27636613 eV
Temperature (T): 333.0 K
Zero-point energy E_ZPE : 422.895 kcal/mol 18.338483 eV
Thermal correction to U(T): 459.407 kcal/mol 19.921791 eV
Thermal correction to H(T): 459.407 kcal/mol 19.921791 eV
Thermal correction to G(T): 386.739 kcal/mol 16.770611 eV
Entropy S : 913.041 J/(mol*K) 0.009463 eV/K
Entropy contribution T*S : 304042.563 J/(mol) 3.151180 eV

RhN4 SA (pyrrolic N): IS0
1.00000000000000
19.7297992706000009 0.0000000000000000 0.0000000000000000
-9.8648996353000005 17.0865073798999987 0.0000000000000000
0.0000000000000000 0.0000000000000000 15.0000000000000000
C N Rh H O
131 6 1 8 2
Direct
0.0011600917477840 0.0016563926559046 0.0771317166963525
0.0416230606776171 0.0843756025946872 0.0819086558332285
0.1259144279281862 0.0000711171614141 0.0818682072155665
0.1663308109968279 0.0827915031418160 0.0855033973183365
0.2507979210468896 -0.0006567163684687 0.0963320885574069
0.2917172536609342 0.0824620784499439 0.0997377347021051
0.3757141393015106 -0.0004926765049656 0.1122305235391890
0.4166907883301273 0.0830217508368185 0.1159566078604637
0.5007946000965647 0.0004243538067802 0.1204725321663591
0.5415791229262276 0.0843598309568961 0.1224197877225804
0.6258283612620004 0.0000223342840093 0.1180259914833281
0.6665776860691288 0.0839856385606928 0.1179086639569507
0.7505125938434003 0.0012358201560486 0.1053995314425384
0.7915120515999664 0.0848620870777585 0.1054991454577746
0.8754363366291623 0.0018569665797901 0.0869398306651527
0.9164862983797137 0.0850437473340223 0.0903864993145991
-0.0003381335549724 0.1263364929571590 0.0880691357536447
0.0409054639535869 0.2092758602724588 0.0963583236178724

0.1243308942434710	0.1247694410251170	0.0854733338595101
0.1647483967424150	0.2073489163478022	0.0941806968248553
0.2489191874865271	0.1232221043473874	0.0943566834809774
0.2887945541490672	0.2053462127406757	0.1015287943264059
0.3745928206855682	0.1241544641496607	0.1113002992471562
0.4150002604300185	0.2070715745431746	0.1191576481927775
0.4995973386197590	0.1264100216814430	0.1231152047089171
0.5401984021272550	0.2108264470358341	0.1294313317983901
0.6248048071651480	0.1259697590251232	0.1224306073445209
0.6656878402880111	0.2097444328259210	0.1255902595434484
0.7497716795670567	0.1265009515830733	0.1132451861409755
0.7907987180387700	0.2101448009359482	0.1162302172509116
0.8746382958404147	0.1265979918072404	0.0997120187581124
0.9159769957092394	0.2099032410544295	0.1050800118995069
0.9993638991399136	0.2511905411897137	0.1041116888625982
0.0411248536952708	0.3341989747368370	0.1121646243501030
0.1240159453668013	0.2501804283446766	0.0994851729448383
0.1657370629470825	0.3330725137643162	0.1107336918571306
0.2468438442431117	0.2471928009001939	0.1013884996423962
0.2866089504036813	0.3290117100563424	0.1135741313454066
0.3706209768820221	0.2451743527029913	0.1139440475349286
0.4082097870031896	0.3256428030851384	0.1265366791093981
0.4977698522423126	0.2531699397057267	0.1317736042547151
0.5376873759449532	0.3386853096459252	0.1450807356897723
0.6234988005207643	0.2514262636422993	0.1310985888124591
0.6640350602825191	0.3348210019587486	0.1372028781592004
0.7489570419104513	0.2519128254202777	0.1233315804972844
0.7901908229750876	0.3359158774785169	0.1269354674152367
0.8742698841709912	0.2516519242328365	0.1131289974616622
0.9158740682054293	0.3350718812897701	0.1178507329200788
0.9995284805591257	0.3760461771481797	0.1172105553682286
0.0421372692155875	0.4593088005091049	0.1204052422462367
0.1246773087119096	0.3751822741397197	0.1154408865741460
0.1681620087175456	0.4581488468592154	0.1223270285493277
0.2486934492546663	0.3735066099864957	0.1184086590198597
0.2948994083085031	0.4563130860800618	0.1307906425589685
0.3670036907652444	0.3666272964878660	0.1262983014242092
0.4166820768969192	0.4450580547643848	0.1479354302166531
0.4866789096834824	0.3756706811285302	0.1481114250102279
0.6212080943598578	0.3750284660216447	0.1475145498573787
0.6686016286417821	0.4588870176950021	0.1640988484948360
0.7485795861071910	0.3777387410303834	0.1341096516042067
0.7915524625743504	0.4623078947379498	0.1339887156802819
0.8741774342651377	0.3770618656676727	0.1233086811617628

0.9163942347491210	0.4603662990599575	0.1236658516670039
0.0001631189721612	0.5011728354251827	0.1210582876051385
0.0417333407674527	0.5843530886765230	0.1178711600303118
0.1260890531871352	0.5000985931737076	0.1217649387738140
0.1677041966201490	0.5832867118667530	0.1214985030369881
0.2525639245528042	0.4987341410924233	0.1283024000005103
0.2932665430116597	0.5820381279324770	0.1296526996143386
0.3803330016561561	0.4961002562393237	0.1448819753994620
0.4170600684588665	0.5799379005082210	0.1476650680012624
0.7515671229395460	0.5054984237850355	0.1422684048773643
0.7875517211182321	0.5887167316179076	0.1336983433790327
0.8747797382189620	0.5026702349719346	0.1261518358735778
0.9152156856570781	0.5857935268884417	0.1200496164516420
0.9996008511198606	0.6262310672609047	0.1142270039160106
0.0428531355901123	0.7089808656665021	0.1052132372975727
0.1256822601258431	0.6250683407998671	0.1172482929299885
0.1681312223501411	0.7082423746104659	0.1124483711406473
0.2514901463609245	0.6241546020092835	0.1242056498363922
0.2935550508331928	0.7074315012400503	0.1219611206036170
0.3766607150083487	0.6225924039646021	0.1360096571018128
0.4192948824666054	0.7069724854623609	0.1328965886844750
0.5001225629381917	0.6274134594250377	0.1674254729363651
0.5467393912299607	0.7094754874289847	0.1425459485852110
0.6812815871577745	0.7094402829242583	0.1326299110598280
0.7505167570548615	0.6396985996800898	0.1328879112513492
0.7993666297767366	0.7167357290152057	0.1046174371805664
0.8727022970086843	0.6281013209040071	0.1182520482926816
0.9184228576931276	0.7103647325886280	0.1015672534802033
0.0015857139183584	0.7508321833333168	0.0966406758654654
0.0433819194296255	0.8338827431198483	0.0868130853966567
0.1264465526171737	0.7499625621031307	0.1049976219310217
0.1681691002253012	0.8331024956085445	0.0992454545180933
0.2517731374930987	0.7492764863084822	0.1151467839391384
0.2932192434223903	0.8326875786020924	0.1122767833449012
0.3774605663520693	0.7486136386825745	0.1255811724747656
0.4185556588019123	0.8325191651708341	0.1222352095486413
0.5036855936230978	0.7496819005934462	0.1330251403682414
0.5440835779947718	0.8329436971564003	0.1252297303205343
0.6302707620743832	0.7458513654528246	0.1335766177897770
0.6695473307935780	0.8309936626401898	0.1176856928357682
0.7583123571238296	0.7579999302321303	0.1044209460146776
0.7959244281931805	0.8385638297988764	0.0918992236169744
0.8799742196754631	0.7543079664639599	0.0920468042110826
0.9199448571245036	0.8363817361300380	0.0804125841871501

0.0024880341636479	0.8765954123121160	0.0791409224905684
0.0431840749689286	0.9596037438612313	0.0770988885200451
0.1265835311526325	0.8749470871361713	0.0901148225841567
0.1678764995820680	0.9581147112568338	0.0879184328423321
0.2514499285501927	0.8744181324125488	0.1045503751113819
0.2927128416681100	0.9577769666770244	0.1039424962372664
0.3765710969121041	0.8742364835097786	0.1170480675917548
0.4175505477443792	0.9578790301963099	0.1169696293385072
0.5018601691313782	0.8746539394881451	0.1228453685183297
0.5426783574546908	0.9584340763539575	0.1208522395553381
0.6272434958998986	0.8735043063331854	0.1195590161006764
0.6676945833025729	0.9578873636790463	0.1142173586193669
0.7518814849237504	0.8768390784218374	0.1012902691422537
0.7923814057748108	0.9600541585557528	0.0966529160967472
0.8779805068949101	0.8784668461592764	0.0804265562320575
0.9181730850556667	0.9609946002847777	0.0791717194935839
0.5875519129610023	0.5520039884449719	0.4329769097518227
0.6388956309593025	0.5175232924660705	0.4367257258699052
0.6082662999942762	0.4371544955638536	0.4195819504126415
0.6558882828878474	0.4037148125512237	0.4237785463922049
0.7352749623184385	0.4496081049205948	0.4446405577197015
0.7661445958597305	0.5293144601229828	0.4617423223440898
0.7188079644447481	0.5631896041105027	0.4583286301005982
0.6054292436798686	0.6334314458513904	0.4192844249099066
0.7016144688076024	0.7611858456380679	0.3769944818489918
0.5322828168434491	0.5979250984727995	0.2162646890184909
0.6780930159972579	0.6368369940517055	0.1544362824307519
0.6372446263779447	0.4926177495034295	0.2047503778271983
0.5116805848021443	0.5036853586297353	0.4423332416995398
0.4460310647775432	0.4610017908203856	0.4490867634560083
0.4906331245657518	0.4494250992889144	0.1661172747268294
0.5853372924889940	0.5455194671330192	0.2078416232852306
0.7438160074167794	0.6252701914982802	0.4721205730449840
0.8279165997651787	0.5658842521750043	0.4786364561667168
0.7726095475219095	0.4235188942759353	0.4478066674566417
0.6307618478451649	0.3413998876865006	0.4100016479539602
0.5467778374113771	0.4003250989206283	0.4015660335865421
0.6655861733673010	0.7624899143966187	0.3220076823025607
0.7634336629752977	0.7910013133691925	0.3579136023020659
0.6925965985674631	0.7885740022892846	0.4360824846027929
0.5571488588768733	0.6549293653574676	0.4259368877698966
0.6816198834314485	0.6814491457379102	0.3960977372637514

Total energy (E): -1299.68279194 eV
Temperature (T): 333.0 K

Zero-point energy E_ZPE : 96.850 kcal/mol 4.199836 eV
Thermal correction to U(T): 106.213 kcal/mol 4.605828 eV
Thermal correction to H(T): 106.213 kcal/mol 4.605828 eV
Thermal correction to G(T): 87.171 kcal/mol 3.780084 eV
Entropy S : 239.256 J/(mol*K) 0.002480 eV/K
Entropy contribution T*S : 79672.174 J/(mol) 0.825744 eV

RhN4 SA (pyrrolic N): TS0 (imaginary frequency: 444.356152 cm^{-1})
1.00000000000000
19.7297992706000009 0.0000000000000000 0.0000000000000000
-9.8648996353000005 17.0865073798999987 0.0000000000000000
0.0000000000000000 0.0000000000000000 15.0000000000000000
C N Rh H O
131 6 1 8 2
Direct
0.0018005014544313 0.0033101984626716 0.0789906872005517
0.0423673866927492 0.0860972903958062 0.0831711009318130
0.1266051971420802 0.0018513472298674 0.0829996952266590
0.1671515173363659 0.0846446224499900 0.0866448053885831
0.2515298705821016 0.0010653706040429 0.0964674899585427
0.2924970454491415 0.0842764617925804 0.1002937045050987
0.3763341787453147 0.0010748557978364 0.1120444275465655
0.4173277297270265 0.0846910528753238 0.1164024638691503
0.5013403580142660 0.0018874384509759 0.1205472607087029
0.5420864199751162 0.0858869295988275 0.1233627909774999
0.6262676698153412 0.0013217366900334 0.1181311384392226
0.6670199692590134 0.0853628952641188 0.1189772012862629
0.7509366728952390 0.0025278259093748 0.1054088387886101
0.7919782277184788 0.0862419710270470 0.1062324511687247
0.8759744224120539 0.0033396879360942 0.0880595432263283
0.9170550703535326 0.0866079394077984 0.0912353147880787
1.0003102457175292 0.1280111213524058 0.0888938356743888
0.0415950663977374 0.2109993076990815 0.0968458725729085
0.1251614154311634 0.1266214237526341 0.0866691522426672
0.1656682323845816 0.2092908829495543 0.0954548534338525
0.2498336343263557 0.1251609806225480 0.0954789210384510
0.2898050521492356 0.2073729111661275 0.1037219512229906
0.3753533333785375 0.1260104584133974 0.1121881535749070
0.4157350847629921 0.2089478144460794 0.1211968696147500
0.5001700232583766 0.1280617400095370 0.1243161767717304
0.5407195313329598 0.2125966557743295 0.1316261213400945
0.6252919114093168 0.1274970636801003 0.1239819179671479
0.6661230670878787 0.2113796323301089 0.1279087885159740

0.7502385123566161	0.1278931005831971	0.1145626138134934
0.7912189900948842	0.2115405728080198	0.1179400419343509
0.8751281172040045	0.1280792882512140	0.1003653737658170
0.9164717174727431	0.2114196153445955	0.1056868835066447
0.9999070280710635	0.2527815542354986	0.1044633406613166
0.0416537886580303	0.3357975898023458	0.1124617716538168
0.1248005746111092	0.2519607711937690	0.1003542074819935
0.1665465936868699	0.3348175916395274	0.1121119791487483
0.2478562041496246	0.2492625757045090	0.1037030353167469
0.2877393770881978	0.3310345103230820	0.1170712653448926
0.3716156391893711	0.2473301350759974	0.1169553901180608
0.4092891827751112	0.3278840248238688	0.1305799661268439
0.4984388899756177	0.2550844294467827	0.1342189735927353
0.5384977758057552	0.3409298572617744	0.1476043537752341
0.6240243212145368	0.2533332368022190	0.1337307976501644
0.6644499617457101	0.3368972208364172	0.1398324643539238
0.7493687981105346	0.2533575211736401	0.1255304059237989
0.7905173685616317	0.3373950716716705	0.1286737127914792
0.8746929568877506	0.2531127330813619	0.1141642958224658
0.9162777927729839	0.3365629147522884	0.1184674300936441
0.9999527525878830	0.3775818030013469	0.1173721443378724
0.0425539558738954	0.4608362641046224	0.1205344977636516
0.1252832687352797	0.3767989705003755	0.1163455932146286
0.1687127779950510	0.4596734020390323	0.1238355935678120
0.2494770347609205	0.3752032214044196	0.1211309591574724
0.2956657962366077	0.4579259664056227	0.1340657371703175
0.3682324087837546	0.3687437764126819	0.1308955187686194
0.4190035917624760	0.4476430968234276	0.1514592101079469
0.4883118282706666	0.3789605103163329	0.1505972607131737
0.6217211823858395	0.3774600547805463	0.1502363791442674
0.6693171601588247	0.4619883462501872	0.1664439563561843
0.7489361816565311	0.3793126893212161	0.1357308892143103
0.7915775174361371	0.4637286196046168	0.1326808863962090
0.8745045114147100	0.3785264985403169	0.1239286249792423
0.9166335624763298	0.4618057338000257	0.1230406634637380
0.0004691728344884	0.5026380151919133	0.1205832056234523
0.0420054417706549	0.5857778461155042	0.1173663725320244
0.1265487116982380	0.5015905770812786	0.1227304848745829
0.1681396543463563	0.5847614331976603	0.1227460023802186
0.2531959549917863	0.5001997450646180	0.1309677682491436
0.2939402604474662	0.5834911377181843	0.1325781587197960
0.3813524207125836	0.4978564230358725	0.1482546794244261
0.4180151392562420	0.5812841767296432	0.1513144314489269
0.7512365861142356	0.5068477934725154	0.1376858108781546

0.7866773906392164	0.5892425761073439	0.1243213614759230
0.8747948480391767	0.5040079629452433	0.1241125737575939
0.9152151685282448	0.5870844457259714	0.1168751771464857
0.9997662046788495	0.6275844817448344	0.1127552037928867
0.0431300953191941	0.7104015358813851	0.1042807010956239
0.1260112702714531	0.6265024639272012	0.1177245044115690
0.1684937348660253	0.7096971776399024	0.1130283774362048
0.2520049506806269	0.6255682193259208	0.1263578777783174
0.2939445711904128	0.7087960547286998	0.1238218502437779
0.3774211399568655	0.6239129770697533	0.1392774168329251
0.4197765614231641	0.7082600800236185	0.1349780961904501
0.5019831551029238	0.6287111254895730	0.1692631419004723
0.5471091704215523	0.7101081301245966	0.1394078539015410
0.6792492102755894	0.7077984877098566	0.1201615630038464
0.7480099843035216	0.6385683183564423	0.1194015169383593
0.7988976857967226	0.7173203559838811	0.0990207547254387
0.8724308801924796	0.6290615594113417	0.1125686110648775
0.9185365578772744	0.7116121602094626	0.0984963135466604
0.0018418808503035	0.7522552838439902	0.0956762193056898
0.0438551165063405	0.8354512785179055	0.0873561772807136
0.1268270820402228	0.7514358961213875	0.1049157003537758
0.1686519399716484	0.8346002591235997	0.0992298617249329
0.2521323511920721	0.7506674005952875	0.1162355331411760
0.2936776872920921	0.8341044636398340	0.1127130502477012
0.3778973680024605	0.7499114092003514	0.1272957489827289
0.4190146270064206	0.8338320968418029	0.1227695150086261
0.5041224948511471	0.7506908240525378	0.1326903004935188
0.5444298186626125	0.8339527874879521	0.1240357946051762
0.6298529376265465	0.7459404049315940	0.1257543163389318
0.6695450957420437	0.8317077050591878	0.1136247481183322
0.7578848697319034	0.7584950424302861	0.0993759583273898
0.7961706371013212	0.8396908058726131	0.0911006478650037
0.8800815696957999	0.7555639115820669	0.0904401179346215
0.9204341086114834	0.8379082298565959	0.0817802072341852
0.0030265523129086	0.8782272831874351	0.0807469366226158
0.0438031127909560	0.9612830027534561	0.0788753436906610
0.1271319116087573	0.8765301821732917	0.0904683102332298
0.1685316133070427	0.9597917085450741	0.0884578365511393
0.2519659767645824	0.8759261213782082	0.1045429652829360
0.2933170385068146	0.9593502441145019	0.1037788427525890
0.3770875518746442	0.8756545671469332	0.1172717181612489
0.4181086718736883	0.9593378830182251	0.1167747051014145
0.5022957392807224	0.8758857295667579	0.1225266002356065
0.5431326301126467	0.9597536809522562	0.1206502438370796

0.6275551173723788	0.8745053074678417	0.1175738013223597
0.6680613311873536	0.9590610211119091	0.1136622865506423
0.7521534082192273	0.8779662700147515	0.0994738239768916
0.7928013941462079	0.9613193739282334	0.0966335648048154
0.8784631021743303	0.8799873309068608	0.0820465670427648
0.9187611392847161	0.9625491352040884	0.0810757409988999
0.5882285096311807	0.5437079805706279	0.3646766594860529
0.6279572767987025	0.5040762017504427	0.4051619709396030
0.6031781798081044	0.4258890690570060	0.3816764568859307
0.6468722486695803	0.3912159357476226	0.4059177407713336
0.7163022456224140	0.4338587834692364	0.4543632892326991
0.7395589550449474	0.5100868595353281	0.4816899521997351
0.6953354978515124	0.5447681045186656	0.4585020858206096
0.6102971182110729	0.6260133974485573	0.3941654368171927
0.7149356050992736	0.7588363583112020	0.3819219733908452
0.5365130909699118	0.6028385256657923	0.2195075059020547
0.6725413563633853	0.6319055435273935	0.1322488421061869
0.6436888750751402	0.4976571869377032	0.2137034807900790
0.4921450553704969	0.4928755325818158	0.4007867957498620
0.4302256681562882	0.4476657893371290	0.3823168594961759
0.4939983951096925	0.4534389736100353	0.1658005634205888
0.5943676038650243	0.5543364179805664	0.2265832133006891
0.7152248123625671	0.6047430235126237	0.4789424705062141
0.7929246548805208	0.5432884206126621	0.5206846130139889
0.7521211707616116	0.4077108095183675	0.4706370751592179
0.6279079610890309	0.3314682535647095	0.3847637841406487
0.5504702656015015	0.3933012141746817	0.3414775993884695
0.7009134343642659	0.7787478576644346	0.3197967795665789
0.7784759403511471	0.7868839318068482	0.3900670808527790
0.6879854564547436	0.7713439162731675	0.4387554209272940
0.5658619079715957	0.6460299716484350	0.4249320342279351
0.6872899866668583	0.6757235156047776	0.3750017292618969

Total energy (E): -1298.77652457 eV
Temperature (T): 333.0 K

Zero-point energy E_ZPE :	94.902 kcal/mol	4.115350 eV
Thermal correction to U(T):	104.874 kcal/mol	4.547779 eV
Thermal correction to H(T):	104.874 kcal/mol	4.547779 eV
Thermal correction to G(T):	84.226 kcal/mol	3.652372 eV
Entropy S :	259.440 J/(mol*K)	0.002689 eV/K
Entropy contribution T*S :	86393.637 J/(mol)	0.895407 eV

RhN4 SA (pyrrolic N): MS0
1.0000000000000

19.7297992706000009	0.0000000000000000	0.0000000000000000
-9.8648996353000005	17.0865073798999987	0.0000000000000000
0.0000000000000000	0.0000000000000000	15.0000000000000000

C N Rh H O

131 6 1 8 2

Direct

0.0018824586017241	0.9993580370355674	0.0887630955330452
0.0423983238021865	0.0821233997073696	0.0897192393307098
0.1266127462687338	0.9979558251932255	0.0903570089191919
0.1671069052791264	0.0807030218155184	0.0896320620496991
0.2516742146106999	0.9973243904559376	0.0998066820404012
0.2926276672869846	0.0804947981189917	0.0986051146617018
0.3766551328166963	0.9975193381404884	0.1124761521491222
0.4176582478436988	0.0811978320434161	0.1123184192169706
0.5016830305693265	0.9983003254646127	0.1206018533073875
0.5424247975031936	0.0823632711144421	0.1201817472810964
0.6265730724404578	0.9976686287355392	0.1204477243597147
0.6673129611767009	0.0817004086145642	0.1191558572020033
0.7512054430526451	0.9988003442295852	0.1111853167404928
0.7922815930593787	0.0824941251004398	0.1103770155436655
0.8762261978870535	0.9994702623376975	0.0969209210588082
0.9172658865732147	0.0826936785195210	0.0980114102688431
0.0004473511178598	0.1240741989985279	0.0942210046328211
0.0418144637436690	0.2072646965118274	0.0981237895046160
0.1251615934214790	0.1226603386330339	0.0893020374691064
0.1657948570392306	0.2055368526743935	0.0925357997072648
0.2499349238526259	0.1213170621576614	0.0931354957228098
0.2900792610894111	0.2037503931298206	0.0950002348468758
0.3757408141207514	0.1224321455093525	0.1059952073277454
0.4162939967488202	0.2056940247718523	0.1088368669949674
0.5006362795224213	0.1246912047543882	0.1181052830120870
0.5411065083216100	0.2092642736707990	0.1211630453095900
0.6255632286207465	0.1238897386017364	0.1208371546448146
0.6663748246036969	0.2077648773600931	0.1222146633683336
0.7505279611884510	0.1242288060196199	0.1157126187239348
0.7915538538935361	0.2079251758409528	0.1169916849055290
0.8754300725727329	0.1243284805461961	0.1048965740961834
0.9167981641320959	0.2077193090037090	0.1075359968368694
0.0001711921480005	0.2491153821288614	0.1048022576636669
0.0419781915201231	0.3322811694645099	0.1100974814271007
0.1250035467239406	0.2482844985977501	0.0974012015403688
0.1670030288247343	0.3314819366715142	0.1049395360546755
0.2482133590367111	0.2457022001645046	0.0947970389672555
0.2886008337598964	0.3280731152745613	0.1025663635545449

0.3723863120644353	0.2441238987544551	0.1027617600870170
0.4105950731207692	0.3253030737733858	0.1100991261811711
0.4990664335893006	0.2520938525483175	0.1195209731673438
0.5387834580718122	0.3381158912829211	0.1293761093212425
0.6243049446750339	0.2497313853365358	0.1243013476558286
0.6646830031762831	0.3331249978748511	0.1299651381317157
0.7496508529163448	0.2497900047577495	0.1215239208239238
0.7907961626752511	0.3337372584200533	0.1241772089177209
0.8749938396458601	0.2495248372760501	0.1138792489382730
0.9165680822369944	0.3329915245796091	0.1167024276343558
0.0002467443941523	0.3740371301229193	0.1152720593272650
0.0427632161320160	0.4572490137708756	0.1185564564518851
0.1256505971431437	0.3733453822285019	0.1109709869201751
0.1690485080814992	0.4562236591676362	0.1178892475755782
0.2501687872325329	0.3721250755145534	0.1084696405403527
0.2964520479512214	0.4548223604069394	0.1202364918715165
0.3695737019713995	0.3662059239351567	0.1100407284365124
0.4215649429095503	0.4456838660261671	0.1269757232659991
0.4899180744716880	0.3771582250484329	0.1266365941061104
0.6219244724157963	0.3738551396110938	0.1375071444731133
0.6677320011036910	0.4565220607305486	0.1600320645443133
0.7489745332026408	0.3755388779289008	0.1295969747997860
0.7912860266971460	0.4598938809265977	0.1298645265396803
0.8747335729992222	0.3749061891816481	0.1212210828384400
0.9167115994872721	0.4581806019345886	0.1217021749531319
0.0005878408682614	0.4989926890680493	0.1201823169370663
0.0421480610000405	0.5821475260822050	0.1194851650399070
0.1267809967725498	0.4980078366309533	0.1198445604122272
0.1683689262987754	0.5811714062250406	0.1220645850683158
0.2536441265337741	0.4967340254331521	0.1225102372814503
0.2941857472075989	0.5798472201501190	0.1278912390883720
0.3824055443478056	0.4947341574267795	0.1312900271172944
0.4179811239862526	0.5770434572169278	0.1431013124546785
0.7506184551677038	0.5026941230128985	0.1378001004610434
0.7867766037323566	0.5858082025151552	0.1297922274034375
0.8747368917923676	0.5002602724541622	0.1239520543586785
0.9153168960081982	0.5834343709992120	0.1206591213205452
0.9998833495033989	0.6239468584760873	0.1173444876655683
0.0433918536363922	0.7069100582806485	0.1114954965258074
0.1262020783510103	0.6228940525381612	0.1199907338716289
0.1687468226146011	0.7061421867155956	0.1177626121084726
0.2522322243014000	0.6219710191581374	0.1254434798056487
0.2942370146702822	0.7052112701861516	0.1259481716866820
0.3775109416843612	0.6200647053671469	0.1359850887798504

0.4199715842341160	0.7044792859963678	0.1364873028695146
0.5009643243289545	0.6228230328826536	0.1686562854387697
0.5473793401046260	0.7060023021591342	0.1433517172735063
0.6792107256455812	0.7032718104217345	0.1275985614932852
0.7482610073773930	0.6349086782863587	0.1270266251612094
0.7994140315388137	0.7139144995659309	0.1086057547232275
0.8725792580465042	0.6254948570806380	0.1191211493111254
0.9189171609483044	0.7082143402381372	0.1075262538633534
0.0021706723011657	0.7488039348816433	0.1048527011277894
0.0440929552649992	0.8319197829448927	0.0975287709480179
0.1270609412906091	0.7479290570715308	0.1118860606664484
0.1688346124685101	0.8310529806891683	0.1068441287784312
0.2523998182938954	0.7471209046492420	0.1205377844926617
0.2939649730124704	0.8306020612185439	0.1175653203267823
0.3782216238889550	0.7462868224424443	0.1299259344607687
0.4193460742612389	0.8302722337402719	0.1262172678307250
0.5044385078911808	0.7467289356283372	0.1363792126356164
0.5446911185163101	0.8301395337891975	0.1284790579177964
0.6298258968038319	0.7418807789785345	0.1316840840932781
0.6697180700950097	0.8278773288909729	0.1203085812140473
0.7583497780353395	0.7548652204762625	0.1089319445667042
0.7963957613380085	0.8358423676030962	0.1008119948682094
0.8805545431391493	0.7521127690547826	0.1006353288329472
0.9208184641731567	0.8344066603168236	0.0929237923573854
0.0032628233820598	0.8746045431091454	0.0918163716588621
0.0438649996231280	0.9574504854641285	0.0890056973708143
0.1272777402659773	0.8729197395038024	0.0992917794112826
0.1685678433334478	0.9560360631212651	0.0955021762698602
0.2522038532310784	0.8723567794366865	0.1102942393424186
0.2935274545582085	0.9557316146726256	0.1072180730243612
0.3774632694617497	0.8721464457629058	0.1207822846015288
0.4184378338616794	0.9557979365743174	0.1182642945887537
0.5025930815938454	0.8721959834129489	0.1259659135018283
0.5433974639780526	0.9560895459472615	0.1226579146864633
0.6278015785268117	0.8707207473642680	0.1229269626480852
0.6683355390214666	0.9553666943987724	0.1183249507191912
0.7523795594007215	0.8742283965699109	0.1077712866857661
0.7930373816203939	0.9574489520967645	0.1046080078353444
0.8787808931355401	0.8762292175465795	0.0929060562902936
0.9189763050214099	0.9586837418971765	0.0915570552295003
0.6254592942871806	0.5765720941037324	0.3468223745722146
0.6526320147784456	0.5357717772685564	0.4035015365845776
0.6234057234007885	0.4540928971023607	0.3959581481462101
0.6557475886586962	0.4176056786151605	0.4452104583157576

0.7181256888667478 0.4613263506693361 0.5033221983468831
0.7465195500342454 0.5416389883163844 0.5138388785687363
0.7135015997107071 0.5782952954723829 0.4659824224668486
0.6300977739030466 0.6491614305687570 0.3814341775383727
0.6938460281030385 0.7885327765319691 0.3673988812247995
0.5303789993960075 0.5922442565320806 0.2190710676302235
0.6722960868254267 0.6282444150578379 0.1376999902083373
0.6364514771394274 0.4865257543135013 0.2091224341491648
0.4321335104817003 0.4412439283432670 0.4414852311294421
0.4071900394170745 0.4640928577501724 0.3910754186662895
0.4970040127549582 0.4537258518819532 0.1368714395655412
0.5866326970176077 0.5411948071089755 0.2268507528241367
0.7366065368828325 0.6410603481702500 0.4737206637266876
0.7948555819020070 0.5756940818321405 0.5595010265701713
0.7440887215028882 0.4327556462844102 0.5410916836241512
0.6320968761129447 0.3546881408080899 0.4384758273699447
0.5736410937527691 0.4196274545838066 0.3524400144797067
0.6746015642032116 0.8093287382137841 0.3101763010378601
0.7556553705271891 0.8295281295762573 0.3816575502936347
0.6571668498484625 0.7808770871962040 0.4256925507496978
0.5840727891644256 0.6475119597205498 0.4374289839414381
0.6881754586979282 0.7148068069051621 0.3427181532900355
Total energy (E): -1300.01029855 eV
Temperature (T): 333.0 K
Zero-point energy E_ZPE : 94.418 kcal/mol 4.094346 eV
Thermal correction to U(T): 104.481 kcal/mol 4.530732 eV
Thermal correction to H(T): 104.481 kcal/mol 4.530732 eV
Thermal correction to G(T): 82.428 kcal/mol 3.574435 eV
Entropy S : 277.083 J/(mol*K) 0.002872 eV/K
Entropy contribution T*S : 92268.593 J/(mol) 0.956297 eV

RhN4 SA (pyrrolic N): IS1
1.00000000000000
19.7297992706000009 0.0000000000000000 0.0000000000000000
-9.8648996353000005 17.0865073798999987 0.0000000000000000
0.0000000000000000 0.0000000000000000 15.0000000000000000
C N Rh H O
139 5 1 19 3
Selective dynamics
Direct
0.0018177275667653 0.0001226380865090 0.0861423490650826
0.0423339899788176 0.0828933175311352 0.0883657538959013
0.1265451393028389 -0.0012857047407447 0.0888626116815592

0.1670239649082165	0.0814875901035361	0.0889335114809687
0.2515950541871011	-0.0018807103019839	0.0996690495485735
0.2925103933018996	0.0812447700322699	0.0986569426134739
0.3765546611307840	0.9982442758988750	0.1127731541536858
0.4175404165531730	0.0819437718933799	0.1126536925749608
0.5016075072733190	0.9990612372707924	0.1206747835372114
0.5423395570724557	0.0831718068609856	0.1204683895385496
0.6265543542791278	0.9984427782889732	0.1198723953815202
0.6672842501649019	0.0825199843900024	0.1192620751389029
0.7511888389668238	-0.0004569004916607	0.1096796148353343
0.7922420100653802	0.0832566071637621	0.1100178058356606
0.8761980704688037	0.0002660092011292	0.0945527460059307
0.9172079165852773	0.0834251848598108	0.0970284774126106
0.0004668719970205	0.1248133201112957	0.0933087034058576
0.0418873430885021	0.2080892708674424	0.0969790507905633
0.1251189684259517	0.1234202511535639	0.0882859205876795
0.1657272502091434	0.2062493774995808	0.0908211394862697
0.2497969788608903	0.1220264058453385	0.0927980035470548
0.2899149833387841	0.2044546902804855	0.0939722753018939
0.3756878655659998	0.1232294720112746	0.1061065513728931
0.4162718723199011	0.2064969630534836	0.1083651669388410
0.5005664961780681	0.1255121451819562	0.1184460270818264
0.5410044081513469	0.2101469912217957	0.1214896647639417
0.6255208529320422	0.1247300472967621	0.1213264039728399
0.6662694204795324	0.2086475071418018	0.1230602616705422
0.7505065533379485	0.1250212501747699	0.1160003895408182
0.7914821456179330	0.2087013812605856	0.1176441048748104
0.8754075186470225	0.1250708115780356	0.1046411647262895
0.9167835477721943	0.2084853620852415	0.1075862751343359
0.0002090229553773	0.2499012867138206	0.1041448809307052
0.0420076122357355	0.3331059312700307	0.1085191480834498
0.1250302991052226	0.2490920990098000	0.0949760932501384
0.1671017564405086	0.3323919910644044	0.1016410630873567
0.2481317808432144	0.2463237987674435	0.0928623228770436
0.2886662130817743	0.3288628122849394	0.0996697460003648
0.3723749391331044	0.2449414521616542	0.1015390200124575
0.4104788460318320	0.3260132387131803	0.1077095964097076
0.4990146028945875	0.2530168612617387	0.1191811523894660
0.5387526429473593	0.3391580805479280	0.1284388092447275
0.6242063235953638	0.2506952882160920	0.1250212077403256
0.6645175301680090	0.3341570032770649	0.1303460924076717
0.7495789685931200	0.2506219955632186	0.1222682014220349
0.7907097769770802	0.3346448188434681	0.1241680711098143
0.8749810862056486	0.2503023542928525	0.1141527060517046

0.9165756579149638	0.3338437226088548	0.1161784493440440
0.0002559331485103	0.3748529156304641	0.1137605880407614
0.0428123147249759	0.4580615313875915	0.1161731676148939
0.1257256145601014	0.3741750734471024	0.1082375453108822
0.1692261650609305	0.4570778732817894	0.1154701869916828
0.2502990891468771	0.3729750459984279	0.1052236689555004
0.2966718118528253	0.4555824385074709	0.1178275402471558
0.3695427421150471	0.3668662290488473	0.1071001041998088
0.4219169577948737	0.4464525300998001	0.1239270899776603
0.4899817391083646	0.3784435506491434	0.1241511900261049
0.6217514226703316	0.3750318072171304	0.1373750351102121
0.6679726589899201	0.4582157883761767	0.1587816420137519
0.7488989719545839	0.3765484290559543	0.1292351257455671
0.7912886918524276	0.4609714151609649	0.1275134861218133
0.8746882517481447	0.3757599865410039	0.1203073702066265
0.9166809811020045	0.4590347503598869	0.1192396777732486
0.0005740035118811	0.4997624990662015	0.1173366474975195
0.0421569955324121	0.5829319023115410	0.1161393520050556
0.1268942045427001	0.4987768207167713	0.1175361447482909
0.1684554491893171	0.5819317076369790	0.1202436908255066
0.2538286398241496	0.4974769537527954	0.1205654266823368
0.2944037829084000	0.5805348509318781	0.1267666777762334
0.3826438123459064	0.4952962097563401	0.1292294518844341
0.4184762071816226	0.5776845363856559	0.1419092337270430
0.7506153811065078	0.5038590615071027	0.1342088823888182
0.7864642814806393	0.5865673016338785	0.1227992149022080
0.8746732946037735	0.5010956410411782	0.1204327541914066
0.9152158065458014	0.5841769161933894	0.1154731077440536
0.9998270613781041	0.6246348492800080	0.1128272134731479
0.0433178795181262	0.7076342373197776	0.1070585953601282
0.1262416094235058	0.6236526860989730	0.1176022698366977
0.1687259787038571	0.7068834226667219	0.1158098556937549
0.2523300223077723	0.6226069754742282	0.1244095273914094
0.2942555085441600	0.7058823077095638	0.1253424808962657
0.3777935076331922	0.6206475509505938	0.1349999538841517
0.4201710335044976	0.7051390488210953	0.1355978459213894
0.5017379963864574	0.6235914263602922	0.1664527601125806
0.5476586718206458	0.7066470936789757	0.1406582153384072
0.6788992092654923	0.7032419701250179	0.1198717945811706
0.7473003169853637	0.6352516317101915	0.1179025352593829
0.7991383493205237	0.7145220694416001	0.0999541399298991
0.8723635013810044	0.6261164450623393	0.1123528082882699
0.9187546942099649	0.7087867469263324	0.1005205903108827
0.0020548547978391	0.7494398638321020	0.0992886522264783

0.0440492333704304	0.8326754449695155	0.0930829859638988
0.1269862553517882	0.7486264861277226	0.1090728727628941
0.1687427083625561	0.8317880539857357	0.1048472555241318
0.2523482878125400	0.7477835618789520	0.1196821676094094
0.2938887169878829	0.8313221389069902	0.1171646227016358
0.3782950914886135	0.7469463467230172	0.1294569718220110
0.4193638603640560	0.8309845811919843	0.1258822822377026
0.5046953827194393	0.7474517988245626	0.1346730046242537
0.5447561591223057	0.8308689145590333	0.1268428718865592
0.6299484744488290	0.7423620433489561	0.1268918842379947
0.6696514927983067	0.8284622139819308	0.1166463914407478
0.7581805939103640	0.7554737386347267	0.1012491151306687
0.7961809842807283	0.8364815528538774	0.0952728696808844
0.8802522604936956	0.7526197512994037	0.0933291983987868
0.9207021581009506	0.8351668339644425	0.0869993641117209
0.0031913866506696	0.8753555894207155	0.0872005972261747
0.0437735102068443	0.9582159163820179	0.0860317837809583
0.1271483926569080	0.8736155534973348	0.0967924558662168
0.1684442771463632	0.9568292319425347	0.0942804640478642
0.2521068018813943	0.8730872864307566	0.1096091244475519
0.2934417551523871	0.9565055184794005	0.1071985411350010
0.3774226967812694	0.8728662848886668	0.1207838960172214
0.4183658071498521	0.9565253885692592	0.1184391948732869
0.5026353022236546	0.8729283819230920	0.1252379484874210
0.5433596416660181	0.9568446677419733	0.1222899682975173
0.6278115282270181	0.8714104310075135	0.1206792093063501
0.6682675442779820	0.9560899001884668	0.1168191248194257
0.7522554989344949	0.8749080828855953	0.1037972106597009
0.7929347965630178	0.9581728430705718	0.1019642945108192
0.8787416875021058	0.8770111474119452	0.0877311394383671
0.9189200663179865	0.9594799104346868	0.0880715236585015
0.6138010360301084	0.5641922963849578	0.3530848291558079
0.6416875795077985	0.5223438077264752	0.4074909170129219
0.6141207828797589	0.4416228589110937	0.3943580604739236
0.6441366784115919	0.4030116153590265	0.4436388784618912
0.7026657695190729	0.4438365402385891	0.5071363078904727
0.7296055247633152	0.5232415252418632	0.5225560646499753
0.6987380052793352	0.5619019078651160	0.4744274837000611
0.6111802916439623	0.6311402963033277	0.3942650107715026
0.6601471314058285	0.7681231023797004	0.3906707726380952
0.3516533912468944	0.3444135328029683	0.4439470133685172
0.2648071103687831	0.3072772532978265	0.4282754450549380
0.2352535177426254	0.3185571343157886	0.3479797700803297
0.1552178172562210	0.2867512168702737	0.3341178416640401

0.1009706667010259	0.2414176464876063	0.4004053885981230
0.1299391645888946	0.2298975101036564	0.4811961657604368
0.2103071538351902	0.2625844023072622	0.4943362283058729
0.3899333440642293	0.4317848757370795	0.4654508237866335
0.5324210995098022	0.5936798936792268	0.2163103950762893
0.6715854500891049	0.6280363697319613	0.1286691355469009
0.6379133118498482	0.4891511702737237	0.2084161450261192
0.0208868830680698	0.2117974296049376	0.3872484968081301
0.4973174240072739	0.4544627005160316	0.1332192069036166
0.5869852640040930	0.5420621384388696	0.2283294845908358
0.7203297773088846	0.6239485696562992	0.4864292468343714
0.7747719570935638	0.5550586730348467	0.5724757991572932
0.7265147917359878	0.4135288191735532	0.5454148304510185
0.6213359756558717	0.3406018717539968	0.4330667817912224
0.5667317192662130	0.4095833767211785	0.3471099508764189
0.6024316797703582	0.7612347665792024	0.3765339175767313
0.7059416080376403	0.8180309878749993	0.3540446164907969
0.6709956187267337	0.7753234024319021	0.4627880583015004
0.3810968017780383	0.3375015739636676	0.3850542516093116
0.2312986080836621	0.2527933051095821	0.5577728003215079
0.0891535715551396	0.1952369110104037	0.5340848716390898
0.2758639011824948	0.3529538304158925	0.2948455988916956
0.1339916614512386	0.2968754597009647	0.2712192140940085
0.3631961019594648	0.3156441109227424	0.5000813092309387
0.0032655599807832	0.2050255542320023	0.3226715393114790
0.9847964380790449	0.1650651482864710	0.4254531495716131
0.3574781954966030	0.4402115442811157	0.5205665817165672
0.3858229932572495	0.4635465034255195	0.4067382792123719
0.4983640937937204	0.5169812976297318	0.4851460979295913
0.5642903421346576	0.6239943782707322	0.4529627686900234
0.6637119951831545	0.7008451648223381	0.3583481600411865
0.4694759642525884	0.4596139646924369	0.4894305098735006

Total energy (E): -1411.81769049 eV
Temperature (T): 333.0 K

Zero-point energy E_ZPE :	200.718 kcal/mol	8.703949 eV
Thermal correction to U(T):	216.541 kcal/mol	9.390089 eV
Thermal correction to H(T):	216.541 kcal/mol	9.390089 eV
Thermal correction to G(T):	184.776 kcal/mol	8.012637 eV
Entropy S :	399.111 J/(mol*K)	0.004136 eV/K
Entropy contribution T*S :	132903.849 J/(mol)	1.377452 eV

RhN4 SA (pyrrolic N): TS1 (imaginary frequency: 179.302201 cm^{-1})
1.00000000000000

19.7297992706000009	0.0000000000000000	0.0000000000000000
-9.8648996353000005	17.0865073798999987	0.0000000000000000
0.0000000000000000	0.0000000000000000	15.0000000000000000

C	N	Rh	H	O
139	5	1	19	3

Direct

0.0017791126752242	0.0012014286456844	0.0806656516056923
0.0424655993284383	0.0840660828043650	0.0818252961391839
0.1266187209016584	0.9998655252762481	0.0819954335123482
0.1672940304031127	0.0827290554432832	0.0821533682715560
0.2517160462750125	0.9992500794770619	0.0935644860670440
0.2928072056998234	0.0825319641759523	0.0942953284495390
0.3766190732192044	0.9993462221748863	0.1087690624391764
0.4176577241610969	0.0829996643369477	0.1110985539059150
0.5015945488745480	0.0001130703391609	0.1184700785790725
0.5423585061540688	0.0841188860653885	0.1198950234768704
0.6265166390649515	0.9995369308733746	0.1187256109094190
0.6672978965235173	0.0835679443998343	0.1180935341512439
0.7512142752599755	0.0006922280000986	0.1086342686433576
0.7922883399449919	0.0843984160609414	0.1075185289688438
0.8761275887377244	0.0013043513073450	0.0916657728096267
0.9172822754009337	0.0846201711596648	0.0924813683700256
0.0004766168405619	0.1260282331581112	0.0875420164713017
0.0419235748981570	0.2092345085160116	0.0921088710580419
0.1253330392132740	0.1247420601194738	0.0816890795226170
0.1661020926946721	0.2076937073540950	0.0862527856422929
0.2501905225593240	0.1234366419679632	0.0879878603412211
0.2904323052581133	0.2058862062366002	0.0922745791168663
0.3757778723242742	0.1243537523219838	0.1049715525617245
0.4162945967574552	0.2074390418501582	0.1115662168831658
0.5004895539945100	0.1263251145146916	0.1190381093439294
0.5410273261506663	0.2108294171400119	0.1255079089835748
0.6255537983079562	0.1256995145206906	0.1211988359047709
0.6664088243140417	0.2095607912002665	0.1245996199779396
0.7505440022522039	0.1261072369837775	0.1141585135058584
0.7915589387897181	0.2097785114058348	0.1163486604525617
0.8754204399535486	0.1261915373841490	0.1009280257755492
0.9168099224084779	0.2096199200204604	0.1041317420525173
0.0002604190373140	0.2510465820172328	0.1004598376238621
0.0420546065103189	0.3342044118383958	0.1064680500976973
0.1252466549386544	0.2504163230646313	0.0912280197629131
0.1671548545741707	0.3335297809008667	0.1004298633817263
0.2485194071102151	0.2478926640833010	0.0911046418420308
0.2886201641943818	0.3299422418350552	0.1021686183981569

0.3723762373978681	0.2459218186691830	0.1045262303527821
0.4101741864517304	0.3266070543732467	0.1168184833965098
0.4988226919611767	0.2534016074239778	0.1260017668218082
0.5386099313554127	0.3389620704096152	0.1403643325019628
0.6242572918014542	0.2514400702263553	0.1293703592801143
0.6646391633584765	0.3348211702242519	0.1372285740034678
0.7496725223620329	0.2515819963951028	0.1231238106353091
0.7908249726860029	0.3355933337638234	0.1268745529241433
0.8750433649549031	0.2513581705035544	0.1122008364792355
0.9166238979852916	0.3348401621588832	0.1156023079184653
0.0002922249226005	0.3758806706584575	0.1129685798084925
0.0428342798719979	0.4591159898769730	0.1163103502039037
0.1257872585519847	0.3753298683508712	0.1072707570894990
0.1691416993526474	0.4581280362042980	0.1158085114817088
0.2503027653522209	0.3740467426325659	0.1075065288473590
0.2962128943340299	0.4564553114866401	0.1230249216622460
0.3691751643012959	0.3676799381798511	0.1158535185062582
0.4191428207999666	0.4457825960174838	0.1398253388738685
0.4883223699469210	0.3768848338560763	0.1405954878775787
0.6216750128444631	0.3751054936650755	0.1472908330220245
0.6684751621419849	0.4587695110609076	0.1687924673103566
0.7491565346132405	0.3773517150770237	0.1349963803280219
0.7917089318390179	0.4618199252261269	0.1350837031544558
0.8748132956953845	0.3767333314043070	0.1220344469364813
0.9168434286667789	0.4599835375951604	0.1222003914869373
0.0006816520137738	0.5008293856164419	0.1186219331977128
0.0422426973558607	0.5839863504073325	0.1166847622626438
0.1268554175269885	0.4998997035919298	0.1173411128616081
0.1684066568578746	0.5830320678197453	0.1190878403986030
0.2535847366926745	0.4986020171444596	0.1227161318839887
0.2941587295265029	0.5817624521263115	0.1276297710263452
0.3815423817803342	0.4959622083249786	0.1394017708571598
0.4177801588493028	0.5792014377815272	0.1478056346041094
0.7514408949896209	0.5048721345251304	0.1440403053487622
0.7874068859310809	0.5876783451179441	0.1345493193829235
0.8749661940100009	0.5021363471631493	0.1260502315209004
0.9155038358864116	0.5852793140666301	0.1206512120310964
0.9999984044375386	0.6257992278700842	0.1146516898741028
0.0433211958243851	0.7086258574644809	0.1067749734072751
0.1262444604263817	0.6247552904409329	0.1161272804128482
0.1687360455586674	0.7079808400319428	0.1125739840707941
0.2522422708554680	0.6238615432978001	0.1229228551845398
0.2941948474499561	0.7071047999686260	0.1221896326863905
0.3774374618862484	0.6220970070862902	0.1366540355055701

0.4198546012384235	0.7064787841960976	0.1351519777030704
0.5007991273525350	0.6256941684291094	0.1709562579521233
0.5471310512776338	0.7083290432724818	0.1458602057141834
0.6803659647911915	0.7070467878218392	0.1342817354961985
0.7494013028048041	0.6376645312303472	0.1335548646806277
0.7993344647742801	0.7157101112521110	0.1094944067630365
0.8728474262885777	0.6273884532768529	0.1198468322458898
0.9187445932665096	0.7098513172064697	0.1051167147070022
0.0019489379884838	0.7504563457304343	0.0998614524159794
0.0438663196245132	0.8335422646722196	0.0904907495404976
0.1270115127595917	0.7497040076203521	0.1059250099529750
0.1687968303407421	0.8328382902808138	0.0999196156873262
0.2523858214182891	0.7489910241537773	0.1153568244904248
0.2939212029596895	0.8324202439130051	0.1119750809196973
0.3781111271370588	0.7482098367426520	0.1267907997546595
0.4192422841836652	0.8321394461614264	0.1226637428740632
0.5042535383027785	0.7488576865915814	0.1360859517273533
0.5446233290568863	0.8321716173188153	0.1274287154291602
0.6303172851167486	0.7445864797766997	0.1360752762750439
0.6699043762863057	0.8300737215662780	0.1214470479758197
0.7582796307877676	0.7568504103354980	0.1099079274471623
0.7963560562253091	0.8377777344517300	0.0987591665090665
0.8801970713469724	0.7537163996114528	0.0978514260112492
0.9204077053051660	0.8358783080055533	0.0874061522252887
0.0029508057229087	0.8762041438690545	0.0843900186319336
0.0437504963195974	0.9591991822646486	0.0805053771385645
0.1271823010557336	0.8746772139746780	0.0916538610915621
0.1685899711212125	0.9578752149218457	0.0876752313369890
0.2521630917134666	0.8741963363379054	0.1039270215617318
0.2935311707291278	0.9575963574163036	0.1016002983883612
0.3773474810417940	0.8739566087458135	0.1162533389381775
0.4183655429195541	0.9576166271188087	0.1146632117550433
0.5025134358632424	0.8741135103162726	0.1235356349110402
0.5433600414556220	0.9579650587901215	0.1205032747844409
0.6278059313542030	0.8727768574818254	0.1224309111040223
0.6683322992716794	0.9572800329968433	0.1166435424419083
0.7524399530149948	0.8761433433003435	0.1066385171480540
0.7930682515760388	0.9594056904127113	0.1015020115936655
0.8784649045276647	0.8779201789352838	0.0878499452012106
0.9188011514832127	0.9604371860322277	0.0850151047772142
0.5934607125011745	0.5470694594417908	0.3802974789792953
0.6442173270599868	0.5214904479866890	0.4213582177287392
0.6254695103585264	0.4429689993013761	0.4077332645235704
0.6689646447741028	0.4135175184971260	0.4474126798537487

0.7306671959514871	0.4608092944097595	0.5049683139110377
0.7481984395416357	0.5376186400253280	0.5221915894311219
0.7061945303245385	0.5681308521803770	0.4802584689311132
0.6038925348888584	0.6259611809623638	0.4022600056929735
0.6833951779289723	0.7625394427843024	0.3775353902293148
0.3644405763831962	0.3498647676506699	0.4362256651275447
0.2785489904703796	0.3179501915476713	0.4230080340061318
0.2499814387202400	0.3353900459551200	0.3456844478614119
0.1702693808261197	0.3038804711220173	0.3310353016709147
0.1152362970781808	0.2528282337768243	0.3936889644788920
0.1432243164390216	0.2371970093896893	0.4729395563925133
0.2233305084940497	0.2695361263570096	0.4868239685841704
0.4099939046428711	0.4350455255089750	0.4740007700881982
0.5321213389489895	0.5958351677392251	0.2202997136453413
0.6753378177203645	0.6325263543446455	0.1511758538591534
0.6386286725482485	0.4906874014377655	0.2159352444965192
0.0354785154449340	0.2210232675553529	0.3782355027034279
0.4934833597068272	0.4509559780427571	0.1573695824160994
0.5878213776967620	0.5456226104737310	0.2277795652279803
0.7200628785899506	0.6278093528341114	0.4949945627405277
0.7946025773794422	0.5735681161438334	0.5692753231761445
0.7643330794669544	0.4374620962568712	0.5370753029923788
0.6543335926917081	0.3532784420154331	0.4345234428934862
0.5758999490442489	0.4056372506777301	0.3651023640632998
0.6288426579870531	0.7609207136255873	0.3586052881086717
0.7306187363069524	0.7987832177312972	0.3310634054471145
0.6990503613797024	0.7852391799486070	0.4458193386712951
0.3927853637609491	0.3508146693907140	0.3725884200867099
0.2437659305847905	0.2557289633436197	0.5481929094598691
0.1017236422856300	0.1987370960691639	0.5232266257009230
0.2913908775985803	0.3735191763388738	0.2951485055122622
0.1496529028374791	0.3176357729376646	0.2699714109014827
0.3749367652810364	0.3128620265476358	0.4825500078505459
0.0192091332929037	0.2184454816622930	0.3135638835773137
0.9992316881686277	0.1720497209536591	0.4131266355740645
0.3825263128957489	0.4385802547865707	0.5375116903161892
0.4025868010005440	0.4748249130286651	0.4272297762031598
0.5304358366672496	0.4985279226653171	0.3878202403166028
0.5554541458129285	0.6371486145816965	0.4395353527839618
0.6739523125428916	0.6851429111281830	0.3714885151792078
0.4874742491292482	0.4580918484666253	0.4857070881458903

Total energy (E): -1410.141400 eV

Temperature (T): 333.0 K

Zero-point energy E_ZPE : 197.970 kcal/mol 8.584797 eV

Thermal correction to U(T): 212.910 kcal/mol 9.232644 eV
Thermal correction to H(T): 212.910 kcal/mol 9.232644 eV
Thermal correction to G(T): 184.061 kcal/mol 7.981654 eV
Entropy S : 362.469 J/(mol*K) 0.003757 eV/K
Entropy contribution T*S : 120702.184 J/(mol) 1.250990 eV

RhN4 SA (pyrrolic N): MS1
1.00000000000000
19.7297992706000009 0.0000000000000000 0.0000000000000000
-9.8648996353000005 17.0865073798999987 0.0000000000000000
0.0000000000000000 0.0000000000000000 15.0000000000000000
C N Rh H O
139 5 1 19 3
Direct
0.0019125159962981 0.0021163090982270 0.0722725383352441
0.0424959890014007 0.0849306297562576 0.0749338426713198
0.1267609658155811 0.0006050402466046 0.0744448235907655
0.1672845289032395 0.0834208739507426 0.0760825823855705
0.2517408394107995 -0.0001343070003844 0.0876579795977132
0.2926941705997856 0.0830705062716743 0.0896906473478620
0.3765598316380029 -0.0000631580582613 0.1043180381527016
0.4175207119510815 0.0834810883736377 0.1075016926353178
0.5015468257799340 0.0007795517241469 0.1145813495883982
0.5423346666324819 0.0847015352987518 0.1167374638942344
0.6265559875124419 0.0003161076894036 0.1144221194340025
0.6673546139884687 0.0842765500936761 0.1146591512150537
0.7513098015200930 0.0015923904253061 0.1031499522756730
0.7923185069610369 0.0852172815998390 0.1026760152175319
0.8762389730330844 0.0023033397858788 0.0841527268879089
0.9173395772847057 0.0855595064825882 0.0859758836631786
0.0005204117602169 0.1268801139478978 0.0815438741382498
0.0418202194942291 0.2099643931342539 0.0879870947165379
0.1253109391159608 0.1254391468419857 0.0760630417474961
0.1658940886462006 0.2082252077853488 0.0825339102314296
0.2500186060399281 0.1239492344149787 0.0834399968571773
0.2900518996412605 0.2062363996504406 0.0891761120219510
0.3755325449655486 0.1246986274829428 0.1016117391141746
0.4159514308570946 0.2076811666460574 0.1093033575816090
0.5003337641239274 0.1267487622176770 0.1160840844311109
0.5408390289000170 0.2111168487414236 0.1231175129190770
0.6255530850005583 0.1262577349070497 0.1184306383654125
0.6663848177653782 0.2100120871906976 0.1221915050879442
0.7505805726461399 0.1268507965380428 0.1105352581658045

0.7915941256703365	0.2104900948966322	0.1133935619173333
0.8754215953887197	0.1270240753340078	0.0957221359517006
0.9167513432893419	0.2103672466335652	0.1001470410408884
0.0001712983873403	0.2517603046878488	0.096979211568182
0.0419165483231775	0.3348316801088537	0.1041952280833547
0.1250707694989235	0.2509959109184541	0.0882188155283139
0.1667951953064993	0.3339956259544987	0.0983760382089650
0.2481674237478211	0.2482280945036143	0.0882520608607702
0.2880152304380736	0.3301643034859548	0.1000280371545844
0.3718383430470147	0.2459968676380944	0.1024201345183424
0.4094269668433541	0.3265031271996445	0.1154081098212307
0.4984816892175289	0.2535530570476633	0.1241864441396082
0.5382920622828280	0.3390152661196970	0.1385684065337416
0.6241072502277789	0.2516676700310577	0.1268785689720187
0.6646209558167396	0.3350460235522764	0.1336213815286115
0.7496870062867445	0.2522171650987869	0.1205308998442241
0.7908642908652459	0.3362233858935166	0.1238962029068140
0.8750432379068327	0.2520664410273512	0.1091628289662002
0.9166143251166259	0.3354982373566833	0.1132088308286619
0.0002560868288357	0.3765614706291088	0.1109267539713045
0.0427596751786847	0.4597896674996647	0.1143691069553610
0.1255456387538077	0.3758674366667938	0.1054314169525725
0.1688440131289488	0.4587036941547483	0.1139759069091034
0.2499049589680994	0.3744871838803226	0.1053967264316796
0.2957492633534955	0.4569313285227894	0.1207243845727318
0.3683560666993779	0.3676648378932486	0.1141843765867290
0.4176171493117879	0.4456239989332883	0.1385210020137143
0.4873281999466527	0.3760598711966833	0.1399844439540728
0.6217814328833231	0.3753363903773309	0.1433143319398900
0.6689331074995162	0.4590262307770879	0.1616522115580205
0.7491677510930433	0.3779939828368409	0.1311166305091050
0.7919835740643980	0.4625741988698536	0.1308128990184211
0.8748223703346901	0.3773941964124318	0.1194030239202732
0.9169339437568275	0.4607121294005691	0.1193845994328585
0.0007209883267407	0.5015855136574225	0.1161767928830285
0.0422510950092333	0.5847606499815738	0.1137376251018765
0.1267077064939627	0.5005986268888633	0.1154745393611162
0.1682354817464725	0.5837659400342233	0.1168472037235945
0.2532105473927106	0.4991991920301379	0.1203148478097939
0.2937562652553877	0.5824180051636396	0.1243722191341559
0.3810722534782269	0.4965637475566834	0.1364131678250273
0.4175636253779181	0.5802420903831413	0.1418625709899994
0.7519229639857218	0.5057270560046010	0.1396445155192503
0.7881200540374677	0.5890045470481404	0.1316208230576203

0.8752239591435274	0.5029974893879768	0.1225046471378594
0.9157632361314153	0.5861941252951375	0.1168739896749673
0.0001440673099173	0.6266641903479775	0.1108213418583341
0.0434337770076055	0.7094176150685262	0.1018624334212087
0.1261999687482918	0.6255417458203386	0.1132977896096113
0.1687202114229047	0.7087290011966322	0.1087420499210384
0.2519927248322836	0.6245902434935794	0.1200833065306011
0.2941037865514458	0.7078769363104856	0.1184860071085567
0.3771225938998260	0.6229464744355362	0.1313010893148354
0.4198031788753325	0.7073123919120105	0.1292158942791024
0.5004158417215102	0.6273457157365213	0.1630791431518768
0.5472550785990095	0.7096762159664000	0.1392772949039267
0.6818190622976698	0.7096798909520295	0.1313081667379978
0.7511217454930906	0.6399758585363469	0.1315118018967102
0.8002241266909326	0.7173377155474139	0.1044524172788985
0.8732945282996448	0.6285132137647437	0.1160510689623964
0.9191144476406676	0.7108931799622454	0.0998879500390728
0.0022213188703064	0.7513300943131962	0.0938182593290075
0.0440853009125574	0.8343285058042179	0.0829038506853322
0.1270473932918509	0.7504265596320622	0.1009043153971902
0.1688600520454381	0.8335202975464570	0.0938260791162039
0.2523602867378331	0.7497551186115848	0.1114161109472176
0.2938920076735195	0.8331377713407085	0.1073073014506404
0.3780247635143756	0.7490161192472908	0.1219657880331670
0.4191730571894193	0.8328941731504047	0.1178882799391220
0.5042491895917961	0.7499173892790710	0.1294648728119478
0.5447300933378355	0.8332025305565217	0.1216433298573488
0.6308483330573923	0.7461796252168753	0.1314952795924349
0.6702997550071230	0.8313786175310601	0.1160028046598599
0.7591911422369076	0.7585537922193560	0.1043514972611726
0.7968703996205193	0.8391015110832002	0.0916752532883926
0.8807832886819460	0.7549586579478775	0.0914931608614101
0.9207472954013165	0.8369183557351446	0.0791789457903883
0.0032237811290876	0.8770681080400800	0.0758095531416515
0.0439258682960017	0.9600482420045467	0.0719082893823646
0.1273699696328112	0.8754311680576584	0.0843764445171286
0.1687210366012212	0.9586148860102545	0.0804792393614338
0.2521868585770117	0.8748451921758511	0.0983777137950188
0.2935460799904118	0.9582244837655428	0.0961392008096172
0.3772642065316266	0.8746602039931974	0.1117625380930038
0.4183293455983971	0.9582967844512541	0.1103974855587169
0.5025065289597609	0.8749671655230321	0.1184765073973811
0.5433905266054443	0.9587643753611328	0.1161287166603573
0.6279664354429019	0.8738429585854465	0.1168469048806530

0.6684651112440221	0.9582162937810432	0.1115284117467602
0.7527602991219647	0.8773057540408110	0.1002962421817796
0.7932386029150232	0.9604587338701953	0.0948799665035142
0.8788177405282278	0.8789970324571582	0.0794285035638245
0.9189621084459341	0.9614584088488889	0.0764604452802847
0.5653738294890628	0.5249398807165098	0.4575304104533483
0.6311615008486171	0.5079503231745829	0.4744966124488543
0.6294800382379394	0.4437638823434231	0.4324359001840717
0.6881194141345887	0.4254080396020384	0.4486320310255115
0.7491825289658289	0.4712947450015951	0.5072928162583025
0.7509708678727021	0.5351754037722652	0.5499149649153512
0.6921492566795564	0.5533408357053292	0.5336804725767624
0.5936943249887117	0.6132264649690994	0.4547726570984080
0.6801021838065121	0.7313556139708487	0.3797247333951903
0.3950022765531703	0.3801989936058036	0.4447861227073389
0.3084061707656109	0.3421881434099194	0.4263798503958930
0.2797863879075008	0.3586616501088319	0.3486693927505694
0.1998560807950094	0.3230435139486081	0.3313741682934880
0.1448534355126992	0.2684843369520824	0.3912821349696053
0.1729062762924400	0.2526243288710803	0.4702083711347338
0.2530782297181957	0.2890964741210388	0.4869284465807862
0.4281985301485852	0.4589590752629653	0.4937701408487720
0.5318168438015806	0.5970388051751038	0.2118371480145807
0.6782605900733412	0.6366439573947587	0.1522992934046685
0.6372497136388986	0.4918319047144266	0.2038290867574898
0.0647611593360089	0.2336830597821956	0.3740201802063934
0.4911071888348171	0.4495834305366272	0.1589146650223021
0.5847669790455395	0.5444149203222931	0.2056573012878005
0.6937456703734202	0.6032663030783860	0.5671991933745988
0.7984797896247632	0.5711345516397913	0.5957944327080161
0.7953305395172140	0.4573415868365721	0.5197377845950989
0.6862217758267848	0.3753301198968681	0.4152911016253097
0.5818133163389564	0.4080542549791464	0.3865277580047605
0.6326984736932475	0.7437097552271085	0.3658909555628425
0.7204273172721674	0.7485040052484194	0.3231842229635106
0.7110454516897941	0.7625773550030680	0.4404642556679119
0.4268202083972576	0.3887699274422950	0.3815624849957213
0.2732376186971164	0.2750530187412483	0.5483663509286852
0.1315205666864804	0.2109953760881260	0.5184280513623155
0.3210111754334558	0.3996721940867010	0.2998800418981949
0.1792918494681243	0.3368562676279577	0.2703113775943663
0.4071547530482998	0.3416093171721297	0.4865222167433564
0.0498758584901656	0.2313124592211042	0.3088275367353130
0.0301626139879290	0.1825844514857496	0.4063159847021957

0.3941773498756430 0.4518010433142787 0.5545982471743890
0.4248359887568847 0.5032259981722325 0.4518354290671143
0.5416225147289898 0.5026179195570379 0.3901045246567844
0.5699209489201601 0.6473689230206963 0.5014600132033442
0.6491920645083188 0.6482004796712504 0.3904309313306742
0.5069668302117246 0.4867879195891039 0.5237615582900770

Total energy (E): -1412.67526288 eV
Temperature (T): 333.0 K
Zero-point energy E_ZPE : 193.809 kcal/mol 8.404361 eV
Thermal correction to U(T): 210.560 kcal/mol 9.130769 eV
Thermal correction to H(T): 210.560 kcal/mol 9.130769 eV
Thermal correction to G(T): 177.832 kcal/mol 7.711514 eV
Entropy S : 411.223 J/(mol*K) 0.004262 eV/K
Entropy contribution T*S : 136937.193 J/(mol) 1.419254 eV

RhN4 SA (pyrrolic N): IS2
1.00000000000000
19.7297992706000009 0.0000000000000000 0.0000000000000000
-9.8648996353000005 17.0865073798999987 0.0000000000000000
0.0000000000000000 0.0000000000000000 15.0000000000000000
C N Rh H O
139 5 1 19 3
Direct
0.0055758553272454 0.0038389766865897 0.0858243993648853
0.0459591777570616 0.0864312950944355 0.0913241272535512
0.1301718201015145 0.0023591556203158 0.0907646623413944
0.1705939677493892 0.0850508415430518 0.0939537877123777
0.2551940489062074 0.0017931412189504 0.1010678739310615
0.2960934081349472 0.0849009917544668 0.1024141091474103
0.3802155654233657 0.0019505433402119 0.1115960999663150
0.4212677011907264 0.0856763574610534 0.1129680839286625
0.5053002475540082 0.0027650745700051 0.1164474284292690
0.5460460652869956 0.0868464601070510 0.1175197171975679
0.6301328046699062 0.0020355029996700 0.1141158114323069
0.6708624918917848 0.0861118694484600 0.1150109244096955
0.7547785137236657 0.0030832371054962 0.1049960048751566
0.7958141275263366 0.0867892578452867 0.1074311137042806
0.8799194806410737 0.0039045748836504 0.0916678507114074
0.9208517577972070 0.0869503500741058 0.0970041312633649
0.0040855240019752 0.1282826852599109 0.0961520881540733
0.0453904189290464 0.2113685366137743 0.1021966147309653
0.1286503717006332 0.1269017298754574 0.0940240957710415
0.1692670146543663 0.2096699279324982 0.0986806703084793

0.2533256183066966	0.1256185336175653	0.0987410517301044
0.2934551810182499	0.2079861507373497	0.1008134594096852
0.3792971423004082	0.1269162521981665	0.1089756050187154
0.4199131166783794	0.2101453001630968	0.1118950567715513
0.5043320938929946	0.1292488452310196	0.1176280150964649
0.5447951082331016	0.2138581280190884	0.1214584872731472
0.6291947118128870	0.1283708953418741	0.1179885077829853
0.6699492061005358	0.2122742169383526	0.1208009246305123
0.7540784397851077	0.1285781210483327	0.1132078489180087
0.7951005130567397	0.2122507639147291	0.1162807348871779
0.8790154634899038	0.1285535814440093	0.1045121111958580
0.9203500634589659	0.2118890885634737	0.1091616334503626
0.0037547107442258	0.2532324887366325	0.1079002210615172
0.0455254904336174	0.3364177279176295	0.1121725757097349
0.1285064006358863	0.2523684145320476	0.1026916311529643
0.1705541057856089	0.3356079634223438	0.1087467521928820
0.2516567708967484	0.2498335329664780	0.1006214320440271
0.2922455044694934	0.3323740648637394	0.1059861350873135
0.3759388105686821	0.2485257652699663	0.1066408756078984
0.4141791224634001	0.3296557740510014	0.1113752241071602
0.5028164045353887	0.2567193033879194	0.1208315698143038
0.5427195643618573	0.3429312122787835	0.1294174833834260
0.6279610584696360	0.2544080857797704	0.1238507265597139
0.6683063093758571	0.3378962346625786	0.1296511805226648
0.7532212914272006	0.2541963762829401	0.1206446241317023
0.7943960263011347	0.3382392319091458	0.1232197840028872
0.8786058710945175	0.2537665548545072	0.1145549550636761
0.9201809355095431	0.3373116411956671	0.1166942957863581
0.0038420888751912	0.3783094462239347	0.1151853981704447
0.0463814785145242	0.4615737075516551	0.1159521881372418
0.1292390016373989	0.3775156362096891	0.1129471215635891
0.1727638435246149	0.4605772648433885	0.1171438833914686
0.2537554944335632	0.3762905938960600	0.1110287200193285
0.3002566878261345	0.4591548554081806	0.1199634417559444
0.3733258419602146	0.3705907794205786	0.1109039991093277
0.4259924336717664	0.4504695993447243	0.1249618878431324
0.4939619487166113	0.3821420930660312	0.1255365092026693
0.6257548647971485	0.3789150727184676	0.1374378045432807
0.6720407625764886	0.4623213332147809	0.1581800521946401
0.7526569014849031	0.3802038903513209	0.1281934961625983
0.7949592805650354	0.4646481355803387	0.1257947639675872
0.8783657135563299	0.3793053293290242	0.1197936821928839
0.9203335925996211	0.4625828634939504	0.1178596621889487
0.0041846766115255	0.5033018874929800	0.1155507883699962

0.0457112972950283	0.5864546820926626	0.1129067522281433
0.1304312132620499	0.5023269060545444	0.1169155936450415
0.1720304054791908	0.5855217301218530	0.1172075143689367
0.2574045360626976	0.5011003430571424	0.1208874024069716
0.2979626349683464	0.5842588500202086	0.1237957013339215
0.3864626773464380	0.4991871448654895	0.1287627835740336
0.4221667276526535	0.5818476579806234	0.1377838183295985
0.7543224345439941	0.5076768995414606	0.1317689771142138
0.7900569190164758	0.5901727127741597	0.1182500876605864
0.8783286796702038	0.5047106872834166	0.1182303480307701
0.9188161171820948	0.5877657816725933	0.1121306780140335
0.0033961339934283	0.6281904029594904	0.1092436991250226
0.0469375970891180	0.7111868140974319	0.1035635949045775
0.1298174302135495	0.6272053488151710	0.1139189382514746
0.1723640077568819	0.7104506458159461	0.1119590761225785
0.2559131212982638	0.6263059811870328	0.1204511477894893
0.2978877102530808	0.7095911651375985	0.1203952095168032
0.3813864462302561	0.6245279632566001	0.1303679912262235
0.4238209768124708	0.7089537737903622	0.1294651143542176
0.5056753503028811	0.6279786930734098	0.1598912571572633
0.5513668163028635	0.7105299336608517	0.1327859032891249
0.6824011548039394	0.7067349856691951	0.1114059613158968
0.7508575447736996	0.6388229361982840	0.1111645302337872
0.8026700757058495	0.7179441925982136	0.0918739377826916
0.8759726358824478	0.6297034467051091	0.1079029006566604
0.9223813961156556	0.7123511296119700	0.0954738785696694
0.0057003762548380	0.7530001075981940	0.0955526224117711
0.0477347228465686	0.8363038115238334	0.0903015437937993
0.1306317848467818	0.7521925638878688	0.1059350677554005
0.1724036825236683	0.8353931875874091	0.1028312390313530
0.2560177519698437	0.7514783141878434	0.1153637299896371
0.2975620739127509	0.8350105373184945	0.1134922723457068
0.3819383621829712	0.7507182213164398	0.1237631986130176
0.4230116783836826	0.8347331942609664	0.1202255835323543
0.5083187070348659	0.7512013017456378	0.1274298804850568
0.5483779108655273	0.8345614867744606	0.1196711977688574
0.6335604585032665	0.7460180607084663	0.1185873053506293
0.6732672018380825	0.8320785812575711	0.1087254608761357
0.7616528604430310	0.7589012238122087	0.0921908405799275
0.7998197028869403	0.8400952662955290	0.0872314859684932
0.8839200457994959	0.7561792934593490	0.0866590081527260
0.9244829726550865	0.8388343903296570	0.0812547825541611
0.0069921974228846	0.8790858445837162	0.0839944597091738
0.0475384048014364	0.9619563615071595	0.0854149761302807

0.1308131342569326	0.8772491689615991	0.0953997530654035
0.1720989212252807	0.9604869872210132	0.0949136857820302
0.2557447767736169	0.8767558279302941	0.1076357123387936
0.2970788039830995	0.9601882422790033	0.1066705729298536
0.3810820437285586	0.8765930342420688	0.1163153495629055
0.4220566166950270	0.9602442656661153	0.1149636754824798
0.5062673672346296	0.8766152501965429	0.1189237616406259
0.5469943014376767	0.9604970784792857	0.1164884546612485
0.6313957419238863	0.8750047894500195	0.1133645267854824
0.6718264173275937	0.9596447725742728	0.1105835048913131
0.7559089097550532	0.8785156717594927	0.0964537585288929
0.7965876832653137	0.9618037285951967	0.0968487865069147
0.8825112960736776	0.8807337839365674	0.0816538281320917
0.9227229574623123	0.9632229765428939	0.0848446186713897
0.6227357956195860	0.5828524368370336	0.3455950162902916
0.6659772346173324	0.5614803056382731	0.4066899741869999
0.6620299380192838	0.4878767625911385	0.4036450107926476
0.7083960069255556	0.4716272902811950	0.4594850562895955
0.7599603409677744	0.5280620695519080	0.5197703729236577
0.7636102455049520	0.6006160925539531	0.5252614904033523
0.7165989234568009	0.6168498851430116	0.4704650025537203
0.6046821463815574	0.6434071575995273	0.3773839726906511
0.6343550992388126	0.7753394697175302	0.3670966674556022
0.1225432957686332	0.2181944860590295	0.4006256153467581
0.2059406394119868	0.2812432102169614	0.4182518646684322
0.2463307477078291	0.3439480725445044	0.3588584565210116
0.3240945836932237	0.4010786658933774	0.3727423832248785
0.3652155073138575	0.3976432227521811	0.4480708655522578
0.3249857612917218	0.3345402518905858	0.5077907946004874
0.2472768188912595	0.2779674155836508	0.4928005326070942
0.1183089360548116	0.1488218712965181	0.3492223915513701
0.5366753551455904	0.5984819275581478	0.2096676095823870
0.6751822906934527	0.6316112681218912	0.1214301214410678
0.6427638485112891	0.4939149413518999	0.2082603718858465
0.4427080000052683	0.4527384980220062	0.4618682113647776
0.5017173662442423	0.4583557463452404	0.1332666823393704
0.5917419192712412	0.5477041373379035	0.2242165922766088
0.7206200990547816	0.6739591136797999	0.4744855446703005
0.8035615275543979	0.6447238906921507	0.5722724810733569
0.7965282100972934	0.5152373482927060	0.5631269494455671
0.7045239906846578	0.4145501930583990	0.4561771369109947
0.6205904157637483	0.4434613835232560	0.3581683353061038
0.5750145296415083	0.7568786798028864	0.3431506314372274
0.6775647113201546	0.8279064349403424	0.3318147504522066

0.6370821026244916 0.7854888530728321 0.4393419981572056
0.0915464683943531 0.2416302629905900 0.3616429155308896
0.2176651920114820 0.2303634605119031 0.5409073458638061
0.3553535328589160 0.3307932269711890 0.5668917771254594
0.2158280650093616 0.3483171887472312 0.3002693511107142
0.3538380909611781 0.4490685973631011 0.3252423362595828
0.0907285764187042 0.1965656092404845 0.4642503552096072
0.4659683627780349 0.5065826589953398 0.4331171539504539
0.4653524357661008 0.4540666239913937 0.5228681570013529
0.1479631182632515 0.1233048360862134 0.3876891780951603
0.1493451456286176 0.1693315150299550 0.2853796043445965
0.0110683286036687 0.0680802513678961 0.3813164769975637
0.5479491151674274 0.6279569930662435 0.4250179696829843
0.6548616017036821 0.7160359049801074 0.3463650017655821
0.0398944351048998 0.0900181637707971 0.3262561841916526

Total energy (E): -1411.90329031 eV

Temperature (T): 333.0 K

Zero-point energy E_ZPE : 200.467 kcal/mol 8.693092 eV

Thermal correction to U(T): 216.469 kcal/mol 9.386985 eV

Thermal correction to H(T): 216.469 kcal/mol 9.386985 eV

Thermal correction to G(T): 184.376 kcal/mol 7.995319 eV

Entropy S : 403.229 J/(mol*K) 0.004179 eV/K

Entropy contribution T*S : 134275.358 J/(mol) 1.391666 eV

RhN_4 SA (pyrrolic N): TS2 (imaginary frequency: 36.572323 cm^{-1})

1.00000000000000
19.7297992706000009 0.0000000000000000 0.0000000000000000
-9.8648996353000005 17.0865073798999987 0.0000000000000000
0.0000000000000000 0.0000000000000000 15.0000000000000000
C N Rh H O
139 5 1 19 3
Direct
0.0030662706134440 0.0027690883795875 0.0785341597624273
0.0434661661695188 0.0854133751373882 0.0821901021660521
0.1277000876262472 0.0011711569038679 0.0819357459116977
0.1680850631989168 0.0838679781781713 0.0839047909747764
0.2526375964273878 0.0004475731256441 0.0934819365563008
0.2936005852723141 0.0835915728025980 0.0952094191501068
0.3775923160855225 0.0006104890389247 0.1071635998188948
0.4186097231882936 0.0842006890041421 0.1098795072784671
0.5026478380360584 0.0015359156849995 0.1148673812274658
0.5434660439485104 0.0854992740832300 0.1166552191057270
0.6276165052814596 0.0010126068478963 0.1136006278826962

0.6684053373741319	0.0849586189139026	0.1140052900792873
0.7523620496885224	0.0021981396276436	0.1033917033395313
0.7934012758452663	0.0857838427390411	0.1040860500262459
0.8774155302307860	0.0028798995118678	0.0873614837617590
0.9184692576916705	0.0860266599953224	0.0906473951764756
0.0015614917987797	0.1272741777281683	0.0879337933105198
0.0427292064365055	0.2102055306111041	0.0947659286825157
0.1261315303152192	0.1258018802947933	0.0838992611210101
0.1666543189268456	0.2084986455499715	0.0902259098965032
0.2508019999745119	0.1243516540712579	0.0900600851129523
0.2908098062638741	0.2066044218732999	0.0948748529514090
0.3765929713057528	0.1253431512233268	0.1050264589644029
0.4170715217567620	0.2084108732303967	0.1115661272058981
0.5015771300142690	0.1276439723254107	0.1167195727330091
0.5421334734580943	0.2120268588797871	0.1227047056660429
0.6266327531973297	0.1270291752659650	0.1175276840153518
0.6675108783014855	0.2108058325197983	0.1206751451853901
0.7516267911152393	0.1274260172333403	0.1107861372750409
0.7926802088261817	0.2110477465639093	0.1135418165707982
0.8765592471292262	0.1275282332280058	0.0991100266818655
0.9178327702469697	0.2107989043984202	0.1037066416240748
0.0011656025541394	0.2520735790660478	0.1021797989564714
0.0428612177067768	0.3351715787354715	0.1087185980100690
0.1258529608060429	0.2511978931262714	0.0960426527762310
0.1675830044061095	0.3341734088479717	0.1059285719905088
0.2489613248904627	0.2485483552034894	0.0949002229398992
0.2889351865451504	0.3305326973977885	0.1058942769059426
0.3727796567138617	0.2464943872773303	0.1058236088986628
0.4105074469399496	0.3270378150111831	0.1175609896804174
0.4997998116069853	0.2545096555121399	0.1242827588404609
0.5395280720630833	0.3399756889753340	0.1381531297729166
0.6254183391352418	0.2526505122734217	0.1251107109058454
0.6659712835296216	0.3360680731793604	0.1311215897281684
0.7508103524415271	0.2528478560247757	0.1193194841895263
0.7919664558263454	0.3367555200589556	0.1220817070819984
0.8760742092239555	0.2525535571180304	0.1107209568037186
0.9175862345524278	0.3359560915332719	0.1140742620414437
0.0012104665933951	0.3770122547773599	0.1130234463961355
0.0437005649191541	0.4602604437192123	0.1153681055419551
0.1263997192607259	0.3761976173504462	0.1110452584775783
0.1697425358245088	0.4591304948746224	0.1172825101869426
0.2506385232672962	0.3746783049597168	0.1119385791951230
0.2966243444623425	0.4573987868847494	0.1244822212669422
0.3695548796199419	0.3682643897954622	0.1175632560157339

0.4194975011564724	0.4466471489475689	0.1397144720182733
0.4888236169407483	0.3766435113437847	0.1399901900783900
0.6233546421485184	0.3766588584992308	0.1417215220926637
0.6705974827390971	0.4597140958529631	0.1608795915129496
0.7502621293226072	0.3786151832875203	0.1282056303195271
0.7927508414791745	0.4629480967903177	0.1269956991727593
0.8758189965810472	0.3778985045296439	0.1184304189634939
0.9178644489824791	0.4612204753156137	0.1177929394685942
0.0016354620457587	0.5020771966294421	0.1155492128743357
0.0431543571550721	0.5852106840745214	0.1128169107466485
0.1276359123567187	0.5010540352906764	0.1168724401650514
0.1691627626512636	0.5842603453159692	0.1170003782765077
0.2541516862085648	0.4997229475152035	0.1229072079561317
0.2946526188539783	0.5829709125959581	0.1249074092688358
0.3821479936127846	0.4972969955541570	0.1373861924343480
0.4182855015401346	0.5806982725048575	0.1401499229920089
0.7523457118863878	0.5059265515743214	0.1354413038681006
0.7887735711727875	0.5894559654483108	0.1263617418242276
0.8760332889572083	0.5033733049528820	0.1194246278391284
0.9165865167225603	0.5865551343132918	0.1138577017164931
0.0009840364715448	0.6270373249208259	0.1093341962397841
0.0444087882180734	0.7099612128289193	0.1018345918930427
0.1271222088640893	0.6260040106136303	0.1129851464907029
0.1696723400443843	0.7092187697111947	0.1092103576604673
0.2529245514550059	0.6251224703430192	0.1200721833246633
0.2950766989321054	0.7083916159853013	0.1182213716866889
0.3780226697696795	0.6234698079728438	0.1305866898078036
0.4208652236568113	0.7079338323231182	0.1276066597423437
0.5021064878229229	0.6277888771890964	0.1562152952871979
0.5484550531661059	0.7105923905065291	0.1340533376826991
0.6822102419294349	0.7092384238119420	0.1246244627137679
0.7521280873616070	0.6400369140002456	0.1246604614842715
0.8011008467581398	0.7175791846082276	0.0986285720694982
0.8740334859627641	0.6288115260103087	0.1118165797432241
0.9200353737396328	0.7113246520540450	0.0971514464844221
0.0032776058381581	0.7518825713021142	0.0939460443880113
0.0451643751312381	0.8350349252207386	0.0860452813050667
0.1280445966152187	0.7509866512078074	0.1022899300798041
0.1698386670994617	0.8341394576063952	0.0972191836087248
0.2533069309027213	0.7502481844244413	0.1119622455559147
0.2948967955794275	0.8337199351989684	0.1089297759853374
0.3790753519055487	0.7495920918427864	0.1212125826880026
0.4202725637061774	0.8335529241212933	0.1175877238128693
0.5053652788762045	0.7507362287827613	0.1267758498306693

0.5457839826246662	0.8339978696787326	0.1197318449633119
0.6316098443112466	0.7465239043792006	0.1258524354553777
0.6712117214602704	0.8319068681775983	0.1125857244694397
0.7598407409671466	0.7586050013149332	0.0988328936627765
0.7977207782447091	0.8394843521002675	0.0889718969422174
0.8818860782292036	0.7554533012444509	0.0883348259050442
0.9219723563458566	0.8377021022064736	0.0794755187346438
0.0044148415549718	0.8778618143013142	0.0794759647554623
0.0450261899413595	0.9607732546843581	0.0783123172341646
0.1283236504539519	0.8760512091429524	0.0891716051439021
0.1696158470933958	0.9592194219941075	0.0869802322941071
0.2531743367543835	0.8754670583335228	0.1017492989557589
0.2945108056865286	0.9588610960703610	0.1004111029295929
0.3783573647854023	0.8753107045773000	0.1127086454503405
0.4194046937182190	0.9589571725094245	0.1117820573530652
0.5035642284807522	0.8757199444357271	0.1176707535957950
0.5444319164168840	0.9594894231454455	0.1155973273089490
0.6289550973134453	0.8744404238584318	0.1146307676727348
0.6694855470170233	0.9588743856856909	0.1104197175185898
0.7537298643628488	0.8778250984918762	0.0982887540635905
0.7942866448523874	0.9610209906777920	0.0954179328947416
0.8799691536612620	0.8796431423175566	0.0798503677474657
0.9201620051076511	0.9621102872547089	0.0801874969007708
0.5854652759073969	0.5594541998204143	0.3916550614045908
0.6480451573484579	0.5534335096456573	0.4315115530284648
0.6500841664085806	0.4828747664491582	0.4200297666427538
0.7080832406709827	0.4727521453851899	0.4594001735062471
0.7646866038542084	0.5314443466321144	0.5140063705294790
0.7629133047305041	0.6008236007173617	0.5276298691686755
0.7064495613170373	0.6124244868719956	0.4864165824453724
0.5739688464726044	0.6273364939071793	0.3920759947896184
0.6360283760820806	0.7659591619675988	0.3750980004933516
0.1482544259180225	0.2418891367652102	0.3794856582525395
0.2286696982703370	0.2988425914653973	0.4135707976149445
0.2801320642147580	0.3669560123716821	0.3652980209942825
0.3546779066512415	0.4183855775219364	0.3960284744828467
0.3826034463773208	0.4032763702765532	0.4781184226428216
0.3295740554137792	0.3336052677736334	0.5261890124720997
0.2552785929178725	0.2835866558775545	0.4945898247384032
0.1457224693310670	0.1702040745378026	0.3349998358025268
0.5356895018690492	0.5967265595489426	0.1976589112071623
0.6787648360601212	0.6361842071050238	0.1445457428671384
0.6416588621671012	0.4918749175176287	0.2111207516858590
0.4551820910129704	0.4537653690193715	0.5053904397344909

0.4932775457886382 0.4506728765299488 0.1581720977943041
0.5891613042730958 0.5451522903256699 0.2033107239925372
0.7055778938881008 0.6664992656304844 0.4975035022366079
0.8059324479295025 0.6460928533719155 0.5713811676456564
0.8094878294007174 0.5229786498068443 0.5461912420148674
0.7094044064102936 0.4188080101543591 0.4479331562642916
0.6058705158788397 0.4369976704964997 0.3779566973336340
0.5813052596590510 0.7526902298224645 0.3415803383467178
0.6868639555071920 0.8081033432727290 0.3363945466301966
0.6370436336476450 0.7893219161897516 0.4419391855052902
0.1282316338913232 0.2698438328107866 0.3308850537291837
0.2158868028919549 0.2314796857144573 0.5330016734595315
0.3497012861798635 0.3215800939481924 0.5889139654645081
0.2599192569939475 0.3793833296876580 0.3028774203223356
0.3935681141170447 0.4721716374987397 0.3600431261032981
0.1063013762810179 0.2210352856683612 0.4354681501435176
0.5325489440168791 0.5049269682175420 0.3755994830286682
0.4667394146977817 0.4350617183088281 0.5645759434864084
0.1674315746373892 0.1422417803986610 0.3820413408798771
0.1844692087559229 0.1890259684791275 0.2765288262893940
0.0343473148253559 0.0929667511091450 0.3525794236153088
0.5099074758019916 0.6232710038465585 0.3965808158920742
0.6429551302187033 0.6968194614899120 0.3818776868811674
0.0696671988119211 0.1151293470649127 0.3020361149637547
Total energy (E): -1410.55024492 eV
Temperature (T): 333.0 K
Zero-point energy E_ZPE : 198.661 kcal/mol 8.614771 eV
Thermal correction to U(T): 215.086 kcal/mol 9.327033 eV
Thermal correction to H(T): 215.086 kcal/mol 9.327033 eV
Thermal correction to G(T): 180.930 kcal/mol 7.845889 eV
Entropy S : 429.155 J/(mol*K) 0.004448 eV/K
Entropy contribution T*S : 142908.570 J/(mol) 1.481143 eV

RhN4 SA (pyrrolic N): MS2
1.00000000000000
19.7297992706000009 0.0000000000000000 0.0000000000000000
-9.8648996353000005 17.0865073798999987 0.0000000000000000
0.0000000000000000 0.0000000000000000 15.0000000000000000
C N Rh H O
139 5 1 19 3
Direct
0.0000612652141919 0.0001643589678419 0.0719996172936122
0.0406183312560757 0.0829134417264700 0.0758329488827778

0.1247726895426298	0.9986105861961783	0.0747970449741863
0.1652425844999763	0.0813750054693054	0.0769751051358194
0.2497597341672767	0.9978854517975652	0.0871284831749531
0.2907379533338165	0.0810697084987323	0.0896880078145497
0.3747141947222846	0.9980460939405366	0.1019661451351627
0.4157040842788858	0.0815946463243983	0.1058203508858859
0.4997964332343516	0.9989401034583912	0.1105479550772476
0.5405843119544753	0.0828762932464913	0.1134087797763089
0.6247486666859254	0.9984835232122872	0.1095513247630933
0.6655729458169809	0.0824731461911243	0.1104057347886589
0.7494863715366925	0.9996134447752053	0.0990072499130094
0.7905916027268445	0.0832837242296912	0.0998040080230032
0.8744552204574635	0.0003178431716738	0.0817842404252455
0.9156241193710755	0.0835101849157379	0.0855340320076699
0.9987465562744902	0.1248574604654300	0.0823986433481208
0.0399959262984285	0.2078366185479467	0.0896453621025431
0.1233228773708147	0.1233733901292475	0.0774173417562527
0.1638005523036553	0.2060084400142780	0.0852551358749956
0.2479353341813386	0.1218547962736340	0.0845099746475821
0.2879321994817901	0.2040569264516447	0.0914564596027058
0.3736427151677836	0.1227566867520735	0.1011106364861153
0.4140897820128765	0.2057003293236382	0.1095262008026285
0.4985752001915030	0.1249023297801251	0.1139217866401735
0.5391469309458876	0.2092799073943573	0.1213937871465717
0.6238014773274280	0.1244808048656104	0.1150018827781799
0.6646660404187896	0.2082130006013488	0.1194973433207857
0.7488231076931591	0.1249424621297401	0.1071470822935177
0.7898160226592006	0.2085554107973964	0.1107226702836527
0.8737711855720244	0.1250315718782621	0.0947029654560056
0.9150540641547104	0.2083845769155814	0.0993771417358309
0.9984542429458670	0.2496997599031082	0.0975327440392637
0.0401793629511720	0.3327732900449851	0.1046524096105079
0.1231025744444499	0.2487997712733682	0.0914643353964927
0.1647801412681555	0.3316829374805089	0.1027392136798834
0.2459968084527911	0.2459886256452090	0.0916715334694517
0.2858024175588065	0.3278005580519516	0.1044187299470549
0.3697411380483075	0.2438551735695372	0.1042621686021865
0.4072551442500753	0.3242279291116765	0.1180392862106590
0.4967377179141470	0.2516547769418018	0.1234651924674253
0.5365758174675124	0.3371292554239434	0.1381407915080687
0.6224435801157514	0.2498858079477387	0.1245386011434093
0.6630098568053708	0.3332910641062818	0.1310471088259592
0.7479492241188007	0.2503001273546560	0.1179302401461223
0.7891855107195216	0.3343210876669082	0.1209927670787095

0.8732608912977652	0.2500931642464670	0.1070636069985611
0.9148773492976625	0.3335520633199135	0.1105000219439482
0.9985379986469344	0.3746038404174575	0.1090571109578045
0.0410556017479857	0.4578623400115654	0.1117426240364251
0.1237038290147954	0.3737888291642488	0.1079286925174591
0.1670234586633060	0.4567300542182770	0.1151395284919245
0.2477621056608329	0.3721852816008902	0.1100847691031139
0.2937462382599217	0.4548833582512944	0.1235877257081126
0.3661617374248594	0.3653227514744782	0.1178685692996863
0.4157523589745367	0.4435333750782703	0.1408827758375403
0.4854838588080002	0.3739864832205438	0.1410811855545274
0.6202242406136672	0.3735790923108259	0.1411939091060743
0.6677412392454897	0.4574417241545773	0.1579708542571523
0.7475603852429203	0.3761908142334373	0.1278637444040531
0.7903706045500916	0.4607289725267342	0.1265133169359394
0.8731234375937629	0.3754737746578221	0.1160936511600952
0.9152232186402786	0.4587577907271498	0.1153758887263125
0.9989729822621850	0.4996072715786054	0.1119453884832783
0.0405215516167120	0.5827405876501754	0.1087482499374641
0.1249909046802169	0.4987292044651257	0.1141727398304663
0.1665710729042701	0.5819213556024327	0.1145547767807351
0.2513859304870242	0.4973149501325940	0.1217223345634109
0.2919821072348014	0.5806088994992491	0.1240610153516842
0.3791710838082195	0.4946066163744099	0.1380869759869993
0.4156885793759890	0.5783923003512434	0.1411928905008248
0.7503251328063465	0.5039559725361230	0.1344214602054260
0.7863418209193890	0.5871844274984201	0.1252315810468142
0.8735246660781185	0.5009818233979714	0.1180645832672269
0.9139176695510566	0.5840820037460371	0.1114225067689682
0.9982901837545181	0.6245166386744391	0.1053599076069067
0.0415085163783953	0.7073457944124316	0.0970670576852585
0.1245004592643726	0.6236101555948154	0.1091881233962003
0.1669143539402116	0.7067912547051634	0.1049802357787103
0.2502913009072320	0.6227950183704520	0.1186425290184451
0.2923164968772258	0.7060451625288415	0.1163807627017407
0.3753551571923941	0.6211291981375856	0.1303769300896866
0.4181100569069998	0.7055432307746160	0.1270535461401934
0.4990692240844893	0.6258023276986612	0.1595369322910733
0.5456691997570878	0.7079649593905732	0.1348242214573065
0.6800838508892929	0.7076929004906667	0.1245056052280081
0.7493783338049420	0.6380921701879655	0.1242383946298782
0.7979487844194949	0.7149328262487806	0.0951481774386208
0.8714260585952102	0.6264247244218991	0.1095115603720549
0.9170846623871444	0.7086873461817271	0.0929552983181025

0.0002657205909417	0.7492217245015204	0.0888652385945649
0.0421180667622027	0.8323251537085085	0.0800993642658513
0.1251814269137278	0.7484687452417854	0.0973464696592051
0.1669221444052417	0.8316294569574243	0.0919071751758108
0.2505208751185674	0.7478181110031157	0.1084906737344083
0.2920639606822491	0.8311865841853462	0.1043959312713797
0.3762720267668788	0.7471991010799583	0.1196864883896982
0.4174286721271327	0.8310480427811003	0.1146598321529630
0.5025624615506895	0.7481743811421295	0.1261943360906888
0.5429045068305468	0.8313675327327622	0.1179159832117577
0.6291193710909343	0.7442301095823086	0.1257421746977744
0.6683961947001702	0.8293920472855475	0.1101489721315170
0.7568751016310937	0.7561698311877669	0.0953452793493623
0.7946215878149722	0.8368793832252962	0.0837185630692560
0.8786481088686308	0.7525989213217021	0.0831703800270602
0.9187304097971053	0.8348022753367816	0.0729645911642739
0.0012823251053163	0.8750481496253859	0.0729580615356279
0.0420092337900136	0.9580606168485736	0.0715177164949670
0.1253334309133051	0.8734682588263575	0.0832102972538603
0.1667131843005820	0.9566296502540433	0.0802785097152562
0.2502922514106817	0.8729165485421494	0.0963566126465740
0.2916269330545113	0.9562870844316693	0.0945572009035282
0.3754957940573575	0.8727770676080276	0.1082802668433081
0.4165494832444877	0.9564202143602500	0.1068397651132615
0.5007185029194453	0.8731126171682251	0.1147033266295782
0.5415810321559118	0.9568667575543279	0.1115843936272226
0.6260777730089683	0.8718654172075451	0.1120611297055598
0.6665822311534930	0.9562653583963417	0.1066355928340187
0.7507394622244484	0.8752208539822613	0.0939462087617909
0.7913633490591800	0.9584447812150068	0.0905219595391073
0.8767753724997477	0.8769160623883440	0.0733362489321944
0.9170758725450491	0.9594623844206163	0.0738311904162223
0.5507884125851423	0.5337901357128447	0.4517317801844781
0.6276882294331674	0.5373020694331709	0.4737895716204173
0.6432827051687624	0.4820269792156442	0.4345119537044638
0.7129593119719456	0.4827315487033334	0.4525849591895978
0.7678948745522028	0.5387390288297919	0.5106331712914158
0.7523453884532717	0.5933733202538036	0.5508960281465148
0.6824614521685004	0.5926332596255451	0.5326638088739877
0.5562086239708561	0.6145165014823673	0.4548283489579564
0.6165231899109161	0.7386835954363601	0.3840696652206906
0.1773304558875629	0.2672483428577631	0.3776341964796972
0.2575096411490124	0.3245029317212559	0.4132529571476438
0.3095623083713064	0.3908595237532550	0.3644394208863570

0.3852840182900369	0.4424442257225639	0.3941864344940200
0.4124139154773047	0.4281987505873577	0.4750327553356480
0.3596811598741621	0.3623785854403699	0.5256862398842075
0.2842484787042932	0.3120507815872753	0.4951363954745235
0.1745622127413788	0.1945155965608734	0.3368156961397799
0.5316776205373087	0.5959160305669079	0.2064778843692270
0.6771036454425182	0.6352387843814699	0.1467449578305168
0.6370763063083640	0.4909442462837439	0.2008738591892482
0.4879593732987825	0.4758909874256586	0.5054652405965785
0.4894070782269612	0.4475876261060657	0.1601146006883069
0.5848066539423321	0.5436920879868781	0.2015484269802144
0.6711335238350375	0.6360715367159895	0.5637149815549157
0.7950287125756993	0.6371994322487383	0.5962016138699813
0.8228132019713973	0.5399332012984611	0.5241315180387706
0.7246571751082082	0.4399251844900793	0.4206744730019781
0.6006225480266556	0.4388126641189697	0.3886171512876613
0.5612900391372412	0.7314696079749363	0.3579236312779140
0.6638365723150702	0.7700466322575426	0.3359391865003595
0.6299236395698385	0.7700382793683925	0.4478843169285864
0.1598148784404966	0.2951877842837350	0.3260560569463297
0.2451208855526995	0.2616085287234978	0.5360714097911001
0.3787808276309224	0.3509176522061933	0.5894932648745336
0.2903128668439288	0.4033221760319959	0.3013858752530766
0.4226439268299538	0.4941377790426554	0.3543832422099011
0.1333460364459422	0.2482939629484304	0.4314500770592020
0.5383052808534465	0.5159348736260077	0.3807938902563541
0.5035027015869828	0.4601982676399083	0.5627848229881625
0.1910420488392620	0.1647027040376370	0.3875910644997475
0.2172756965315381	0.2120593070663651	0.2822198562214719
0.0607250829954625	0.1231845833932416	0.3441512386470877
0.5166559217004010	0.6321526002516222	0.5010155545359327
0.6104716676441962	0.6630887836307401	0.3950554056716491
0.1002129015262433	0.1411727646398305	0.2974807975758893

Total energy (E): -1412.8315 eV

Temperature (T): 333.0 K

Zero-point energy E_ZPE : 201.923 kcal/mol 8.756216 eV

Thermal correction to U(T): 217.457 kcal/mol 9.429844 eV

Thermal correction to H(T): 217.457 kcal/mol 9.429844 eV

Thermal correction to G(T): 186.095 kcal/mol 8.069836 eV

Entropy S : 394.056 J/(mol*K) 0.004084 eV/K

Entropy contribution T*S : 131220.734 J/(mol) 1.360007 eV

RhN4 SA (pyrrolic N): IS3

```
1.00000000000000
   19.7297992706000009    0.0000000000000000    0.0000000000000000
   -9.8648996353000005   17.0865073798999987    0.0000000000000000
    0.0000000000000000    0.0000000000000000   15.0000000000000000
   C    N    Rh   H    O
   148     5     1    27     5
Direct
  0.0384442213698680  0.0161671168601724  0.1263438831867496
  0.0791171263504744  0.0990453790457777  0.1240683599120955
  0.1632085748497855  0.0149194567704196  0.1261249050116370
  0.2038819753103104  0.0977476278096896  0.1216386709289961
  0.2882600602494690  0.0143377896782011  0.1336992477190032
  0.3293939274189666  0.0975589817520298  0.1296295715884016
  0.4131716567017388  0.0144492952977617  0.1463069471516050
  0.4542788054057623  0.0981540808705808  0.1453932961697526
  0.5381395622855145  0.0151264496152267  0.1548583663964732
  0.5789717679785760  0.0991446307786274  0.1549475005095375
  0.6630546320532962  0.0143822350930446  0.1553453721465971
  0.7039010569329256  0.0984268765540094  0.1549110378492236
  0.7877417442081672  0.0154469680063990  0.1464572882471186
  0.8289297669821197  0.0991594588208814  0.1461519709108295
  0.9127579601256079  0.0161983696398119  0.1333815028225849
  0.9539148045116671  0.0994180599761479  0.1331479156875061
  0.0371362161632785  0.1408929875098096  0.1277853530596457
  0.0785680062641686  0.2241290419693812  0.1293713887465876
  0.1619891355500951  0.1396994744451378  0.1205406630989198
  0.2027850570146262  0.2227006990196461  0.1199987109485876
  0.2869070188057974  0.1385257185036532  0.1220996682056695
  0.3274335449833386  0.2211986882916500  0.1194534994997392
  0.4125782152128047  0.1395323151492102  0.1368791658454722
  0.4533805500457120  0.2228989481664012  0.1379127269441288
  0.5372447389242164  0.1414921573314764  0.1521471502925064
  0.5777579806351760  0.2259980032062668  0.1558798807758552
  0.6621502328563684  0.1406308583967067  0.1565991632349469
  0.7029532817008879  0.2244715377229551  0.1587861236569505
  0.7871618787783929  0.1408588381813869  0.1518066414445655
  0.8282189463509733  0.2245210822137662  0.1533355920607485
  0.9120845254876996  0.1410071944641140  0.1401885959514655
  0.9535737745110139  0.2244402312139984  0.1421590802558680
  0.0369477254226171  0.2658606050936617  0.1372028164050956
  0.0787535574006330  0.3490072952569360  0.1419738005391882
  0.1617993883787636  0.2652437982981565  0.1258497931553714
  0.2038161836218721  0.3484272180128300  0.1328323296418523
  0.2854650474280188  0.2631312503871912  0.1186671151819690
```

0.3259741570745415	0.3457174770084365	0.1258956268913904
0.4100533363123818	0.2616838153456650	0.1266953076207273
0.4485191220127046	0.3430950733587625	0.1341089394220420
0.5357770546369174	0.2688173346144164	0.1525354936115536
0.5753898951747957	0.3543672577762131	0.1649119840677379
0.6608636170401229	0.2665259854136423	0.1608977922802796
0.7011310639554929	0.3499403966966300	0.1671385250451487
0.7862653115580726	0.2663618952725823	0.1582728103258533
0.8273885243294370	0.3502968519305786	0.1609701621487341
0.9117543108245532	0.2661361976818366	0.1495053314703464
0.9533277115834989	0.3496543581338678	0.1521230278907631
0.0369988235138769	0.3906644252134989	0.1491833938403620
0.0794721774499862	0.4738949624315980	0.1528378199856137
0.1624664016511933	0.3901021536451907	0.1410490258291260
0.2058332777223185	0.4729571846690555	0.1491153732745257
0.2872104878026122	0.3891773551241539	0.1352117647638346
0.3332190993856180	0.4715573435717846	0.1500051882704350
0.4073458068109612	0.3840199926235293	0.1345524496549478
0.4570649094774189	0.4617722334051474	0.1611020123700757
0.5263151277256846	0.3929051016200727	0.1607094787346059
0.6581600457049253	0.3907889446955172	0.1744411778406233
0.7043979699379683	0.4734831159523158	0.1988026171863840
0.7854818701273382	0.3920407282300112	0.1666867975854917
0.8280843847515497	0.4763630790377158	0.1668662762553239
0.9113949365116866	0.3914743553499911	0.1574690985135855
0.9534404602528498	0.4747673814079584	0.1579186402703261
0.0372715896041749	0.5155895705738425	0.1557736152463436
0.0787028252627025	0.5988007226000558	0.1555380271873385
0.1634988086058760	0.5146771622479541	0.1530644598471345
0.2049494063078353	0.5978229600243942	0.1560185675689585
0.2903458689906950	0.5134500900089090	0.1535117165639893
0.3308372730196573	0.5965079914413024	0.1599384950429218
0.4186582848559824	0.5109599867853390	0.1640550396694876
0.4551419896490678	0.5938173955891972	0.1742441756097923
0.7874090818857890	0.5188314159101666	0.1751211938013510
0.8226175963596510	0.6016222044944155	0.1672409012237100
0.9114787121048789	0.5167876674061676	0.1606994066281255
0.9517639950014678	0.5999432660159311	0.1580253917003614
0.0363780238506837	0.6405865792481322	0.1545761891071741
0.0797701814690183	0.7236137370998097	0.1494865812662303
0.1627272060334680	0.6395940211325273	0.1552471414959034
0.2051360091639634	0.7228703064232050	0.1532726510320056
0.2887881614773653	0.6386212200173242	0.1585671044987930
0.3306318428703492	0.7219267694284520	0.1590968545526768

0.4141939599628426	0.6367698909556172	0.1667769188989326
0.4562293614280137	0.7210952016563935	0.1671263378321616
0.5375112602366408	0.6399550464474263	0.1992838331456422
0.5829146547700379	0.7229192210641729	0.1756821853858267
0.7144093544371002	0.7187654100775132	0.1649727109314301
0.7826776290897134	0.6502116153692313	0.1659719201871981
0.8353460283365189	0.7303106467652706	0.1515769446364692
0.9087770423694254	0.6418452090708334	0.1575317768571514
0.9551482895765260	0.7247629724899446	0.1483036998707271
0.0384083344468299	0.7655011740495384	0.1446920191984324
0.0804292823880097	0.8487228153089387	0.1374598056443586
0.1634493596009282	0.7647168914452093	0.1488181081228920
0.2052475423269759	0.8479092960888295	0.1437420155727974
0.2887880645140725	0.7639088335610973	0.1548975827513244
0.3303596690083852	0.8474242160068084	0.1520564095736786
0.4145004198431528	0.7630521085932102	0.1619605255881262
0.4556737686527539	0.8470310301899566	0.1590599164640645
0.5405162653464156	0.7637090952402386	0.1673177212793859
0.5809774742568931	0.8471037343623430	0.1609250483241473
0.6658420308789247	0.7584157263621004	0.1665487878046861
0.7059299317325790	0.8444451068778064	0.1552134112466798
0.7944572832881446	0.7712433198408395	0.1505435533331416
0.8327182860464114	0.8522821959833543	0.1415565951077357
0.9164643938629040	0.7686233483234619	0.1439169556722267
0.9571043298930622	0.8511058790452767	0.1356120613871127
0.0396159306691442	0.8914013391048545	0.1326193809743113
0.0803443852798528	0.9742437630825406	0.1274774404118923
0.1636153636792455	0.8897771200851258	0.1371477046796806
0.2050185829261988	0.9729483526379684	0.1314408555826161
0.2886289919680083	0.8892909938541483	0.1457175401842453
0.3300058525254058	0.9726781571643616	0.1416864362296882
0.4138452845950771	0.8889691341551458	0.1545516450275042
0.4548872911008092	0.9726498677331445	0.1522606852239671
0.5389674367713079	0.8890797201833959	0.1589737934683858
0.5798487801109450	0.9729274479227524	0.1567868611142602
0.6641223023534454	0.8874387713737216	0.1567668008745259
0.7047818075574818	0.9720474909442447	0.1530443477333781
0.7887346523218218	0.8908213874058710	0.1444273987274257
0.8294998492374983	0.9740968351674670	0.1403480702221067
0.9152727646834844	0.8929170589686464	0.1343207441122020
0.9555225620750012	0.9753906835936896	0.1301349050071405
0.5627739140485021	0.4917710061731847	0.3780475602508010
0.5123330641165119	0.4136740608745708	0.4087886481786616
0.4537762638908535	0.3545873199510984	0.3546529263198005

0.4107176848915300	0.2775803440090472	0.3839605626694103
0.4229660480969951	0.2570105151717793	0.4692007108315878
0.4782837688847124	0.3150427824544680	0.5255357132397096
0.5223735817359000	0.3919086328600833	0.4964256725623366
0.6083415501950163	0.5568751625360665	0.4372741269242563
0.7062958574202508	0.6942085305938119	0.4043910323610592
0.3231991810645526	0.4414852970948048	0.3746290466510089
0.2421549900511818	0.3708392688402237	0.3709268554817949
0.1751336875938971	0.3785114413245829	0.3723918176011305
0.1002274474374793	0.3136624367881966	0.3682437018575544
0.0889210827479235	0.2373562115287084	0.3625375068818454
0.1557096397068168	0.2289180933667206	0.3605439174771241
0.2303609206269530	0.2947894521312023	0.3649215614203378
0.9931935695872712	0.0941602801437546	0.3734607858230023
0.9050468615391974	0.0398530382578487	0.3810549719893979
0.8553271815804230	0.0650024228056276	0.4157339470939757
0.7748715022377374	0.0132814251402722	0.4226631388854883
0.7431894577099252	0.9352501866565677	0.3967306108463680
0.7927087460038927	0.9093575832597987	0.3643290236405892
0.8729016310256210	0.9612215798019208	0.3563051647527409
0.0300869693449509	0.0818623770595210	0.4587389407213558
0.0607263069712509	0.1155566769816371	0.6101850506270096
0.3616068327974530	0.4581260632167820	0.4666220686268050
0.5698613953700049	0.6129913002602692	0.2516017862395015
0.7064187284084023	0.6423821619346645	0.1745903956303503
0.6776782581850004	0.5064229066798812	0.2499585081261950
0.0134898583934660	0.1740915911533097	0.3569903807279555
0.5315913844033698	0.4670024967011680	0.1787114150869478
0.6097036636910668	0.5454697347283298	0.2645410113570829
0.5659148236765902	0.4362841695755777	0.5403579086279263
0.4876482918458773	0.2998936862774873	0.5926091150714152
0.3894594861608613	0.1964984620465384	0.4918709932694695
0.3670163719923967	0.2332842341394323	0.3398599694451640
0.4410947294718902	0.3703225608760296	0.2901544771011064
0.6654319080037852	0.7131580155669395	0.3818033013744958
0.7611888690587683	0.7241965984678127	0.3669899347238403
0.7167723795147071	0.7035398002437507	0.4761876480921304
0.3214592638005550	0.4939963957051373	0.3532951528878212
0.2810753097162980	0.2865953379262573	0.3626779319777856
0.1498419557323852	0.1711480007429723	0.3541787787565988
0.1818787557499430	0.4368968292839714	0.3758490370364698
0.0492625667326029	0.3216729169026368	0.3687142597718696
0.9110823480466737	0.9407972676811798	0.3298144602927127
0.7685634432458363	0.8488055375239177	0.3430492377485092

0.6803152280864790 0.8949095045942764 0.4015147877255053
0.7370045448843039 0.0341349600191463 0.4489147434805752
0.8788379792517050 0.1249718215742808 0.4393580821700844
0.1224396436469498 0.1328085040897837 0.6004930568054421
0.0282237997121666 0.0544377189532660 0.6326407947734646
0.0550819348016539 0.1542721022663885 0.6580530832316426
0.3622920842943210 0.4346409536609954 0.3278073326770551
0.0149355753242623 0.0722481868556140 0.3191946364968051
0.4710114835824792 0.5316634927289416 0.5050116442685647
0.9699333944550520 0.1865677393649342 0.3645026619871158
0.3709286885150651 0.4095277734351129 0.4867911558503268
0.3228410332940226 0.4625750937291452 0.5167092438639370
0.5980660101838928 0.5718520090470911 0.5134447150046340
0.6735246009967821 0.6116137515011469 0.3850727357207525
0.4343632748450938 0.5303799035941632 0.4605054174918058
0.0555040153298297 0.0368561446385723 0.4626779774390599
0.0280098961491252 0.1246903595685406 0.5277369245594431

Total energy (E): -1536.82816666 eV
Temperature (T): 333.0 K
Zero-point energy E_ZPE : 293.851 kcal/mol 12.742575 eV
Thermal correction to U(T): 318.091 kcal/mol 13.793720 eV
Thermal correction to H(T): 318.091 kcal/mol 13.793720 eV
Thermal correction to G(T): 269.875 kcal/mol 11.702912 eV
Entropy S : 605.802 J/(mol*K) 0.006279 eV/K
Entropy contribution T*S : 201732.219 J/(mol) 2.090808 eV

RhN4 SA (pyrrolic N): TS3 (imaginary frequency: 475.991953 cm^{-1})
1.00000000000000
19.7297992706000009 0.0000000000000000 0.0000000000000000
-9.8648996353000005 17.0865073798999987 0.0000000000000000
0.0000000000000000 0.0000000000000000 15.0000000000000000
C N Rh H O
148 5 1 27 5
Direct
0.0266917556352883 0.0165224642233625 0.1149839967502077
0.0674481503636517 0.0994859497376750 0.1139214139212146
0.1515842710658305 0.0152995848311617 0.1154863492455562
0.1923528270270571 0.0982281376956025 0.1131620712211172
0.2766450708237077 0.0146731916771473 0.1257027445354032
0.3178520513010714 0.0980358754478649 0.1244684236808890
0.4014923259999680 0.0147371555308313 0.1406667049763932
0.4426060718669336 0.0984769670619832 0.1419988757664762
0.5264296589255786 0.0153894669743366 0.1510298925863137

0.5672421124244841	0.0994144804197579	0.1523360783649110
0.6513459405010380	0.0146886850015949	0.1516060477606650
0.6921658736271198	0.0987273696990735	0.1514469636988929
0.7759795690146776	0.0157813749859234	0.1408563816893969
0.8171670470652432	0.0995076759252784	0.1405120577353470
0.9009109945599374	0.0165018944291175	0.1244475316051474
0.9421613541574176	0.0998226743767649	0.1247201286285606
0.0253835146915299	0.1413467666739709	0.1190970859465472
0.0669062987472296	0.2245643399723425	0.1231881624895086
0.1504263038275104	0.1402797621518665	0.1121508471550511
0.1913159864657203	0.2233232018867978	0.1150907128683558
0.2753864545760443	0.1390731669908535	0.1172901152688587
0.3158943879898339	0.2217670615054163	0.1194635063855912
0.4009153823453909	0.1399526337518771	0.1345286672981086
0.4416197836234300	0.2232672215704744	0.1395382728662263
0.5254677207014072	0.1417578681409040	0.1504472887699841
0.5660037564865289	0.2262557543563880	0.1565714685930316
0.6504147824646549	0.1409164637931120	0.1543256238036328
0.6912740273421972	0.2247840793135229	0.1579866968615131
0.7754428289729263	0.1412046472327689	0.1477540991673380
0.8164855550374723	0.2248711194055226	0.1502817967263391
0.9003144583395999	0.1413443632416588	0.1336707915538434
0.9417830973034153	0.2247670636173676	0.1370724056642023
0.0252245751809018	0.2662257741421040	0.1325680655531326
0.0670020656396648	0.3493284382496603	0.1391350741076196
0.1502699768479821	0.2658012573041631	0.1214923685642813
0.1922400960885036	0.3488470588528155	0.1315068455091932
0.2739965766160973	0.2637978391744159	0.1182510913165252
0.3143255681592177	0.3459693787479616	0.1293360417189617
0.3980728254478061	0.2620371506944629	0.1309141968129197
0.4363299036594874	0.3431214958042909	0.1422354510750123
0.5240323507050535	0.2691137687941852	0.1550039599921473
0.5638767736019866	0.3546907474659431	0.1694774132184022
0.6491767680790287	0.2668059509102166	0.1620370641230191
0.6895479121882847	0.3501389478491547	0.1705440496525467
0.7745649231121527	0.2666928480958928	0.1570960216865614
0.8156739901628894	0.3506561863004487	0.1609787036955675
0.8999726215881337	0.2664502532236743	0.1458977239200537
0.9415019920289182	0.3499369599410792	0.1494243817892071
0.0251826775337581	0.3909663498633126	0.1464890040625614
0.0676115718199341	0.4742256700596885	0.1502499028449619
0.1507630417001768	0.3904543949310498	0.1396145131344971
0.1939905641165482	0.4732288252783521	0.1485911690211587
0.2755252825328764	0.3895098664675065	0.1370363518237861

0.3213185187530730	0.4717746245223590	0.1531620956298974
0.3953025493688070	0.3841050138496735	0.1421154900846522
0.4451126012788941	0.4620620748780605	0.1675081659974576
0.5143972582280335	0.3930208745992850	0.1673141052005770
0.6466939414511573	0.3908796342388148	0.1797396812513400
0.6930739094728343	0.4738547933402493	0.2043729610779164
0.7738799022738633	0.3923773783774856	0.1690530028537252
0.8161910715765414	0.4768013493527851	0.1689855933897247
0.8996146011111109	0.3917937379957765	0.1560753523182868
0.9415029135390566	0.4750618605541629	0.1562561929724816
0.0253809620461805	0.5159276320475484	0.1528883911416181
0.0668991344739069	0.5991250719606553	0.1513870605200919
0.1516389402786614	0.5150043587650395	0.1510603035823671
0.1931073018884143	0.5981574343919553	0.1531424983497320
0.2784760832950214	0.5137414754616075	0.1547896164803510
0.3189525120660784	0.5969004858436391	0.1605455430307710
0.4068149837336891	0.5115311220588987	0.1685471079453036
0.4429913610355286	0.5944350277374130	0.1780729029659518
0.7757314109861929	0.5196563438186498	0.1784098004072251
0.8113693331157340	0.6023492182197053	0.1678101519456675
0.8994966938901134	0.5171486051174012	0.1600296468047070
0.9400216263200418	0.6003396768186340	0.1554414361027958
0.0246185730738383	0.6409456816090665	0.1498811136486499
0.0680078539947616	0.7238447817932874	0.1425246708495561
0.1509285001075557	0.6399170465180616	0.1506797890985354
0.1934206530853728	0.7231871617386059	0.1471938901132820
0.2769636000438901	0.6390165781822393	0.1566440524034193
0.3188748560748345	0.7222928067072661	0.1558926526857504
0.4022609525851087	0.6372998666134777	0.1689260583283947
0.4445023121404749	0.7216039991703261	0.1675596027086537
0.5259624729469127	0.6408090748968079	0.2020150235255516
0.5717394491267713	0.7234933512913057	0.1766776210879041
0.7036808654272880	0.7202493223169674	0.1650131366878514
0.7724969070379123	0.6516203632832207	0.1660344383133495
0.8241732987257895	0.7310644541903678	0.1482473101810049
0.8972485759248722	0.6423761219038689	0.1549667873419798
0.9435113377148434	0.7251370001849755	0.1424717194393353
0.0267045692132476	0.7657847705981277	0.1367409575615873
0.0686896385767189	0.8489133491724730	0.1274039772295209
0.1517231024801787	0.7649647721436764	0.1410825694816826
0.1935386074829314	0.8481307513609301	0.1346863203086666
0.2770779249332924	0.7642319468702157	0.1493985685981251
0.3186512201388340	0.8477045365868942	0.1455319538736257
0.4028103468785538	0.7634207143042663	0.1598270587813314

0.4439633948543514	0.8473680349245948	0.1554849282693771
0.5289132822403537	0.7639578502078808	0.1675097912637820
0.5693021224796589	0.8472931826496348	0.1591105029112489
0.6545035192298462	0.7591573410376059	0.1662394039208944
0.6944565640867477	0.8449859494997587	0.1526979676080901
0.7831189340852134	0.7719263604092182	0.1472992717704572
0.8211797874868362	0.8528096545085782	0.1353637380320506
0.9051066405883662	0.7692048483398972	0.1374782652702162
0.9454121090531336	0.8513562680730862	0.1264149477401027
0.0278855287417711	0.8916359723307222	0.1218080815465494
0.0686651888088282	0.9745658972795921	0.1158411601858389
0.1519747194924047	0.8900402951934310	0.1269425520494755
0.1934317016518635	0.9732469870414897	0.1214279045119866
0.2769518305063867	0.8895283391211750	0.1375645336224093
0.3183767285987820	0.9729712508062340	0.1341196570334669
0.4021244049590148	0.8892514676765880	0.1492302676409763
0.4431736601487365	0.9729311723652388	0.1471269813071420
0.5272308808859567	0.8892950410104611	0.1559209401228572
0.5681391813810530	0.9731731062269368	0.1531867867168789
0.6524769167310550	0.8877988919244508	0.1540788764676241
0.6931333395138579	0.9724028688504102	0.1489950495894250
0.7770980289242737	0.8912313322214097	0.1392039255973175
0.8177731482862305	0.9744629145787879	0.1335208810116379
0.9034370135142802	0.8932122058503958	0.1252987915275241
0.9436724821231589	0.9756849408948554	0.1198208188191579
0.5517999323187728	0.5094050644417912	0.4106276867323070
0.5472604182353918	0.4381001235988834	0.4475114250945860
0.5255385418133900	0.3720447804188641	0.3936838057012053
0.5228806683171534	0.3051468262442298	0.4280830015524627
0.5393317198673281	0.3018338442488225	0.5180658363842623
0.5583661762570248	0.3661344012171853	0.5731901512427182
0.5632593051516485	0.4339806177357967	0.5385603777490443
0.6124791224155659	0.5859076895669502	0.4510599517428640
0.7351919129988215	0.7015016752696627	0.4108308841087433
0.3565626725083458	0.4245743225540435	0.3797039656713838
0.2737412853372073	0.3598574697364186	0.3688283066579350
0.2118220905303090	0.3757657202843205	0.3711103453595397
0.1344025752079173	0.3164660642528139	0.3644557157086174
0.1153768564406507	0.2376900003515463	0.3556103043242095
0.1770025245653404	0.2211283199741794	0.3517235505370663
0.2545110812874426	0.2816130082000948	0.3584338275139042
0.0096926752357293	0.0981921012751565	0.3655701768340167
0.9208846992580971	0.0506259282080036	0.3729280832225915
0.8765937107753730	0.0822337209413550	0.4074072174321083

0.7952964921847664	0.0363498437872972	0.4145232706302893
0.7573363440188668	0.9577681559240814	0.3890017168541719
0.8013741158595218	0.9254406436408293	0.3566377132131172
0.8824375522865036	0.9715232132071123	0.3483628949284632
0.0430852139523699	0.0814427284039759	0.4509974026532912
0.0760733868954827	0.1146836096541028	0.6022639912254208
0.3864219449381758	0.4394530332037723	0.4778763406802006
0.5565806035608727	0.6111434971773948	0.2527470553878094
0.6965351090155490	0.6442492449812394	0.1759990972578179
0.6634843559869015	0.5043655464422274	0.2553421244689454
0.0374906276324474	0.1799608680330454	0.3494734628646454
0.5197994502773911	0.4674099714985846	0.1852049838852788
0.6047091260034538	0.5524849550651889	0.2627725317372036
0.5764498502729651	0.4831416246068306	0.5823037272336814
0.5692467252169641	0.3633455100159806	0.6440643765059361
0.5370028527788446	0.2494266878226014	0.5452610576927388
0.5084967278998612	0.2558084731911616	0.3839316703230834
0.5131425089211206	0.3744407683578312	0.3235243078138249
0.7161992913725405	0.7450889316172677	0.4121111753277941
0.7794257914938677	0.7151645216339928	0.3593278194452817
0.7567660796024069	0.6976111492728003	0.4768989674944034
0.3637258168766892	0.4801242187244193	0.3543478227219926
0.3013974181046652	0.2674864101640038	0.3544786247481665
0.1648419359687132	0.1612622924163893	0.3423473995635018
0.2250244123036285	0.4363222291149992	0.3776720726990921
0.0874596587958895	0.3305941514236352	0.3659153162688007
0.9164413200557049	0.9462410353107926	0.3219031365998229
0.7723261475746395	0.8643977240699821	0.3356178647216878
0.6938315183740151	0.9219860018961188	0.3940914923601322
0.7617274098387672	0.0621946960214275	0.4406391233792988
0.9049390859780041	0.1427194208452793	0.4307663839518502
0.1361071701648423	0.1262671671663022	0.5927008415256823
0.0390076407847157	0.0541788650595301	0.6251955085453715
0.0748362767806636	0.1561734853512886	0.6496824587717097
0.3958231298346409	0.4112135454112526	0.3408119600833053
0.0280489015517300	0.0732189446611933	0.3111852614459989
0.5031726557281837	0.5081608533158434	0.3746514241529779
-0.0017665155531081	0.1980581374999370	0.3588818495330331
0.3898544003323026	0.3880832175151206	0.5029254239668007
0.3413123913203305	0.4428427753970011	0.5191822271933559
0.6181861013424890	0.6107647248380821	0.5258932034542579
0.6692411635247015	0.6255725726586040	0.3868223820673646
0.4548629478001031	0.5094963010316887	0.4892574262808086
0.0618109651528895	0.0312510082749423	0.4553291394758588

0.0460772061553081 0.1271728319979437 0.5195583823310609
Total energy (E): -1535.12267393 eV
Temperature (T): 333.0 K
Zero-point energy E_ZPE : 291.941 kcal/mol 12.659776 eV
Thermal correction to U(T): 315.204 kcal/mol 13.668539 eV
Thermal correction to H(T): 315.204 kcal/mol 13.668539 eV
Thermal correction to G(T): 269.049 kcal/mol 11.667080 eV
Entropy S : 579.914 J/(mol*K) 0.006010 eV/K
Entropy contribution T*S : 193111.417 J/(mol) 2.001459 eV

RhN4 SA (pyrrolic N): MS3
1.00000000000000
19.7297992706000009 0.0000000000000000 0.0000000000000000
-9.8648996353000005 17.0865073798999987 0.0000000000000000
0.0000000000000000 0.0000000000000000 15.0000000000000000
C N Rh H O
148 5 1 27 5
Direct
0.0208059342776660 0.0160824411784912 0.1078308856087424
0.0612569682967484 0.0988723469899919 0.1079319010398749
0.1455706506336016 0.0144908419292260 0.1090020604906352
0.1860372683719672 0.0972694360190265 0.1072996371405197
0.2705665123793008 0.0137724988210204 0.1202510263735859
0.3115748219267743 0.0969583876396217 0.1193129828221671
0.3954988149401669 0.0138459121199514 0.1363019588274460
0.4364345576977022 0.0974010625366220 0.1377453904707852
0.5204515621145608 0.0146038165518956 0.1476367548383863
0.5611908188865639 0.0985520576805970 0.1488548849110206
0.6454795037398647 0.0141559173718676 0.1492683321719062
0.6863054826561930 0.0981323477327283 0.1488309514671908
0.7703180127581899 0.0155683400513826 0.1380737368192065
0.8113708969967222 0.0991632028588233 0.1375578141094423
0.8951146170352445 0.0163100107197670 0.1187620988333084
0.9362022088709633 0.0994719746367237 0.1198990445567279
0.0192984690878246 0.1407850481694532 0.1140342145589501
0.0606872233051749 0.2239317358436438 0.1191218489966852
0.1440476426900818 0.1393245422520279 0.1065271697178121
0.1845906628147419 0.2221564708749579 0.1099410319276895
0.2688699410417337 0.1378170429490270 0.1118629017086477
0.3089306221442296 0.2202458073253316 0.1139614632672279
0.3945290650668664 0.1386238242398423 0.1299654159107040
0.4348826210778459 0.2216999026423026 0.1353947201056321
0.5192257457448014 0.1406420933493897 0.1464492079253730

0.5596546730361698	0.2250110136360754	0.1522875014709269
0.6444238716496017	0.1400988733085651	0.1511469791308308
0.6852392194219635	0.2238512437055302	0.1540594385047117
0.7695572142910568	0.1407270394792074	0.1450278235197281
0.8105499064037291	0.2243843684810420	0.1471658031031424
0.8944080924870726	0.1409431994817665	0.1300805725524902
0.9357800796231869	0.2242835832118500	0.1339389734826483
0.0191450075022408	0.2656691395896181	0.1293792435649737
0.0608990207827248	0.3487307543575242	0.1364250277925737
0.1438272575355205	0.2649624966453913	0.1170683038417340
0.1856237153854179	0.3479501440601042	0.1273595441517568
0.2669272956338223	0.2621488586175615	0.1126962331629680
0.3067111499450325	0.3441622637852845	0.1236358647738593
0.3909277275138189	0.2600929723104428	0.1260185765014596
0.4284474350126090	0.3407661736439714	0.1373847118477410
0.5172459476431059	0.2674846261781165	0.1511007455311335
0.5568142880999195	0.3528874114877114	0.1643678880885977
0.6428820569553434	0.2654538065159474	0.1571302997223115
0.6833279310654825	0.3488158949524975	0.1633336661374390
0.7685835052660290	0.2661208121784437	0.1531583393473047
0.8097842573355278	0.3501607274963850	0.1562743549822639
0.8940453092668568	0.2660001424323513	0.1429614675198155
0.9356219876360412	0.3494960542165915	0.1466759893044304
0.0192666540314519	0.3905042084121713	0.1440265348915018
0.0618125996479651	0.4737385906197277	0.1476165946635214
0.1445238756267536	0.3897425177243956	0.1363591145486506
0.1879196748460863	0.4726244229620826	0.1449764782769411
0.2687722875166141	0.3884385023903889	0.1321680651225872
0.3147255626344598	0.4707700118462088	0.1484722321757868
0.3870844723440173	0.3816504401322686	0.1360245084188400
0.4357256375790174	0.4591468821126156	0.1623664325573537
0.5059984299162463	0.3903155691013581	0.1635147440157947
0.6402235538855040	0.3889487677004154	0.1706574615647782
0.6868298207993107	0.4728966914652518	0.1863510399687723
0.7679660606729707	0.3919140821546739	0.1623347340775783
0.8110037727726430	0.4765511450587832	0.1633042876586357
0.8938200778361011	0.3914060238757187	0.1527853838630808
0.9360139500801734	0.4747266275514301	0.1535563038632589
0.0197828045699480	0.5155498841734651	0.1504054261664238
0.0613355958175748	0.5987776796665105	0.1484524073269406
0.1457883032024893	0.5145157212629529	0.1477301925497431
0.1873722547521616	0.5976563981544537	0.1491948018390266
0.2722476837736110	0.5130557795303046	0.1501461347349937
0.3130216988117325	0.5962909989629838	0.1548349620944247

0.3998599173310697	0.5100217548852645	0.1642397030048033
0.4370639036728075	0.5939682124857730	0.1730283091833361
0.7708279684016723	0.5195997482970514	0.1710864748466052
0.8069559593602882	0.6029887133167380	0.1658697589793514
0.8943566158346110	0.5170150033625430	0.1569687634898713
0.9348187185705509	0.6002527481527246	0.1526971962300313
0.0192243932539270	0.6407454278564869	0.1467340235785623
0.0625295546457560	0.7235590994934020	0.1384551991191411
0.1452926381370555	0.6395139921680036	0.1470021165134821
0.1877565510151570	0.7226985295418568	0.1427852221687021
0.2711691574243482	0.6384598942654907	0.1515336758443139
0.3131835029940333	0.7217867411322039	0.1505251419670763
0.3964405905805332	0.6367761306843970	0.1622378465130211
0.4388186723136211	0.7210351036592118	0.1614722486900941
0.5186364487257225	0.6408740217103580	0.1989605529622494
0.5658203297115489	0.7227826025889386	0.1744562743571476
0.7009487476736030	0.7243394929964395	0.1665776135727935
0.7695498496929527	0.6538972964005549	0.1664317563841435
0.8194098592046389	0.7317786007430032	0.1417254528506845
0.8923774328508260	0.6426721512893736	0.1525283125090250
0.9382854283970250	0.7251661140283718	0.1377174690661994
0.0213256994970948	0.7655258042579419	0.1315871082822243
0.0630589988902891	0.8484875879589983	0.1207716160391308
0.1460816716240577	0.7644953292854775	0.1365061892858811
0.1878105182310082	0.8475733426018069	0.1293777334170143
0.2713994880952621	0.7637066441745272	0.1444340944976127
0.3128873412070447	0.8470616578626858	0.1406265572657999
0.3970176646078578	0.7628786475026503	0.1542835552614909
0.4381288213263394	0.8467101617349004	0.1509333012183808
0.5230994685531085	0.7634239324133962	0.1631216123498427
0.5636214328435645	0.8468729817901115	0.1561796589830540
0.6499080159684779	0.7601248174848616	0.1672304813658182
0.6892334987791643	0.8452876525649798	0.1513423142076524
0.7786882208844452	0.7732578020363404	0.1412183809822204
0.8161132708854809	0.8535433435016464	0.1282790797721398
0.8999887009163827	0.7692678496068638	0.1297084060204877
0.9398828681625360	0.8512301194427356	0.1175384385642881
0.0222678925191053	0.8912515794557353	0.1139369399037907
0.0627902737373573	0.9740399495020359	0.1084778741620271
0.1462335058641070	0.8894587627609059	0.1208018252640102
0.1874626328465497	0.9725263334175570	0.1152189065297126
0.2711167972267836	0.8888345375168605	0.1324950144315700
0.3124093037019682	0.9721575560019885	0.1291416836074976
0.3962385727714133	0.8885283053920325	0.1446916862801298

0.4372766557208687	0.9721684782111820	0.1430650741422661
0.5214710790188594	0.8887137585708992	0.1524988210613491
0.5622970218001435	0.9725255185436601	0.1503507011417253
0.6468998158774754	0.8877234401519249	0.1521751206106620
0.6874163501752953	0.9720738753347706	0.1468453410619832
0.7716711567692983	0.8913990585254191	0.1353866138630598
0.8121366464589326	0.9745101979552258	0.1294304886465610
0.8980746150108586	0.8932945029015860	0.1166845930859709
0.9379822489965917	0.9755990867406630	0.1123612921681688
0.5391273785789583	0.5083051684230069	0.4616501620565396
0.5598503503545630	0.4472612045539641	0.4916417073666073
0.5707215120154099	0.4009648241758605	0.4290064469954128
0.5895903493877404	0.3447878829983526	0.4567113387223135
0.5974940646583352	0.3344951079989960	0.5475191963798239
0.5863997534306901	0.3804622025009727	0.6104322279468468
0.5676690306345979	0.4366407229160109	0.5827121612250186
0.6062134357067663	0.5909478327852086	0.4827296068437812
0.7327830024909264	0.6874719826300576	0.4339414041913756
0.3778859129191972	0.4183018094331777	0.3839044870107851
0.2932809072431548	0.3568707630105457	0.3702704155569236
0.2342611075122896	0.3769516310579242	0.3730482708927388
0.1556279776751802	0.3207793555456730	0.3648162906445847
0.1322089842077604	0.2410579834497078	0.3536754076271466
0.1909142473726186	0.2204400973125061	0.3490812753804934
0.2697159837852286	0.2777911152896161	0.3574642624483018
0.0209362912454178	0.1038216633727464	0.3616951161600319
0.9319580701382544	0.0599291435704459	0.3690739728069602
0.8908653275371664	0.0944099652409258	0.4051920514180660
0.8094594883483602	0.0515123500729195	0.4135173864950181
0.7681431381286364	0.9729911552330450	0.3877872160413701
0.8088806092604391	0.9376514619625879	0.3538171006794130
0.8900459136243849	0.9809221136308506	0.3440135334767833
0.0517463105108511	0.0838648129458282	0.4470092165528682
0.0832124167960333	0.1135534171341435	0.5991181738105082
0.4001777611109304	0.4293891666655575	0.4833625778584257
0.5474333029625417	0.6113251289698635	0.2553657095201022
0.6966917187651119	0.6511798882565698	0.1859720415472643
0.6521760009007659	0.5071220914895919	0.2169613275867281
0.0530175600823356	0.1864722187114381	0.3458006173630128
0.5086063005586433	0.4632169869834363	0.1831080878017145
0.5998140222275136	0.5589706386558093	0.2292245512723239
0.5586969388424053	0.4726280915973652	0.6312750771086423
0.5925478276794308	0.3725347091431521	0.6813885675700317
0.6120818799183065	0.2905949794058482	0.5694523085879287

0.5978414100334413	0.3089604675875540	0.4072438924882854
0.5646994711508365	0.4092561576953565	0.3580836085761869
0.7158422325868763	0.7325904174350950	0.4293537343928129
0.7723456947009155	0.6935847645863893	0.3793992967904148
0.7605011297849789	0.6921909272119654	0.4988595385832670
0.3889837673766105	0.4749890106110492	0.3573344477216508
0.3140442950357499	0.2601433939058727	0.3532520820598036
0.1756062764691938	0.1597475230312725	0.3383316513896943
0.2504904365946914	0.4382371178773783	0.3816810126823145
0.1111628235759460	0.3383422706915546	0.3670382958259835
0.9215140325851071	0.9532606112116451	0.3165993094792903
0.7771819703253154	0.8763333268794746	0.3336167901097386
0.7046052819676208	0.9394681858939096	0.3943512934458959
0.7784394960780243	0.0795216825879739	0.4411707783060554
0.9218545709970288	0.1547379922475965	0.4292079282200398
0.1419205611090102	0.1213176021614958	0.5904133159728415
0.0428928364552180	0.0533305680412145	0.6208337063897357
0.0842956529631234	0.1558438633904821	0.6470922278399952
0.4158065293777141	0.4020638370611842	0.3468375165203744
0.0372513000911283	0.0772191470187060	0.3071891577194849
0.5329395247744338	0.5051010961776030	0.3882200909724635
0.0165966133492156	0.2075025680467630	0.3588097351289578
0.4033961199110740	0.3783493239712054	0.5083913930960234
0.3551157264958438	0.4336673827439091	0.5219271023317359
0.6083876177876728	0.6329551088425838	0.5431564753142853
0.6650699457490624	0.6105715840753514	0.4228807436059574
0.4710172949461852	0.5008466488375519	0.5032280536766741
0.0672013460823459	0.0313136441538122	0.4508692656483572
0.0563626661105750	0.1297019385259016	0.5162719334485008

Total energy (E): -1537.41831376 eV
Temperature (T): 333.0 K
Zero-point energy E_ZPE : 295.332 kcal/mol 12.806790 eV
Thermal correction to U(T): 317.126 kcal/mol 13.751890 eV
Thermal correction to H(T): 317.126 kcal/mol 13.751890 eV
Thermal correction to G(T): 274.334 kcal/mol 11.896264 eV
Entropy S : 537.660 J/(mol*K) 0.005572 eV/K
Entropy contribution T*S : 179040.617 J/(mol) 1.855626 eV

RhN3P SA (pyridinic N): IS2
1.00000000000000

19.7297992706000009	0.0000000000000000	0.0000000000000000
-9.8648996353000005	17.0865073798999987	0.0000000000000000
0.0000000000000000	0.0000000000000000	15.0000000000000000

C P N Rh H O
139 1 4 1 19 3
Direct

0.9991542808415504	0.9990014297908608	0.1081753440763865
0.0414922464325506	0.0825815537159637	0.1045277651606294
0.1237208417755051	0.9986922228587400	0.1087750361835874
0.1659989401415305	0.0822750039303049	0.1075693298980696
0.2488761075068232	0.9988424597381841	0.1043745133243289
0.2908900336371145	0.0821290830464688	0.1058993202133598
0.3740169080422286	0.9990137869400926	0.0964845550479244
0.4160556446092388	0.0822502528107363	0.0976106574532974
0.4994158759279391	-0.0005878977768177	0.0897254972177213
0.5414873050348832	0.0825353400895310	0.0895815298157615
0.6249042881847116	0.0000122618497534	0.0878472340530254
0.6669724618841184	0.0831408101757657	0.0870952894334637
0.7501813756301604	0.0000770528598278	0.0932145333765777
0.7922222404961935	0.0832431084771537	0.0914442907542925
0.8749500278826724	-0.0004206160872185	0.1024149917003451
0.9171592500479612	0.0830529508237592	0.0988258141775285
0.0005347597694914	0.1247865220850387	0.1013160761542843
0.0427590729570343	0.2083374498106892	0.1020374800245308
0.1249060501816971	0.1244069822607440	0.1060342214857088
0.1672674100493357	0.2080379444770959	0.1079930566040187
0.2495298125339603	0.1240454491240365	0.1088778950234389
0.2917233790593185	0.2074257922216917	0.1145749407678368
0.3744265370837128	0.1238121381426131	0.1043486784890381
0.4164601266301917	0.2067051009608329	0.1114716267827992
0.4995252531428966	0.1236162830803494	0.0945584126053994
0.5416531289138221	0.2064859377596344	0.0997974102484290
0.6249991194895563	0.1242754083483828	0.0883201743256433
0.6671170552730928	0.2073448592536098	0.0924144311246562
0.7504861137740132	0.1247566679541143	0.0890775288481278
0.7924896364024410	0.2079595793160607	0.0919308722805557
0.8756942608030027	0.1248324049026466	0.0948064029546648
0.9176362319406772	0.2081926359789427	0.0961017736413536
0.0010755748503061	0.2499527920660790	0.1003351434994855
0.0429596371956091	0.3333285747817922	0.1040016599509337
0.1262688409481313	0.2503100547043298	0.1068035339997387
0.1685165378058635	0.3339482144651433	0.1120498172267705
0.2505952061126647	0.2496418856412080	0.1146947890372322
0.2925197873826744	0.3328763462495165	0.1239677485726665
0.3751935070038803	0.2488132217535687	0.1207337780580734
0.4169930956961792	0.3309140990587278	0.1368745710176474
0.4998282823853252	0.2472626742296797	0.1096600596108753

0.5422276044279070	0.3292403227062785	0.1221541863877526
0.6252318078493339	0.2482955809632565	0.0989672248193420
0.6673332382687366	0.3310329008178601	0.1081970409093814
0.7506009411519249	0.2492093016892125	0.0940992277990403
0.7924152829911885	0.3323215429014177	0.1008432581496039
0.8758888772800287	0.2497244987238017	0.0953747833134068
0.9176543000707598	0.3329559416964365	0.0998368754982289
0.0010516167978055	0.3747696817718370	0.1035631592515227
0.0425741045091276	0.4580787868514533	0.1075883926699587
0.1263848946110425	0.3751865606763179	0.1099715677494359
0.1680443292996763	0.4585350732966197	0.1137501868666923
0.2518870144649189	0.3759072547159955	0.1218487972097495
0.2938058315598924	0.4598065959425076	0.1287176633008051
0.3747338468337113	0.3732191729801118	0.1406136041753133
0.4123568834262513	0.4551295451310962	0.1608543897324351
0.5011884866193812	0.3692923568334741	0.1422262375868598
0.5461682089146659	0.4479082533711466	0.1678629362618168
0.6255422389804148	0.3712278342033495	0.1212538406243626
0.6684668806499054	0.4536891858481216	0.1342064957549065
0.7506552793756658	0.3733278621376609	0.1080421879928879
0.7924631640111711	0.4565138980022632	0.1161013976712111
0.8758299026914261	0.3743401791196497	0.1027672365560530
0.9173316176201718	0.4575615633563986	0.1083274295406463
0.0005844453056743	0.4994890724781291	0.1089543051896025
0.0414864969530996	0.5827702134800091	0.1110165284491842
0.1259733568347577	0.4998918538247012	0.1109957014715370
0.1672270064750818	0.5831945728279023	0.1114877191259750
0.2514799171657828	0.5005224177016168	0.1212191599144324
0.2931502748834644	0.5839370780168851	0.1212080966250119
0.3770123487716369	0.5019368983301785	0.1458800538861613
0.4208309617445157	0.5876350750732774	0.1438314456920507
0.6283587649606430	0.4932006476534468	0.1566417702998859
0.7509641158174770	0.4973017226384535	0.1278740802585232
0.7901125054324376	0.5806292575708984	0.1337575543549240
0.8754575821699022	0.4988855277567192	0.1141124903024369
0.9157734034597884	0.5821998590298624	0.1184081233900492
0.9991718324764272	0.6240881980353077	0.1146733900482205
0.0401081281348716	0.7073639613269649	0.1150357081813303
0.1249087604663542	0.6245002207779803	0.1099948273354927
0.1658418233443304	0.7077552175358789	0.1085989029243880
0.2507877168865239	0.6250187140496924	0.1139184524430880
0.2914502137101083	0.7082462337997762	0.1094045721804900
0.3768714494272084	0.6264633079609666	0.1271845477631732
0.4168274283815427	0.7094861247714630	0.1167300675393037

0.5412157333617246	0.7117674672723321	0.1282605518636947
0.7454465207990028	0.6195508269993648	0.1470881921440118
0.7854457901467410	0.7046539321642024	0.1391800722504854
0.8732997044133568	0.6233486029256671	0.1260237964395352
0.9138894534593670	0.7067635730125219	0.1261740723247385
0.9976403335294529	0.7485722967308308	0.1193766367926999
0.0398034433880634	0.8319224258307890	0.1161634533298181
0.1236607610785819	0.7490865942419905	0.1103350791682626
0.1653449185720752	0.8323771203471344	0.1081470131514718
0.2492492597700296	0.7494581389778562	0.1059831775046590
0.2907122028845257	0.8327099939952628	0.1017562999242511
0.3747901025474615	0.7502136759959378	0.1080386671802657
0.4161544032971269	0.8333212429534020	0.0995282870255481
0.5000857289966041	0.7519655687689636	0.1135008498670707
0.5415526127739689	0.8343907013813475	0.1000439989432695
0.6254360365337610	0.7540061655248238	0.1225759463355785
0.6662920977915286	0.8350744480592167	0.1061783214709079
0.7456028669994765	0.7496394798767854	0.1326664665879357
0.7898614742780607	0.8326734816270073	0.1179758878636727
0.8701658080115643	0.7470344341817288	0.1296278828322918
0.9136620704098269	0.8308679237485775	0.1216517954673607
0.9975872945501307	0.8730161576279858	0.1165561937532697
0.0400720892266950	0.9567190992884812	0.1112211831129901
0.1233394199352550	0.8737213716687082	0.1106656562534347
0.1653612862037254	0.9570584815754221	0.1079605913289360
0.2488090926968142	0.8741161348616153	0.1029397973602733
0.2906388651605378	0.9573622762517578	0.1009314897889879
0.3741720824720471	0.8744807791318847	0.0974393956374881
0.4159325677109558	0.9577009342891120	0.0936191822087080
0.4994489554786702	0.8751488702833091	0.0951529701471148
0.5413411165112654	0.9582017472981621	0.0894335270671328
0.6246197257966587	0.8760073822250840	0.0972127163070736
0.6667984502347845	0.9588388563986214	0.0912926787282083
0.7495363798084018	0.8753883698053768	0.1075061749804854
0.7916451386516007	0.9583142048270883	0.1002106199699000
0.8732231367640269	0.8736588210013970	0.1165320877155816
0.9156914144020927	0.9571814975815317	0.1089178700821994
0.6185932021870136	0.6471604164368254	0.3184890995742862
0.6635537156977334	0.6275508295902698	0.3774491148676259
0.6659175141549042	0.5569567842040308	0.3710893485438685
0.7111661251284956	0.5409778462050668	0.4289044125646578
0.7556399840386895	0.5944025625452573	0.4957526639450336
0.7534764484090843	0.6640065809173445	0.5044587872837407
0.7083715982284777	0.6804840687523590	0.4468831031914923

0.5887320944864566	0.6978979099192910	0.3504373965834511
0.5936619978912079	0.8204582141006342	0.3408146322745817
0.1162729821353430	0.2366488623646042	0.3950199464726631
0.1969340195044468	0.3058587702381420	0.4107196106377640
0.2287313978759903	0.3709487612810076	0.3537316627564726
0.3045436615832172	0.4334630841401453	0.3647999902397889
0.3527988571178333	0.4330998306242589	0.4338433415853652
0.3205588729731968	0.3686454364707119	0.4926329526683676
0.2444940150747800	0.3067656857075474	0.4809135938205323
0.1193259620676223	0.1690096691404503	0.3481786936505369
0.5001787677620821	0.4930244908628110	0.2194851781643790
0.6666134489585823	0.5744883717477005	0.1645540322782981
0.5023790750128819	0.6331801252256178	0.1513624482152421
0.6661121935937623	0.7159905860042580	0.1396840192581280
0.4301003919376731	0.4921567644557654	0.4411665251800708
0.5900306681452856	0.6126074502110480	0.1929480170419725
0.7085049083541185	0.7356805187222210	0.4534489079544249
0.7881751408728191	0.7061429233184342	0.5561434907435699
0.7914983951456753	0.5819236840225505	0.5406584897557943
0.7116957080294520	0.4861700954657594	0.4220543025160474
0.6303749553037802	0.5139094731066118	0.3209867520978349
0.5317081021874748	0.7932277186159057	0.3219412531806922
0.6288009333311675	0.8755908645585632	0.3050167686508471
0.5991371046538359	0.8302975393332029	0.4131129306566145
0.0813885599189724	0.2538709125789650	0.3534132550121116
0.2212761033388649	0.2581410114623637	0.5282974543428682
0.3557028871320403	0.3676323149205977	0.5483875836967955
0.1931864872890444	0.3727730313145892	0.2989598673681594
0.3273034351904311	0.4831020814296194	0.3191405541665518
0.0848460943947539	0.2151593159422069	0.4589201345761141
0.4470070398798876	0.5463270247653540	0.4163864098728399
0.4583155044291504	0.4941855659930333	0.4984312534501473
0.1520157059583402	0.1486359489002474	0.3895751567529078
0.1508832972424470	0.1896168039833608	0.2847119512295221
0.0142812681009632	0.0831159329752015	0.3799280461874543
0.5342119635532516	0.6756105477094756	0.4040578429905730
0.6251878999884387	0.7711160435616778	0.3147474364027565
0.0434235304275693	0.1050888821976157	0.3250697332708757

Total energy (E): -1415.53114265 eV
Temperature (T): 333.0 K
Zero-point energy E_ZPE : 199.915 kcal/mol 8.669125 eV
Thermal correction to U(T): 216.107 kcal/mol 9.371288 eV
Thermal correction to H(T): 216.107 kcal/mol 9.371288 eV
Thermal correction to G(T): 183.525 kcal/mol 7.958417 eV

Entropy S : 409.373 J/(mol*K) 0.004243 eV/K
Entropy contribution T*S : 136321.318 J/(mol) 1.412871 eV

RhN3P SA (pyridinic N): TS2 (imaginary frequency: 288.177487 cm^{-1})
1.00000000000000
19.7297992706000009 0.0000000000000000 0.0000000000000000
-9.8648996353000005 17.0865073798999987 0.0000000000000000
0.0000000000000000 0.0000000000000000 15.0000000000000000
C P N Rh H O
139 1 4 1 19 3
Direct
0.9994089690305034 0.9991203187301727 0.1089098546061657
0.0417997274073837 0.0827492624913356 0.1062975727206698
0.1239644587479556 0.9988077744926208 0.1091680838686993
0.1662621637841352 0.0824236439772325 0.1083301633292688
0.2491743414208041 0.9989990705996349 0.1039624624730202
0.2911836422730875 0.0822998519230387 0.1050919786207108
0.3743562583206380 0.9992325009160200 0.0953689509879355
0.4163862207884367 0.0824630101711054 0.0962661762755530
0.4997477298004208 0.9996316540143267 0.0884190157339797
0.5418210714084766 0.0827640759764975 0.0884614488897119
0.6252785743618412 0.0002892108737180 0.0870504004169252
0.6673560555611678 0.0834461889665747 0.0862871972600762
0.7506037578034854 0.0003574224857600 0.0927316322430766
0.7926140286349249 0.0835156559244944 0.0908660646597180
0.8752999637648254 0.9998276581317421 0.1022983098167013
0.9175135102509258 0.0833101577001912 0.0994051730641903
0.0008845492422778 0.1250248339626800 0.1030994645217616
0.0430749424300797 0.2085937025149111 0.1042912007573143
0.1251646894652853 0.1245249160498604 0.1079553931066093
0.1673923090044461 0.2080797501270263 0.1102357111951953
0.2498183297904604 0.1241849786705528 0.1088266631559680
0.2920276510632048 0.2075506087166635 0.1144803462596426
0.3747081080364366 0.1239921387708734 0.1029932717545803
0.4167613553646305 0.2068741869318518 0.1102868892021175
0.4998895576937412 0.1238516930838928 0.0932922250787307
0.5420448459226963 0.2067501000436864 0.0989284303578405
0.6253941381910846 0.1246020469394825 0.0875750701500135
0.6674873722813681 0.2076939894638785 0.0919679858556458
0.7508807595408374 0.1250689569491305 0.0883580564664705
0.7928710492340156 0.2082939719580792 0.0913766192946203
0.8760742109522343 0.1251321362444088 0.0949587674101253
0.9180437805940580 0.2084994167334696 0.0966086472664256

0.0014575399896745	0.2502382625664953	0.1017968177875604
0.0433802766518852	0.3336581321685564	0.1051608330187455
0.1265252265472375	0.2504626687531512	0.1094761408848840
0.1687853856809275	0.3341370649860154	0.1143329698007649
0.2506941518552047	0.2496215141076936	0.1157485890662758
0.2926178422758011	0.3328398795457528	0.1248888984735231
0.3755102336430544	0.2488956503552151	0.1198483706319569
0.4173841577688451	0.3310490093466729	0.1362450186372346
0.5002375013463630	0.2474792614828487	0.1090955118466889
0.5426164707176911	0.3292185777746290	0.1232806097156119
0.6255996349837755	0.2486544360658414	0.0986096873809892
0.6676965890759287	0.3313774683809310	0.1086922296277811
0.7509849965749931	0.2495647597180958	0.0936351535301616
0.7928193446480239	0.3327185810283126	0.1003975300893906
0.8762953323601201	0.2500457387922984	0.0952627189639846
0.9180646920593498	0.3332967224658133	0.0997364490606914
0.0014662307751470	0.3751183787477216	0.1038829398058224
0.0429696091564135	0.4584069194167459	0.1075921625868316
0.1267396540559296	0.3754966839078506	0.1113818502461321
0.1684415947908628	0.4588788333782841	0.1141914897274508
0.2521595103068230	0.3760907332457196	0.1235299929146536
0.2942368838937898	0.4602711881102217	0.1298598095888322
0.3749906792714073	0.3731059520217759	0.1403335214208521
0.4123778902868565	0.4549300341391013	0.1600165228072672
0.5014244478105951	0.3690517661890214	0.1434355924239876
0.5464729732034418	0.4471212704994655	0.1707746377654470
0.6259935015147086	0.3714409831674430	0.1232088552647310
0.6690930418904759	0.4538680363902812	0.1382609713928052
0.7510994555241661	0.3737177090758708	0.1082563608988196
0.7928941754036198	0.4568086174772144	0.1171436386095756
0.8762470375253621	0.3747179975845543	0.1022429081010616
0.9177236330224265	0.4579443291386908	0.1081205898399901
0.0009758233790562	0.4998204813513022	0.1090006932350321
0.0418611515366805	0.5830555763667122	0.1118521753809569
0.1263702724111032	0.5002117188636030	0.1110573210040995
0.1676366666687903	0.5835093698389466	0.1116310847073699
0.2518382265875265	0.5009303684020302	0.1215612106890043
0.2936058929068814	0.5842910130919712	0.1207674067417381
0.3772259120566354	0.5023528359560768	0.1465729665022515
0.4214833530817540	0.5882870220936011	0.1447690959634549
0.6293841141317206	0.4932891507487191	0.1626697126956949
0.7515001283816846	0.4974706159086846	0.1309087365415021
0.7903536454449831	0.5807697934862357	0.1371830009907218
0.8758448633245177	0.4991969103456576	0.1146819340037143

0.9161043584144718	0.5824677149310687	0.1197064049234931
0.9995013577554264	0.6243315596823751	0.1158471727299694
0.0403913045084397	0.7075938486258220	0.1161227498538334
0.1252945214486778	0.6247937230342433	0.1108193978515725
0.1662228560222385	0.7080243650140561	0.1093665867252719
0.2512035481303453	0.6253597929611762	0.1134876173229847
0.2918240176753331	0.7085336035795448	0.1087353206337781
0.3773995185304152	0.6268651801741042	0.1268565105742012
0.4171843428460435	0.7098149629222962	0.1161814479828705
0.5414529342398183	0.7123886221936744	0.1314535189702677
0.7456435860204277	0.6194885287446164	0.1520750720283917
0.7856846599445853	0.7047969055858984	0.1426020194818922
0.8735854487585408	0.6235406719387533	0.1281716081843330
0.9140895413556581	0.7068603369928793	0.1278376895377906
0.9978358431464848	0.7487067905492647	0.1202779736474259
0.0400176068711166	0.8320257198882621	0.1165409891485594
0.1239739263826327	0.7493119946015344	0.1111000236885546
0.1656420488548868	0.8325400275464748	0.1083929533100106
0.2496217889677394	0.7497284125065694	0.1059768517349090
0.2910775038356722	0.8329432801044370	0.1013889960769201
0.3751110015732769	0.7504656294257028	0.1068738146489134
0.4164701554387426	0.8335216667750849	0.0982084736065844
0.5004657628103528	0.7523656962988442	0.1142392806337935
0.5419394927363373	0.8347626625198410	0.1003120772484906
0.6260838775622315	0.7548499324003642	0.1268946690507284
0.6667063545681087	0.8355959233913112	0.1087034485460891
0.7458629177303854	0.7500791927376935	0.1357064236667665
0.7901163542553803	0.8328863136640642	0.1193288020162043
0.8702035871371837	0.7470500679752891	0.1315394625082597
0.9138151224881310	0.8309601582449131	0.1223599103103785
0.9977257701288972	0.8730619739310724	0.1169083967921898
0.0402797071426331	0.9567650724323190	0.1116935851117659
0.1235633012371010	0.8738325848796874	0.1107920318870587
0.1656359195085181	0.9572112733338664	0.1080101463345448
0.2491143685344359	0.8742839690849760	0.1028377869591598
0.2909378703282798	0.9575323123141031	0.1003445281458306
0.3745189613770045	0.8746877186259950	0.0963182722812107
0.4162675922072735	0.9579233315927713	0.0923429220540349
0.4998220413273192	0.8754081089664182	0.0941719939495788
0.5417157238006114	0.9584547584302544	0.0883418606429367
0.6250127293926077	0.8764268249633594	0.0981334686975923
0.6671667207909174	0.9591183161172592	0.0910108852260328
0.7499525844258396	0.8757353277491911	0.1085426084155453
0.7920347876098297	0.9585910378390252	0.1000127961394145

0.8734886915217444	0.8738091392668096	0.1168819771430012
0.9159548609460832	0.9573380692150595	0.1089806186859519
0.6010411652723342	0.6327791345001513	0.3907525251000459
0.6680356527637749	0.6318432813018987	0.4264375438513526
0.6856332908716219	0.5728989253079351	0.4015578314537021
0.7496203347004501	0.5713754397069053	0.4377059589609362
0.7970047126264891	0.6271340533301429	0.5014563771767876
0.7794244301880823	0.6844608356736801	0.5286231747153434
0.7165068025394790	0.6875453130400545	0.4911423506556953
0.5904810024788326	0.7023048303340583	0.4047174980593817
0.6260488848673109	0.8293628577539581	0.3550118753502808
0.1900274063705852	0.3000211895280999	0.3928018486315632
0.2698032193340356	0.3642092048196061	0.4209317835690610
0.3102682949566887	0.4344524080572610	0.3722580311524787
0.3844638829959386	0.4928315976075030	0.3966809369582411
0.4243114731545091	0.4833733110167580	0.4714500529137167
0.3829857469305376	0.4106863076857944	0.5192397526466790
0.3078535018974891	0.3543335669425826	0.4954150435867132
0.1931645996654515	0.2292998702845327	0.3542867643644808
0.5018592116062220	0.4960086742705836	0.2120473015367513
0.6670975745976769	0.5744164738958506	0.1727118342818505
0.5028377652593071	0.6342094378620048	0.1550346929170970
0.6665809062276680	0.7167144871320934	0.1440101767745999
0.4945079114498204	0.5428851619714498	0.4959348682719452
0.5900471581430563	0.6135418980791817	0.1953360352327944
0.7025200974580772	0.7316611095112038	0.5139157659017226
0.8149371957725587	0.7268082501285935	0.5796830606460260
0.8472286495547267	0.6257541122453786	0.5298071586052217
0.7631881645651164	0.5266183988233684	0.4163327029383847
0.6491368293547403	0.5296152216523441	0.3518587825076684
0.5660844072891386	0.8157772463973915	0.3397869443998315
0.6675314280936505	0.8719418020870418	0.3074673017936693
0.6402678572481357	0.8512900372302775	0.4236622746380181
0.1648757330821948	0.3213346074544708	0.3420871826294779
0.2772002146918863	0.3007370703323130	0.5349037322104573
0.4112085086331440	0.4016487551747122	0.5770707875217619
0.2820552654062367	0.4430657859341764	0.3141860110688510
0.4140247972091892	0.5477572843586843	0.3596578166465225
0.1497333554290259	0.2799851322228519	0.4503981759110003
0.5559989129565648	0.5828438516964359	0.3550532427140519
0.5189044038279410	0.5260440463262704	0.5456750969419689
0.2181021091632358	0.2066460903084207	0.4041656724288001
0.2319243035212153	0.2477690637163005	0.2958693713511973
0.0838042040894084	0.1469666540097526	0.3739331394704311

0.5509692983946493 0.7089389443832038 0.4626954928730964
0.6329900843781822 0.7600001732557595 0.3444869083872463
0.1187747634774977 0.1685693578980008 0.3230427096567541
Total energy (E): -1413.76862614 eV
Temperature (T): 333.0 K
Zero-point energy E_ZPE : 198.356 kcal/mol 8.601535 eV
Thermal correction to U(T): 214.136 kcal/mol 9.285834 eV
Thermal correction to H(T): 214.136 kcal/mol 9.285834 eV
Thermal correction to G(T): 182.125 kcal/mol 7.897698 eV
Entropy S : 402.206 J/(mol*K) 0.004169 eV/K
Entropy contribution T*S : 133934.701 J/(mol) 1.388136 eV

RhN3P SA (pyridinic N): MS2
1.00000000000000
19.7297992706000009 0.0000000000000000 0.0000000000000000
-9.8648996353000005 17.0865073798999987 0.0000000000000000
0.0000000000000000 0.0000000000000000 15.0000000000000000
C P N Rh H O
139 1 4 1 19 3
Direct
0.9996328708898975 0.9994967896192155 0.1079850286693765
0.0420345635580845 0.0831648625693858 0.1054962594269091
0.1241691692918721 0.9992413721566213 0.1090213586779465
0.1664652157312996 0.0828599692417170 0.1081257569275729
0.2493526731173906 0.9994859957975026 0.1032197131237926
0.2913609916682930 0.0827759911907822 0.1045449755563689
0.3745389433706186 0.9997901624755827 0.0945788028769633
0.4165802372411632 0.0830119877942396 0.0955753471502436
0.4999496079553298 0.0002459756996736 0.0886771221701630
0.5420184949864472 0.0833376014082206 0.0885122566748984
0.6254851018821074 0.0008975579790198 0.0881880019315776
0.6675771743118026 0.0840007021120846 0.0871718793703844
0.7508424234242937 0.0008470195234961 0.0929825950207433
0.7928429311730495 0.0839917830705362 0.0913805253264569
0.8755383352917949 0.0002253067769113 0.1011795261963963
0.9177375877850141 0.0837140530146433 0.0988541925921084
0.0011198293719546 0.1254275880606698 0.1022953041862041
0.0433499133119957 0.2090245094530009 0.1033638270677410
0.1254089691186364 0.1249738581254204 0.1074270083198645
0.1676297696057560 0.2085141675193594 0.1096420367249824
0.2500141313201478 0.1246308961586444 0.1086357144133477
0.2921913032260007 0.2079733949243272 0.1145580505127582
0.3748779228515233 0.1244951321605058 0.1024083987424749

0.4169142136153197	0.2073256275219668	0.1102005879538159
0.5000920846485328	0.1244149563327336	0.0928597984794767
0.5422612874793786	0.2072738822803497	0.0987864350848500
0.6256347413058569	0.1251520720056396	0.0879603981890890
0.6677417324828342	0.2081942040384607	0.0923182339303710
0.7511091690706364	0.1255547870189769	0.0890558896571440
0.7931050654295446	0.2087523860162634	0.0916780451237432
0.8763086407550444	0.1255547867559434	0.0948530239170951
0.9183016810595175	0.2089335585412188	0.0962241281158173
0.0017343735004419	0.2506572142890059	0.1009805412587777
0.0436843485223826	0.3340835104621526	0.1040505523615442
0.1267945760376165	0.2508684553637415	0.1084942523795848
0.1690618299174171	0.3345178070610994	0.1131811846770508
0.2508389871243865	0.2499776272880971	0.1156922139431464
0.2927875045693656	0.3331588690027340	0.1249307757493603
0.3756980631740063	0.2493048459886354	0.1203056149215797
0.4175760905464816	0.3313468841786099	0.1375717058085817
0.5004309222533802	0.2479470107138440	0.1092866162951508
0.5427892702077399	0.3295324242328030	0.1244391774801375
0.6258229792107572	0.2491113911303437	0.0988621184603707
0.6679362244179505	0.3317622786527661	0.1092350156733513
0.7512381493105068	0.2500208861049186	0.0940206647589325
0.7931070434575135	0.3331455048612827	0.1006977656615567
0.8765685961060959	0.2504844292784393	0.0950849374356241
0.9183704617448706	0.3337533007175941	0.0993380719102860
0.0017689721435193	0.3755485735397386	0.1029933163505979
0.0432707201152470	0.4588218189295740	0.1066627080463955
0.1270578079867954	0.3758958527362714	0.1098907679823519
0.1687724370130194	0.4593170214480944	0.1123861148873669
0.2524186074038967	0.3764195934379879	0.1227353821652534
0.2945593408776550	0.4606572219434370	0.1282769397979661
0.3751599857726431	0.3733968619120893	0.1410464795209724
0.4126630784344527	0.4553711689315324	0.1594309431309267
0.5016158219802491	0.3692646886141914	0.1454547414983302
0.5467443435891790	0.4472797032822970	0.1728557809998237
0.6262069979232073	0.3717212496441951	0.1241808832245050
0.6694189074492894	0.4541019553338819	0.1394198566099707
0.7513957136579781	0.3741041838710765	0.1088397423037766
0.7932507062058043	0.4571411084223809	0.1180273683870251
0.8765397912373393	0.3751346503922671	0.1022961132255377
0.9180446745506333	0.4583339799815831	0.1085307978619101
0.0012864178490335	0.5002261792413958	0.1088809211322579
0.0421491875490942	0.5834710329241852	0.1119734723390098
0.1266640780071525	0.5006297756155080	0.1096175558041814

0.1678687390898930	0.5839277409196345	0.1104985116527213
0.2521527860229871	0.5013462161737681	0.1194716466174938
0.2937804174201205	0.5846842632237744	0.1184817862809222
0.3775308426267665	0.5026773974235492	0.1444898366558327
0.4217947352251075	0.5886999954054924	0.1404903344283568
0.6298182378785003	0.4935285424390048	0.1639928246047515
0.7518335775421863	0.4977594139060039	0.1317186824251211
0.7906984217149544	0.5810494484966313	0.1373704211004149
0.8761901882810803	0.4995462295721610	0.1156323507329175
0.9164680059609445	0.5828149718280722	0.1207530233613629
0.9998255076129139	0.6247219565826567	0.1166164006152402
0.0406902692641689	0.7080169314884255	0.1168978755062644
0.1255391957727515	0.6252378199072337	0.1104555230216352
0.1664252395457302	0.7084670688857363	0.1089967868917732
0.2513812529980788	0.6257844218047931	0.1119281599562789
0.2919651357481013	0.7089992733245880	0.1075107304235041
0.3775594058106590	0.6272718734389935	0.1236489130068114
0.4172469747973843	0.7102354445530124	0.1134124803092583
0.5413858124047368	0.7124109181131483	0.1246293638658077
0.7457934711722528	0.6196646244141391	0.1504506559073277
0.7856747544796021	0.7048095572295054	0.1385177092087803
0.8739523792966408	0.6238777900506137	0.1288630522107568
0.9143789928771201	0.7071928005947987	0.1282004816694458
0.9981283090589622	0.7491159967538420	0.1211094135881025
0.0402654857372624	0.8324352756178295	0.1168788497217783
0.1242176779403077	0.7497700309394915	0.1111376537252532
0.1658534800934119	0.8329967770443409	0.1080642722784367
0.2497841626863197	0.7502016995733140	0.1051772189060499
0.2912293771403850	0.8334271474465432	0.1005457068194667
0.3752010123233327	0.7510008174400378	0.1053775018628803
0.4166113897000105	0.8340830585825589	0.0975071466982265
0.5004441079581009	0.7527922298982941	0.1105886730196881
0.5420531717025849	0.8353987906551114	0.0990184198553320
0.6262153467556726	0.7547616438574355	0.1187804660021520
0.6669216767299961	0.8360753444398000	0.1048505568869952
0.7458749008384054	0.7498871103074917	0.1283253083988710
0.7903528637694353	0.8331087711588894	0.1149246769331120
0.8702967054622647	0.7472317966803738	0.1298042104000193
0.9139761933100962	0.8312282477514094	0.1206832649605340
0.9979164603060903	0.8734155540709678	0.1165229925647756
0.0404664927263234	0.9571392786720367	0.1112620424414694
0.1237747497360178	0.8742607792466290	0.1107459020956388
0.1658263384883232	0.9576588587142426	0.1076794468573758
0.2492790220057179	0.8747703704543354	0.1020129259951101

0.2910902977678455	0.9580211652455833	0.0994103810052705
0.3746794012010766	0.8752347444713799	0.0957135788200298
0.4164428286345937	0.9584950520668216	0.0919536351684478
0.5000112148512074	0.8760690967793819	0.0941076807984463
0.5419273491921867	0.9591029600745453	0.0891090948967070
0.6251592334272649	0.8770619036810943	0.0972421714464150
0.6673514681370858	0.9597382861059321	0.0916979504090254
0.7501980531198053	0.8760379286728281	0.1051225908658911
0.7923011181968906	0.9590581370823672	0.0987538006622198
0.8737450419256209	0.8741159384694306	0.1144139128336055
0.9161987241017481	0.9577067933110059	0.1074621163476444
0.5912373372081963	0.6198425901597037	0.4412664649593110
0.6719206367806925	0.6317322215575237	0.4625185763613515
0.6965777909960134	0.5840980083566953	0.4209080274936021
0.7684230671510361	0.5909269571021439	0.4430894002256229
0.8163629628882121	0.6454843744303909	0.5071338303380755
0.7921094192383136	0.6930765561777629	0.5488558245558347
0.7200093778633903	0.6860400341808742	0.5268591834195600
0.5914106314444706	0.6978450582665259	0.4463103903741649
0.6363297816130626	0.8185847709664040	0.3698380249901035
0.2238150079106715	0.3289785122776467	0.3939217684593502
0.3026009902182341	0.3931719870530964	0.4255201745735158
0.3449125594664552	0.4630575250493342	0.3779648473537027
0.4208490700549306	0.5199661125213759	0.4019841121717963
0.4577632682559860	0.5079151218901197	0.4758332037686194
0.4140687415511706	0.4390324780870278	0.5265246709629834
0.3385733429241219	0.3833486165142548	0.5016328618363833
0.2283843963064378	0.2577903046654716	0.3601485019749407
0.5031022263359959	0.4984286595958582	0.2097140531768676
0.6674490392770334	0.5747666158130322	0.1719227473693242
0.5031755674516116	0.6343854250401975	0.1479482235715308
0.6663898805761246	0.7158499734080889	0.1312350954403931
0.5340532781211788	0.5603696808762871	0.4995092418781978
0.5899381844763544	0.6138218167455013	0.1857048581171155
0.7009710526851446	0.7230620425404678	0.5597519959431243
0.8293283830384529	0.7358753242245019	0.5986941768210868
0.8726534591139692	0.6509832074309373	0.5242856789385951
0.7870751649397981	0.5535304567613876	0.4104505261104533
0.6591965288168139	0.5416263736573427	0.3708907964385759
0.5803475841881430	0.8139300554677197	0.3509107160416984
0.6808101418967057	0.8507593641869450	0.3189236669974917
0.6546910661911087	0.8475740740804560	0.4349242952173704
0.2020843372928803	0.3503217173484683	0.3393778294305259
0.3068067522417113	0.3304212872016910	0.5420140968787707

0.4405443928449768	0.4293244672604296	0.5855101886062725
0.3179982087190797	0.4736338594616862	0.3200037517597975
0.4512512393841708	0.5737709172942863	0.3631941636065630
0.1801866687753006	0.3098998027871461	0.4481163472251876
0.5776947158388579	0.6004170197657941	0.3708683827177013
0.5558098273360984	0.5465028217674298	0.5531806923008143
0.2494399399701685	0.2347195606511109	0.4140778847830137
0.2709115187178486	0.2756897545765242	0.3053237558868270
0.1175179135724729	0.1769149455175360	0.3716341038841229
0.5642247541734269	0.7186338681157984	0.5061998753887479
0.6282663638941290	0.7416395400108139	0.3731673502390970
0.1559632475138070	0.1971202242442545	0.3238332465401971

Total energy (E): -1416.20890810 eV

Temperature (T): 333.0 K

Zero-point energy E_ZPE : 201.902 kcal/mol 8.755287 eV

Thermal correction to U(T): 218.062 kcal/mol 9.456047 eV

Thermal correction to H(T): 218.062 kcal/mol 9.456047 eV

Thermal correction to G(T): 184.948 kcal/mol 8.020092 eV

Entropy S : 416.062 J/(mol*K) 0.004312 eV/K

Entropy contribution T*S : 138548.529 J/(mol) 1.435955 eV
